# Glucose-Lowering Medication Classes and Cardiovascular Outcomes in Patients With Type 2 Diabetes

**DOI:** 10.1001/jamanetworkopen.2025.36100

**Published:** 2025-10-15

**Authors:** Romain Neugebauer, Jaejin An, Sarah Krahe Dombrowski, Caryn Oshiro, Andrea Cassidy-Bushrow, Lisa Gilliam, Gregg Simonson, Andrew J. Karter, Richard Bergenstal, Holly Finertie, Maher M. Yassin, Greg Knowlton, Sharon R. Lin, Wendy Dyer, Noel Pimentel, Keanu Izadian, Julie Schmittdiel, Tainayah W. Thomas, Stephanie A. Hooker, Margaret B. Nolan, Eric Wright, Lindsey Aurora, Luis A. Rodriguez, Jasleen Kaur, Alyce S. Adams, Mark J. van der Laan, Patrick J. O’Connor

**Affiliations:** 1 Division of Research, Kaiser Permanente Northern California, Pleasanton; 2 Department of Research and Evaluation, Kaiser Permanente Southern California, Pasadena; 3 Center for Pharmacy Innovation and Outcomes, Geisinger, Danville, Pennsylvania; 4 Center for Integrated Health Care Research, Kaiser Permanente Hawaii, Honolulu; 5 Department of Public Health Sciences, Henry Ford Health, Detroit, Michigan; 6 Michigan State University Health Sciences, Detroit; 7 Department of Pediatrics and Human Development, College of Human Medicine, Michigan State University, East Lansing; 8 Endocrinology and Internal Medicine, Kaiser Permanente Northern California, South San Francisco; 9 HealthPartners Institute, International Diabetes Center, Minneapolis, Minnesota; 10 HealthPartners Institute, Minneapolis, Minnesota; 11 Department of Epidemiology and Population Health, Stanford University School of Medicine, Stanford, California; 12 Division of Cardiology, Department of Medicine, Henry Ford Health, Detroit, Michigan; 13 Department of Endocrinology, HealthPartners, St Paul, Minnesota; 14 Department of Health Policy, Stanford University School of Medicine, Stanford, California; 15 Division of Biostatistics, School of Public Health, University of California, Berkeley

## Abstract

**Importance:**

Major adverse cardiovascular events (MACEs) are primary causes of morbidity and mortality in adults with type 2 diabetes (T2D), yet few head-to-head randomized trials have compared the effects of glucose-lowering medications on MACEs, and most observational analyses are limited by inadequate bias adjustment methods.

**Objective:**

To compare the effectiveness of sustained exposure to 4 classes of glucose-lowering medications (sulfonylureas, dipeptidyl peptidase-4 inhibitors [DPP4is], sodium-glucose cotransporter-2 inhibitors [SGLT2is], and glucagon-like peptide-1 receptor agonists [GLP-1RAs]) on MACEs in US adults with T2D using modern causal methods combined with machine learning.

**Design, Setting, and Participants:**

This comparative effectiveness study included adults with T2D who were members of 6 large US health care delivery systems and initiated treatment with 1 of 4 medication classes (sulfonylureas, DPP4is, SGLT2is, and GLP-1RAs) between January 1, 2014, and December 31, 2021. Data analysis was conducted from May 1 to December 31, 2024.

**Exposure:**

New use of a sulfonylurea, DPP4i, SGLT2i, or GLP-1RA based on filled prescriptions.

**Main Outcomes and Measures:**

The primary outcome was MACEs defined as nonfatal myocardial infarction, nonfatal stroke, or cardiovascular death. Analyses were conducted using targeted learning within a trial emulation framework. Heterogeneity of treatment effects was assessed for prespecified subgroups.

**Results:**

This study included 296 676 adults. The cohort for emulating a 4-arm trial included a subset of 241 981 adults (mean [SD] age, 57.2 [12.9] years; 54.3% male) with T2D. In adjusted analyses, 2.5-year MACE risk was lowest in patients with sustained exposure to GLP-1RAs, followed by SGLT2is , sulfonylureas, and DPP4is. Comparing DPP4is with sulfonylureas and SGLT2is with GLP-1RAs, the 2.5-year cumulative risk difference was 1.9% (95% CI, 1.1%-2.7%) and 1.5% (1.1%-1.9%), respectively. Risk differences in patients with vs without atherosclerotic cardiovascular disease (ASCVD) were similar in direction but typically much smaller for patients without ASCVD. Evidence of a benefit of GLP-1RAs over SGLT2is was most pronounced in patients with baseline ASCVD or heart failure (HF), age 65 years or older, or low to moderate kidney impairment but was not found in patients younger than 50 years.

**Conclusions and Relevance:**

In this study, MACE risk varied significantly by medication class, with most protection achieved with sustained treatment with GLP-1RAs followed by SGLT2is, sulfonylureas, and DPP4is. The magnitude of benefit of GLP-1RAs over SGLT2is depended on baseline age, ASCVD, HF, and kidney impairment. These results, along with consideration of cost, availability, and collateral clinical benefits, may inform treatment decisions for adults with T2D.

## Key Points

**Question:**

Do sustained treatments with 4 classes of glucose-lowering medications have differential effects on major adverse cardiovascular events (MACEs) in US adults with type 2 diabetes (T2D)?

**Findings:**

In this comparative effectiveness study of 296 676 US adults with T2D, MACEs varied significantly across 4 classes of glucose-lowering medications: sustained treatment with GLP-1RAs was most protective against MACEs followed by SGLT2is, sulfonylureas, and DPP4is. The benefit of GLP-1RAs over SGLT2is varied in magnitude across subgroups defined by baseline age, atherosclerotic cardiovascular disease, heart failure, and kidney impairment.

**Meaning:**

These findings may help inform clinician and patient selection of glucose-lowering medications to treat T2D when the goal is to optimize cardiovascular outcomes.

## Teaser

This comparative effectiveness study examines major adverse cardiovascular events among patients with type 2 diabetes receiving sulfonylureas, dipeptidyl peptidase-4 (DPP4) inhibitors, sodium-glucose cotransporter-2 (SGLT2) inhibitors, or glucagon-like peptide-1 (GLP-1) receptor agonists.

## Introduction

Major cardiovascular adverse events (MACE), including nonfatal myocardial infarction (MI), nonfatal ischemic and hemorrhagic stroke (cerebrovascular accident [CVA]), and cardiovascular death, are the leading cause of excess mortality in adults with type 2 diabetes (T2D).^1,2^ Thus, a major goal of clinical care of patients with T2D is to prevent cardiovascular disease (CVD) and minimize MACEs.^1,2,3,4^ Results of cardiovascular outcome trials indicated that compared with placebo, most dipeptidyl peptidase-4 inhibitors (DPP4is) were neutral for cardiovascular outcomes,^5,6^ whereas several glucagon-like peptide-1 receptor agonists (GLP-1RAs)^7,8^ and sodium-glucose cotransporter 2 inhibitors (SGLT2is)^9,10^ substantially reduced MACEs in adults with T2D and high cardiovascular risk. However, few randomized trials have enrolled patients with low to moderate cardiovascular risk, and few have directly compared the effects of different glucose-lowering medication classes on MACEs—information needed by patients and clinicians to inform treatment decisions.^11,12^ Prior large comparative observational studies have attempted to directly compare the cardiovascular effects of different agents or classes of agents.^13,14,15,16,17,18^ However, the methodology used in prior studies often threatens the validity or limits the clinical relevance of their findings, due to focusing on intention-to-treat (ITT) analyses despite high rates of treatment discontinuation or crossover events, not accounting for time-varying confounding and attrition bias, making unlikely statistical modeling assumptions, not adjusting for important confounders, and not adequately assessing the heterogeneity of treatment effects (HTE).

In this context, we designed a multisite comparative effectiveness study grounded in a principled causal inference framework^19,20^ and targeted learning^21,22^ (1) to detect differences in MACE risk and (2) to assess HTEs in a diverse population of adults with T2D treated in clinical care settings who were new users of 4 classes of glucose-lowering medications: sulfonylureas, DPP4is, SGLT2is, or GLP-1RAs.

## Methods

This comparative effectiveness study was approved by the Kaiser Permanente Northern California Institutional Review Board, who waived the need for informed consent due to minimal risk to human participants. The study followed the Strengthening the Reporting of Observational Studies in Epidemiology (STROBE) reporting guideline.

### Study Design

This retrospective cohort study emulated several unblinded, head-to-head, 2-arm and 4-arm target randomized clinical trials (RCTs) (eTable 1 in Supplement 1) of initiators of the 4 medication classes (sulfonylureas, DPP4is, SGLT2is, or GLP-1RAs) using targeted learning^21,22^ to account for more than 400 time-independent and time-varying covariates. Primary analyses contrasted the effects of initial and sustained treatment with 1 of the 4 medications studied (per protocol [PP] analyses). Secondary analyses contrasted the effects of initial treatment only (ITT analyses). Sensitivity analyses were conducted to strengthen the reliability and relevance of findings and assess robustness to unmeasured confounding. We adopted a framework^23^ proposed in 2024 on the use of causal language to describe the analytic approach and interpretation of findings.

Each target trial was emulated by constructing a separate cohort using the same general eligibility criteria described herein. Longitudinal data on exposure, outcome, right-censoring, and covariates were assembled on each participant from cohort entry until the earliest of the following: (1) administrative end of study (December 31, 2021), (2) disenrollment from pharmacy coverage or health plan, (3) noncardiovascular death, (4) death from an unknown cause, or (5) 3-point MACE (primary outcome). To address both confounding and attrition bias (ie, selection bias from informative right-censoring), we used targeted learning to estimate the cumulative incidence curves associated with each treatment and to estimate cumulative risk differences (RDs) at 2.5 years of follow-up.

Primary PP analyses required (1) initiation and sustained exposure to one of the compared medications and (2) no initiation of any of the comparator medications. Secondary ITT analyses focused on the evaluation of treatment protocols that only required initiation of the compared medications while disregarding persistence and initiation of comparators. Both PP and ITT analyses were replicated in separate patient subgroups to assess comparative effectiveness for (1) primary prevention of MACEs, (2) secondary prevention of MACEs, and (3) primary prevention of MACEs in patients previously treated with metformin monotherapy. PP and ITT analyses comparing SGLT2is with GLP-1RAs were also conducted in additional subgroups defined by baseline heart failure (HF) status, combination of HF and atherosclerotic CVD (ASCVD) statuses, chronic kidney disease status, age, sex, and race and ethnicity. Effect evaluation within race and ethnicity subgroups was motivated by study stakeholder partners. Patients self-reported race and ethnicity as American Indian or Alaska Native, Asian, Black, Hispanic, Native Hawaiian or Other Pacific Islander, White, other race or ethnicity, multiple races or ethnicities, or unknown.

PP and ITT sensitivity analyses evaluated a modified 3-point MACE outcome that included an expanded definition of cardiovascular death. Because successful RCT emulation with observational data relies on the strong assumption of no unmeasured sources of confounding or attrition bias, sensitivity analyses were conducted to gauge the robustness of findings to plausible levels of missing information (ie, unmeasured sources of confounding or attrition bias) required for this assumption to hold. Other sensitivity analyses were implemented to control for differential exposure to metabolic bariatric surgery or other medications not compared in the emulated RCTs. Finally, all primary, secondary, subgroup, and sensitivity analyses were replicated using an inverse probability weighting (IPW) approach, instead of targeted learning, to estimate the same cumulative incidence curves and RDs and to estimate hazard ratios (HRs).

### Study Setting and Participants

**Figure 1.**
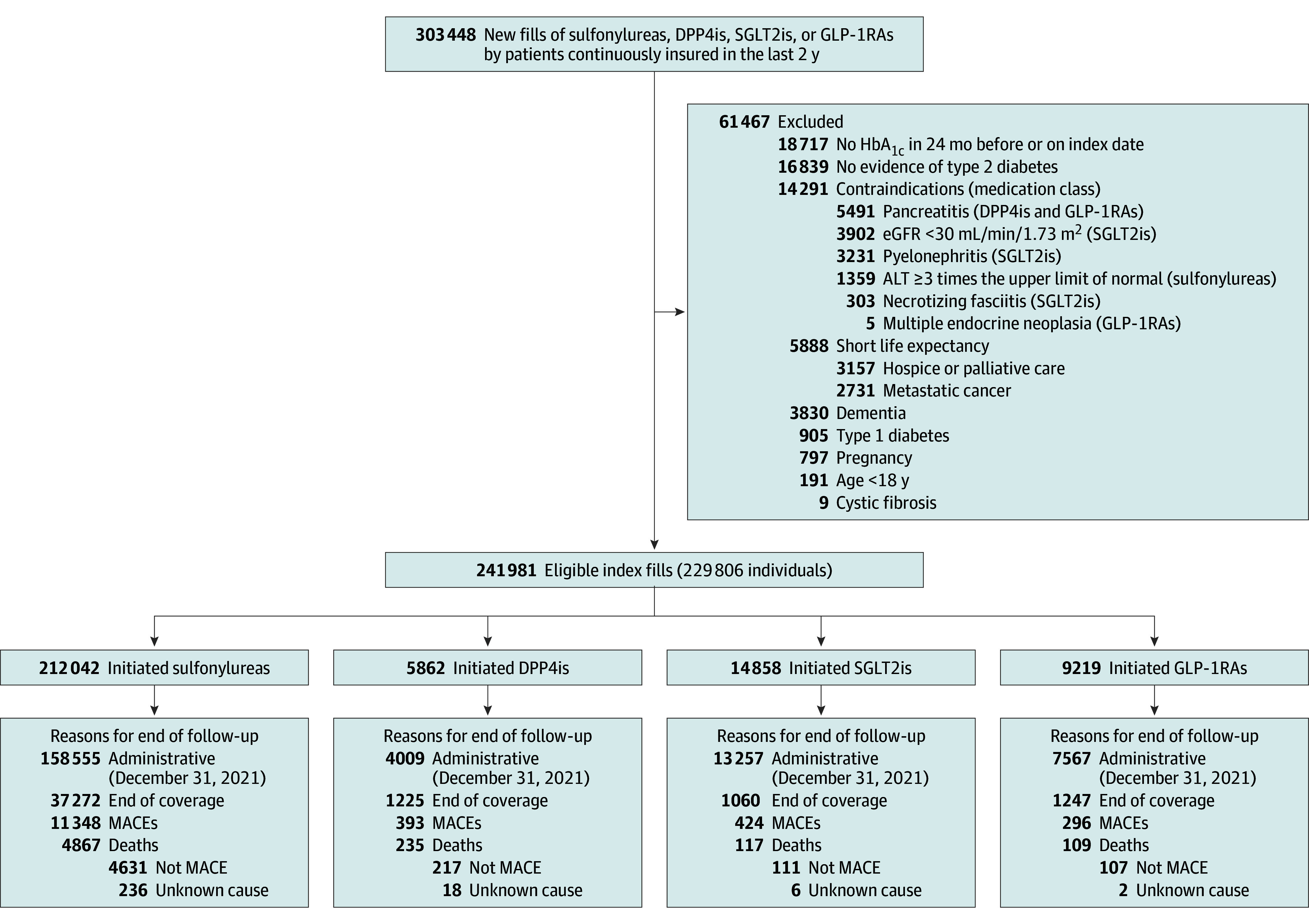
Study Flow Diagram Inclusion and exclusion counts leading to creation of the cohort for emulating the 4-arm randomized clinical trial, along with sample sizes and counts for each observed end of follow-up in each treatment arm. New fills represents the count of patients who initiated 1 of the 4 medication classes compared and for whom there was no evidence of a dispensing for any of these 4 medication classes in the prior 2 years. ALT indicates alanine transaminase; DPP4i, dipeptidyl peptidase-4 inhibitor; eGFR, estimated glomerular filtration rate; GLP-1RA glucagon-like peptide-1 receptor agonist; HbA_1c_, hemoglobin A_1c_; MACE, major adverse cardiovascular event; SGLT2i, sodium-glucose cotransporter-2 inhibitor.

This study included all study-eligible patients at 6 health care delivery systems with integrated administrative and electronic health record (EHR) data who initiated treatment with 1 of the 4 medication classes (sulfonylureas, DPP4is, SGLT2is, or GLP-1RAs) between January 1, 2014, and December 31, 2021. The 6 systems were Geisinger in Pennsylvania, the Henry Ford Health System in Michigan, HealthPartners in Minnesota and Wisconsin, and Kaiser Permanente of Northern California, Southern California, and Hawaii. For each emulated RCT, a separate cohort was constructed of all participants who met all cohort eligibility criteria simultaneously (Figure 1, Table 1, and eFigures 1, 4, 10, 16, 22, 28, and 34 in Supplement 1). The date of cohort entry when all criteria were met simultaneously is referred to as the index date, which is also the start of follow-up.

**Table 1.** Inclusion and Exclusion Counts Leading to Cohort Creation for Emulating 8 RCTs (2-Arm), With Sample Sizes and Counts for Each Observed End of Follow-Up in Each Treatment Arm^a^

Cohort characteristic	Sulfonylureas vs DPP4is	Sulfonylureas vs SGLT2is	Sulfonylureas vs GLP-1RAs	DPP4is vs SGLT2is	DPP4is vs GLP-1RAs	SGLT2is vs GLP-1RAs	Exenatide vs liraglutide	Semaglutide vs liraglutide
No. of new fills	270 653	287 519	283 843	95 438	71 651	94 559	26 445	32 314
No. of exclusions								
T1D	337	498	703	296	510	624	327	363
No T2D	10 668	13 116	14 556	2452	3892	6175	1943	3468
Cystic fibrosis	8	7	8	3	5	1	0	1
No HbA_1c_	15 365	16 064	16 629	9741	10 773	10 223	3348	5080
Age <18 y	98	93	182	12	102	97	88	87
Pyelonephritis	NA	3020	NA	1157	NA	1322	NA	NA
Necrotizing fasciitis	NA	267	NA	130	NA	163	NA	NA
MEN	NA	NA	5	NA	3	1	1	1
Pancreatitis	4996	NA	5114	2020	1088	1916	394	455
eGFR	NA	3652	NA	1192	NA	675	NA	NA
ALT	1348	1392	1354		NA	NA	NA	NA
Dementia	3846	3820	3826	1413	959	1069	151	244
Metastatic cancer	2739	2817	2754	638	414	528	97	121
Hospice care	3295	3055	3306	1091	1024	904	276	376
Pregnancy	806	805	816	10	17	17	7	8
No. of index fills	227 147	238 913	234 590	75 283	52 864	70 844	19 813	22 110
No. of patients in exposure group	220 085 vs 7062	219 957 vs 18 956	221 910 vs 12 680	28 194 vs 47 089	30 165 vs 22 699	49 103 vs 21 741	5959 vs 13 854	6047 vs 16 063
Administrative end of follow-up	164 369 vs 4898	164 230 vs 16 663	165 597 vs 10 384	20 639 vs 42 289	22 168 vs 18 900	43 984 vs 17 694	4602 vs 11 524	5334 vs 13 471
End of coverage	38 225 vs 1408	38 671 vs 1575	38 589 vs 1703	4935 vs 3311	5114 vs 2859	3499 vs 3064	985 vs 1763	509 vs 1951
Noncardiovascular death	5009 vs 251	4913 vs 160	5084 vs 161	758 vs 323	833 vs 247	352 vs 270	101 vs 143	51 vs 165
Unknown cause of death	249 vs 21	250 vs 7	255 vs 6	54 vs 18	63 vs 6	18 vs 10	2 vs 7	4 vs 7
MACE	12 233 vs 484	11 893 vs 551	12 385 vs 426	1808 vs 1148	1987 vs 687	1250 vs 703	269 vs 417	149 vs 469

Abbreviations: ALT, alanine transaminase; DPP4i, dipeptidyl peptidase-4 inhibitor; eGFR, estimated glomerular filtration rate; GLP-1RA, glucagon-like peptide-1 receptor agonist; HbA_1c_, hemoglobin A_1c_; MACE, major adverse cardiovascular event; MEN, multiple endocrine neoplasia; SGLT2i, sodium-glucose cotransporter-2 inhibitor; T1D, type 1 diabetes; T2D, type 2 diabetes.

^a^ Rows contain analogs of the counts presented in Figure 1. In particular, the first row represents the count of patients who initiated 1 of the 2 compared medications (either medication classes or agents) and for whom there was no evidence of a dispensing for any of these 2 medications in the prior 2 years.

### Data Sources

Patient-level EHR data, including demographics, vital signs, clinical characteristics, diagnoses, procedures, medications dispensed, tobacco use, and selected laboratory test results, were used to define cohort eligibility, exposures, outcomes, and covariates. Administrative data were obtained to ensure accurate assessment of cohort eligibility, censoring events, and complete capture of key clinical outcomes including MI and stroke. Death and cause of death data were ascertained hierarchically from the National Death Index, state death indexes, and health plan administrative data.

### Exposure

Pharmacy dispensing data were used to determine exposure to each medication from index date until end of follow-up. On any given day, a patient was considered exposed to a specific medication if that day fell within the coverage period of the patient’s closest prior medication dispensing. We defined the coverage period for each medication dispensing as the interval of time starting on the dispensing date and lasting twice the days’ supply. For a day that did not meet the aforementioned exposure criterion, patients were considered unexposed to the medication unless that day fell within an allowable medication coverage gap of 90 days or less defined by the interval of time between the end of the coverage period from the prior closest dispensing for that medication and the next dispensing for the same medication.

### Outcomes

A MACE was defined as the earliest occurrence after the index date of any 1 of the following 3 events: nonfatal MI, nonfatal CVA, or cardiovascular death. MI and CVA were assigned on the earliest date of a first principal inpatient or first primary emergency department diagnosis code. Cardiovascular death was assigned on the date on which cause of death codes listed in the National Death Index, state death index, or other reliable source were attributed to 1 or more cause of death codes specifically related to MI or CVA. In sensitivity analyses, we considered an expanded definition of cardiovascular death.^24^

### Covariates

We included more than 400 time-independent and time-varying covariates in the analyses, including exposures to glucose-lowering medications not compared in specific emulated RCTs such as metformin and insulin.^24^ Subject-matter expertise drove the selection of covariates. In addition to covariates defining patient subgroups of interest, covariates that could plausibly affect treatment decisions, the occurrence of censoring events, or the outcome were included in the adjustment set. In particular, the calendar year of the index date, major cardiovascular risk factors (age, sex, blood pressure, lipid levels, and kidney function), glycated hemoglobin, smoking status, and exposure to statins, angiotensin receptor blockers, and angiotensin-converting enzyme inhibitors were included in the adjustment set.

### Statistical Analysis

Primary (PP) analyses of new and sustained users, secondary (ITT) analyses of all new users, and sensitivity analyses are detailed in eAppendix 1 in Supplement 1. For each emulated target trial, we identified a separate cohort of eligible patients and their index dates before assembling a longitudinal dataset^25^ with measurements updated every 30 days for each cohort member using the LtAtStructR R package.^26^ In this dataset, covariates that were not measured at baseline or during follow-up were encoded using the missingness indicator approach.^27,28^ This approach consisted of creating indicators of covariate measurement at each time point (ie, a 30-day interval); replacing unobserved baseline values with the mode and mean of observed values for categorical and continuous covariates, respectively; and replacing unobserved time-varying values with the last observed value, if any, and otherwise the imputed baseline value. The resulting candidate covariates and their associated measurement indicators were considered together for bias adjustment. The assumption underlying this approach is described in eAppendix 1 in Supplement 1.

The same dataset was then used to implement both PP and ITT analyses^29^ under nonparametric marginal structural models^25^ using targeted learning^22^ to estimate the cumulative incidence curve for each treatment arm of the emulated target trial. Two-arm contrasts of these curves defined the primary study effect measures: the RD at 2.5 years of follow-up (with the corresponding number needed to treat [NNT] defined as the inverse of RD) and the average risk difference (ARD) over 2.5 years of follow-up (ie, the difference in the area under any 2 cumulative incidence curves).^30^ Targeted learning is a general estimation procedure that combines a doubly robust estimation approach known as targeted minimum loss–based estimation^21,31^ (TMLE) with machine learning to estimate propensity scores and a sequence of outcome regressions.

For both PP and ITT analyses, outcome regressions were estimated using LASSO,^32^ and propensity scores were estimated using an ensemble learning estimation approach known as super learning^33^ (SL) and its implementation in the SL3 R package.^34^ For ITT and PP analyses, we estimated propensity scores for treatment initiation and each type of right-censoring events at each time point separately. For PP analyses, we also estimated propensity scores for treatment continuation (ie, no treatment interruption or crossover) at each time point separately. In the PP analysis, follow-up ended at the time of a right-censoring event or treatment protocol deviation in each arm (eg, crossover or discontinuation of the treatment initiated on the index date); in the ITT analysis, follow-up only ended at the time of a right-censoring event (eg, patients continued to contribute outcomes to their treatment arm even after treatment crossover or discontinuation of the initial therapy started on the index date). To improve the robustness of the findings, subgroup and effect modification analyses by baseline patient characteristics were implemented by replicating the PP and ITT analytic approach (including refitting all propensity scores) using only data from the patients in the subgroup considered.

Sensitivity analyses included IPW estimation^35^ of the same cumulative incidence curves, RD, and ARD at 2.5 years as those evaluated with targeted learning. Unadjusted estimates were derived using this IPW estimation approach but without weighting (ie, using equal weights set to 1 for all person-time observations). All TMLE and IPW estimates were implemented using untruncated and truncated^36^ weights (eg, the 99th percentile of the weight distribution for IPW, and absolute truncation values defaulting to 20 for IPW and 200 for TMLE). Finally, we implemented sensitivity analyses to unmeasured confounding including the calculation of g values.^22,37^ All estimation procedures were implemented using the stremr R package,^38^ which provides conservative estimates of SEs using the estimators’ influence curves.

Data analysis was conducted from May 1 to December 31, 2024, using R, version 4.3.0 (R Project for Statistical Computing). Two-sided *P* <.05 was considered statistically signficant.

## Results

This study included 296 676 adults. The cohort for emulating a 4-arm trial included a subset of 241 981 adults (mean [SD] age, 57.2 [12.9] years) with T2D; 45.7% were female and 54.3% were male. Patients identified as American Indian or Alaska Native (0.7%), Asian (15.7%), Black (10.1%), Hispanic (35.3%), Native Hawaiian or Other Pacific Islander (1.6%), White (49.8%), other race or ethnicity (0.1%), or multiple races or ethnicities (2.9%); race and ethnicity was unknown for 19.1%. Detailed data on baseline clinical and demographic characteristics of patients initiating medications compared in specific emulated trials are provided in Table 2 and eTables 2, 9, 14, 19, 24, 29, and 34 in Supplement 1. Results of primary (PP) analyses of new and sustained users, secondary (ITT) analyses of all new users, and sensitivity analyses are detailed in eAppendix 3 in Supplement 1, which includes eFigures 1 to 74 and eTables 1 to 66.

**Table 2.** Baseline Values for Selected Covariates in the Patient Cohort Used to Emulate a 4-Arm RCT for Comparing Sulfonylureas, DPP4is, SGLT2is, and GLP-1RAs^a^

Variable	Medication class				Total (n = 241 981)
	Sulfonylureas (n = 212 042)	DPP4is (n = 5862)	GLP-1RAs (n = 9219)	SGLT2is (n = 14 858)	
**Demographic**					
Age, mean (SD), y	56.9 (12.9)	61.7 (12.9)	56.0 (12.3)	60.5 (12.0)	57.2 (12.9)
Age group, y					
<45	36 000 (16.9)	546 (9.3)	1663 (18.0)	1503 (10.1)	39 712 (16.4)
45-64	116 736 (55.1)	2844 (48.5)	5203 (56.4)	7470 (50.3)	132 253 (54.7)
65-74	41 264 (19.5)	1528 (26.1)	1909 (20.7)	4306 (29.0)	49 007 (20.3)
≥75	18 042 (8.5)	944 (16.1)	444 (4.8)	1579 (10.6)	21 009 (8.7)
Sex					
Female	95 560 (45.1)	3123 (53.3)	5319 (57.7)	6537 (44.0)	110 539 (45.7)
Male	116 476 (54.9)	2739 (46.7)	3900 (42.3)	8321 (56.0)	131 436 (54.3)
Other^b^	4 (0)	0	0	0	4 (0)
Unknown	2 (0)	0	0	0	2 (0)
Race^c^					
American Indian or Alaska Native	1505 (0.7)	26 (0.4)	48 (0.5)	92 (0.6)	1671 (0.7)
Asian	33 756 (15.9)	735 (12.5)	731 (7.9)	2690 (18.1)	37 912 (15.7)
Black	20 977 (9.9)	669 (11.4)	1343 (14.6)	1483 (10.0)	24 472 (10.1)
Native Hawaiian or Other Pacific Islander	3303 (1.6)	61 (1.0)	143 (1.6)	371 (2.5)	3878 (1.6)
White	103 428 (48.8)	3689 (62.9)	5609 (60.8)	7666 (51.6)	120 392 (49.8)
Other race^d^	149 (0.1)	29 (0.5)	57 (0.6)	39 (0.3)	274 (0.1)
Multiple races	5740 (2.7)	186 (3.2)	523 (5.7)	662 (4.5)	7111 (2.9)
Unknown	43 184 (20.4)	467 (8.0)	765 (8.3)	1855 (12.5)	46 271 (19.1)
Hispanic ethnicity	78 965 (37.2)	1098 (18.7)	1943 (21.1)	3501 (23.6)	85 507 (35.3)
Current smoker	19 616 (9.3)	553 (9.4)	892 (9.7)	1229 (8.3)	22 290 (9.2)
BMI, mean (SD)	32.8 (7.3)	33.0 (7.6)	38.6 (8.3)	34.4 (7.7)	33.1 (7.5)
Missing	5904 (2.8)	272 (4.6)	264 (2.9)	630 (4.2)	7070 (2.9)
Insurance					
Medicaid	12 436 (5.9)	660 (11.3)	1387 (15.1)	1642 (11.1)	16 125 (6.7)
Medicare	58 294 (27.5)	2420 (41.3)	2610 (28.3)	5715 (38.5)	69 039 (28.5)
**Clinical**					
HbA_1c_, mean (SD), %	9.3 (2.1)	8.2 (1.7)	8.8 (1.9)	8.6 (1.7)	9.2 (2.1)
Missing	216 (0.1)	14 (0.2)	16 (0.2)	13 (0.1)	259 (0.1)
eGFR, mean (SD), mL/min/1.73 m^2^	89.0 (21.6)	80.8 (24.2)	86.0 (23.8)	79.8 (22.7)	88.1 (22.0)
Missing	5782 (2.7)	212 (3.6)	242 (2.6)	249 (1.7)	6485 (2.7)
UACR, mean (SD), mg/g	45.9 (82.7)	50.8 (92.0)	61.8 (102.1)	83.6 (118.0)	48.9 (86.8)
Missing	41 920 (19.8)	1341 (22.9)	2400 (26.0)	2708 (18.2)	48 369 (20.0)
LDL, mean (SD), mg/dL	96.6 (39.1)	89.8 (35.9)	91.2 (37.3)	85.0 (37.0)	95.5 (38.9)
Missing	23 654 (11.2)	533 (9.1)	953 (10.3)	1833 (12.3)	26 973 (11.2)
SBP, mean (SD), mm Hg	129.5 (14.5)	129.2 (15.2)	130.6 (14.6)	130.5 (15.7)	129.6 (14.6)
Missing	3769 (1.8)	252 (4.3)	182 (2.0)	327 (2.2)	4530 (1.9)
DBP, mean (SD), mm Hg	75.0 (10.7)	73.1 (11.0)	74.2 (11.2)	72.7 (11.7)	74.8 (10.8)
Missing	3766 (1.8)	252 (4.3)	182 (2.0)	327 (2.2)	4527 (1.9)
Comorbid condition^e^					
Atrial fibrillation	8265 (3.9)	467 (8.0)	492 (5.3)	1474 (9.9)	10 698 (4.4)
ASCVD	19 370 (9.1)	1013 (17.3)	1313 (14.2)	3021 (20.3)	24 717 (10.2)
CAD	2742 (1.3)	160 (2.7)	233 (2.5)	727 (4.9)	3862 (1.6)
Cerebrovascular disease	1749 (0.8)	72 (1.2)	71 (0.8)	183 (1.2)	2075 (0.9)
COPD	7354 (3.5)	411 (7.0)	575 (6.2)	983 (6.6)	9323 (3.9)
Depression	27 309 (12.9)	1133 (19.3)	2435 (26.4)	2316 (15.6)	33 193 (13.7)
Hypertension	114 520 (54.0)	4051 (69.1)	6421 (69.7)	10 673 (71.8)	135 665 (56.1)
PVD	375 (0.2)	31 (0.5)	41 (0.4)	64 (0.4)	511 (0.2)
HF	7705 (3.6)	429 (7.3)	716 (7.8)	2342 (15.8)	11 192 (4.6)
Metabolic bariatric surgery	2057 (0.97)	108 (1.8)	317 (3.4)	212 (1.4)	2694 (1.1)
Concurrent medications					
Metformin	144 034 (67.9)	3913 (66.8)	6237 (67.7)	10 470 (70.5)	164 654 (68.0)
Metformin monotherapy	132 066 (62.3)	2849 (48.6)	2485 (27.0)	5992 (40.3)	143 392 (59.3)
Insulin	15 199 (7.2)	1518 (25.9)	5611 (60.9)	6503 (43.8)	28 831 (11.9)
Statin	118 477 (55.9)	3756 (64.1)	6335 (68.7)	11 381 (76.6)	139 949 (57.8)

Abbreviations: ASCVD, atherosclerotic cardiovascular disease; BMI, body mass index (calculated as weight in kilograms divided by height in meters squared); CAD, coronary artery disease; COPD, chronic obstructive pulmonary disease; DBP, diastolic blood pressure; DPP4i, dipeptidyl peptidase-4 inhibitor; eGFR, estimated glomerular filtration rate; GLP-1RA, glucagon-like peptide-1 receptor agonist; HbA_1c_, hemoglobin A_1c_; HF, heart failure; LDL, low-density lipoprotein; PVD, peripheral vascular disease; SBP, systolic blood pressure; SGLT2i, sodium-glucose cotransporter-2 inhibitor; UACR, urinary albumin-creatinine ratio.

SI conversion factors: To convert HbA_1c_ to proportion of total hemoglobin, multiply by 0.01; to convert LDL to mmol/L, multiply by 0.0259.

^a^ For each continuous variable, means (SDs) are displayed for all patients in the cohort (last column) and by medication class initiated at cohort entry. For each categorical variable and for each possible level of that variable, the count and proportion are displayed instead. Values may not sum to 100% due to rounding.

^b^ Patients self-reported their sex and had the option to choose “other,” which was not further defined.

^c^ Sum totals may exceed the number of adults in the column headings because adults could report Hispanic ethnicity in addition to a given race.

^d^ Patients self-reported their race and had the option to choose “other,” which was not further defined.

^e^ Defined using diagnosis codes.

As illustrated in Figure 1, of 241 981 eligible index fills (n = 229 806 patients) at cohort entry, most were triggered by initiation of sulfonylureas (n = 212 042 fills [87.6%]) compared with DPP4is (n = 5862 fills [2.4%]), SGLT2is (n = 14 858 fills [6.1%]), or GLP-1RAs (n = 9219 fills [3.8%]). At least two-thirds of patients in all 4 exposure groups used metformin at baseline, whereas baseline insulin use was highest among GLP-1RA initiators (60.9%) compared with sulfonylurea initiators (7.2%), DPP4i initiators (25.9%), and SGLT2i initiators (43.8%). In the 4-arm cohort, 48.4%, 44.1%, 41.4%, and 21.9% of sulfonylurea, DPP4i, GLP-1RA, and SGLT2i initiators discontinued the treatment they initiated at cohort entry, whereas 7.1%, 28.3%, 11.4%, and 12.1% initiated a comparator medication, respectively (eTables 3-8 in Supplement 1 for the 4-arm cohort and eTables 10, 15, 20,25, 30, and 35 in Supplement 1 for the 2-arm cohorts). In the 4-arm cohort, median time to protocol deviation in PP analyses was 7 months (IQR, 3-13 months) for GLP-1RA initiators, 8 months (IQR, 4-16 months) for DPP4i initiators, 8 months (IQR, 4-11 months) for SGLT2i initiators, and 10 months (IQR, 8-22 months) for sulfonylurea initiators. Most GLP-1RA users were taking exenatide (approximately 20.0%), liraglutide (approximately 50.0%), or semaglutide (approximately 20.0%).

**Figure 2.**
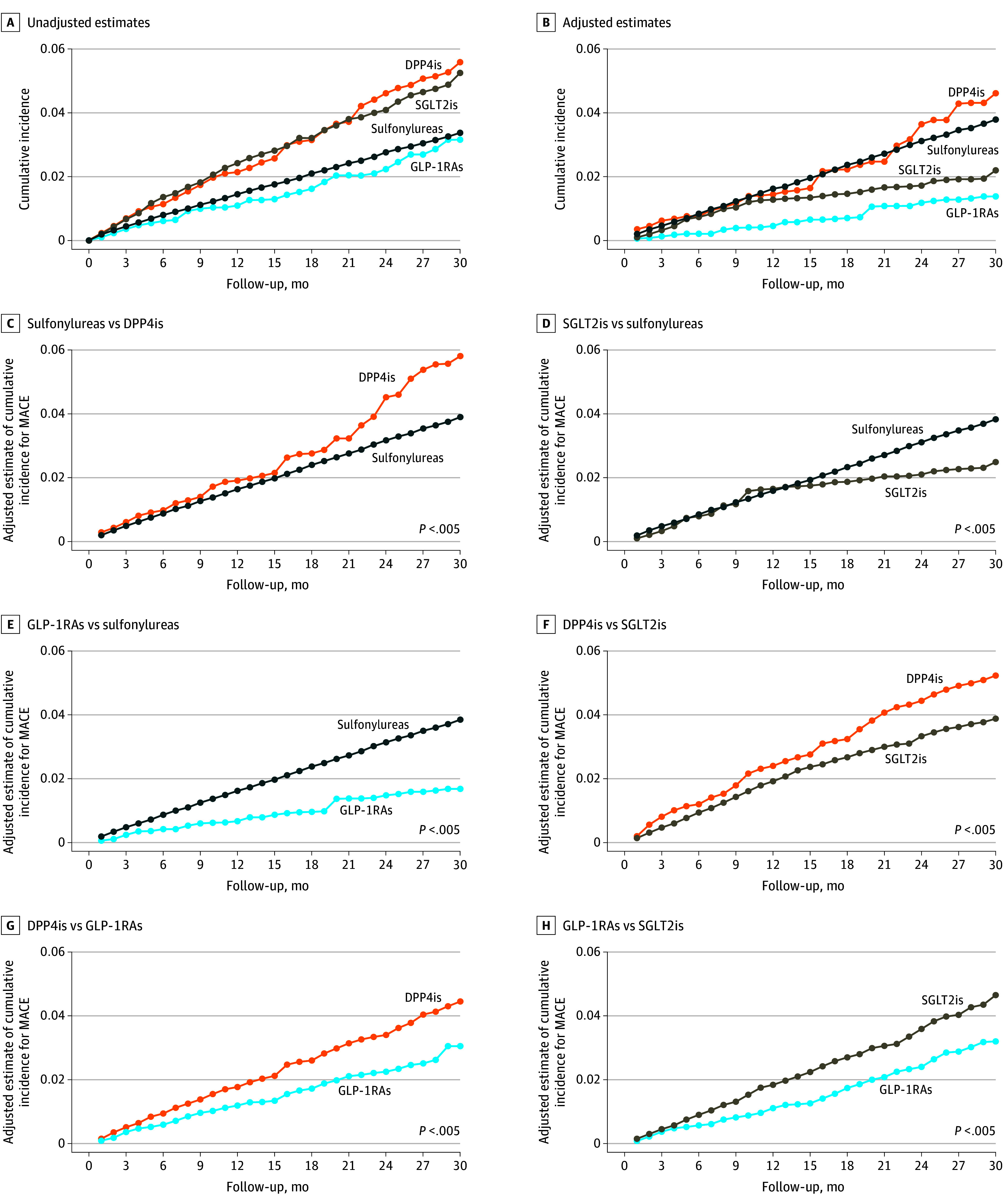
Cumulative Incidence Curves for Major Adverse Cardiovascular Events (MACEs) for Glucose-Lowering Medications A to H, Each plot represents unadjusted (A) or adjusted (B-H) estimates of cumulative incidence curves for MACEs derived with targeted learning (ie, targeted minimum loss–based estimation with super learning estimates of propensity scores) and no weight truncation. A and B, The plots emulate inferences (PP analyses) from 4-arm randomized clinical trials (RCTs) for comparing sulfonylureas, dipeptidyl peptidase-4 inhibitors (DPP4is), sodium-glucose cotransporter-2 inhibitors (SGLT2i), and glucagon-like peptide-1 receptor agonists (GLP-1RA) before (A) and after (B) covariate adjustment. C to H, The plots emulate inferences (PP analyses) from one of six 2-arm RCTs after covariate adjustment to compare sulfonylureas to DPP4is (C), SGLT2is to sulfonylureas (D), GLP-1RAs to sulfonylureas (E), SGLT2is to DPP4is (F), GLP-1RAs to DPP4is (G), and GLP-1RAs to SGLT2is (H). Plots emulating inferences from 2-arm RCTs display a *P* value for the test that the average risk difference through 2.5 years of follow-up (30 months) is 0.

Adjusted MACE risk estimates among sustained users (PP analyses) varied substantially across classes of glucose-lowering medications, with the lowest risk observed with sustained exposure to GLP-1RAs, followed by SGLT2is, sulfonylureas, and DPP4is (Figure 2). The ordering of the 4 adjusted cumulative incidence curves in Figure 2B and the corresponding comparisons of the adjusted 2.5-year ARD estimates between any 2 arms of the 4-arm cohort indicated a consistent decrease in MACE risk with sustained exposure to GLP-1RAs compared with SGLT2is (ARD, −0.006 [95% CI, −0.008 to −0.004]; *P* < .001), sulfonylureas (ARD, −0.013 [95% CI, −0.015 to −0.012]; *P* < .001), and DPP4is (ARD, −0.015 [95% CI, −0.018 to −0.011]; *P* < .001) over time. In addition, there was a consistent decrease in risk with sustained exposure to SGLT2is compared with sulfonylureas (ARD, −0.007 [95% CI, −0.009 to −0.005]; *P* < .001) and DPP4is (ARD, −0.009 [95% CI, −0.013 to −0.005]; *P* < .001), and a decrease in risk with sustained exposure to sulfonylureas compared with DPP4is (ARD, −0.001 [95% CI, −0.005 to 0.002]; *P* = .47). Similar decreases in adjusted MACE risk estimates were found in the 2-arm cohorts (Figure 2) when comparing areas under the cumulative incidence curves (ARDs) and adjusted 2.5-year RDs (Table 3). Adjusted estimates from the 2-arm cohort for comparing sulfonylureas with DPP4is indicated a consistent statistically significant increase in MACE risk with DPP4is compared with sulfonylureas over time (Figure 2C). Comparing DPP4is with sulfonylureas and SGLT2is with GLP-1RAs, the 2.5-year cumulative risk difference was 1.9% (95% CI, 1.1%-2.7%) and 1.5% (95% CI, 1.1%-1.9%), respectively.

**Table 3.** Cumulative Incidences and Their Difference at 2.5-Year Follow-Up From PP and ITT Analyses in Emulated 2-Arm RCTs Using TMLE With Super Learning (Untruncated Weights)

Treatment vs control subgroup comparison	No. of patients		PP analysis				ITT analysis		Effect modification *P* value
	Treatment group	Control group	Risk, %		RD (95% CI)^a^	NNT	RD (95% CI)	NNT	
			In treatment group	In control group					
**Sulfonylureas vs GLP-1RAs**									
All	221 910	12 680	3.9	1.7	2.2 (1.9-2.4)	46	0.7 (0.1-1.3)	141	NA
With ASCVD	21 291	1993	14.0	3.1	10.9 (9.9-11.9)	9	5.4 (3.8-7.1)	18	<.005
Without ASCVD	200 619	10 687	2.7	1.6	1.2 (0.9-1.5)	85	0.5 (−0.1 to 1.0)	NA	
Without ASCVD and with metformin monotherapy	123 942	2443	2.5	1.2	1.3 (0.8-1.7)	80	0.5 (0.3-0.6)	219	NA
**Sulfonylureas vs SGLT2is**									
All	219 957	18 956	3.8	2.5	1.3 (1.0-1.7)	75	1.1 (0.7-1.4)	95	NA
With ASCVD	20 578	3805	13.4	6.7	6.7 (5.6-7.8)	15	2.9 (−0.5 to 6.2)	NA	<.005
Without ASCVD	199 379	15 151	2.7	2.0	0.7 (0.3-1.2)	135	0.5 (0.1-0.8)	220	
Without ASCVD and with metformin monotherapy	124 039	5362	2.3	1.8	0.6 (−0.1 to 1.2)	NA	0.4 (−0.04 to 0.9)	NA	NA
**DPP4is vs GLP-1RAs**									
All	30 165	22 699	4.5	3.1	1.4 (0.7-2.1)	71	0.3 (−0.3 to 0.9)	NA	NA
With ASCVD	4049	2984	13.1	9.0	4.1 (1.7-6.4)	25	0.7 (−1.6 to 3.1)	NA	.02
Without ASCVD	26 116	19 715	3.0	1.8	1.2 (0.7-1.7)	86	0.3 (−0.2 to 0.8)	NA	
Without ASCVD and with metformin monotherapy	3452	2677	2.6	1.7	0.9 (−0.1 to 1.9)	NA	0.3 (−0.6 to 1.1)	NA	NA
**DPP4is vs SGLT2is**									
All	28 194	47 089	5.2	3.9	1.3 (0.8-1.9)	74	1.2 (0.7-1.6)	84	NA
With ASCVD	3623	7674	13.9	12.3	1.6 (−0.5 to 3.8)	NA	1.1 (−0.6 to 2.9)	NA	.44
Without ASCVD	24 571	39 415	3.5	2.7	0.8 (0.2-1.3)	133	0.6 (0.1-1.1)	161	
Without ASCVD and with metformin monotherapy	3361	6086	3.2	2.6	0.6 (−0.5 to 1.7)	NA	0.4 (−0.4 to 1.2)	NA	NA
**DPP4is vs sulfonylureas**									
All	7062	220 085	5.8	3.9	1.9 (1.1-2.7)	52	0.5 (−0.04 to 1.0)	NA	NA
With ASCVD	1289	21 000	16.7	14.3	2.4 (−1.4 to 6.3)	NA	−2.1 (−4.5 to 0.4)	NA	.67
Without ASCVD	5773	199 085	4.3	2.7	1.6 (0.8-2.3)	64	0.4 (−0.1 to 0.9)	NA	
Without ASCVD and with metformin monotherapy	2593	123 719	3.1	2.4	0.7 (−0.3 to 1.7)	NA	0.4 (−0.3 to 1.1)	NA	NA
**SGLT2is vs GLP-1RAs**									
All	49 103	21 741	4.7	3.2	1.5 (1.1-1.9)	69	0.7 (0.4-1.1)	139	NA
With ASCVD	7925	2743	12.9	7.6	5.3 (3.9-6.7)	19	3.9 (2.7-5.1)	26	<.005
Without ASCVD	41 178	18 998	3.4	2.5	0.9 (0.4-1.3)	118	0.03 (−0.3 to 0.4)	NA	
Without ASCVD and with metformin monotherapy	6136	2630	2.9	1.4	1.4 (0.6-2.3)	70	0.1 (−0.6 to 0.9)	NA	NA
With HF	5117	1391	16.3	10.2	6.1 (4.2-8.1)	16	5.7 (4.1-7.4)	17	<.005
Without HF	43 536	20 186	3.6	2.8	0.8 (0.4-1.3)	123	0.1 (−0.3 to 0.4)	NA	
Without HF and without ASCVD	38 546	18 178	3.1	2.2	0.9 (0.4-1.4)	113	0.1 (−0.3 to 0.5)	NA	.20
Without HF and with ASCVD	4990	2008	9.4	7.5	1.9 (0.5-3.3)	53	1.1 (−0.2 to 2.3)	NA	
With HF and without ASCVD	2357	719	12.1	9.4	2.7 (0.3-5.1)	37	2.0 (0.1-3.9)	50	<.005
With HF and ASCVD	2760	672	20.0	9.1	10.9 (8.1-13.6)	9	10.4 (7.8-12.9)	10	
CKD risk									
Low	22 777	10 148	3.0	1.9	1.1 (0.6-1.5)	95	0.7 (0.3-1.1)	145	<.005
Moderate	12 019	4755	6.3	3.2	3.0 (2.0-4.1)	33	1.7 (0.9-2.5)	59	
High	8189	2334	7.3	6.5	0.8 (−0.5 to 2.1)	NA	−0.9 (−2.0 to 0.2)	NA	NA
Sex									
Female	20 968	11 769	3.5	2.5	1.0 (0.5-1.6)	96	0.6 (0.2-1.0)	169	.09
Male	28 134	9972	5.6	3.8	1.8 (1.1-2.5)	55	0.9 (0.4-1.5)	109	
Age group, y									
<50	8264	5828	1.2	1.2	0 (−0.5 to 0.5)	NA	0.01 (−0.4 to 0.5)	NA	NA
50-<65	21 997	10 272	3.6	3.0	0.6 (0.1-1.1)	159	0.3 (−0.1 to 0.7)	NA	<.005
≥65	18 842	5641	8.3	4.7	3.6 (2.8-4.4)	28	1.6 (0.8-2.4)	63	
Race									
Asian	9471	1777	3.5	1.7	1.8 (1.2-2.5)	55	0.9 (0.1-1.7)	107	NA
Black	4285	2637	5.1	4.1	1.0 (−0.2 to 2.1)	NA	1.4 (0.5-2.3)	71	
Native Hawaiian or Other Pacific Islander	992	337	4.9	1.8	3.2 (1.6-4.7)	32	1.4 (−0.2 to 3.1)	NA	
White	18 155	10 505	5.6	4.3	1.2 (0.5-2.0)	81	0.3 (−0.3 to 0.9)	NA	
Multiple races	1419	903	2.4	1.9	0.4 (−0.7 to 1.6)	NA	0.5 (−0. 7 to 1.7)	NA	
Hispanic ethnicity	13 624	5145	3.3	1.4	2.0 (1.5-2.4)	51	1.0 (0.3-1.6)	106	
**Exenatide vs liraglutide**									
All	5959	13 854	3.1	3.7	−0.6 (−1.9 to 0.7)	NA	−1.1 (−1.8 to −0.4)	90	NA
**Semaglutide vs liraglutide**									
All	6047	16 063	3.5	3.8	−0.2 (−1.0 to 0.5)	NA	0.8 (−0 to 1.6)	126	NA

Abbreviations: ASCVD, atherosclerotic cardiovascular disease; CKD, chronic kidney disease; DPP4i, dipeptidyl peptidase-4 inhibitor; GLP-1RA, glucagon-like peptide-1 receptor agonist; HF, heart failure; ITT, intention to treat; NA, not applicable; PP, per protocol; RD, risk difference; SGLT2i, sodium-glucose cotransporter-2 inhibitor.

^a^ RDs (risk in treatment arm minus risk in control arm) are provided for all medication initiators or subgroups, along with *P* value for the effect modification test that RDs estimated in PP analyses are equal between 2 subgroups.

For all six 2-way medication class comparisons of sustained users (Table 3), adjusted 2.5-year RD estimates among patients with and without ASCVD were similar in direction to those obtained for the overall cohort. RDs were generally much smaller (and corresponding NNTs much higher) in sustained users without ASCVD vs with ASCVD. Among sustained users with no ASCVD previously receiving metformin monotherapy, RDs were generally smaller in magnitude and statistically insignificant except when comparing SGLT2is and sulfonylureas with GLP-1RAs.

The decreased MACE risk estimates in sustained users of GLP-1RAs compared with SGLT2is were most pronounced in patients with ASCVD, in those with HF (with or without ASCVD), in those aged 65 years or older, and in those with moderate baseline kidney impairment. There was no statistically significant decreased risk of MACEs in sustained users of GLP-1RAs compared with SGLT2is in those younger than 50 years (Table 3 and eFigure 51 in Supplement 1). PP analyses showed a decreased risk in sustained users of sulfonylureas compared with DPP4is overall and in those without ASCVD. Although MACE risk was smaller with sustained use of sulfonylureas compared with DPP4is, the RD at 2.5 years was not statistically significant for those with ASCVD or who previously received metformin monotherapy without ASCVD.

The adjusted 2.5-year RD estimates (Table 3) and ARDs (eFigures 60-61 and eTables 59-60 in Supplement 1) from the primary PP analyses of sustained users of exenatide vs liraglutide and semaglutide vs liraglutide did not indicate significant differences in MACE risks between the GLP-1RAs compared. The adjusted 2.5-year RD estimates from secondary ITT analyses of all new users were generally similar in direction to those from primary PP analyses of sustained users but typically much smaller in magnitude or not statistically significant (Table 3).

Results from sensitivity analyses that examined the secondary MACE outcome (broader definition of cardiovascular death) support conclusions from the primary outcome (eFigures 62-67 and eTables 61-66 in Supplement 1). When comparing GLP-1RAs with SGLT2is, sulfonylureas with DPP4is, and GLP-1RAs with DPP4is in the 2-arm cohorts, the sensitivity analyses (eAppendix 4 and eFigures 69-74 in Supplement 1) suggested that adjustment for a plausible level of unmeasured confounding and selection bias would actually result in strengthening the evidence obtained based on only observed covariates. When comparing sulfonylureas with SGLT2is, sulfonylureas with GLP-1RAs, and DPP4is with SGLT2is in the 2-arm cohorts, sensitivity analyses suggested that adjustment for a plausible level of unmeasured confounding and selection bias would slightly weaken the evidence based on only observed covariates.

## Discussion

This study evaluated the head-to-head comparative effectiveness of initiation followed by sustained use of 4 classes of glucose-lowering medications (primary PP analyses) using principled causal inference methods combined with machine learning adjustment for baseline and time-varying covariates. MACE risk varied significantly across medication classes, with the best results observed with GLP-1RAs followed by SGLT2is, sulfonylureas, and DPP4is. Differences in MACE risk were much smaller (and NNTs were much higher) for patients without ASCVD than for those with ASCVD. Agent-specific comparisons of sustained users of exenatide, liraglutide, and semaglutide did not reveal significant differences in MACE risk.

This study provides data on the cardiovascular effects of GLP-1RAs compared with SGLT2is. The benefit of GLP-1RAs over SGLT2is was most pronounced in patients with ASCVD, HF (with or without ASCVD), moderate kidney impairment, or age 65 years or older. GLP-1RAs performed well compared with SGLT2is in patients with HF and in some patients with kidney impairment. In patients younger than 50 years, SGLT2is and GLP-1RAs had a similar effect on MACE. However, most GLP-1RA users were taking exenatide (approximately 20.0%), liraglutide (approximately 50.0%), or semaglutide (approximately 20.0%); therefore, our results may underestimate benefits obtainable with newer incretin mimetics. Of note, among patients with no ASCVD who were receiving metformin monotherapy at baseline, there were no significant differences in MACEs in PP analysis for the following 2-way comparisons: GLP-1RAs vs DPP4is, SGLT2is vs DPP4is, SGLT2is vs sulfonylureas, and DPP4is vs sulfonylureas. However, due to limited sample sizes, the findings cannot rule out clinically significant differences in risk, consistent with the overall population findings.

Prior RCTs have established the cardiovascular effects of DPP4is, SGLT2is, and GLP-1RAs against placebo,^13,14,15^ but rarely have head-to-head RCTs compared cardiovascular outcomes of these medications with one another. The current study sheds light on the cardiovascular effects of SGLT2is compared with GLP-1RAs and of sulfonylureas compared with DPP4is, the benefits of early initiation of GLP-1RAs or SGLT2is based on clinical indications, and the heterogeneity of results in various subgroups of patients, particularly among those with vs without ASCVD.

We note that the results of comparing sustained users of sulfonylureas with DPP4is are qualitatively consistent with MACE findings of the GRADE randomized trial (eFigure 68 in Supplement 1), which directly compared the effect of these medications on cardiovascular outcomes in secondary analyses.^39^ In well-resourced health care systems, incretin mimetics and SGLT2is are often preferred medications; but for patients who cannot afford newer agents and for hundreds of millions of individuals with T2D in low- and middle-income countries, comparisons that involve sulfonylureas and DPP4is with or without metformin remain highly relevant.

Our finding of a beneficial effect of GLP-1RAs over SGLT2is was also consistent with a prior European observational study.^17^ However, our overall results were not always consistent with findings from other large comparative observational studies that addressed the same comparative effectiveness questions.^13,14,15,16,17,18^ The inconsistency might be explained in part by differences in study participants and by several methodological limitations or analytic choices in prior published work that lacked clinical data on important confounders such as blood pressure, lipid levels, smoking, kidney function, and body mass index; focused sharply on ITT effect despite high medication discontinuation and crossover^29^; did not address time-varying confounders or attrition bias; had restrictive entry criteria; did not account for interaction effects or made other strong modeling assumptions in propensity score development; or evaluated HRs instead of RDs as the primary effect measure despite violations of the proportionality assumption (see details in eAppendix 2 in Supplement 1).

### Strengths and Limitations

This study has several strengths. It had broad inclusion criteria and robust representation of underserved communities, inclusion of clinical measures from EHRs, application of principled causal inference methods combined with machine learning within a trial emulation framework, and conduct of sensitivity analyses that confirmed findings from primary analyses.

A number of limitations constrain the interpretation of our results. This study was conducted at 6 US sites, and replication in other patient populations is warranted, especially in uninsured patients. We did not include metformin, thiazolidinediones, or insulin as direct comparators in our analyses, and follow-up beyond 2.5 years might further clarify comparative effectiveness questions. We estimated medication exposure based on pharmacy fill data, an imperfect estimator of biological medication exposure. Despite our principled analytic approach, there is some possibility of measurement error, unmeasured sources of confounding, or selection bias, although sensitivity analyses suggest that our findings are robust to residual bias from unmeasured covariates.

## Conclusions

In this comparative effectiveness study of adults with T2D, MACE risk varied significantly by medication class, with the most risk reduction achieved with sustained treatment with GLP-1RAs, followed by SGLT2is, sulfonylureas, and DPP4is, in that order. The magnitude of benefit of GLP-1RAs over SGLT2is depended on patient baseline age, ASCVD, HF, and kidney impairment. These results, along with consideration of collateral clinical effects, cost, and availability, may inform treatment decisions for adults with T2D.^40,41^

## Notes

Supplement 1. **eAppendix 1.** Detailed Description of the Analytic Approach **eTable 1.** Summary of the Specifications and Emulations of the Various Target Trials Considered in This Study **eAppendix 2.** Limitations of Prior Observational Studies **eAppendix 3.** Detailed Results From Primary (Per Protocol), Secondary (Intention to Treat), and Sensitivity Analyses **eFigure 1.** MACE (Primary Definition), 4-Arm Drug Class Comparison, Sulfonylureas vs DPP4is vs SGLT2is vs GLP1-RAs, CONSORT Diagram **eTable 2.** MACE (Primary Definition), 4-Arm Drug Class Comparison, Sulfonylureas vs DPP4is vs SGLT2is vs GLP1-RAs, Patient Characteristics at Baseline (Overall and by Medication Initiated) **eFigure 2.** MACE (Primary Definition), 4-Arm Drug Class Comparison, Sulfonylureas vs DPP4is vs SGLT2is vs GLP1-RAs, Cumulative Incidence Curves From PP Analyses With IPW, TMLE, and SL **eFigure 3.** MACE (Primary Definition), 4-Arm Drug Class Comparison, Sulfonylureas vs DPP4is vs SGLT2is vs GLP1-RAs, Cumulative Incidence Curves From ITT Analyses With IPW, TMLE, and SL **eTable 3.** MACE (Primary Definition), 4-Arm Drug Class Comparison, Sulfonylureas vs DPP4is vs SGLT2is vs GLP1-RAs, RD and HR Effect Measures for Comparing Sulfonylureas to DPP4is at 2.5 Years **eTable 4.** MACE (Primary Definition), 4-Arm Drug Class Comparison, Sulfonylureas vs DPP4is vs SGLT2is vs GLP1-RAs, RD and HR Effect Measures for Comparing Sulfonylureas to SGLT2is at 2.5 Years **eTable 5.** MACE (Primary Definition), 4-Arm Drug Class Comparison, Sulfonylureas vs DPP4is vs SGLT2is vs GLP1-RAs, RD and HR Effect Measures for Comparing Sulfonylureas to GLP-1RAs at 2.5 Years **eTable 6.** MACE (Primary Definition), 4-Arm Drug Class Comparison, Sulfonylureas vs DPP4is vs SGLT2is vs GLP1-RAs, RD and HR Effect Measures for Comparing DPP4is to SGLT2is at 2.5 Years **eTable 7.** MACE (Primary Definition), 4-Arm Drug Class Comparison, Sulfonylureas vs DPP4is vs SGLT2is vs GLP1-RAs, RD and HR Effect Measures for Comparing DPP4is to GLP-1RAs at 2.5 Years **eTable 8.** MACE (Primary Definition), 4-Arm Drug Class Comparison, Sulfonylureas vs DPP4is vs SGLT2is vs GLP1-RAs, RD and HR Effect Measures for Comparing SGLT2is to DPP4is at 2.5 Years **eFigure 4.** MACE (Primary Definition), 2-Arm Drug Class Comparison, Sulfonylureas vs DPP4is, CONSORT Diagram **eTable 9.** MACE (Primary Definition), 2-Arm Drug Class Comparison, Sulfonylureas vs DPP4is, Patient Characteristics at Baseline (Overall and by Medication Initiated) **eFigure 5.** MACE (Primary Definition), 2-Arm Drug Class Comparison, Sulfonylureas vs DPP4is, Cumulative Incidence Curves From PP and ITT Analyses With IPW, TMLE, and SL **eFigure 6.** MACE (Primary Definition), 2-Arm Drug Class Comparison, Sulfonylureas vs DPP4is, Cumulative Incidence Curves From Sensitivity PP Analyses With IPW, TMLE, and SL **eTable 10.** MACE (Primary Definition), 2-Arm Drug Class Comparison, Sulfonylureas vs DPP4is, RD and HR Effect Measures at 2.5 Years **eFigure 7.** MACE (Primary Definition), 2-Arm Drug Class Comparison, Sulfonylureas vs DPP4is, ASCVD Subgroup, Cumulative Incidence Curves From PP and ITT Analyses With IPW, TMLE, and SL **eTable 11.** MACE (Primary Definition), 2-Arm Drug Class Comparison, Sulfonylureas vs DPP4is, ASCVD Subgroup, RD and HR Effect Measures at 2.5 Years **eFigure 8.** MACE (Primary Definition), 2-Arm Drug Class Comparison, Sulfonylureas vs DPP4is, No ASCVD Subgroup, Cumulative Incidence Curves From PP and ITT Analyses With IPW, TMLE, and SL **eTable 12.** MACE (Primary Definition), 2-Arm Drug Class Comparison, Sulfonylureas vs DPP4is, No ASCVD Subgroup, RD and HR Effect Measures at 2.5 Years **eFigure 9.** MACE (Primary Definition), 2-Arm Drug Class Comparison, Sulfonylureas vs DPP4is, No ASCVD and MET Subgroup, Cumulative Incidence Curves From PP and ITT Analyses With IPW, TMLE, and SL **eTable 13.** MACE (Primary Definition), 2-Arm Drug Class Comparison, Sulfonylureas vs DPP4is, No ASCVD and MET Subgroup, RD and HR Effect Measures at 2.5 Years **eFigure 10.** MACE (Primary Definition), 2-Arm Drug Class Comparison, Sulfonylureas vs SGLT2is, CONSORT Diagram **eTable 14.** MACE (Primary Definition), 2-Arm Drug Class Comparison, Sulfonylureas vs SGLT2is, Patient Characteristics at Baseline (Overall and by Medication Initiated) **eFigure 11.** MACE (Primary Definition), 2-Arm Drug Class Comparison, Sulfonylureas vs SGLT2is, Cumulative Incidence Curves From PP and ITT Analyses With IPW, TMLE, and SL **eFigure 12.** MACE (Primary Definition), 2-Arm Drug Class Comparison, Sulfonylureas vs SGLT2is, Cumulative Incidence Curves From Sensitivity PP Analyses With IPW, TMLE, and SL **eTable 15.** MACE (Primary Definition), 2-Arm Drug Class Comparison, Sulfonylureas vs SGLT2is, RD and HR Effect Measures at 2.5 Years **eFigure 13.** MACE (Primary Definition), 2-Arm Drug Class Comparison, Sulfonylureas vs SGLT2is, ASCVD Subgroup, Cumulative Incidence Curves From PP and ITT Analyses With IPW, TMLE, and SL **eTable 16.** MACE (Primary Definition), 2-Arm Drug Class Comparison, Sulfonylureas vs SGLT2is, ASCVD Subgroup, RD and HR Effect Measures at 2.5 Years **eFigure 14.** MACE (Primary Definition), 2-Arm Drug Class Comparison, Sulfonylureas vs SGLT2is, No ASCVD Subgroup, Cumulative Incidence Curves From PP and ITT Analyses With IPW, TMLE, and SL **eTable 17.** MACE (Primary Definition), 2-Arm Drug Class Comparison, Sulfonylureas vs SGLT2is, No ASCVD Subgroup, RD and HR Effect Measures at 2.5 Years **eFigure 15.** MACE (Primary Definition), 2-Arm Drug Class Comparison, Sulfonylureas vs SGLT2is, No ASCVD and MET Subgroup, Cumulative Incidence Curves From PP and ITT Analyses With IPW, TMLE, and SL **eTable 18.** MACE (Primary Definition), 2-Arm Drug Class Comparison, Sulfonylureas vs SGLT2is, No ASCVD and MET Subgroup, RD and HR Effect Measures at 2.5 Years **eFigure 16.** MACE (Primary Definition), 2-Arm Drug Class Comparison, Sulfonylureas vs GLP-1RAs, CONSORT Diagram **eTable 19.** MACE (Primary Definition), 2-Arm Drug Class Comparison, Sulfonylureas vs GLP-1RAs, Patient Characteristics at Baseline (Overall and by Medication Initiated) **eFigure 17.** MACE (Primary Definition), 2-Arm Drug Class Comparison, Sulfonylureas vs GLP-1RAs, Cumulative Incidence Curves From PP and ITT Analyses With IPW, TMLE, and SL **eFigure 18.** MACE (Primary Definition), 2-Arm Drug Class Comparison, Sulfonylureas vs GLP-1RAs, Cumulative Incidence Curves From Sensitivity PP Analyses With IPW, TMLE, and SL **eTable 20.** MACE (Primary Definition), 2-Arm Drug Class Comparison, Sulfonylureas vs GLP-1RAs, RD and HR Effect Measures at 2.5 Years **eFigure 19.** MACE (Primary Definition), 2-Arm Drug Class Comparison, Sulfonylureas vs GLP-1RAs, ASCVD Subgroup, Cumulative Incidence Curves From PP and ITT Analyses With IPW, TMLE, and SL **eTable 21.** MACE (Primary Definition), 2-Arm Drug Class Comparison, Sulfonylureas vs GLP-1RAs, ASCVD Subgroup, RD and HR Effect Measures at 2.5 Years **eFigure 20.** MACE (Primary Definition), 2-Arm Drug Class Comparison, Sulfonylureas vs GLP-1RAs, No ASCVD Subgroup, Cumulative Incidence Curves From PP and ITT Analyses With IPW, TMLE, and SL **eTable 22.** MACE (Primary Definition), 2-Arm Drug Class Comparison, Sulfonylureas vs GLP-1RAs, No ASCVD Subgroup, RD and HR Effect Measures at 2.5 Years **eFigure 21.** MACE (Primary Definition), 2-Arm Drug Class Comparison, Sulfonylureas vs GLP-1RAs, No ASCVD and MET Subgroup, Cumulative Incidence Curves From PP and ITT Analyses With IPW, TMLE, and SL **eTable 23.** MACE (Primary Definition), 2-Arm Drug Class Comparison, Sulfonylureas vs GLP-1RAs, No ASCVD and MET Subgroup, RD and HR Effect Measures at 2.5 Years **eFigure 22.** MACE (Primary Definition), 2-Arm Drug Class Comparison, DPP4is vs SGLT2is, CONSORT Diagram **eTable 24.** MACE (Primary Definition), 2-Arm Drug Class Comparison, DPP4is vs SGLT2is, Patient Characteristics at Baseline (Overall and by Medication Initiated) **eFigure 23.** MACE (Primary Definition), 2-Arm Drug Class Comparison, DPP4is vs SGLT2is, Cumulative Incidence Curves From PP and ITT Analyses With IPW, TMLE, and SL **eFigure 24.** MACE (Primary Definition), 2-Arm Drug Class Comparison, DPP4is vs SGLT2is, Cumulative Incidence Curves From Sensitivity PP Analyses With IPW, TMLE, and SL **eTable 25.** MACE (Primary Definition), 2-Arm Drug Class Comparison, DPP4is vs SGLT2is, RD and HR Effect Measures at 2.5 Years **eFigure 25.** MACE (Primary Definition), 2-Arm Drug Class Comparison, DPP4is vs SGLT2is, ASCVD Subgroup, Cumulative Incidence Curves From PP and ITT Analyses With IPW, TMLE, and SL **eTable 26.** MACE (Primary Definition), 2-Arm Drug Class Comparison, DPP4is vs SGLT2is, ASCVD Subgroup, RD and HR Effect Measures at 2.5 Years **eFigure 26.** MACE (Primary Definition), 2-Arm Drug Class Comparison, DPP4is vs SGLT2is, No ASCVD Subgroup, Cumulative Incidence Curves From PP and ITT Analyses With IPW, TMLE, and SL **eTable 27.** MACE (Primary Definition), 2-Arm Drug Class Comparison, DPP4is vs SGLT2is, No ASCVD Subgroup, RD and HR Effect Measures at 2.5 Years **eFigure 27.** MACE (Primary Definition), 2-Arm Drug Class Comparison, DPP4is vs SGLT2is, No ASCVD and MET Subgroup, Cumulative Incidence Curves From PP and ITT Analyses With IPW, TMLE, and SL **eTable 28.** MACE (Primary Definition), 2-Arm Drug Class Comparison, DPP4is vs SGLT2is, No ASCVD and MET Subgroup, RD and HR Effect Measures at 2.5 Years **eFigure 28.** MACE (Primary Definition), 2-Arm Drug Class Comparison, DPP4is vs GLP-1RAs, CONSORT Diagram **eTable 29.** MACE (Primary Definition), 2-Arm Drug Class Comparison, DPP4is vs GLP-1RAs, Patient Characteristics at Baseline (Overall and by Medication Initiated) **eFigure 29.** MACE (Primary Definition), 2-Arm Drug Class Comparison, DPP4is vs GLP-1RAs, Cumulative Incidence Curves From PP and ITT Analyses With IPW, TMLE, and SL **eFigure 30.** MACE (Primary Definition), 2-Arm Drug Class Comparison, DPP4is vs GLP-1RAs, Cumulative Incidence Curves From Sensitivity PP Analyses With IPW, TMLE, and SL **eTable 30.** MACE (Primary Definition), 2-Arm Drug Class Comparison, DPP4is vs GLP-1RAs, RD and HR Effect Measures at 2.5 Years **eFigure 31.** MACE (Primary Definition), 2-Arm Drug Class Comparison, DPP4is vs GLP-1RAs, ASCVD Subgroup, Cumulative Incidence Curves From PP and ITT Analyses With IPW, TMLE, and SL **eTable 31.** MACE (Primary Definition), 2-Arm Drug Class Comparison, DPP4is vs GLP-1RAs, ASCVD Subgroup, RD and HR Effect Measures at 2.5 Years **eFigure 32.** MACE (Primary Definition), 2-Arm Drug Class Comparison, DPP4is vs GLP-1RAs, No ASCVD Subgroup, Cumulative Incidence Curves From PP and ITT Analyses With IPW, TMLE, and SL **eTable 32.** MACE (Primary Definition), 2-Arm Drug Class Comparison, DPP4is vs GLP-1RAs, No ASCVD Subgroup, RD and HR Effect Measures at 2.5 Years **eFigure 33.** MACE (Primary Definition), 2-Arm Drug Class Comparison, DPP4is vs GLP-1RAs, No ASCVD and MET Subgroup, Cumulative Incidence Curves From PP and ITT Analyses With IPW, TMLE, and SL **eTable 33.** MACE (Primary Definition), 2-Arm Drug Class Comparison, DPP4is vs GLP-1RAs, No ASCVD and MET Subgroup, RD and HR Effect Measures at 2.5 Years **eFigure 34.** MACE (Primary Definition), 2-Arm Drug Class Comparison, SGLT2is vs GLP-1RAs, CONSORT Diagram **eTable 34.** MACE (Primary Definition), 2-Arm Drug Class Comparison, SGLT2is vs GLP-1RAs, Patient Characteristics at Baseline (Overall and by Medication Initiated) **eFigure 35.** MACE (Primary Definition), 2-Arm Drug Class Comparison, SGLT2is vs GLP-1RAs, Cumulative Incidence Curves From PP and ITT Analyses With IPW, TMLE, and SL **eFigure 36.** MACE (Primary Definition), 2-Arm Drug Class Comparison, SGLT2is vs GLP-1RAs, Cumulative Incidence Curves From Sensitivity PP Analyses With IPW, TMLE, and SL **eTable 35.** MACE (Primary Definition), 2-Arm Drug Class Comparison, SGLT2is vs GLP-1RAs, RD and HR Effect Measures at 2.5 Years **eFigure 37.** MACE (Primary Definition), 2-Arm Drug Class Comparison, SGLT2is vs GLP-1RAs, ASCVD Subgroup, Cumulative Incidence Curves From PP and ITT Analyses With IPW, TMLE, and SL **eTable 36.** MACE (Primary Definition), 2-Arm Drug Class Comparison, SGLT2is vs GLP-1RAs, ASCVD Subgroup, RD and HR Effect Measures at 2.5 Years **eFigure 38.** MACE (Primary Definition), 2-Arm Drug Class Comparison, SGLT2is vs GLP-1RAs, No ASCVD Subgroup, Cumulative Incidence Curves From PP and ITT Analyses With IPW, TMLE, and SL **eTable 37.** MACE (Primary Definition), 2-Arm Drug Class Comparison, SGLT2is vs GLP-1RAs, No ASCVD Subgroup, RD and HR Effect Measures at 2.5 Years **eFigure 39.** MACE (Primary Definition), 2-Arm Drug Class Comparison, SGLT2is vs GLP-1RAs, No ASCVD and MET Subgroup, Cumulative Incidence Curves From PP and ITT Analyses With IPW, TMLE, and SL **eTable 38.** MACE (Primary Definition), 2-Arm Drug Class Comparison, SGLT2is vs GLP-1RAs, No ASCVD and MET Subgroup, RD and HR Effect Measures at 2.5 Years **eFigure 40.** MACE (Primary Definition), 2-Arm Drug Class Comparison, SGLT2is vs GLP-1RAs, HF Subgroup, Cumulative Incidence Curves From PP and ITT Analyses With IPW, TMLE, and SL **eTable 39.** MACE (Primary Definition), 2-Arm Drug Class Comparison, SGLT2is vs GLP-1RAs, HF Subgroup, RD and HR Effect Measures at 2.5 Years **eFigure 41.** MACE (Primary Definition), 2-Arm Drug Class Comparison, SGLT2is vs GLP-1RAs, No HF Subgroup, Cumulative Incidence Curves From PP and ITT Analyses With IPW, TMLE, and SL **eTable 40.** MACE (Primary Definition), 2-Arm Drug Class Comparison, SGLT2is vs GLP-1RAs, No HF Subgroup, RD and HR Effect Measures at 2.5 Years **eFigure 42.** MACE (Primary Definition), 2-Arm Drug Class Comparison, SGLT2is vs GLP-1RAs, No HF and No ASCVD Subgroup, Cumulative Incidence Curves From PP and ITT Analyses With IPW, TMLE, and SL **eTable 41.** MACE (Primary Definition), 2-Arm Drug Class Comparison, SGLT2is vs GLP-1RAs, No HF and No ASCVD Subgroup, RD and HR Effect Measures at 2.5 Years **eFigure 43.** MACE (Primary Definition), 2-Arm Drug Class Comparison, SGLT2is vs GLP-1RAs, No HF and ASCVD Subgroup, Cumulative Incidence Curves From PP and ITT Analyses With IPW, TMLE, and SL **eTable 42.** MACE (Primary Definition), 2-Arm Drug Class Comparison, SGLT2is vs GLP-1RAs, No HF and ASCVD Subgroup, RD and HR Effect Measures at 2.5 Years **eFigure 44.** MACE (Primary Definition), 2-Arm Drug Class Comparison, SGLT2is vs GLP-1RAs, HF and No ASCVD Subgroup, Cumulative Incidence Curves From PP and ITT Analyses With IPW, TMLE, and SL **eTable 43.** MACE (Primary Definition), 2-Arm Drug Class Comparison, SGLT2is vs GLP-1RAs, HF and No ASCVD Subgroup, RD and HR Effect Measures at 2.5 Years **eFigure 45.** MACE (Primary Definition), 2-Arm Drug Class Comparison, SGLT2is vs GLP-1RAs, HF and ASCVD Subgroup, Cumulative Incidence Curves From PP and ITT Analyses With IPW, TMLE, and SL **eTable 44.** MACE (Primary Definition), 2-Arm Drug Class Comparison, SGLT2is vs GLP-1RAs, HF and ASCVD Subgroup, RD and HR Effect Measures at 2.5 Years **eFigure 46.** MACE (Primary Definition), 2-Arm Drug Class Comparison, SGLT2is vs GLP-1RAs, Low CKD Risk Subgroup, Cumulative Incidence Curves From PP and ITT Analyses With IPW, TMLE, and SL **eTable 45.** MACE (Primary Definition), 2-Arm Drug Class Comparison, SGLT2is vs GLP-1RAs, Low CKD Risk Subgroup, RD and HR Effect Measures at 2.5 Years **eFigure 47.** MACE (Primary Definition), 2-Arm Drug Class Comparison, SGLT2is vs GLP-1RAs, Moderate CKD Risk Subgroup, Cumulative Incidence Curves From PP and ITT Analyses With IPW, TMLE, and SL **eTable 46.** MACE (Primary Definition), 2-Arm Drug Class Comparison, SGLT2is vs GLP-1RAs, Moderate CKD Risk Subgroup, RD and HR Effect Measures at 2.5 Years **eFigure 48.** MACE (Primary Definition), 2-Arm Drug Class Comparison, SGLT2is vs GLP-1RAs, High CKD Risk Subgroup, Cumulative Incidence Curves From PP and ITT Analyses With IPW, TMLE, and SL **eTable 47.** MACE (Primary Definition), 2-Arm Drug Class Comparison, SGLT2is vs GLP-1RAs, High CKD Risk Subgroup, RD and HR Effect Measures at 2.5 Years **eFigure 49.** MACE (Primary Definition), 2-Arm Drug Class Comparison, SGLT2is vs GLP-1RAs, Male Subgroup, Cumulative Incidence Curves From PP and ITT Analyses With IPW, TMLE, and SL **eTable 48.** MACE (Primary Definition), 2-Arm Drug Class Comparison, SGLT2is vs GLP-1RAs, Male Subgroup, RD and HR Effect Measures at 2.5 Years **eFigure 50.** MACE (Primary Definition), 2-Arm Drug Class Comparison, SGLT2is vs GLP-1RAs, Female Subgroup, Cumulative Incidence Curves From PP and ITT Analyses With IPW, TMLE, and SL **eTable 49.** MACE (Primary Definition), 2-Arm Drug Class Comparison, SGLT2is vs GLP-1RAs, Female Subgroup, RD and HR Effect Measures at 2.5 Years **eFigure 51.** MACE (Primary Definition), 2-Arm Drug Class Comparison, SGLT2is vs GLP-1RAs, Age <50 Subgroup, Cumulative Incidence Curves From PP and ITT Analyses With IPW, TMLE, and SL **eTable 50.** MACE (Primary Definition), 2-Arm Drug Class Comparison, SGLT2is vs GLP-1RAs, Age <50 Subgroup, RD and HR Effect Measures at 2.5 Years **eFigure 52.** MACE (Primary Definition), 2-Arm Drug Class Comparison, SGLT2is vs GLP-1RAs, Age 50 to <65 Subgroup, Cumulative Incidence Curves From PP and ITT Analyses With IPW, TMLE, and SL **eTable 51.** MACE (Primary Definition), 2-Arm Drug Class Comparison, SGLT2is vs GLP-1RAs, Age 50 to <65 Subgroup, RD and HR Effect Measures at 2.5 Years **eFigure 53.** MACE (Primary Definition), 2-Arm Drug Class Comparison, SGLT2is vs GLP-1RAs, Age ≥65 Subgroup, Cumulative Incidence Curves From PP and ITT Analyses With IPW, TMLE, and SL **eTable 52.** MACE (Primary Definition), 2-Arm Drug Class Comparison, SGLT2is vs GLP-1RAs, Age ≥65 Subgroup, RD and HR Effect Measures at 2.5 Years **eFigure 54.** MACE (Primary Definition), 2-Arm Drug Class Comparison, SGLT2is vs GLP-1RAs, Asian Subgroup, Cumulative Incidence Curves From PP and ITT Analyses With IPW, TMLE, and SL **eTable 53.** MACE (Primary Definition), 2-Arm Drug Class Comparison, SGLT2is vs GLP-1RAs, Asian Subgroup, RD and HR Effect Measures at 2.5 Years **eFigure 55.** MACE (Primary Definition), 2-Arm Drug Class Comparison, SGLT2is vs GLP-1RAs, Black Subgroup, Cumulative Incidence Curves From PP and ITT Analyses With IPW, TMLE, and SL **eTable 54.** MACE (Primary Definition), 2-Arm Drug Class Comparison, SGLT2is vs GLP-1RAs, Black Subgroup, RD and HR Effect Measures at 2.5 Years **eFigure 56.** MACE (Primary Definition), 2-Arm Drug Class Comparison, SGLT2is vs GLP-1RAs, Native Hawaiian or Other Pacific Islander Subgroup, Cumulative Incidence Curves From PP and ITT Analyses With IPW, TMLE, and SL **eTable 55.** MACE (Primary Definition), 2-Arm Drug Class Comparison, SGLT2is vs GLP-1RAs, Native Hawaiian or Other Pacific Islander Subgroup, RD and HR Effect Measures at 2.5 Years **eFigure 57.** MACE (Primary Definition), 2-Arm Drug Class Comparison, SGLT2is vs GLP-1RAs, Multiple Race Subgroup, Cumulative Incidence Curves From PP and ITT Analyses With IPW, TMLE, and SL **eTable 56.** MACE (Primary Definition), 2-Arm Drug Class Comparison, SGLT2is vs GLP-1RAs, Multiple Race Subgroup, RD and HR Effect Measures at 2.5 Years **eFigure 58.** MACE (Primary Definition), 2-Arm Drug Class Comparison, SGLT2is vs GLP-1RAs, White Subgroup, Cumulative Incidence Curves From PP and ITT Analyses With IPW, TMLE, and SL **eTable 57.** MACE (Primary Definition), 2-Arm Drug Class Comparison, SGLT2is vs GLP-1RAs, White Subgroup, RD and HR Effect Measures at 2.5 Years **eFigure 59.** MACE (Primary Definition), 2-Arm Drug Class Comparison, SGLT2is vs GLP-1RAs, Hispanic Subgroup, Cumulative Incidence Curves From PP and ITT Analyses With IPW, TMLE, and SL **eTable 58.** MACE (Primary Definition), 2-Arm Drug Class Comparison, SGLT2is vs GLP-1RAs, Hispanic Subgroup, RD and HR Effect Measures at 2.5 Years **eFigure 60.** MACE (Primary Definition), 2-Arm Drug Agent, Exenatide vs Liraglutide, Cumulative Incidence Curves From PP and ITT Analyses With IPW, TMLE, and SL **eTable 59.** MACE (Primary Definition), 2-Arm Drug Agent, Exenatide vs Liraglutide, RD and HR Effect Measures at 2.5 Years **eFigure 61.** MACE (Primary Definition), 2-Arm Drug Agent, Semaglutide vs Liraglutide, Cumulative Incidence Curves From PP and ITT Analyses With IPW, TMLE, and SL **eTable 60.** MACE (Primary Definition), 2-Arm Drug Agent, Semaglutide vs Liraglutide, RD and HR Effect Measures at 2.5 Years **eFigure 62.** MACE (Broader Definition), 2-Arm Drug Class, Sulfonylureas vs DPP4is, Cumulative Incidence Curves From PP and ITT Analyses With IPW, TMLE, and SL **eTable 61.** MACE (Broader Definition), 2-Arm Drug Class, Sulfonylureas vs DPP4is, RD and HR Effect Measures at 2.5 Years **eFigure 63.** MACE (Broader Definition), 2-Arm Drug Class, Sulfonylureas vs SGLT2is, Cumulative Incidence Curves From PP and ITT Analyses With IPW, TMLE, and SL **eTable 62.** MACE (Broader Definition), 2-Arm Drug Class, Sulfonylureas vs SGLT2is, RD and HR Effect Measures at 2.5 Years **eFigure 64.** MACE (Broader Definition), 2-Arm Drug Class, Sulfonylureas vs GLP-1RAs, Cumulative Incidence Curves From PP and ITT Analyses With IPW, TMLE, and SL **eTable 63.** MACE (Broader Definition), 2-Arm Drug Class, Sulfonylureas vs GLP-1RAs, RD and HR Effect Measures at 2.5 Years **eFigure 65.** MACE (Broader Definition), 2-Arm Drug Class, DPP4is vs SGLT2is, Cumulative Incidence Curves From PP and ITT Analyses With IPW, TMLE, and SL **eTable 64.** MACE (Broader Definition), 2-Arm Drug Class, DPP4is vs SGLT2is, RD and HR Effect Measures at 2.5 Years **eFigure 66.** MACE (Broader Definition), 2-Arm Drug Class, DPP4is vs GLP-1RAs, Cumulative Incidence Curves From PP and ITT Analyses With IPW, TMLE, and SL **eTable 65.** MACE (Broader Definition), 2-Arm Drug Class, DPP4is vs GLP-1RAs, RD and HR Effect Measures at 2.5 Years **eFigure 67.** MACE (Broader Definition), 2-Arm Drug Class, SGLT2is vs GLP-1RAs, Cumulative Incidence Curves From PP and ITT Analyses With IPW, TMLE, and SL **eTable 66.** MACE (Broader Definition), 2-Arm Drug Class, SGLT2is vs GLP-1RAs, RD and HR Effect Measures at 2.5 Years **eFigure 68.** MACE (Primary Definition), Sulfonylureas vs DPP4is in ON TARGET DM and GRADE Trials, Cumulative Incidence Curves From PP Analyses With TMLE and SL for Emulating 2-Arm And 4-Arm Trials Compared to Cumulative Incidence Curves From the GRADE Trial **eAppendix 4.** MACE (Primary Definition), Sensitivity Analyses for Unmeasured Sources of Confounding and Selection Bias, Methodology Description **eFigure 69.** MACE (Primary Definition), Sensitivity Analyses for Unmeasured Sources of Confounding and Selection Bias, All Pairwise Comparisons From 2-Arm Cohorts, Sulfonylurea vs DPP4i Results **eFigure 70.** MACE (Primary Definition), Sensitivity Analyses for Unmeasured Sources of Confounding and Selection Bias, All Pairwise Comparisons From 2-Arm Cohorts, Sulfonylurea vs SGLT2i Results **eFigure 71.** MACE (Primary Definition), Sensitivity Analyses for Unmeasured Sources of Confounding and Selection Bias, All Pairwise Comparisons From 2-Arm Cohorts, Sulfonylurea vs GLP-1RA Results **eFigure 72.** MACE (Primary Definition), Sensitivity Analyses for Unmeasured Sources of Confounding and Selection Bias, All Pairwise Comparisons From 2-Arm Cohorts, DPP4i vs SGLT2i Results **eFigure 73.** MACE (Primary Definition), Sensitivity Analyses for Unmeasured Sources of Confounding and Selection Bias, All Pairwise Comparisons From 2-Arm Cohorts, DPP4i vs GLP-1RA Results **eFigure 74.** MACE (Primary Definition), Sensitivity Analyses for Unmeasured Sources of Confounding and Selection Bias, All Pairwise Comparisons From 2-Arm Cohorts, SGLT2i vs GLP-1RA Results
eReferences

Supplement 2.
Data Sharing Statement
