# Supplementary material for: Glucose-Lowering Medication Classes and Cardiovascular Outcomes in Patients With Type 2 Diabetes
Source: JAMA Netw Open. 2025 Oct 15;8(10):e2536100. doi: 10.1001/jamanetworkopen.2025.36100 (PMC12529185; doi:10.1001/jamanetworkopen.2025.36100)
Supplement: Supplement 1. — eAppendix 1. Detailed Description of the Analytic Approach eTable 1. Summary of the Specifications and Emulations of the Various Target Trials Considered in This Study eAppendix 2. Limitations of Prior Observational Studies eAppendix 3. Detailed Results From Primary (Per Protocol), Secondary (Intention to Treat), and Sensitivity Analyses eFigure 1. MACE (Primary Definition), 4-Arm Drug Class Comparison, Sulfonylureas vs DPP4is vs SGLT2is vs GLP1-RAs, CONSORT Diagram eTable 2. MACE (Primary Definition), 4-Arm Drug Class Comparison, Sulfonylureas vs DPP4is vs SGLT2is vs GLP1-RAs, Patient Characteristics at Baseline (Overall and by Medication Initiated) eFigure 2. MACE (Primary Definition), 4-Arm Drug Class Comparison, Sulfonylureas vs DPP4is vs SGLT2is vs GLP1-RAs, Cumulative Incidence Curves From PP Analyses With IPW, TMLE, and SL eFigure 3. MACE (Primary Definition), 4-Arm Drug Class Comparison, Sulfonylureas vs DPP4is vs SGLT2is vs GLP1-RAs, Cumulative Incidence Curves From ITT Analyses With IPW, TMLE, and SL eTable 3. MACE (Primary Definition), 4-Arm Drug Class Comparison, Sulfonylureas vs DPP4is vs SGLT2is vs GLP1-RAs, RD and HR Effect Measures for Comparing Sulfonylureas to DPP4is at 2.5 Years eTable 4. MACE (Primary Definition), 4-Arm Drug Class Comparison, Sulfonylureas vs DPP4is vs SGLT2is vs GLP1-RAs, RD and HR Effect Measures for Comparing Sulfonylureas to SGLT2is at 2.5 Years eTable 5. MACE (Primary Definition), 4-Arm Drug Class Comparison, Sulfonylureas vs DPP4is vs SGLT2is vs GLP1-RAs, RD and HR Effect Measures for Comparing Sulfonylureas to GLP-1RAs at 2.5 Years eTable 6. MACE (Primary Definition), 4-Arm Drug Class Comparison, Sulfonylureas vs DPP4is vs SGLT2is vs GLP1-RAs, RD and HR Effect Measures for Comparing DPP4is to SGLT2is at 2.5 Years eTable 7. MACE (Primary Definition), 4-Arm Drug Class Comparison, Sulfonylureas vs DPP4is vs SGLT2is vs GLP1-RAs, RD and HR Effect Measures for Comparing DPP4is to GLP-1RAs at 2.5 Years eTable 8. MACE (Primary Def [file jamanetwopen-e2536100-s001.pdf]

Supplemental Online Content

Neugebauer R, An J, Dombrowski SK, et al. Glucose-lowering medication classes and cardiovascular outcomes in patients with type 2 diabetes. *JAMA Netw Open*. 2025;8(10):e2536100. doi:10.1001/jamanetworkopen.2025.36100

eAppendix 1. Detailed Description of the Analytic Approach

eTable 1. Summary of the Specifications and Emulations of the Various Target Trials Considered in This Study

eAppendix 2. Limitations of Prior Observational Studies

eAppendix 3. Detailed Results From Primary (Per Protocol), Secondary (Intention to Treat), and Sensitivity Analyses

eFigure 1. MACE (Primary Definition), 4-Arm Drug Class Comparison, Sulfonylureas vs DPP4is vs SGLT2is vs GLP1-RAs, CONSORT Diagram

eTable 2. MACE (Primary Definition), 4-Arm Drug Class Comparison, Sulfonylureas vs DPP4is vs SGLT2is vs GLP1-RAs, Patient Characteristics at Baseline (Overall and by Medication Initiated)

eFigure 2. MACE (Primary Definition), 4-Arm Drug Class Comparison, Sulfonylureas vs DPP4is vs SGLT2is vs GLP1-RAs, Cumulative Incidence Curves From PP Analyses With IPW, TMLE, and SL

eFigure 3. MACE (Primary Definition), 4-Arm Drug Class Comparison, Sulfonylureas vs DPP4is vs SGLT2is vs GLP1-RAs, Cumulative Incidence Curves From ITT Analyses With IPW, TMLE, and SL

eTable 3. MACE (Primary Definition), 4-Arm Drug Class Comparison, Sulfonylureas vs DPP4is vs SGLT2is vs GLP1-RAs, RD and HR Effect Measures for Comparing Sulfonylureas to DPP4is at 2.5 Years

eTable 4. MACE (Primary Definition), 4-Arm Drug Class Comparison, Sulfonylureas vs DPP4is vs SGLT2is vs GLP1-RAs, RD and HR Effect Measures for Comparing Sulfonylureas to SGLT2is at 2.5 Years

eTable 5. MACE (Primary Definition), 4-Arm Drug Class Comparison, Sulfonylureas vs DPP4is vs SGLT2is vs GLP1-RAs, RD and HR Effect Measures for Comparing Sulfonylureas to GLP-1RAs at 2.5 Years

eTable 6. MACE (Primary Definition), 4-Arm Drug Class Comparison, Sulfonylureas vs DPP4is vs SGLT2is vs GLP1-RAs, RD and HR Effect Measures for Comparing DPP4is to SGLT2is at 2.5 Years

eTable 7. MACE (Primary Definition), 4-Arm Drug Class Comparison, Sulfonylureas vs DPP4is vs SGLT2is vs GLP1-RAs, RD and HR Effect Measures for Comparing DPP4is to GLP-1RAs at 2.5 Years

© 2025 Neugebauer R et al. *JAMA Network Open*.

eTable 8. MACE (Primary Definition), 4-Arm Drug Class Comparison, Sulfonylureas vs DPP4is vs SGLT2is vs GLP1-RAs, RD and HR Effect Measures for Comparing SGLT2is to DPP4is at 2.5 Years

eFigure 4. MACE (Primary Definition), 2-Arm Drug Class Comparison, Sulfonylureas vs DPP4is, CONSORT Diagram

eTable 9. MACE (Primary Definition), 2-Arm Drug Class Comparison, Sulfonylureas vs DPP4is, Patient Characteristics at Baseline (Overall and by Medication Initiated)

eFigure 5. MACE (Primary Definition), 2-Arm Drug Class Comparison, Sulfonylureas vs DPP4is, Cumulative Incidence Curves From PP and ITT Analyses With IPW, TMLE, and SL

eFigure 6. MACE (Primary Definition), 2-Arm Drug Class Comparison, Sulfonylureas vs DPP4is, Cumulative Incidence Curves From Sensitivity PP Analyses With IPW, TMLE, and SL

eTable 10. MACE (Primary Definition), 2-Arm Drug Class Comparison, Sulfonylureas vs DPP4is, RD and HR Effect Measures at 2.5 Years

eFigure 7. MACE (Primary Definition), 2-Arm Drug Class Comparison, Sulfonylureas vs DPP4is, ASCVD Subgroup, Cumulative Incidence Curves From PP and ITT Analyses With IPW, TMLE, and SL

eTable 11. MACE (Primary Definition), 2-Arm Drug Class Comparison, Sulfonylureas vs DPP4is, ASCVD Subgroup, RD and HR Effect Measures at 2.5 Years

eFigure 8. MACE (Primary Definition), 2-Arm Drug Class Comparison, Sulfonylureas vs DPP4is, No ASCVD Subgroup, Cumulative Incidence Curves From PP and ITT Analyses With IPW, TMLE, and SL

eTable 12. MACE (Primary Definition), 2-Arm Drug Class Comparison, Sulfonylureas vs DPP4is, No ASCVD Subgroup, RD and HR Effect Measures at 2.5 Years

eFigure 9. MACE (Primary Definition), 2-Arm Drug Class Comparison, Sulfonylureas vs DPP4is, No ASCVD and MET Subgroup, Cumulative Incidence Curves From PP and ITT Analyses With IPW, TMLE, and SL

eTable 13. MACE (Primary Definition), 2-Arm Drug Class Comparison, Sulfonylureas vs DPP4is, No ASCVD and MET Subgroup, RD and HR Effect Measures at 2.5 Years

eFigure 10. MACE (Primary Definition), 2-Arm Drug Class Comparison, Sulfonylureas vs SGLT2is, CONSORT Diagram

eTable 14. MACE (Primary Definition), 2-Arm Drug Class Comparison, Sulfonylureas vs SGLT2is, Patient Characteristics at Baseline (Overall and by Medication Initiated)

eFigure 11. MACE (Primary Definition), 2-Arm Drug Class Comparison, Sulfonylureas vs SGLT2is, Cumulative Incidence Curves From PP and ITT Analyses With IPW, TMLE, and SL

eFigure 12. MACE (Primary Definition), 2-Arm Drug Class Comparison, Sulfonylureas vs SGLT2is, Cumulative Incidence Curves From Sensitivity PP Analyses With IPW, TMLE, and SL

eTable 15. MACE (Primary Definition), 2-Arm Drug Class Comparison, Sulfonylureas vs SGLT2is, RD and HR Effect Measures at 2.5 Years

eFigure 13. MACE (Primary Definition), 2-Arm Drug Class Comparison, Sulfonylureas vs SGLT2is, ASCVD Subgroup, Cumulative Incidence Curves From PP and ITT Analyses With IPW, TMLE, and SL

© 2025 Neugebauer R et al. *JAMA Network Open*.

eTable 16. MACE (Primary Definition), 2-Arm Drug Class Comparison, Sulfonylureas vs SGLT2is, ASCVD Subgroup, RD and HR Effect Measures at 2.5 Years

eFigure 14. MACE (Primary Definition), 2-Arm Drug Class Comparison, Sulfonylureas vs SGLT2is, No ASCVD Subgroup, Cumulative Incidence Curves From PP and ITT Analyses With IPW, TMLE, and SL

eTable 17. MACE (Primary Definition), 2-Arm Drug Class Comparison, Sulfonylureas vs SGLT2is, No ASCVD Subgroup, RD and HR Effect Measures at 2.5 Years

eFigure 15. MACE (Primary Definition), 2-Arm Drug Class Comparison, Sulfonylureas vs SGLT2is, No ASCVD and MET Subgroup, Cumulative Incidence Curves From PP and ITT Analyses With IPW, TMLE, and SL

eTable 18. MACE (Primary Definition), 2-Arm Drug Class Comparison, Sulfonylureas vs SGLT2is, No ASCVD and MET Subgroup, RD and HR Effect Measures at 2.5 Years

eFigure 16. MACE (Primary Definition), 2-Arm Drug Class Comparison, Sulfonylureas vs GLP-1RAs, CONSORT Diagram

eTable 19. MACE (Primary Definition), 2-Arm Drug Class Comparison, Sulfonylureas vs GLP-1RAs, Patient Characteristics at Baseline (Overall and by Medication Initiated)

eFigure 17. MACE (Primary Definition), 2-Arm Drug Class Comparison, Sulfonylureas vs GLP-1RAs, Cumulative Incidence Curves From PP and ITT Analyses With IPW, TMLE, and SL

eFigure 18. MACE (Primary Definition), 2-Arm Drug Class Comparison, Sulfonylureas vs GLP-1RAs, Cumulative Incidence Curves From Sensitivity PP Analyses With IPW, TMLE, and SL

eTable 20. MACE (Primary Definition), 2-Arm Drug Class Comparison, Sulfonylureas vs GLP-1RAs, RD and HR Effect Measures at 2.5 Years

eFigure 19. MACE (Primary Definition), 2-Arm Drug Class Comparison, Sulfonylureas vs GLP-1RAs, ASCVD Subgroup, Cumulative Incidence Curves From PP and ITT Analyses With IPW, TMLE, and SL

eTable 21. MACE (Primary Definition), 2-Arm Drug Class Comparison, Sulfonylureas vs GLP-1RAs, ASCVD Subgroup, RD and HR Effect Measures at 2.5 Years

eFigure 20. MACE (Primary Definition), 2-Arm Drug Class Comparison, Sulfonylureas vs GLP-1RAs, No ASCVD Subgroup, Cumulative Incidence Curves From PP and ITT Analyses With IPW, TMLE, and SL

eTable 22. MACE (Primary Definition), 2-Arm Drug Class Comparison, Sulfonylureas vs GLP-1RAs, No ASCVD Subgroup, RD and HR Effect Measures at 2.5 Years

eFigure 21. MACE (Primary Definition), 2-Arm Drug Class Comparison, Sulfonylureas vs GLP-1RAs, No ASCVD and MET Subgroup, Cumulative Incidence Curves From PP and ITT Analyses With IPW, TMLE, and SL

eTable 23. MACE (Primary Definition), 2-Arm Drug Class Comparison, Sulfonylureas vs GLP-1RAs, No ASCVD and MET Subgroup, RD and HR Effect Measures at 2.5 Years

eFigure 22. MACE (Primary Definition), 2-Arm Drug Class Comparison, DPP4is vs SGLT2is, CONSORT Diagram

eTable 24. MACE (Primary Definition), 2-Arm Drug Class Comparison, DPP4is vs SGLT2is, Patient Characteristics at Baseline (Overall and by Medication Initiated)

© 2025 Neugebauer R et al. *JAMA Network Open*.

eFigure 23. MACE (Primary Definition), 2-Arm Drug Class Comparison, DPP4is vs SGLT2is, Cumulative Incidence Curves From PP and ITT Analyses With IPW, TMLE, and SL

eFigure 24. MACE (Primary Definition), 2-Arm Drug Class Comparison, DPP4is vs SGLT2is, Cumulative Incidence Curves From Sensitivity PP Analyses With IPW, TMLE, and SL

eTable 25. MACE (Primary Definition), 2-Arm Drug Class Comparison, DPP4is vs SGLT2is, RD and HR Effect Measures at 2.5 Years

eFigure 25. MACE (Primary Definition), 2-Arm Drug Class Comparison, DPP4is vs SGLT2is, ASCVD Subgroup, Cumulative Incidence Curves From PP and ITT Analyses With IPW, TMLE, and SL

eTable 26. MACE (Primary Definition), 2-Arm Drug Class Comparison, DPP4is vs SGLT2is, ASCVD Subgroup, RD and HR Effect Measures at 2.5 Years

eFigure 26. MACE (Primary Definition), 2-Arm Drug Class Comparison, DPP4is vs SGLT2is, No ASCVD Subgroup, Cumulative Incidence Curves From PP and ITT Analyses With IPW, TMLE, and SL

eTable 27. MACE (Primary Definition), 2-Arm Drug Class Comparison, DPP4is vs SGLT2is, No ASCVD Subgroup, RD and HR Effect Measures at 2.5 Years

eFigure 27. MACE (Primary Definition), 2-Arm Drug Class Comparison, DPP4is vs SGLT2is, No ASCVD and MET Subgroup, Cumulative Incidence Curves From PP and ITT Analyses With IPW, TMLE, and SL

eTable 28. MACE (Primary Definition), 2-Arm Drug Class Comparison, DPP4is vs SGLT2is, No ASCVD and MET Subgroup, RD and HR Effect Measures at 2.5 Years

eFigure 28. MACE (Primary Definition), 2-Arm Drug Class Comparison, DPP4is vs GLP-1RAs, CONSORT Diagram

eTable 29. MACE (Primary Definition), 2-Arm Drug Class Comparison, DPP4is vs GLP-1RAs, Patient Characteristics at Baseline (Overall and by Medication Initiated)

eFigure 29. MACE (Primary Definition), 2-Arm Drug Class Comparison, DPP4is vs GLP-1RAs, Cumulative Incidence Curves From PP and ITT Analyses With IPW, TMLE, and SL

eFigure 30. MACE (Primary Definition), 2-Arm Drug Class Comparison, DPP4is vs GLP-1RAs, Cumulative Incidence Curves From Sensitivity PP Analyses With IPW, TMLE, and SL

eTable 30. MACE (Primary Definition), 2-Arm Drug Class Comparison, DPP4is vs GLP-1RAs, RD and HR Effect Measures at 2.5 Years

eFigure 31. MACE (Primary Definition), 2-Arm Drug Class Comparison, DPP4is vs GLP-1RAs, ASCVD Subgroup, Cumulative Incidence Curves From PP and ITT Analyses With IPW, TMLE, and SL

eTable 31. MACE (Primary Definition), 2-Arm Drug Class Comparison, DPP4is vs GLP-1RAs, ASCVD Subgroup, RD and HR Effect Measures at 2.5 Years

eFigure 32. MACE (Primary Definition), 2-Arm Drug Class Comparison, DPP4is vs GLP-1RAs, No ASCVD Subgroup, Cumulative Incidence Curves From PP and ITT Analyses With IPW, TMLE, and SL

eTable 32. MACE (Primary Definition), 2-Arm Drug Class Comparison, DPP4is vs GLP-1RAs, No ASCVD Subgroup, RD and HR Effect Measures at 2.5 Years

© 2025 Neugebauer R et al. *JAMA Network Open*.

eFigure 33. MACE (Primary Definition), 2-Arm Drug Class Comparison, DPP4is vs GLP-1RAs, No ASCVD and MET Subgroup, Cumulative Incidence Curves From PP and ITT Analyses With IPW, TMLE, and SL

eTable 33. MACE (Primary Definition), 2-Arm Drug Class Comparison, DPP4is vs GLP-1RAs, No ASCVD and MET Subgroup, RD and HR Effect Measures at 2.5 Years

eFigure 34. MACE (Primary Definition), 2-Arm Drug Class Comparison, SGLT2is vs GLP-1RAs, CONSORT Diagram

eTable 34. MACE (Primary Definition), 2-Arm Drug Class Comparison, SGLT2is vs GLP-1RAs, Patient Characteristics at Baseline (Overall and by Medication Initiated)

eFigure 35. MACE (Primary Definition), 2-Arm Drug Class Comparison, SGLT2is vs GLP-1RAs, Cumulative Incidence Curves From PP and ITT Analyses With IPW, TMLE, and SL

eFigure 36. MACE (Primary Definition), 2-Arm Drug Class Comparison, SGLT2is vs GLP-1RAs, Cumulative Incidence Curves From Sensitivity PP Analyses With IPW, TMLE, and SL

eTable 35. MACE (Primary Definition), 2-Arm Drug Class Comparison, SGLT2is vs GLP-1RAs, RD and HR Effect Measures at 2.5 Years

eFigure 37. MACE (Primary Definition), 2-Arm Drug Class Comparison, SGLT2is vs GLP-1RAs, ASCVD Subgroup, Cumulative Incidence Curves From PP and ITT Analyses With IPW, TMLE, and SL

eTable 36. MACE (Primary Definition), 2-Arm Drug Class Comparison, SGLT2is vs GLP-1RAs, ASCVD Subgroup, RD and HR Effect Measures at 2.5 Years

eFigure 38. MACE (Primary Definition), 2-Arm Drug Class Comparison, SGLT2is vs GLP-1RAs, No ASCVD Subgroup, Cumulative Incidence Curves From PP and ITT Analyses With IPW, TMLE, and SL

eTable 37. MACE (Primary Definition), 2-Arm Drug Class Comparison, SGLT2is vs GLP-1RAs, No ASCVD Subgroup, RD and HR Effect Measures at 2.5 Years

eFigure 39. MACE (Primary Definition), 2-Arm Drug Class Comparison, SGLT2is vs GLP-1RAs, No ASCVD and MET Subgroup, Cumulative Incidence Curves From PP and ITT Analyses With IPW, TMLE, and SL

eTable 38. MACE (Primary Definition), 2-Arm Drug Class Comparison, SGLT2is vs GLP-1RAs, No ASCVD and MET Subgroup, RD and HR Effect Measures at 2.5 Years

eFigure 40. MACE (Primary Definition), 2-Arm Drug Class Comparison, SGLT2is vs GLP-1RAs, HF Subgroup, Cumulative Incidence Curves From PP and ITT Analyses With IPW, TMLE, and SL

eTable 39. MACE (Primary Definition), 2-Arm Drug Class Comparison, SGLT2is vs GLP-1RAs, HF Subgroup, RD and HR Effect Measures at 2.5 Years

eFigure 41. MACE (Primary Definition), 2-Arm Drug Class Comparison, SGLT2is vs GLP-1RAs, No HF Subgroup, Cumulative Incidence Curves From PP and ITT Analyses With IPW, TMLE, and SL

eTable 40. MACE (Primary Definition), 2-Arm Drug Class Comparison, SGLT2is vs GLP-1RAs, No HF Subgroup, RD and HR Effect Measures at 2.5 Years

© 2025 Neugebauer R et al. *JAMA Network Open*.

eFigure 42. MACE (Primary Definition), 2-Arm Drug Class Comparison, SGLT2is vs GLP-1RAs, No HF and No ASCVD Subgroup, Cumulative Incidence Curves From PP and ITT Analyses With IPW, TMLE, and SL

eTable 41. MACE (Primary Definition), 2-Arm Drug Class Comparison, SGLT2is vs GLP-1RAs, No HF and No ASCVD Subgroup, RD and HR Effect Measures at 2.5 Years

eFigure 43. MACE (Primary Definition), 2-Arm Drug Class Comparison, SGLT2is vs GLP-1RAs, No HF and ASCVD Subgroup, Cumulative Incidence Curves From PP and ITT Analyses With IPW, TMLE, and SL

eTable 42. MACE (Primary Definition), 2-Arm Drug Class Comparison, SGLT2is vs GLP-1RAs, No HF and ASCVD Subgroup, RD and HR Effect Measures at 2.5 Years

eFigure 44. MACE (Primary Definition), 2-Arm Drug Class Comparison, SGLT2is vs GLP-1RAs, HF and No ASCVD Subgroup, Cumulative Incidence Curves From PP and ITT Analyses With IPW, TMLE, and SL

eTable 43. MACE (Primary Definition), 2-Arm Drug Class Comparison, SGLT2is vs GLP-1RAs, HF and No ASCVD Subgroup, RD and HR Effect Measures at 2.5 Years

eFigure 45. MACE (Primary Definition), 2-Arm Drug Class Comparison, SGLT2is vs GLP-1RAs, HF and ASCVD Subgroup, Cumulative Incidence Curves From PP and ITT Analyses With IPW, TMLE, and SL

eTable 44. MACE (Primary Definition), 2-Arm Drug Class Comparison, SGLT2is vs GLP-1RAs, HF and ASCVD Subgroup, RD and HR Effect Measures at 2.5 Years

eFigure 46. MACE (Primary Definition), 2-Arm Drug Class Comparison, SGLT2is vs GLP-1RAs, Low CKD Risk Subgroup, Cumulative Incidence Curves From PP and ITT Analyses With IPW, TMLE, and SL

eTable 45. MACE (Primary Definition), 2-Arm Drug Class Comparison, SGLT2is vs GLP-1RAs, Low CKD Risk Subgroup, RD and HR Effect Measures at 2.5 Years

eFigure 47. MACE (Primary Definition), 2-Arm Drug Class Comparison, SGLT2is vs GLP-1RAs, Moderate CKD Risk Subgroup, Cumulative Incidence Curves From PP and ITT Analyses With IPW, TMLE, and SL

eTable 46. MACE (Primary Definition), 2-Arm Drug Class Comparison, SGLT2is vs GLP-1RAs, Moderate CKD Risk Subgroup, RD and HR Effect Measures at 2.5 Years

eFigure 48. MACE (Primary Definition), 2-Arm Drug Class Comparison, SGLT2is vs GLP-1RAs, High CKD Risk Subgroup, Cumulative Incidence Curves From PP and ITT Analyses With IPW, TMLE, and SL

eTable 47. MACE (Primary Definition), 2-Arm Drug Class Comparison, SGLT2is vs GLP-1RAs, High CKD Risk Subgroup, RD and HR Effect Measures at 2.5 Years

eFigure 49. MACE (Primary Definition), 2-Arm Drug Class Comparison, SGLT2is vs GLP-1RAs, Male Subgroup, Cumulative Incidence Curves From PP and ITT Analyses With IPW, TMLE, and SL

eTable 48. MACE (Primary Definition), 2-Arm Drug Class Comparison, SGLT2is vs GLP-1RAs, Male Subgroup, RD and HR Effect Measures at 2.5 Years

© 2025 Neugebauer R et al. *JAMA Network Open*.

eFigure 50. MACE (Primary Definition), 2-Arm Drug Class Comparison, SGLT2is vs GLP-1RAs, Female Subgroup, Cumulative Incidence Curves From PP and ITT Analyses With IPW, TMLE, and SL

eTable 49. MACE (Primary Definition), 2-Arm Drug Class Comparison, SGLT2is vs GLP-1RAs, Female Subgroup, RD and HR Effect Measures at 2.5 Years

eFigure 51. MACE (Primary Definition), 2-Arm Drug Class Comparison, SGLT2is vs GLP-1RAs, Age <50 Subgroup, Cumulative Incidence Curves From PP and ITT Analyses With IPW, TMLE, and SL

eTable 50. MACE (Primary Definition), 2-Arm Drug Class Comparison, SGLT2is vs GLP-1RAs, Age <50 Subgroup, RD and HR Effect Measures at 2.5 Years

eFigure 52. MACE (Primary Definition), 2-Arm Drug Class Comparison, SGLT2is vs GLP-1RAs, Age 50 to <65 Subgroup, Cumulative Incidence Curves From PP and ITT Analyses With IPW, TMLE, and SL

eTable 51. MACE (Primary Definition), 2-Arm Drug Class Comparison, SGLT2is vs GLP-1RAs, Age 50 to <65 Subgroup, RD and HR Effect Measures at 2.5 Years

eFigure 53. MACE (Primary Definition), 2-Arm Drug Class Comparison, SGLT2is vs GLP-1RAs, Age ≥65 Subgroup, Cumulative Incidence Curves From PP and ITT Analyses With IPW, TMLE, and SL

eTable 52. MACE (Primary Definition), 2-Arm Drug Class Comparison, SGLT2is vs GLP-1RAs, Age ≥65 Subgroup, RD and HR Effect Measures at 2.5 Years

eFigure 54. MACE (Primary Definition), 2-Arm Drug Class Comparison, SGLT2is vs GLP-1RAs, Asian Subgroup, Cumulative Incidence Curves From PP and ITT Analyses With IPW, TMLE, and SL

eTable 53. MACE (Primary Definition), 2-Arm Drug Class Comparison, SGLT2is vs GLP-1RAs, Asian Subgroup, RD and HR Effect Measures at 2.5 Years

eFigure 55. MACE (Primary Definition), 2-Arm Drug Class Comparison, SGLT2is vs GLP-1RAs, Black Subgroup, Cumulative Incidence Curves From PP and ITT Analyses With IPW, TMLE, and SL

eTable 54. MACE (Primary Definition), 2-Arm Drug Class Comparison, SGLT2is vs GLP-1RAs, Black Subgroup, RD and HR Effect Measures at 2.5 Years

eFigure 56. MACE (Primary Definition), 2-Arm Drug Class Comparison, SGLT2is vs GLP-1RAs, Native Hawaiian or Other Pacific Islander Subgroup, Cumulative Incidence Curves From PP and ITT Analyses With IPW, TMLE, and SL

eTable 55. MACE (Primary Definition), 2-Arm Drug Class Comparison, SGLT2is vs GLP-1RAs, Native Hawaiian or Other Pacific Islander Subgroup, RD and HR Effect Measures at 2.5 Years

eFigure 57. MACE (Primary Definition), 2-Arm Drug Class Comparison, SGLT2is vs GLP-1RAs, Multiple Race Subgroup, Cumulative Incidence Curves From PP and ITT Analyses With IPW, TMLE, and SL

eTable 56. MACE (Primary Definition), 2-Arm Drug Class Comparison, SGLT2is vs GLP-1RAs, Multiple Race Subgroup, RD and HR Effect Measures at 2.5 Years

© 2025 Neugebauer R et al. *JAMA Network Open*.

eFigure 58. MACE (Primary Definition), 2-Arm Drug Class Comparison, SGLT2is vs GLP-1RAs, White Subgroup, Cumulative Incidence Curves From PP and ITT Analyses With IPW, TMLE, and SL

eTable 57. MACE (Primary Definition), 2-Arm Drug Class Comparison, SGLT2is vs GLP-1RAs, White Subgroup, RD and HR Effect Measures at 2.5 Years

eFigure 59. MACE (Primary Definition), 2-Arm Drug Class Comparison, SGLT2is vs GLP-1RAs, Hispanic Subgroup, Cumulative Incidence Curves From PP and ITT Analyses With IPW, TMLE, and SL

eTable 58. MACE (Primary Definition), 2-Arm Drug Class Comparison, SGLT2is vs GLP-1RAs, Hispanic Subgroup, RD and HR Effect Measures at 2.5 Years

eFigure 60. MACE (Primary Definition), 2-Arm Drug Agent, Exenatide vs Liraglutide, Cumulative Incidence Curves From PP and ITT Analyses With IPW, TMLE, and SL

eTable 59. MACE (Primary Definition), 2-Arm Drug Agent, Exenatide vs Liraglutide, RD and HR Effect Measures at 2.5 Years

eFigure 61. MACE (Primary Definition), 2-Arm Drug Agent, Semaglutide vs Liraglutide, Cumulative Incidence Curves From PP and ITT Analyses With IPW, TMLE, and SL

eTable 60. MACE (Primary Definition), 2-Arm Drug Agent, Semaglutide vs Liraglutide, RD and HR Effect Measures at 2.5 Years

eFigure 62. MACE (Broader Definition), 2-Arm Drug Class, Sulfonylureas vs DPP4is, Cumulative Incidence Curves From PP and ITT Analyses With IPW, TMLE, and SL

eTable 61. MACE (Broader Definition), 2-Arm Drug Class, Sulfonylureas vs DPP4is, RD and HR Effect Measures at 2.5 Years

eFigure 63. MACE (Broader Definition), 2-Arm Drug Class, Sulfonylureas vs SGLT2is, Cumulative Incidence Curves From PP and ITT Analyses With IPW, TMLE, and SL

eTable 62. MACE (Broader Definition), 2-Arm Drug Class, Sulfonylureas vs SGLT2is, RD and HR Effect Measures at 2.5 Years

eFigure 64. MACE (Broader Definition), 2-Arm Drug Class, Sulfonylureas vs GLP-1RAs, Cumulative Incidence Curves From PP and ITT Analyses With IPW, TMLE, and SL

eTable 63. MACE (Broader Definition), 2-Arm Drug Class, Sulfonylureas vs GLP-1RAs, RD and HR Effect Measures at 2.5 Years

eFigure 65. MACE (Broader Definition), 2-Arm Drug Class, DPP4is vs SGLT2is, Cumulative Incidence Curves From PP and ITT Analyses With IPW, TMLE, and SL

eTable 64. MACE (Broader Definition), 2-Arm Drug Class, DPP4is vs SGLT2is, RD and HR Effect Measures at 2.5 Years

eFigure 66. MACE (Broader Definition), 2-Arm Drug Class, DPP4is vs GLP-1RAs, Cumulative Incidence Curves From PP and ITT Analyses With IPW, TMLE, and SL

eTable 65. MACE (Broader Definition), 2-Arm Drug Class, DPP4is vs GLP-1RAs, RD and HR Effect Measures at 2.5 Years

eFigure 67. MACE (Broader Definition), 2-Arm Drug Class, SGLT2is vs GLP-1RAs, Cumulative Incidence Curves From PP and ITT Analyses With IPW, TMLE, and SL

© 2025 Neugebauer R et al. *JAMA Network Open*.

eTable 66. MACE (Broader Definition), 2-Arm Drug Class, SGLT2is vs GLP-1RAs, RD and HR Effect Measures at 2.5 Years

eFigure 68. MACE (Primary Definition), Sulfonylureas vs DPP4is in ON TARGET DM and GRADE Trials, Cumulative Incidence Curves From PP Analyses With TMLE and SL for Emulating 2-Arm And 4-Arm Trials Compared to Cumulative Incidence Curves From the GRADE Trial

eAppendix 4. MACE (Primary Definition), Sensitivity Analyses for Unmeasured Sources of Confounding and Selection Bias, Methodology Description

eFigure 69. MACE (Primary Definition), Sensitivity Analyses for Unmeasured Sources of Confounding and Selection Bias, All Pairwise Comparisons From 2-Arm Cohorts, Sulfonylurea vs DPP4i Results

eFigure 70. MACE (Primary Definition), Sensitivity Analyses for Unmeasured Sources of Confounding and Selection Bias, All Pairwise Comparisons From 2-Arm Cohorts, Sulfonylurea vs SGLT2i Results

eFigure 71. MACE (Primary Definition), Sensitivity Analyses for Unmeasured Sources of Confounding and Selection Bias, All Pairwise Comparisons From 2-Arm Cohorts, Sulfonylurea vs GLP-1RA Results

eFigure 72. MACE (Primary Definition), Sensitivity Analyses for Unmeasured Sources of Confounding and Selection Bias, All Pairwise Comparisons From 2-Arm Cohorts, DPP4i vs SGLT2i Results

eFigure 73. MACE (Primary Definition), Sensitivity Analyses for Unmeasured Sources of Confounding and Selection Bias, All Pairwise Comparisons From 2-Arm Cohorts, DPP4i vs GLP-1RA Results

eFigure 74. MACE (Primary Definition), Sensitivity Analyses for Unmeasured Sources of Confounding and Selection Bias, All Pairwise Comparisons From 2-Arm Cohorts, SGLT2i vs GLP-1RA Results

eReferences

This supplemental material has been provided by the authors to give readers additional information about their work.

© 2025 Neugebauer R et al. *JAMA Network Open*.

eAppendix 1. Detailed Description of the Analytic Approach

In practice, not all patients will have all the same drug initiation options to choose from due to differences in contraindications or history of prior treatment. For example, a patient who recently used a sulfonylurea and DPP4 will only be considered for GLP1-RA or SGLT2 initiation. In addition, in a given healthcare system, treatment options might also be limited by formularies or other logistical constraints. For these reasons, we aimed to emulate several target trials to generate evidence that is adapted to the particular decision faced by a given patient based on their history of treatments, contraindications, and available treatment options at the point of care. For each emulated target trial (see eTable 1), a separate cohort of eligible patients and their index dates were identified before assembling longitudinal data on each cohort member. The following general eligibility criteria were used to identify each cohort: 1) a pharmacy dispensing for one of the medications compared in the emulated RCT on a date on or between January 1, 2014 and December 31, 2021; 2) continuous health plan and pharmacy coverage in the prior 2 years (allowing gaps of 92 days or less); 3) no dispensing for any of the medications compared in the RCT in the prior 2 years (washout period); 3) evidence of type 2 diabetes in the prior 2 years; 4) at least one HbA1c test in the prior 2 years; 5) no evidence of shortened life expectancy (dementia, metastatic cancer, palliative or hospice care) in the prior two years; 6) no evidence of cystic fibrosis in the prior 2 years; 7) not pregnant; 8) at least 18 years of age; and 9) no prior evidence of contraindications to any of the medications specifically compared. The following drug class-specific contraindications were considered: pancreatitis for GLP1-RA and DPP4i, alanine transaminase (ALT) equal to or greater than three times the upper limit of normal for SU, pyelonephritis for SGLT2i, necrotizing fasciitis for SGLT2i, eGFR<30 ml/min/1.73m^2 for SGLT2i, and multiple endocrine neoplasia type 2 for GLP-1RA. Using the *LtAtStructR* R package,[1] the longitudinal data on each cohort were consolidated into a single analytic dataset with temporally ordered measurements on covariates, exposures, right-censoring events, and outcomes updated every 30 days from index date until the end of follow-up. In this dataset, covariates that were not measured at baseline or during follow-up were encoded using the missingness indicator approach.[2-6]The resulting analytic dataset encodes the complex longitudinal data structure introduced for evaluating the effect of time-varying exposures (treatment plans) in problems with time-varying sources of confounding and attrition bias (informative right-censoring) using G-methods.[7]

The missingness indicator approach used in the study follows common practice for fitting marginal structural models in cohort studies. It consists of

1) Creating separate indicators of covariate measurement at baseline and for each 30-day follow-up interval;
2) Replacing unobserved baseline values with an imputed value, and unobserved time-varying values with the last measured value (if any and otherwise with the imputed baseline value). Imputed values can be arbitrary (e.g., replace unmeasured baseline value with 0 for continuous variables or “unknown” for categorical value) or based on a more or less complex imputation approach. Due to the large adjustment set in this study, we used simplistic imputation approach for all partially observed covariates. The approach assigns the mode and mean (of the measured baseline values) for categorical and continuous covariates, respectively;
3) Combining the covariates defined in the prior two steps to define the covariate adjustment set used to estimate nuisance parameters (i.e., propensity scores and outcome regressions).

Valid effect estimation with the missingness indicator approach relies on the assumption that the information content of the adjustment set defined in step 3 is sufficient to uphold the usual sequential randomization assumption (i.e., no unmeasured sources of confounding or attrition bias). This assumption is often incorrectly construed as requiring that the baseline imputation models and last observed value carried imputation result in correctly uncovering the true value (or distribution) of the unobserved covariate measurements. Instead, the missingness indicator approach rests largely on the assumption that unobserved covariate values are not missing information, i.e., information required to identify the causal effect of interest.[4,6]

We describe a common covariate scenario where such an assumption is reasonable before describing a counterexample where this assumption is violated.

For a covariate such as a laboratory measurement (e.g., eGFR), it is plausible to assume that a patient’s unobserved lab test value (because it was not ordered or not performed) is unlikely to influence a clinician’s (or patient’s) treatment decision and instead the decision might be influenced by an earlier

© 2025 Neugebauer G et al. *JAMA Network Open*.

measurement of the covariate, The use of last observed carried forward approach can then be viewed as a means to define a covariate that tracks the relevant potential confounding information (i.e., the last information known to the clinician/patient). Such unobserved covariate information should then not be construed as missing since this information is not required for effect identification (i.e. the sequential randomization assumption holds without this information).

eTable 1. Summary of the Specifications and Emulations of the Various Target Trials Considered in This Study

Causal Estimands	Eligibility criteria† (common to all emulated target trials):<br>1) a pharmacy dispensing for one of the medications compared in the emulated RCT on a date on or between January 1, 2014 and December 31, 2021; 2) continuous health plan and pharmacy coverage in the prior 2 years (allowing gaps of 92 days or less); 3) no dispensing for any of the medications compared in the RCT in the prior 2 years (washout period); 3) evidence of type 2 diabetes in the prior 2 years; 4) at least one A1c test in the prior 2 years; 5) no evidence of shortened life expectancy (dementia, metastatic cancer, palliative or hospice care) in the prior two years; 6) no evidence of cystic fibrosis in the prior 2 years; 7) not pregnant; 8) at least 18 years of age; and 9) no prior evidence of contraindications to any of the medications specifically compared. The following drug class-specific contraindications were considered: pancreatitis for GLP1-RA and DPP4i, alanine transaminase equal to or greater than three times the upper limit of normal for SU, pyelonephritis for SGLT2i, necrotizing fasciitis for SGLT2i, eGFR<30 for SGLT2i, and multiple endocrine neoplasia type 2 for GLP-1RA.<br><br>In the target trial, criterion 1 corresponds to a trial participant agreeing to initiate one of the medications compared in the trial and formally requesting a pharmacy dispensing for such a medication by visiting a pharmacy in person or ordering the medication online for example. The actual medication dispensed would then be randomized at the time of the pharmacy dispensing request (i.e., index date).<br><br>Treatment strategies†:<br>Each protocol requires: medication initiation on index date followed by sustained exposure to this medication and no initiation of the comparator medication(s).<br>• 2-arm trial: 1) all 6 pairwise contrasts of DPP4, GLP-1RA, SGLT2i, and SU; 2) exenatide versus liraglutide; 2) semaglutide versus liraglutide.<br>• 4-arm trial (only class comparisons): DPP4 versus GLP-1RA versus SGLT2i versus SU<br><br>Assignment: unmasked<br><br>Outcomes†: 3-point MACE defined as earliest of nonfatal stroke, nonfatal myocardial infarction, and cardiovascular (CV) death<br><br>Start of follow-up: date(s) when all eligibility criteria are met<br>End of follow-up: earliest of outcome occurrence of a censoring event (see list below)<br><br>Causal contrasts‡ (identical for the target trial and its emulation owing to the nonparametric marginal structural model adopted in the study):<br>• Average risk difference through 30 months (2.5 years) = difference between two cumulative incidence curves through month 30.<br>• Risk difference at 30 months of follow-up<br>• The two contrasts above are evaluated overall (all new users) and within subgroups† of participants defined by baseline covariates (see Table 3 of manuscript for the list of subgroups considered by emulated trial).
Identifiability Assumptions	Loss to follow-up events†: disenrollment from the health plan or loss of pharmacy coverage, non-CV death, unknown cause of death, administrative end of follow-up (12/31/2021).<br><br>The assumed sequential randomization assumption (i.e., exchangeability/no unmeasured sources of confounding or attrition bias) rests of 400 baseline and time-varying covariates†.
Estimators	Trial estimator: Kaplan-Meier estimator (emulated by the discrete-time Kaplan-Meier estimator in this study based on 30-day intervals and referred to thereafter as the unadjusted estimator):<br><br>Emulation based on follow-up time discretization using the 30-day unit of time (i.e. adjusted estimator in this study):<br>• Primary: TMLE with SL estimation of propensity scores (learners considered are listed in Appendix Section 1) and LASSO estimation of outcome regressions.<br>• Subgroup analyses: the primary estimator is replicated within each subgroup considered.<br>• Sensitivity analyses: 1) IPW estimator; 2) various weight truncation levels; 3) MACE defined based on a broader list of CV deaths; 4) noMBS protocols: 2-arm class-level RCTs with modified treatment strategies that preclude metabolic bariatric surgery (MBS) during following and that exclude participants with a history of MBS at index date; 5) no3 protocols: 2-arm class-level RCTs with modified treatment strategies that preclude initiation of the other three drug classes (not just the comparator drug class); 6) robustness of findings to plausible levels of bias from residual unmeasured sources of confounding or attrition bias.

† Data element definitions provided at: https://romainkp.github.io/OTDM_Data_Dictionary.github.io/
‡ Implicitly assumes no loss to follow-up

For other covariates such as race/ethnicity, the assumption just described might not be plausible because although the patient's race/ethnicity might not be captured in the EHR, this information could influence clinicians when making new treatment decisions so the missing race/ethnicity information in the EHR

© 2025 Neugebauer R et al. *JAMA Network Open*.

would then be needed for causal estimand identification. In this case, the missingness indicator approach could result in the definition of a covariate adjustment set for which the sequential randomization assumption does not hold. This assumption violation from partially missing covariate information can be approached just like the more stringent violation of the sequential randomization due to completely missing covariate data (e.g. diet and exercise) using sensitivity analyses such as those implemented in this study (g value, see section D1 of the Appendix). Other approaches could be considered such as methods that aim to correctly estimate the distribution of such unobserved covariates (e.g., multiple imputation) but these approaches often rely on strong parametric assumptions or themselves assume that the information in the observed data is sufficient to correctly guess/estimate the required missing information.

Because the missingness indicator approach does not rely on correctly fitting an imputation model (i.e., correct estimation of another nuisance parameter) and instead assumes that the sequential randomization assumption holds for the covariates included in the adjustment set (as they are defined by the three-step approach above), the standard error of the effect estimates do not need to account for the variability resulting from fitting imputation models as would be the case with multiple imputation for example.

The same analytic dataset was used to implement both PP and ITT analyses[8] under nonparametric marginal structural models[9] using targeted learning[10] to estimate the cumulative incidence curve for each treatment arm of the emulated target trial. Two-arm contrasts of these curves defined the primary study effect measures: the risk difference (RD) at 2.5 years of follow-up and the average risk difference (ARD) over 2.5 years of follow-up, i.e., the difference in the area under any two cumulative incidence curves.[11,12] For each RD, we computed the point estimate, 95% confidence intervals, and the p value for the 2-sided test of the null hypothesis that the RD is zero. For the ARD, we computed the p value for the 2-sided test of the null hypothesis that the ARD is equal to 0 (i.e., the difference between the areas under two cumulative incidence curves through 2.5 years is zero). The ARD p value is the analog of the log rank test computed in RCTs to compare two survival curves based on Kaplan-Meier estimates. Targeted learning is a general estimation procedure that combined a doubly robust estimation approach known as Targeted Minimum Loss based Estimation[13,14] (TMLE) combined with machine learning to estimate propensity scores and a sequence of outcome regressions. The specific TMLE algorithm used in this study was detailed in prior work[15] which also showed that TMLE performance to address both baseline and time-varying sources of confounding and attrition bias is on par with inverse probability weighting (IPW) estimation[16,17] while TMLE can also improve estimation efficiency over IPW. In rare cases when TMLE estimates of cumulative incidence curves were not monotone increasing, antitonic regression[18,19] was applied to the vector of TMLE point estimates. We note that the TMLE implementation in this work is based on complier-stratified estimates of the conditional expectations for each outcome, i.e., estimates based on only data from patients who followed the treatment protocol from index date until outcome collection.

For both PP and ITT analyses, outcome regressions were estimated using LASSO[20] and propensity scores were estimated using an ensemble learning estimation approach[21] known as Super Learning[22] (SL) and its implementation in the *SL3* R package[23]. The SL library of learners included logistic regression, LASSO, recursive partitioning and regression trees, random forest, and extreme gradient boosting. Learners were combined with feature screening such that learners were fitted based on the entire covariate set or only the top 5, 10, 20, 30, 50, 100, 150, 300, or 400 covariates most statistically significantly associated with the propensity score outcome. The p value for the Pearson correlation coefficient measuring the association between each covariate and the propensity score outcome was used to rank covariates. For ITT and PP analyses, we estimated propensity scores for treatment initiation and each type of right-censoring events at each time point separately. For PP analyses, we also estimated propensity scores for treatment continuation (i.e., no treatment interruption or crossover) at each time point separately. While in PP analysis, follow-up ends at the time of a right-censoring event or treatment protocol deviation in each arm (e.g., crossover or discontinuation of the treatment initiated on index date), in the ITT analysis, follow-up only ends at the time of a right-censoring event (e.g., patients continue to contribute outcomes to their treatment arm even after treatment crossover or discontinuation of the initial therapy started on index date). Conservative estimates of the standard errors for the RD and ARD were obtained analytically using the TMLE influence curve and the delta method.[24] This analytic approach accounts for repeated longitudinal observations on the same patient when cohort eligibility criteria were met more than once by the same patient. Subgroup analyses by baseline patient characteristics were implemented by replicating the PP and ITT analytic approach just described (including refitting all propensity scores) using only data from the patients in the subgroup considered. Effect modification was assessed by testing the null hypothesis that the RDs from two patient subgroups (e.g., males versus females) were equal based on a conservative estimate of the standard error for the difference of RDs obtained analytically using the delta method.

© 2025 Neugebauer R et al. *JAMA Network Open*.

We examined the sensitivity of findings from the primary PP analyses emulating 2-arm RCTs by replicating the same analytic approach with the difference that protocols in each treatment arm were modified to also preclude either 1) exposure to MBS, or 2) initiation of any of the other three classes of glucose lowering medications considered in this study (as opposed to only the single comparator medication class in the 2-arm RCT). We refer to the first and second type of sensitivity analyses as "noMBS PP analyses" and "no3 PP analyses".

For instance, the primary PP analysis that emulates a 2-arm RCT for comparing SU to SGLT2 evaluates treatment protocols in each arm that allow patient to undergo MBS during follow-up. The sensitivity noMBS PP analysis emulates a similar 2-arm RCT with the difference that 1) patients with a history of MBS prior to cohort entry are excluded from the analysis, and 2) the protocol in both the SU and SGLT2 arms precludes patients from undergoing an MBS procedure during follow-up. The sensitivity no3 PP analysis emulates a 2-arm RCT in which 1) the protocol for the SU treatment arm precludes initiation of not only SGLT2i but also now DPP4i and GLP-1RA, and 2) the protocol for the SGLT2i treatment arm precludes initiation of not only SU but also now DPP4i and GLP-1RA.

All primary and sensitivity analyses above were replicated in additional sensitivity analyses based on IPW estimation with the same SL propensity score estimates and also using estimates from logistic models. The specific IPW implementation was detailed in Section 3.1.3 of prior work.[25] In short, the approach derives separate IPW estimates of the discrete-time hazard at each time point and separately for each treatment arm (i.e., a *saturated* logistic MSM is fitted) before consolidating these estimates into estimates of the same cumulative incidence curves, RD, and ARD at 2.5 years as those evaluated with targeted learning. Unadjusted estimates were derived using this same hazard-based estimation approach but without weighting (i.e., using equal weights set to 1 for all person-time observations). In addition, the same IPW implementation was replicated using a non-saturated, working,[26] logistic MSM that assumes proportionality (i.e., constant hazard ratios over time) to derive hazard ratio (HR) effect measures.

All TMLE and IPW estimates were implemented using untruncated and truncated[27,28] weights (e.g., the 99th percentile of the weight distribution for IPW, and absolute truncation values defaulting to 20 for IPW and 200 for TMLE). Finally, we implemented sensitivity analyses to unmeasured confounding including the calculation of g-values[10,29] to understand the level of unmeasured confounding or sources of attrition bias that would need to exist to negate statistically significant findings (see result in eAppendix 4 below for details). All estimation procedures were implemented using the *stremr* R package.[30]

eAppendix 2. Limitations of Prior Observational Studies

Our finding of a beneficial effect of GLP-1RA over SGLT2i is consistent with a prior European observational study.[31] However, our overall results are not always consistent with findings from other large comparative observational studies that assessed the same comparative effectiveness questions.[31-38] The inconsistency might be explained in part by differences in study subjects and by the several methodological limitations or analytic choices of prior published work.

First, many authors favored the evaluation of ITT effects, i.e., the effects of initiating one drug versus another, despite high rates of subsequent treatment discontinuation or crossover events (as demonstrated in our study results and elsewhere)[39,40] which are known to possibly result in misleading CER evidence and, in particular, in studies with active comparators.[8] Second, when authors favored the evaluation of PP effects, i.e., the effects of *sustained* exposure to one drug versus another, their evaluation was typically based on an "as treated" analysis where follow-up is simply artificially right-censored at the time of treatment discontinuation with no attempt to account for time-varying risk factors for MACE that might also impact treatment discontinuation or crossover. It is well established that ignoring such time-varying covariates can lead to residual confounding bias and that accounting for time-dependent confounders requires the use of advanced causal inference methods such as IPW and TMLE because alternate approaches such as matching methods cannot adequately address time-varying confounders that are themselves impacted by early treatment decisions. Third, most authors ignored potential collider bias (also known as selection bias) that can result from loss to follow-up (attrition) when either baseline or time-varying covariates impact both loss to follow-up and MACE risk. Fourth, most authors make arbitrary assumptions when estimating propensity scores such as assuming a logistic model with no interaction terms which can result in residual confounding or increase estimation variability from inadequate adjustment for observed confounders instead of using more nonparametric covariate adjustment methods such as Super Learning[25,41-44]. Fifth, most authors evaluated effect measures defined by parametric causal models (e.g., Cox proportional hazards

© 2025 Neugebauer R et al. *JAMA Network Open*.

marginal structural models) that rely on arbitrary assumptions such as constant hazard ratios over time which can result in misleading inference. For example, in our PP analysis comparing SU to DPP4i, the nonparametric MSM fit we obtained with TMLE and IPW suggest that hazard ratios are not constant over time with an increased risk from DPP4i growing much larger around month 21 of follow-up (similar to what was observed in the GRADE study, eFigure 68). When fitting an MSM that incorrectly assumes proportionality using IPW in our study, the resulting HR estimates we obtained did not reveal the statistically significant deleterious effect of DPP4i compared to SU that we found in our analyses based on a nonparametric MSM (eTable 10). This apparent inconsistency can be explained by the fact that the single HR measure defined by the mis-specified MSM (that incorrectly assumes proportionality) is a weighted average of the non-constant hazard ratios over time with larger weights given to early time points where sample sizes are larger and where the HRs are similar between SU and DPP4i users. Because HRs in the first 21 months of follow-up are similar in the SU and DPP4i exposure groups, the resulting average HR over 30 months of follow-up did not reveal the increased cumulative MACE risk from DPP4i at 2.5 years that is observed in PP analyses based on a nonparametric MSM (that does not incorrectly assume proportionality). We conjecture that findings from other studies that assumed proportionality to derive a single HR effect measure could also be biased for the reasons just described and as exemplified by results from sensitivity analyses in our study. Sixth, many authors did not have access to cause of death information and instead evaluated a modified MACE outcome which included all-cause mortality instead of only CV mortality as done in our study and CVOTs. Finally, many authors relied on claim-only data which did not capture critical confounding information available in granular EHR data (e.g., weight, height, systolic and diastolic blood pressure, smoking status, lipid values and quantitative renal function tests) which could have resulted in residual bias from unmeasured confounding that was however captured in this study.

© 2025 Neugebauer R et al. *JAMA Network Open*.

eAppendix 3. Detailed Results From Primary (Per Protocol), Secondary (Intention to Treat), and Sensitivity Analyses

eFigure 1. MACE (Primary Definition), 4-Arm Drug Class Comparison, Sulfonylureas vs DPP4is vs SGLT2is vs GLP1-RAs, CONSORT Diagram
Flow diagram describing the inclusion and exclusion steps and counts leading to the creation of the cohort for emulating the 4-arm RCT to compare the risk of MACE in new users of SU, DPP4i, GLP-1RA, and SGLT2i along with sample sizes and counts for each observed end of follow-up type by treatment initiated at cohort entry.

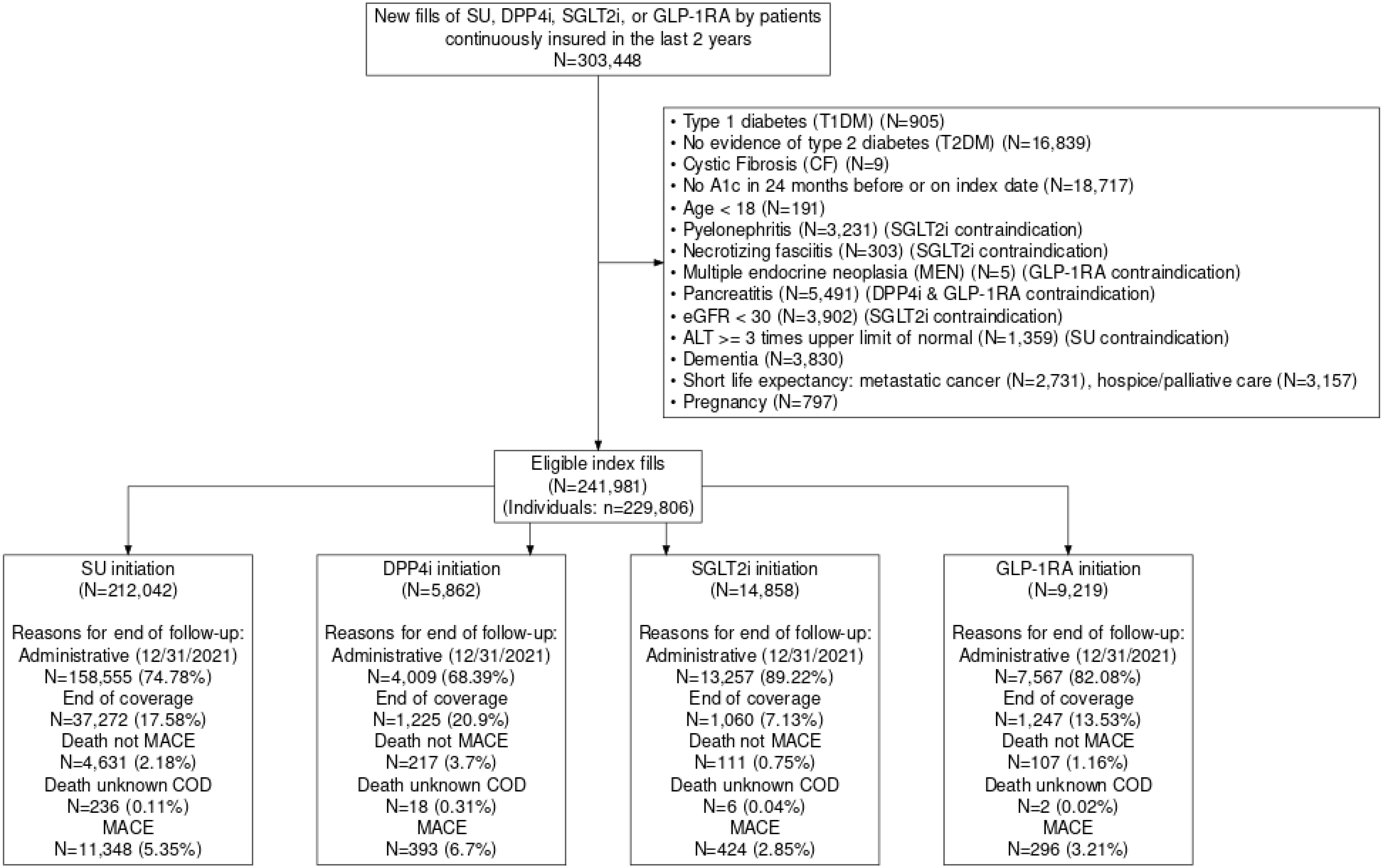

© 2025 Neugebauer R et al. *JAMA Network Open*.

eTable 2. MACE (Primary Definition), 4-Arm Drug Class Comparison, Sulfonylureas vs DPP4is vs SGLT2is vs GLP1-RAs, Patient Characteristics at Baseline (Overall and by Medication Initiated)

Summary statistics of the baseline values for selected covariates in the cohort of patients used to emulate a 4-arm RCT for comparing SU, DPP4i, SGLT2i, and GLP-1RA. For each continuous variable, the mean and standard deviation are displayed for all patients in the cohort (last column) and by drug class initiated at cohort entry. For each categorical variable and for each possible level of that variable, the count and proportion are displayed instead.

	SU<br>n = 212,042	DPP4i<br>n = 5,862	GLP-1RA<br>n = 9,219	SGLT2i<br>n = 14,858	Total<br>n = 241,981
**Demographics**					
Age	56.91 (12.91)	61.69 (12.88)	55.95 (12.34)	60.51 (11.95)	57.21 (12.88)
Agegrp					
<45	36,000 (16.98%)	546 (9.31%)	1,663 (18.04%)	1,503 (10.12%)	39,712 (16.41%)
[45-65)	116,736 (55.05%)	2,844 (48.52%)	5,203 (56.44%)	7,470 (50.28%)	132,253 (54.65%)
[65-75)	41,264 (19.46%)	1,528 (26.07%)	1,909 (20.71%)	4,306 (28.98%)	49,007 (20.25%)
>=75	18,042 (8.51%)	944 (16.1%)	444 (4.82%)	1,579 (10.63%)	21,009 (8.68%)
Ethnicity					
Hispanic	78,965 (37.24%)	1,098 (18.73%)	1,943 (21.08%)	3,501 (23.56%)	85,507 (35.34%)
Nonhispanic	133,077 (62.76%)	4,764 (81.27%)	7,276 (78.92%)	11,357 (76.44%)	156,474 (64.66%)
Female Head Of Hh	0.150 (0.072)	0.137 (0.075)	0.139 (0.077)	0.136 (0.071)	0.149 (0.072)
Missing	884 (0.417%)	438 (7.472%)	471 (5.109%)	420 (2.827%)	2,213 (0.915%)
Hh Public Assistance	0.039 (0.035)	0.034 (0.033)	0.036 (0.034)	0.035 (0.032)	0.039 (0.035)
Missing	884 (0.417%)	438 (7.472%)	471 (5.109%)	420 (2.827%)	2,213 (0.915%)
Household Income Less 30k	0.206 (0.119)	0.227 (0.133)	0.208 (0.125)	0.178 (0.114)	0.205 (0.119)
Missing	884 (0.417%)	438 (7.472%)	471 (5.109%)	420 (2.827%)	2,213 (0.915%)
Houspoverty	0.105 (0.089)	0.102 (0.095)	0.095 (0.088)	0.082 (0.078)	0.103 (0.089)
Missing	889 (0.419%)	438 (7.472%)	475 (5.152%)	420 (2.827%)	2,222 (0.918%)
Index Yr					
2014	25,969 (12.25%)	405 (6.91%)	218 (2.36%)	45 (0.3%)	26,637 (11.01%)
2015	25,837 (12.18%)	637 (10.87%)	328 (3.56%)	139 (0.94%)	26,941 (11.13%)
2016	28,046 (13.23%)	878 (14.98%)	420 (4.56%)	297 (2%)	29,641 (12.25%)
2017	28,229 (13.31%)	870 (14.84%)	673 (7.3%)	557 (3.75%)	30,329 (12.53%)
2018	25,515 (12.03%)	795 (13.56%)	1,235 (13.4%)	883 (5.94%)	28,428 (11.75%)
2019	25,932 (12.23%)	890 (15.18%)	1,369 (14.85%)	1,485 (9.99%)	29,676 (12.26%)
2020	23,651 (11.15%)	729 (12.44%)	1,871 (20.3%)	3,467 (23.33%)	29,718 (12.28%)
2021	28,863 (13.61%)	658 (11.22%)	3,105 (33.68%)	7,985 (53.74%)	40,611 (16.78%)
Bmi	32.81 (7.28)	32.98 (7.62)	38.61 (8.30)	34.35 (7.68)	33.13 (7.45)
Missing	5,904 (2.78%)	272 (4.64%)	264 (2.86%)	630 (4.24%)	7,070 (2.92%)
Smoking Status					
Formersmoker	57,569 (27.15%)	1,842 (31.42%)	2,979 (32.31%)	4,910 (33.05%)	67,300 (27.81%)
Currentsmoker	19,616 (9.25%)	553 (9.43%)	892 (9.68%)	1,229 (8.27%)	22,290 (9.21%)
Passivesmoker	1,158 (0.55%)	24 (0.41%)	51 (0.55%)	64 (0.43%)	1,297 (0.54%)
Neversmoker	131,261 (61.9%)	3,331 (56.82%)	5,148 (55.84%)	8,420 (56.67%)	148,160 (61.23%)
Unknown	2,438 (1.15%)	112 (1.91%)	149 (1.62%)	235 (1.58%)	2,934 (1.21%)
Low Educ	0.181 (0.134)	0.139 (0.112)	0.135 (0.111)	0.138 (0.110)	0.175 (0.132)
Missing	881 (0.415%)	438 (7.472%)	469 (5.087%)	420 (2.827%)	2,208 (0.912%)
Mgr Male	0.047 (0.045)	0.043 (0.042)	0.044 (0.043)	0.051 (0.051)	0.047 (0.045)
Missing	883 (0.416%)	438 (7.472%)	470 (5.098%)	420 (2.827%)	2,211 (0.914%)
Ndi	0.29 (0.17)	0.26 (0.16)	0.25 (0.15)	0.23 (0.14)	0.28 (0.17)
Missing	889 (0.42%)	438 (7.47%)	475 (5.15%)	420 (2.83%)	2,222 (0.92%)
Pct Crowding	0.093 (0.087)	0.057 (0.072)	0.065 (0.076)	0.075 (0.076)	0.090 (0.086)
Missing	884 (0.417%)	438 (7.472%)	471 (5.109%)	420 (2.827%)	2,213 (0.915%)
Racegrp					
White	103,428 (48.78%)	3,689 (62.93%)	5,609 (60.84%)	7,666 (51.6%)	120,392 (49.75%)
Asian	33,756 (15.92%)	735 (12.54%)	731 (7.93%)	2,690 (18.1%)	37,912 (15.67%)
Blackorafricanamerican	20,977 (9.89%)	669 (11.41%)	1,343 (14.57%)	1,483 (9.98%)	24,472 (10.11%)
Hawaiianorpacificislander	3,303 (1.56%)	61 (1.04%)	143 (1.55%)	371 (2.5%)	3,878 (1.6%)
Americanindianoralaskanative	1,505 (0.71%)	26 (0.44%)	48 (0.52%)	92 (0.62%)	1,671 (0.69%)
Multirace	5,740 (2.71%)	186 (3.17%)	523 (5.67%)	662 (4.46%)	7,111 (2.94%)
Other	149 (0.07%)	29 (0.49%)	57 (0.62%)	39 (0.26%)	274 (0.11%)
Unknown	43,184 (20.37%)	467 (7.97%)	765 (8.3%)	1,855 (12.48%)	46,271 (19.12%)

© 2025 Neugebauer R et al. *JAMA Network Open*.

	SU n = 212,042	DPP4i n = 5,862	GLP-1RA n = 9,219	SGLT2i n = 14,858	Total n = 241,981
Sex					
Female	95,560 (45.07%)	3,123 (53.28%)	5,319 (57.7%)	6,537 (44%)	110,539 (45.68%)
Male	116,476 (54.93%)	2,739 (46.72%)	3,900 (42.3%)	8,321 (56%)	131,436 (54.32%)
Other	4 (0%)	0 (0%)	0 (0%)	0 (0%)	4 (0%)
Unknown	2 (0%)	0 (0%)	0 (0%)	0 (0%)	2 (0%)
Site					
Kpnc	87,695 (41.36%)	672 (11.46%)	1,473 (15.98%)	5,400 (36.34%)	95,240 (39.36%)
Kpsc	110,811 (52.26%)	2,233 (38.09%)	3,075 (33.36%)	5,215 (35.1%)	121,334 (50.14%)
Kphi	5,289 (2.49%)	162 (2.76%)	760 (8.24%)	1,413 (9.51%)	7,624 (3.15%)
Hpi	2,567 (1.21%)	537 (9.16%)	1,350 (14.64%)	600 (4.04%)	5,054 (2.09%)
Hfhs	3,577 (1.69%)	919 (15.68%)	932 (10.11%)	618 (4.16%)	6,046 (2.5%)
Ghs	2,103 (0.99%)	1,339 (22.84%)	1,629 (17.67%)	1,612 (10.85%)	6,683 (2.76%)
Unemployment	0.050 (0.027)	0.045 (0.029)	0.040 (0.027)	0.038 (0.023)	0.049 (0.027)
Missing	878 (0.414%)	438 (7.472%)	469 (5.087%)	420 (2.827%)	2,205 (0.911%)
**Insurance**					
Ins Commercial	165,908 (78.24%)	3,190 (54.42%)	5,697 (61.8%)	8,665 (58.32%)	183,460 (75.82%)
Ins Highdeductible	19,445 (9.17%)	270 (4.61%)	590 (6.4%)	719 (4.84%)	21,024 (8.69%)
Ins Medicaid	12,436 (5.86%)	660 (11.26%)	1,387 (15.05%)	1,642 (11.05%)	16,125 (6.66%)
Ins Medicare	58,294 (27.49%)	2,420 (41.28%)	2,610 (28.31%)	5,715 (38.46%)	69,039 (28.53%)
Ins Medicare A	34,314 (16.18%)	1,600 (27.29%)	1,860 (20.18%)	4,030 (27.12%)	41,804 (17.28%)
Ins Medicare B	32,115 (15.15%)	1,513 (25.81%)	1,751 (18.99%)	3,849 (25.91%)	39,228 (16.21%)
Ins Medicare C	27,484 (12.96%)	1,393 (23.76%)	1,578 (17.12%)	3,441 (23.16%)	33,896 (14.01%)
Ins Medicare D	29,926 (14.11%)	1,365 (23.29%)	1,641 (17.8%)	3,727 (25.08%)	36,659 (15.15%)
Ins Other Coverage	47,024 (22.18%)	1,225 (20.9%)	1,922 (20.85%)	3,017 (20.31%)	53,188 (21.98%)
Ins Privatepay	39,506 (18.63%)	809 (13.8%)	941 (10.21%)	2,181 (14.68%)	43,437 (17.95%)
Ins Selffunded	3,791 (1.79%)	263 (4.49%)	737 (7.99%)	442 (2.97%)	5,233 (2.16%)
Ins Statesubsidized	607 (0.29%)	310 (5.29%)	597 (6.48%)	483 (3.25%)	1,997 (0.83%)
**Clinical data**					
A1c Age	41.94 (77.88)	56.39 (102.69)	57.93 (90.75)	47.91 (77.25)	43.27 (79.16)
Missing	216 (0.1%)	14 (0.24%)	16 (0.17%)	13 (0.09%)	259 (0.11%)
Acc Aha 201310yrcvdrisk	0.167 (0.150)	0.212 (0.170)	0.153 (0.137)	0.203 (0.158)	0.170 (0.151)
Missing	25,193 (11.881%)	690 (11.771%)	1,059 (11.487%)	2,009 (13.521%)	28,951 (11.964%)
Chf Dx Status					
0	203,529 (95.99%)	5,378 (91.74%)	8,431 (91.45%)	12,339 (83.05%)	229,677 (94.92%)
1	7,705 (3.63%)	429 (7.32%)	716 (7.77%)	2,342 (15.76%)	11,192 (4.63%)
999	808 (0.38%)	55 (0.94%)	72 (0.78%)	177 (1.19%)	1,112 (0.46%)
Cv Risk Subgrp					
Low	82,644 (38.98%)	1,726 (29.44%)	3,696 (40.09%)	4,140 (27.86%)	92,206 (38.1%)
Moderate	43,301 (20.42%)	1,131 (19.29%)	1,967 (21.34%)	2,927 (19.7%)	49,326 (20.38%)
High	56,836 (26.8%)	2,089 (35.64%)	2,194 (23.8%)	4,928 (33.17%)	66,047 (27.29%)
Other	4,525 (2.13%)	248 (4.23%)	325 (3.53%)	935 (6.29%)	6,033 (2.49%)
Unknown	24,736 (11.67%)	668 (11.4%)	1,037 (11.25%)	1,928 (12.98%)	28,369 (11.72%)
Diab Duration	4.87 (3.21)	5.93 (3.28)	6.99 (3.72)	7.64 (3.93)	5.14 (3.37)
Missing	351 (0.17%)	33 (0.56%)	8 (0.09%)	0 (0%)	392 (0.16%)
A1c	9.25 (2.08)	8.24 (1.65)	8.79 (1.91)	8.60 (1.73)	9.17 (2.06)
Missing	216 (0.1%)	14 (0.24%)	16 (0.17%)	13 (0.09%)	259 (0.11%)
Acr	45.90 (82.67)	50.75 (91.95)	61.81 (102.12)	83.57 (118.03)	48.94 (86.79)
Missing	41,920 (19.77%)	1,341 (22.88%)	2,400 (26.03%)	2,708 (18.23%)	48,369 (19.99%)
Afib Dx	8,265 (3.9%)	467 (7.97%)	492 (5.34%)	1,474 (9.92%)	10,698 (4.42%)
Alt	33.35 (22.25)	30.60 (19.02)	31.76 (19.32)	29.74 (18.17)	33.01 (21.87)
Missing	44,616 (21.04%)	1,078 (18.39%)	1,932 (20.96%)	3,463 (23.31%)	51,089 (21.11%)
Amputation Dxpx	1,154 (0.54%)	68 (1.16%)	202 (2.19%)	265 (1.78%)	1,689 (0.7%)
Anemia Dx	12,191 (5.75%)	699 (11.92%)	1,046 (11.35%)	1,782 (11.99%)	15,718 (6.5%)
Anxiety Dx	36,121 (17.03%)	1,350 (23.03%)	2,769 (30.04%)	3,258 (21.93%)	43,498 (17.98%)
Arrythmia Dx	7,267 (3.43%)	473 (8.07%)	590 (6.4%)	1,374 (9.25%)	9,704 (4.01%)
Ascvd Dxpx Max	19,370 (9.13%)	1,013 (17.28%)	1,313 (14.24%)	3,021 (20.33%)	24,717 (10.21%)
Ascvd Dxpx Ppv	4,525 (2.13%)	248 (4.23%)	325 (3.53%)	935 (6.29%)	6,033 (2.49%)

© 2025 Neugebauer R et al. *JAMA Network Open*.

	SU n = 212,042	DPP4i n = 5,862	GLP-1RA n = 9,219	SGLT2i n = 14,858	Total n = 241,981
Asthma Dx	21,267 (10.03%)	656 (11.19%)	1,570 (17.03%)	1,996 (13.43%)	25,489 (10.53%)
Bariatric Px	2,057 (0.97%)	108 (1.84%)	317 (3.44%)	212 (1.43%)	2,694 (1.11%)
Bipolar Dx	1,864 (0.88%)	79 (1.35%)	214 (2.32%)	175 (1.18%)	2,332 (0.96%)
Blind Dx	722 (0.34%)	23 (0.39%)	28 (0.3%)	40 (0.27%)	813 (0.34%)
Cad Dxpx Max	12,017 (5.67%)	605 (10.32%)	808 (8.76%)	2,122 (14.28%)	15,552 (6.43%)
Cad Dxpx Ppv	2,742 (1.29%)	160 (2.73%)	233 (2.53%)	727 (4.89%)	3,862 (1.6%)
Cad Dxpx Sens	3,087 (1.46%)	185 (3.16%)	259 (2.81%)	792 (5.33%)	4,323 (1.79%)
Cancer Mets Dx	0 (0%)	0 (0%)	0 (0%)	0 (0%)	0 (0%)
Cancer Nomets Dx	7,742 (3.65%)	377 (6.43%)	638 (6.92%)	834 (5.61%)	9,591 (3.96%)
Cevd Dxpx Ppv	1,749 (0.82%)	72 (1.23%)	71 (0.77%)	183 (1.23%)	2,075 (0.86%)
Cevd Dxpx Sens	6,398 (3.02%)	345 (5.89%)	428 (4.64%)	893 (6.01%)	8,064 (3.33%)
Chf Dx Ppv	1,735 (0.82%)	95 (1.62%)	153 (1.66%)	629 (4.23%)	2,612 (1.08%)
Chf Dx Sens	7,705 (3.63%)	429 (7.32%)	716 (7.77%)	2,342 (15.76%)	11,192 (4.63%)
Ckd Dx	41,668 (19.65%)	1,612 (27.5%)	2,702 (29.31%)	5,698 (38.35%)	51,680 (21.36%)
Copd Dx	7,354 (3.47%)	411 (7.01%)	575 (6.24%)	983 (6.62%)	9,323 (3.85%)
Coupled Dbp	75.03 (10.72)	73.06 (10.99)	74.24 (11.23)	72.69 (11.71)	74.81 (10.83)
Missing	3,769 (1.78%)	252 (4.3%)	182 (1.97%)	327 (2.2%)	4,530 (1.87%)
Coupled Sbp	129.48 (14.47)	129.15 (15.16)	130.55 (14.63)	130.51 (15.70)	129.57 (14.57)
Missing	3,769 (1.78%)	252 (4.3%)	182 (1.97%)	327 (2.2%)	4,530 (1.87%)
Covid Prd	52,514 (24.77%)	1,387 (23.66%)	4,976 (53.98%)	11,452 (77.08%)	70,329 (29.06%)
Creat	0.88 (0.26)	0.94 (0.32)	0.91 (0.29)	0.98 (0.31)	0.89 (0.27)
Missing	5,782 (2.73%)	212 (3.62%)	242 (2.63%)	249 (1.68%)	6,485 (2.68%)
Cysticfibrosis Dx	0 (0%)	0 (0%)	0 (0%)	0 (0%)	0 (0%)
Dbp	74.19 (10.71)	72.45 (11.01)	73.61 (11.34)	71.74 (11.84)	73.97 (10.83)
Missing	3,766 (1.78%)	252 (4.3%)	182 (1.97%)	327 (2.2%)	4,527 (1.87%)
Dementia Dx	0 (0%)	0 (0%)	0 (0%)	0 (0%)	0 (0%)
Depr Dx	27,309 (12.88%)	1,133 (19.33%)	2,435 (26.41%)	2,316 (15.59%)	33,193 (13.72%)
Dietitian	9,557 (4.51%)	399 (6.81%)	1,463 (15.87%)	1,170 (7.87%)	12,589 (5.2%)
Dka Dx	2,808 (1.32%)	85 (1.45%)	262 (2.84%)	203 (1.37%)	3,358 (1.39%)
Dka Dx Count	0.01 (0.13)	0.02 (0.14)	0.04 (0.28)	0.02 (0.16)	0.02 (0.14)
Esrd Dx	8,139 (3.84%)	484 (8.26%)	820 (8.89%)	1,830 (12.32%)	11,273 (4.66%)
Esrd Px	408 (0.19%)	40 (0.68%)	45 (0.49%)	46 (0.31%)	539 (0.22%)
Etoh Dx	3,488 (1.64%)	102 (1.74%)	174 (1.89%)	315 (2.12%)	4,079 (1.69%)
Fasciitis Dx	0 (0%)	0 (0%)	0 (0%)	0 (0%)	0 (0%)
Fpg	181.45 (73.90)	159.77 (61.24)	173.96 (76.35)	166.40 (69.11)	180.45 (73.72)
Missing	139,168 (65.63%)	4,612 (78.68%)	7,423 (80.52%)	12,323 (82.94%)	163,526 (67.58%)
Frailty Dx	13,060 (6.16%)	859 (14.65%)	1,387 (15.05%)	1,680 (11.31%)	16,986 (7.02%)
Gfr Epi 09	88.95 (21.62)	80.84 (24.15)	85.95 (23.81)	79.79 (22.71)	88.08 (21.99)
Missing	5,782 (2.73%)	212 (3.62%)	242 (2.63%)	249 (1.68%)	6,485 (2.68%)
Hdl	44.55 (11.42)	44.79 (12.11)	43.52 (11.74)	43.67 (11.69)	44.47 (11.47)
Missing	29,836 (14.07%)	701 (11.96%)	1,186 (12.86%)	2,103 (14.15%)	33,826 (13.98%)
Hgb	14.05 (1.62)	13.53 (1.59)	13.59 (1.55)	13.70 (1.62)	13.99 (1.62)
Missing	59,461 (28.04%)	1,314 (22.42%)	2,244 (24.34%)	3,649 (24.56%)	66,668 (27.55%)
Htn Dx	114,520 (54.01%)	4,051 (69.11%)	6,421 (69.65%)	10,673 (71.83%)	135,665 (56.06%)
Hypo Dx	151 (0.07%)	16 (0.27%)	33 (0.36%)	56 (0.38%)	256 (0.11%)
Hypo Dx Count	0.00 (0.09)	0.02 (0.19)	0.03 (0.21)	0.03 (0.27)	0.01 (0.12)
Hypo Dx Event	151 (0.07%)	16 (0.27%)	33 (0.36%)	56 (0.38%)	256 (0.11%)
Hypothyroidism Dx	19,887 (9.38%)	979 (16.7%)	1,450 (15.73%)	1,962 (13.21%)	24,278 (10.03%)
Ldl	96.62 (39.09)	89.76 (35.92)	91.16 (37.28)	85.02 (36.99)	95.54 (38.94)
Missing	23,654 (11.16%)	533 (9.09%)	953 (10.34%)	1,833 (12.34%)	26,973 (11.15%)
Leukemia Lymphoma Dx	1,208 (0.57%)	59 (1.01%)	48 (0.52%)	111 (0.75%)	1,426 (0.59%)
Lipid Dx	124,806 (58.86%)	4,123 (70.33%)	6,265 (67.96%)	10,579 (71.2%)	145,773 (60.24%)
Liver Dx	302 (0.14%)	25 (0.43%)	30 (0.33%)	24 (0.16%)	381 (0.16%)
Mci Dx	948 (0.45%)	53 (0.9%)	57 (0.62%)	83 (0.56%)	1,141 (0.47%)
Men2 Dx	0 (0%)	0 (0%)	0 (0%)	0 (0%)	0 (0%)
Nephropathy Dx	11,883 (5.6%)	404 (6.89%)	431 (4.68%)	456 (3.07%)	13,174 (5.44%)
Neuro Dx	4,736 (2.23%)	248 (4.23%)	419 (4.54%)	517 (3.48%)	5,920 (2.45%)

© 2025 Neugebauer R et al. *JAMA Network Open*.

	SU<br>n = 212,042	DPP4i<br>n = 5,862	GLP-1RA<br>n = 9,219	SGLT2i<br>n = 14,858	Total<br>n = 241,981
Pancreatitis Dx	0 (0%)	0 (0%)	0 (0%)	0 (0%)	0 (0%)
Pcr	0.000 (0.000)	0.000 (0.000)	0.000 (0.000)	0.000 (0.000)	0.000 (0.000)
Missing	198,970 (93.835%)	5,544 (94.575%)	8,582 (93.09%)	12,570 (84.601%)	225,666 (93.258%)
Potassium	4.23 (0.40)	4.28 (0.43)	4.24 (0.42)	4.28 (0.42)	4.24 (0.40)
Missing	17,166 (8.1%)	327 (5.58%)	446 (4.84%)	580 (3.9%)	18,519 (7.65%)
Pregnancy	0 (0%)	0 (0%)	0 (0%)	0 (0%)	0 (0%)
Pud Dx	197 (0.09%)	7 (0.12%)	11 (0.12%)	24 (0.16%)	239 (0.1%)
Pvd Dxpx Ppv	375 (0.18%)	31 (0.53%)	41 (0.44%)	64 (0.43%)	511 (0.21%)
Pvd Dxpx Sens	3,557 (1.68%)	254 (4.33%)	323 (3.5%)	593 (3.99%)	4,727 (1.95%)
Pyelo Dx	0 (0%)	0 (0%)	0 (0%)	0 (0%)	0 (0%)
Retinopathy Dxpx	6,428 (3.03%)	347 (5.92%)	555 (6.02%)	712 (4.79%)	8,042 (3.32%)
Rpg	216.03 (113.91)	180.96 (84.26)	196.24 (96.29)	189.83 (88.79)	211.41 (110.57)
Missing	137,757 (64.97%)	2,338 (39.88%)	3,945 (42.79%)	7,626 (51.33%)	151,666 (62.68%)
Sbp	128.69 (14.29)	128.54 (15.06)	129.89 (14.49)	129.60 (15.74)	128.79 (14.42)
Missing	3,769 (1.78%)	252 (4.3%)	182 (1.97%)	327 (2.2%)	4,530 (1.87%)
Schiz Dx	1,261 (0.59%)	46 (0.78%)	70 (0.76%)	81 (0.55%)	1,458 (0.6%)
Sodium	138.04 (3.05)	138.73 (2.96)	138.70 (2.93)	138.90 (2.97)	138.15 (3.05)
Missing	48,886 (23.05%)	578 (9.86%)	1,037 (11.25%)	2,444 (16.45%)	52,945 (21.88%)
Sud Dx	1,964 (0.93%)	72 (1.23%)	158 (1.71%)	174 (1.17%)	2,368 (0.98%)
Tc	177.31 (52.06)	167.25 (46.35)	167.68 (46.60)	162.87 (47.58)	175.81 (51.62)
Missing	28,991 (13.67%)	697 (11.89%)	1,176 (12.76%)	2,091 (14.07%)	32,955 (13.62%)
Trig	214.82 (211.51)	184.61 (149.02)	195.14 (159.81)	197.20 (160.03)	212.26 (205.81)
Missing	40,333 (19.02%)	905 (15.44%)	1,919 (20.82%)	3,171 (21.34%)	46,328 (19.15%)
Tsh	2.04 (1.90)	2.20 (2.26)	2.16 (2.22)	2.18 (1.98)	2.06 (1.93)
Missing	76,848 (36.24%)	2,077 (35.43%)	3,185 (34.55%)	5,643 (37.98%)	87,753 (36.26%)
Valvular Dx	3,317 (1.56%)	265 (4.52%)	257 (2.79%)	730 (4.91%)	4,569 (1.89%)
Vasculitis Dx	2,527 (1.19%)	110 (1.88%)	120 (1.3%)	154 (1.04%)	2,911 (1.2%)
Only Met No Ascvd	121,634 (57.36%)	2,455 (41.88%)	2,264 (24.56%)	5,056 (34.03%)	131,409 (54.31%)
Renal Function Status					
Lowrisk	110,256 (52%)	2,661 (45.39%)	3,870 (41.98%)	5,844 (39.33%)	122,631 (50.68%)
Moderaterisk	41,064 (19.37%)	1,097 (18.71%)	1,879 (20.38%)	3,471 (23.36%)	47,511 (19.63%)
Highrisk	13,425 (6.33%)	508 (8.67%)	731 (7.93%)	1,877 (12.63%)	16,541 (6.84%)
Veryhighrisk	4,150 (1.96%)	236 (4.03%)	287 (3.11%)	927 (6.24%)	5,600 (2.31%)
Unknown	43,147 (20.35%)	1,360 (23.2%)	2,452 (26.6%)	2,739 (18.43%)	49,698 (20.54%)
Total Visit C	0.42 (1.73)	1.07 (3.41)	1.15 (3.62)	1.40 (3.78)	0.53 (2.09)
Total Visit E	0.13 (1.07)	0.66 (2.78)	1.16 (3.22)	0.56 (2.44)	0.21 (1.42)
Total Visit N	0.10 (1.78)	0.35 (3.17)	0.30 (2.12)	0.39 (1.89)	0.13 (1.85)
**Concurrent medications**					
Aa	2 (0%)	0 (0%)	5 (0.05%)	0 (0%)	7 (0%)
Aceinhibitors	73,936 (34.87%)	2,189 (37.34%)	3,505 (38.02%)	5,878 (39.56%)	85,508 (35.34%)
Agi	211 (0.1%)	44 (0.75%)	25 (0.27%)	48 (0.32%)	328 (0.14%)
Anticoagulants	6,777 (3.2%)	397 (6.77%)	503 (5.46%)	1,344 (9.05%)	9,021 (3.73%)
Anticonvulsants	11,978 (5.65%)	656 (11.19%)	1,684 (18.27%)	1,942 (13.07%)	16,260 (6.72%)
Antidepressantcomb	3 (0%)	0 (0%)	1 (0.01%)	0 (0%)	4 (0%)
Antidepressantmaoi	23 (0.01%)	2 (0.03%)	2 (0.02%)	2 (0.01%)	29 (0.01%)
Antidepressantndri	3,592 (1.69%)	177 (3.02%)	500 (5.42%)	393 (2.65%)	4,662 (1.93%)
Antidepressantother	0 (0%)	0 (0%)	0 (0%)	0 (0%)	0 (0%)
Antidepressantsari	5,095 (2.4%)	200 (3.41%)	471 (5.11%)	527 (3.55%)	6,293 (2.6%)
Antidepressantsnri	5,512 (2.6%)	310 (5.29%)	829 (8.99%)	843 (5.67%)	7,494 (3.1%)
Antidepressantspo	57 (0.03%)	10 (0.17%)	16 (0.17%)	18 (0.12%)	101 (0.04%)
Antidepressantssri	16,944 (7.99%)	726 (12.38%)	1,528 (16.57%)	1,651 (11.11%)	20,849 (8.62%)
Antidepressanttca	5,552 (2.62%)	170 (2.9%)	414 (4.49%)	415 (2.79%)	6,551 (2.71%)
Antidepressantteca	1,168 (0.55%)	61 (1.04%)	97 (1.05%)	119 (0.8%)	1,445 (0.6%)
Antiplatelets	11,469 (5.41%)	560 (9.55%)	996 (10.8%)	2,117 (14.25%)	15,142 (6.26%)
Antipsychotic1stgen	440 (0.21%)	28 (0.48%)	24 (0.26%)	28 (0.19%)	520 (0.21%)
Antipsychotic2ndgen	3,051 (1.44%)	154 (2.63%)	314 (3.41%)	263 (1.77%)	3,782 (1.56%)
Anxiety	2,252 (1.06%)	120 (2.05%)	327 (3.55%)	285 (1.92%)	2,984 (1.23%)

© 2025 Neugebauer R et al. *JAMA Network Open*.

	SU n = 212,042	DPP4i n = 5,862	GLP-1RA n = 9,219	SGLT2i n = 14,858	Total n = 241,981
Arb	34,480 (16.26%)	1,368 (23.34%)	2,378 (25.79%)	4,587 (30.87%)	42,813 (17.69%)
Benzodiazepines	7,548 (3.56%)	360 (6.14%)	554 (6.01%)	536 (3.61%)	8,998 (3.72%)
Betablockers	44,561 (21.02%)	1,894 (32.31%)	2,927 (31.75%)	5,948 (40.03%)	55,330 (22.87%)
Clonidine	1,736 (0.82%)	91 (1.55%)	109 (1.18%)	190 (1.28%)	2,126 (0.88%)
Dihydropyridineccb	27,018 (12.74%)	1,088 (18.56%)	1,654 (17.94%)	3,131 (21.07%)	32,891 (13.59%)
Hypnoticother	1,543 (0.73%)	80 (1.36%)	145 (1.57%)	120 (0.81%)	1,888 (0.78%)
Injectableantipsychotic	9 (0%)	3 (0.05%)	7 (0.08%)	2 (0.01%)	21 (0.01%)
Ins	15,199 (7.17%)	1,518 (25.9%)	5,611 (60.86%)	6,503 (43.77%)	28,831 (11.91%)
Ins Analog	2,969 (1.4%)	755 (12.88%)	2,678 (29.05%)	2,095 (14.1%)	8,497 (3.51%)
Ins Combo	1,386 (0.65%)	191 (3.26%)	691 (7.5%)	1,187 (7.99%)	3,455 (1.43%)
Ins Human	12,965 (6.11%)	890 (15.18%)	3,451 (37.43%)	5,123 (34.48%)	22,429 (9.27%)
Ins La	13,238 (6.24%)	1,248 (21.29%)	4,311 (46.76%)	4,835 (32.54%)	23,632 (9.77%)
Ins Sa	3,889 (1.83%)	739 (12.61%)	3,352 (36.36%)	3,622 (24.38%)	11,602 (4.79%)
Ksparingdiuretics	5,322 (2.51%)	289 (4.93%)	571 (6.19%)	1,217 (8.19%)	7,399 (3.06%)
Lithium	317 (0.15%)	14 (0.24%)	28 (0.3%)	25 (0.17%)	384 (0.16%)
Loopdiuretics	8,960 (4.23%)	626 (10.68%)	1,109 (12.03%)	2,268 (15.26%)	12,963 (5.36%)
Meg	100 (0.05%)	62 (1.06%)	47 (0.51%)	46 (0.31%)	255 (0.11%)
Met	144,034 (67.93%)	3,913 (66.75%)	6,237 (67.65%)	10,470 (70.47%)	164,654 (68.04%)
Nondihydropyridineccb	3,295 (1.55%)	168 (2.87%)	215 (2.33%)	359 (2.42%)	4,037 (1.67%)
Otherlipidmeds	5,448 (2.57%)	262 (4.47%)	435 (4.72%)	639 (4.3%)	6,784 (2.8%)
Pcsk9mab	22 (0.01%)	2 (0.03%)	12 (0.13%)	21 (0.14%)	57 (0.02%)
Statins	118,477 (55.87%)	3,756 (64.07%)	6,335 (68.72%)	11,381 (76.6%)	139,949 (57.83%)
Stimulants	826 (0.39%)	36 (0.61%)	100 (1.08%)	66 (0.44%)	1,028 (0.42%)
Suold	1,452 (0.68%)	7 (0.12%)	2 (0.02%)	0 (0%)	1,461 (0.6%)
Thiazidediuretics	48,777 (23%)	1,443 (24.62%)	2,454 (26.62%)	3,631 (24.44%)	56,305 (23.27%)
Tir	0 (0%)	0 (0%)	0 (0%)	0 (0%)	0 (0%)
Tzd	1,731 (0.82%)	177 (3.02%)	159 (1.72%)	327 (2.2%)	2,394 (0.99%)
Only Met Therapy	132,066 (62.28%)	2,849 (48.6%)	2,485 (26.96%)	5,992 (40.33%)	143,392 (59.26%)

© 2025 Neugebauer R et al. *JAMA Network Open*.

eFigure 2. MACE (Primary Definition), 4-Arm Drug Class Comparison, Sulfonylureas vs DPP4is vs SGLT2is vs GLP1-RAs, Cumulative Incidence Curves From PP Analyses With IPW, TMLE, and SL
Each plot emulates inferences from Per-Protocol (PP) analyses for a 4-arm RCT comparing SU, DPP4i, SGLT2i, and GLP-1RA and represents unadjusted or adjusted estimates of cumulative incidence curves for MACE derived with inverse probability weighting (IPW) and Targeted Minimum Loss-based Estimation (TMLE) with Super Learning (SL) estimates of propensity scores with four weight truncation schemes: IPW and TMLE without weight truncation (untruncated), IPW with truncation of stabilized weights at value 20 (trunc20) or at the 99th percentile of weight values (trunc99), and TMLE with truncation of unstabilized weights at value 200 (trunc200). Each table displays p values for the test that the average risk difference (ARD) through 2.5 years of follow-up (30 months) between any two arms is 0.

PP

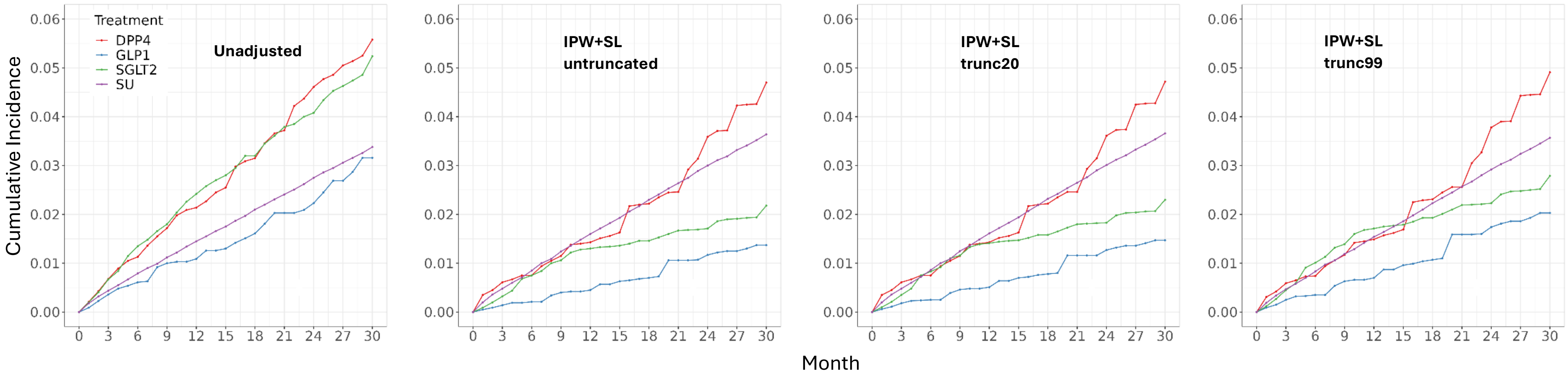

		ARD p value
DPP4	SU	<0.00005
SGLT2	SU	<0.00005
GLP1	SU	0.0527
SGLT2	DPP4	0.8614
GLP1	DPP4	<0.00005
GLP1	SGLT2	<0.00005

		ARD p value
DPP4	SU	0.6014
SGLT2	SU	<0.00005
GLP1	SU	<0.00005
SGLT2	DPP4	0.0127
GLP1	DPP4	<0.00005
GLP1	SGLT2	0.0055

		ARD p value
DPP4	SU	0.6107
SGLT2	SU	0.0007
GLP1	SU	<0.00005
SGLT2	DPP4	0.0295
GLP1	DPP4	<0.00005
GLP1	SGLT2	0.0056

		ARD p value
DPP4	SU	0.3165
SGLT2	SU	0.2523
GLP1	SU	0.0001
SGLT2	DPP4	0.1414
GLP1	DPP4	0.0013
GLP1	SGLT2	0.0196

		ARD p value
DPP4	SU	0.4715
SGLT2	SU	<0.00005
GLP1	SU	<0.00005
SGLT2	DPP4	<0.00005
GLP1	DPP4	<0.00005
GLP1	SGLT2	<0.00005

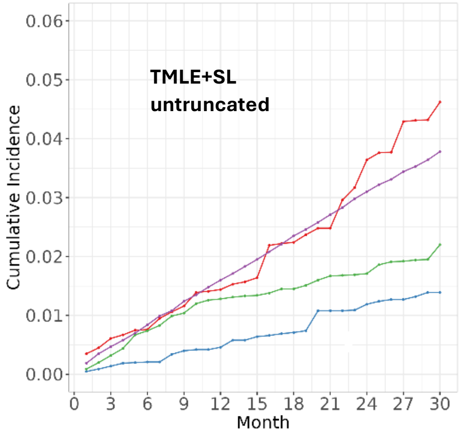

		ARD p value
DPP4	SU	0.0698
SGLT2	SU	0.0001
GLP1	SU	<0.00005
SGLT2	DPP4	0.0002
GLP1	DPP4	<0.00005
GLP1	SGLT2	<0.00005

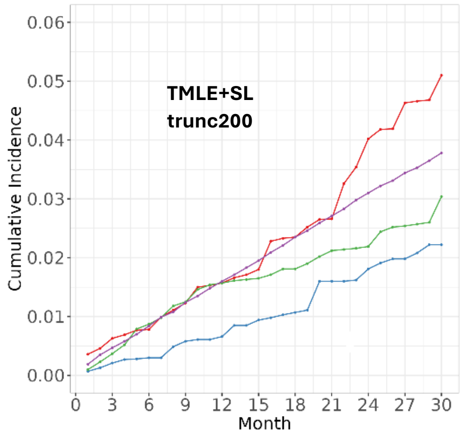

© 2025 Neugebauer R et al. *JAMA Network Open*.

eFigure 3. MACE (Primary Definition), 4-Arm Drug Class Comparison, Sulfonylureas vs DPP4is vs SGLT2is vs GLP1-RAs, Cumulative Incidence Curves From ITT Analyses With IPW, TMLE, and SL
Each plot emulates inferences from Intention-To-Treat (ITT) analyses for a 4-arm RCT comparing SU, DPP4i, SGLT2i, and GLP-1RA and represents unadjusted or adjusted estimates of cumulative incidence curves for MACE derived with inverse probability weighting (IPW) and Targeted Minimum Loss-based Estimation (TMLE) with Super Learning (SL) estimates of propensity scores with four weight truncation schemes: IPW and TMLE without weight truncation (untruncated), IPW with truncation of stabilized weights at value 20 (trunc20) or at the 99th percentile of weight values (trunc99), and TMLE with truncation of unstabilized weights at value 200 (trunc200). Each table displays p values for the test that the average risk difference (ARD) through 2.5 years of follow-up (30 months) between any two arms is 0.

ITT

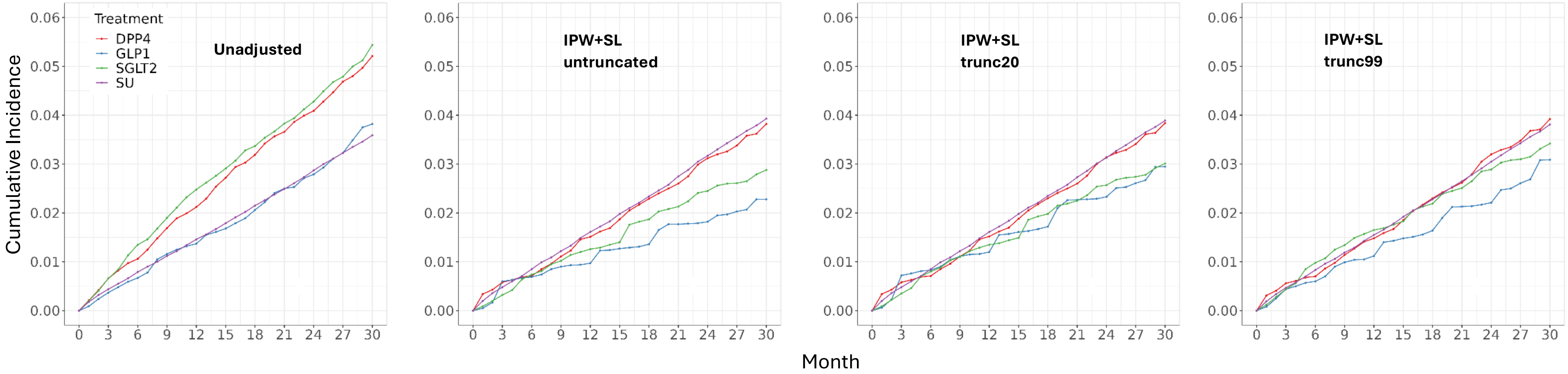

		ARD p value
DPP4	SU	<0.00005
SGLT2	SU	<0.00005
GLP1	SU	0.8564
SGLT2	DPP4	0.4525
GLP1	DPP4	<0.00005
GLP1	SGLT2	<0.00005

		ARD p value
DPP4	SU	0.7689
SGLT2	SU	0.0304
GLP1	SU	0.0671
SGLT2	DPP4	0.2318
GLP1	DPP4	0.1563
GLP1	SGLT2	0.5293

		ARD p value
DPP4	SU	0.8225
SGLT2	SU	0.0977
GLP1	SU	0.3867
SGLT2	DPP4	0.354
GLP1	DPP4	0.5137
GLP1	SGLT2	0.9463

		ARD p value
DPP4	SU	0.9596
SGLT2	SU	0.7764
GLP1	SU	0.0681
SGLT2	DPP4	0.8209
GLP1	DPP4	0.1642
GLP1	SGLT2	0.2058

		ARD p value
DPP4	SU	0.6118
SGLT2	SU	<0.00005
GLP1	SU	0.0052
SGLT2	DPP4	0.048
GLP1	DPP4	0.0362
GLP1	SGLT2	0.3575

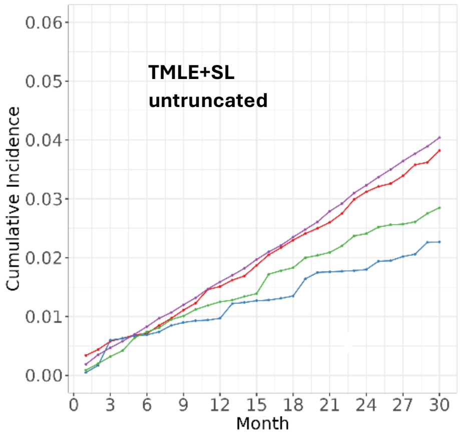

		ARD p value
DPP4	SU	0.7154
SGLT2	SU	0.001
GLP1	SU	0.0001
SGLT2	DPP4	0.1722
GLP1	DPP4	0.0281
GLP1	SGLT2	0.1998

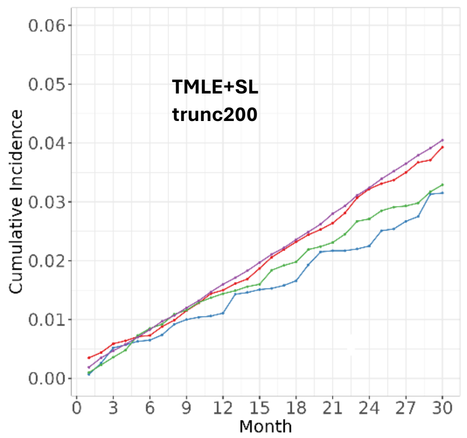

© 2025 Neugebauer R et al. *JAMA Network Open*.

eTable 3. MACE (Primary Definition), 4-Arm Drug Class Comparison, Sulfonylureas vs DPP4is vs SGLT2is vs GLP1-RAs, RD and HR Effect Measures for Comparing Sulfonylureas to DPP4is at 2.5 Years
Estimation results from ITT and PP analyses to compare MACE risks over 2.5 years between SU and DPP4i initiators based on an emulated 4-arm RCT for comparing SU, DPP4i, SGLT2i, and GLP-1RA. For PP analyses, rates of protocol deviations are described by medication class initiated at baseline. Unadjusted point and interval estimates and adjusted point and interval IPW and TMLE estimates of risks, risk differences (RD), and hazard ratios (HR) based on propensity scores (PS) estimated with either logistic models or super learning (SL) are presented for four weight truncation schemes along with the corresponding 99th percentile and maximum value of the stabilized and unstabilized inverse probability weights used for implementing IPW and TMLE, respectively. RD is the risk in treatment arm minus the risk in control arm and NNT is the number needed to treat.

Analysis type	Protocol Deviations* by exposure group (%)	PS estimation	99th IP weights	Max IP weight	Estimator	Treatment (DPP4i) risk in %	Control (SU) risk in %	RD [95% CI] in %	NNT	HR [95% CI]
PP	<u>Discontinuation</u><br>SU: 48.35<br>DPP4i: 44.08<br><br><u>Crossover</u><br>SU: 7.09<br>DPP4i: 28.31	SL	[redacted]	[redacted]	Unadjusted	5.58	3.38	2.20 [1.13, 3.26]	46	1.49 [1.17, 1.81]
			56.76	12,029.13	TMLE untruncated	4.62	3.78	0.84 [0.08, 1.60]	119	[redacted]
					TMLE truncated at 200	5.10	3.78	1.32 [0.69, 1.94]	76	
			3.16	667.50	IPW untruncated	4.70	3.64	1.06 [-0.47, 2.58]		0.95 [0.58, 1.32]
					IPW truncated at 20	4.72	3.66	1.06 [-0.47, 2.59]		0.95 [0.58, 1.32]
					IPW truncated at 99th	4.91	3.57	1.34 [-0.24, 2.91]		1.00 [0.65, 1.36]
		Logistic model	4.22	2.54e+15	IPW untruncated	5.04	2.84	2.20 [0.34, 4.06]	45	0.93 [0.49, 1.37]
					IPW truncated at 20	5.08	3.98	1.10 [-0.76, 2.97]		0.98 [0.52, 1.44]
					IPW truncated at 99th	5.40	3.73	1.67 [-0.27, 3.61]		1.05 [0.65, 1.46]
ITT	[redacted]	SL	[redacted]	[redacted]	Unadjusted	5.21	3.59	1.62 [0.96, 2.28]	62	1.46 [1.18, 1.73]
			51.30	15,530.99	TMLE untruncated	3.82	4.04	-0.22 [-0.82, 0.39]		[redacted]
					TMLE truncated at 200	3.93	4.05	-0.13 [-0.67, 0.42]		
			2.99	1,619.89	IPW untruncated	3.82	3.93	-0.11 [-1.02, 0.80]		0.94 [0.59, 1.30]
					IPW truncated at 20	3.84	3.89	-0.05 [-0.96, 0.86]		0.95 [0.59, 1.30]
					IPW truncated at 99th	3.92	3.81	0.11 [-0.75, 0.98]		0.96 [0.65, 1.26]
		Logistic model	3.79	6,946.90	IPW untruncated	3.68	4.09	-0.41 [-1.37, 0.55]		0.87 [0.50, 1.25]
					IPW truncated at 20	3.76	4.01	-0.25 [-1.21, 0.70]		0.91 [0.53, 1.30]
					IPW truncated at 99th	3.94	3.86	0.08 [-0.84, 1.00]		0.95 [0.63, 1.27]

* Discontinuation refers to the interruption of the comparator medication initiated on index date; Crossover refers to the initiation of the comparator medication initiated by patient at baseline in the other arm.

© 2025 Neugebauer R et al. *JAMA Network Open*.

eTable 4. MACE (Primary Definition), 4-Arm Drug Class Comparison, Sulfonylureas vs DPP4is vs SGLT2is vs GLP1-RAs, RD and HR Effect Measures for Comparing Sulfonylureas to SGLT2is at 2.5 Years

Estimation results from ITT and PP analyses to compare MACE risks over 2.5 years between SU and SGLT2i initiators based on an emulated 4-arm RCT for comparing SU, DPP4i, SGLT2i, and GLP-1RA. For PP analyses, rates of protocol deviations are described by medication class initiated at baseline. Unadjusted point and interval estimates and adjusted point and interval IPW and TMLE estimates of risks, risk differences (RD), and hazard ratios (HR) based on propensity scores (PS) estimated with either logistic models or super learning (SL) are presented for four weight truncation schemes along with the corresponding 99th percentile and maximum value of the stabilized and unstabilized inverse probability weights used for implementing IPW and TMLE, respectively. RD is the risk in treatment arm minus the risk in control arm and NNT is the number needed to treat.

Analysis type	Protocol Deviations* by exposure group (%)	PS estimation	99th IP weights	Max IP weight	Estimator	Treatment (SU) risk in %	Control (SGLT2i) risk in %	RD [95% CI] in %	NNT	HR [95% CI]
PP	<u>Discontinuation</u><br>SU: 48.35<br>SGLT2i: 21.87<br><br><u>Crossover</u><br>SU: 7.09<br>SGLT2i: 12.08	SL			Unadjusted	3.38	5.24	-1.86 [-2.82, -0.91]	54	0.61 [0.52, 0.70]
			56.76	12,029.13	TMLE untruncated	3.78	2.20	1.58 [1.28, 1.89]	63	
					TMLE truncated at 200	3.78	3.04	0.74 [0.46, 1.03]	135	
			3.16	667.50	IPW untruncated	3.64	2.18	1.46 [0.84, 2.08]	68	1.23 [0.89, 1.57]
					IPW truncated at 20	3.66	2.30	1.35 [0.73, 1.98]	74	1.13 [0.82, 1.44]
					IPW truncated at 99th	3.57	2.79	0.78 [0.09, 1.47]	128	0.89 [0.67, 1.11]
		Logistic model	4.22	2.54e+15	IPW untruncated	2.84	2.16	0.69 [-0.28, 1.65]		1.42 [0.67, 2.17]
					IPW truncated at 20	3.98	2.60	1.37 [0.32, 2.42]	73	1.02 [0.58, 1.46]
					IPW truncated at 99th	3.73	2.87	0.86 [0.06, 1.67]	116	0.90 [0.67, 1.14]
ITT		SL			Unadjusted	3.59	5.44	-1.85 [-2.51, -1.19]	54	0.59 [0.52, 0.67]
			51.30	15,530.99	TMLE untruncated	4.04	2.85	1.19 [0.74, 1.64]	84	
					TMLE truncated at 200	4.05	3.29	0.76 [0.41, 1.11]	132	
			2.99	1,619.89	IPW untruncated	3.93	2.88	1.04 [0.15, 1.94]	96	1.26 [0.95, 1.57]
					IPW truncated at 20	3.89	3.01	0.88 [-0.02, 1.79]		1.18 [0.89, 1.46]
					IPW truncated at 99th	3.81	3.42	0.39 [-0.42, 1.19]		0.93 [0.72, 1.13]
		Logistic model	3.79	6,946.90	IPW untruncated	4.09	2.83	1.26 [-0.69, 3.21]		1.43 [0.70, 2.15]
					IPW truncated at 20	4.01	3.51	0.50 [-1.07, 2.07]		1.04 [0.64, 1.45]
					IPW truncated at 99th	3.86	3.38	0.48 [-0.36, 1.32]		0.93 [0.72, 1.15]

* Discontinuation refers to the interruption of the comparator medication initiated on index date; Crossover refers to the initiation of the comparator medication initiated by patient at baseline in the other arm.

© 2025 Neugebauer R et al. *JAMA Network Open*.

eTable 5. MACE (Primary Definition), 4-Arm Drug Class Comparison, Sulfonylureas vs DPP4is vs SGLT2is vs GLP1-RAs, RD and HR Effect Measures for Comparing Sulfonylureas to GLP-1RAs at 2.5 Years

Estimation results from ITT and PP analyses to compare MACE risks over 2.5 years between SU and GLP-1RA initiators based on an emulated 4-arm RCT for comparing SU, DPP4i, SGLT2i, and GLP-1RA. For PP analyses, rates of protocol deviations are described by medication class initiated at baseline. Unadjusted point and interval estimates and adjusted point and interval IPW and TMLE estimates of risks, risk differences (RD), and hazard ratios (HR) based on propensity scores (PS) estimated with either logistic models or super learning (SL) are presented for four weight truncation schemes along with the corresponding 99th percentile and maximum value of the stabilized and unstabilized inverse probability weights used for implementing IPW and TMLE, respectively. RD is the risk in treatment arm minus the risk in control arm and NNT is the number needed to treat.

Analysis type	Protocol Deviations* by exposure group (%)	PS estimation	99th IP weights	Max IP weight	Estimator	Treatment (SU) risk in %	Control (GLP-1RA) risk in %	RD [95% CI] in %	NNT	HR [95% CI]
PP	Discontinuation<br>SU: 48.35<br>GLP-1RA: 41.38<br><br>Crossover<br>SU: 7.09<br>GLP-1RA: 11.42	SL			Unadjusted	3.38	3.16	0.22 [-0.56, 1.00]		1.29 [0.97, 1.61]
			56.76	12,029.13	TMLE untruncated	3.78	1.39	2.39 [2.09, 2.68]	42	
					TMLE truncated at 200	3.78	2.22	1.56 [1.31, 1.82]	64	
			3.16	667.50	IPW untruncated	3.64	1.37	2.27 [1.51, 3.04]	44	3.65 [1.98, 5.32]
					IPW truncated at 20	3.66	1.47	2.18 [1.39, 2.98]	46	3.11 [1.73, 4.49]
					IPW truncated at 99th	3.57	2.03	1.55 [0.50, 2.59]	65	2.15 [1.21, 3.10]
		Logistic model	4.22	2.54e+15	IPW untruncated	2.84	0.95	1.89 [1.35, 2.43]	53	4.00 [1.51, 6.48]
					IPW truncated at 20	3.98	1.26	2.71 [2.06, 3.36]	37	2.73 [1.12, 4.34]
					IPW truncated at 99th	3.73	1.82	1.91 [1.05, 2.77]	52	1.83 [0.80, 2.86]
ITT		SL			Unadjusted	3.59	3.82	-0.23 [-0.79, 0.34]		1.07 [0.85, 1.28]
			51.30	15,530.99	TMLE untruncated	4.04	2.27	1.77 [0.96, 2.57]	57	
					TMLE truncated at 200	4.05	3.15	0.91 [0.49, 1.33]	110	
			2.99	1,619.89	IPW untruncated	3.93	2.28	1.64 [0.37, 2.92]	61	1.54 [0.17, 2.91]
					IPW truncated at 20	3.89	2.95	0.94 [-0.48, 2.37]		1.24 [0.24, 2.25]
					IPW truncated at 99th	3.81	3.09	0.72 [-0.44, 1.87]		1.35 [0.81, 1.90]
		Logistic model	3.79	6,946.90	IPW untruncated	4.09	1.95	2.13 [0.81, 3.45]	47	1.92 [0.20, 3.63]
					IPW truncated at 20	4.01	3.16	0.85 [-0.76, 2.47]		1.27 [0.37, 2.17]
					IPW truncated at 99th	3.86	3.34	0.53 [-0.84, 1.89]		1.19 [0.66, 1.72]

* Discontinuation refers to the interruption of the comparator medication initiated on index date; Crossover refers to the initiation of the comparator medication initiated by patient at baseline in the other arm.

© 2025 Neugebauer R et al. *JAMA Network Open*.

eTable 6. MACE (Primary Definition), 4-Arm Drug Class Comparison, Sulfonylureas vs DPP4is vs SGLT2is vs GLP1-RAs, RD and HR Effect Measures for Comparing DPP4is to SGLT2is at 2.5 Years

Estimation results from ITT and PP analyses to compare MACE risks over 2.5 years between DPP4i and SGLT2i initiators based on an emulated 4-arm RCT for comparing SU, DPP4i, SGLT2i, and GLP-1RA. For PP analyses, rates of protocol deviations are described by medication class initiated at baseline. Unadjusted point and interval estimates and adjusted point and interval IPW and TMLE estimates of risks, risk differences (RD), and hazard ratios (HR) based on propensity scores (PS) estimated with either logistic models or super learning (SL) are presented for four weight truncation schemes along with the corresponding 99th percentile and maximum value of the stabilized and unstabilized inverse probability weights used for implementing IPW and TMLE, respectively. RD is the risk in treatment arm minus the risk in control arm and NNT is the number needed to treat.

Analysis type	Protocol Deviations* by exposure group (%)	PS estimation	99th IP weights	Max IP weight	Estimator	Treatment (DPP4i) risk in %	Control (SGLT2i) risk in %	RD [95% CI] in %	NNT	HR [95% CI]
PP	Discontinuation<br>DPP4i: 44.08<br>SGLT2i: 21.87<br><br>Crossover<br>DPP4i: 28.31<br>SGLT2i: 12.08	SL			Unadjusted	5.58	5.24	0.33 [-1.09, 1.75]		0.91 [0.68, 1.14]
			56.76	12,029.13	TMLE untruncated	4.62	2.20	2.43 [1.63, 3.22]	41	
					TMLE truncated at 200	5.10	3.04	2.06 [1.40, 2.72]	49	
			3.16	667.50	IPW untruncated	4.70	2.18	2.52 [0.89, 4.15]	40	1.17 [0.61, 1.73]
					IPW truncated at 20	4.72	2.30	2.41 [0.78, 4.05]	41	1.07 [0.57, 1.58]
					IPW truncated at 99th	4.91	2.79	2.12 [0.41, 3.83]	47	0.89 [0.51, 1.27]
		Logistic model	4.22	2.54e+15	IPW untruncated	5.04	2.16	2.89 [0.82, 4.95]	35	1.32 [0.39, 2.24]
					IPW truncated at 20	5.08	2.60	2.48 [0.36, 4.59]	40	0.99 [0.37, 1.62]
					IPW truncated at 99th	5.40	2.87	2.53 [0.45, 4.62]	39	0.95 [0.52, 1.38]
ITT		SL			Unadjusted	5.21	5.44	-0.23 [-1.15, 0.69]		0.86 [0.67, 1.06]
			51.30	15,530.99	TMLE untruncated	3.82	2.85	0.98 [0.25, 1.71]	102	
					TMLE truncated at 200	3.93	3.29	0.63 [0.01, 1.26]	159	
			2.99	1,619.89	IPW untruncated	3.82	2.88	0.94 [-0.32, 2.19]		1.19 [0.66, 1.72]
					IPW truncated at 20	3.84	3.01	0.83 [-0.44, 2.10]		1.11 [0.62, 1.60]
					IPW truncated at 99th	3.92	3.42	0.50 [-0.67, 1.67]		0.89 [0.55, 1.23]
		Logistic model	3.79	6,946.90	IPW untruncated	3.68	2.83	0.85 [-1.29, 2.99]		1.25 [0.43, 2.06]
					IPW truncated at 20	3.76	3.51	0.25 [-1.58, 2.07]		0.95 [0.41, 1.50]
					IPW truncated at 99th	3.94	3.38	0.56 [-0.67, 1.80]		0.88 [0.53, 1.24]

* Discontinuation refers to the interruption of the comparator medication initiated on index date; Crossover refers to the initiation of the comparator medication initiated by patient at baseline in the other arm.

© 2025 Neugebauer R et al. *JAMA Network Open*.

eTable 7. MACE (Primary Definition), 4-Arm Drug Class Comparison, Sulfonylureas vs DPP4is vs SGLT2is vs GLP1-RAs, RD and HR Effect Measures for Comparing DPP4is to GLP-1RAs at 2.5 Years

Estimation results from ITT and PP analyses to compare MACE risks over 2.5 years between DPP4i and GLP-1RA initiators based on an emulated 4-arm RCT for comparing SU, DPP4i, SGLT2i, and GLP-1RA. For PP analyses, rates of protocol deviations are described by medication class initiated at baseline. Unadjusted point and interval estimates and adjusted point and interval IPW and TMLE estimates of risks, risk differences (RD), and hazard ratios (HR) based on propensity scores (PS) estimated with either logistic models or super learning (SL) are presented for four weight truncation schemes along with the corresponding 99th percentile and maximum value of the stabilized and unstabilized inverse probability weights used for implementing IPW and TMLE, respectively. RD is the risk in treatment arm minus the risk in control arm and NNT is the number needed to treat.

Analysis type	Protocol Deviations* by exposure group (%)	PS estimation	99th IP weights	Max IP weight	Estimator	Treatment (DPP4i) risk in %	Control (GLP-1RA) risk in %	RD [95% CI] in %	NNT	HR [95% CI]
PP	<u>Discontinuation</u><br>DPP4i: 44.08<br>GLP-1RA: 41.38<br><br><u>Crossover</u><br>DPP4i: 28.31<br>GLP-1RA: 11.42	SL	[redacted]		Unadjusted	5.58	3.16	2.41 [1.10, 3.73]	41	1.93 [1.30, 2.55]
			56.76	12,029.13	TMLE untruncated	4.62	1.39	3.23 [2.44, 4.02]	31	[redacted]
					TMLE truncated at 200	5.10	2.22	2.88 [2.23, 3.52]	35	
			3.16	667.50	IPW untruncated	4.70	1.37	3.33 [1.64, 5.02]	30	3.46 [1.39, 5.54]
					IPW truncated at 20	4.72	1.47	3.24 [1.54, 4.95]	31	2.95 [1.21, 4.68]
					IPW truncated at 99th	4.91	2.03	2.88 [1.00, 4.77]	35	2.16 [0.95, 3.37]
		Logistic model	4.22	2.54e+15	IPW untruncated	5.04	0.95	4.09 [2.19, 6.00]	24	3.71 [0.84, 6.58]
					IPW truncated at 20	5.08	1.26	3.82 [1.87, 5.76]	26	2.67 [0.67, 4.66]
					IPW truncated at 99th	5.40	1.82	3.58 [1.47, 5.69]	28	1.93 [0.62, 3.23]
ITT	[redacted]	SL	[redacted]		Unadjusted	5.21	3.82	1.39 [0.54, 2.25]	72	1.55 [1.14, 1.97]
			51.30	15,530.99	TMLE untruncated	3.82	2.27	1.55 [0.56, 2.54]	64	[redacted]
					TMLE truncated at 200	3.93	3.15	0.78 [0.11, 1.45]	128	
			2.99	1,619.89	IPW untruncated	3.82	2.28	1.54 [-0.02, 3.09]	65	1.45 [0.05, 2.85]
					IPW truncated at 20	3.84	2.95	0.89 [-0.79, 2.57]		1.18 [0.13, 2.22]
					IPW truncated at 99th	3.92	3.09	0.83 [-0.61, 2.27]		1.29 [0.63, 1.95]
		Logistic model	3.79	6,946.90	IPW untruncated	3.68	1.95	1.73 [0.14, 3.32]	58	1.68 [0.03, 3.32]
					IPW truncated at 20	3.76	3.16	0.60 [-1.27, 2.46]		1.16 [0.21, 2.12]
					IPW truncated at 99th	3.94	3.34	0.61 [-1.03, 2.25]		1.12 [0.50, 1.75]

* Discontinuation refers to the interruption of the comparator medication initiated on index date; Crossover refers to the initiation of the comparator medication initiated by patient at baseline in the other arm.

© 2025 Neugebauer R et al. *JAMA Network Open*.

eTable 8. MACE (Primary Definition), 4-Arm Drug Class Comparison, Sulfonylureas vs DPP4is vs SGLT2is vs GLP1-RAs, RD and HR Effect Measures for Comparing SGLT2is to DPP4is at 2.5 Years

Estimation results from ITT and PP analyses to compare MACE risks over 2.5 years between SGLT2i and GLP-1RA initiators based on an emulated 4-arm RCT for comparing SU, DPP4i, SGLT2i, and GLP-1RA. For PP analyses, rates of protocol deviations are described by medication class initiated at baseline. Unadjusted point and interval estimates and adjusted point and interval IPW and TMLE estimates of risks, risk differences (RD), and hazard ratios (HR) based on propensity scores (PS) estimated with either logistic models or super learning (SL) are presented for four weight truncation schemes along with the corresponding 99th percentile and maximum value of the stabilized and unstabilized inverse probability weights used for implementing IPW and TMLE, respectively. RD is the risk in treatment arm minus the risk in control arm and NNT is the number needed to treat.

Analysis type	Protocol Deviations* by exposure group (%)	PS estimation	99th IP weights	Max IP weight	Estimator	Treatment (SGLT2i) risk in %	Control (GLP-1RA) risk in %	RD [95% CI] in %	NNT	HR [95% CI]
PP	Discontinuation<br>SGLT2i: 21.87<br>GLP-1RA: 41.38<br><br>Crossover<br>SGLT2i: 12.08<br>GLP-1RA: 11.42	SL	[redacted]	[redacted]	Unadjusted	5.24	3.16	2.08 [0.87, 3.30]	48	2.12 [1.52, 2.71]
			56.76	12,029.13	TMLE untruncated	2.20	1.39	0.81 [0.43, 1.18]	124	[redacted]
					TMLE truncated at 200	3.04	2.22	0.82 [0.49, 1.15]	122	
			3.16	667.50	IPW untruncated	2.18	1.37	0.81 [-0.15, 1.77]		2.97 [1.39, 4.54]
					IPW truncated at 20	2.30	1.47	0.83 [-0.15, 1.82]		2.74 [1.33, 4.16]
					IPW truncated at 99th	2.79	2.03	0.76 [-0.47, 2.00]		2.42 [1.21, 3.63]
		Logistic model	4.22	2.54e+15	IPW untruncated	2.16	0.95	1.21 [0.15, 2.26]	83	2.82 [0.54, 5.09]
					IPW truncated at 20	2.60	1.26	1.34 [0.15, 2.53]	75	2.68 [0.74, 4.63]
					IPW truncated at 99th	2.87	1.82	1.05 [-0.11, 2.20]		2.03 [0.78, 3.28]
ITT	[redacted]	SL	[redacted]	[redacted]	Unadjusted	5.44	3.82	1.63 [0.77, 2.48]	62	1.80 [1.38, 2.21]
			51.30	15,530.99	TMLE untruncated	2.85	2.27	0.58 [-0.33, 1.48]		[redacted]
					TMLE truncated at 200	3.29	3.15	0.15 [-0.37, 0.67]		
			2.99	1,619.89	IPW untruncated	2.88	2.28	0.60 [-0.94, 2.14]		1.22 [0.09, 2.34]
					IPW truncated at 20	3.01	2.95	0.06 [-1.62, 1.74]		1.06 [0.17, 1.95]
					IPW truncated at 99th	3.42	3.09	0.33 [-1.07, 1.73]		1.46 [0.79, 2.13]
		Logistic model	3.79	6,946.90	IPW untruncated	2.83	1.95	0.88 [-1.45, 3.20]		1.34 [-0.03, 2.72]
					IPW truncated at 20	3.51	3.16	0.35 [-1.89, 2.60]		1.22 [0.23, 2.20]
					IPW truncated at 99th	3.38	3.34	0.04 [-1.55, 1.64]		1.28 [0.64, 1.91]

* Discontinuation refers to the interruption of the comparator medication initiated on index date; Crossover refers to the initiation of the comparator medication initiated by patient at baseline in the other arm.

© 2025 Neugebauer R et al. *JAMA Network Open*.

eFigure 4. MACE (Primary Definition), 2-Arm Drug Class Comparison, Sulfonylureas vs DPP4is, CONSORT Diagram
Flow diagram describing the inclusion and exclusion steps and counts leading to the creation of the cohort for emulating the 2-arm RCT to compare the risk of MACE in new users of SU and DPP4i along with sample sizes and counts for each observed end of follow-up type by treatment initiated at cohort entry.

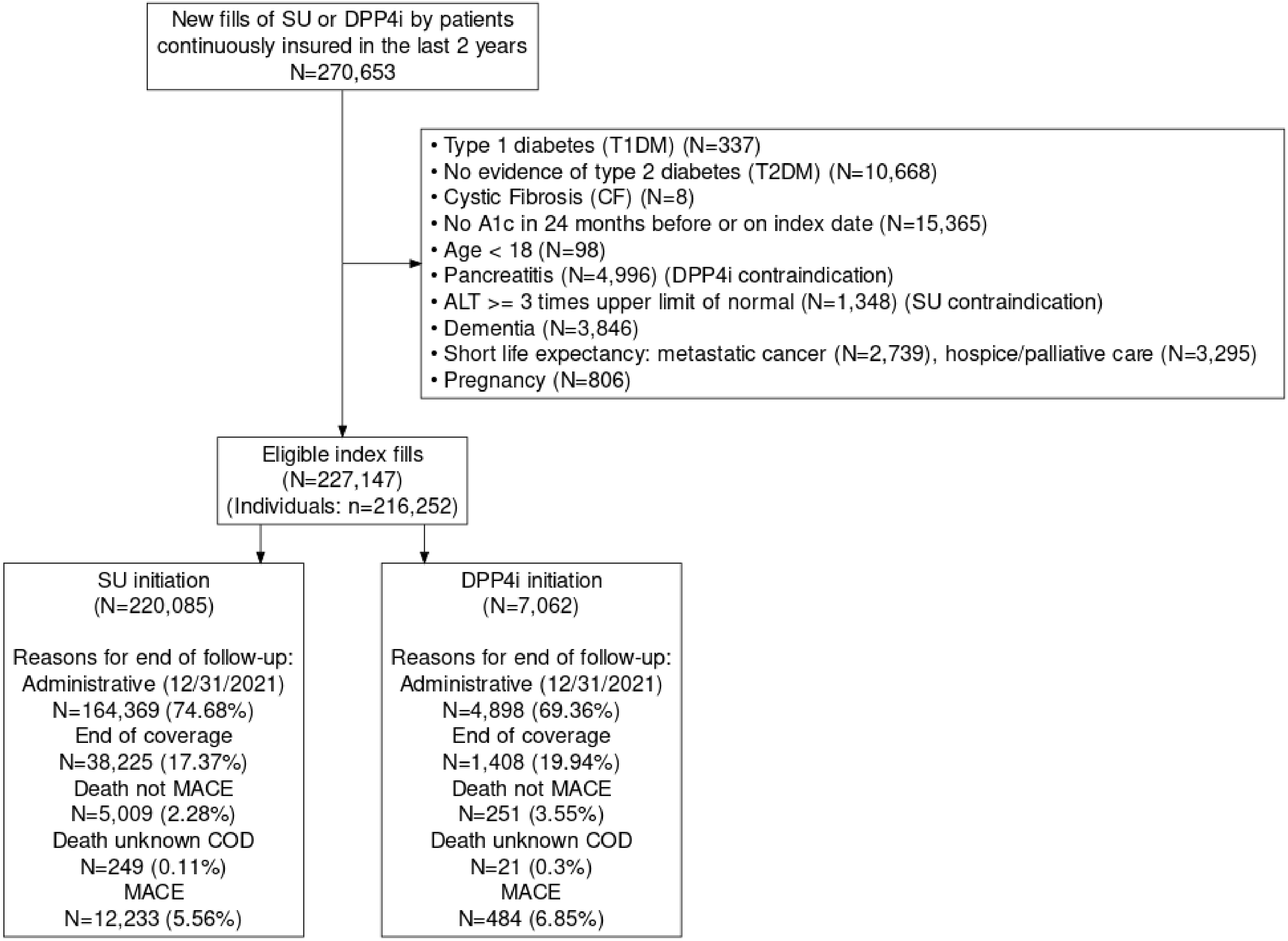

© 2025 Neugebauer R et al. *JAMA Network Open*.

eTable 9. MACE (Primary Definition), 2-Arm Drug Class Comparison, Sulfonylureas vs DPP4is, Patient Characteristics at Baseline (Overall and by Medication Initiated)

Summary statistics of the baseline values for selected covariates in the cohort of patients used to emulate a 2-arm RCT for comparing SU and DPP4i. For each continuous variable, the mean and standard deviation are displayed for all patients in the cohort (last column) and by drug class initiated at cohort entry. For each categorical variable and for each possible level of that variable, the count and proportion are displayed instead.

	SU<br>n = 220,085	DPP4i<br>n = 7,062	Total<br>n = 227,147
**Demographics**			
Age	57.06 (12.96)	61.65 (12.84)	57.20 (12.98)
Agegrp			
<45	36,992 (16.81%)	665 (9.42%)	37,657 (16.58%)
[45-65)	120,478 (54.74%)	3,429 (48.56%)	123,907 (54.55%)
[65-75)	43,187 (19.62%)	1,847 (26.15%)	45,034 (19.83%)
>=75	19,428 (8.83%)	1,121 (15.87%)	20,549 (9.05%)
Ethnicity			
Hispanic	81,383 (36.98%)	1,300 (18.41%)	82,683 (36.4%)
Nonhispanic	138,702 (63.02%)	5,762 (81.59%)	144,464 (63.6%)
Female Head Of Hh	0.150 (0.072)	0.137 (0.074)	0.150 (0.072)
Missing	1,021 (0.464%)	537 (7.604%)	1,558 (0.686%)
Hh Public Assistance	0.039 (0.035)	0.035 (0.033)	0.039 (0.035)
Missing	1,021 (0.464%)	537 (7.604%)	1,558 (0.686%)
Household Income Less 30k	0.206 (0.119)	0.226 (0.132)	0.207 (0.119)
Missing	1,021 (0.464%)	537 (7.604%)	1,558 (0.686%)
Houspoverty	0.105 (0.089)	0.101 (0.093)	0.105 (0.089)
Missing	1,026 (0.466%)	537 (7.604%)	1,563 (0.688%)
Index Yr			
2014	26,562 (12.07%)	440 (6.23%)	27,002 (11.89%)
2015	26,469 (12.03%)	688 (9.74%)	27,157 (11.96%)
2016	28,716 (13.05%)	948 (13.42%)	29,664 (13.06%)
2017	29,004 (13.18%)	991 (14.03%)	29,995 (13.21%)
2018	26,394 (11.99%)	963 (13.64%)	27,357 (12.04%)
2019	27,028 (12.28%)	1,116 (15.8%)	28,144 (12.39%)
2020	24,976 (11.35%)	993 (14.06%)	25,969 (11.43%)
2021	30,936 (14.06%)	923 (13.07%)	31,859 (14.03%)
Bmi	32.81 (7.31)	33.14 (7.68)	32.82 (7.32)
Missing	6,128 (2.78%)	319 (4.52%)	6,447 (2.84%)
Smoking Status			
Formersmoker	60,123 (27.32%)	2,235 (31.65%)	62,358 (27.45%)
Currentsmoker	20,268 (9.21%)	671 (9.5%)	20,939 (9.22%)
Passivesmoker	1,185 (0.54%)	26 (0.37%)	1,211 (0.53%)
Neversmoker	135,970 (61.78%)	3,997 (56.6%)	139,967 (61.62%)
Unknown	2,539 (1.15%)	133 (1.88%)	2,672 (1.18%)
Low Educ	0.180 (0.134)	0.138 (0.111)	0.179 (0.133)
Missing	1,018 (0.463%)	537 (7.604%)	1,555 (0.685%)
Mgr Male	0.047 (0.045)	0.042 (0.041)	0.046 (0.045)
Missing	1,020 (0.463%)	537 (7.604%)	1,557 (0.685%)
Ndi	0.29 (0.17)	0.26 (0.16)	0.28 (0.17)
Missing	1,026 (0.47%)	537 (7.6%)	1,563 (0.69%)
Pct Crowding	0.093 (0.087)	0.057 (0.073)	0.092 (0.087)
Missing	1,021 (0.464%)	537 (7.604%)	1,558 (0.686%)
Racegrp			
White	107,612 (48.9%)	4,450 (63.01%)	112,062 (49.33%)
Asian	35,000 (15.9%)	888 (12.57%)	35,888 (15.8%)
Blackorafricanamerican	21,914 (9.96%)	799 (11.31%)	22,713 (10%)
Hawaiianorpacificislander	3,461 (1.57%)	77 (1.09%)	3,538 (1.56%)
Americanindianoralaskanative	1,556 (0.71%)	30 (0.42%)	1,586 (0.7%)
Multirace	6,086 (2.77%)	239 (3.38%)	6,325 (2.78%)
Other	163 (0.07%)	35 (0.5%)	198 (0.09%)
Unknown	44,293 (20.13%)	544 (7.7%)	44,837 (19.74%)
Sex			
Female	100,360 (45.6%)	3,819 (54.08%)	104,179 (45.86%)

© 2025 Neugebauer R et al. *JAMA Network Open*.

	SU<br>n = 220,085	DPP4i<br>n = 7,062	Total<br>n = 227,147
Male	119,719 (54.4%)	3,243 (45.92%)	122,962 (54.13%)
Other	4 (0%)	0 (0%)	4 (0%)
Unknown	2 (0%)	0 (0%)	2 (0%)
Site			
Kpnc	90,750 (41.23%)	794 (11.24%)	91,544 (40.3%)
Kpsc	114,188 (51.88%)	2,576 (36.48%)	116,764 (51.4%)
Kphi	5,752 (2.61%)	220 (3.12%)	5,972 (2.63%)
Hpi	2,960 (1.34%)	680 (9.63%)	3,640 (1.6%)
Hfhs	3,802 (1.73%)	1,034 (14.64%)	4,836 (2.13%)
Ghs	2,633 (1.2%)	1,758 (24.89%)	4,391 (1.93%)
Unemployment	0.050 (0.027)	0.044 (0.028)	0.050 (0.027)
Missing	1,015 (0.461%)	537 (7.604%)	1,552 (0.683%)
**Insurance**			
Ins Commercial	171,073 (77.73%)	3,728 (52.79%)	174,801 (76.96%)
Ins Highdeductible	19,892 (9.04%)	308 (4.36%)	20,200 (8.89%)
Ins Medicaid	13,260 (6.02%)	872 (12.35%)	14,132 (6.22%)
Ins Medicare	61,783 (28.07%)	2,954 (41.83%)	64,737 (28.5%)
Ins Medicare A	36,536 (16.6%)	1,986 (28.12%)	38,522 (16.96%)
Ins Medicare B	34,233 (15.55%)	1,889 (26.75%)	36,122 (15.9%)
Ins Medicare C	29,338 (13.33%)	1,734 (24.55%)	31,072 (13.68%)
Ins Medicare D	31,898 (14.49%)	1,719 (24.34%)	33,617 (14.8%)
Ins Other Coverage	48,776 (22.16%)	1,462 (20.7%)	50,238 (22.12%)
Ins Privatepay	41,342 (18.78%)	953 (13.49%)	42,295 (18.62%)
Ins Selffunded	4,002 (1.82%)	315 (4.46%)	4,317 (1.9%)
Ins Statesubsidized	746 (0.34%)	432 (6.12%)	1,178 (0.52%)
**Clinical data**			
A1c Age	42.51 (78.64)	58.95 (105.08)	43.02 (79.65)
Missing	226 (0.1%)	18 (0.25%)	244 (0.11%)
Acc Aha 201310yrcvdrisk	0.168 (0.151)	0.211 (0.171)	0.170 (0.152)
Missing	26,223 (11.915%)	844 (11.951%)	27,067 (11.916%)
Chf Dx Status			
0	210,278 (95.54%)	6,391 (90.5%)	216,669 (95.39%)
1	8,906 (4.05%)	602 (8.52%)	9,508 (4.19%)
999	901 (0.41%)	69 (0.98%)	970 (0.43%)
Cv Risk Subgrp			
Low	85,219 (38.72%)	2,087 (29.55%)	87,306 (38.44%)
Moderate	44,656 (20.29%)	1,350 (19.12%)	46,006 (20.25%)
High	59,562 (27.06%)	2,495 (35.33%)	62,057 (27.32%)
Other	4,916 (2.23%)	313 (4.43%)	5,229 (2.3%)
Unknown	25,732 (11.69%)	817 (11.57%)	26,549 (11.69%)
Diab Duration	4.93 (3.24)	6.12 (3.34)	4.97 (3.25)
Missing	364 (0.17%)	37 (0.52%)	401 (0.18%)
A1c	9.22 (2.08)	8.24 (1.65)	9.19 (2.07)
Missing	226 (0.1%)	18 (0.25%)	244 (0.11%)
Acr	47.10 (84.20)	53.27 (94.79)	47.28 (84.53)
Missing	44,204 (20.08%)	1,776 (25.15%)	45,980 (20.24%)
Afib Dx	9,011 (4.09%)	576 (8.16%)	9,587 (4.22%)
Alt	33.12 (22.16)	30.05 (18.80)	33.02 (22.06)
Missing	46,210 (21%)	1,303 (18.45%)	47,513 (20.92%)
Amputation Dxpx	1,379 (0.63%)	99 (1.4%)	1,478 (0.65%)
Anemia Dx	14,018 (6.37%)	989 (14%)	15,007 (6.61%)
Anxiety Dx	38,064 (17.3%)	1,683 (23.83%)	39,747 (17.5%)
Arrythmia Dx	8,007 (3.64%)	619 (8.77%)	8,626 (3.8%)
Ascvd Dxpx Max	21,000 (9.54%)	1,289 (18.25%)	22,289 (9.81%)
Ascvd Dxpx Ppv	4,916 (2.23%)	313 (4.43%)	5,229 (2.3%)
Asthma Dx	22,465 (10.21%)	810 (11.47%)	23,275 (10.25%)
Bariatric Px	2,242 (1.02%)	134 (1.9%)	2,376 (1.05%)

© 2025 Neugebauer R et al. *JAMA Network Open*.

	SU n = 220,085	DPP4i n = 7,062	Total n = 227,147
Bipolar Dx	1,971 (0.9%)	107 (1.52%)	2,078 (0.91%)
Blind Dx	792 (0.36%)	31 (0.44%)	823 (0.36%)
Cad Dxpx Max	13,113 (5.96%)	780 (11.05%)	13,893 (6.12%)
Cad Dxpx Ppv	2,999 (1.36%)	208 (2.95%)	3,207 (1.41%)
Cad Dxpx Sens	3,372 (1.53%)	240 (3.4%)	3,612 (1.59%)
Cancer Mets Dx	0 (0%)	0 (0%)	0 (0%)
Cancer Nomets Dx	8,282 (3.76%)	481 (6.81%)	8,763 (3.86%)
Cevd Dxpx Ppv	1,871 (0.85%)	91 (1.29%)	1,962 (0.86%)
Cevd Dxpx Sens	6,928 (3.15%)	436 (6.17%)	7,364 (3.24%)
Chf Dx Ppv	2,087 (0.95%)	143 (2.02%)	2,230 (0.98%)
Chf Dx Sens	8,906 (4.05%)	602 (8.52%)	9,508 (4.19%)
Ckd Dx	45,707 (20.77%)	2,137 (30.26%)	47,844 (21.06%)
Copd Dx	7,931 (3.6%)	524 (7.42%)	8,455 (3.72%)
Coupled Dbp	74.93 (10.79)	72.87 (11.06)	74.87 (10.80)
Missing	3,911 (1.78%)	300 (4.25%)	4,211 (1.85%)
Coupled Sbp	129.52 (14.58)	129.12 (15.32)	129.51 (14.60)
Missing	3,911 (1.78%)	300 (4.25%)	4,211 (1.85%)
Covid Prd	55,912 (25.4%)	1,916 (27.13%)	57,828 (25.46%)
Creat	0.91 (0.51)	1.07 (0.92)	0.92 (0.53)
Missing	5,937 (2.7%)	253 (3.58%)	6,190 (2.73%)
Cysticfibrosis Dx	0 (0%)	0 (0%)	0 (0%)
Dbp	74.08 (10.78)	72.26 (11.10)	74.02 (10.80)
Missing	3,908 (1.78%)	300 (4.25%)	4,208 (1.85%)
Dementia Dx	0 (0%)	0 (0%)	0 (0%)
Depr Dx	28,909 (13.14%)	1,439 (20.38%)	30,348 (13.36%)
Dietitian	10,285 (4.67%)	537 (7.6%)	10,822 (4.76%)
Dka Dx	2,974 (1.35%)	116 (1.64%)	3,090 (1.36%)
Dka Dx Count	0.01 (0.13)	0.02 (0.16)	0.01 (0.13)
Esrd Dx	10,145 (4.61%)	777 (11%)	10,922 (4.81%)
Esrd Px	755 (0.34%)	107 (1.52%)	862 (0.38%)
Etoh Dx	3,637 (1.65%)	129 (1.83%)	3,766 (1.66%)
Fasciitis Dx	166 (0.08%)	13 (0.18%)	179 (0.08%)
Fpg	181.00 (73.93)	159.69 (62.68)	180.59 (73.79)
Missing	145,096 (65.93%)	5,594 (79.21%)	150,690 (66.34%)
Frailty Dx	14,287 (6.49%)	1,142 (16.17%)	15,429 (6.79%)
Gfr Epi 09	88.02 (22.90)	78.37 (26.75)	87.72 (23.09)
Missing	5,937 (2.7%)	253 (3.58%)	6,190 (2.73%)
Hdl	44.56 (11.46)	44.66 (12.23)	44.56 (11.48)
Missing	31,053 (14.11%)	850 (12.04%)	31,903 (14.05%)
Hgb	13.99 (1.65)	13.44 (1.68)	13.98 (1.66)
Missing	60,799 (27.63%)	1,540 (21.81%)	62,339 (27.44%)
Htn Dx	120,504 (54.75%)	4,973 (70.42%)	125,477 (55.24%)
Hypo Dx	188 (0.09%)	23 (0.33%)	211 (0.09%)
Hypo Dx Count	0.01 (0.13)	0.03 (0.22)	0.01 (0.13)
Hypo Dx Event	188 (0.09%)	23 (0.33%)	211 (0.09%)
Hypothyroidism Dx	21,162 (9.62%)	1,221 (17.29%)	22,383 (9.85%)
Ldl	96.37 (39.07)	89.53 (36.90)	96.16 (39.02)
Missing	24,628 (11.19%)	659 (9.33%)	25,287 (11.13%)
Leukemia Lymphoma Dx	1,294 (0.59%)	76 (1.08%)	1,370 (0.6%)
Lipid Dx	130,619 (59.35%)	5,053 (71.55%)	135,672 (59.73%)
Liver Dx	331 (0.15%)	29 (0.41%)	360 (0.16%)
Mci Dx	1,021 (0.46%)	63 (0.89%)	1,084 (0.48%)
Men2 Dx	2 (0%)	1 (0.01%)	3 (0%)
Nephropathy Dx	13,113 (5.96%)	551 (7.8%)	13,664 (6.02%)
Neuro Dx	5,121 (2.33%)	321 (4.55%)	5,442 (2.4%)
Pancreatitis Dx	0 (0%)	0 (0%)	0 (0%)
Pcr	0.000 (0.000)	0.000 (0.000)	0.000 (0.000)

© 2025 Neugebauer R et al. *JAMA Network Open*.

	SU n = 220,085	DPP4i n = 7,062	Total n = 227,147
Missing	205,346 (93.303%)	6,569 (93.019%)	211,915 (93.294%)
Potassium	4.24 (0.40)	4.30 (0.44)	4.24 (0.40)
Missing	17,493 (7.95%)	380 (5.38%)	17,873 (7.87%)
Pregnancy	0 (0%)	0 (0%)	0 (0%)
Pud Dx	225 (0.1%)	9 (0.13%)	234 (0.1%)
Pvd Dxpx Ppv	423 (0.19%)	35 (0.5%)	458 (0.2%)
Pvd Dxpx Sens	3,926 (1.78%)	325 (4.6%)	4,251 (1.87%)
Pyelo Dx	2,304 (1.05%)	89 (1.26%)	2,393 (1.05%)
Retinopathy Dxpx	7,252 (3.3%)	462 (6.54%)	7,714 (3.4%)
Rpg	214.89 (113.42)	182.32 (84.47)	213.17 (112.31)
Missing	141,260 (64.18%)	2,664 (37.72%)	143,924 (63.36%)
Sbp	128.71 (14.41)	128.50 (15.23)	128.71 (14.43)
Missing	3,911 (1.78%)	300 (4.25%)	4,211 (1.85%)
Schiz Dx	1,325 (0.6%)	50 (0.71%)	1,375 (0.61%)
Sodium	138.05 (3.07)	138.74 (2.98)	138.08 (3.07)
Missing	49,924 (22.68%)	666 (9.43%)	50,590 (22.27%)
Sud Dx	2,094 (0.95%)	89 (1.26%)	2,183 (0.96%)
Tc	177.04 (52.00)	166.84 (46.45)	176.72 (51.86)
Missing	30,196 (13.72%)	846 (11.98%)	31,042 (13.67%)
Trig	214.31 (210.06)	186.13 (148.81)	213.40 (208.42)
Missing	41,984 (19.08%)	1,101 (15.59%)	43,085 (18.97%)
Tsh	2.04 (1.91)	2.19 (2.21)	2.05 (1.92)
Missing	79,503 (36.12%)	2,492 (35.29%)	81,995 (36.1%)
Valvular Dx	3,750 (1.7%)	342 (4.84%)	4,092 (1.8%)
Vasculitis Dx	2,712 (1.23%)	132 (1.87%)	2,844 (1.25%)
Only Met No Ascvd	123,719 (56.21%)	2,593 (36.72%)	126,312 (55.61%)
Renal Function Status			
Lowrisk	112,760 (51.23%)	3,045 (43.12%)	115,805 (50.98%)
Moderaterisk	42,279 (19.21%)	1,264 (17.9%)	43,543 (19.17%)
Highrisk	13,861 (6.3%)	572 (8.1%)	14,433 (6.35%)
Veryhighrisk	5,730 (2.6%)	382 (5.41%)	6,112 (2.69%)
Unknown	45,455 (20.65%)	1,799 (25.47%)	47,254 (20.8%)
Total Visit C	0.45 (1.82)	1.20 (3.71)	0.48 (1.91)
Total Visit E	0.15 (1.15)	0.77 (2.97)	0.17 (1.25)
Total Visit N	0.17 (2.48)	0.63 (4.86)	0.18 (2.59)
**Concurrent medications**			
Aa	2 (0%)	0 (0%)	2 (0%)
Aceinhibitors	76,605 (34.81%)	2,614 (37.02%)	79,219 (34.88%)
Agi	232 (0.11%)	53 (0.75%)	285 (0.13%)
Anticoagulants	7,358 (3.34%)	509 (7.21%)	7,867 (3.46%)
Anticonvulsants	12,937 (5.88%)	864 (12.23%)	13,801 (6.08%)
Antidepressantcomb	3 (0%)	0 (0%)	3 (0%)
Antidepressantmaoi	27 (0.01%)	2 (0.03%)	29 (0.01%)
Antidepressantndri	3,813 (1.73%)	219 (3.1%)	4,032 (1.78%)
Antidepressantother	0 (0%)	0 (0%)	0 (0%)
Antidepressantsari	5,410 (2.46%)	264 (3.74%)	5,674 (2.5%)
Antidepressantsnri	5,962 (2.71%)	411 (5.82%)	6,373 (2.81%)
Antidepressantspo	62 (0.03%)	10 (0.14%)	72 (0.03%)
Antidepressantssri	17,820 (8.1%)	910 (12.89%)	18,730 (8.25%)
Antidepressanttca	5,881 (2.67%)	218 (3.09%)	6,099 (2.69%)
Antidepressantteca	1,256 (0.57%)	75 (1.06%)	1,331 (0.59%)
Antiplatelets	12,332 (5.6%)	709 (10.04%)	13,041 (5.74%)
Antipsychotic1stgen	468 (0.21%)	31 (0.44%)	499 (0.22%)
Antipsychotic2ndgen	3,233 (1.47%)	192 (2.72%)	3,425 (1.51%)
Anxiety	2,429 (1.1%)	168 (2.38%)	2,597 (1.14%)
Arb	36,472 (16.57%)	1,693 (23.97%)	38,165 (16.8%)
Benzodiazepines	7,930 (3.6%)	441 (6.24%)	8,371 (3.69%)

© 2025 Neugebauer R et al. *JAMA Network Open*.

	SU<br>n = 220,085	DPP4i<br>n = 7,062	Total<br>n = 227,147
Betablockers	47,583 (21.62%)	2,383 (33.74%)	49,966 (22%)
Clonidine	1,961 (0.89%)	114 (1.61%)	2,075 (0.91%)
Dihydropyridineccb	29,024 (13.19%)	1,379 (19.53%)	30,403 (13.38%)
Glp1	820 (0.37%)	175 (2.48%)	995 (0.44%)
Hypnoticother	1,625 (0.74%)	103 (1.46%)	1,728 (0.76%)
Injectableantipsychotic	13 (0.01%)	4 (0.06%)	17 (0.01%)
Ins	16,792 (7.63%)	2,025 (28.67%)	18,817 (8.28%)
Ins Analog	3,527 (1.6%)	1,072 (15.18%)	4,599 (2.02%)
Ins Combo	1,563 (0.71%)	253 (3.58%)	1,816 (0.8%)
Ins Human	14,103 (6.41%)	1,123 (15.9%)	15,226 (6.7%)
Ins La	14,548 (6.61%)	1,648 (23.34%)	16,196 (7.13%)
Ins Sa	4,418 (2.01%)	997 (14.12%)	5,415 (2.38%)
Ksparingdiuretics	5,725 (2.6%)	359 (5.08%)	6,084 (2.68%)
Lithium	337 (0.15%)	18 (0.25%)	355 (0.16%)
Loopdiuretics	10,474 (4.76%)	862 (12.21%)	11,336 (4.99%)
Meg	124 (0.06%)	83 (1.18%)	207 (0.09%)
Met	148,512 (67.48%)	4,590 (65%)	153,102 (67.4%)
Nondihydropyridineccb	3,514 (1.6%)	205 (2.9%)	3,719 (1.64%)
Otherlipidmeds	5,730 (2.6%)	346 (4.9%)	6,076 (2.67%)
Pcsk9mab	29 (0.01%)	4 (0.06%)	33 (0.01%)
Sglt2	1,351 (0.61%)	378 (5.35%)	1,729 (0.76%)
Statins	123,704 (56.21%)	4,616 (65.36%)	128,320 (56.49%)
Stimulants	860 (0.39%)	48 (0.68%)	908 (0.4%)
Suold	1,502 (0.68%)	7 (0.1%)	1,509 (0.66%)
Thiazidediuretics	50,514 (22.95%)	1,695 (24%)	52,209 (22.98%)
Tir	0 (0%)	0 (0%)	0 (0%)
Tzd	1,896 (0.86%)	216 (3.06%)	2,112 (0.93%)
Only Met Therapy	134,538 (61.13%)	3,008 (42.59%)	137,546 (60.55%)

© 2025 Neugebauer R et al. *JAMA Network Open*.

eFigure 5. MACE (Primary Definition), 2-Arm Drug Class Comparison, Sulfonylureas vs DPP4is, Cumulative Incidence Curves From PP and ITT Analyses With IPW, TMLE, and SL
Each plot emulates inferences from a 2-arm RCT comparing SU and DPP4i and represents unadjusted or adjusted estimates of cumulative incidence curves for MACE derived with inverse probability weighting (IPW) and Targeted Minimum Loss-based Estimation (TMLE) with Super Learning (SL) estimates of propensity scores with four weight truncation schemes: IPW and TMLE without weight truncation (untruncated), IPW with truncation of stabilized weights at value 20 (trunc20) or at the 99th percentile of weight values (trunc99), and TMLE with truncation of unstabilized weights at value 200 (trunc200). The red divider line separates results of Per-Protocol (PP) analyses (top half) from Intention-To-Treat (ITT) analyses (bottom half). Each plot displays a p value for the test that the average risk difference (ARD) through 2.5 years of follow-up (30 months) is 0.

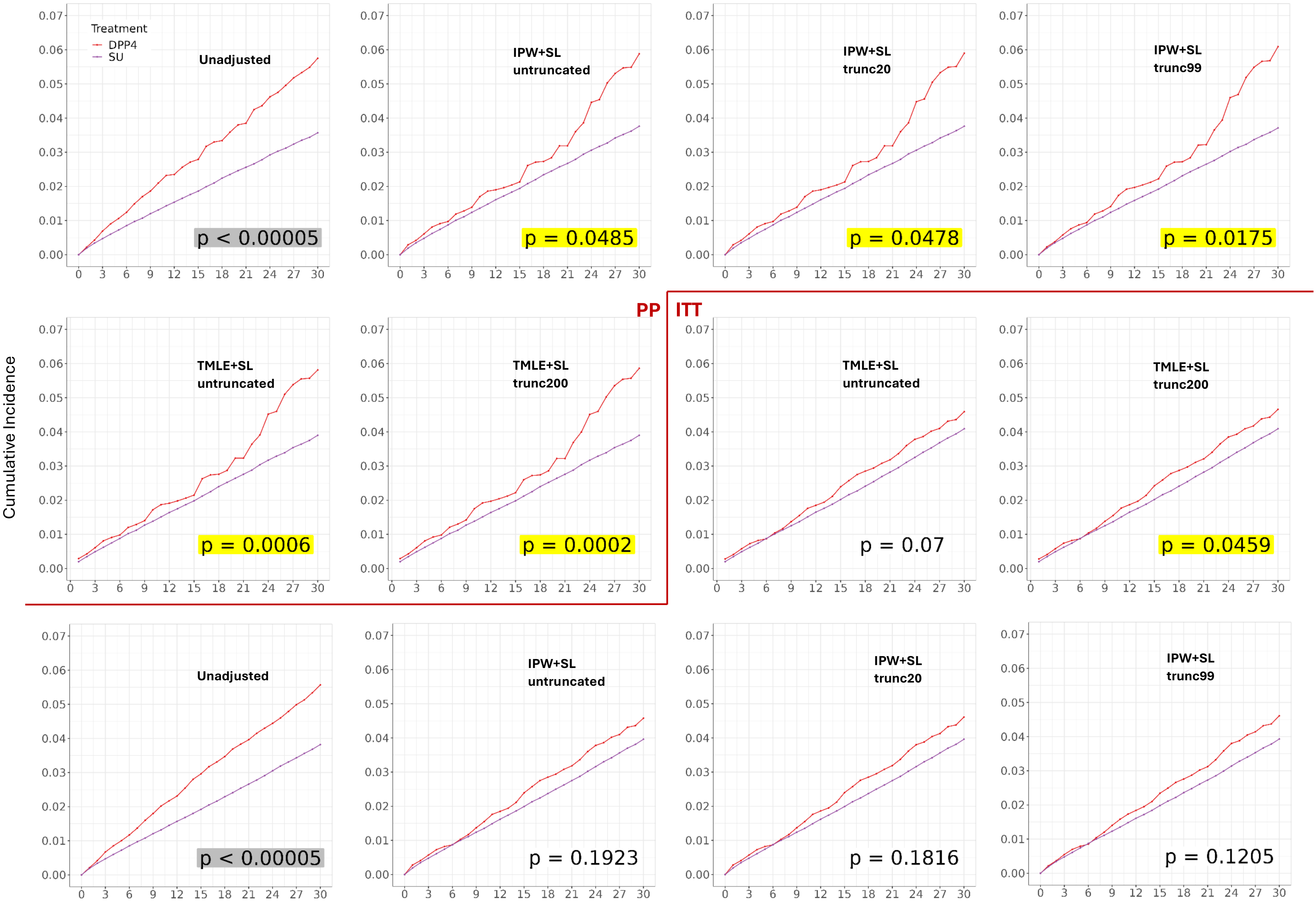

© 2025 Neugebauer R et al. *JAMA Network Open*.

eFigure 6. MACE (Primary Definition), 2-Arm Drug Class Comparison, Sulfonylureas vs DPP4is, Cumulative Incidence Curves From Sensitivity PP Analyses With IPW, TMLE, and SL
Each plot emulates inferences from a 2-arm RCT comparing SU and DPP4i and represents unadjusted or adjusted estimates of cumulative incidence curves for MACE from sensitivity PP analyses referred to as “NoMBS PP” and “No3 PP”. NoMBS PP analyses are restricted to patients without a history of MBS at baseline and the protocols they evaluate preclude metabolic bariatric surgery (MBS) procedures. The protocols in the No3 PP analyses preclude exposure to three medication classes: the comparator medication from the other arm, SGLT2i and GLP-1RA.

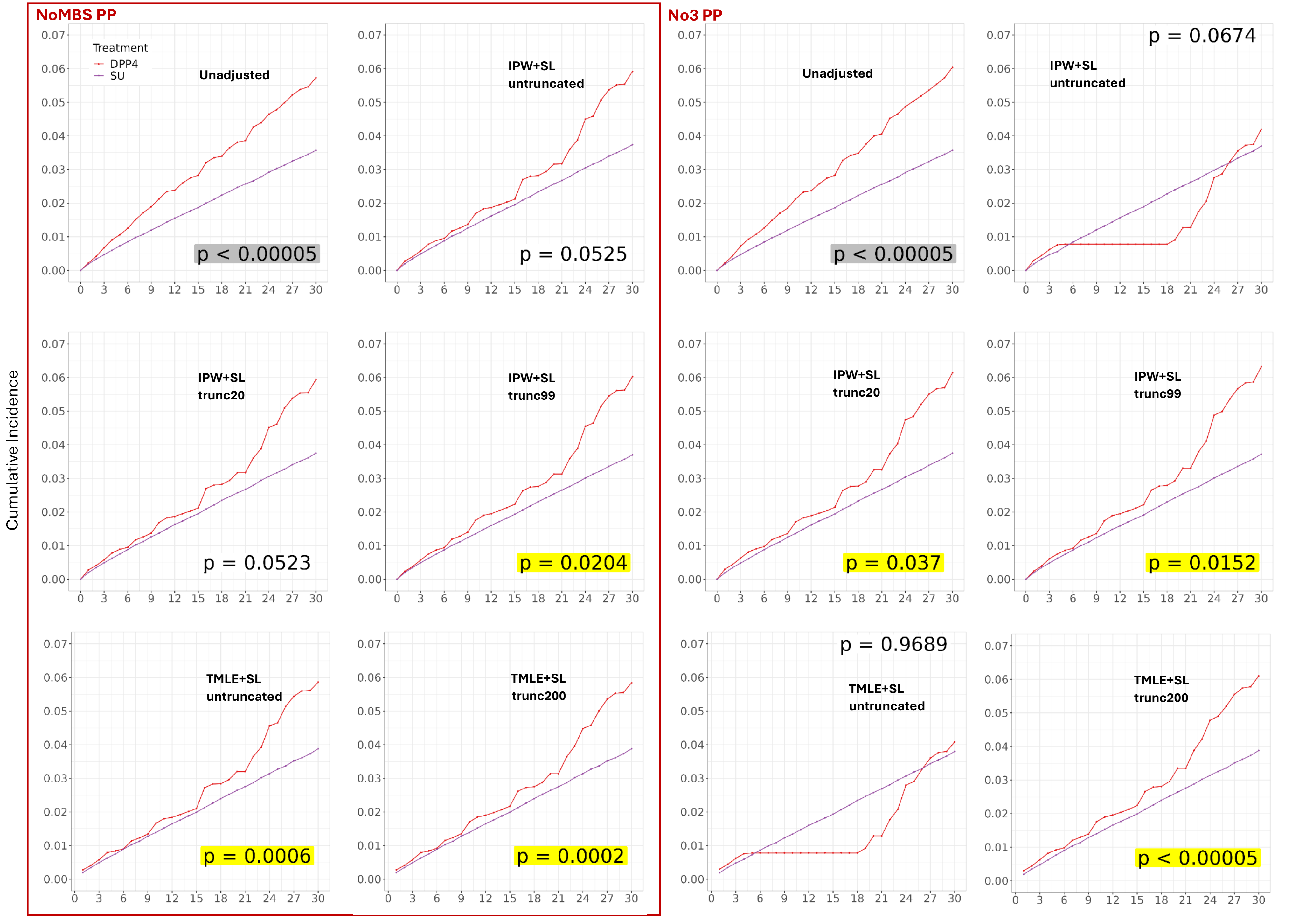

© 2025 Neugebauer R et al. *JAMA Network Open*.

eTable 10. MACE (Primary Definition), 2-Arm Drug Class Comparison, Sulfonylureas vs DPP4is, RD and HR Effect Measures at 2.5 Years

Estimation results from ITT, PP, NoMBS PP, and No3 PP analyses of emulated 2-arm RCTs comparing MACE risks over 2.5 years between SU and DPP4i initiators. For PP analyses, rates of protocol deviations are described by medication class initiated at baseline. Unadjusted point and interval estimates and adjusted point and interval IPW and TMLE estimates of risks, risk differences (RD), and hazard ratios (HR) based on propensity scores (PS) estimated with either logistic models or super learning (SL) are presented for four weight truncation schemes along with the corresponding 99th percentile and maximum value of the stabilized and unstabilized inverse probability weights used for implementing IPW and TMLE, respectively. RD is the risk in treatment arm minus the risk in control arm and NNT is the number needed to treat.

Analysis type	Protocol Deviations* by exposure group (%)	PS estimation	99th IP weights	Max IP weight	Estimator	Treatment (DPP4i) risk in %	Control (SU) risk in %	RD [95% CI] in %	NNT	HR [95% CI]
PP	Discontinuation<br>SU: 49.47%<br>DPP4i: 53.34%<br><br>Crossover<br>SU: 2.25%<br>DPP4i: 14.44%	SL			Unadjusted	5.75	3.57	2.18 [1.22, 3.15]	46	1.53 [1.28, 1.79]
			21.66	5,325.45	TMLE untruncated	5.81	3.90	1.91 [1.13, 2.70]	52	
					TMLE truncated at 200	5.86	3.90	1.96 [1.30, 2.62]	51	
			2.28	127.94	IPW untruncated	5.88	3.76	2.12 [0.40, 3.84]	47	1.21 [0.84, 1.57]
					IPW truncated at 20	5.90	3.76	2.14 [0.41, 3.87]	47	1.21 [0.84, 1.57]
					IPW truncated at 99th	6.09	3.71	2.37 [0.64, 4.11]	42	1.22 [0.90, 1.53]
		Logistic model	2.89	520.95	IPW untruncated	5.98	4.28	1.70 [-0.50, 3.90]		1.16 [0.73, 1.59]
					IPW truncated at 20	6.03	3.99	2.04 [-0.12, 4.19]		1.21 [0.77, 1.66]
					IPW truncated at 99th	6.00	3.81	2.19 [0.41, 3.97]	46	1.24 [0.89, 1.59]
ITT		SL			Unadjusted	5.57	3.82	1.76 [1.12, 2.39]	57	1.52 [1.31, 1.72]
			21.78	6,849.86	TMLE untruncated	4.59	4.09	0.49 [-0.04, 1.03]		
					TMLE truncated at 200	4.66	4.09	0.57 [0.03, 1.10]	176	
			1.87	134.53	IPW untruncated	4.58	3.96	0.63 [-0.30, 1.55]		1.17 [0.88, 1.45]
					IPW truncated at 20	4.61	3.96	0.65 [-0.28, 1.58]		1.17 [0.88, 1.46]
					IPW truncated at 99th	4.61	3.93	0.68 [-0.14, 1.51]		1.16 [0.92, 1.40]
		Logistic model	2.55	Inf	IPW untruncated	4.63	2.48	2.15 [1.07, 3.23]	47	2.00 [1.43, 2.58]
					IPW truncated at 20	4.73	4.05	0.69 [-0.41, 1.79]		1.16 [0.84, 1.48]
					IPW truncated at 99th	4.89	3.98	0.91 [-0.04, 1.86]		1.21 [0.94, 1.48]
NoMBS PP	Discontinuation<br>SU: 49.13%<br>DPP4i: 53.07%<br><br>Crossover<br>SU: 2.65%<br>DPP4i: 14.90%<br><br>MBS occurrence<br>SU: 0.42%<br>DPP4i: 0.38%	SL			Unadjusted	5.73	3.57	2.16 [1.19, 3.12]	46	1.54 [1.28, 1.79]
			21.14	5,031.88	TMLE untruncated	5.86	3.88	1.98 [1.19, 2.78]	50	
					TMLE truncated at 200	5.84	3.88	1.96 [1.31, 2.62]	51	
			2.24	108.00	IPW untruncated	5.92	3.74	2.18 [0.40, 3.96]	46	1.19 [0.83, 1.54]
					IPW truncated at 20	5.94	3.75	2.19 [0.40, 3.98]	46	1.19 [0.83, 1.55]
					IPW truncated at 99th	6.03	3.70	2.33 [0.60, 4.05]	43	1.20 [0.89, 1.51]
		Logistic model	2.88	624.90	IPW untruncated	5.93	4.26	1.67 [-0.49, 3.83]		1.16 [0.73, 1.58]
					IPW truncated at 20	5.97	4.00	1.97 [-0.15, 4.08]		1.20 [0.76, 1.64]
					IPW truncated at 99th	6.04	3.81	2.23 [0.41, 4.06]	45	1.24 [0.89, 1.59]
No3 PP	Discontinuation<br>SU: 47.82%<br>DPP4i: 40.19%<br><br>Crossover to comparator drug<br>SU: 2.07%<br>DPP4i: 12.76%<br><br>Initiation of one of the two non-comparator drugs<br>SU: 5.99%<br>DPP4i: 20.99%	SL			Unadjusted	6.04	3.57	2.46 [1.40, 3.53]	41	1.57 [1.29, 1.85]
			19.74	7.15e+16	TMLE untruncated	4.08	3.80	0.28 [-1.75, 2.30]		
					TMLE truncated at 200	6.10	3.88	2.22 [1.57, 2.86]	45	
			2.17	5.95e+14	IPW untruncated	4.20	3.70	0.50 [-1.18, 2.18]		0 [0, 0]
					IPW truncated at 20	6.14	3.75	2.39 [0.53, 4.24]	42	1.20 [0.81, 1.59]
					IPW truncated at 99th	6.32	3.72	2.61 [0.73, 4.49]	38	1.21 [0.87, 1.55]
		Logistic model	2.72	3.65e+19	IPW untruncated	4.11	3.04	1.06 [-0.85, 2.97]		0 [0, 0]
					IPW truncated at 20	6.14	4.14	2.00 [-0.22, 4.22]		1.16 [0.69, 1.63]
					IPW truncated at 99th	6.02	3.83	2.19 [0.35, 4.03]	46	1.19 [0.83, 1.56]

* Discontinuation refers to the interruption of the comparator medication initiated on index date; Crossover refers to the initiation of the comparator medication initiated by patient at baseline in the other arm; MBS occurrence refers to patient's undergoing metabolic bariatric surgery (MBS).

© 2025 Neugebauer R et al. *JAMA Network Open*.

eFigure 7. MACE (Primary Definition), 2-Arm Drug Class Comparison, Sulfonylureas vs DPP4is, ASCVD Subgroup, Cumulative Incidence Curves From PP and ITT Analyses With IPW, TMLE, and SL
Each plot emulates inferences among patients with ASCVD from a 2-arm RCT comparing SU and DPP4i and represents unadjusted or adjusted estimates of cumulative incidence curves for MACE derived with IPW and TMLE with SL estimates of propensity scores with four weight truncation schemes: IPW and TMLE without weight truncation (untruncated), IPW with truncation of stabilized weights at value 20 (trunc20) or at the 99th percentile of weight values (trunc99), and TMLE with truncation of unstabilized weights at value 200 (trunc200). The red divider line separates results of Per-Protocol (PP) analyses (top half) from Intention-To-Treat (ITT) analyses (bottom half). Each plot displays a p value for the test that the average risk difference (ARD) through 2.5 years of follow-up (30 months) is 0.

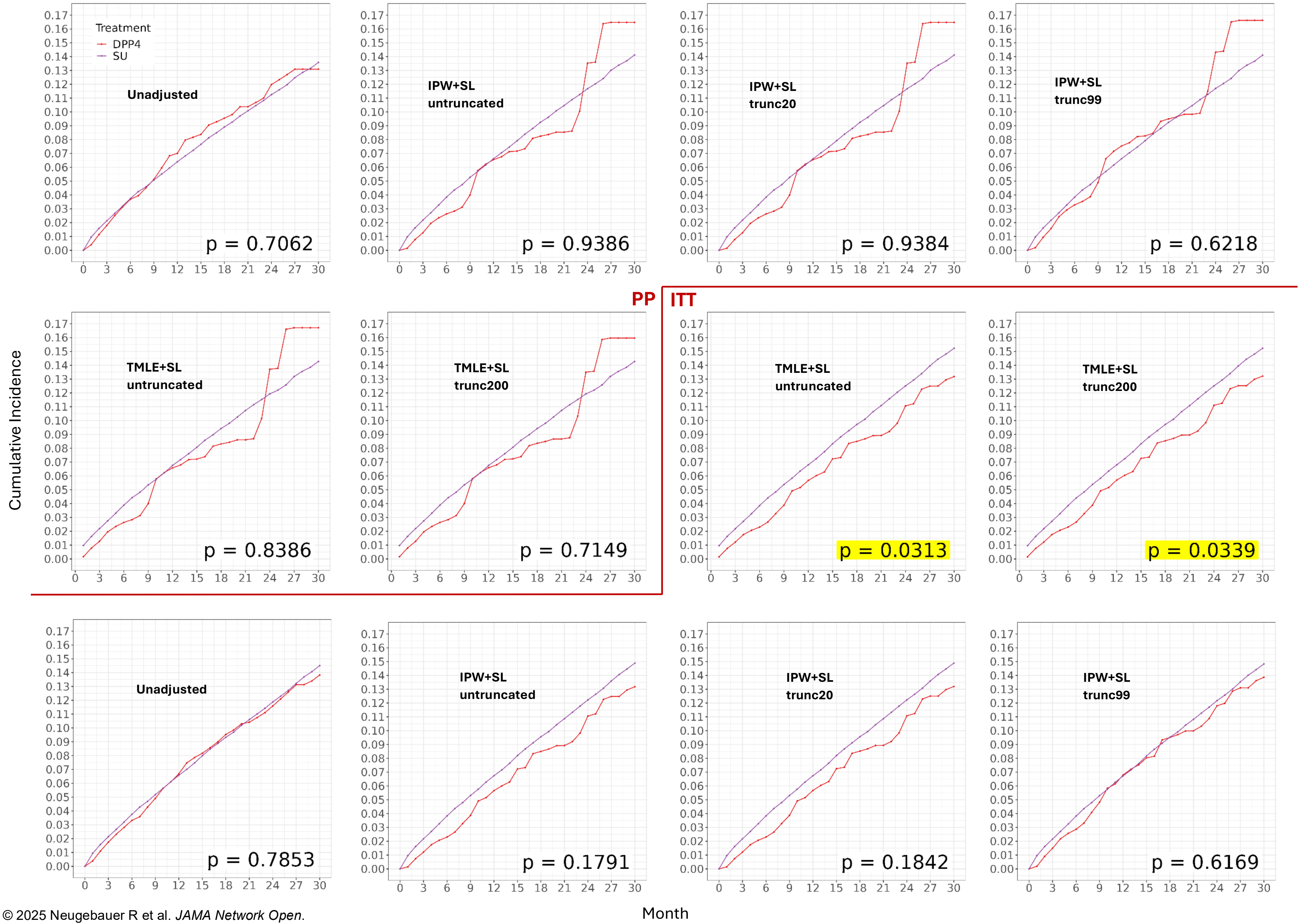

© 2025 Neugebauer R et al. *JAMA Network Open*.

eTable 11. MACE (Primary Definition), 2-Arm Drug Class Comparison, Sulfonylureas vs DPP4is, ASCVD Subgroup, RD and HR Effect Measures at 2.5 Years

Estimation results among patients with ASCVD from ITT and PP analyses of emulated 2-arm RCTs comparing MACE risks over 2.5 years between SU and DPP4i initiators. For PP analyses, rates of protocol deviations are described by medication class initiated at baseline. Unadjusted point and interval estimates and adjusted point and interval IPW and TMLE estimates of risks, risk differences (RD), and hazard ratios (HR) based on propensity scores (PS) estimated with either logistic models or super learning (SL) are presented for four weight truncation schemes along with the corresponding 99th percentile and maximum value of the stabilized and unstabilized inverse probability weights used for implementing IPW and TMLE, respectively. RD is the risk in treatment arm minus the risk in control arm and NNT is the number needed to treat.

Analysis type	Protocol Deviations* by exposure group (%)	PS estimation	99th IP weights	Max IP weight	Estimator	Treatment (DPP4i) risk in %	Control (SU) risk in %	RD [95% CI] in %	NNT	HR [95% CI]
PP	Discontinuation<br>SU: 48.45<br>DPP4i: 55.16<br><br>Crossover<br>SU: 2.31<br>DPP4i: 12.41	SL			Unadjusted	13.09	13.58	-0.49 [-3.55, 2.57]		1.03 [0.79, 1.28]
			25.82	460.98	TMLE untruncated	16.71	14.28	2.42 [-1.42, 6.26]		
					TMLE truncated at 200	15.97	14.28	1.68 [-1.60, 4.97]		
			2.00	30.78	IPW untruncated	16.48	14.12	2.36 [-5.72, 10.43]		0.80 [0.51, 1.09]
					IPW truncated at 20	16.48	14.12	2.36 [-5.72, 10.43]		0.80 [0.51, 1.09]
					IPW truncated at 99th	16.62	14.11	2.51 [-4.43, 9.45]		0.95 [0.63, 1.28]
		Logistic model	2.78	109.73	IPW untruncated	16.69	14.26	2.42 [-5.98, 10.83]		0.97 [0.55, 1.39]
					IPW truncated at 20	16.69	14.31	2.38 [-6.03, 10.79]		0.97 [0.55, 1.38]
					IPW truncated at 99th	16.99	14.21	2.78 [-4.68, 10.23]		1.07 [0.65, 1.48]
ITT		SL			Unadjusted	13.83	14.51	-0.68 [-2.92, 1.56]		1.01 [0.81, 1.20]
			25.98	1,100.46	TMLE untruncated	13.18	15.23	-2.05 [-4.50, 0.41]		
					TMLE truncated at 200	13.21	15.23	-2.01 [-4.47, 0.44]		
			1.99	49.35	IPW untruncated	13.18	14.89	-1.71 [-5.61, 2.19]		0.84 [0.57, 1.10]
					IPW truncated at 20	13.20	14.89	-1.69 [-5.60, 2.21]		0.84 [0.57, 1.11]
					IPW truncated at 99th	13.87	14.83	-0.96 [-4.41, 2.50]		0.96 [0.69, 1.24]
		Logistic model	2.47	76.45	IPW untruncated	13.60	14.78	-1.17 [-5.47, 3.12]		0.95 [0.61, 1.30]
					IPW truncated at 20	13.64	14.77	-1.13 [-5.43, 3.17]		0.95 [0.61, 1.30]
					IPW truncated at 99th	14.52	14.66	-0.15 [-3.95, 3.65]		1.04 [0.72, 1.36]

* Discontinuation refers to the interruption of the comparator medication initiated on index date; Crossover refers to the initiation of the comparator medication initiated by patient at baseline in the other arm.

© 2025 Neugebauer R et al. *JAMA Network Open*.

eFigure 8. MACE (Primary Definition), 2-Arm Drug Class Comparison, Sulfonylureas vs DPP4is, No ASCVD Subgroup, Cumulative Incidence Curves From PP and ITT Analyses With IPW, TMLE, and SL
Each plot emulates inferences among patients with No ASCVD from a 2-arm RCT comparing SU and DPP4i and represents unadjusted or adjusted estimates of cumulative incidence curves for MACE derived with IPW and TMLE with SL estimates of propensity scores with four weight truncation schemes: IPW and TMLE without weight truncation (untruncated), IPW with truncation of stabilized weights at value 20 (trunc20) or at the 99th percentile of weight values (trunc99), and TMLE with truncation of unstabilized weights at value 200 (trunc200). The red divider line separates results of Per-Protocol (PP) analyses (top half) from Intention-To-Treat (ITT) analyses (bottom half). Each plot displays a p value for the test that the average risk difference (ARD) through 2.5 years of follow-up (30 months) is 0.

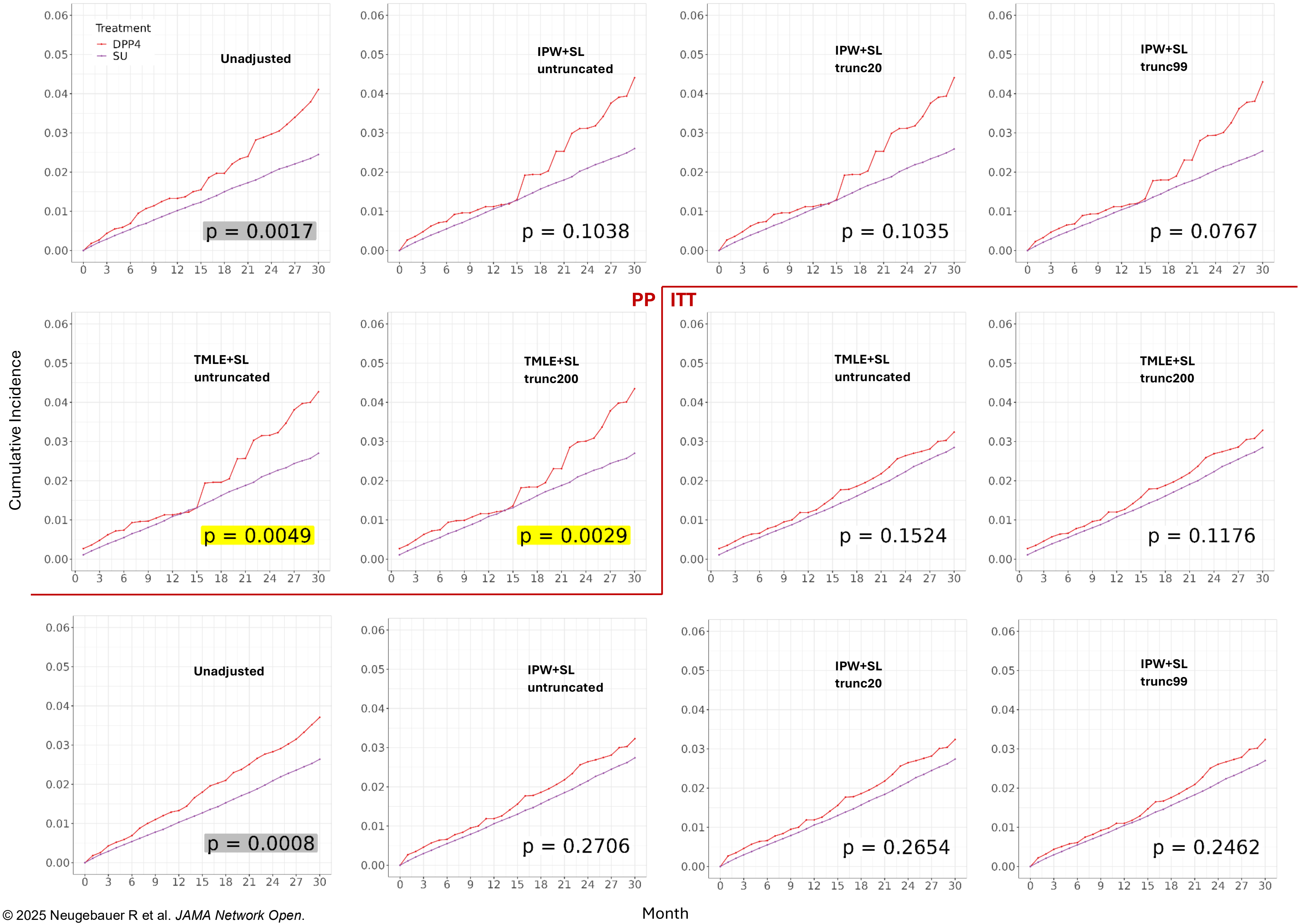

© 2025 Neugebauer R et al. *JAMA Network Open*.

eTable 12. MACE (Primary Definition), 2-Arm Drug Class Comparison, Sulfonylureas vs DPP4is, No ASCVD Subgroup, RD and HR Effect Measures at 2.5 Years

Estimation results among patients with No ASCVD from ITT and PP analyses of emulated 2-arm RCTs comparing MACE risks over 2.5 years between SU and DPP4i initiators. For PP analyses, rates of protocol deviations are described by medication class initiated at baseline. Unadjusted point and interval estimates and adjusted point and interval IPW and TM LE estimates of risks, risk differences (RD), and hazard ratios (HR) based on propensity scores (PS) estimated with either logistic models or super learning (SL) are presented for four weight truncation schemes along with the corresponding 99th percentile and maximum value of the stabilized and unstabilized inverse probability weights used for implementing IPW and TMLE, respectively. RD is the risk in treatment arm minus the risk in control arm and NNT is the number needed to treat.

Analysis type	Protocol Deviations* by exposure group (%)	PS estimation	99th IP weights	Max IP weight	Estimator	Treatment (DPP4i) risk in %	Control (SU) risk in %	RD [95% CI] in %	NNT	HR [95% CI]
PP	Discontinuation<br>SU: 49.58<br>DPP4i: 52.94<br><br>Crossover<br>SU: 2.25<br>DPP4i: 14.90	SL			Unadjusted	4.11	2.45	1.67 [0.71, 2.63]	60	1.38 [1.06, 1.7]
			19.63	5,360.78	TMLE untruncated	4.27	2.70	1.57 [0.81, 2.32]	64	
					TMLE truncated at 200	4.35	2.70	1.65 [1.03, 2.26]	61	
			2.22	193.92	IPW untruncated	4.41	2.60	1.81 [0.15, 3.47]	55	1.33 [0.77, 1.89]
					IPW truncated at 20	4.41	2.59	1.81 [0.15, 3.47]	55	1.33 [0.77, 1.89]
					IPW truncated at 99th	4.30	2.54	1.77 [0.29, 3.24]	57	1.27 [0.80, 1.73]
		Logistic model	2.94	127,932,186.00	IPW untruncated	3.35	2.46	0.90 [-1.01, 2.81]		1.30 [0.62, 1.98]
					IPW truncated at 20	4.25	2.77	1.49 [-0.51, 3.49]		1.44 [0.73, 2.15]
					IPW truncated at 99th	4.02	2.59	1.43 [0.00, 2.86]	70	1.34 [0.81, 1.87]
ITT		SL			Unadjusted	3.71	2.64	1.07 [0.49, 1.65]	94	1.40 [1.14, 1.66]
			21.40	7,477.95	TMLE untruncated	3.24	2.85	0.38 [-0.14, 0.91]		
					TMLE truncated at 200	3.29	2.85	0.44 [-0.09, 0.97]		
			1.85	134.55	IPW untruncated	3.23	2.74	0.50 [-0.37, 1.37]		1.19 [0.79, 1.59]
					IPW truncated at 20	3.24	2.74	0.51 [-0.37, 1.38]		1.19 [0.79, 1.59]
					IPW truncated at 99th	3.24	2.70	0.54 [-0.25, 1.34]		1.15 [0.82, 1.48]
		Logistic model	2.53	35,480,710.00	IPW untruncated	2.79	2.28	0.51 [-0.49, 1.50]		1.20 [0.71, 1.69]
					IPW truncated at 20	3.38	2.77	0.61 [-0.46, 1.67]		1.31 [0.80, 1.82]
					IPW truncated at 99th	3.36	2.73	0.63 [-0.26, 1.53]		1.23 [0.85, 1.62]

* Discontinuation refers to the interruption of the comparator medication initiated on index date; Crossover refers to the initiation of the comparator medication initiated by patient at baseline in the other arm.

© 2025 Neugebauer R et al. *JAMA Network Open*.

eFigure 9. MACE (Primary Definition), 2-Arm Drug Class Comparison, Sulfonylureas vs DPP4is, No ASCVD and MET Subgroup, Cumulative Incidence Curves From PP and ITT Analyses With IPW, TMLE, and SL
Each plot emulates inferences among patients with No ASCVD and MET from a 2-arm RCT comparing SU and DPP4i and represents unadjusted or adjusted estimates of cumulative incidence curves for MACE derived with IPW and TMLE with SL estimates of propensity scores with four weight truncation schemes: IPW and TMLE without weight truncation (untruncated), IPW with truncation of stabilized weights at value 20 (trunc20) or at the 99th percentile of weight values (trunc99), and TMLE with truncation of unstabilized weights at value 200 (trunc200). The red divider line separates results of Per-Protocol (PP) analyses (top half) from Intention-To-Treat (ITT) analyses (bottom half). Each plot displays a p value for the test that the average risk difference (ARD) through 2.5 years of follow-up (30 months) is 0.

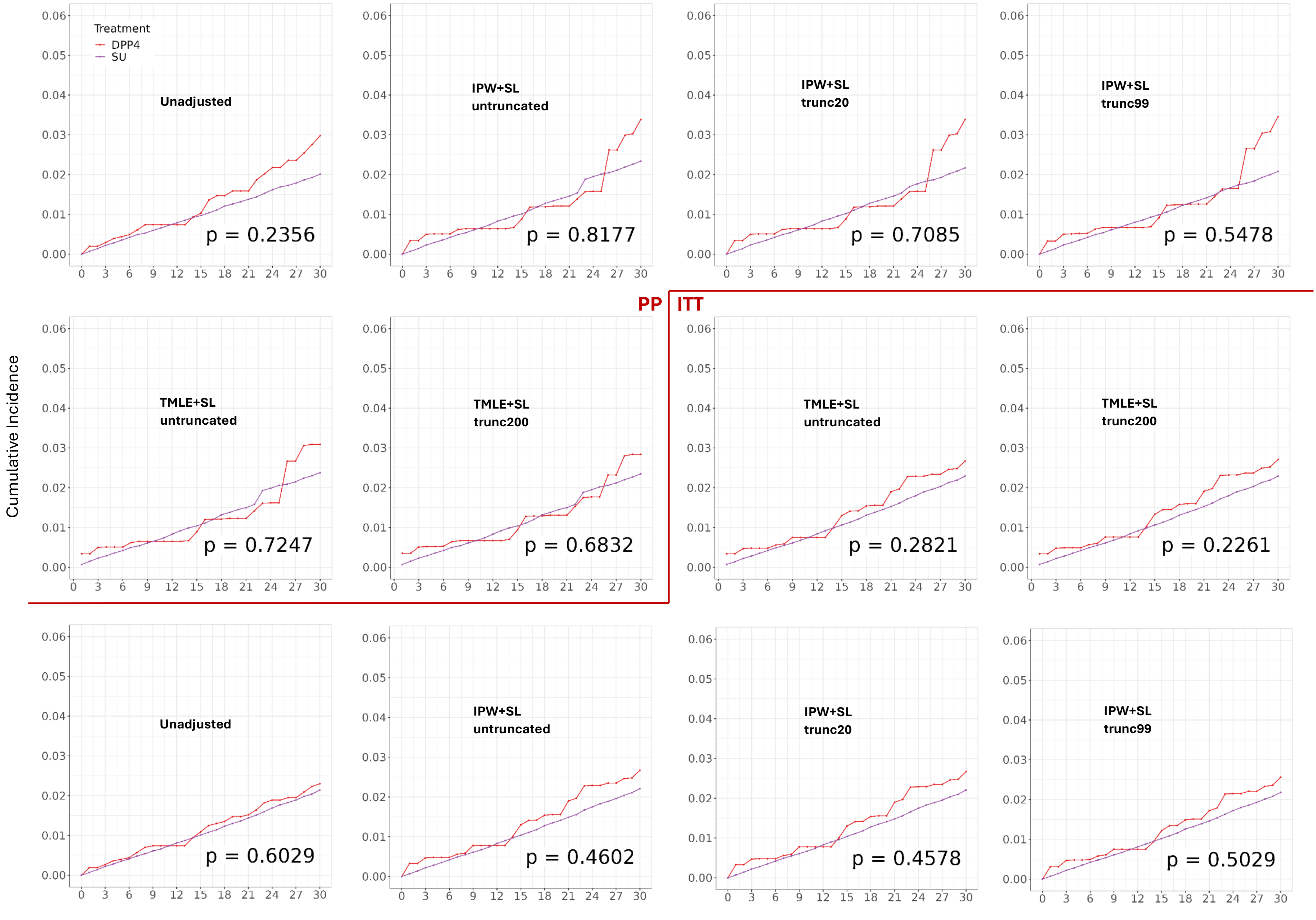

© 2025 Neugebauer R et al. *JAMA Network Open*.

eTable 13. MACE (Primary Definition), 2-Arm Drug Class Comparison, Sulfonylureas vs DPP4is, No ASCVD and MET Subgroup, RD and HR Effect Measures at 2.5 Years

Estimation results among patients with No ASCVD and MET from ITT and PP analyses of emulated 2-arm RCTs comparing MACE risks over 2.5 years between SU and DPP4i initiators. For PP analyses, rates of protocol deviations are described by medication class initiated at baseline. Unadjusted point and interval estimates and adjusted point and interval IPW and TMLE estimates of risks, risk differences (RD), and hazard ratios (HR) based on propensity scores (PS) estimated with either logistic models or super learning (SL) are presented for four weight truncation schemes along with the corresponding 99th percentile and maximum value of the stabilized and unstabilized inverse probability weights used for implementing IPW and TMLE, respectively. RD is the risk in treatment arm minus the risk in control arm and NNT is the number needed to treat.

Analysis type	Protocol Deviations* by exposure group (%)	PS estimation	99th IP weights	Max IP weight	Estimator	Treatment (DPP4i) risk in %	Control (SU) risk in %	RD [95% CI] in %	NNT	HR [95% CI]
PP	Discontinuation<br>SU: 45.34<br>DPP4i: 47.78<br><br>Crossover<br>SU: 2.54<br>DPP4i: 18.24	SL	[redacted]		Unadjusted	2.98	2.01	0.97 [-0.23, 2.17]		1.24 [0.75, 1.73]
			11.10	4,534.20	TMLE untruncated	3.09	2.38	0.71 [-0.26, 1.67]		[redacted]
					TMLE truncated at 200	2.84	2.35	0.49 [-0.18, 1.16]		
			2.18	237.43	IPW untruncated	3.39	2.34	1.05 [-1.42, 3.52]		1.03 [0.36, 1.71]
					IPW truncated at 20	3.39	2.17	1.23 [-1.20, 3.65]		1.03 [0.36, 1.71]
					IPW truncated at 99th	3.46	2.08	1.38 [-1.02, 3.77]		1.09 [0.43, 1.75]
		Logistic model	2.65	127,542,306,056.00	IPW untruncated	3.09	3.29	-0.20 [-5.47, 5.06]		133634.80 [-87874.78, 355144.38]
					IPW truncated at 20	3.09	2.20	0.89 [-0.80, 2.58]		1.03 [0.47, 1.59]
					IPW truncated at 99th	3.22	2.07	1.14 [-0.58, 2.87]		1.14 [0.53, 1.76]
ITT	[redacted]	SL	[redacted]		Unadjusted	2.30	2.14	0.16 [-0.52, 0.84]		1.09 [0.72, 1.46]
			10.90	3,744.80	TMLE untruncated	2.67	2.29	0.38 [-0.31, 1.06]		[redacted]
					TMLE truncated at 200	2.71	2.29	0.42 [-0.25, 1.09]		
			1.68	61.24	IPW untruncated	2.67	2.21	0.46 [-0.79, 1.72]		1.24 [0.56, 1.91]
					IPW truncated at 20	2.67	2.21	0.46 [-0.79, 1.72]		1.24 [0.57, 1.92]
					IPW truncated at 99th	2.56	2.18	0.38 [-0.73, 1.48]		1.17 [0.59, 1.75]
		Logistic model	2.22	62,274,677,854.00	IPW untruncated	2.81	0.00	2.81 [1.62, 4.00]	36	82250.65 [-62643.18, 227144.49]
					IPW truncated at 20	2.83	2.22	0.60 [-0.60, 1.80]		1.22 [0.60, 1.84]
					IPW truncated at 99th	2.89	2.16	0.72 [-0.46, 1.90]		1.26 [0.67, 1.84]

* Discontinuation refers to the interruption of the comparator medication initiated on index date; Crossover refers to the initiation of the comparator medication initiated by patient at baseline in the other arm.

© 2025 Neugebauer R et al. *JAMA Network Open*.

eFigure 10. MACE (Primary Definition), 2-Arm Drug Class Comparison, Sulfonylureas vs SGLT2is, CONSORT Diagram
Flow diagram describing the inclusion and exclusion steps and counts leading to the creation of the cohort for emulating the 2-arm RCT to compare the risk of MACE in new users of SU and SGLT2i along with sample sizes and counts for each observed end of follow-up type by treatment initiated at cohort entry.

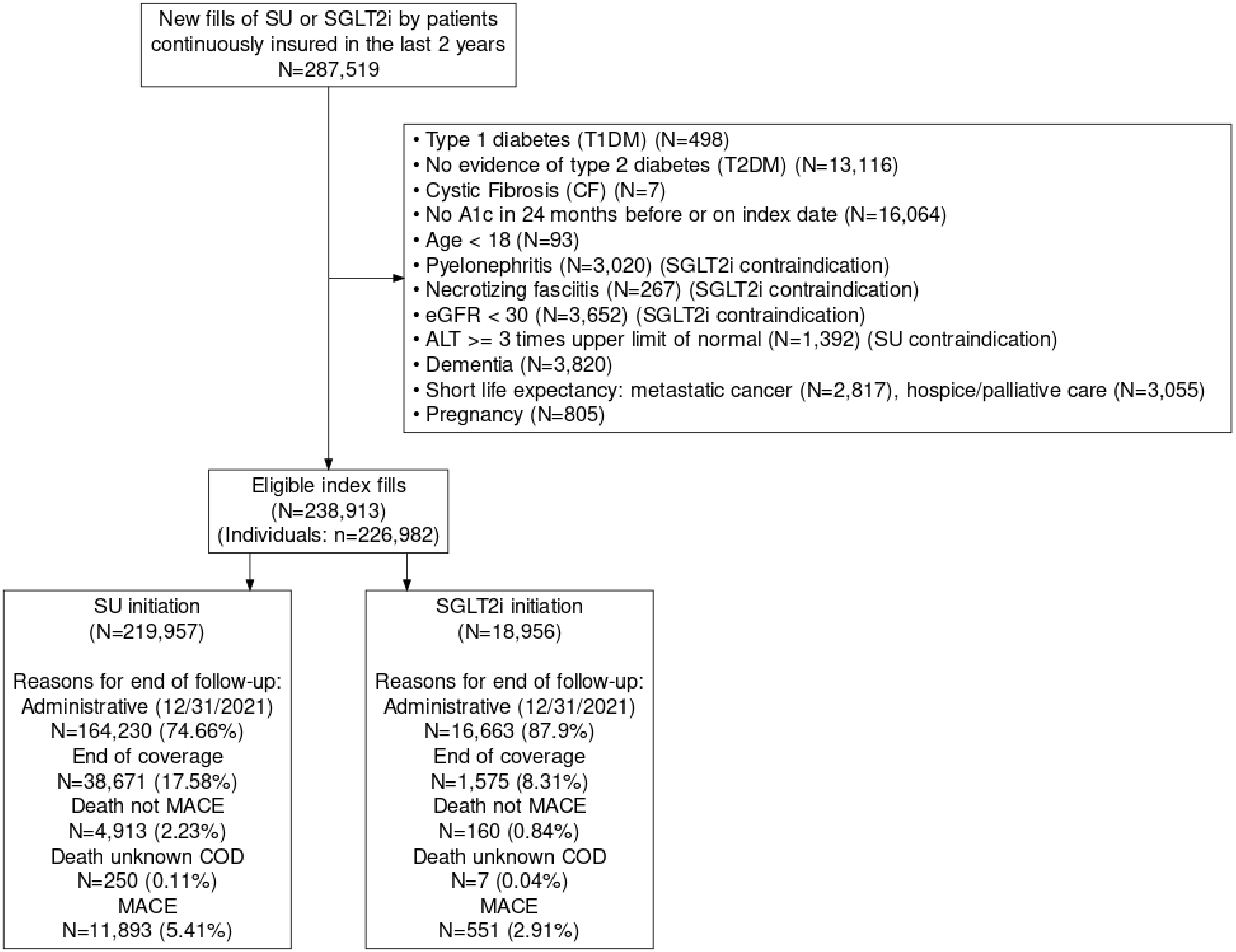

© 2025 Neugebauer R et al. *JAMA Network Open*.

eTable 14. MACE (Primary Definition), 2-Arm Drug Class Comparison, Sulfonylureas vs SGLT2is, Patient Characteristics at Baseline (Overall and by Medication Initiated)
Summary statistics of the baseline values for selected covariates in the cohort of patients used to emulate a 2-arm RCT for comparing SU and SGLT2i. For each continuous variable, the mean and standard deviation are displayed for all patients in the cohort (last column) and by drug class initiated at cohort entry. For each categorical variable and for each possible level of that variable, the count and proportion are displayed instead.

	SU<br>n = 219,957	SGLT2i<br>n = 18,956	Total<br>n = 238,913
**Demographics**			
Age	56.98 (12.92)	59.98 (11.94)	57.21 (12.87)
Agegrp			
<45	37,150 (16.89%)	2,047 (10.8%)	39,197 (16.41%)
[45-65)	120,816 (54.93%)	9,813 (51.77%)	130,629 (54.68%)
[65-75)	43,043 (19.57%)	5,217 (27.52%)	48,260 (20.2%)
>=75	18,948 (8.61%)	1,879 (9.91%)	20,827 (8.72%)
Ethnicity			
Hispanic	81,254 (36.94%)	4,183 (22.07%)	85,437 (35.76%)
Nonhispanic	138,703 (63.06%)	14,773 (77.93%)	153,476 (64.24%)
Female Head Of Hh	0.150 (0.072)	0.135 (0.071)	0.149 (0.072)
Missing	1,098 (0.499%)	717 (3.782%)	1,815 (0.76%)
Hh Public Assistance	0.039 (0.035)	0.035 (0.032)	0.039 (0.035)
Missing	1,098 (0.499%)	717 (3.782%)	1,815 (0.76%)
Household Income Less 30k	0.206 (0.119)	0.183 (0.116)	0.204 (0.119)
Missing	1,098 (0.499%)	717 (3.782%)	1,815 (0.76%)
Houspoverty	0.105 (0.089)	0.083 (0.079)	0.103 (0.089)
Missing	1,103 (0.501%)	717 (3.782%)	1,820 (0.762%)
Index Yr			
2014	26,727 (12.15%)	94 (0.5%)	26,821 (11.23%)
2015	26,583 (12.09%)	254 (1.34%)	26,837 (11.23%)
2016	28,947 (13.16%)	454 (2.4%)	29,401 (12.31%)
2017	29,224 (13.29%)	866 (4.57%)	30,090 (12.59%)
2018	26,547 (12.07%)	1,297 (6.84%)	27,844 (11.65%)
2019	27,142 (12.34%)	2,084 (10.99%)	29,226 (12.23%)
2020	24,736 (11.25%)	4,448 (23.46%)	29,184 (12.22%)
2021	30,051 (13.66%)	9,459 (49.9%)	39,510 (16.54%)
Bmi	32.80 (7.29)	34.62 (7.77)	32.94 (7.34)
Missing	6,123 (2.78%)	801 (4.23%)	6,924 (2.9%)
Smoking Status			
Formersmoker	60,071 (27.31%)	6,275 (33.1%)	66,346 (27.77%)
Currentsmoker	20,623 (9.38%)	1,719 (9.07%)	22,342 (9.35%)
Passivesmoker	1,186 (0.54%)	71 (0.37%)	1,257 (0.53%)
Neversmoker	135,497 (61.6%)	10,590 (55.87%)	146,087 (61.15%)
Unknown	2,580 (1.17%)	301 (1.59%)	2,881 (1.21%)
Low Educ	0.180 (0.134)	0.134 (0.109)	0.176 (0.132)
Missing	1,095 (0.498%)	716 (3.777%)	1,811 (0.758%)
Mgr Male	0.046 (0.045)	0.049 (0.050)	0.047 (0.045)
Missing	1,097 (0.499%)	717 (3.782%)	1,814 (0.759%)
Ndi	0.29 (0.17)	0.23 (0.14)	0.28 (0.17)
Missing	1,103 (0.5%)	717 (3.78%)	1,820 (0.76%)
Pct Crowding	0.093 (0.087)	0.071 (0.076)	0.091 (0.086)
Missing	1,098 (0.499%)	717 (3.782%)	1,815 (0.76%)
Racegrp			
White	107,998 (49.1%)	10,260 (54.13%)	118,258 (49.5%)
Asian	34,702 (15.78%)	3,182 (16.79%)	37,884 (15.86%)
Blackorafricanamerican	21,853 (9.94%)	1,839 (9.7%)	23,692 (9.92%)
Hawaiianorpacificislander	3,411 (1.55%)	452 (2.38%)	3,863 (1.62%)
Americanindianoralaskanative	1,567 (0.71%)	117 (0.62%)	1,684 (0.7%)
Multirace	6,013 (2.73%)	879 (4.64%)	6,892 (2.88%)
Other	179 (0.08%)	62 (0.33%)	241 (0.1%)
Unknown	44,234 (20.11%)	2,165 (11.42%)	46,399 (19.42%)

© 2025 Neugebauer R et al. *JAMA Network Open*.

	SU n = 219,957	SGLT2i n = 18,956	Total n = 238,913
Sex			
Female	99,325 (45.16%)	8,543 (45.07%)	107,868 (45.15%)
Male	120,626 (54.84%)	10,413 (54.93%)	131,039 (54.85%)
Other	4 (0%)	0 (0%)	4 (0%)
Unknown	2 (0%)	0 (0%)	2 (0%)
Site			
Kpnc	90,178 (41%)	6,111 (32.24%)	96,289 (40.3%)
Kpsc	114,332 (51.98%)	6,201 (32.71%)	120,533 (50.45%)
Kphi	5,542 (2.52%)	1,791 (9.45%)	7,333 (3.07%)
Hpi	3,061 (1.39%)	1,104 (5.82%)	4,165 (1.74%)
Hfhs	4,044 (1.84%)	955 (5.04%)	4,999 (2.09%)
Ghs	2,800 (1.27%)	2,794 (14.74%)	5,594 (2.34%)
Unemployment	0.050 (0.027)	0.038 (0.024)	0.049 (0.027)
Missing	1,092 (0.496%)	716 (3.777%)	1,808 (0.757%)
**Insurance**			
Ins Commercial	171,223 (77.84%)	10,850 (57.24%)	182,073 (76.21%)
Ins Highdeductible	19,966 (9.08%)	886 (4.67%)	20,852 (8.73%)
Ins Medicaid	13,198 (6%)	2,365 (12.48%)	15,563 (6.51%)
Ins Medicare	61,048 (27.75%)	7,023 (37.05%)	68,071 (28.49%)
Ins Medicare A	36,097 (16.41%)	5,050 (26.64%)	41,147 (17.22%)
Ins Medicare B	33,813 (15.37%)	4,824 (25.45%)	38,637 (16.17%)
Ins Medicare C	28,978 (13.17%)	4,345 (22.92%)	33,323 (13.95%)
Ins Medicare D	31,482 (14.31%)	4,683 (24.7%)	36,165 (15.14%)
Ins Other Coverage	48,762 (22.17%)	3,712 (19.58%)	52,474 (21.96%)
Ins Privatepay	40,927 (18.61%)	2,553 (13.47%)	43,480 (18.2%)
Ins Selffunded	4,045 (1.84%)	708 (3.73%)	4,753 (1.99%)
Ins Statesubsidized	782 (0.36%)	900 (4.75%)	1,682 (0.7%)
**Clinical data**			
A1c Age	42.34 (78.40)	50.77 (81.37)	43.00 (78.67)
Missing	237 (0.11%)	23 (0.12%)	260 (0.11%)
Acc Aha 201310yrcvdrisk	0.168 (0.151)	0.196 (0.156)	0.170 (0.151)
Missing	26,062 (11.849%)	2,487 (13.12%)	28,549 (11.95%)
Chf Dx Status			
0	210,871 (95.87%)	15,993 (84.37%)	226,864 (94.96%)
1	8,221 (3.74%)	2,756 (14.54%)	10,977 (4.59%)
999	865 (0.39%)	207 (1.09%)	1,072 (0.45%)
Cv Risk Subgrp			
Low	85,264 (38.76%)	5,504 (29.04%)	90,768 (37.99%)
Moderate	44,915 (20.42%)	3,834 (20.23%)	48,749 (20.4%)
High	59,404 (27.01%)	6,074 (32.04%)	65,478 (27.41%)
Other	4,796 (2.18%)	1,154 (6.09%)	5,950 (2.49%)
Unknown	25,578 (11.63%)	2,390 (12.61%)	27,968 (11.71%)
Diab Duration	4.90 (3.22)	7.65 (3.84)	5.12 (3.36)
Missing	364 (0.17%)	1 (0.01%)	365 (0.15%)
A1c	9.23 (2.08)	8.60 (1.71)	9.18 (2.06)
Missing	237 (0.11%)	23 (0.12%)	260 (0.11%)
Acr	46.12 (82.91)	80.33 (116.07)	48.85 (86.52)
Missing	43,595 (19.82%)	3,676 (19.39%)	47,271 (19.79%)
Afib Dx	8,794 (4%)	1,751 (9.24%)	10,545 (4.41%)
Alt	33.28 (22.19)	30.15 (18.52)	33.03 (21.94)
Missing	45,974 (20.9%)	4,228 (22.3%)	50,202 (21.01%)
Amputation Dxpx	1,230 (0.56%)	338 (1.78%)	1,568 (0.66%)
Anemia Dx	13,215 (6.01%)	2,287 (12.06%)	15,502 (6.49%)
Anxiety Dx	38,151 (17.34%)	4,406 (23.24%)	42,557 (17.81%)
Arrythmia Dx	7,828 (3.56%)	1,731 (9.13%)	9,559 (4%)
Ascvd Dxpx Max	20,578 (9.36%)	3,805 (20.07%)	24,383 (10.21%)
Ascvd Dxpx Ppv	4,796 (2.18%)	1,154 (6.09%)	5,950 (2.49%)

© 2025 Neugebauer R et al. *JAMA Network Open*.

	SU<br>n = 219,957	SGLT2i<br>n = 18,956	Total<br>n = 238,913
Asthma Dx	22,282 (10.13%)	2,624 (13.84%)	24,906 (10.42%)
Bariatric Px	2,211 (1.01%)	293 (1.55%)	2,504 (1.05%)
Bipolar Dx	2,006 (0.91%)	259 (1.37%)	2,265 (0.95%)
Blind Dx	755 (0.34%)	52 (0.27%)	807 (0.34%)
Cad Dxpx Max	12,790 (5.81%)	2,628 (13.86%)	15,418 (6.45%)
Cad Dxpx Ppv	2,916 (1.33%)	884 (4.66%)	3,800 (1.59%)
Cad Dxpx Sens	3,292 (1.5%)	969 (5.11%)	4,261 (1.78%)
Cancer Mets Dx	0 (0%)	0 (0%)	0 (0%)
Cancer Nomets Dx	8,214 (3.73%)	1,151 (6.07%)	9,365 (3.92%)
Cevd Dxpx Ppv	1,832 (0.83%)	243 (1.28%)	2,075 (0.87%)
Cevd Dxpx Sens	6,770 (3.08%)	1,157 (6.1%)	7,927 (3.32%)
Chf Dx Ppv	1,848 (0.84%)	719 (3.79%)	2,567 (1.07%)
Chf Dx Sens	8,221 (3.74%)	2,756 (14.54%)	10,977 (4.59%)
Ckd Dx	43,800 (19.91%)	7,067 (37.28%)	50,867 (21.29%)
Copd Dx	7,854 (3.57%)	1,324 (6.98%)	9,178 (3.84%)
Coupled Dbp	75.00 (10.74)	72.91 (11.54)	74.83 (10.82)
Missing	3,927 (1.79%)	466 (2.46%)	4,393 (1.84%)
Coupled Sbp	129.45 (14.49)	130.31 (15.56)	129.52 (14.58)
Missing	3,927 (1.79%)	466 (2.46%)	4,393 (1.84%)
Covid Prd	54,787 (24.91%)	13,907 (73.36%)	68,694 (28.75%)
Creat	0.88 (0.27)	0.97 (0.31)	0.89 (0.27)
Missing	6,047 (2.75%)	363 (1.91%)	6,410 (2.68%)
Cysticfibrosis Dx	0 (0%)	0 (0%)	0 (0%)
Dbp	74.15 (10.73)	72.04 (11.65)	73.99 (10.82)
Missing	3,924 (1.78%)	466 (2.46%)	4,390 (1.84%)
Dementia Dx	0 (0%)	0 (0%)	0 (0%)
Depr Dx	28,988 (13.18%)	3,285 (17.33%)	32,273 (13.51%)
Dietitian	10,091 (4.59%)	1,656 (8.74%)	11,747 (4.92%)
Dka Dx	3,049 (1.39%)	314 (1.66%)	3,363 (1.41%)
Dka Dx Count	0.01 (0.13)	0.02 (0.19)	0.02 (0.14)
Esrd Dx	8,808 (4%)	2,292 (12.09%)	11,100 (4.65%)
Esrd Px	452 (0.21%)	63 (0.33%)	515 (0.22%)
Etoh Dx	4,082 (1.86%)	451 (2.38%)	4,533 (1.9%)
Fasciitis Dx	0 (0%)	0 (0%)	0 (0%)
Fpg	181.18 (73.82)	168.78 (69.57)	180.67 (73.69)
Missing	144,784 (65.82%)	15,745 (83.06%)	160,529 (67.19%)
Frailty Dx	14,133 (6.43%)	2,370 (12.5%)	16,503 (6.91%)
Gfr Epi 09	88.79 (21.72)	80.64 (22.75)	88.14 (21.92)
Missing	6,047 (2.75%)	363 (1.91%)	6,410 (2.68%)
Hdl	44.52 (11.49)	43.37 (11.65)	44.43 (11.50)
Missing	30,853 (14.03%)	2,588 (13.65%)	33,441 (14%)
Hgb	14.03 (1.62)	13.70 (1.61)	14.00 (1.62)
Missing	61,152 (27.8%)	4,639 (24.47%)	65,791 (27.54%)
Htn Dx	119,798 (54.46%)	13,703 (72.29%)	133,501 (55.88%)
Hypo Dx	164 (0.07%)	79 (0.42%)	243 (0.1%)
Hypo Dx Count	0.01 (0.09)	0.03 (0.26)	0.01 (0.12)
Hypo Dx Event	164 (0.07%)	79 (0.42%)	243 (0.1%)
Hypothyroidism Dx	21,004 (9.55%)	2,623 (13.84%)	23,627 (9.89%)
Ldl	96.33 (39.05)	84.92 (36.96)	95.43 (39.01)
Missing	24,462 (11.12%)	2,233 (11.78%)	26,695 (11.17%)
Leukemia Lymphoma Dx	1,271 (0.58%)	145 (0.76%)	1,416 (0.59%)
Lipid Dx	130,414 (59.29%)	13,693 (72.24%)	144,107 (60.32%)
Liver Dx	373 (0.17%)	43 (0.23%)	416 (0.17%)
Mci Dx	1,003 (0.46%)	117 (0.62%)	1,120 (0.47%)
Men2 Dx	2 (0%)	0 (0%)	2 (0%)
Nephropathy Dx	12,413 (5.64%)	654 (3.45%)	13,067 (5.47%)
Neuro Dx	5,077 (2.31%)	742 (3.91%)	5,819 (2.44%)

© 2025 Neugebauer R et al. *JAMA Network Open*.

	SU<br>n = 219,957	SGLT2i<br>n = 18,956	Total<br>n = 238,913
Pancreatitis Dx	4,041 (1.84%)	565 (2.98%)	4,606 (1.93%)
Pcr	0.000 (0.000)	0.000 (0.000)	0.000 (0.000)
Missing	206,277 (93.781%)	16,319 (86.089%)	222,596 (93.17%)
Potassium	4.23 (0.40)	4.29 (0.42)	4.24 (0.40)
Missing	17,659 (8.03%)	765 (4.04%)	18,424 (7.71%)
Pregnancy	0 (0%)	0 (0%)	0 (0%)
Pud Dx	228 (0.1%)	26 (0.14%)	254 (0.11%)
Pvd Dxpx Ppv	404 (0.18%)	79 (0.42%)	483 (0.2%)
Pvd Dxpx Sens	3,853 (1.75%)	779 (4.11%)	4,632 (1.94%)
Pyelo Dx	0 (0%)	0 (0%)	0 (0%)
Retinopathy Dxpx	6,817 (3.1%)	967 (5.1%)	7,784 (3.26%)
Rpg	215.68 (113.41)	191.81 (88.71)	213.01 (111.18)
Missing	141,406 (64.29%)	9,093 (47.97%)	150,499 (62.99%)
Sbp	128.66 (14.32)	129.47 (15.59)	128.73 (14.43)
Missing	3,927 (1.79%)	466 (2.46%)	4,393 (1.84%)
Schiz Dx	1,355 (0.62%)	112 (0.59%)	1,467 (0.61%)
Sodium	138.04 (3.06)	138.84 (2.96)	138.11 (3.06)
Missing	50,097 (22.78%)	2,895 (15.27%)	52,992 (22.18%)
Sud Dx	2,184 (0.99%)	239 (1.26%)	2,423 (1.01%)
Tc	177.27 (52.91)	162.88 (48.88)	176.12 (52.75)
Missing	29,971 (13.63%)	2,560 (13.5%)	32,531 (13.62%)
Trig	215.99 (217.63)	200.71 (173.02)	214.80 (214.53)
Missing	41,627 (18.93%)	3,904 (20.6%)	45,531 (19.06%)
Tsh	2.04 (1.91)	2.17 (2.04)	2.05 (1.92)
Missing	79,573 (36.18%)	7,163 (37.79%)	86,736 (36.3%)
Valvular Dx	3,592 (1.63%)	891 (4.7%)	4,483 (1.88%)
Vasculitis Dx	2,669 (1.21%)	208 (1.1%)	2,877 (1.2%)
Only Met No Ascvd	124,039 (56.39%)	5,362 (28.29%)	129,401 (54.16%)
Renal Function Status			
Lowrisk	114,010 (51.83%)	7,561 (39.89%)	121,571 (50.89%)
Moderaterisk	42,602 (19.37%)	4,347 (22.93%)	46,949 (19.65%)
Highrisk	14,052 (6.39%)	2,252 (11.88%)	16,304 (6.82%)
Veryhighrisk	4,418 (2.01%)	1,074 (5.67%)	5,492 (2.3%)
Unknown	44,875 (20.4%)	3,722 (19.63%)	48,597 (20.34%)
Total Visit C	0.44 (1.77)	1.39 (3.78)	0.51 (2.02)
Total Visit E	0.15 (1.15)	0.73 (2.77)	0.20 (1.36)
Total Visit N	0.11 (1.77)	0.38 (1.88)	0.13 (1.78)
**Concurrent medications**			
Aa	2 (0%)	1 (0.01%)	3 (0%)
Aceinhibitors	76,858 (34.94%)	7,608 (40.14%)	84,466 (35.35%)
Agi	235 (0.11%)	65 (0.34%)	300 (0.13%)
Anticoagulants	7,225 (3.28%)	1,621 (8.55%)	8,846 (3.7%)
Anticonvulsants	12,911 (5.87%)	2,694 (14.21%)	15,605 (6.53%)
Antidepressantcomb	3 (0%)	0 (0%)	3 (0%)
Antidepressantmaoi	23 (0.01%)	5 (0.03%)	28 (0.01%)
Antidepressantndri	3,826 (1.74%)	567 (2.99%)	4,393 (1.84%)
Antidepressantother	0 (0%)	1 (0.01%)	1 (0%)
Antidepressantsari	5,439 (2.47%)	735 (3.88%)	6,174 (2.58%)
Antidepressantsnri	5,948 (2.7%)	1,189 (6.27%)	7,137 (2.99%)
Antidepressantspo	64 (0.03%)	28 (0.15%)	92 (0.04%)
Antidepressantssri	17,905 (8.14%)	2,287 (12.06%)	20,192 (8.45%)
Antidepressanttca	5,830 (2.65%)	584 (3.08%)	6,414 (2.68%)
Antidepressantteca	1,247 (0.57%)	164 (0.87%)	1,411 (0.59%)
Antiplatelets	12,094 (5.5%)	2,707 (14.28%)	14,801 (6.2%)
Antipsychotic1stgen	473 (0.22%)	40 (0.21%)	513 (0.21%)
Antipsychotic2ndgen	3,286 (1.49%)	397 (2.09%)	3,683 (1.54%)
Anxiety	2,446 (1.11%)	422 (2.23%)	2,868 (1.2%)

© 2025 Neugebauer R et al. *JAMA Network Open*.

	SU<br>n = 219,957	SGLT2i<br>n = 18,956	Total<br>n = 238,913
Arb	36,024 (16.38%)	5,703 (30.09%)	41,727 (17.47%)
Benzodiazepines	8,058 (3.66%)	772 (4.07%)	8,830 (3.7%)
Betablockers	46,874 (21.31%)	7,488 (39.5%)	54,362 (22.75%)
Clonidine	1,846 (0.84%)	243 (1.28%)	2,089 (0.87%)
Dihydropyridineccb	28,371 (12.9%)	3,934 (20.75%)	32,305 (13.52%)
Dpp4	1,884 (0.86%)	1,158 (6.11%)	3,042 (1.27%)
Glp1	654 (0.3%)	1,516 (8%)	2,170 (0.91%)
Hypnoticother	1,676 (0.76%)	176 (0.93%)	1,852 (0.78%)
Injectableantipsychotic	12 (0.01%)	6 (0.03%)	18 (0.01%)
Ins	16,506 (7.5%)	8,700 (45.9%)	25,206 (10.55%)
Ins Analog	3,452 (1.57%)	3,291 (17.36%)	6,743 (2.82%)
Ins Combo	1,505 (0.68%)	1,424 (7.51%)	2,929 (1.23%)
Ins Human	13,871 (6.31%)	6,282 (33.14%)	20,153 (8.44%)
Ins La	14,361 (6.53%)	6,560 (34.61%)	20,921 (8.76%)
Ins Sa	4,299 (1.95%)	4,779 (25.21%)	9,078 (3.8%)
Ksparingdiuretics	5,628 (2.56%)	1,469 (7.75%)	7,097 (2.97%)
Lithium	343 (0.16%)	31 (0.16%)	374 (0.16%)
Loopdiuretics	9,535 (4.33%)	2,781 (14.67%)	12,316 (5.16%)
Meg	159 (0.07%)	118 (0.62%)	277 (0.12%)
Met	149,311 (67.88%)	13,374 (70.55%)	162,685 (68.09%)
Nondihydropyridineccb	3,475 (1.58%)	450 (2.37%)	3,925 (1.64%)
Otherlipidmeds	5,953 (2.71%)	916 (4.83%)	6,869 (2.88%)
Pcsk9mab	26 (0.01%)	35 (0.18%)	61 (0.03%)
Statins	123,227 (56.02%)	14,527 (76.64%)	137,754 (57.66%)
Stimulants	888 (0.4%)	114 (0.6%)	1,002 (0.42%)
Suold	1,500 (0.68%)	0 (0%)	1,500 (0.63%)
Thiazidediuretics	50,390 (22.91%)	4,569 (24.1%)	54,959 (23%)
Tir	0 (0%)	0 (0%)	0 (0%)
Tzd	1,897 (0.86%)	451 (2.38%)	2,348 (0.98%)
Only Met Therapy	134,873 (61.32%)	6,364 (33.57%)	141,237 (59.12%)

© 2025 Neugebauer R et al. *JAMA Network Open*.

eFigure 11. MACE (Primary Definition), 2-Arm Drug Class Comparison, Sulfonylureas vs SGLT2is, Cumulative Incidence Curves From PP and ITT Analyses With IPW, TMLE, and SL
Each plot emulates inferences from a 2-arm RCT comparing SU and SGLT2i and represents unadjusted or adjusted estimates of cumulative incidence curves for MACE derived with inverse probability weighting (IPW) and Targeted Minimum Loss-based Estimation (TMLE) with Super Learning (SL) estimates of propensity scores with four weight truncation schemes: IPW and TMLE without weight truncation (untruncated), IPW with truncation of stabilized weights at value 20 (trunc20) or at the 99th percentile of weight values (trunc99), and TMLE with truncation of unstabilized weights at value 200 (trunc200). The red divider line separates results of Per-Protocol (PP) analyses (top half) from Intention-To-Treat (ITT) analyses (bottom half). Each plot displays a p value for the test that the average risk difference (ARD) through 2.5 years of follow-up (30 months) is 0.

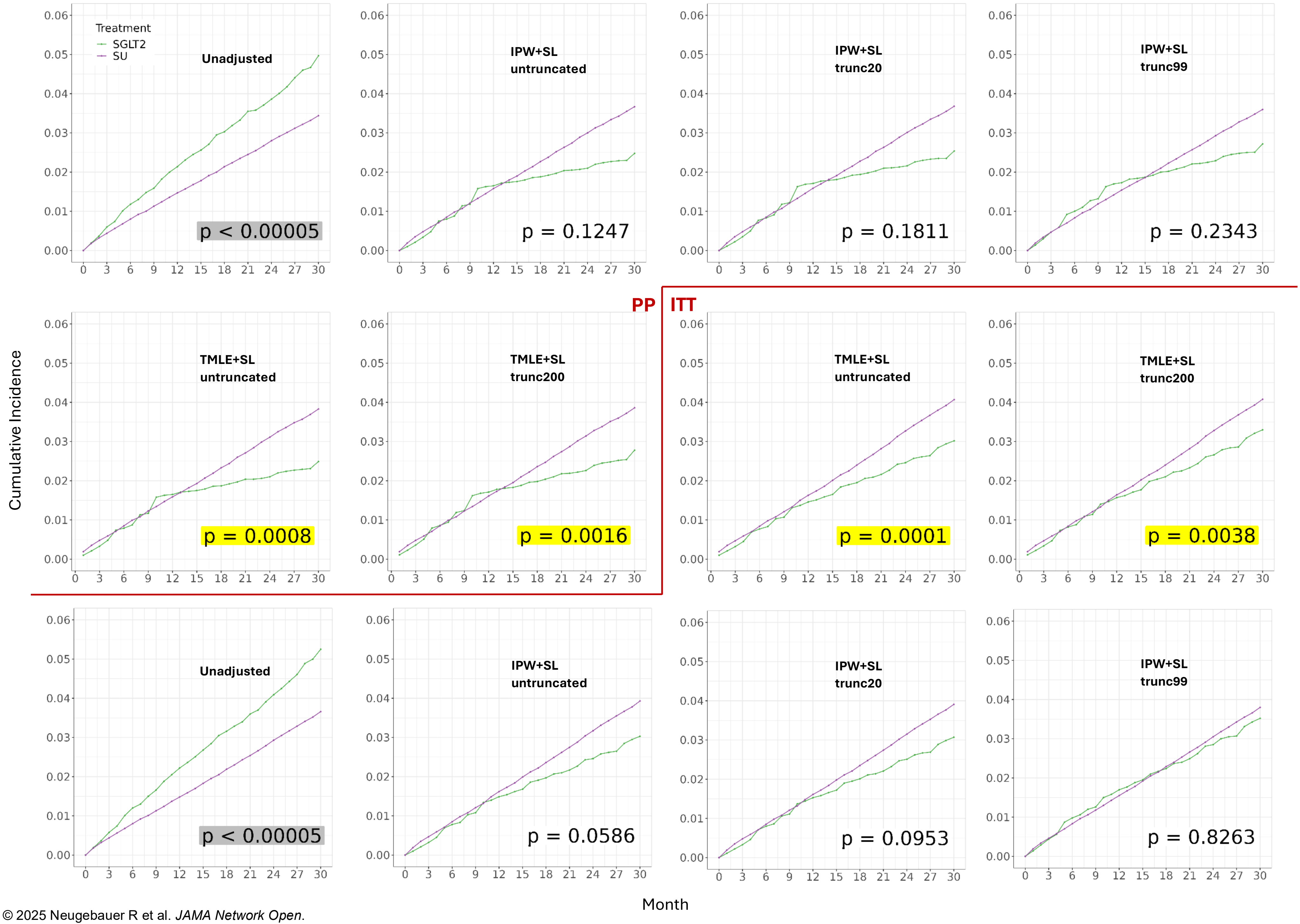

© 2025 Neugebauer R et al. *JAMA Network Open*.

eFigure 12. MACE (Primary Definition), 2-Arm Drug Class Comparison, Sulfonylureas vs SGLT2is, Cumulative Incidence Curves From Sensitivity PP Analyses With IPW, TMLE, and SL
Each plot emulates inferences from a 2-arm RCT comparing SU and SGLT2i and represents unadjusted or adjusted estimates of cumulative incidence curves for MACE from sensitivity PP analyses referred to as "NoMBS PP" and "No3 PP". NoMBS PP analyses are restricted to patients without a history of MBS at baseline and the protocols they evaluate preclude metabolic bariatric surgery (MBS) procedures. The protocols in the No3 PP analyses preclude exposure to three medication classes: the comparator medication from the other arm, DPP4i and GLP-1RA.

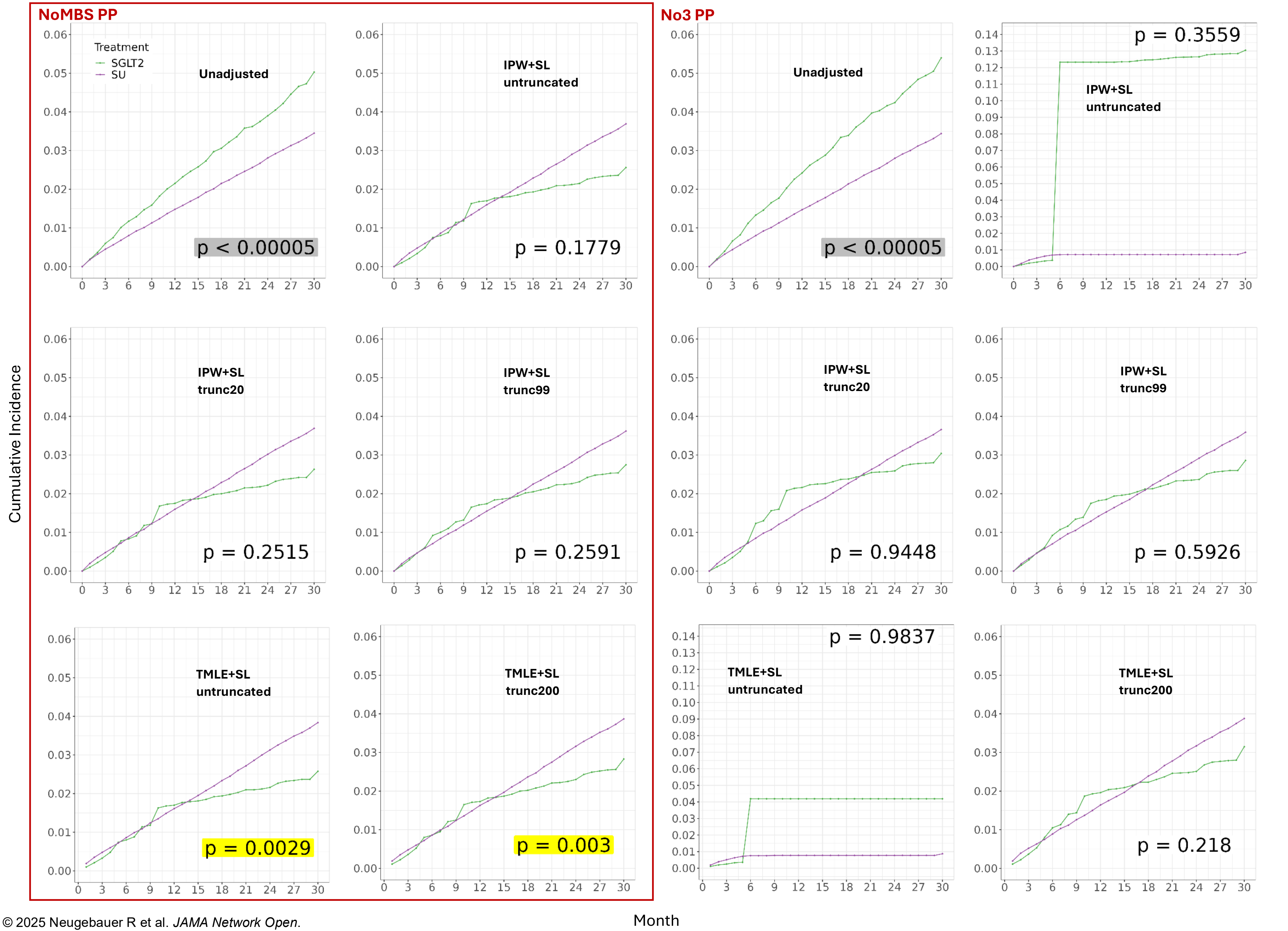

© 2025 Neugebauer R et al. *JAMA Network Open*.

eTable 15. MACE (Primary Definition), 2-Arm Drug Class Comparison, Sulfonylureas vs SGLT2is, RD and HR Effect Measures at 2.5 Years

Estimation results from ITT, PP, NoMBS PP, and No3 PP analyses of emulated 2-arm RCTs comparing MACE risks over 2.5 years between SU and SGLT2i initiators. For PP analyses, rates of protocol deviations are described by medication class initiated at baseline. Unadjusted point and interval estimates and adjusted point and interval IPW and TMLE estimates of risks, risk differences (RD), and hazard ratios (HR) based on propensity scores (PS) estimated with either logistic models or super learning (SL) are presented for four weight truncation schemes along with the corresponding 99th percentile and maximum value of the stabilized and unstabilized inverse probability weights used for implementing IPW and TMLE, respectively. RD is the risk in treatment arm minus the risk in control arm and NNT is the number needed to treat.

Analysis type	Protocol Deviations* by exposure group (%)	PS estimation	99th IP weights	Max IP weight	Estimator	Treatment (SU) risk in %	Control (SGLT2i) risk in %	RD [95% CI] in %	NNT	HR [95% CI]
PP	Discontinuation<br>SU: 49.59<br>SGLT2i: 27.04<br><br>Crossover<br>SU: 4.43<br>SGLT2i: 5.85	SL			Unadjusted	3.44	4.97	-1.52 [-2.26, -0.78]	66	0.70 [0.61, 0.78]
			15.41	3,833.93	TMLE untruncated	3.83	2.49	1.34 [0.96, 1.73]	75	
					TMLE truncated at 200	3.86	2.78	1.08 [0.75, 1.40]	93	
			2.71	1,192.00	IPW untruncated	3.67	2.48	1.18 [0.40, 1.97]	84	1.05 [0.71, 1.38]
					IPW truncated at 20	3.68	2.54	1.13 [0.34, 1.93]	88	1.01 [0.69, 1.34]
					IPW truncated at 99th	3.60	2.72	0.88 [0.30, 1.47]	113	0.90 [0.71, 1.10]
		Logistic model	3.50	2,246,858,183	IPW untruncated	3.39	2.58	0.81 [-0.51, 2.12]		0.20 [0.07, 0.33]
					IPW truncated at 20	3.89	2.73	1.16 [0.03, 2.28]	87	0.90 [0.53, 1.28]
					IPW truncated at 99th	3.74	2.78	0.97 [0.31, 1.62]	104	0.86 [0.65, 1.06]
ITT		SL			Unadjusted	3.66	5.25	-1.59 [-2.14, -1.04]	63	0.68 [0.60, 0.76]
			12.15	1,581.40	TMLE untruncated	4.07	3.02	1.05 [0.69, 1.41]	95	
					TMLE truncated at 200	4.08	3.30	0.78 [0.43, 1.13]	128	
			2.39	1,236.32	IPW untruncated	3.93	3.03	0.90 [0.15, 1.64]	112	1.13 [0.82, 1.44]
					IPW truncated at 20	3.91	3.07	0.84 [0.09, 1.59]	119	1.10 [0.80, 1.40]
					IPW truncated at 99th	3.80	3.52	0.28 [-0.44, 1.01]		0.93 [0.75, 1.11]
		Logistic model	3.14	1,726,161,611	IPW untruncated	0.85	3.63	-2.78 [-4.65, -0.91]	36	0.11 [0.02, 0.20]
					IPW truncated at 20	4.02	3.79	0.22 [-1.21, 1.66]		1.02 [0.68, 1.37]
					IPW truncated at 99th	3.86	3.67	0.19 [-0.60, 0.99]		0.90 [0.71, 1.08]
NoMBS PP	Discontinuation<br>SU: 49.24<br>SGLT2i: 26.79<br><br>Crossover<br>SU: 4.83<br>SGLT2i: 6.14<br><br>MBS occurrence<br>SU: 0.42<br>SGLT2i: 0.33	SL			Unadjusted	3.45	5.03	-1.57 [-2.32, -0.82]	64	0.70 [0.61, 0.79]
			14.98	3,299.49	TMLE untruncated	3.84	2.58	1.26 [0.86, 1.66]	79	
					TMLE truncated at 200	3.87	2.83	1.04 [0.71, 1.36]	97	
			2.68	1,318.89	IPW untruncated	3.69	2.56	1.12 [0.29, 1.96]	89	1.04 [0.70, 1.38]
					IPW truncated at 20	3.69	2.63	1.06 [0.21, 1.92]	94	1.01 [0.68, 1.34]
					IPW truncated at 99th	3.62	2.75	0.86 [0.26, 1.47]	116	0.90 [0.70, 1.10]
		Logistic model	3.46	110,220,533	IPW untruncated	2.90	2.69	0.21 [-1.15, 1.57]		0.27 [0.10, 0.44]
					IPW truncated at 20	3.90	2.84	1.06 [-0.14, 2.26]		0.89 [0.51, 1.27]
					IPW truncated at 99th	3.75	2.86	0.90 [0.20, 1.59]	112	0.86 [0.65, 1.07]
No3 PP	Discontinuation<br>SU: 47.72<br>SGLT2i: 19.45<br><br>Crossover to comparator drug<br>SU: 3.83<br>SGLT2i: 4.58<br><br>Initiation of one of the two non-comparator drugs<br>SU: 4.52<br>SGLT2i: 20.42	SL			Unadjusted	3.44	5.40	-1.96 [-2.88, -1.04]	51	0.64 [0.55, 0.73]
			14.46	1.72e+26	TMLE untruncated	0.87	0.36	0.51 [-1378.76, 1379.78]		
					TMLE truncated at 200	3.88	3.15	0.73 [0.36, 1.10]	138	
			2.50	4.61e+25	IPW untruncated	0.85	13.05	-12.21 [-38.35, 13.94]		0.02 [-0.03, 0.06]
					IPW truncated at 20	3.66	3.04	0.62 [-0.56, 1.81]		0.77 [0.40, 1.13]
					IPW truncated at 99th	3.59	2.86	0.73 [0.04, 1.42]	137	0.82 [0.62, 1.02]
		Logistic model	3.17	9.51e+48	IPW untruncated	0.66	31.68	-31.02 [-89.35, 27.31]		0 [0, 0]
					IPW truncated at 20	3.87	3.29	0.58 [-0.86, 2.02]		0.68 [0.35, 1.01]
					IPW truncated at 99th	3.72	3.01	0.71 [-0.06, 1.47]		0.74 [0.55, 0.94]

* Discontinuation refers to the interruption of the comparator medication initiated on index date; Crossover refers to the initiation of the comparator medication initiated by patient at baseline in the other arm; MBS occurrence refers to patient's undergoing metabolic bariatric surgery (MBS).

© 2025 Neugebauer R et al. *JAMA Network Open*.

eFigure 13. MACE (Primary Definition), 2-Arm Drug Class Comparison, Sulfonylureas vs SGLT2is, ASCVD Subgroup, Cumulative Incidence Curves From PP and ITT Analyses With IPW, TMLE, and SL
Each plot emulates inferences among patients with ASCVD from a 2-arm RCT comparing SU and SGLT2i and represents unadjusted or adjusted estimates of cumulative incidence curves for MACE derived with IPW and TMLE with SL estimates of propensity scores with four weight truncation schemes: IPW and TMLE without weight truncation (untruncated), IPW with truncation of stabilized weights at value 20 (trunc20) or at the 99th percentile of weight values (trunc99), and TMLE with truncation of unstabilized weights at value 200 (trunc200). The red divider line separates results of Per-Protocol (PP) analyses (top half) from Intention-To-Treat (ITT) analyses (bottom half). Each plot displays a p value for the test that the average risk difference (ARD) through 2.5 years of follow-up (30 months) is 0.

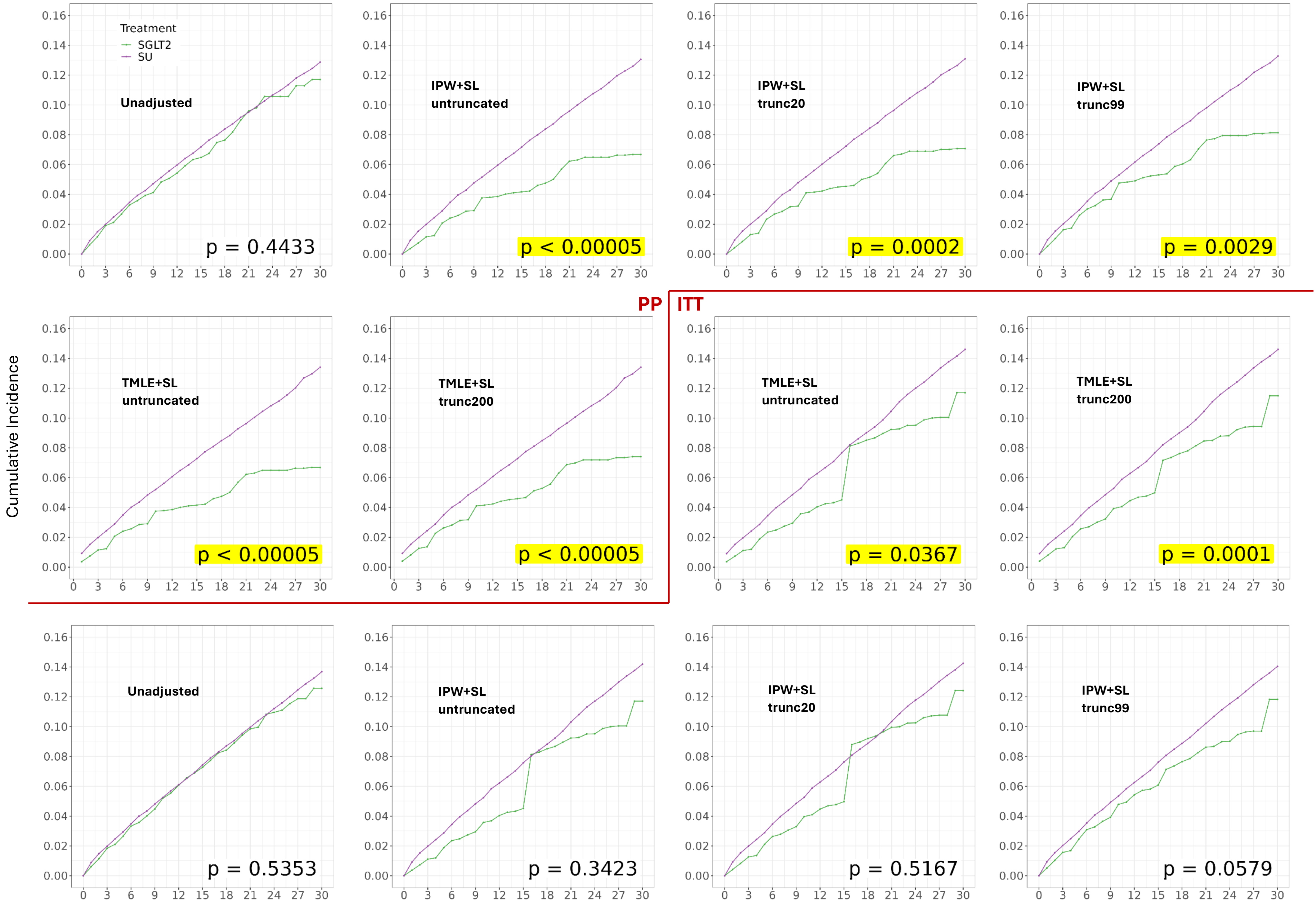

© 2025 Neugebauer R et al. *JAMA Network Open*.

eTable 16. MACE (Primary Definition), 2-Arm Drug Class Comparison, Sulfonylureas vs SGLT2is, ASCVD Subgroup, RD and HR Effect Measures at 2.5 Years

Estimation results among patients with ASCVD from ITT and PP analyses of emulated 2-arm RCTs comparing MACE risks over 2.5 years between SU and SGLT2i initiators. For PP analyses, rates of protocol deviations are described by medication class initiated at baseline. Unadjusted point and interval estimates and adjusted point and interval IPW and TMLE estimates of risks, risk differences (RD), and hazard ratios (HR) based on propensity scores (PS) estimated with either logistic models or super learning (SL) are presented for four weight truncation schemes along with the corresponding 99th percentile and maximum value of the stabilized and unstabilized inverse probability weights used for implementing IPW and TMLE, respectively. RD is the risk in treatment arm minus the risk in control arm and NNT is the number needed to treat.

Analysis type	Protocol Deviations* by exposure group (%)	PS estimation	99th IP weights	Max IP weight	Estimator	Treatment (SU) risk in %	Control (SGLT2i) risk in %	RD [95% CI] in %	NNT	HR [95% CI]
PP	Discontinuation<br>SU: 48.52<br>SGLT2i: 25.10<br><br>Crossover<br>SU: 5.19<br>SGLT2i: 4.36	SL			Unadjusted	12.87	11.71	1.17 [-1.30, 3.64]		1.10 [0.90, 1.30]
			14.07	1,294.31	TMLE untruncated	13.41	6.69	6.72 [5.60, 7.84]	15	
					TMLE truncated at 200	13.41	7.41	6.00 [4.95, 7.05]	17	
			2.75	250.41	IPW untruncated	13.05	6.68	6.37 [4.08, 8.65]	16	1.59 [1.01, 2.18]
					IPW truncated at 20	13.10	7.07	6.03 [3.75, 8.31]	17	1.45 [0.95, 1.95]
					IPW truncated at 99th	13.28	8.13	5.14 [2.86, 7.43]	19	1.27 [0.91, 1.63]
		Logistic model	3.63	961.66	IPW untruncated	13.14	5.67	7.48 [5.13, 9.82]	13	1.94 [1.09, 2.80]
					IPW truncated at 20	13.53	6.74	6.79 [4.51, 9.07]	15	1.53 [1.01, 2.05]
					IPW truncated at 99th	13.56	7.75	5.80 [3.51, 8.10]	17	1.30 [0.90, 1.70]
ITT		SL			Unadjusted	13.68	12.57	1.11 [-0.80, 3.03]		1.02 [0.86, 1.19]
			11.35	800.99	TMLE untruncated	14.60	11.70	2.89 [-0.45, 6.24]		
					TMLE truncated at 200	14.60	11.49	3.11 [1.11, 5.11]	32	
			2.70	239.08	IPW untruncated	14.19	11.71	2.49 [-4.57, 9.54]		1.61 [1.08, 2.13]
					IPW truncated at 20	14.25	12.42	1.83 [-5.44, 9.11]		1.45 [1.02, 1.89]
					IPW truncated at 99th	14.04	11.83	2.20 [-1.62, 6.03]		1.20 [0.90, 1.51]
		Logistic model	3.31	2,093.00	IPW untruncated	13.96	9.62	4.35 [-4.24, 12.94]		2.19 [1.21, 3.16]
					IPW truncated at 20	14.25	11.05	3.20 [-3.32, 9.72]		1.58 [1.11, 2.06]
					IPW truncated at 99th	13.90	10.81	3.09 [-0.34, 6.53]		1.26 [0.92, 1.60]

* Discontinuation refers to the interruption of the comparator medication initiated on index date; Crossover refers to the initiation of the comparator medication initiated by patient at baseline in the other arm.

© 2025 Neugebauer R et al. *JAMA Network Open*.

eFigure 14. MACE (Primary Definition), 2-Arm Drug Class Comparison, Sulfonylureas vs SGLT2is, No ASCVD Subgroup, Cumulative Incidence Curves From PP and ITT Analyses With IPW, TMLE, and SL
Each plot emulates inferences among patients with No ASCVD from a 2-arm RCT comparing SU and SGLT2i and represents unadjusted or adjusted estimates of cumulative incidence curves for MACE derived with IPW and TMLE with SL estimates of propensity scores with four weight truncation schemes: IPW and TMLE without weight truncation (untruncated), IPW with truncation of stabilized weights at value 20 (trunc20) or at the 99th percentile of weight values (trunc99), and TMLE with truncation of unstabilized weights at value 200 (trunc200). The red divider line separates results of Per-Protocol (PP) analyses (top half) from Intention-To-Treat (ITT) analyses (bottom half). Each plot displays a p value for the test that the average risk difference (ARD) through 2.5 years of follow-up (30 months) is 0.

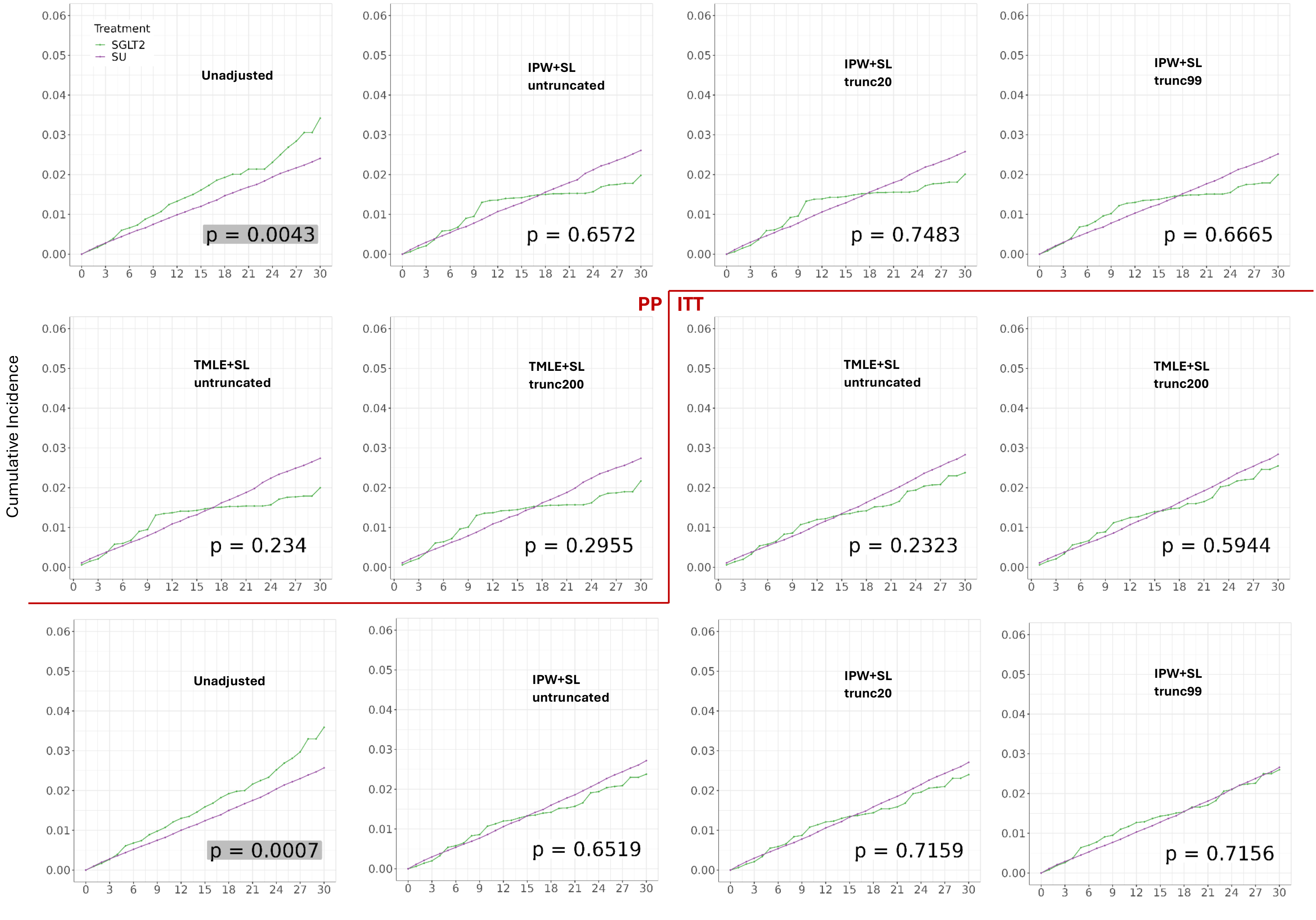

© 2025 Neugebauer R et al. *JAMA Network Open*.

eTable 17. MACE (Primary Definition), 2-Arm Drug Class Comparison, Sulfonylureas vs SGLT2is, No ASCVD Subgroup, RD and HR Effect Measures at 2.5 Years

Estimation results among patients with No ASCVD from ITT and PP analyses of emulated 2-arm RCTs comparing MACE risks over 2.5 years between SU and SGLT2i initiators. For PP analyses, rates of protocol deviations are described by medication class initiated at baseline. Unadjusted point and interval estimates and adjusted point and interval IPW and TMLE estimates of risks, risk differences (RD), and hazard ratios (HR) based on propensity scores (PS) estimated with either logistic models or super learning (SL) are presented for four weight truncation schemes along with the corresponding 99th percentile and maximum value of the stabilized and unstabilized inverse probability weights used for implementing IPW and TMLE, respectively. RD is the risk in treatment arm minus the risk in control arm and NNT is the number needed to treat.

Analysis type	Protocol Deviations* by exposure group (%)	PS estimation	99th IP weights	Max IP weight	Estimator	Treatment (SU) risk in %	Control (SGLT2i) risk in %	RD [95% CI] in %	NNT	HR [95% CI]
PP	Discontinuation<br>SU: 49.70<br>SGLT2i: 27.53<br><br>Crossover<br>SU: 4.36<br>SGLT2i: 6.23	SL	[redacted]		Unadjusted	2.41	3.42	-1.01 [-1.73, -0.29]	99	0.79 [0.64, 0.93]
			15.55	2,994.07	TMLE untruncated	2.74	2.00	0.74 [0.33, 1.15]	135	[redacted]
					TMLE truncated at 200	2.74	2.17	0.57 [0.22, 0.92]	176	
			2.65	404.97	IPW untruncated	2.61	1.98	0.63 [-0.21, 1.47]		0.87 [0.50, 1.24]
					IPW truncated at 20	2.58	2.01	0.57 [-0.27, 1.41]		0.85 [0.49, 1.21]
					IPW truncated at 99th	2.52	2.00	0.52 [-0.08, 1.11]		0.81 [0.56, 1.07]
		Logistic model	3.39	5,906.96	IPW untruncated	2.78	2.12	0.66 [-0.64, 1.96]		0.81 [0.33, 1.29]
					IPW truncated at 20	2.69	2.20	0.49 [-0.74, 1.73]		0.75 [0.34, 1.16]
					IPW truncated at 99th	2.58	2.02	0.57 [-0.11, 1.24]		0.79 [0.52, 1.06]
ITT	[redacted]	SL	[redacted]		Unadjusted	2.57	3.59	-1.02 [-1.54, -0.49]	98	0.79 [0.66, 0.92]
			12.21	1,153.76	TMLE untruncated	2.83	2.38	0.45 [0.10, 0.81]	220	[redacted]
					TMLE truncated at 200	2.84	2.55	0.29 [-0.07, 0.64]		
			2.32	628.80	IPW untruncated	2.72	2.38	0.34 [-0.45, 1.12]		0.94 [0.6, 1.28]
					IPW truncated at 20	2.71	2.40	0.31 [-0.47, 1.10]		0.93 [0.59, 1.26]
					IPW truncated at 99th	2.66	2.60	0.06 [-0.68, 0.79]		0.84 [0.61, 1.08]
		Logistic model	3.04	3,037.44	IPW untruncated	2.83	2.42	0.41 [-0.75, 1.57]		1.01 [0.56, 1.45]
					IPW truncated at 20	2.76	2.72	0.03 [-1.19, 1.25]		0.89 [0.51, 1.27]
					IPW truncated at 99th	2.68	2.70	-0.02 [-0.82, 0.77]		0.82 [0.58, 1.06]

* Discontinuation refers to the interruption of the comparator medication initiated on index date; Crossover refers to the initiation of the comparator medication initiated by patient at baseline in the other arm.

© 2025 Neugebauer R et al. *JAMA Network Open*.

eFigure 15. MACE (Primary Definition), 2-Arm Drug Class Comparison, Sulfonylureas vs SGLT2is, No ASCVD and MET Subgroup, Cumulative Incidence Curves From PP and ITT Analyses With IPW, TMLE, and SL
Each plot emulates inferences among patients with No ASCVD and MET from a 2-arm RCT comparing SU and SGLT2i and represents unadjusted or adjusted estimates of cumulative incidence curves for MACE derived with IPW and TMLE with SL estimates of propensity scores with four weight truncation schemes: IPW and TMLE without weight truncation (untruncated), IPW with truncation of stabilized weights at value 20 (trunc20) or at the 99th percentile of weight values (trunc99), and TMLE with truncation of unstabilized weights at value 200 (trunc200). The red divider line separates results of Per-Protocol (PP) analyses (top half) from Intention-To-Treat (ITT) analyses (bottom half). Each plot displays a p value for the test that the average risk difference (ARD) through 2.5 years of follow-up (30 months) is 0.

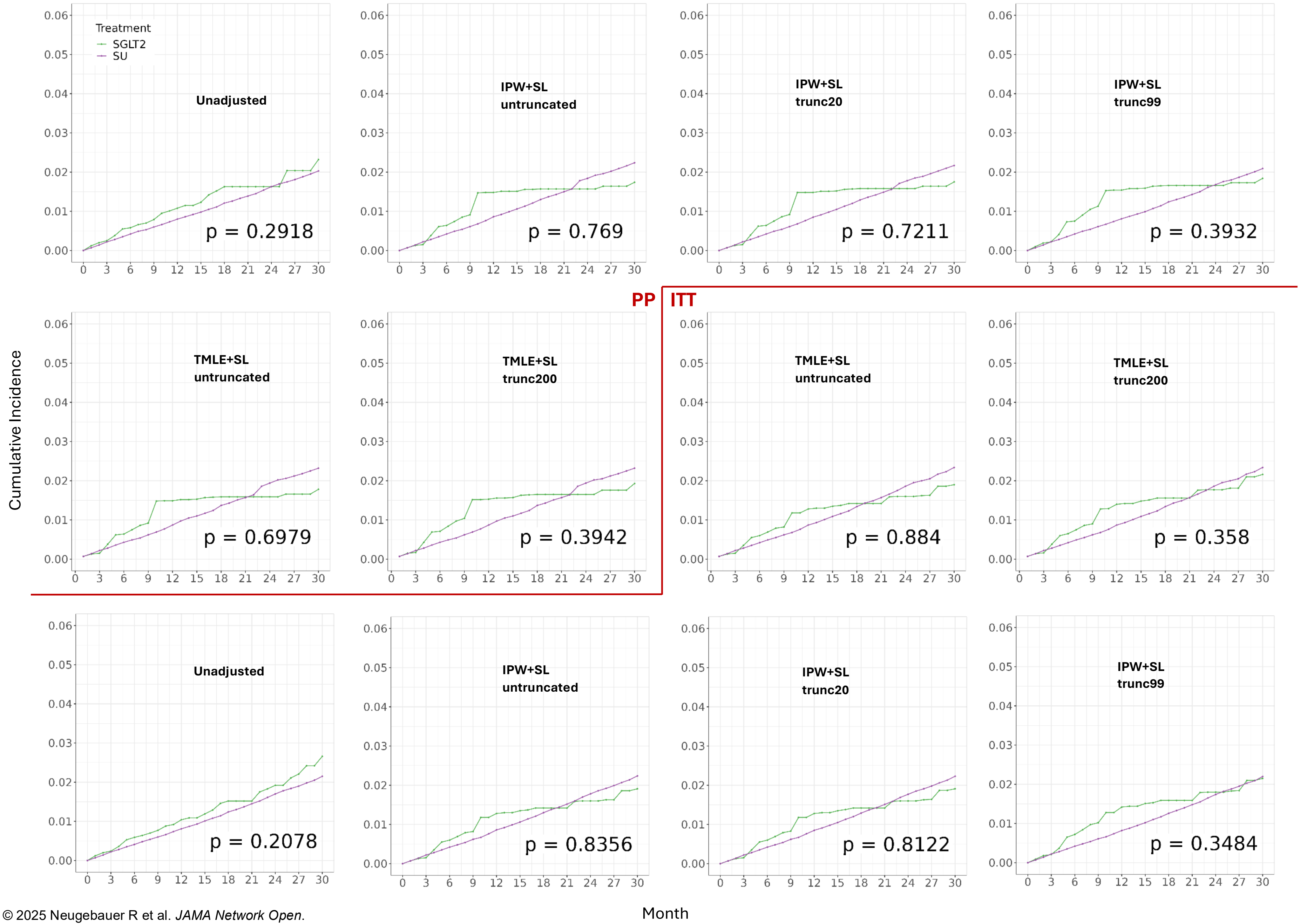

© 2025 Neugebauer R et al. *JAMA Network Open*.

eTable 18. MACE (Primary Definition), 2-Arm Drug Class Comparison, Sulfonylureas vs SGLT2is, No ASCVD and MET Subgroup, RD and HR Effect Measures at 2.5 Years

Estimation results among patients with No ASCVD and MET from ITT and PP analyses of emulated 2-arm RCTs comparing MACE risks over 2.5 years between SU and SGLT2i initiators. For PP analyses, rates of protocol deviations are described by medication class initiated at baseline. Unadjusted point and interval estimates and adjusted point and interval IPW and TMLE estimates of risks, risk differences (RD), and hazard ratios (HR) based on propensity scores (PS) estimated with either logistic models or super learning (SL) are presented for four weight truncation schemes along with the corresponding 99th percentile and maximum value of the stabilized and unstabilized inverse probability weights used for implementing IPW and TMLE, respectively. RD is the risk in treatment arm minus the risk in control arm and NNT is the number needed to treat.

Analysis type	Protocol Deviations* by exposure group (%)	PS estimation	99th IP weights	Max IP weight	Estimator	Treatment (SU) risk in %	Control (SGLT2i) risk in %	RD [95% CI] in %	NNT	HR [95% CI]
PP	Discontinuation<br>SU: 45.40<br>SGLT2i: 23.63<br><br>Crossover<br>SU: 4.95<br>SGLT2i: 9.49	SL	[redacted]	[redacted]	Unadjusted	2.03	2.32	-0.29 [-1.28, 0.70]		0.74 [0.49, 0.99]
			14.03	2,662.21	TMLE untruncated	2.32	1.78	0.55 [-0.07, 1.17]		[redacted]
					TMLE truncated at 200	2.32	1.93	0.40 [-0.10, 0.89]		
			6.65	223.82	IPW untruncated	2.24	1.74	0.49 [-0.64, 1.63]		0.63 [0.26, 1.01]
					IPW truncated at 20	2.17	1.75	0.42 [-0.70, 1.55]		0.63 [0.25, 1.00]
					IPW truncated at 99th	2.09	1.84	0.25 [-0.63, 1.13]		0.55 [0.29, 0.81]
		Logistic model	3.01	2,490.03	IPW untruncated	7.49	1.86	5.63 [-4.25, 15.52]		0.60 [0.19, 1.02]
					IPW truncated at 20	2.26	1.94	0.32 [-1.12, 1.75]		0.56 [0.18, 0.94]
					IPW truncated at 99th	2.12	1.79	0.33 [-0.58, 1.24]		0.54 [0.27, 0.81]
ITT	[redacted]	SL	[redacted]	[redacted]	Unadjusted	2.15	2.66	-0.50 [-1.30, 0.29]		0.78 [0.53, 1.03]
			11.04	1,062.58	TMLE untruncated	2.34	1.90	0.43 [-0.04, 0.91]		[redacted]
					TMLE truncated at 200	2.34	2.16	0.18 [-0.28, 0.65]		
			1.89	136.86	IPW untruncated	2.24	1.91	0.33 [-0.59, 1.25]		0.71 [0.33, 1.09]
					IPW truncated at 20	2.23	1.91	0.32 [-0.60, 1.24]		0.70 [0.32, 1.08]
					IPW truncated at 99th	2.20	2.15	0.05 [-0.83, 0.92]		0.60 [0.34, 0.87]
		Logistic model	2.62	2,336.42	IPW untruncated	2.27	1.73	0.54 [-0.45, 1.53]		0.71 [0.26, 1.16]
					IPW truncated at 20	2.24	1.95	0.30 [-0.74, 1.34]		0.66 [0.24, 1.07]
					IPW truncated at 99th	2.18	2.09	0.10 [-0.76, 0.95]		0.62 [0.33, 0.90]

* Discontinuation refers to the interruption of the comparator medication initiated on index date; Crossover refers to the initiation of the comparator medication initiated by patient at baseline in the other arm.

© 2025 Neugebauer R et al. *JAMA Network Open*.

eFigure 16. MACE (Primary Definition), 2-Arm Drug Class Comparison, Sulfonylureas vs GLP-1RAs, CONSORT Diagram

Flow diagram describing the inclusion and exclusion steps and counts leading to the creation of the cohort for emulating the 2-arm RCT to compare the risk of MACE in new users of SU and GLP-1RA along with sample sizes and counts for each observed end of follow-up type by treatment initiated at cohort entry.

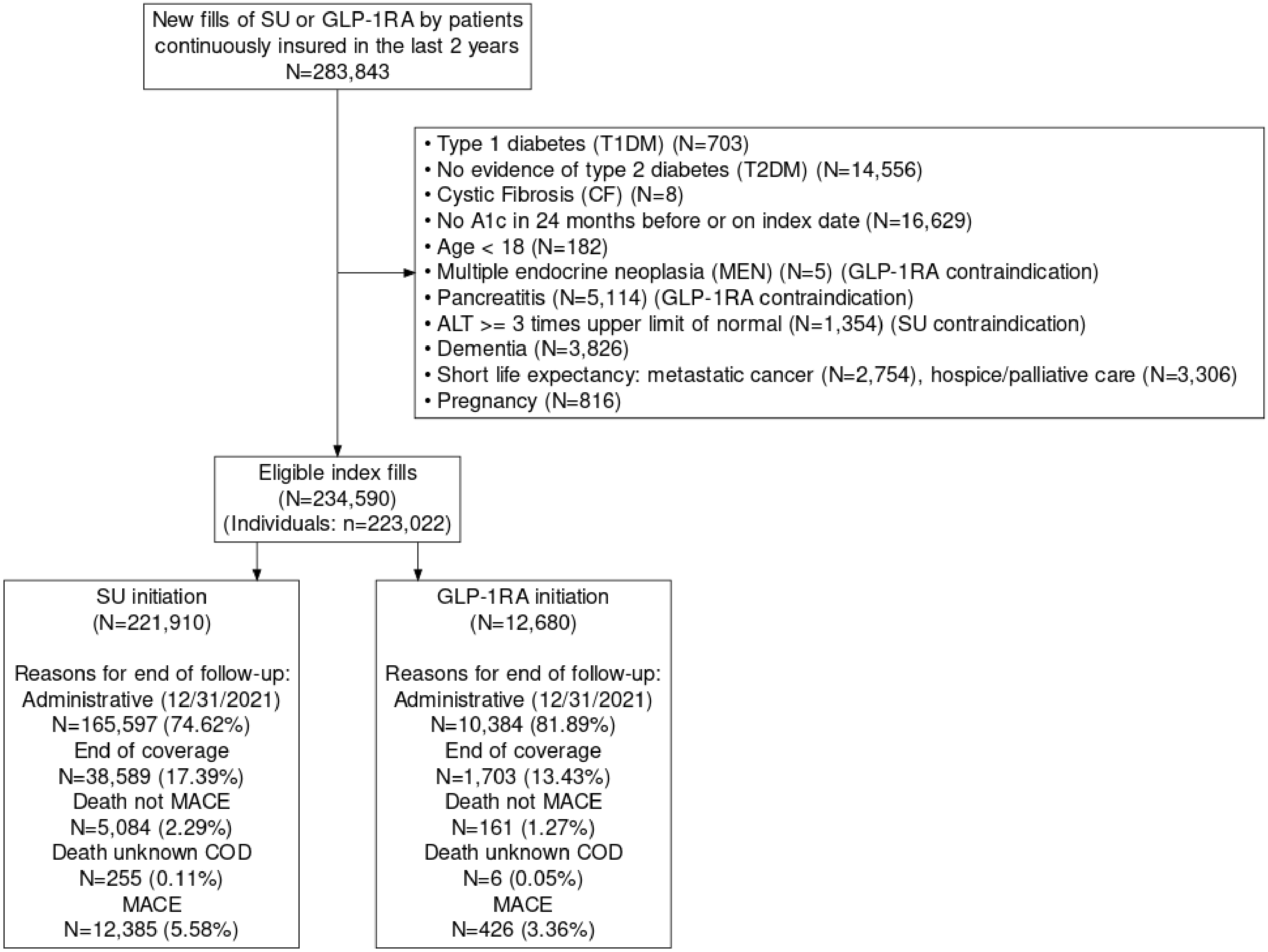

© 2025 Neugebauer R et al. *JAMA Network Open*.

eTable 19. MACE (Primary Definition), 2-Arm Drug Class Comparison, Sulfonylureas vs GLP-1RAs, Patient Characteristics at Baseline (Overall and by Medication Initiated)

Summary statistics of the baseline values for selected covariates in the cohort of patients used to emulate a 2-arm RCT for comparing SU and GLP-1RA. For each continuous variable, the mean and standard deviation are displayed for all patients in the cohort (last column) and by drug class initiated at cohort entry. For each categorical variable and for each possible level of that variable, the count and proportion are displayed instead.

	SU n = 221,910	GLP-1RA n = 12,680	Total n = 234,590
**Demographics**			
Age	57.13 (12.97)	56.51 (12.21)	57.10 (12.93)
Agegrp			
<45	37,020 (16.68%)	2,125 (16.76%)	39,145 (16.69%)
[45-65)	121,274 (54.65%)	7,180 (56.62%)	128,454 (54.76%)
[65-75)	43,801 (19.74%)	2,692 (21.23%)	46,493 (19.82%)
>=75	19,815 (8.93%)	683 (5.39%)	20,498 (8.74%)
Ethnicity			
Hispanic	81,809 (36.87%)	2,497 (19.69%)	84,306 (35.94%)
Nonhispanic	140,101 (63.13%)	10,183 (80.31%)	150,284 (64.06%)
Female Head Of Hh	0.150 (0.072)	0.137 (0.076)	0.150 (0.072)
Missing	1,135 (0.511%)	748 (5.899%)	1,883 (0.803%)
Hh Public Assistance	0.039 (0.035)	0.036 (0.034)	0.039 (0.035)
Missing	1,135 (0.511%)	748 (5.899%)	1,883 (0.803%)
Household Income Less 30k	0.206 (0.119)	0.211 (0.126)	0.206 (0.119)
Missing	1,135 (0.511%)	748 (5.899%)	1,883 (0.803%)
Houspoverty	0.105 (0.089)	0.095 (0.088)	0.104 (0.089)
Missing	1,140 (0.514%)	752 (5.931%)	1,892 (0.807%)
Index Yr			
2014	26,789 (12.07%)	266 (2.1%)	27,055 (11.53%)
2015	26,652 (12.01%)	420 (3.31%)	27,072 (11.54%)
2016	28,978 (13.06%)	527 (4.16%)	29,505 (12.58%)
2017	29,307 (13.21%)	921 (7.26%)	30,228 (12.89%)
2018	26,677 (12.02%)	1,619 (12.77%)	28,296 (12.06%)
2019	27,336 (12.32%)	1,924 (15.17%)	29,260 (12.47%)
2020	25,162 (11.34%)	2,661 (20.99%)	27,823 (11.86%)
2021	31,009 (13.97%)	4,342 (34.24%)	35,351 (15.07%)
Bmi	32.79 (7.30)	38.11 (8.31)	33.07 (7.45)
Missing	6,197 (2.79%)	419 (3.3%)	6,616 (2.82%)
Smoking Status			
Formersmoker	60,614 (27.31%)	4,098 (32.32%)	64,712 (27.59%)
Currentsmoker	20,416 (9.2%)	1,302 (10.27%)	21,718 (9.26%)
Passivesmoker	1,189 (0.54%)	60 (0.47%)	1,249 (0.53%)
Neversmoker	137,108 (61.79%)	7,016 (55.33%)	144,124 (61.44%)
Unknown	2,583 (1.16%)	204 (1.61%)	2,787 (1.19%)
Low Educ	0.180 (0.134)	0.132 (0.108)	0.177 (0.133)
Missing	1,132 (0.51%)	746 (5.883%)	1,878 (0.801%)
Mgr Male	0.046 (0.045)	0.043 (0.043)	0.046 (0.045)
Missing	1,134 (0.511%)	747 (5.891%)	1,881 (0.802%)
Ndi	0.29 (0.17)	0.25 (0.15)	0.28 (0.17)
Missing	1,140 (0.51%)	752 (5.93%)	1,892 (0.81%)
Pct Crowding	0.093 (0.087)	0.061 (0.074)	0.091 (0.087)
Missing	1,135 (0.511%)	748 (5.899%)	1,883 (0.803%)
Racegrp			
White	108,600 (48.94%)	7,946 (62.67%)	116,546 (49.68%)
Asian	35,376 (15.94%)	1,003 (7.91%)	36,379 (15.51%)
Blackorafricanamerican	22,091 (9.95%)	1,748 (13.79%)	23,839 (10.16%)
Hawaiianorpacificislander	3,480 (1.57%)	192 (1.51%)	3,672 (1.57%)
Americanindianoralaskanative	1,557 (0.7%)	72 (0.57%)	1,629 (0.69%)
Multirace	6,106 (2.75%)	647 (5.1%)	6,753 (2.88%)
Other	174 (0.08%)	77 (0.61%)	251 (0.11%)
Unknown	44,526 (20.06%)	995 (7.85%)	45,521 (19.4%)
Sex			

© 2025 Neugebauer R et al. *JAMA Network Open*.

	SU n = 221,910	GLP-1RA n = 12,680	Total n = 234,590
Female	101,231 (45.62%)	7,284 (57.44%)	108,515 (46.26%)
Male	120,673 (54.38%)	5,396 (42.56%)	126,069 (53.74%)
Other	4 (0%)	0 (0%)	4 (0%)
Unknown	2 (0%)	0 (0%)	2 (0%)
Site			
Kpnc	91,077 (41.04%)	1,982 (15.63%)	93,059 (39.67%)
Kpsc	115,111 (51.87%)	3,864 (30.47%)	118,975 (50.72%)
Kphi	5,764 (2.6%)	904 (7.13%)	6,668 (2.84%)
Hpi	2,930 (1.32%)	1,784 (14.07%)	4,714 (2.01%)
Hfhs	4,049 (1.82%)	1,301 (10.26%)	5,350 (2.28%)
Ghs	2,979 (1.34%)	2,845 (22.44%)	5,824 (2.48%)
Unemployment	0.050 (0.027)	0.040 (0.026)	0.050 (0.027)
Missing	1,129 (0.509%)	746 (5.883%)	1,875 (0.799%)
**Insurance**			
Ins Commercial	172,108 (77.56%)	7,428 (58.58%)	179,536 (76.53%)
Ins Highdeductible	19,988 (9.01%)	753 (5.94%)	20,741 (8.84%)
Ins Medicaid	13,376 (6.03%)	2,006 (15.82%)	15,382 (6.56%)
Ins Medicare	62,666 (28.24%)	3,771 (29.74%)	66,437 (28.32%)
Ins Medicare A	37,102 (16.72%)	2,803 (22.11%)	39,905 (17.01%)
Ins Medicare B	34,784 (15.67%)	2,643 (20.84%)	37,427 (15.95%)
Ins Medicare C	29,823 (13.44%)	2,415 (19.05%)	32,238 (13.74%)
Ins Medicare D	32,390 (14.6%)	2,510 (19.79%)	34,900 (14.88%)
Ins Other Coverage	49,229 (22.18%)	2,539 (20.02%)	51,768 (22.07%)
Ins Privatepay	41,707 (18.79%)	1,224 (9.65%)	42,931 (18.3%)
Ins Selffunded	3,998 (1.8%)	1,012 (7.98%)	5,010 (2.14%)
Ins Statesubsidized	796 (0.36%)	990 (7.81%)	1,786 (0.76%)
**Clinical data**			
A1c Age	42.67 (78.83)	59.40 (93.29)	43.57 (79.76)
Missing	230 (0.1%)	28 (0.22%)	258 (0.11%)
Acc Aha 201310yrcvdrisk	0.169 (0.152)	0.158 (0.140)	0.169 (0.151)
Missing	26,418 (11.905%)	1,478 (11.656%)	27,896 (11.891%)
Chf Dx Status			
0	211,986 (95.53%)	11,486 (90.58%)	223,472 (95.26%)
1	9,016 (4.06%)	1,083 (8.54%)	10,099 (4.3%)
999	908 (0.41%)	111 (0.88%)	1,019 (0.43%)
Cv Risk Subgrp			
Low	85,588 (38.57%)	4,932 (38.9%)	90,520 (38.59%)
Moderate	45,049 (20.3%)	2,702 (21.31%)	47,751 (20.36%)
High	60,375 (27.21%)	3,112 (24.54%)	63,487 (27.06%)
Other	4,980 (2.24%)	489 (3.86%)	5,469 (2.33%)
Unknown	25,918 (11.68%)	1,445 (11.4%)	27,363 (11.66%)
Diab Duration	4.93 (3.24)	7.21 (3.67)	5.06 (3.30)
Missing	364 (0.16%)	9 (0.07%)	373 (0.16%)
A1c	9.22 (2.08)	8.77 (1.85)	9.19 (2.07)
Missing	230 (0.1%)	28 (0.22%)	258 (0.11%)
Acr	47.08 (84.17)	64.53 (104.87)	47.95 (85.41)
Missing	44,458 (20.03%)	3,342 (26.36%)	47,800 (20.38%)
Afib Dx	9,158 (4.13%)	714 (5.63%)	9,872 (4.21%)
Alt	33.07 (22.12)	31.32 (19.17)	32.98 (21.97)
Missing	46,513 (20.96%)	2,599 (20.5%)	49,112 (20.94%)
Amputation Dxpx	1,380 (0.62%)	309 (2.44%)	1,689 (0.72%)
Anemia Dx	14,234 (6.41%)	1,586 (12.51%)	15,820 (6.74%)
Anxiety Dx	38,312 (17.26%)	3,823 (30.15%)	42,135 (17.96%)
Arrythmia Dx	8,139 (3.67%)	901 (7.11%)	9,040 (3.85%)
Ascvd Dxpx Max	21,291 (9.59%)	1,993 (15.72%)	23,284 (9.93%)
Ascvd Dxpx Ppv	4,980 (2.24%)	489 (3.86%)	5,469 (2.33%)
Asthma Dx	22,593 (10.18%)	2,138 (16.86%)	24,731 (10.54%)

© 2025 Neugebauer R et al. *JAMA Network Open*.

	SU<br>n = 221,910	GLP-1RA<br>n = 12,680	Total<br>n = 234,590
Bariatric Px	2,239 (1.01%)	394 (3.11%)	2,633 (1.12%)
Bipolar Dx	1,982 (0.89%)	294 (2.32%)	2,276 (0.97%)
Blind Dx	807 (0.36%)	42 (0.33%)	849 (0.36%)
Cad Dxpx Max	13,299 (5.99%)	1,218 (9.61%)	14,517 (6.19%)
Cad Dxpx Ppv	3,033 (1.37%)	344 (2.71%)	3,377 (1.44%)
Cad Dxpx Sens	3,421 (1.54%)	386 (3.04%)	3,807 (1.62%)
Cancer Mets Dx	0 (0%)	0 (0%)	0 (0%)
Cancer Nomets Dx	8,353 (3.76%)	921 (7.26%)	9,274 (3.95%)
Cevd Dxpx Ppv	1,893 (0.85%)	108 (0.85%)	2,001 (0.85%)
Cevd Dxpx Sens	7,018 (3.16%)	659 (5.2%)	7,677 (3.27%)
Chf Dx Ppv	2,127 (0.96%)	229 (1.81%)	2,356 (1%)
Chf Dx Sens	9,016 (4.06%)	1,083 (8.54%)	10,099 (4.3%)
Ckd Dx	46,210 (20.82%)	3,913 (30.86%)	50,123 (21.37%)
Copd Dx	8,025 (3.62%)	883 (6.96%)	8,908 (3.8%)
Coupled Dbp	74.90 (10.79)	74.02 (11.12)	74.86 (10.81)
Missing	3,979 (1.79%)	301 (2.37%)	4,280 (1.82%)
Coupled Sbp	129.52 (14.59)	130.33 (14.83)	129.56 (14.60)
Missing	3,979 (1.79%)	301 (2.37%)	4,280 (1.82%)
Covid Prd	56,171 (25.31%)	7,003 (55.23%)	63,174 (26.93%)
Creat	0.92 (0.51)	0.96 (0.57)	0.92 (0.52)
Missing	5,965 (2.69%)	383 (3.02%)	6,348 (2.71%)
Cysticfibrosis Dx	0 (0%)	0 (0%)	0 (0%)
Dbp	74.05 (10.79)	73.39 (11.21)	74.01 (10.81)
Missing	3,976 (1.79%)	301 (2.37%)	4,277 (1.82%)
Dementia Dx	0 (0%)	0 (0%)	0 (0%)
Depr Dx	29,087 (13.11%)	3,352 (26.44%)	32,439 (13.83%)
Dietitian	10,302 (4.64%)	1,906 (15.03%)	12,208 (5.2%)
Dka Dx	2,992 (1.35%)	386 (3.04%)	3,378 (1.44%)
Dka Dx Count	0.01 (0.13)	0.04 (0.27)	0.02 (0.14)
Esrd Dx	10,315 (4.65%)	1,300 (10.25%)	11,615 (4.95%)
Esrd Px	779 (0.35%)	91 (0.72%)	870 (0.37%)
Etoh Dx	3,662 (1.65%)	237 (1.87%)	3,899 (1.66%)
Fasciitis Dx	166 (0.07%)	41 (0.32%)	207 (0.09%)
Fpg	180.82 (73.79)	173.38 (75.14)	180.60 (73.84)
Missing	146,519 (66.03%)	10,322 (81.4%)	156,841 (66.86%)
Frailty Dx	14,485 (6.53%)	2,036 (16.06%)	16,521 (7.04%)
Gfr Epi 09	87.92 (22.94)	84.12 (25.04)	87.71 (23.07)
Missing	5,965 (2.69%)	383 (3.02%)	6,348 (2.71%)
Hdl	44.57 (11.47)	43.37 (11.77)	44.50 (11.49)
Missing	31,242 (14.08%)	1,649 (13%)	32,891 (14.02%)
Hgb	13.99 (1.65)	13.60 (1.60)	13.97 (1.65)
Missing	61,214 (27.59%)	3,019 (23.81%)	64,233 (27.38%)
Htn Dx	121,692 (54.84%)	9,056 (71.42%)	130,748 (55.73%)
Hypo Dx	192 (0.09%)	50 (0.39%)	242 (0.1%)
Hypo Dx Count	0.01 (0.13)	0.03 (0.21)	0.01 (0.13)
Hypo Dx Event	192 (0.09%)	50 (0.39%)	242 (0.1%)
Hypothyroidism Dx	21,416 (9.65%)	2,105 (16.6%)	23,521 (10.03%)
Ldl	96.29 (39.05)	90.20 (38.08)	95.96 (39.02)
Missing	24,770 (11.16%)	1,323 (10.43%)	26,093 (11.12%)
Leukemia Lymphoma Dx	1,310 (0.59%)	74 (0.58%)	1,384 (0.59%)
Lipid Dx	131,945 (59.46%)	8,902 (70.21%)	140,847 (60.04%)
Liver Dx	339 (0.15%)	39 (0.31%)	378 (0.16%)
Mci Dx	1,032 (0.47%)	83 (0.65%)	1,115 (0.48%)
Men2 Dx	0 (0%)	0 (0%)	0 (0%)
Nephropathy Dx	13,228 (5.96%)	652 (5.14%)	13,880 (5.92%)
Neuro Dx	5,170 (2.33%)	606 (4.78%)	5,776 (2.46%)
Pancreatitis Dx	0 (0%)	0 (0%)	0 (0%)

© 2025 Neugebauer R et al. *JAMA Network Open*.

	SU n = 221,910	GLP-1RA n = 12,680	Total n = 234,590
Pcr	0.000 (0.000)	0.000 (0.000)	0.000 (0.000)
Missing	207,053 (93.305%)	11,675 (92.074%)	218,728 (93.238%)
Potassium	4.24 (0.40)	4.26 (0.42)	4.24 (0.41)
Missing	17,572 (7.92%)	633 (4.99%)	18,205 (7.76%)
Pregnancy	0 (0%)	0 (0%)	0 (0%)
Pud Dx	228 (0.1%)	21 (0.17%)	249 (0.11%)
Pvd Dxpx Ppv	427 (0.19%)	65 (0.51%)	492 (0.21%)
Pvd Dxpx Sens	3,989 (1.8%)	526 (4.15%)	4,515 (1.92%)
Pyelo Dx	2,319 (1.05%)	294 (2.32%)	2,613 (1.11%)
Retinopathy Dxpx	7,323 (3.3%)	818 (6.45%)	8,141 (3.47%)
Rpg	214.63 (113.11)	196.45 (93.34)	213.03 (111.63)
Missing	142,163 (64.06%)	4,961 (39.12%)	147,124 (62.72%)
Sbp	128.71 (14.41)	129.69 (14.70)	128.76 (14.43)
Missing	3,979 (1.79%)	301 (2.37%)	4,280 (1.82%)
Schiz Dx	1,335 (0.6%)	100 (0.79%)	1,435 (0.61%)
Sodium	138.06 (3.07)	138.74 (2.93)	138.10 (3.07)
Missing	50,124 (22.59%)	1,403 (11.06%)	51,527 (21.96%)
Sud Dx	2,098 (0.95%)	220 (1.74%)	2,318 (0.99%)
Tc	176.94 (51.97)	167.09 (46.46)	176.40 (51.73)
Missing	30,393 (13.7%)	1,626 (12.82%)	32,019 (13.65%)
Trig	214.08 (209.63)	199.32 (168.07)	213.29 (207.64)
Missing	42,248 (19.04%)	2,493 (19.66%)	44,741 (19.07%)
Tsh	2.05 (1.91)	2.16 (2.14)	2.05 (1.93)
Missing	80,214 (36.15%)	4,452 (35.11%)	84,666 (36.09%)
Valvular Dx	3,825 (1.72%)	380 (3%)	4,205 (1.79%)
Vasculitis Dx	2,736 (1.23%)	180 (1.42%)	2,916 (1.24%)
Only Met No Ascvd	123,942 (55.85%)	2,443 (19.27%)	126,385 (53.87%)
Renal Function Status			
Lowrisk	113,721 (51.25%)	5,190 (40.93%)	118,911 (50.69%)
Moderaterisk	42,640 (19.21%)	2,537 (20.01%)	45,177 (19.26%)
Highrisk	14,003 (6.31%)	1,038 (8.19%)	15,041 (6.41%)
Veryhighrisk	5,826 (2.63%)	501 (3.95%)	6,327 (2.7%)
Unknown	45,720 (20.6%)	3,414 (26.92%)	49,134 (20.94%)
Total Visit C	0.46 (1.83)	1.23 (3.61)	0.50 (1.97)
Total Visit E	0.15 (1.13)	1.24 (3.39)	0.21 (1.38)
Total Visit N	0.17 (2.54)	0.43 (2.76)	0.18 (2.55)
**Concurrent medications**			
Aa	2 (0%)	6 (0.05%)	8 (0%)
Aceinhibitors	77,272 (34.82%)	4,902 (38.66%)	82,174 (35.03%)
Agi	247 (0.11%)	44 (0.35%)	291 (0.12%)
Anticoagulants	7,491 (3.38%)	755 (5.95%)	8,246 (3.52%)
Anticonvulsants	13,063 (5.89%)	2,415 (19.05%)	15,478 (6.6%)
Antidepressantcomb	3 (0%)	1 (0.01%)	4 (0%)
Antidepressantmaoi	26 (0.01%)	3 (0.02%)	29 (0.01%)
Antidepressantndri	3,819 (1.72%)	674 (5.32%)	4,493 (1.92%)
Antidepressantother	0 (0%)	0 (0%)	0 (0%)
Antidepressantsari	5,440 (2.45%)	655 (5.17%)	6,095 (2.6%)
Antidepressantsnri	5,972 (2.69%)	1,170 (9.23%)	7,142 (3.04%)
Antidepressantspo	60 (0.03%)	22 (0.17%)	82 (0.03%)
Antidepressantssri	17,991 (8.11%)	2,177 (17.17%)	20,168 (8.6%)
Antidepressanttca	5,918 (2.67%)	561 (4.42%)	6,479 (2.76%)
Antidepressantteca	1,270 (0.57%)	136 (1.07%)	1,406 (0.6%)
Antiplatelets	12,462 (5.62%)	1,439 (11.35%)	13,901 (5.93%)
Antipsychotic1stgen	473 (0.21%)	36 (0.28%)	509 (0.22%)
Antipsychotic2ndgen	3,265 (1.47%)	434 (3.42%)	3,699 (1.58%)
Anxiety	2,435 (1.1%)	456 (3.6%)	2,891 (1.23%)
Arb	36,903 (16.63%)	3,383 (26.68%)	40,286 (17.17%)

© 2025 Neugebauer R et al. *JAMA Network Open*.

	SU n = 221,910	GLP-1RA n = 12,680	Total n = 234,590
Benzodiazepines	8,027 (3.62%)	763 (6.02%)	8,790 (3.75%)
Betablockers	48,203 (21.72%)	4,248 (33.5%)	52,451 (22.36%)
Clonidine	1,978 (0.89%)	163 (1.29%)	2,141 (0.91%)
Dihydropyridineccb	29,343 (13.22%)	2,363 (18.64%)	31,706 (13.52%)
Dpp4	2,092 (0.94%)	1,032 (8.14%)	3,124 (1.33%)
Hypnoticother	1,647 (0.74%)	211 (1.66%)	1,858 (0.79%)
Injectableantipsychotic	9 (0%)	8 (0.06%)	17 (0.01%)
Ins	16,831 (7.58%)	7,605 (59.98%)	24,436 (10.42%)
Ins Analog	3,495 (1.57%)	3,905 (30.8%)	7,400 (3.15%)
Ins Combo	1,555 (0.7%)	919 (7.25%)	2,474 (1.05%)
Ins Human	14,179 (6.39%)	4,369 (34.46%)	18,548 (7.91%)
Ins La	14,596 (6.58%)	5,852 (46.15%)	20,448 (8.72%)
Ins Sa	4,414 (1.99%)	4,481 (35.34%)	8,895 (3.79%)
Ksparingdiuretics	5,777 (2.6%)	771 (6.08%)	6,548 (2.79%)
Lithium	341 (0.15%)	37 (0.29%)	378 (0.16%)
Loopdiuretics	10,614 (4.78%)	1,679 (13.24%)	12,293 (5.24%)
Meg	167 (0.08%)	128 (1.01%)	295 (0.13%)
Met	149,757 (67.49%)	8,531 (67.28%)	158,288 (67.47%)
Nondihydropyridineccb	3,574 (1.61%)	320 (2.52%)	3,894 (1.66%)
Otherlipidmeds	5,809 (2.62%)	706 (5.57%)	6,515 (2.78%)
Pcsk9mab	29 (0.01%)	16 (0.13%)	45 (0.02%)
Sglt2	1,342 (0.6%)	1,446 (11.4%)	2,788 (1.19%)
Statins	124,916 (56.29%)	8,949 (70.58%)	133,865 (57.06%)
Stimulants	857 (0.39%)	130 (1.03%)	987 (0.42%)
Suold	1,524 (0.69%)	2 (0.02%)	1,526 (0.65%)
Thiazidediuretics	50,883 (22.93%)	3,278 (25.85%)	54,161 (23.09%)
Tir	0 (0%)	0 (0%)	0 (0%)
Tzd	1,968 (0.89%)	258 (2.03%)	2,226 (0.95%)
Only Met Therapy	134,811 (60.75%)	2,695 (21.25%)	137,506 (58.62%)

© 2025 Neugebauer R et al. *JAMA Network Open*.

eFigure 17. MACE (Primary Definition), 2-Arm Drug Class Comparison, Sulfonylureas vs GLP-1RAs, Cumulative Incidence Curves From PP and ITT Analyses With IPW, TMLE, and SL
Each plot emulates inferences from a 2-arm RCT comparing SU and GLP-1RA and represents unadjusted or adjusted estimates of cumulative incidence curves for MACE derived with inverse probability weighting (IPW) and Targeted Minimum Loss-based Estimation (TMLE) with Super Learning (SL) estimates of propensity scores with four weight truncation schemes: IPW and TMLE without weight truncation (untruncated), IPW with truncation of stabilized weights at value 20 (trunc20) or at the 99th percentile of weight values (trunc99), and TMLE with truncation of unstabilized weights at value 200 (trunc200). The red divi der line separates results of Per-Protocol (PP) analyses (top half) from Intention-To-Treat (ITT) analyses (bottom half). Each plot displays a p value for the test that the average risk difference (ARD) through 2.5 years of follow-up (30 months) is 0.

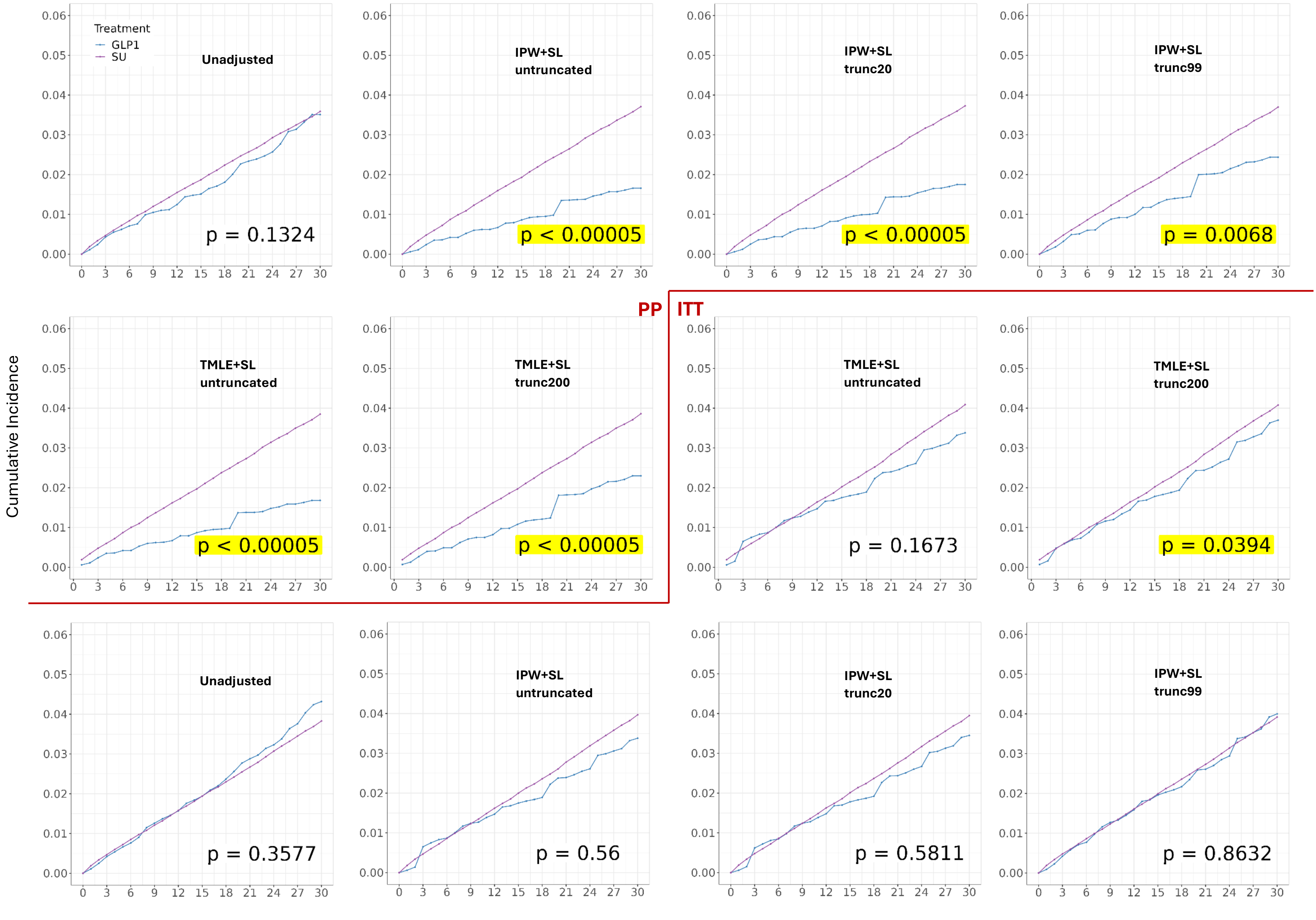

© 2025 Neugebauer R et al. *JAMA Network Open*.

eFigure 18. MACE (Primary Definition), 2-Arm Drug Class Comparison, Sulfonylureas vs GLP-1RAs, Cumulative Incidence Curves From Sensitivity PP Analyses With IPW, TMLE, and SL
Each plot emulates inferences from a 2-arm RCT comparing SU and GLP-1RA and represents unadjusted or adjusted estimates of cumulative incidence curves for MACE from sensitivity PP analyses referred to as "NoMBS PP" and "No3 PP". NoMBS PP analyses are restricted to patients without a history of MBS at baseline and the protocols they evaluate preclude metabolic bariatric surgery (MBS) procedures. The protocols in the No3 PP analyses preclude exposure to three medication classes: the comparator medication from the other arm, DPP4i and SGLT2i.

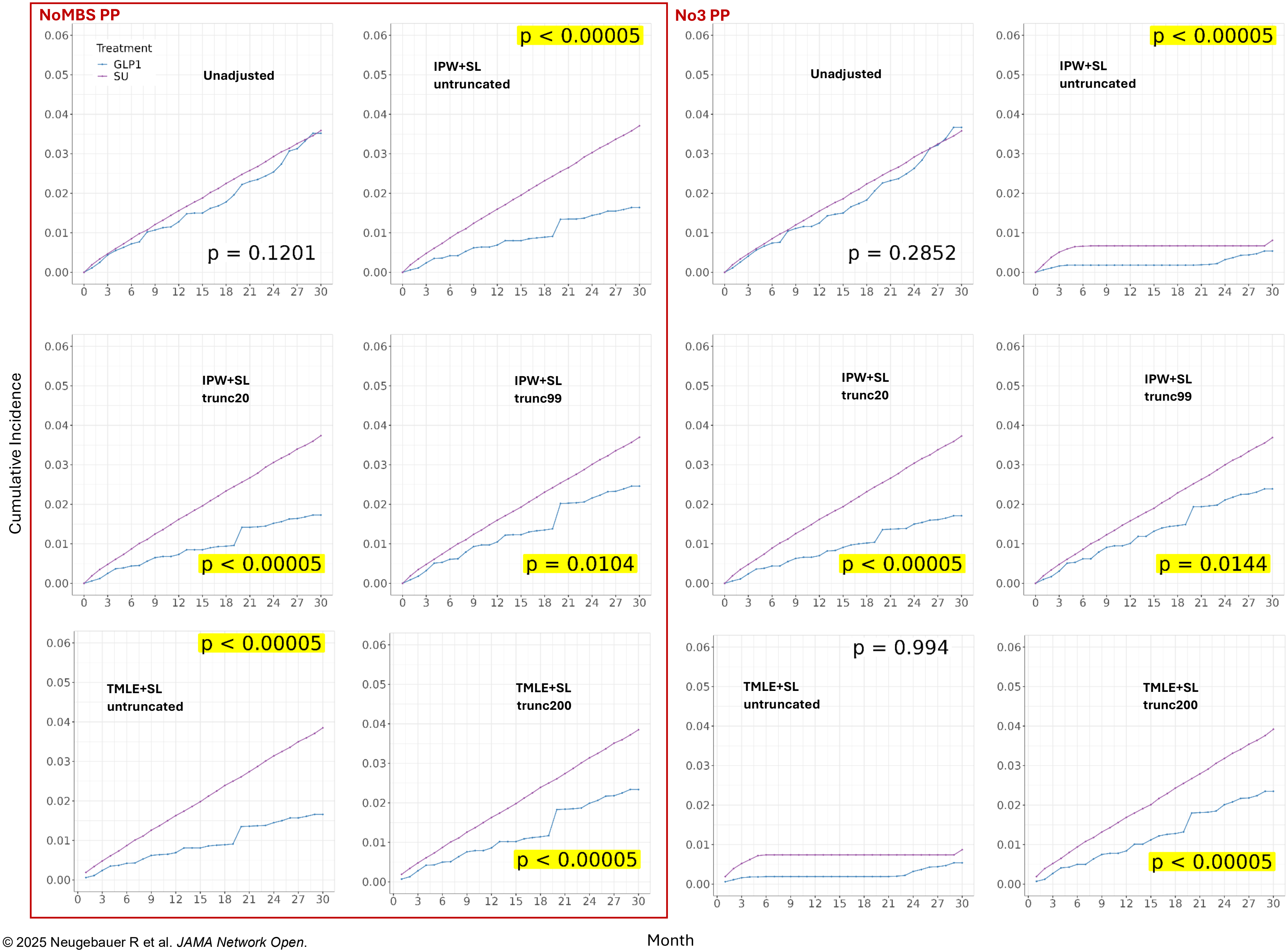

© 2025 Neugebauer R et al. *JAMA Network Open*.

eTable 20. MACE (Primary Definition), 2-Arm Drug Class Comparison, Sulfonylureas vs GLP-1RAs, RD and HR Effect Measures at 2.5 Years

Estimation results from ITT, PP, NoMBS PP, and No3 PP analyses of emulated 2-arm RCTs comparing MACE risks over 2.5 years between SU and GLP-1RA initiators. For PP analyses, rates of protocol deviations are described by medication class initiated at baseline. Unadjusted point and interval estimates and adjusted point and interval IPW and TMLE estimates of risks, risk differences (RD), and hazard ratios (HR) based on propensity scores (PS) estimated with either logistic models or super learning (SL) are presented for four weight truncation schemes along with the corresponding 99th percentile and maximum value of the stabilized and unstabilized inverse probability weights used for implementing IPW and TMLE, respectively. RD is the risk in treatment arm minus the risk in control arm and NNT is the number needed to treat.

Analysis type	Protocol Deviations* by exposure group (%)	PS estimation	99th IP weights	Max IP weight	Estimator	Treatment (SU) risk in %	Control (GLP-1RA) risk in %	RD [95% CI] in %	NNT	HR [95% CI]
PP	Discontinuation<br>SU: 49.75<br>GLP-1RA: 43.76<br><br>Crossover<br>SU: 1.56<br>GLP-1RA: 3.76	SL	[redacted]		Unadjusted	3.59	3.51	0.08 [-0.58, 0.74]		1.22 [1.00, 1.45]
			10.10	5,749.67	TMLE untruncated	3.85	1.68	2.17 [1.92, 2.42]	46	[redacted]
					TMLE truncated at 200	3.86	2.30	1.55 [1.31, 1.79]	64	
			2.60	413.76	IPW untruncated	3.71	1.66	2.05 [1.34, 2.76]	49	2.28 [1.32, 3.24]
					IPW truncated at 20	3.73	1.75	1.98 [1.25, 2.71]	51	2.17 [1.27, 3.08]
					IPW truncated at 99th	3.70	2.44	1.26 [0.31, 2.21]	80	1.53 [0.97, 2.10]
		Logistic model	3.23	2,107.06	IPW untruncated	4.11	1.15	2.96 [2.17, 3.74]	34	2.57 [1.10, 4.04]
					IPW truncated at 20	3.93	1.56	2.37 [1.73, 3.00]	42	1.98 [0.93, 3.04]
					IPW truncated at 99th	3.80	2.26	1.54 [0.75, 2.33]	65	1.46 [0.89, 2.02]
ITT	[redacted]	SL	[redacted]		Unadjusted	3.83	4.32	-0.49 [-1.00, 0.03]		1.00 [0.86, 1.14]
			7.44	2,740.82	TMLE untruncated	4.09	3.38	0.71 [0.14, 1.28]	141	[redacted]
					TMLE truncated at 200	4.08	3.70	0.39 [-0.02, 0.79]		
			2.27	629.69	IPW untruncated	3.97	3.38	0.59 [-0.71, 1.89]		1.14 [0.47, 1.80]
					IPW truncated at 20	3.95	3.45	0.50 [-0.76, 1.76]		1.13 [0.53, 1.73]
					IPW truncated at 99th	3.92	4.00	-0.07 [-1.18, 1.03]		1.05 [0.75, 1.35]
		Logistic model	2.92	3,429.46	IPW untruncated	4.08	2.89	1.18 [-0.06, 2.42]		1.24 [0.55, 1.92]
					IPW truncated at 20	4.02	3.48	0.54 [-0.73, 1.81]		1.05 [0.54, 1.55]
					IPW truncated at 99th	3.97	4.08	-0.11 [-1.27, 1.06]		0.97 [0.68, 1.26]
NoMBS PP	Discontinuation<br>SU: 49.41<br>GLP-1RA: 43.02<br><br>Crossover<br>SU: 1.94<br>GLP-1RA: 4.86<br><br>MBS occurrence<br>SU: 0.41<br>GLP-1RA: 1.07	SL	[redacted]		Unadjusted	3.59	3.52	0.07 [-0.60, 0.75]		1.23 [1, 1.45]
			9.75	5,280.23	TMLE untruncated	3.85	1.66	2.19 [1.94, 2.44]	46	[redacted]
					TMLE truncated at 200	3.85	2.34	1.52 [1.28, 1.76]	66	
			2.59	344.52	IPW untruncated	3.71	1.64	2.07 [1.33, 2.80]	48	2.33 [1.30, 3.36]
					IPW truncated at 20	3.74	1.73	2.00 [1.25, 2.76]	50	2.20 [1.24, 3.17]
					IPW truncated at 99th	3.70	2.46	1.24 [0.24, 2.25]	81	1.54 [0.94, 2.13]
		Logistic model	3.25	122,987,278	IPW untruncated	2.93	1.13	1.81 [1.15, 2.47]	55	2.65 [1.12, 4.19]
					IPW truncated at 20	3.95	1.50	2.45 [1.83, 3.07]	41	2.03 [0.93, 3.13]
					IPW truncated at 99th	3.81	2.22	1.59 [0.81, 2.37]	63	1.47 [0.88, 2.06]
No3 PP	Discontinuation<br>SU: 47.50<br>GLP-1RA: 34.09<br><br>Crossover to comparator drug<br>SU: 1.11<br>GLP-1RA: 2.48<br><br>Initiation of one of the two non-comparator drugs<br>SU: 7.58<br>GLP-1RA: 25.12	SL	[redacted]		Unadjusted	3.58	3.67	-0.09 [-0.89, 0.72]		1.19 [0.94, 1.44]
			9.24	4.33e+27	TMLE untruncated	0.87	0.54	0.33 [-2684.15, 2684.81]		[redacted]
					TMLE truncated at 200	3.92	2.35	1.57 [1.31, 1.82]	64	
			2.36	1.18e+27	IPW untruncated	0.81	0.54	0.27 [-0.05, 0.59]		100.34 [-140.67, 341.36]
					IPW truncated at 20	3.73	1.71	2.02 [1.23, 2.81]	50	2.22 [1.17, 3.27]
					IPW truncated at 99th	3.69	2.39	1.30 [0.25, 2.35]	77	1.51 [0.87, 2.16]
		Logistic model	2.92	9.01e+53	IPW untruncated	0.35	0.49	-0.14 [-0.48, 0.21]		0.17 [-0.41, 0.74]
					IPW truncated at 20	4.04	1.51	2.53 [1.85, 3.22]	39	1.94 [0.78, 3.10]
					IPW truncated at 99th	3.81	2.19	1.62 [0.78, 2.46]	62	1.41 [0.79, 2.03]

* Discontinuation refers to the interruption of the comparator medication initiated on index date; Crossover refers to the initiation of the comparator medication initiated by patient at baseline in the other arm; MBS occurrence refers to patient's undergoing metabolic bariatric surgery (MBS).

© 2025 Neugebauer R et al. *JAMA Network Open*.

eFigure 19. MACE (Primary Definition), 2-Arm Drug Class Comparison, Sulfonylureas vs GLP-1RAs, ASCVD Subgroup, Cumulative Incidence Curves From PP and ITT Analyses With IPW, TMLE, and SL
Each plot emulates inferences among patients with ASCVD from a 2-arm RCT comparing SU and GLP-1RA and represents unadjusted or adjusted estimates of cumulative incidence curves for MACE derived with IPW and TMLE with SL estimates of propensity scores with four weight truncation schemes: IPW and TMLE without weight truncation (untruncated), IPW with truncation of stabilized weights at value 20 (trunc20) or at the 99th percentile of weight values (trunc99), and TMLE with truncation of unstabilized weights at value 200 (trunc200). The red divider line separates results of Per-Protocol (PP) analyses (top half) from Intention-To-Treat (ITT) analyses (bottom half). Each plot displays a p value for the test that the average risk difference (ARD) through 2.5 years of follow-up (30 months) is 0.

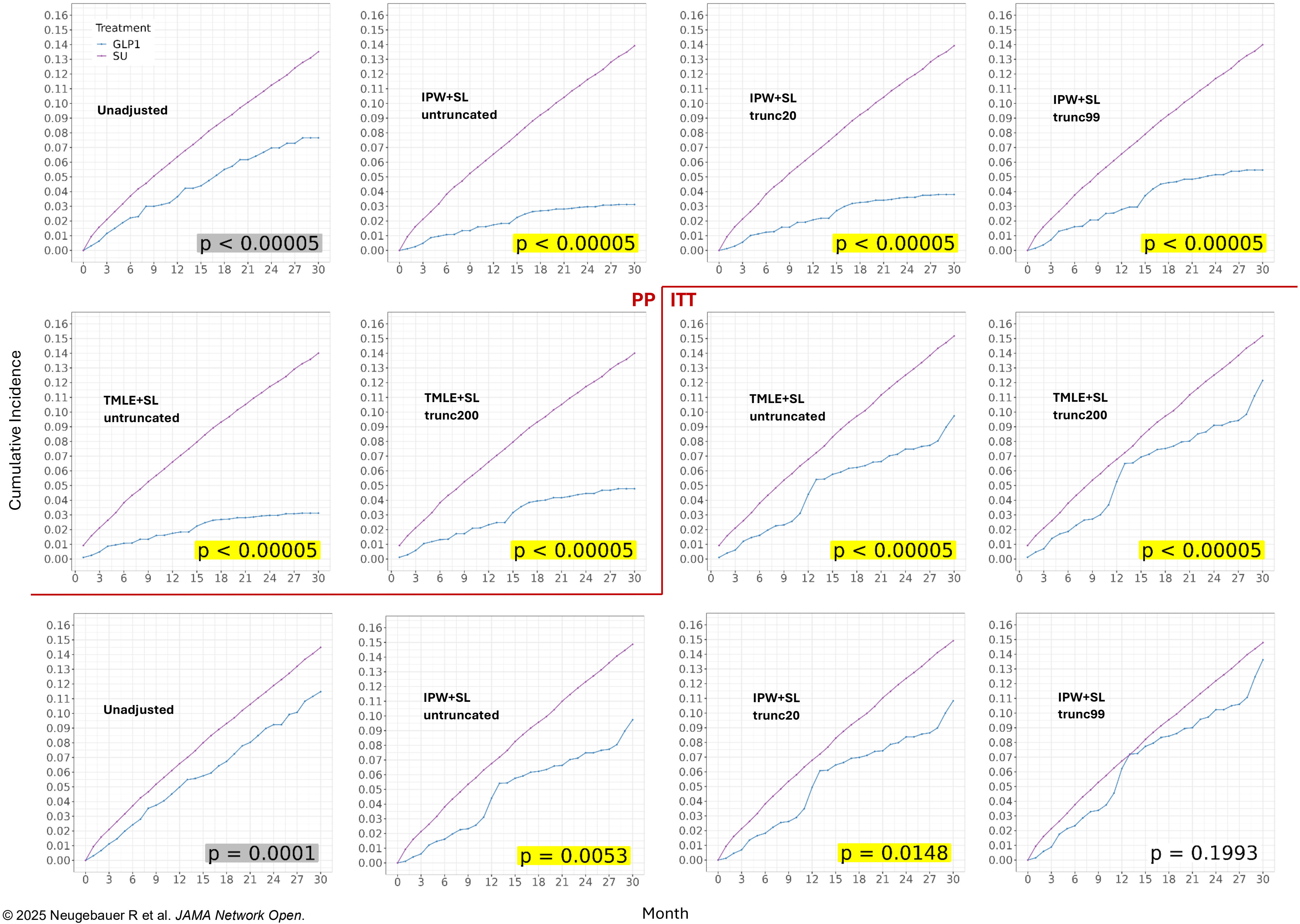

© 2025 Neugebauer R et al. *JAMA Network Open*.

eTable 21. MACE (Primary Definition), 2-Arm Drug Class Comparison, Sulfonylureas vs GLP-1RAs, ASCVD Subgroup, RD and HR Effect Measures at 2.5 Years

Estimation results among patients with ASCVD from ITT and PP analyses of emulated 2-arm RCTs comparing MACE risks over 2.5 years between SU and GLP-1RA initiators. For PP analyses, rates of protocol deviations are described by medication class initiated at baseline. Unadjusted point and interval estimates and adjusted point and interval IPW and TMLE estimates of risks, risk differences (RD), and hazard ratios (HR) based on propensity scores (PS) estimated with either logistic models or super learning (SL) are presented for four weight truncation schemes along with the corresponding 99th percentile and maximum value of the stabilized and unstabilized inverse probability weights used for implementing IPW and TMLE, respectively. RD is the risk in treatment arm minus the risk in control arm and NNT is the number needed to treat.

Analysis type	Protocol Deviations* by exposure group (%)	PS estimation	99th IP weights	Max IP weight	Estimator	Treatment (SU) risk in %	Control (GLP-1RA) risk in %	RD [95% CI] in %	NNT	HR [95% CI]
PP	Discontinuation<br>SU: 48.76<br>GLP-1RA: 45.76<br><br>Crossover<br>SU: 1.46<br>GLP-1RA: 3.46	SL	[redacted]		Unadjusted	13.52	7.66	5.86 [3.65, 8.07]	17	1.75 [1.28, 2.22]
			8.91	3,558.91	TMLE untruncated	14.01	3.12	10.89 [9.92, 11.86]	9	[redacted]
					TMLE truncated at 200	14.01	4.78	9.24 [8.45, 10.02]	11	
			2.17	90.58	IPW untruncated	13.92	3.12	10.80 [8.69, 12.91]	9	3.71 [1.64, 5.78]
					IPW truncated at 20	13.93	3.80	10.13 [8.11, 12.15]	10	3.12 [1.72, 4.53]
					IPW truncated at 99th	13.99	5.47	8.52 [6.11, 10.94]	12	2.31 [1.39, 3.24]
		Logistic model	3.02	560.86	IPW untruncated	13.46	1.20	12.27 [10.76, 13.77]	8	7.90 [-0.23, 16.03]
					IPW truncated at 20	13.78	3.07	10.71 [8.81, 12.61]	9	3.34 [1.27, 5.40]
					IPW truncated at 99th	13.95	4.65	9.30 [6.91, 11.68]	11	2.35 [0.98, 3.71]
ITT	[redacted]	SL	[redacted]		Unadjusted	14.49	11.48	3.01 [0.99, 5.04]	33	1.42 [1.13, 1.71]
			7.09	1,290.23	TMLE untruncated	15.18	9.74	5.44 [3.78, 7.10]	18	[redacted]
					TMLE truncated at 200	15.18	12.14	3.04 [1.74, 4.35]	33	
			2.14	192.16	IPW untruncated	14.88	9.74	5.14 [0.12, 10.16]	19	1.67 [0.83, 2.51]
					IPW truncated at 20	14.92	10.83	4.09 [-0.56, 8.75]		1.48 [0.83, 2.13]
					IPW truncated at 99th	14.79	13.62	1.17 [-3.58, 5.92]		1.21 [0.76, 1.66]
		Logistic model	2.73	552.76	IPW untruncated	14.24	8.26	5.98 [-2.41, 14.36]		1.95 [0.01, 3.89]
					IPW truncated at 20	14.55	15.58	-1.03 [-10.88, 8.82]		1.06 [0.31, 1.81]
					IPW truncated at 99th	14.57	15.34	-0.77 [-7.70, 6.15]		1.14 [0.67, 1.6]

* Discontinuation refers to the interruption of the comparator medication initiated on index date; Crossover refers to the initiation of the comparator medication initiated by patient at baseline in the other arm.

© 2025 Neugebauer R et al. *JAMA Network Open*.

eFigure 20. MACE (Primary Definition), 2-Arm Drug Class Comparison, Sulfonylureas vs GLP-1RAs, No ASCVD Subgroup, Cumulative Incidence Curves From PP and ITT Analyses With IPW, TMLE, and SL
Each plot emulates inferences among patients with No ASCVD from a 2-arm RCT comparing SU and GLP-1RA and represents unadjusted or adjusted estimates of cumulative incidence curves for MACE derived with IPW and TMLE with SL estimates of propensity scores with four weight truncation schemes: IPW and TMLE without weight truncation (untruncated), IPW with truncation of stabilized weights at value 20 (trunc20) or at the 99th percentile of weight values (trunc99), and TMLE with truncation of unstabilized weights at value 200 (trunc200). The red divider line separates results of Per-Protocol (PP) analyses (top half) from Intention-To-Treat (ITT) analyses (bottom half). Each plot displays a p value for the test that the average risk difference (ARD) through 2.5 years of follow-up (30 months) is 0.

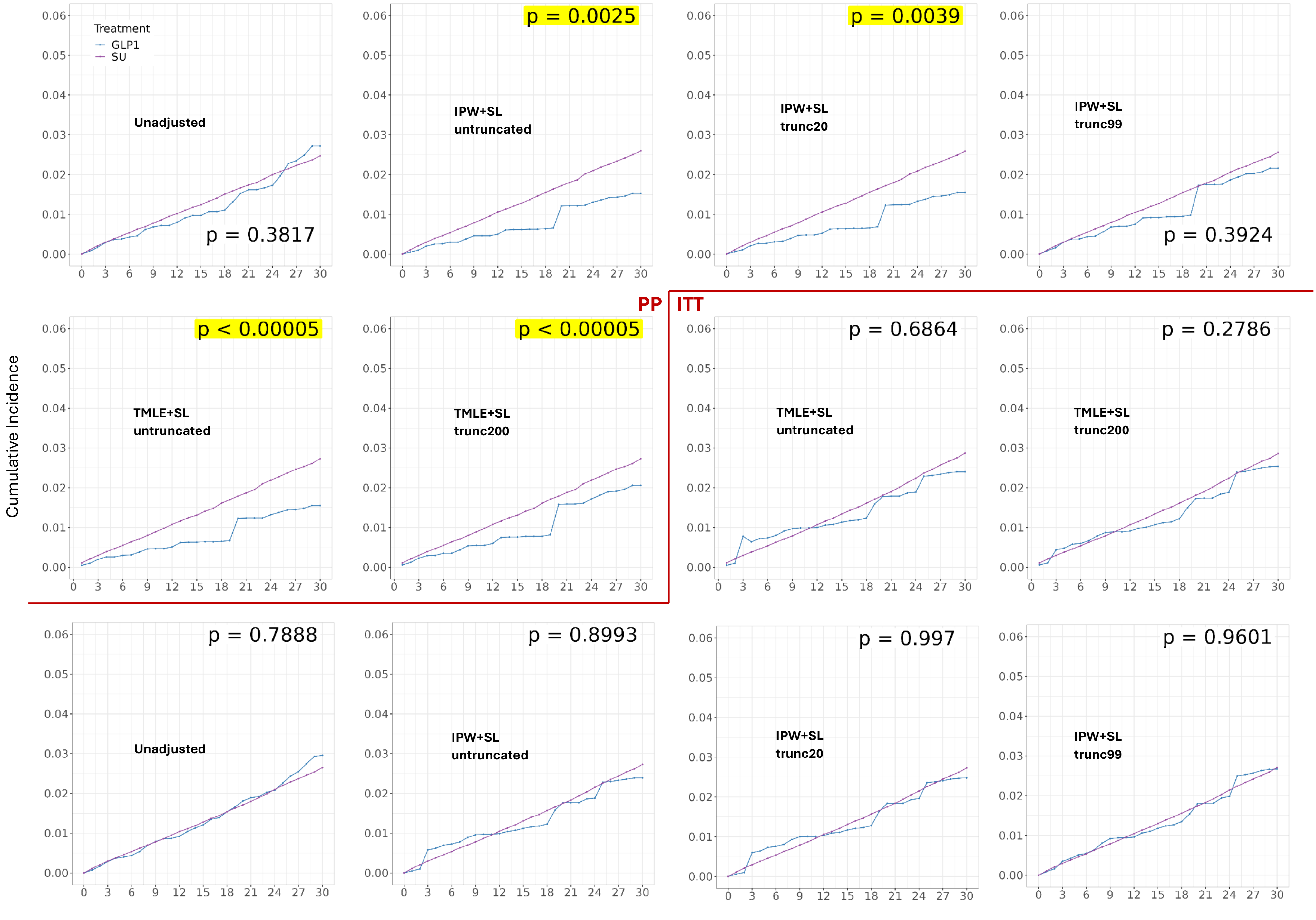

© 2025 Neugebauer R et al. *JAMA Network Open*.

eTable 22. MACE (Primary Definition), 2-Arm Drug Class Comparison, Sulfonylureas vs GLP-1RAs, No ASCVD Subgroup, RD and HR Effect Measures at 2.5 Years

Estimation results among patients with No ASCVD from ITT and PP analyses of emulated 2-arm RCTs comparing MACE risks over 2.5 years between SU and GLP-1RA initiators. For PP analyses, rates of protocol deviations are described by medication class initiated at baseline. Unadjusted point and interval estimates and adjusted point and interval IPW and TMLE estimates of risks, risk differences (RD), and hazard ratios (HR) based on propensity scores (PS) estimated with either logistic models or super learning (SL) are presented for four weight truncation schemes along with the corresponding 99th percentile and maximum value of the stabilized and unstabilized inverse probability weights used for implementing IPW and TMLE, respectively. RD is the risk in treatment arm minus the risk in control arm and NNT is the number needed to treat.

Analysis type	Protocol Deviations* by exposure group (%)	PS estimation	99th IP weights	Max IP weight	Estimator	Treatment (SU) risk in %	Control (GLP-1RA) risk in %	RD [95% CI] in %	NNT	HR [95% CI]
PP	Discontinuation<br>SU: 49.86<br>GLP-1RA: 43.39<br><br>Crossover<br>SU: 1.57<br>GLP-1RA: 3.81	SL			Unadjusted	2.47	2.72	-0.25 [-0.92, 0.42]		1.27 [0.95, 1.58]
			9.65	2,554.28	TMLE untruncated	2.73	1.55	1.18 [0.90, 1.45]	85	
					TMLE truncated at 200	2.73	2.06	0.67 [0.40, 0.93]	150	
			2.48	556.16	IPW untruncated	2.60	1.53	1.07 [0.16, 1.98]	93	2.03 [0.96, 3.09]
					IPW truncated at 20	2.59	1.55	1.04 [0.12, 1.95]	96	1.96 [0.93, 2.98]
					IPW truncated at 99th	2.56	2.16	0.40 [-0.81, 1.62]		1.35 [0.68, 2.02]
		Logistic model	3.31	596,936.40	IPW untruncated	2.07	1.23	0.85 [0.14, 1.55]	118	1.99 [0.58, 3.40]
					IPW truncated at 20	2.72	1.39	1.34 [0.61, 2.06]	75	1.61 [0.51, 2.71]
					IPW truncated at 99th	2.63	1.96	0.67 [-0.27, 1.60]		1.25 [0.60, 1.90]
ITT		SL			Unadjusted	2.65	2.96	-0.31 [-0.78, 0.17]		1.05 [0.84, 1.25]
			7.04	3,177.52	TMLE untruncated	2.87	2.40	0.46 [-0.09, 1.02]		
					TMLE truncated at 200	2.86	2.54	0.32 [-0.06, 0.70]		
			2.18	604.05	IPW untruncated	2.73	2.39	0.34 [-0.91, 1.59]		1.08 [0.20, 1.97]
					IPW truncated at 20	2.73	2.48	0.26 [-1.02, 1.53]		1.04 [0.20, 1.88]
					IPW truncated at 99th	2.71	2.67	0.04 [-0.95, 1.03]		1.09 [0.67, 1.51]
		Logistic model	2.90	371,122.50	IPW untruncated	1.95	2.09	-0.14 [-1.27, 0.99]		1.10 [0.23, 1.98]
					IPW truncated at 20	2.74	2.35	0.39 [-0.76, 1.54]		0.96 [0.28, 1.64]
					IPW truncated at 99th	2.72	2.59	0.13 [-0.81, 1.07]		1.01 [0.60, 1.42]

* Discontinuation refers to the interruption of the comparator medication initiated on index date; Crossover refers to the initiation of the comparator medication initiated by patient at baseline in the other arm.

© 2025 Neugebauer R et al. *JAMA Network Open*.

eFigure 21. MACE (Primary Definition), 2-Arm Drug Class Comparison, Sulfonylureas vs GLP-1RAs, No ASCVD and MET Subgroup, Cumulative Incidence Curves From PP and ITT Analyses With IPW, TMLE, and SL
Each plot emulates inferences among patients with No ASCVD and MET from a 2-arm RCT comparing SU and GLP-1RA and represents unadjusted or adjusted estimates of cumulative incidence curves for MACE derived with IPW and TMLE with SL estimates of propensity scores with four weight truncation schemes: IPW and TMLE without weight truncation (untruncated), IPW with truncation of stabilized weights at value 20 (trunc20) or at the 99th percentile of weight values (trunc99), and TMLE with truncation of unstabilized weights at value 200 (trunc200). The red divi der line separates results of Per-Protocol (PP) analyses (top half) from Intention-To-Treat (ITT) analyses (bottom half). Each plot displays a p value for the test that the average risk difference (ARD) through 2.5 years of follow-up (30 months) is 0.

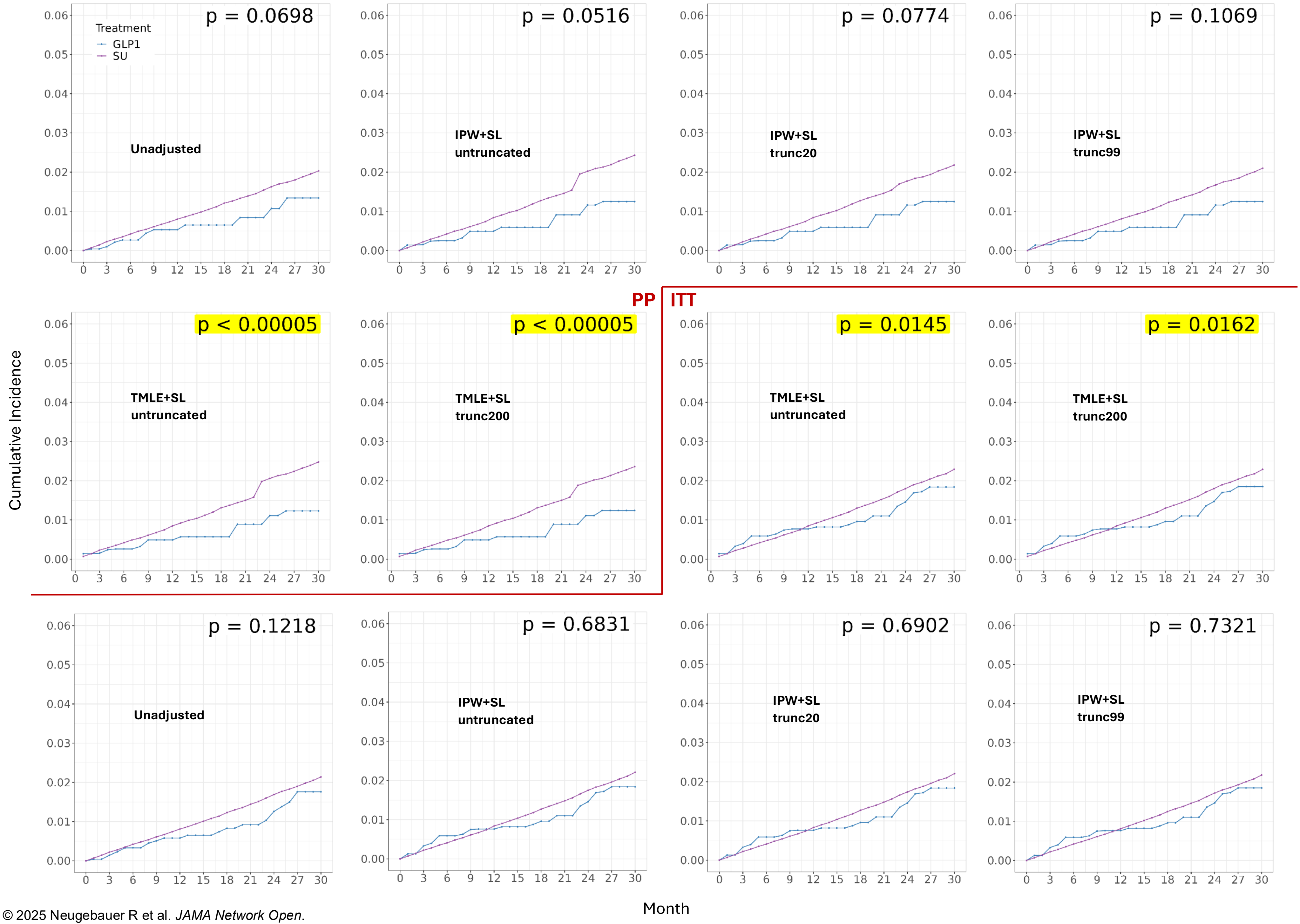

© 2025 Neugebauer R et al. *JAMA Network Open*.

eTable 23. MACE (Primary Definition), 2-Arm Drug Class Comparison, Sulfonylureas vs GLP-1RAs, No ASCVD and MET Subgroup, RD and HR Effect Measures at 2.5 Years

Estimation results among patients with No ASCVD and MET from ITT and PP analyses of emulated 2-arm RCTs comparing MACE risks over 2.5 years between SU and GLP-1RA initiators. For PP analyses, rates of protocol deviations are described by medication class initiated at baseline. Unadjusted point and interval estimates and adjusted point and interval IPW and TMLE estimates of risks, risk differences (RD), and hazard ratios (HR) based on propensity scores (PS) estimated with either logistic models or super learning (SL) are presented for four weight truncation schemes along with the corresponding 99th percentile and maximum value of the stabilized and unstabilized inverse probability weights used for implementing IPW and TMLE, respectively. RD is the risk in treatment arm minus the risk in control arm and NNT is the number needed to treat.

Analysis type	Protocol Deviations* by exposure group (%)	PS estimation	99th IP weights	Max IP weight	Estimator	Treatment (SU) risk in %	Control (GLP-1RA) risk in %	RD [95% CI] in %	NNT	HR [95% CI]
PP	<u>Discontinuation</u><br>SU: 45.79<br>GLP-1RA: 39.34<br><br><u>Crossover</u><br>SU: 1.52<br>GLP-1RA: 5.28	SL	[redacted]		Unadjusted	2.03	1.34	0.69 [-0.22, 1.60]		1.60 [0.55, 2.65]
			6.91	2,695.92	TMLE untruncated	2.48	1.23	1.25 [0.75, 1.74]	80	[redacted]
					TMLE truncated at 200	2.36	1.24	1.13 [0.78, 1.47]	89	
			2.14	174.95	IPW untruncated	2.43	1.25	1.18 [0.04, 2.32]	85	1.62 [0.01, 3.23]
					IPW truncated at 20	2.18	1.25	0.93 [-0.05, 1.91]		1.62 [0.01, 3.23]
					IPW truncated at 99th	2.10	1.25	0.85 [-0.12, 1.82]		1.58 [0.01, 3.15]
		Logistic model	3.02	4.72e+13	IPW untruncated	0.48	0.49	-0.01 [-0.48, 0.46]		0.55 [-0.32, 1.42]
					IPW truncated at 20	2.27	0.54	1.73 [1.17, 2.29]	58	1.98 [-1.00, 4.97]
					IPW truncated at 99th	2.11	0.81	1.31 [0.59, 2.03]	76	1.41 [-0.62, 3.44]
ITT	[redacted]	SL	[redacted]		Unadjusted	2.14	1.76	0.38 [-0.41, 1.17]		1.48 [0.67, 2.29]
			3.79	1,733.13	TMLE untruncated	2.29	1.84	0.46 [0.28, 0.64]	219	[redacted]
					TMLE truncated at 200	2.29	1.85	0.45 [0.27, 0.62]	224	
			1.65	93.27	IPW untruncated	2.21	1.84	0.37 [-0.65, 1.39]		1.10 [0.21, 1.99]
					IPW truncated at 20	2.21	1.84	0.37 [-0.65, 1.39]		1.09 [0.21, 1.98]
					IPW truncated at 99th	2.18	1.85	0.33 [-0.70, 1.35]		1.07 [0.20, 1.94]
		Logistic model	2.46	1.05e+15	IPW untruncated	0.54	1.70	-1.16 [-2.64, 0.32]		0.12 [-0.03, 0.28]
					IPW truncated at 20	2.18	1.92	0.26 [-1.39, 1.91]		0.74 [-0.12, 1.60]
					IPW truncated at 99th	2.16	2.19	-0.03 [-1.75, 1.70]		0.85 [0.02, 1.68]

* Discontinuation refers to the interruption of the comparator medication initiated on index date; Crossover refers to the initiation of the comparator medication initiated by patient at baseline in the other arm.

© 2025 Neugebauer R et al. *JAMA Network Open*.

eFigure 22. MACE (Primary Definition), 2-Arm Drug Class Comparison, DPP4is vs SGLT2is, CONSORT Diagram
Flow diagram describing the inclusion and exclusion steps and counts leading to the creation of the cohort for emulating the 2-arm RCT to compare the risk of MACE in new users of DPP4i and SGLT2i along with sample sizes and counts for each observed end of follow-up type by treatment initiated at cohort entry.

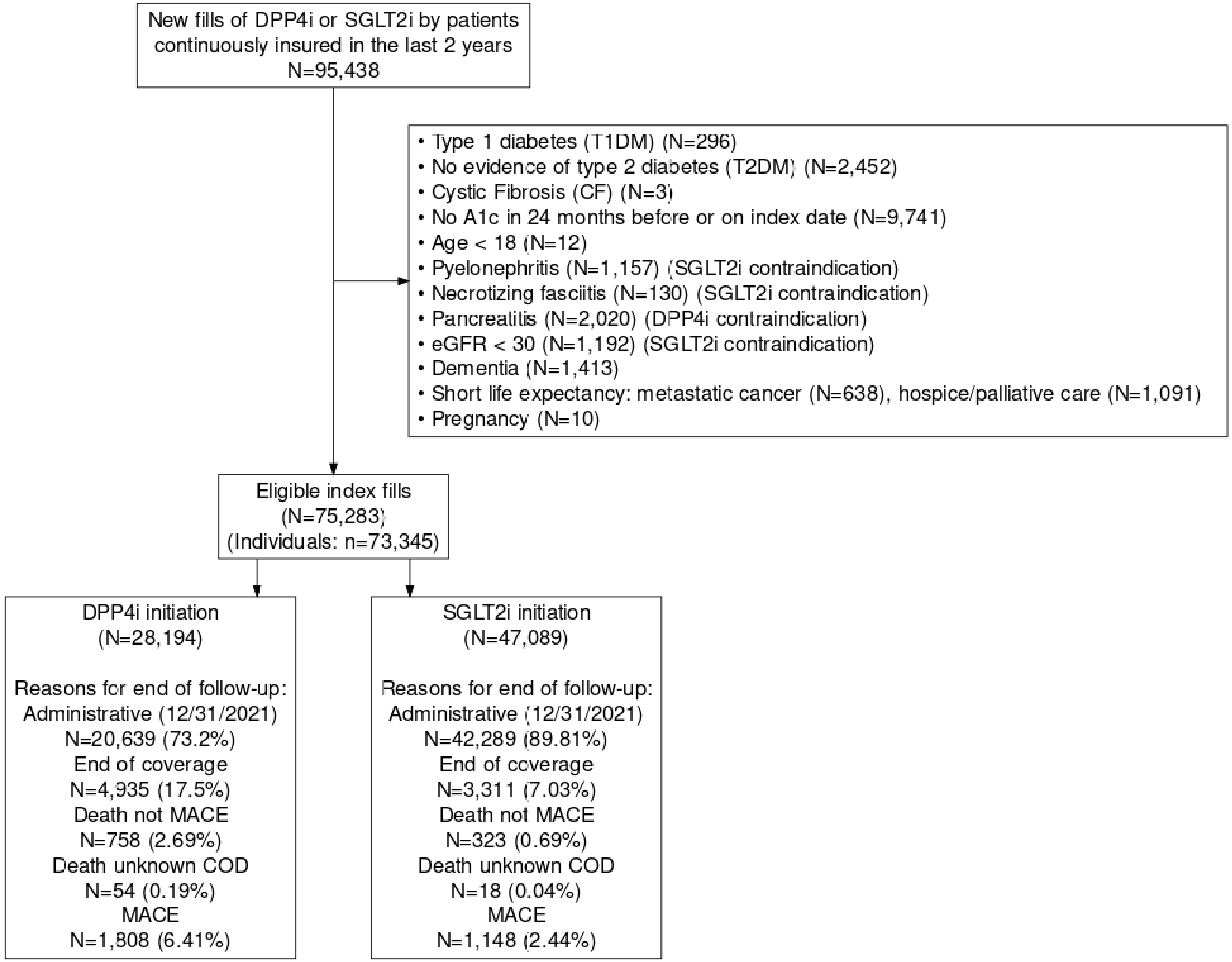

© 2025 Neugebauer R et al. *JAMA Network Open*.

eTable 24. MACE (Primary Definition), 2-Arm Drug Class Comparison, DPP4is vs SGLT2is, Patient Characteristics at Baseline (Overall and by Medication Initiated)

Summary statistics of the baseline values for selected covariates in the cohort of patients used to emulate a 2-arm RCT for comparing DPP4i and SGLT2i. For each continuous variable, the mean and standard deviation are displayed for all patients in the cohort (last column) and by drug class initiated at cohort entry. For each categorical variable and for each possible level of that variable, the count and proportion are displayed instead.

	DPP4i n = 28,194	SGLT2i n = 47,089	Total n = 75,283
**Demographics**			
Age	61.15 (11.95)	60.11 (11.53)	60.50 (11.70)
Agegrp			
<45	2,349 (8.33%)	4,563 (9.69%)	6,912 (9.18%)
[45-65)	14,803 (52.5%)	25,050 (53.2%)	39,853 (52.94%)
[65-75)	7,373 (26.15%)	13,130 (27.88%)	20,503 (27.23%)
>=75	3,669 (13.01%)	4,346 (9.23%)	8,015 (10.65%)
Ethnicity			
Hispanic	8,410 (29.83%)	12,976 (27.56%)	21,386 (28.41%)
Nonhispanic	19,784 (70.17%)	34,113 (72.44%)	53,897 (71.59%)
Female Head Of Hh	0.147 (0.073)	0.139 (0.071)	0.142 (0.072)
Missing	885 (3.139%)	905 (1.922%)	1,790 (2.378%)
Hh Public Assistance	0.037 (0.034)	0.036 (0.033)	0.036 (0.033)
Missing	885 (3.139%)	905 (1.922%)	1,790 (2.378%)
Household Income Less 30k	0.218 (0.125)	0.176 (0.110)	0.191 (0.117)
Missing	885 (3.139%)	905 (1.922%)	1,790 (2.378%)
Houspoverty	0.106 (0.092)	0.083 (0.077)	0.092 (0.083)
Missing	885 (3.139%)	906 (1.924%)	1,791 (2.379%)
Index Yr			
2014	1,657 (5.88%)	173 (0.37%)	1,830 (2.43%)
2015	3,372 (11.96%)	518 (1.1%)	3,890 (5.17%)
2016	5,387 (19.11%)	1,254 (2.66%)	6,641 (8.82%)
2017	5,163 (18.31%)	1,998 (4.24%)	7,161 (9.51%)
2018	3,965 (14.06%)	2,913 (6.19%)	6,878 (9.14%)
2019	3,797 (13.47%)	4,583 (9.73%)	8,380 (11.13%)
2020	2,581 (9.15%)	11,616 (24.67%)	14,197 (18.86%)
2021	2,272 (8.06%)	24,034 (51.04%)	26,306 (34.94%)
Bmi	32.35 (7.12)	33.72 (7.45)	33.20 (7.35)
Missing	572 (2.03%)	1,972 (4.19%)	2,544 (3.38%)
Smoking Status			
Formersmoker	8,411 (29.83%)	14,848 (31.53%)	23,259 (30.9%)
Currentsmoker	2,275 (8.07%)	3,560 (7.56%)	5,835 (7.75%)
Passivesmoker	129 (0.46%)	196 (0.42%)	325 (0.43%)
Neversmoker	17,112 (60.69%)	27,841 (59.12%)	44,953 (59.71%)
Unknown	267 (0.95%)	644 (1.37%)	911 (1.21%)
Low Educ	0.171 (0.132)	0.147 (0.116)	0.156 (0.123)
Missing	885 (3.139%)	904 (1.92%)	1,789 (2.376%)
Mgr Male	0.043 (0.041)	0.051 (0.050)	0.048 (0.047)
Missing	885 (3.139%)	906 (1.924%)	1,791 (2.379%)
Ndi	0.28 (0.17)	0.24 (0.15)	0.26 (0.16)
Missing	885 (3.14%)	907 (1.93%)	1,792 (2.38%)
Pct Crowding	0.082 (0.086)	0.081 (0.080)	0.082 (0.082)
Missing	885 (3.139%)	905 (1.922%)	1,790 (2.378%)
Racegrp			
White	15,075 (53.47%)	23,304 (49.49%)	38,379 (50.98%)
Asian	4,662 (16.54%)	8,973 (19.06%)	13,635 (18.11%)
Blackorafricanamerican	2,837 (10.06%)	4,328 (9.19%)	7,165 (9.52%)
Hawaiianorpacificislander	386 (1.37%)	1,036 (2.2%)	1,422 (1.89%)
Americanindianoralaskanative	174 (0.62%)	309 (0.66%)	483 (0.64%)
Multirace	1,014 (3.6%)	1,914 (4.06%)	2,928 (3.89%)
Other	75 (0.27%)	105 (0.22%)	180 (0.24%)
Unknown	3,971 (14.08%)	7,120 (15.12%)	11,091 (14.73%)

© 2025 Neugebauer R et al. *JAMA Network Open*.

	DPP4i n = 28,194	SGLT2i n = 47,089	Total n = 75,283
Sex			
Female	13,579 (48.16%)	20,222 (42.94%)	33,801 (44.9%)
Male	14,614 (51.83%)	26,866 (57.05%)	41,480 (55.1%)
Other	1 (0%)	0 (0%)	1 (0%)
Unknown	0 (0%)	1 (0%)	1 (0%)
Site			
Kpnc	4,854 (17.22%)	18,128 (38.5%)	22,982 (30.53%)
Kpsc	16,553 (58.71%)	18,990 (40.33%)	35,543 (47.21%)
Kphi	830 (2.94%)	3,496 (7.42%)	4,326 (5.75%)
Hpi	1,420 (5.04%)	1,829 (3.88%)	3,249 (4.32%)
Hfhs	1,977 (7.01%)	1,391 (2.95%)	3,368 (4.47%)
Ghs	2,560 (9.08%)	3,255 (6.91%)	5,815 (7.72%)
Unemployment	0.049 (0.027)	0.039 (0.023)	0.042 (0.025)
Missing	885 (3.139%)	903 (1.918%)	1,788 (2.375%)
**Insurance**			
Ins Commercial	17,720 (62.85%)	29,155 (61.91%)	46,875 (62.27%)
Ins Highdeductible	1,804 (6.4%)	2,682 (5.7%)	4,486 (5.96%)
Ins Medicaid	2,607 (9.25%)	4,675 (9.93%)	7,282 (9.67%)
Ins Medicare	10,457 (37.09%)	16,656 (35.37%)	27,113 (36.01%)
Ins Medicare A	5,488 (19.47%)	11,116 (23.61%)	16,604 (22.06%)
Ins Medicare B	5,179 (18.37%)	10,715 (22.75%)	15,894 (21.11%)
Ins Medicare C	4,424 (15.69%)	9,440 (20.05%)	13,864 (18.42%)
Ins Medicare D	4,690 (16.63%)	10,394 (22.07%)	15,084 (20.04%)
Ins Other Coverage	7,679 (27.24%)	10,371 (22.02%)	18,050 (23.98%)
Ins Privatepay	5,190 (18.41%)	7,288 (15.48%)	12,478 (16.57%)
Ins Selffunded	749 (2.66%)	1,274 (2.71%)	2,023 (2.69%)
Ins Statesubsidized	574 (2.04%)	997 (2.12%)	1,571 (2.09%)
**Clinical data**			
A1c Age	41.96 (74.87)	44.45 (69.04)	43.52 (71.29)
Missing	19 (0.07%)	24 (0.05%)	43 (0.06%)
Acc Aha 201310yrcvdrisk	0.197 (0.161)	0.193 (0.154)	0.195 (0.157)
Missing	2,521 (8.942%)	6,383 (13.555%)	8,904 (11.827%)
Chf Dx Status			
0	26,540 (94.13%)	41,584 (88.31%)	68,124 (90.49%)
1	1,483 (5.26%)	5,077 (10.78%)	6,560 (8.71%)
999	171 (0.61%)	428 (0.91%)	599 (0.8%)
Cv Risk Subgrp			
Low	9,138 (32.41%)	14,074 (29.89%)	23,212 (30.83%)
Moderate	6,112 (21.68%)	9,553 (20.29%)	15,665 (20.81%)
High	9,704 (34.42%)	14,955 (31.76%)	24,659 (32.76%)
Other	776 (2.75%)	2,291 (4.87%)	3,067 (4.07%)
Unknown	2,464 (8.74%)	6,216 (13.2%)	8,680 (11.53%)
Diab Duration	6.60 (2.84)	8.10 (3.59)	7.54 (3.41)
Missing	33 (0.12%)	0 (0%)	33 (0.04%)
A1c	8.58 (1.49)	8.77 (1.60)	8.70 (1.57)
Missing	19 (0.07%)	24 (0.05%)	43 (0.06%)
Acr	50.21 (90.63)	74.57 (110.57)	65.41 (104.19)
Missing	3,512 (12.46%)	6,138 (13.03%)	9,650 (12.82%)
Afib Dx	1,533 (5.44%)	3,534 (7.5%)	5,067 (6.73%)
Alt	30.83 (20.44)	30.62 (21.77)	30.70 (21.26)
Missing	4,939 (17.52%)	10,631 (22.58%)	15,570 (20.68%)
Amputation Dxpx	218 (0.77%)	601 (1.28%)	819 (1.09%)
Anemia Dx	2,674 (9.48%)	4,957 (10.53%)	7,631 (10.14%)
Anxiety Dx	5,390 (19.12%)	9,537 (20.25%)	14,927 (19.83%)
Arrythmia Dx	1,474 (5.23%)	3,217 (6.83%)	4,691 (6.23%)
Ascvd Dxpx Max	3,623 (12.85%)	7,674 (16.3%)	11,297 (15.01%)
Ascvd Dxpx Ppv	776 (2.75%)	2,291 (4.87%)	3,067 (4.07%)

© 2025 Neugebauer R et al. *JAMA Network Open*.

	DPP4i n = 28,194	SGLT2i n = 47,089	Total n = 75,283
Asthma Dx	2,796 (9.92%)	5,836 (12.39%)	8,632 (11.47%)
Bariatric Px	315 (1.12%)	518 (1.1%)	833 (1.11%)
Bipolar Dx	259 (0.92%)	452 (0.96%)	711 (0.94%)
Blind Dx	132 (0.47%)	102 (0.22%)	234 (0.31%)
Cad Dxpx Max	2,345 (8.32%)	5,209 (11.06%)	7,554 (10.03%)
Cad Dxpx Ppv	496 (1.76%)	1,729 (3.67%)	2,225 (2.96%)
Cad Dxpx Sens	578 (2.05%)	1,872 (3.98%)	2,450 (3.25%)
Cancer Mets Dx	0 (0%)	0 (0%)	0 (0%)
Cancer Nomets Dx	1,417 (5.03%)	2,357 (5.01%)	3,774 (5.01%)
Cevd Dxpx Ppv	244 (0.87%)	517 (1.1%)	761 (1.01%)
Cevd Dxpx Sens	1,094 (3.88%)	2,405 (5.11%)	3,499 (4.65%)
Chf Dx Ppv	282 (1%)	1,275 (2.71%)	1,557 (2.07%)
Chf Dx Sens	1,483 (5.26%)	5,077 (10.78%)	6,560 (8.71%)
Ckd Dx	8,533 (30.27%)	16,891 (35.87%)	25,424 (33.77%)
Copd Dx	1,305 (4.63%)	2,431 (5.16%)	3,736 (4.96%)
Coupled Dbp	72.49 (10.42)	72.88 (11.05)	72.73 (10.82)
Missing	463 (1.64%)	867 (1.84%)	1,330 (1.77%)
Coupled Sbp	128.92 (13.94)	130.17 (14.70)	129.70 (14.43)
Missing	463 (1.64%)	867 (1.84%)	1,330 (1.77%)
Covid Prd	4,853 (17.21%)	35,650 (75.71%)	40,503 (53.8%)
Creat	0.93 (0.30)	0.95 (0.30)	0.94 (0.30)
Missing	404 (1.43%)	469 (1%)	873 (1.16%)
Cysticfibrosis Dx	0 (0%)	0 (0%)	0 (0%)
Dbp	71.82 (10.45)	71.98 (11.16)	71.92 (10.90)
Missing	463 (1.64%)	867 (1.84%)	1,330 (1.77%)
Dementia Dx	0 (0%)	0 (0%)	0 (0%)
Depr Dx	4,379 (15.53%)	6,512 (13.83%)	10,891 (14.47%)
Dietitian	1,817 (6.44%)	3,678 (7.81%)	5,495 (7.3%)
Dka Dx	270 (0.96%)	619 (1.31%)	889 (1.18%)
Dka Dx Count	0.01 (0.11)	0.02 (0.15)	0.01 (0.14)
Esrd Dx	1,960 (6.95%)	4,922 (10.45%)	6,882 (9.14%)
Esrd Px	91 (0.32%)	81 (0.17%)	172 (0.23%)
Etoh Dx	396 (1.4%)	827 (1.76%)	1,223 (1.62%)
Fasciitis Dx	0 (0%)	0 (0%)	0 (0%)
Fpg	170.44 (59.58)	174.78 (66.70)	172.78 (63.56)
Missing	21,035 (74.61%)	38,709 (82.2%)	59,744 (79.36%)
Frailty Dx	2,777 (9.85%)	4,426 (9.4%)	7,203 (9.57%)
Gfr Epi 09	82.83 (22.85)	82.01 (22.20)	82.31 (22.45)
Missing	404 (1.43%)	469 (1%)	873 (1.16%)
Hdl	44.41 (11.47)	43.31 (11.25)	43.73 (11.34)
Missing	3,093 (10.97%)	6,817 (14.48%)	9,910 (13.16%)
Hgb	13.60 (1.57)	13.69 (1.59)	13.66 (1.58)
Missing	7,246 (25.7%)	12,392 (26.32%)	19,638 (26.09%)
Htn Dx	19,182 (68.04%)	32,885 (69.84%)	52,067 (69.16%)
Hypo Dx	55 (0.2%)	124 (0.26%)	179 (0.24%)
Hypo Dx Count	0.01 (0.15)	0.02 (0.20)	0.02 (0.18)
Hypo Dx Event	55 (0.2%)	124 (0.26%)	179 (0.24%)
Hypothyroidism Dx	3,668 (13.01%)	5,661 (12.02%)	9,329 (12.39%)
Ldl	84.99 (33.68)	82.43 (35.21)	83.42 (34.65)
Missing	2,276 (8.07%)	5,965 (12.67%)	8,241 (10.95%)
Leukemia Lymphoma Dx	178 (0.63%)	286 (0.61%)	464 (0.62%)
Lipid Dx	20,917 (74.19%)	33,795 (71.77%)	54,712 (72.68%)
Liver Dx	68 (0.24%)	61 (0.13%)	129 (0.17%)
Mci Dx	191 (0.68%)	238 (0.51%)	429 (0.57%)
Men2 Dx	1 (0%)	0 (0%)	1 (0%)
Nephropathy Dx	2,539 (9.01%)	1,318 (2.8%)	3,857 (5.12%)
Neuro Dx	756 (2.68%)	1,424 (3.02%)	2,180 (2.9%)

© 2025 Neugebauer R et al. *JAMA Network Open*.

	DPP4i n = 28,194	SGLT2i n = 47,089	Total n = 75,283
Pancreatitis Dx	0 (0%)	0 (0%)	0 (0%)
Pcr	0.000 (0.000)	0.000 (0.000)	0.000 (0.000)
Missing	26,576 (94.261%)	40,755 (86.549%)	67,331 (89.437%)
Potassium	4.29 (0.41)	4.29 (0.41)	4.29 (0.41)
Missing	934 (3.31%)	1,631 (3.46%)	2,565 (3.41%)
Pregnancy	0 (0%)	0 (0%)	0 (0%)
Pud Dx	37 (0.13%)	49 (0.1%)	86 (0.11%)
Pvd Dxpx Ppv	91 (0.32%)	145 (0.31%)	236 (0.31%)
Pvd Dxpx Sens	753 (2.67%)	1,468 (3.12%)	2,221 (2.95%)
Pyelo Dx	0 (0%)	0 (0%)	0 (0%)
Retinopathy Dxpx	1,918 (6.8%)	2,028 (4.31%)	3,946 (5.24%)
Rpg	192.35 (84.80)	198.43 (87.14)	196.19 (86.33)
Missing	16,230 (57.57%)	26,656 (56.61%)	42,886 (56.97%)
Sbp	128.33 (13.85)	129.32 (14.70)	128.95 (14.39)
Missing	463 (1.64%)	867 (1.84%)	1,330 (1.77%)
Schiz Dx	185 (0.66%)	255 (0.54%)	440 (0.58%)
Sodium	138.38 (2.86)	138.79 (2.89)	138.63 (2.89)
Missing	3,083 (10.93%)	8,808 (18.71%)	11,891 (15.8%)
Sud Dx	222 (0.79%)	394 (0.84%)	616 (0.82%)
Tc	160.82 (42.78)	159.69 (45.16)	160.12 (44.27)
Missing	3,040 (10.78%)	6,782 (14.4%)	9,822 (13.05%)
Trig	185.43 (141.18)	200.94 (158.09)	194.88 (151.89)
Missing	4,309 (15.28%)	9,915 (21.06%)	14,224 (18.89%)
Tsh	2.04 (1.77)	2.11 (1.84)	2.09 (1.82)
Missing	10,733 (38.07%)	18,248 (38.75%)	28,981 (38.5%)
Valvular Dx	794 (2.82%)	1,597 (3.39%)	2,391 (3.18%)
Vasculitis Dx	392 (1.39%)	405 (0.86%)	797 (1.06%)
Only Met No Ascvd	3,361 (11.92%)	6,086 (12.92%)	9,447 (12.55%)
Renal Function Status			
Lowrisk	15,028 (53.3%)	21,422 (45.49%)	36,450 (48.42%)
Moderaterisk	6,183 (21.93%)	11,532 (24.49%)	17,715 (23.53%)
Highrisk	2,376 (8.43%)	5,473 (11.62%)	7,849 (10.43%)
Veryhighrisk	1,006 (3.57%)	2,449 (5.2%)	3,455 (4.59%)
Unknown	3,601 (12.77%)	6,213 (13.19%)	9,814 (13.04%)
Total Visit C	0.72 (2.63)	1.04 (3.17)	0.92 (2.98)
Total Visit E	0.46 (2.19)	0.53 (2.36)	0.51 (2.30)
Total Visit N	0.25 (2.89)	0.29 (1.59)	0.28 (2.17)
**Concurrent medications**			
Aa	0 (0%)	0 (0%)	0 (0%)
Aceinhibitors	12,616 (44.75%)	20,440 (43.41%)	33,056 (43.91%)
Agi	491 (1.74%)	422 (0.9%)	913 (1.21%)
Anticoagulants	1,364 (4.84%)	3,254 (6.91%)	4,618 (6.13%)
Anticonvulsants	2,413 (8.56%)	5,350 (11.36%)	7,763 (10.31%)
Antidepressantcomb	0 (0%)	1 (0%)	1 (0%)
Antidepressantmaoi	6 (0.02%)	6 (0.01%)	12 (0.02%)
Antidepressantndri	598 (2.12%)	1,162 (2.47%)	1,760 (2.34%)
Antidepressantother	0 (0%)	1 (0%)	1 (0%)
Antidepressantsari	788 (2.79%)	1,561 (3.31%)	2,349 (3.12%)
Antidepressantsnri	1,034 (3.67%)	2,271 (4.82%)	3,305 (4.39%)
Antidepressantspo	17 (0.06%)	40 (0.08%)	57 (0.08%)
Antidepressantssri	2,685 (9.52%)	4,686 (9.95%)	7,371 (9.79%)
Antidepressanttca	915 (3.25%)	1,232 (2.62%)	2,147 (2.85%)
Antidepressantteca	187 (0.66%)	333 (0.71%)	520 (0.69%)
Antiplatelets	2,332 (8.27%)	6,081 (12.91%)	8,413 (11.18%)
Antipsychotic1stgen	77 (0.27%)	85 (0.18%)	162 (0.22%)
Antipsychotic2ndgen	466 (1.65%)	756 (1.61%)	1,222 (1.62%)
Anxiety	381 (1.35%)	801 (1.7%)	1,182 (1.57%)

© 2025 Neugebauer R et al. *JAMA Network Open*.

	DPP4i n = 28,194	SGLT2i n = 47,089	Total n = 75,283
Arb	6,755 (23.96%)	13,646 (28.98%)	20,401 (27.1%)
Benzodiazepines	1,320 (4.68%)	1,508 (3.2%)	2,828 (3.76%)
Betablockers	8,483 (30.09%)	16,824 (35.73%)	25,307 (33.62%)
Clonidine	354 (1.26%)	543 (1.15%)	897 (1.19%)
Dihydropyridineccb	4,908 (17.41%)	9,555 (20.29%)	14,463 (19.21%)
Glp1	321 (1.14%)	2,566 (5.45%)	2,887 (3.83%)
Hypnoticother	290 (1.03%)	369 (0.78%)	659 (0.88%)
Injectableantipsychotic	6 (0.02%)	6 (0.01%)	12 (0.02%)
Ins	4,424 (15.69%)	16,098 (34.19%)	20,522 (27.26%)
Ins Analog	1,589 (5.64%)	4,458 (9.47%)	6,047 (8.03%)
Ins Combo	429 (1.52%)	2,266 (4.81%)	2,695 (3.58%)
Ins Human	3,109 (11.03%)	12,857 (27.3%)	15,966 (21.21%)
Ins La	3,866 (13.71%)	13,132 (27.89%)	16,998 (22.58%)
Ins Sa	1,442 (5.11%)	6,195 (13.16%)	7,637 (10.14%)
Ksparingdiuretics	1,060 (3.76%)	2,778 (5.9%)	3,838 (5.1%)
Lithium	35 (0.12%)	57 (0.12%)	92 (0.12%)
Loopdiuretics	2,070 (7.34%)	5,312 (11.28%)	7,382 (9.81%)
Meg	156 (0.55%)	139 (0.3%)	295 (0.39%)
Met	22,167 (78.62%)	37,357 (79.33%)	59,524 (79.07%)
Nondihydropyridineccb	671 (2.38%)	984 (2.09%)	1,655 (2.2%)
Otherlipidmeds	1,216 (4.31%)	2,019 (4.29%)	3,235 (4.3%)
Pcsk9mab	8 (0.03%)	65 (0.14%)	73 (0.1%)
Statins	20,522 (72.79%)	37,835 (80.35%)	58,357 (77.52%)
Stimulants	106 (0.38%)	238 (0.51%)	344 (0.46%)
Sunew	19,829 (70.33%)	26,716 (56.74%)	46,545 (61.83%)
Suold	15 (0.05%)	0 (0%)	15 (0.02%)
Thiazidediuretics	7,547 (26.77%)	12,129 (25.76%)	19,676 (26.14%)
Tir	0 (0%)	0 (0%)	0 (0%)
Tzd	1,996 (7.08%)	3,018 (6.41%)	5,014 (6.66%)
Only Met Therapy	3,862 (13.7%)	7,211 (15.31%)	11,073 (14.71%)

© 2025 Neugebauer R et al. *JAMA Network Open*.

eFigure 23. MACE (Primary Definition), 2-Arm Drug Class Comparison, DPP4is vs SGLT2is, Cumulative Incidence Curves From PP and ITT Analyses With IPW, TMLE, and SL
Each plot emulates inferences from a 2-arm RCT comparing DPP4i and SGLT2i and represents unadjusted or adjusted estimates of cumulative incidence curves for MACE derived with inverse probability weighting (IPW) and Targeted Minimum Loss-based Estimation (TMLE) with Super Learning (SL) estimates of propensity scores with four weight truncation schemes: IPW and TMLE without weight truncation (untruncated), IPW with truncation of stabilized weights at value 20 (trunc20) or at the 99th percentile of weight values (trunc99), and TMLE with truncation of unstabilized weights at value 200 (trunc200). The red divider line separates results of Per-Protocol (PP) analyses (top half) from Intention-To-Treat (ITT) analyses (bottom half). Each plot displays a p value for the test that the average risk difference (ARD) through 2.5 years of follow-up (30 months) is 0.

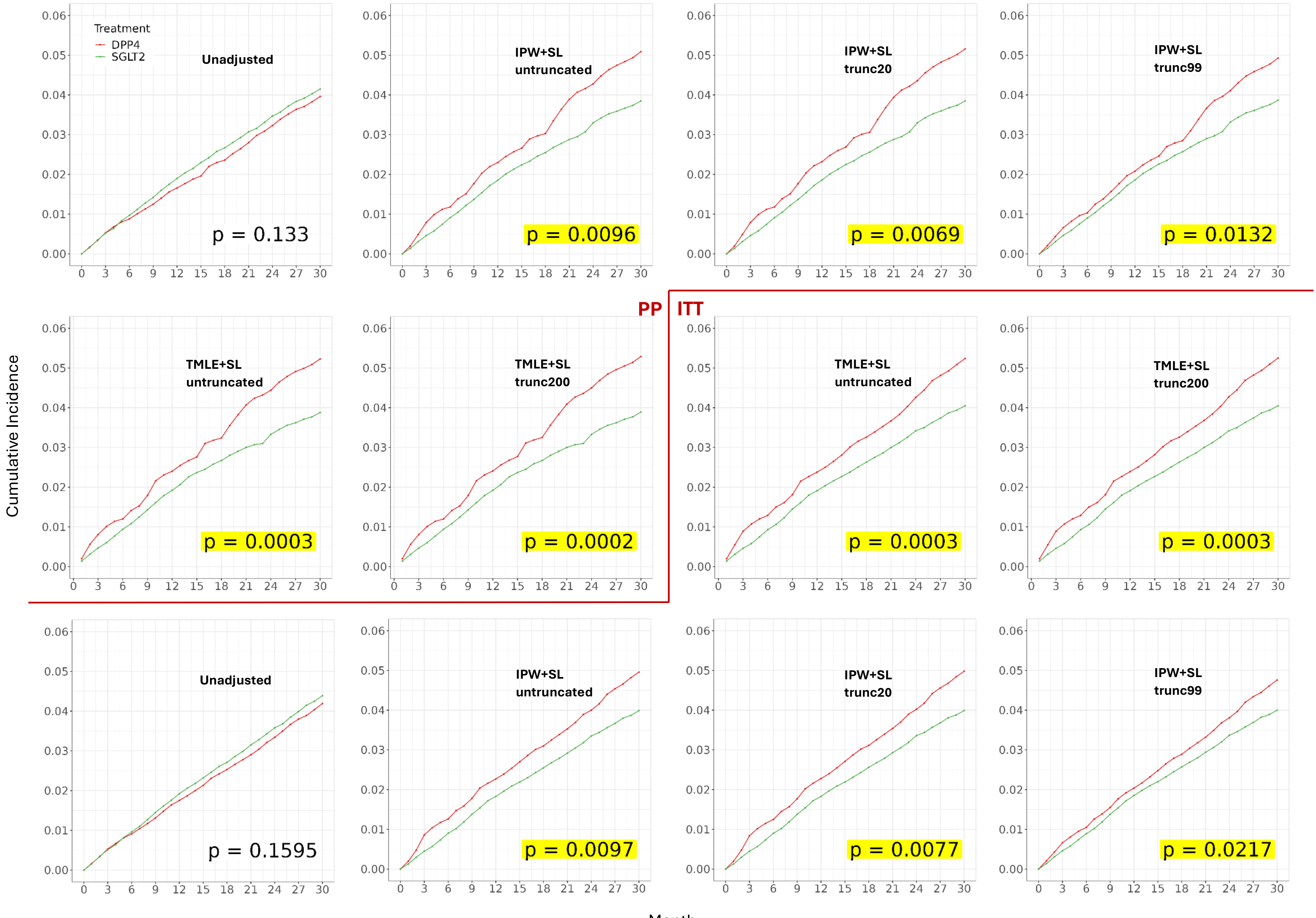

© 2025 Neugebauer R et al. *JAMA Network Open*.

eFigure 24. MACE (Primary Definition), 2-Arm Drug Class Comparison, DPP4is vs SGLT2is, Cumulative Incidence Curves From Sensitivity PP Analyses With IPW, TMLE, and SL
Each plot emulates inferences from a 2-arm RCT comparing DPP4i and SGLT2i and represents unadjusted or adjusted estimates of cumulative incidence curves for MACE from sensitivity PP analyses referred to as "NoMBS PP" and "No3 PP". NoMBS PP analyses are restricted to patients without a history of MBS at baseline and the protocols they evaluate preclude metabolic bariatric surgery (MBS) procedures. The protocols in the No3 PP analyses preclude exposure to three medication classes: the comparator medication from the other arm, SU and GLP-1RA.

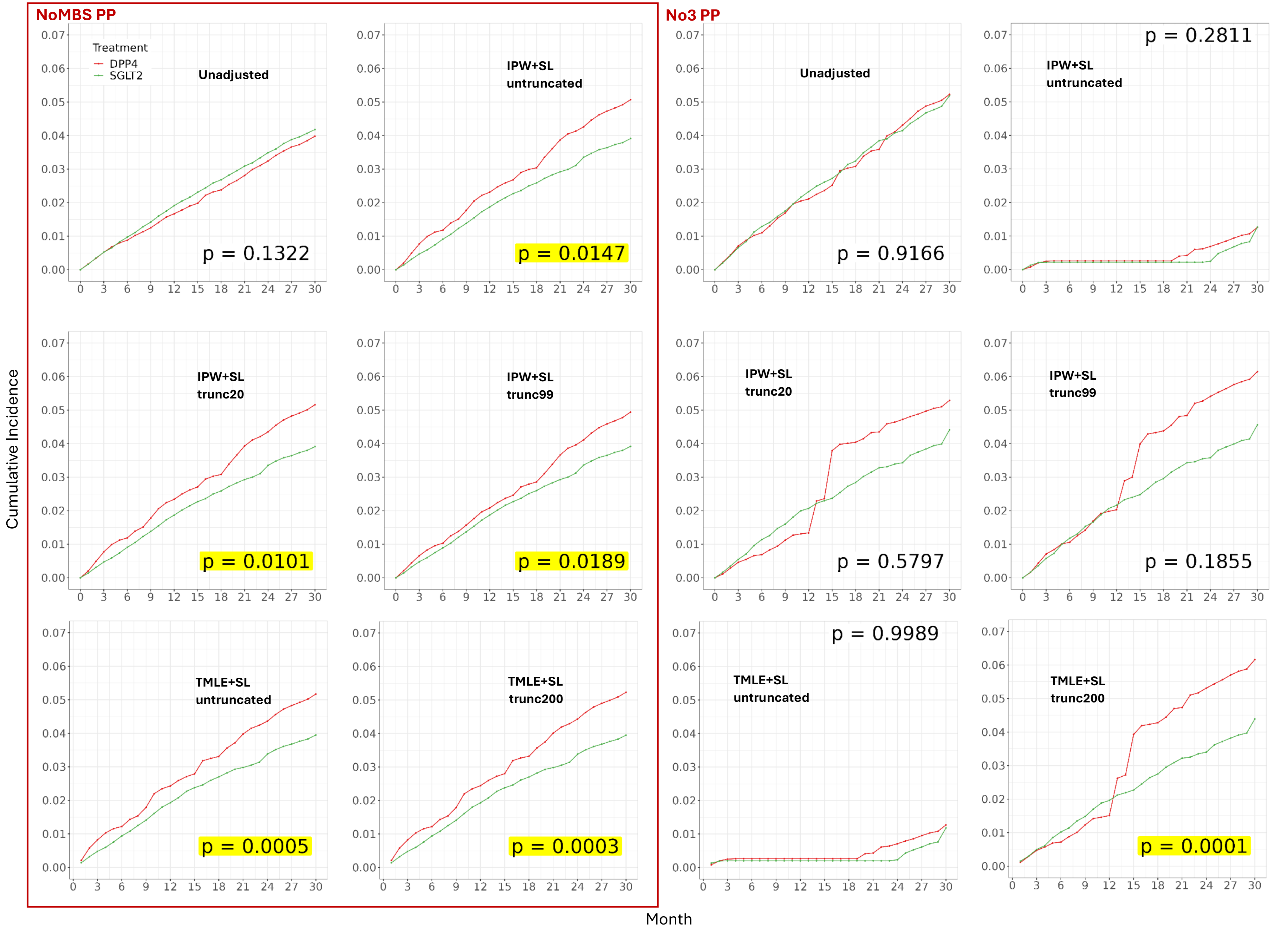

© 2025 Neugebauer R et al. *JAMA Network Open*.

eTable 25. MACE (Primary Definition), 2-Arm Drug Class Comparison, DPP4is vs SGLT2is, RD and HR Effect Measures at 2.5 Years

Estimation results from ITT, PP, NoMBS PP, and No3 PP analyses of emulated 2-arm RCTs comparing MACE risks over 2.5 years between DPP4i and SGLT2i initiators. For PP analyses, rates of protocol deviations are described by medication class initiated at baseline. Unadjusted point and interval estimates and adjusted point and interval IPW and TMLE estimates of risks, risk differences (RD), and hazard ratios (HR) based on propensity scores (PS) estimated with either logistic models or super learning (SL) are presented for four weight truncation schemes along with the corresponding 99th percentile and maximum value of the stabilized and unstabilized inverse probability weights used for implementing IPW and TMLE, respectively. RD is the risk in treatment arm minus the risk in control arm and NNT is the number needed to treat.

Analysis type	Protocol Deviations* by exposure group (%)	PS estimation	99th IP weights	Max IP weight	Estimator	Treatment (DPP4i) risk in %	Control (SGLT2i) risk in %	RD [95% CI] in %	NNT	HR [95% CI]
PP	Discontinuation<br>DPP4i: 60.78<br>SGLT2i: 26.18<br><br>Crossover<br>DPP4i: 10.83<br>SGLT2i: 1.93	SL			Unadjusted	3.96	4.15	-0.19 [-0.73, 0.35]		0.89 [0.77, 1.00]
			18.25	2,275.40	TMLE untruncated	5.23	3.88	1.34 [0.76, 1.93]	74	
					TMLE truncated at 200	5.29	3.89	1.40 [0.82, 1.98]	71	
			4.49	257.83	IPW untruncated	5.09	3.85	1.24 [0.32, 2.15]	81	1.28 [0.98, 1.59]
					IPW truncated at 20	5.16	3.85	1.31 [0.39, 2.22]	77	1.29 [0.99, 1.60]
					IPW truncated at 99th	4.93	3.87	1.06 [0.27, 1.85]	94	1.15 [0.94, 1.35]
		Logistic model	6.05	2,999.47	IPW untruncated	5.47	4.17	1.30 [-0.35, 2.95]		1.30 [0.73, 1.86]
					IPW truncated at 20	5.44	4.13	1.31 [-0.02, 2.65]	76	1.15 [0.86, 1.43]
					IPW truncated at 99th	4.95	4.15	0.81 [-0.16, 1.78]		1.10 [0.89, 1.32]
ITT		SL			Unadjusted	4.19	4.39	-0.20 [-0.61, 0.21]		0.92 [0.81, 1.02]
			12.00	739.01	TMLE untruncated	5.24	4.05	1.19 [0.73, 1.64]	84	
					TMLE truncated at 200	5.25	4.05	1.20 [0.74, 1.65]	84	
			4.14	244.08	IPW untruncated	4.96	3.99	0.98 [0.32, 1.63]	102	1.27 [1.00, 1.55]
					IPW truncated at 20	4.98	3.99	0.99 [0.34, 1.64]	101	1.28 [1.00, 1.55]
					IPW truncated at 99th	4.76	4.00	0.75 [0.21, 1.30]	133	1.13 [0.95, 1.30]
		Logistic model	5.54	1,341.42	IPW untruncated	5.24	4.16	1.08 [-0.06, 2.22]		1.26 [0.78, 1.73]
					IPW truncated at 20	4.95	4.15	0.80 [-0.02, 1.62]	124	1.11 [0.85, 1.37]
					IPW truncated at 99th	4.77	4.12	0.64 [-0.00, 1.29]	156	1.07 [0.88, 1.26]
NoMBS PP	Discontinuation<br>DPP4i: 60.52<br>SGLT2i: 25.89<br><br>Crossover<br>DPP4i: 11.15<br>SGLT2i: 2.25<br><br>MBS occurrence<br>DPP4i: 0.33<br>SGLT2i: 0.33	SL			Unadjusted	3.98	4.18	-0.20 [-0.75, 0.34]		0.89 [0.77, 1.01]
			18.08	3,071.47	TMLE untruncated	5.17	3.95	1.22 [0.64, 1.81]	82	
					TMLE truncated at 200	5.23	3.95	1.29 [0.71, 1.87]	78	
			4.49	351.17	IPW untruncated	5.07	3.91	1.16 [0.24, 2.08]	86	1.28 [0.97, 1.58]
					IPW truncated at 20	5.16	3.91	1.24 [0.32, 2.17]	80	1.29 [0.98, 1.60]
					IPW truncated at 99th	4.94	3.92	1.01 [0.21, 1.82]	99	1.15 [0.94, 1.35]
		Logistic model	6.03	3,113.98	IPW untruncated	5.53	4.25	1.28 [-0.39, 2.95]		1.30 [0.73, 1.87]
					IPW truncated at 20	5.53	4.20	1.33 [-0.03, 2.69]		1.16 [0.87, 1.45]
					IPW truncated at 99th	5.03	4.21	0.81 [-0.18, 1.80]		1.11 [0.89, 1.33]
No3 PP	Discontinuation<br>DPP4i: 12.31<br>SGLT2i: 8.81<br><br>Crossover to comparator drug<br>DPP4i: 2.06<br>SGLT2i: 0.56<br><br>Initiation of one of the two non-comparator drugs<br>DPP4i: 78.55<br>SGLT2i: 65.77	SL			Unadjusted	5.23	5.19	0.04 [-1.20, 1.28]		1.00 [0.58, 1.41]
			23.69	3.75e+19	TMLE untruncated	1.27	1.18	0.09 [-3.38, 3.56]		
					TMLE truncated at 200	6.16	4.39	1.76 [0.99, 2.53]	57	
			3.80	9.97e+17	IPW untruncated	1.26	1.27	-0.01 [-0.94, 0.92]		1.14 [0.30, 1.98]
					IPW truncated at 20	5.29	4.41	0.87 [-2.41, 4.15]		0.85 [0.31, 1.40]
					IPW truncated at 99th	6.15	4.56	1.59 [-0.94, 4.12]		1.18 [0.42, 1.94]
		Logistic model	4.63	1.91e+17	IPW untruncated	0.86	1.22	-0.36 [-1.24, 0.51]		0.61 [-0.07, 1.28]
					IPW truncated at 20	5.98	4.50	1.48 [-3.22, 6.18]		0.87 [0.29, 1.46]
					IPW truncated at 99th	5.89	4.64	1.25 [-1.50, 4.00]		1.13 [0.37, 1.90]

* Discontinuation refers to the interruption of the comparator medication initiated on index date; Crossover refers to the initiation of the comparator medication initiated by patient at baseline in the other arm; MBS occurrence refers to patient's undergoing metabolic bariatric surgery (MBS).

© 2025 Neugebauer R et al. *JAMA Network Open*.

eFigure 25. MACE (Primary Definition), 2-Arm Drug Class Comparison, DPP4is vs SGLT2is, ASCVD Subgroup, Cumulative Incidence Curves From PP and ITT Analyses With IPW, TMLE, and SL
Each plot emulates inferences among patients with ASCVD from a 2-arm RCT comparing DPP4i and SGLT2i and represents unadjusted or adjusted estimates of cumulative incidence curves for MACE derived with IPW and TMLE with SL estimates of propensity scores with four weight truncation schemes: IPW and TMLE without weight truncation (untruncated), IPW with truncation of stabilized weights at value 20 (trunc20) or at the 99th percentile of weight values (trunc99), and TMLE with truncation of unstabilized weights at value 200 (trunc200). The red divi der line separates results of Per-Protocol (PP) analyses (top half) from Intention-To-Treat (ITT) analyses (bottom half). Each plot displays a p value for the test that the average risk difference (ARD) through 2.5 years of follow-up (30 months) is 0.

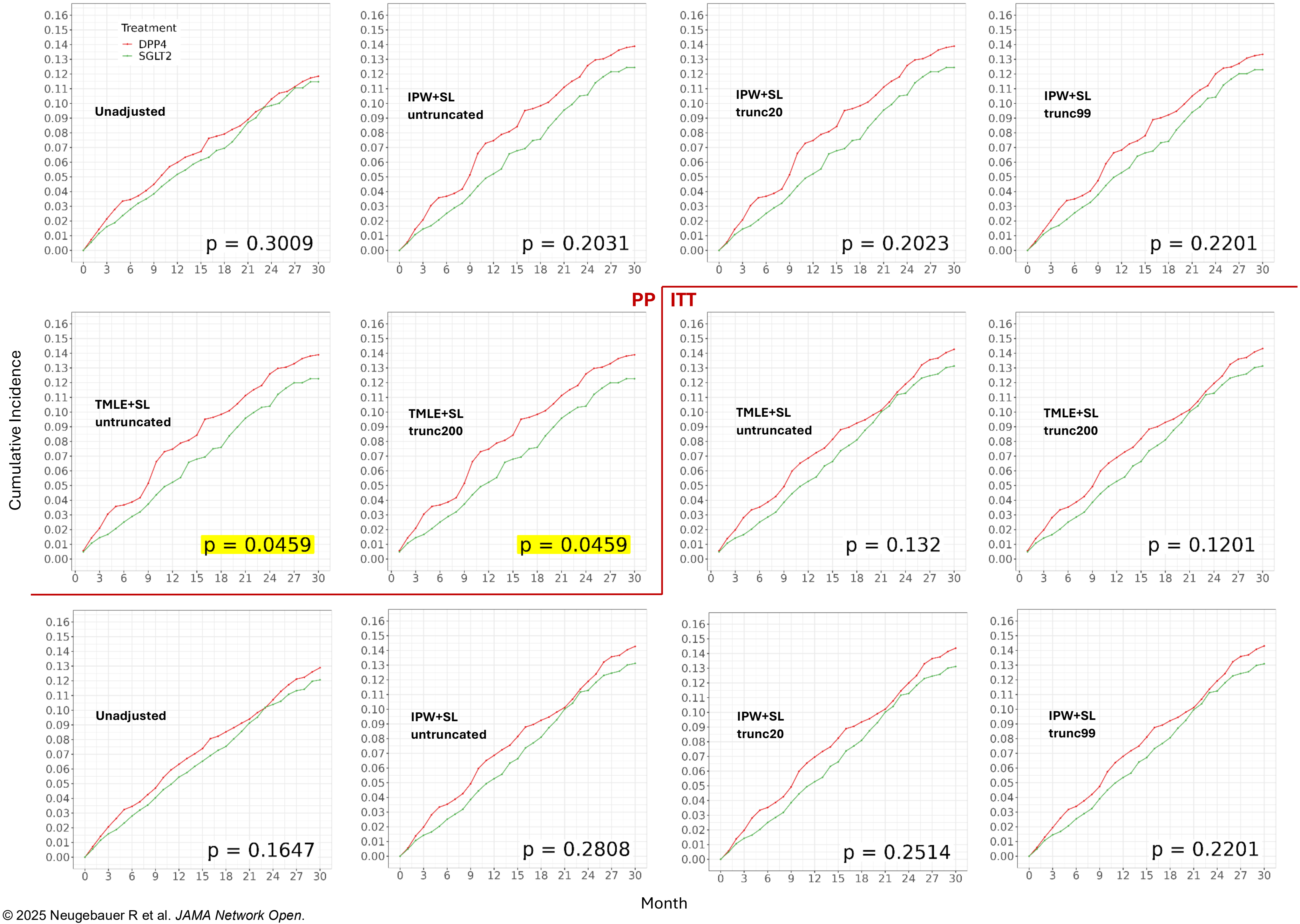

© 2025 Neugebauer R et al. *JAMA Network Open*.

eTable 26. MACE (Primary Definition), 2-Arm Drug Class Comparison, DPP4is vs SGLT2is, ASCVD Subgroup, RD and HR Effect Measures at 2.5 Years

Estimation results among patients with ASCVD from ITT and PP analyses of emulated 2-arm RCTs comparing MACE risks over 2.5 years between DPP4i and SGLT2i initiators. For PP analyses, rates of protocol deviations are described by medication class initiated at baseline. Unadjusted point and interval estimates and adjusted point and interval IPW and TMLE estimates of risks, risk differences (RD), and hazard ratios (HR) based on propensity scores (PS) estimated with either logistic models or super learning (SL) are presented for four weight truncation schemes along with the corresponding 99th percentile and maximum value of the stabilized and unstabilized inverse probability weights used for implementing IPW and TMLE, respectively. RD is the risk in treatment arm minus the risk in control arm and NNT is the number needed to treat.

Analysis type	Protocol Deviations* by exposure group (%)	PS estimation	99th IP weights	Max IP weight	Estimator	Treatment (DPP4i) risk in %	Control (SGLT2i) risk in %	RD [95% CI] in %	NNT	HR [95% CI]
PP	Discontinuation<br>DPP4i: 59.26<br>SGLT2i: 24.33<br><br>Crossover<br>DPP4i: 10.99<br>SGLT2i: 1.49	SL			Unadjusted	11.85	11.47	0.38 [-1.97, 2.73]		1.19 [0.96, 1.43]
			16.77	179.13	TMLE untruncated	13.90	12.27	1.63 [-0.54, 3.80]		
					TMLE truncated at 200	13.90	12.27	1.63 [-0.54, 3.80]		
			4.19	26.09	IPW untruncated	13.89	12.44	1.46 [-2.61, 5.52]		1.46 [0.95, 1.96]
					IPW truncated at 20	13.90	12.44	1.46 [-2.60, 5.53]		1.46 [0.95, 1.96]
					IPW truncated at 99th	13.34	12.29	1.05 [-2.49, 4.58]		1.32 [0.97, 1.68]
		Logistic model	5.95	326.22	IPW untruncated	14.30	12.28	2.03 [-3.76, 7.81]		1.73 [0.51, 2.95]
					IPW truncated at 20	13.38	12.35	1.03 [-3.38, 5.43]		1.41 [0.82, 1.99]
					IPW truncated at 99th	13.30	12.28	1.02 [-2.93, 4.97]		1.27 [0.88, 1.67]
ITT		SL			Unadjusted	12.89	12.06	0.83 [-0.94, 2.60]		1.18 [0.98, 1.39]
			12.61	988.37	TMLE untruncated	14.27	13.13	1.14 [-0.61, 2.90]		
					TMLE truncated at 200	14.32	13.13	1.19 [-0.56, 2.94]		
			3.99	262.27	IPW untruncated	14.28	13.12	1.16 [-1.86, 4.18]		1.33 [0.94, 1.73]
					IPW truncated at 20	14.37	13.12	1.25 [-1.78, 4.28]		1.35 [0.95, 1.75]
					IPW truncated at 99th	14.31	13.09	1.22 [-1.56, 4.00]		1.29 [0.98, 1.59]
		Logistic model	5.55	369.73	IPW untruncated	16.63	13.55	3.07 [-3.07, 9.21]		1.50 [0.51, 2.49]
					IPW truncated at 20	15.83	13.60	2.23 [-2.76, 7.22]		1.27 [0.81, 1.73]
					IPW truncated at 99th	14.75	13.77	0.98 [-2.52, 4.47]		1.21 [0.88, 1.54]

* Discontinuation refers to the interruption of the comparator medication initiated on index date; Crossover refers to the initiation of the comparator medication initiated by patient at baseline in the other arm.

© 2025 Neugebauer R et al. *JAMA Network Open*.

eFigure 26. MACE (Primary Definition), 2-Arm Drug Class Comparison, DPP4is vs SGLT2is, No ASCVD Subgroup, Cumulative Incidence Curves From PP and ITT Analyses With IPW, TMLE, and SL
Each plot emulates inferences among patients with No ASCVD from a 2-arm RCT comparing DPP4i and SGLT2i and represents unadjusted or adjusted estimates of cumulative incidence curves for MACE derived with IPW and TMLE with SL estimates of propensity scores with four weight truncation schemes: IPW and TMLE without weight truncation (untruncated), IPW with truncation of stabilized weights at value 20 (trunc20) or at the 99th percentile of weight values (trunc99), and TMLE with truncation of unstabilized weights at value 200 (trunc200). The red divider line separates results of Per-Protocol (PP) analyses (top half) from Intention-To-Treat (ITT) analyses (bottom half). Each plot displays a p value for the test that the average risk difference (ARD) through 2.5 years of follow-up (30 months) is 0.

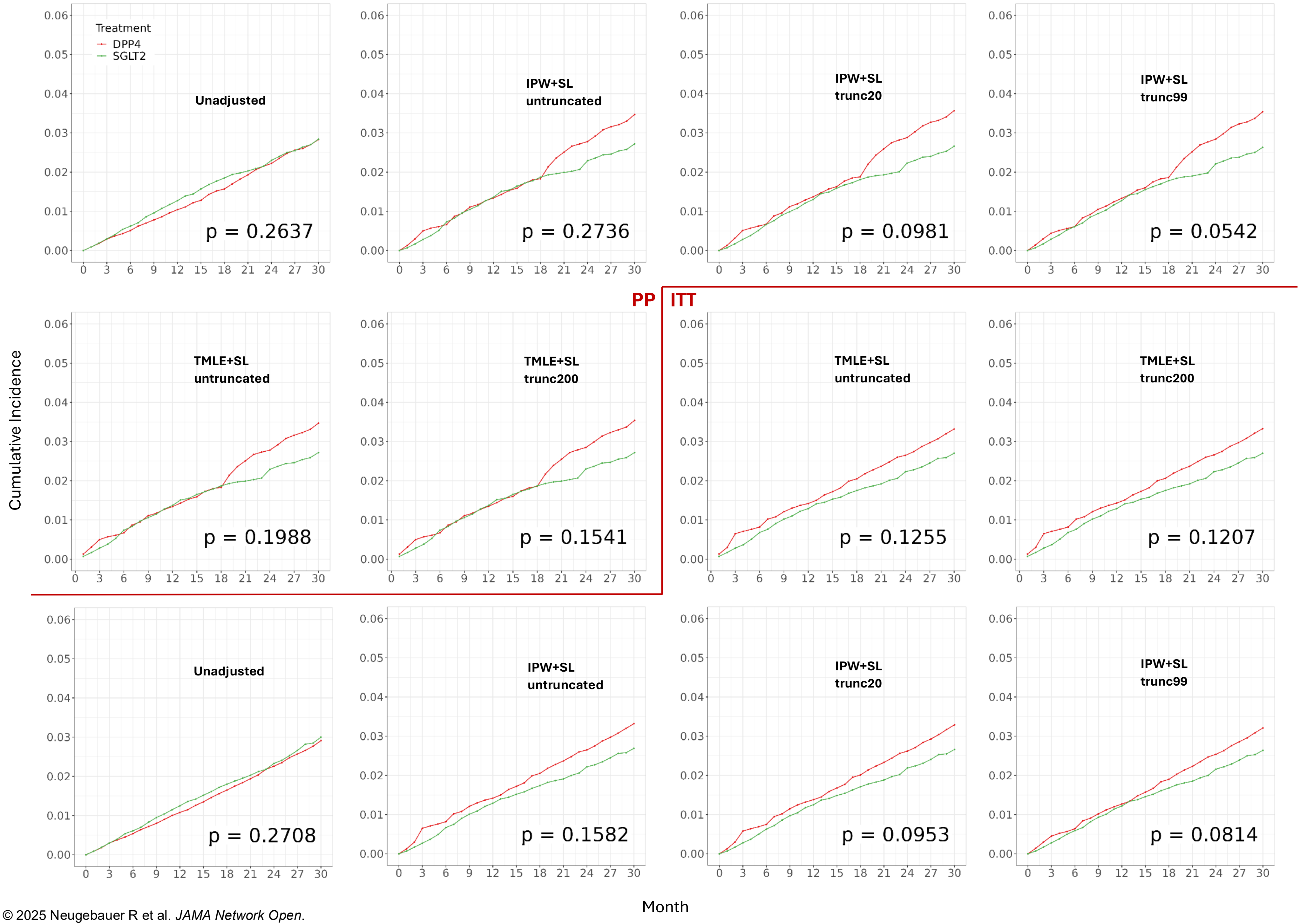

© 2025 Neugebauer R et al. *JAMA Network Open*.

eTable 27. MACE (Primary Definition), 2-Arm Drug Class Comparison, DPP4is vs SGLT2is, No ASCVD Subgroup, RD and HR Effect Measures at 2.5 Years

Estimation results among patients with No ASCVD from ITT and PP analyses of emulated 2-arm RCTs comparing MACE risks over 2.5 years between DPP4i and SGLT2i initiators. For PP analyses, rates of protocol deviations are described by medication class initiated at baseline. Unadjusted point and interval estimates and adjusted point and interval IPW and TMLE estimates of risks, risk differences (RD), and hazard ratios (HR) based on propensity scores (PS) estimated with either logistic models or super learning (SL) are presented for four weight truncation schemes along with the corresponding 99th percentile and maximum value of the stabilized and unstabilized inverse probability weights used for implementing IPW and TMLE, respectively. RD is the risk in treatment arm minus the risk in control arm and NNT is the number needed to treat.

Analysis type	Protocol Deviations* by exposure group (%)	PS estimation	99th IP weights	Max IP weight	Estimator	Treatment (DPP4i) risk in %	Control (SGLT2i) risk in %	RD [95% CI] in %	NNT	HR [95% CI]
PP	Discontinuation<br>DPP4i: 61.00<br>SGLT2i: 26.54<br><br>Crossover<br>DPP4i: 10.81<br>SGLT2i: 2.01	SL			Unadjusted	2.83	2.84	-0.01 [-0.51, 0.48]		0.84 [0.69, 0.98]
			20.13	4,353.30	TMLE untruncated	3.47	2.72	0.75 [0.17, 1.33]	133	
					TMLE truncated at 200	3.54	2.72	0.82 [0.24, 1.39]	122	
			5.07	515.57	IPW untruncated	3.47	2.72	0.76 [-0.09, 1.60]		1.04 [0.66, 1.41]
					IPW truncated at 20	3.57	2.66	0.91 [0.09, 1.74]	109	1.11 [0.76, 1.46]
					IPW truncated at 99th	3.54	2.63	0.91 [0.16, 1.65]	110	1.09 [0.82, 1.37]
		Logistic model	5.96	1,897.58	IPW untruncated	3.47	2.98	0.49 [-0.84, 1.82]		0.89 [0.52, 1.27]
					IPW truncated at 20	3.78	2.87	0.91 [-0.33, 2.15]		1.05 [0.71, 1.38]
					IPW truncated at 99th	3.46	2.86	0.60 [-0.26, 1.46]		1.07 [0.79, 1.36]
ITT		SL			Unadjusted	2.91	3.00	-0.10 [-0.47, 0.27]		0.88 [0.74, 1.02]
			13.51	1,349.80	TMLE untruncated	3.32	2.70	0.62 [0.11, 1.13]	161	
					TMLE truncated at 200	3.33	2.70	0.63 [0.12, 1.14]	159	
			4.71	459.54	IPW untruncated	3.32	2.69	0.63 [-0.01, 1.27]	159	1.18 [0.74, 1.62]
					IPW truncated at 20	3.29	2.66	0.63 [0.07, 1.19]	159	1.17 [0.81, 1.52]
					IPW truncated at 99th	3.21	2.64	0.57 [0.09, 1.06]	174	1.09 [0.84, 1.34]
		Logistic model	5.45	1,845.65	IPW untruncated	3.17	2.75	0.42 [-0.26, 1.11]		1.07 [0.64, 1.51]
					IPW truncated at 20	3.21	2.70	0.51 [-0.08, 1.10]		1.13 [0.78, 1.47]
					IPW truncated at 99th	3.17	2.70	0.47 [-0.05, 1.00]		1.07 [0.81, 1.32]

* Discontinuation refers to the interruption of the comparator medication initiated on index date; Crossover refers to the initiation of the comparator medication initiated by patient at baseline in the other arm.

© 2025 Neugebauer R et al. *JAMA Network Open*.

eFigure 27. MACE (Primary Definition), 2-Arm Drug Class Comparison, DPP4is vs SGLT2is, No ASCVD and MET Subgroup, Cumulative Incidence Curves From PP and ITT Analyses With IPW, TMLE, and SL
Each plot emulates inferences among patients with No ASCVD and MET from a 2-arm RCT comparing DPP4i and SGLT2i and represents unadjusted or adjusted estimates of cumulative incidence curves for MACE derived with IPW and TMLE with SL estimates of propensity scores with four weight truncation schemes: IPW and TMLE without weight truncation (untruncated), IPW with truncation of stabilized weights at value 20 (trunc20) or at the 99th percentile of weight values (trunc99), and TMLE with truncation of unstabilized weights at value 200 (trunc200). The red divi der line separates results of Per-Protocol (PP) analyses (top half) from Intention-To-Treat (ITT) analyses (bottom half). Each plot displays a p value for the test that the average risk difference (ARD) through 2.5 years of follow-up (30 months) is 0.

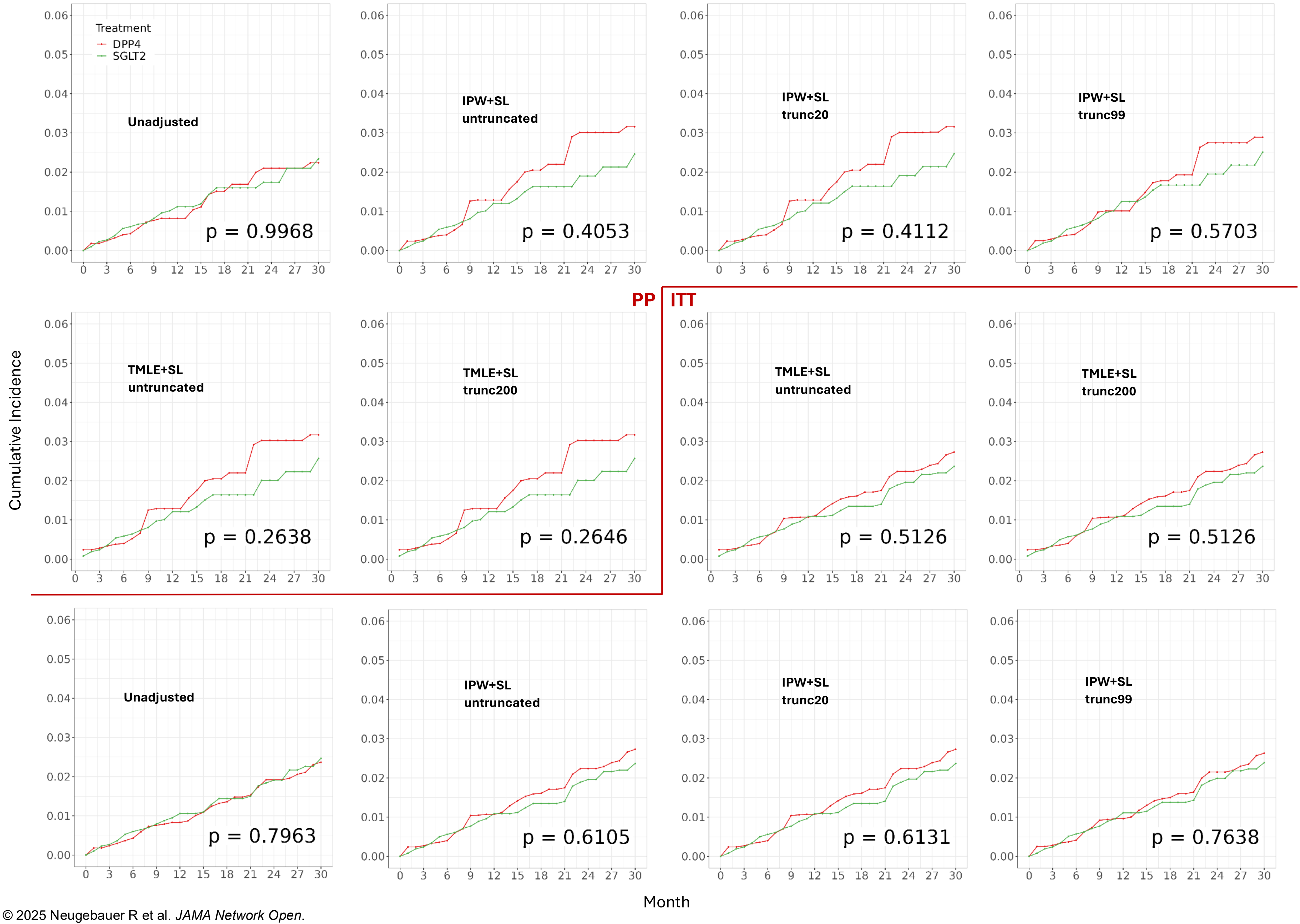

© 2025 Neugebauer R et al. *JAMA Network Open*.

eTable 28. MACE (Primary Definition), 2-Arm Drug Class Comparison, DPP4is vs SGLT2is, No ASCVD and MET Subgroup, RD and HR Effect Measures at 2.5 Years

Estimation results among patients with No ASCVD and MET from ITT and PP analyses of emulated 2-arm RCTs comparing MACE risks over 2.5 years between DPP4i and SGLT2i initiators. For PP analyses, rates of protocol deviations are described by medication class initiated at baseline. Unadjusted point and interval estimates and adjusted point and interval IPW and TMLE estimates of risks, risk differences (RD), and hazard ratios (HR) based on propensity scores (PS) estimated with either logistic models or super learning (SL) are presented for four weight truncation schemes along with the corresponding 99th percentile and maximum value of the stabilized and unstabilized inverse probability weights used for implementing IPW and TMLE, respectively. RD is the risk in treatment arm minus the risk in control arm and NNT is the number needed to treat.

Analysis type	Protocol Deviations* by exposure group (%)	PS estimation	99th IP weights	Max IP weight	Estimator	Treatment (DPP4i) risk in %	Control (SGLT2i) risk in %	RD [95% CI] in %	NNT	HR [95% CI]
PP	Discontinuation<br>DPP4i: 57.07<br>SGLT2i: 27.18<br><br>Crossover<br>DPP4i: 9.61<br>SGLT2i: 2.45	SL			Unadjusted	2.24	2.34	-0.10 [-1.30, 1.10]		0.77 [0.36, 1.19]
			14.04	481.00	TMLE untruncated	3.17	2.57	0.60 [-0.54, 1.74]		
					TMLE truncated at 200	3.17	2.57	0.60 [-0.54, 1.74]		
			3.37	81.64	IPW untruncated	3.16	2.46	0.69 [-1.27, 2.66]		1.06 [0.17, 1.94]
					IPW truncated at 20	3.16	2.47	0.68 [-1.28, 2.65]		1.06 [0.18, 1.95]
					IPW truncated at 99th	2.89	2.51	0.38 [-1.33, 2.09]		0.89 [0.29, 1.50]
		Logistic model	5.55	103.12	IPW untruncated	3.34	2.74	0.60 [-1.71, 2.91]		0.93 [-0.08, 1.95]
					IPW truncated at 20	3.42	2.75	0.67 [-1.70, 3.03]		0.97 [-0.08, 2.02]
					IPW truncated at 99th	3.21	2.64	0.57 [-1.56, 2.69]		0.86 [0.08, 1.65]
ITT		SL			Unadjusted	2.37	2.47	-0.10 [-1.01, 0.81]		0.83 [0.43, 1.23]
			9.89	350.91	TMLE untruncated	2.73	2.37	0.36 [-0.44, 1.16]		
					TMLE truncated at 200	2.73	2.37	0.36 [-0.44, 1.16]		
			3.28	104.11	IPW untruncated	2.73	2.37	0.36 [-0.86, 1.59]		1.03 [0.29, 1.76]
					IPW truncated at 20	2.73	2.37	0.36 [-0.86, 1.59]		1.04 [0.29, 1.78]
					IPW truncated at 99th	2.63	2.39	0.24 [-0.87, 1.35]		0.94 [0.36, 1.52]
		Logistic model	5.36	402.37	IPW untruncated	2.83	2.67	0.15 [-1.36, 1.66]		0.91 [0.02, 1.79]
					IPW truncated at 20	2.92	2.69	0.24 [-1.31, 1.78]		0.97 [0.04, 1.90]
					IPW truncated at 99th	2.87	2.60	0.28 [-1.12, 1.68]		0.97 [0.18, 1.76]

* Discontinuation refers to the interruption of the comparator medication initiated on index date; Crossover refers to the initiation of the comparator medication initiated by patient at baseline in the other arm.

© 2025 Neugebauer R et al. *JAMA Network Open*.

eFigure 28. MACE (Primary Definition), 2-Arm Drug Class Comparison, DPP4is vs GLP-1RAs, CONSORT Diagram
Flow diagram describing the inclusion and exclusion steps and counts leading to the creation of the cohort for emulating the 2-arm RCT to compare the risk of MACE in new users of DPP4i and GLP-1RA along with sample sizes and counts for each observed end of follow-up type by treatment initiated at cohort entry.

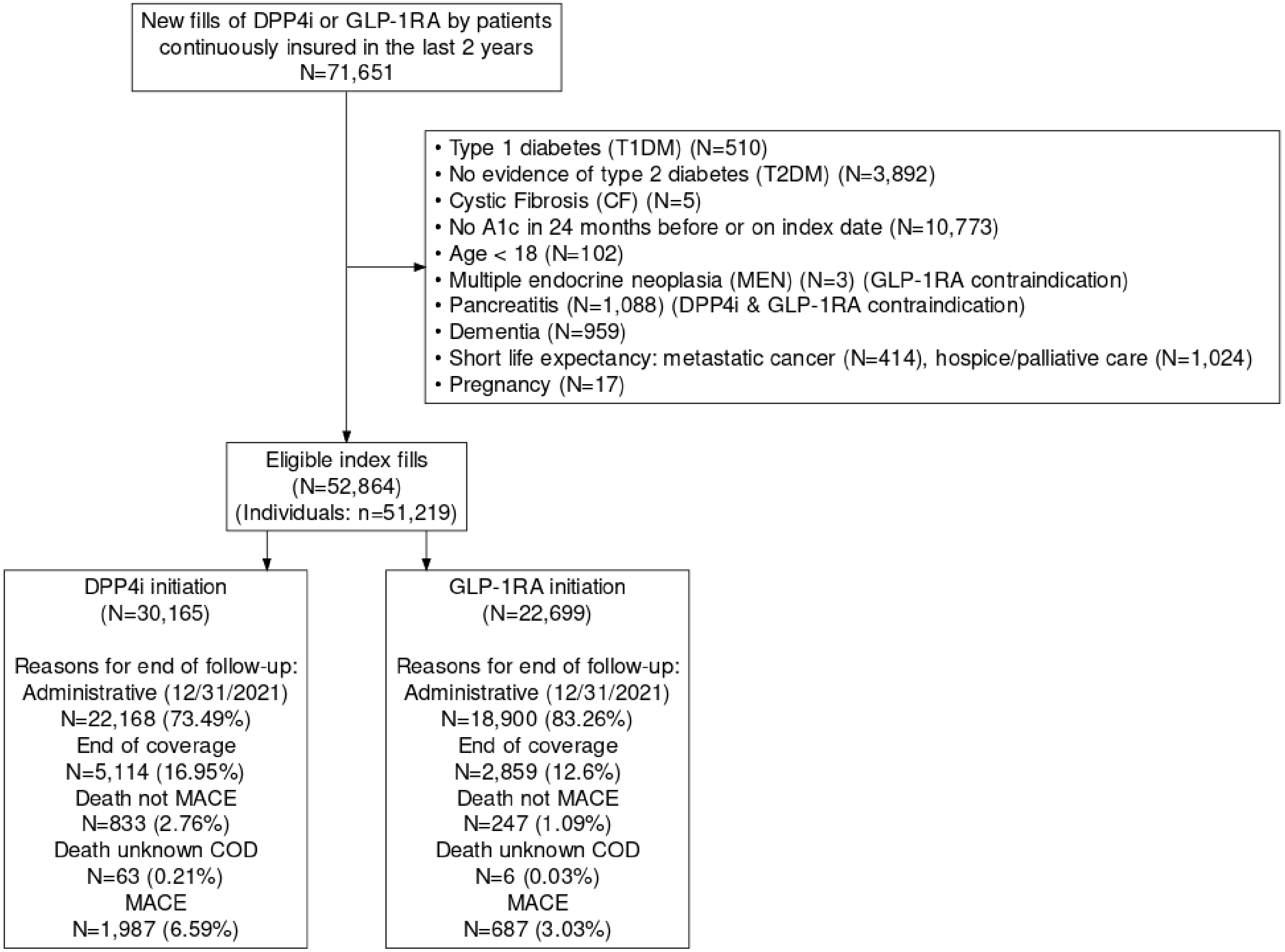

© 2025 Neugebauer R et al. *JAMA Network Open*.

eTable 29. MACE (Primary Definition), 2-Arm Drug Class Comparison, DPP4is vs GLP-1RAs, Patient Characteristics at Baseline (Overall and by Medication Initiated)
Summary statistics of the baseline values for selected covariates in the cohort of patients used to emulate a 2-arm RCT for comparing DPP4i and GLP-1RA. For each continuous variable, the mean and standard deviation are displayed for all patients in the cohort (last column) and by drug class initiated at cohort entry. For each categorical variable and for each possible level of that variable, the count and proportion are displayed instead.

	DPP4i n = 30,165	GLP-1RA n = 22,699	Total n = 52,864
**Demographics**			
Age	61.39 (12.01)	56.41 (11.85)	59.25 (12.19)
Agegrp			
<45	2,466 (8.18%)	3,630 (15.99%)	6,096 (11.53%)
[45-65)	15,625 (51.8%)	13,249 (58.37%)	28,874 (54.62%)
[65-75)	7,986 (26.47%)	4,715 (20.77%)	12,701 (24.03%)
>=75	4,088 (13.55%)	1,105 (4.87%)	5,193 (9.82%)
Ethnicity			
Hispanic	8,911 (29.54%)	5,452 (24.02%)	14,363 (27.17%)
Nonhispanic	21,254 (70.46%)	17,247 (75.98%)	38,501 (72.83%)
Female Head Of Hh	0.146 (0.073)	0.140 (0.076)	0.144 (0.074)
Missing	971 (3.219%)	937 (4.128%)	1,908 (3.609%)
Hh Public Assistance	0.037 (0.034)	0.036 (0.033)	0.037 (0.033)
Missing	971 (3.219%)	937 (4.128%)	1,908 (3.609%)
Household Income Less 30k	0.217 (0.125)	0.202 (0.122)	0.211 (0.124)
Missing	971 (3.219%)	937 (4.128%)	1,908 (3.609%)
Houspoverty	0.106 (0.092)	0.093 (0.085)	0.100 (0.089)
Missing	972 (3.222%)	942 (4.15%)	1,914 (3.621%)
Index Yr			
2014	1,670 (5.54%)	496 (2.19%)	2,166 (4.1%)
2015	3,401 (11.27%)	736 (3.24%)	4,137 (7.83%)
2016	5,527 (18.32%)	1,048 (4.62%)	6,575 (12.44%)
2017	5,384 (17.85%)	1,668 (7.35%)	7,052 (13.34%)
2018	4,259 (14.12%)	3,015 (13.28%)	7,274 (13.76%)
2019	4,171 (13.83%)	3,383 (14.9%)	7,554 (14.29%)
2020	2,966 (9.83%)	4,637 (20.43%)	7,603 (14.38%)
2021	2,787 (9.24%)	7,716 (33.99%)	10,503 (19.87%)
Bmi	32.26 (7.09)	37.62 (8.12)	34.55 (8.00)
Missing	615 (2.04%)	620 (2.73%)	1,235 (2.34%)
Smoking Status			
Formersmoker	9,008 (29.86%)	7,197 (31.71%)	16,205 (30.65%)
Currentsmoker	2,433 (8.07%)	2,128 (9.37%)	4,561 (8.63%)
Passivesmoker	133 (0.44%)	101 (0.44%)	234 (0.44%)
Neversmoker	18,303 (60.68%)	12,926 (56.95%)	31,229 (59.07%)
Unknown	288 (0.95%)	347 (1.53%)	635 (1.2%)
Low Educ	0.170 (0.131)	0.142 (0.115)	0.158 (0.125)
Missing	971 (3.219%)	935 (4.119%)	1,906 (3.605%)
Mgr Male	0.043 (0.041)	0.044 (0.043)	0.044 (0.042)
Missing	971 (3.219%)	936 (4.124%)	1,907 (3.607%)
Ndi	0.28 (0.17)	0.25 (0.15)	0.27 (0.16)
Missing	972 (3.22%)	942 (4.15%)	1,914 (3.62%)
Pct Crowding	0.082 (0.086)	0.071 (0.079)	0.077 (0.083)
Missing	971 (3.219%)	937 (4.128%)	1,908 (3.609%)
Racegrp			
White	16,106 (53.39%)	13,559 (59.73%)	29,665 (56.12%)
Asian	5,066 (16.79%)	2,010 (8.86%)	7,076 (13.39%)
Blackorafricanamerican	3,064 (10.16%)	2,874 (12.66%)	5,938 (11.23%)
Hawaiianorpacificislander	423 (1.4%)	412 (1.82%)	835 (1.58%)
Americanindianoralaskanative	191 (0.63%)	127 (0.56%)	318 (0.6%)
Multirace	1,078 (3.57%)	1,192 (5.25%)	2,270 (4.29%)
Other	79 (0.26%)	98 (0.43%)	177 (0.33%)
Unknown	4,158 (13.78%)	2,427 (10.69%)	6,585 (12.46%)
Sex			

© 2025 Neugebauer R et al. *JAMA Network Open*.

	DPP4i n = 30,165	GLP-1RA n = 22,699	Total n = 52,864
Female	14,647 (48.56%)	12,416 (54.7%)	27,063 (51.19%)
Male	15,517 (51.44%)	10,283 (45.3%)	25,800 (48.8%)
Other	1 (0%)	0 (0%)	1 (0%)
Site			
Kpnc	5,174 (17.15%)	4,574 (20.15%)	9,748 (18.44%)
Kpsc	17,637 (58.47%)	8,642 (38.07%)	26,279 (49.71%)
Kphi	932 (3.09%)	1,735 (7.64%)	2,667 (5.05%)
Hpi	1,440 (4.77%)	2,600 (11.45%)	4,040 (7.64%)
Hfhs	2,063 (6.84%)	1,779 (7.84%)	3,842 (7.27%)
Ghs	2,919 (9.68%)	3,369 (14.84%)	6,288 (11.89%)
Unemployment	0.048 (0.027)	0.041 (0.026)	0.045 (0.027)
Missing	971 (3.219%)	935 (4.119%)	1,906 (3.605%)
**Insurance**			
Ins Commercial	18,651 (61.83%)	14,536 (64.04%)	33,187 (62.78%)
Ins Highdeductible	1,875 (6.22%)	1,573 (6.93%)	3,448 (6.52%)
Ins Medicaid	2,845 (9.43%)	2,994 (13.19%)	5,839 (11.05%)
Ins Medicare	11,504 (38.14%)	6,255 (27.56%)	17,759 (33.59%)
Ins Medicare A	6,058 (20.08%)	4,425 (19.49%)	10,483 (19.83%)
Ins Medicare B	5,732 (19%)	4,168 (18.36%)	9,900 (18.73%)
Ins Medicare C	4,896 (16.23%)	3,696 (16.28%)	8,592 (16.25%)
Ins Medicare D	5,200 (17.24%)	3,958 (17.44%)	9,158 (17.32%)
Ins Other Coverage	8,254 (27.36%)	5,049 (22.24%)	13,303 (25.16%)
Ins Privatepay	5,627 (18.65%)	2,605 (11.48%)	8,232 (15.57%)
Ins Selffunded	765 (2.54%)	1,420 (6.26%)	2,185 (4.13%)
Ins Statesubsidized	642 (2.13%)	1,146 (5.05%)	1,788 (3.38%)
**Clinical data**			
A1c Age	42.99 (76.52)	54.65 (85.37)	47.99 (80.65)
Missing	22 (0.07%)	30 (0.13%)	52 (0.1%)
Acc Aha 201310yrcvdrisk	0.200 (0.163)	0.153 (0.137)	0.180 (0.154)
Missing	2,722 (9.024%)	2,653 (11.688%)	5,375 (10.168%)
Chf Dx Status			
0	28,174 (93.4%)	20,867 (91.93%)	49,041 (92.77%)
1	1,797 (5.96%)	1,657 (7.3%)	3,454 (6.53%)
999	194 (0.64%)	175 (0.77%)	369 (0.7%)
Cv Risk Subgrp			
Low	9,598 (31.82%)	9,090 (40.05%)	18,688 (35.35%)
Moderate	6,449 (21.38%)	4,835 (21.3%)	11,284 (21.35%)
High	10,563 (35.02%)	5,415 (23.86%)	15,978 (30.22%)
Other	899 (2.98%)	758 (3.34%)	1,657 (3.13%)
Unknown	2,656 (8.8%)	2,601 (11.46%)	5,257 (9.94%)
Diab Duration	6.68 (2.89)	7.36 (3.50)	6.97 (3.18)
Missing	37 (0.12%)	9 (0.04%)	46 (0.09%)
A1c	8.55 (1.49)	8.94 (1.77)	8.72 (1.63)
Missing	22 (0.07%)	30 (0.13%)	52 (0.1%)
Acr	52.18 (92.96)	61.33 (101.03)	55.92 (96.44)
Missing	4,103 (13.6%)	4,686 (20.64%)	8,789 (16.63%)
Afib Dx	1,711 (5.67%)	1,177 (5.19%)	2,888 (5.46%)
Alt	30.50 (20.27)	32.10 (21.31)	31.17 (20.73)
Missing	5,303 (17.58%)	4,573 (20.15%)	9,876 (18.68%)
Amputation Dxpx	277 (0.92%)	435 (1.92%)	712 (1.35%)
Anemia Dx	3,231 (10.71%)	2,541 (11.19%)	5,772 (10.92%)
Anxiety Dx	5,800 (19.23%)	6,355 (28%)	12,155 (22.99%)
Arrythmia Dx	1,666 (5.52%)	1,342 (5.91%)	3,008 (5.69%)
Ascvd Dxpx Max	4,049 (13.42%)	2,984 (13.15%)	7,033 (13.3%)
Ascvd Dxpx Ppv	899 (2.98%)	758 (3.34%)	1,657 (3.13%)
Asthma Dx	3,033 (10.05%)	3,497 (15.41%)	6,530 (12.35%)
Bariatric Px	333 (1.1%)	602 (2.65%)	935 (1.77%)

© 2025 Neugebauer R et al. *JAMA Network Open*.

	DPP4i n = 30,165	GLP-1RA n = 22,699	Total n = 52,864
Bipolar Dx	280 (0.93%)	415 (1.83%)	695 (1.31%)
Blind Dx	141 (0.47%)	70 (0.31%)	211 (0.4%)
Cad Dxpx Max	2,603 (8.63%)	1,861 (8.2%)	4,464 (8.44%)
Cad Dxpx Ppv	574 (1.9%)	536 (2.36%)	1,110 (2.1%)
Cad Dxpx Sens	660 (2.19%)	603 (2.66%)	1,263 (2.39%)
Cancer Mets Dx	0 (0%)	0 (0%)	0 (0%)
Cancer Nomets Dx	1,541 (5.11%)	1,481 (6.52%)	3,022 (5.72%)
Cevd Dxpx Ppv	277 (0.92%)	173 (0.76%)	450 (0.85%)
Cevd Dxpx Sens	1,267 (4.2%)	970 (4.27%)	2,237 (4.23%)
Chf Dx Ppv	368 (1.22%)	339 (1.49%)	707 (1.34%)
Chf Dx Sens	1,797 (5.96%)	1,657 (7.3%)	3,454 (6.53%)
Ckd Dx	9,645 (31.97%)	6,891 (30.36%)	16,536 (31.28%)
Copd Dx	1,475 (4.89%)	1,293 (5.7%)	2,768 (5.24%)
Coupled Dbp	72.36 (10.50)	73.88 (10.90)	73.01 (10.70)
Missing	498 (1.65%)	391 (1.72%)	889 (1.68%)
Coupled Sbp	128.98 (14.15)	130.00 (14.32)	129.42 (14.24)
Missing	498 (1.65%)	391 (1.72%)	889 (1.68%)
Covid Prd	5,753 (19.07%)	12,353 (54.42%)	18,106 (34.25%)
Creat	0.99 (0.65)	0.94 (0.49)	0.97 (0.59)
Missing	445 (1.48%)	467 (2.06%)	912 (1.73%)
Cysticfibrosis Dx	0 (0%)	0 (0%)	0 (0%)
Dbp	71.67 (10.55)	73.22 (10.98)	72.33 (10.76)
Missing	498 (1.65%)	391 (1.72%)	889 (1.68%)
Dementia Dx	0 (0%)	0 (0%)	0 (0%)
Depr Dx	4,659 (15.45%)	5,319 (23.43%)	9,978 (18.87%)
Dietitian	1,990 (6.6%)	3,149 (13.87%)	5,139 (9.72%)
Dka Dx	301 (1%)	559 (2.46%)	860 (1.63%)
Dka Dx Count	0.01 (0.12)	0.03 (0.23)	0.02 (0.18)
Esrd Dx	2,590 (8.59%)	2,035 (8.97%)	4,625 (8.75%)
Esrd Px	204 (0.68%)	104 (0.46%)	308 (0.58%)
Etoh Dx	426 (1.41%)	417 (1.84%)	843 (1.59%)
Fasciitis Dx	24 (0.08%)	70 (0.31%)	94 (0.18%)
Fpg	169.33 (59.32)	180.96 (74.68)	173.56 (65.56)
Missing	22,637 (75.04%)	18,390 (81.02%)	41,027 (77.61%)
Frailty Dx	3,114 (10.32%)	3,052 (13.45%)	6,166 (11.66%)
Gfr Epi 09	81.13 (24.72)	85.56 (24.19)	83.02 (24.59)
Missing	445 (1.48%)	467 (2.06%)	912 (1.73%)
Hdl	44.40 (11.56)	43.00 (11.29)	43.81 (11.47)
Missing	3,319 (11%)	3,000 (13.22%)	6,319 (11.95%)
Hgb	13.54 (1.62)	13.65 (1.59)	13.59 (1.61)
Missing	7,554 (25.04%)	5,670 (24.98%)	13,224 (25.02%)
Htn Dx	20,743 (68.77%)	15,638 (68.89%)	36,381 (68.82%)
Hypo Dx	68 (0.23%)	80 (0.35%)	148 (0.28%)
Hypo Dx Count	0.02 (0.17)	0.03 (0.31)	0.02 (0.24)
Hypo Dx Event	68 (0.23%)	80 (0.35%)	148 (0.28%)
Hypothyroidism Dx	3,975 (13.18%)	3,253 (14.33%)	7,228 (13.67%)
Ldl	85.00 (34.04)	88.50 (36.48)	86.48 (35.13)
Missing	2,454 (8.14%)	2,461 (10.84%)	4,915 (9.3%)
Leukemia Lymphoma Dx	200 (0.66%)	121 (0.53%)	321 (0.61%)
Lipid Dx	22,498 (74.58%)	15,793 (69.58%)	38,291 (72.43%)
Liver Dx	75 (0.25%)	62 (0.27%)	137 (0.26%)
Mci Dx	202 (0.67%)	126 (0.56%)	328 (0.62%)
Men2 Dx	0 (0%)	0 (0%)	0 (0%)
Nephropathy Dx	2,822 (9.36%)	1,009 (4.45%)	3,831 (7.25%)
Neuro Dx	846 (2.8%)	904 (3.98%)	1,750 (3.31%)
Pancreatitis Dx	0 (0%)	0 (0%)	0 (0%)
Pcr	0.000 (0.000)	0.000 (0.000)	0.000 (0.000)

© 2025 Neugebauer R et al. *JAMA Network Open*.

	DPP4i n = 30,165	GLP-1RA n = 22,699	Total n = 52,864
Missing	28,088 (93.115%)	20,946 (92.277%)	49,034 (92.755%)
Potassium	4.29 (0.42)	4.26 (0.42)	4.28 (0.42)
Missing	992 (3.29%)	944 (4.16%)	1,936 (3.66%)
Pregnancy	0 (0%)	0 (0%)	0 (0%)
Pud Dx	40 (0.13%)	27 (0.12%)	67 (0.13%)
Pvd Dxpx Ppv	113 (0.37%)	92 (0.41%)	205 (0.39%)
Pvd Dxpx Sens	865 (2.87%)	723 (3.19%)	1,588 (3%)
Pyelo Dx	320 (1.06%)	482 (2.12%)	802 (1.52%)
Retinopathy Dxpx	2,128 (7.05%)	1,277 (5.63%)	3,405 (6.44%)
Rpg	191.74 (84.46)	202.18 (92.78)	196.77 (88.71)
Missing	17,083 (56.63%)	10,555 (46.5%)	27,638 (52.28%)
Sbp	128.35 (14.06)	129.36 (14.19)	128.78 (14.13)
Missing	498 (1.65%)	391 (1.72%)	889 (1.68%)
Schiz Dx	197 (0.65%)	160 (0.7%)	357 (0.68%)
Sodium	138.40 (2.90)	138.59 (2.90)	138.48 (2.90)
Missing	3,196 (10.6%)	2,770 (12.2%)	5,966 (11.29%)
Sud Dx	247 (0.82%)	328 (1.44%)	575 (1.09%)
Tc	160.95 (43.35)	165.49 (46.53)	162.87 (44.78)
Missing	3,271 (10.84%)	2,971 (13.09%)	6,242 (11.81%)
Trig	185.74 (141.83)	204.06 (171.33)	193.32 (154.97)
Missing	4,627 (15.34%)	4,700 (20.71%)	9,327 (17.64%)
Tsh	2.05 (1.78)	2.10 (1.96)	2.07 (1.86)
Missing	11,414 (37.84%)	8,233 (36.27%)	19,647 (37.17%)
Valvular Dx	908 (3.01%)	583 (2.57%)	1,491 (2.82%)
Vasculitis Dx	422 (1.4%)	254 (1.12%)	676 (1.28%)
Only Met No Ascvd	3,452 (11.44%)	2,677 (11.79%)	6,129 (11.59%)
Renal Function Status			
Lowrisk	15,608 (51.74%)	10,325 (45.49%)	25,933 (49.06%)
Moderaterisk	6,455 (21.4%)	4,959 (21.85%)	11,414 (21.59%)
Highrisk	2,450 (8.12%)	1,825 (8.04%)	4,275 (8.09%)
Veryhighrisk	1,454 (4.82%)	816 (3.59%)	2,270 (4.29%)
Unknown	4,198 (13.92%)	4,774 (21.03%)	8,972 (16.97%)
Total Visit C	0.77 (2.68)	1.02 (3.22)	0.88 (2.93)
Total Visit E	0.46 (2.15)	1.06 (3.34)	0.72 (2.74)
Total Visit N	0.41 (3.83)	0.35 (2.43)	0.39 (3.30)
**Concurrent medications**			
Aa	0 (0%)	8 (0.04%)	8 (0.02%)
Aceinhibitors	13,310 (44.12%)	9,311 (41.02%)	22,621 (42.79%)
Agi	511 (1.69%)	160 (0.7%)	671 (1.27%)
Anticoagulants	1,514 (5.02%)	1,234 (5.44%)	2,748 (5.2%)
Anticonvulsants	2,672 (8.86%)	3,725 (16.41%)	6,397 (12.1%)
Antidepressantcomb	0 (0%)	1 (0%)	1 (0%)
Antidepressantmaoi	6 (0.02%)	2 (0.01%)	8 (0.02%)
Antidepressantndri	647 (2.14%)	1,031 (4.54%)	1,678 (3.17%)
Antidepressantother	0 (0%)	0 (0%)	0 (0%)
Antidepressantsari	855 (2.83%)	1,072 (4.72%)	1,927 (3.65%)
Antidepressantsnri	1,110 (3.68%)	1,817 (8%)	2,927 (5.54%)
Antidepressantspo	18 (0.06%)	32 (0.14%)	50 (0.09%)
Antidepressantssri	2,874 (9.53%)	3,482 (15.34%)	6,356 (12.02%)
Antidepressanttca	998 (3.31%)	939 (4.14%)	1,937 (3.66%)
Antidepressantteca	209 (0.69%)	218 (0.96%)	427 (0.81%)
Antiplatelets	2,588 (8.58%)	2,398 (10.56%)	4,986 (9.43%)
Antipsychotic1stgen	83 (0.28%)	53 (0.23%)	136 (0.26%)
Antipsychotic2ndgen	510 (1.69%)	654 (2.88%)	1,164 (2.2%)
Anxiety	411 (1.36%)	672 (2.96%)	1,083 (2.05%)
Arb	7,324 (24.28%)	5,923 (26.09%)	13,247 (25.06%)
Benzodiazepines	1,439 (4.77%)	1,157 (5.1%)	2,596 (4.91%)

© 2025 Neugebauer R et al. *JAMA Network Open*.

	DPP4i<br>n = 30,165	GLP-1RA<br>n = 22,699	Total<br>n = 52,864
Betablockers	9,325 (30.91%)	7,119 (31.36%)	16,444 (31.11%)
Clonidine	427 (1.42%)	283 (1.25%)	710 (1.34%)
Dihydropyridineccb	5,521 (18.3%)	4,061 (17.89%)	9,582 (18.13%)
Hypnoticother	312 (1.03%)	342 (1.51%)	654 (1.24%)
Injectableantipsychotic	5 (0.02%)	10 (0.04%)	15 (0.03%)
Ins	4,773 (15.82%)	12,473 (54.95%)	17,246 (32.62%)
Ins Analog	1,697 (5.63%)	5,048 (22.24%)	6,745 (12.76%)
Ins Combo	484 (1.6%)	1,481 (6.52%)	1,965 (3.72%)
Ins Human	3,372 (11.18%)	8,394 (36.98%)	11,766 (22.26%)
Ins La	4,131 (13.69%)	10,138 (44.66%)	14,269 (26.99%)
Ins Sa	1,587 (5.26%)	5,824 (25.66%)	7,411 (14.02%)
Ksparingdiuretics	1,160 (3.85%)	1,232 (5.43%)	2,392 (4.52%)
Lithium	38 (0.13%)	53 (0.23%)	91 (0.17%)
Loopdiuretics	2,543 (8.43%)	2,478 (10.92%)	5,021 (9.5%)
Meg	178 (0.59%)	163 (0.72%)	341 (0.65%)
Met	23,231 (77.01%)	16,769 (73.88%)	40,000 (75.67%)
Nondihydropyridineccb	746 (2.47%)	522 (2.3%)	1,268 (2.4%)
Otherlipidmeds	1,303 (4.32%)	1,122 (4.94%)	2,425 (4.59%)
Pcsk9mab	9 (0.03%)	32 (0.14%)	41 (0.08%)
Sglt2	1,014 (3.36%)	2,428 (10.7%)	3,442 (6.51%)
Statins	22,076 (73.18%)	16,565 (72.98%)	38,641 (73.1%)
Stimulants	116 (0.38%)	216 (0.95%)	332 (0.63%)
Sunew	21,060 (69.82%)	9,320 (41.06%)	30,380 (57.47%)
Suold	15 (0.05%)	2 (0.01%)	17 (0.03%)
Thiazidediuretics	7,956 (26.37%)	6,078 (26.78%)	14,034 (26.55%)
Tir	0 (0%)	0 (0%)	0 (0%)
Tzd	2,141 (7.1%)	1,094 (4.82%)	3,235 (6.12%)
Only Met Therapy	3,967 (13.15%)	2,952 (13%)	6,919 (13.09%)

© 2025 Neugebauer R et al. *JAMA Network Open*.

eFigure 29. MACE (Primary Definition), 2-Arm Drug Class Comparison, DPP4is vs GLP-1RAs, Cumulative Incidence Curves From PP and ITT Analyses With IPW, TMLE, and SL
Each plot emulates inferences from a 2-arm RCT comparing DPP4i and GLP-1RA and represents unadjusted or adjusted estimates of cumulative incidence curves for MACE derived with inverse probability weighting (IPW) and Targeted Minimum Loss-based Estimation (TMLE) with Super Learning (SL) estimates of propensity scores with four weight truncation schemes: IPW and TMLE without weight truncation (untruncated), IPW with truncation of stabilized weights at value 20 (trunc20) or at the 99th percentile of weight values (trunc99), and TMLE with truncation of unstabilized weights at value 200 (trunc200). The red divider line separates results of Per-Protocol (PP) analyses (top half) from Intention-To-Treat (ITT) analyses (bottom half). Each plot displays a p value for the test that the average risk difference (ARD) through 2.5 years of follow-up (30 months) is 0.

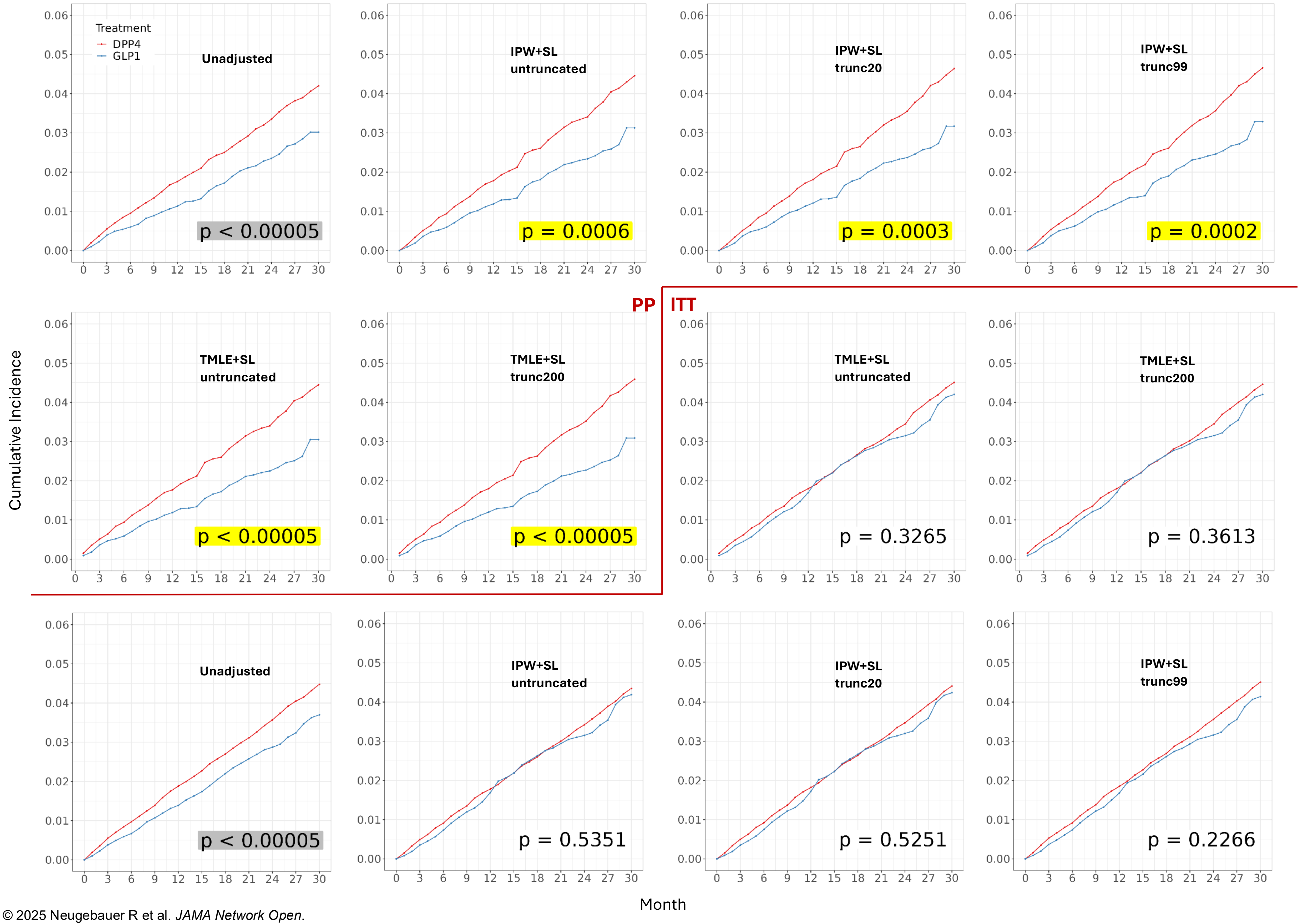

© 2025 Neugebauer R et al. *JAMA Network Open*.

eFigure 30. MACE (Primary Definition), 2-Arm Drug Class Comparison, DPP4is vs GLP-1RAs, Cumulative Incidence Curves From Sensitivity PP Analyses With IPW, TMLE, and SL
Each plot emulates inferences from a 2-arm RCT comparing DPP4i and GLP-1RA and represents unadjusted or adjusted estimates of cumulative incidence curves for MACE from sensitivity PP analyses referred to as "NoMBS PP" and "No3 PP". NoMBS PP analyses are restricted to patients without a history of MBS at baseline and the protocols they evaluate preclude metabolic bariatric surgery (MBS) procedures. The protocols in the No3 PP analyses preclude exposure to three medication classes: the comparator medication from the other arm, SGLT2i and SU.

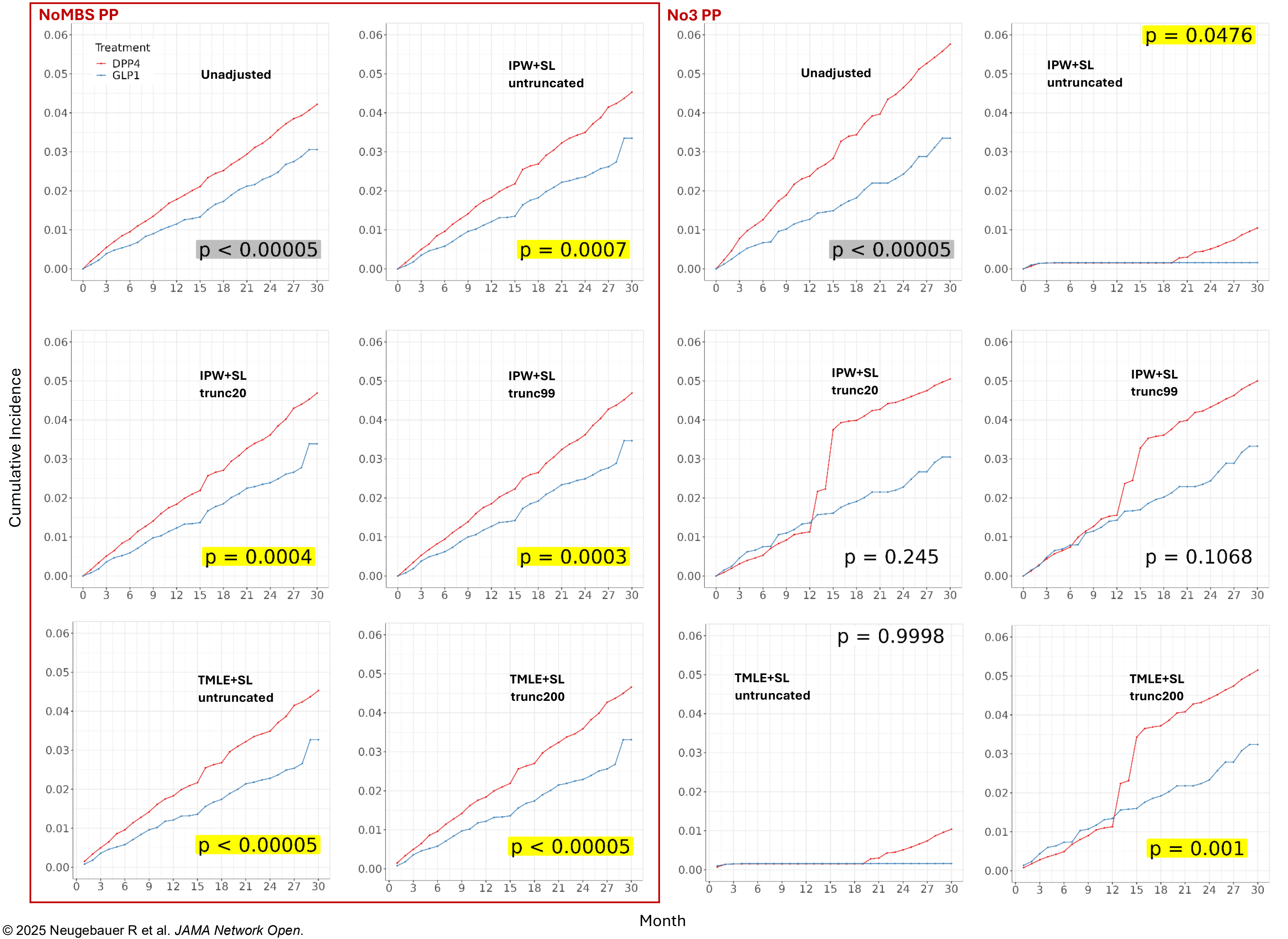

© 2025 Neugebauer R et al. *JAMA Network Open*.

eTable 30. MACE (Primary Definition), 2-Arm Drug Class Comparison, DPP4is vs GLP-1RAs, RD and HR Effect Measures at 2.5 Years

Estimation results from ITT, PP, NoMBS PP, and No3 PP analyses of emulated 2-arm RCTs comparing MACE risks over 2.5 years between DPP4i and GLP-1RA initiators. For PP analyses, rates of protocol deviations are described by medication class initiated at baseline. Unadjusted point and interval estimates and adjusted point and interval IPW and TMLE estimates of risks, risk differences (RD), and hazard ratios (HR) based on propensity scores (PS) estimated with either logistic models or super learning (SL) are presented for four weight truncation schemes along with the corresponding 99th percentile and maximum value of the stabilized and unstabilized inverse probability weights used for implementing IPW and TMLE, respectively. RD is the risk in treatment arm minus the risk in control arm and NNT is the number needed to treat.

Analysis type	Protocol Deviations* by exposure group (%)	PS estimation	99th IP weights	Max IP weight	Estimator	Treatment (DPP4i) risk in %	Control (GLP-1RA) risk in %	RD [95% CI] in %	NNT	HR [95% CI]
PP	Discontinuation<br>DPP4i: 62.29<br>GLP-1RA: 45.37<br><br>Crossover<br>DPP4i: 6.00<br>GLP-1RA: 1.33	SL			Unadjusted	4.20	3.02	1.19 [0.62, 1.75]	84	1.51 [1.26, 1.76]
			24.18	36,201.44	TMLE untruncated	4.45	3.05	1.40 [0.67, 2.13]	71	
					TMLE truncated at 200	4.59	3.09	1.51 [0.80, 2.22]	66	
			5.98	6,100.37	IPW untruncated	4.46	3.13	1.33 [0.24, 2.42]	75	1.51 [1.11, 1.92]
					IPW truncated at 20	4.64	3.17	1.47 [0.38, 2.56]	68	1.51 [1.11, 1.91]
					IPW truncated at 99th	4.66	3.29	1.37 [0.31, 2.42]	73	1.45 [1.10, 1.80]
		Logistic model	7.28	6,698.52	IPW untruncated	4.08	4.32	-0.25 [-3.93, 3.44]		1.58 [1.03, 2.14]
					IPW truncated at 20	4.87	3.96	0.92 [-1.57, 3.40]		1.42 [0.98, 1.86]
					IPW truncated at 99th	4.62	3.40	1.22 [-0.05, 2.48]		1.39 [1.02, 1.75]
ITT		SL			Unadjusted	4.48	3.70	0.78 [0.35, 1.21]	128	1.29 [1.12, 1.45]
			15.05	1,942.65	TMLE untruncated	4.51	4.20	0.31 [-0.29, 0.92]		
					TMLE truncated at 200	4.46	4.20	0.26 [-0.33, 0.86]		
			5.38	801.46	IPW untruncated	4.35	4.19	0.16 [-0.67, 0.99]		1.03 [0.80, 1.27]
					IPW truncated at 20	4.41	4.24	0.17 [-0.67, 1.01]		1.03 [0.80, 1.27]
					IPW truncated at 99th	4.51	4.14	0.36 [-0.34, 1.06]		1.08 [0.87, 1.28]
		Logistic model	6.25	785.29	IPW untruncated	4.23	4.21	0.02 [-0.88, 0.93]		0.99 [0.73, 1.25]
					IPW truncated at 20	4.35	4.33	0.02 [-0.89, 0.92]		0.99 [0.74, 1.24]
					IPW truncated at 99th	4.41	4.19	0.22 [-0.54, 0.98]		1.05 [0.84, 1.27]
NoMBS PP	Discontinuation<br>DPP4i: 62.07<br>GLP-1RA: 44.67<br><br>Crossover<br>DPP4i: 6.28<br>GLP-1RA: 2.39<br><br>MBS occurrence<br>DPP4i: 0.33<br>GLP-1RA: 1.07	SL			Unadjusted	4.22	3.06	1.16 [0.58, 1.73]	86	1.51 [1.26, 1.77]
			23.83	32,735.70	TMLE untruncated	4.53	3.27	1.25 [0.43, 2.08]	80	
					TMLE truncated at 200	4.66	3.31	1.35 [0.54, 2.15]	74	
			5.86	5,556.06	IPW untruncated	4.53	3.35	1.18 [-0.16, 2.52]		1.55 [1.13, 1.98]
					IPW truncated at 20	4.69	3.39	1.30 [-0.04, 2.64]		1.53 [1.12, 1.94]
					IPW truncated at 99th	4.69	3.47	1.22 [-0.00, 2.45]	82	1.46 [1.11, 1.82]
		Logistic model	7.13	7,102.69	IPW untruncated	4.12	5.48	-1.37 [-6.98, 4.24]		1.54 [1.02, 2.07]
					IPW truncated at 20	4.94	4.05	0.89 [-1.77, 3.55]		1.45 [1.00, 1.90]
					IPW truncated at 99th	4.65	3.43	1.22 [-0.10, 2.53]		1.41 [1.04, 1.78]
No3 PP	Discontinuation<br>DPP4i: 12.11<br>GLP-1RA: 21.63<br><br>Crossover to comparator drug<br>DPP4i: 1.51<br>GLP-1RA: 0.44<br><br>Initiation of one of the two non-comparator drugs<br>DPP4i: 79.20<br>GLP-1RA: 53.62	SL			Unadjusted	5.76	3.35	2.40 [1.23, 3.57]	42	1.70 [0.86, 2.55]
			34.49	6.81e+23	TMLE untruncated	1.04	0.16	0.88 [-3691.68, 3693.44]		
					TMLE truncated at 200	5.15	3.24	1.91 [0.85, 2.96]	52	
			5.04	1.51e+22	IPW untruncated	1.05	0.16	0.89 [0.42, 1.35]	112	1.06 [0.02, 2.09]
					IPW truncated at 20	5.05	3.05	1.99 [-1.49, 5.47]		0.75 [0.23, 1.27]
					IPW truncated at 99th	5.00	3.33	1.67 [-0.55, 3.90]		0.96 [0.29, 1.63]
		Logistic model	5.72	3.42e+22	IPW untruncated	0.91	0.06	0.85 [0.45, 1.25]	117	6.87 [-9.20, 22.95]
					IPW truncated at 20	4.92	3.17	1.75 [-2.01, 5.52]		0.62 [0.08, 1.16]
					IPW truncated at 99th	4.84	3.42	1.42 [-1.01, 3.84]		0.76 [0.09, 1.44]

* Discontinuation refers to the interruption of the comparator medication initiated on index date; Crossover refers to the initiation of the comparator medication initiated by patient at baseline in the other arm; MBS occurrence refers to patient's undergoing metabolic bariatric surgery (MBS).

© 2025 Neugebauer R et al. *JAMA Network Open*.

eFigure 31. MACE (Primary Definition), 2-Arm Drug Class Comparison, DPP4is vs GLP-1RAs, ASCVD Subgroup, Cumulative Incidence Curves From PP and ITT Analyses With IPW, TMLE, and SL
Each plot emulates inferences among patients with ASCVD from a 2-arm RCT comparing DPP4i and GLP-1RA and represents unadjusted or adjusted estimates of cumulative incidence curves for MACE derived with IPW and TMLE with SL estimates of propensity scores with four weight truncation schemes: IPW and TMLE without weight truncation (untruncated), IPW with truncation of stabilized weights at value 20 (trunc20) or at the 99th percentile of weight values (trunc99), and TMLE with truncation of unstabilized weights at value 200 (trunc200). The red divider line separates results of Per-Protocol (PP) analyses (top half) from Intention-To-Treat (ITT) analyses (bottom half). Each plot displays a p value for the test that the average risk difference (ARD) through 2.5 years of follow-up (30 months) is 0.

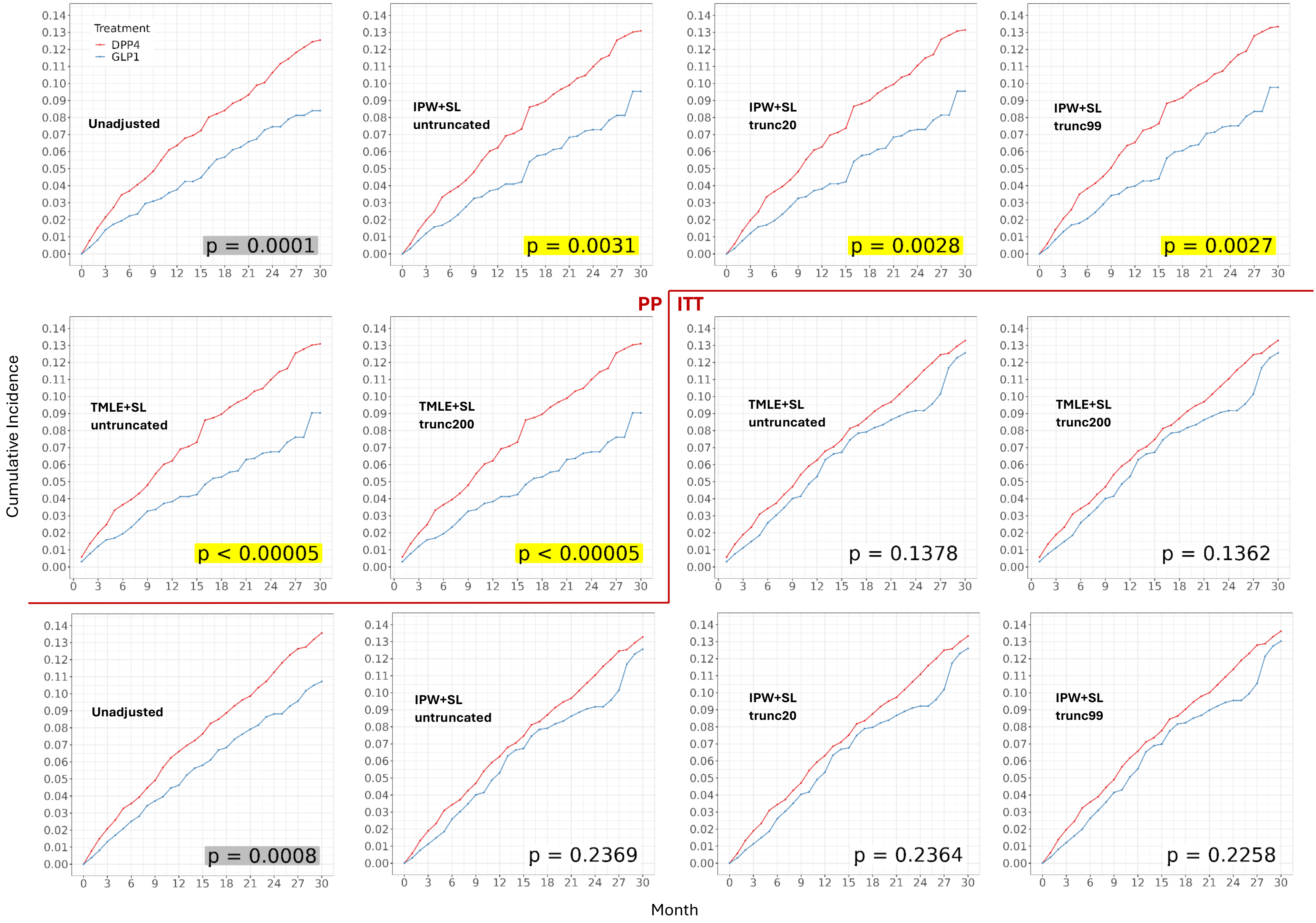

© 2025 Neugebauer R et al. *JAMA Network Open*.

eTable 31. MACE (Primary Definition), 2-Arm Drug Class Comparison, DPP4is vs GLP-1RAs, ASCVD Subgroup, RD and HR Effect Measures at 2.5 Years

Estimation results among patients with ASCVD from ITT and PP analyses of emulated 2-arm RCTs comparing MACE risks over 2.5 years between DPP4i and GLP-1RA initiators. For PP analyses, rates of protocol deviations are described by medication class initiated at baseline. Unadjusted point and interval estimates and adjusted point and interval IPW and TMLE estimates of risks, risk differences (RD), and hazard ratios (HR) based on propensity scores (PS) estimated with either logistic models or super learning (SL) are presented for four weight truncation schemes along with the corresponding 99th percentile and maximum value of the stabilized and unstabilized inverse probability weights used for implementing IPW and TMLE, respectively. RD is the risk in treatment arm minus the risk in control arm and NNT is the number needed to treat.

Analysis type	Protocol Deviations* by exposure group (%)	PS estimation	99th IP weights	Max IP weight	Estimator	Treatment (DPP4i) risk in %	Control (GLP-1RA) risk in %	RD [95% CI] in %	NNT	HR [95% CI]
PP	<u>Discontinuation</u><br>DPP4i: 59.47<br>GLP-1RA: 45.64<br><br><u>Crossover</u><br>DPP4i: 6.55<br>GLP-1RA: 1.85	SL	[redacted]	[redacted]	Unadjusted	12.55	8.40	4.15 [1.74, 6.57]	24	1.59 [1.20, 1.97]
			18.54	256.22	TMLE untruncated	13.09	9.04	4.05 [1.74, 6.37]	25	[redacted]
					TMLE truncated at 200	13.10	9.04	4.06 [1.75, 6.37]	25	
			4.72	77.37	IPW untruncated	13.09	9.54	3.55 [-0.78, 7.88]		1.64 [1.09, 2.19]
					IPW truncated at 20	13.15	9.55	3.59 [-0.74, 7.92]		1.64 [1.10, 2.19]
					IPW truncated at 99th	13.34	9.77	3.57 [-0.73, 7.87]		1.62 [1.09, 2.16]
		Logistic model	5.89	165.68	IPW untruncated	15.02	9.88	5.14 [-2.25, 12.54]		1.62 [1.00, 2.24]
					IPW truncated at 20	15.60	9.95	5.65 [-1.70, 13.01]		1.67 [1.04, 2.30]
					IPW truncated at 99th	13.79	10.46	3.33 [-2.30, 8.96]		1.56 [1.01, 2.11]
ITT	[redacted]	SL	[redacted]	[redacted]	Unadjusted	13.57	10.72	2.86 [0.93, 4.78]	35	1.31 [1.05, 1.57]
			13.98	306.03	TMLE untruncated	13.28	12.56	0.73 [-1.63, 3.08]		[redacted]
					TMLE truncated at 200	13.29	12.56	0.73 [-1.63, 3.09]		
			4.84	143.40	IPW untruncated	13.28	12.56	0.72 [-2.89, 4.32]		1.13 [0.79, 1.47]
					IPW truncated at 20	13.33	12.60	0.72 [-2.88, 4.32]		1.13 [0.79, 1.46]
					IPW truncated at 99th	13.62	13.03	0.59 [-3.05, 4.22]		1.13 [0.81, 1.45]
		Logistic model	5.82	140.71	IPW untruncated	13.11	11.87	1.24 [-2.75, 5.22]		1.11 [0.74, 1.48]
					IPW truncated at 20	13.38	12.47	0.91 [-2.94, 4.75]		1.09 [0.74, 1.43]
					IPW truncated at 99th	13.78	13.13	0.65 [-3.19, 4.48]		1.07 [0.76, 1.39]

* Discontinuation refers to the interruption of the comparator medication initiated on index date; Crossover refers to the initiation of the comparator medication initiated by patient at baseline in the other arm.

© 2025 Neugebauer R et al. *JAMA Network Open*.

eFigure 32. MACE (Primary Definition), 2-Arm Drug Class Comparison, DPP4is vs GLP-1RAs, No ASCVD Subgroup, Cumulative Incidence Curves From PP and ITT Analyses With IPW, TMLE, and SL
Each plot emulates inferences among patients with No ASCVD from a 2-arm RCT comparing DPP4i and GLP-1RA and represents unadjusted or adjusted estimates of cumulative incidence curves for MACE derived with IPW and TMLE with SL estimates of propensity scores with four weight truncation schemes: IPW and TMLE without weight truncation (untruncated), IPW with truncation of stabilized weights at value 20 (trunc20) or at the 99th percentile of weight values (trunc99), and TMLE with truncation of unstabilized weights at value 200 (trunc200). The red divider line separates results of Per-Protocol (PP) analyses (top half) from Intention-To-Treat (ITT) analyses (bottom half). Each plot displays a p value for the test that the average risk difference (ARD) through 2.5 years of follow-up (30 months) is 0.

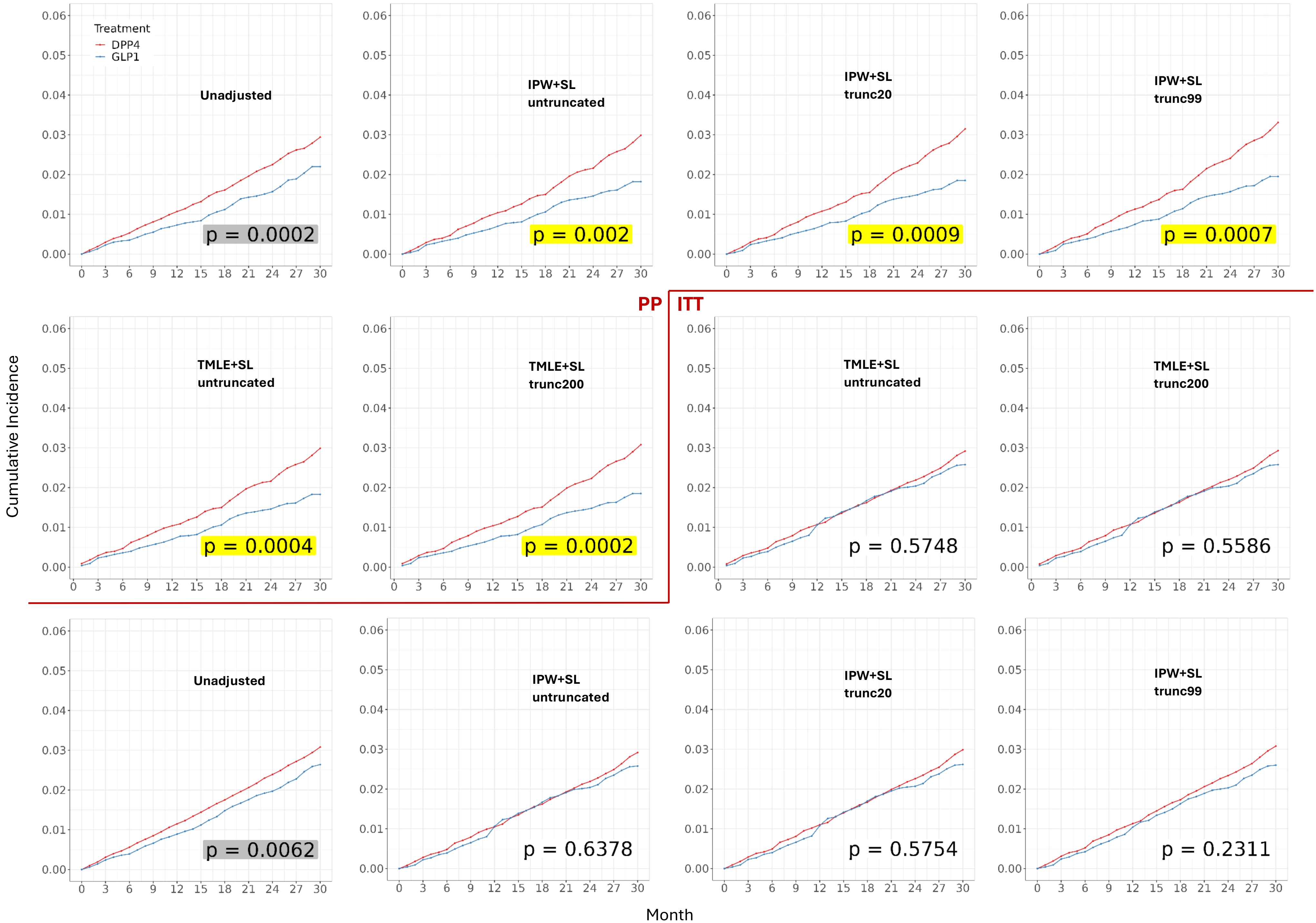

© 2025 Neugebauer R et al. *JAMA Network Open*.

eTable 32. MACE (Primary Definition), 2-Arm Drug Class Comparison, DPP4is vs GLP-1RAs, No ASCVD Subgroup, RD and HR Effect Measures at 2.5 Years

Estimation results among patients with No ASCVD from ITT and PP analyses of emulated 2-arm RCTs comparing MACE risks over 2.5 years between DPP4i and GLP-1RA initiators. For PP analyses, rates of protocol deviations are described by medication class initiated at baseline. Unadjusted point and interval estimates and adjusted point and interval IPW and TMLE estimates of risks, risk differences (RD), and hazard ratios (HR) based on propensity scores (PS) estimated with either logistic models or super learning (SL) are presented for four weight truncation schemes along with the corresponding 99th percentile and maximum value of the stabilized and unstabilized inverse probability weights used for implementing IPW and TMLE, respectively. RD is the risk in treatment arm minus the risk in control arm and NNT is the number needed to treat.

Analysis type	Protocol Deviations* by exposure group (%)	PS estimation	99th IP weights	Max IP weight	Estimator	Treatment (DPP4i) risk in %	Control (GLP-1RA) risk in %	RD [95% CI] in %	NNT	HR [95% CI]
PP	<u>Discontinuation</u><br>DPP4i: 62.73<br>GLP-1RA: 45.33<br><br><u>Crossover</u><br>DPP4i: 5.91<br>GLP-1RA: 1.24	SL			Unadjusted	2.94	2.20	0.74 [0.21, 1.27]	134	1.47 [1.14, 1.80]
			25.78	25,521.54	TMLE untruncated	2.99	1.83	1.17 [0.69, 1.65]	86	
					TMLE truncated at 200	3.08	1.85	1.23 [0.76, 1.70]	81	
			6.37	4,338.48	IPW untruncated	2.99	1.82	1.17 [0.54, 1.81]	85	1.48 [0.98, 1.98]
					IPW truncated at 20	3.15	1.85	1.29 [0.65, 1.94]	77	1.51 [1.00, 2.01]
					IPW truncated at 99th	3.31	1.95	1.36 [0.69, 2.02]	74	1.50 [1.01, 1.99]
		Logistic model	7.24	3,163.14	IPW untruncated	2.52	1.63	0.89 [0.13, 1.65]	112	1.41 [0.93, 1.90]
					IPW truncated at 20	3.12	1.74	1.37 [0.59, 2.15]	73	1.44 [0.95, 1.94]
					IPW truncated at 99th	3.18	1.87	1.32 [0.61, 2.03]	76	1.45 [0.96, 1.95]
ITT		SL			Unadjusted	3.08	2.64	0.44 [0.04, 0.83]	228	1.27 [1.04, 1.49]
			15.91	1,740.91	TMLE untruncated	2.92	2.58	0.34 [-0.15, 0.83]		
					TMLE truncated at 200	2.93	2.58	0.35 [-0.15, 0.84]		
			5.75	724.09	IPW untruncated	2.92	2.58	0.34 [-0.28, 0.96]		1.04 [0.71, 1.38]
					IPW truncated at 20	2.99	2.62	0.37 [-0.26, 1.00]		1.06 [0.72, 1.40]
					IPW truncated at 99th	3.08	2.60	0.48 [-0.06, 1.02]		1.14 [0.84, 1.43]
		Logistic model	6.25	827.91	IPW untruncated	2.80	2.66	0.14 [-0.57, 0.85]		0.94 [0.59, 1.28]
					IPW truncated at 20	2.91	2.70	0.21 [-0.51, 0.93]		0.98 [0.63, 1.33]
					IPW truncated at 99th	2.99	2.57	0.42 [-0.13, 0.96]		1.12 [0.82, 1.42]

* Discontinuation refers to the interruption of the comparator medication initiated on index date; Crossover refers to the initiation of the comparator medication initiated by patient at baseline in the other arm.

© 2025 Neugebauer R et al. *JAMA Network Open*.

eFigure 33. MACE (Primary Definition), 2-Arm Drug Class Comparison, DPP4is vs GLP-1RAs, No ASCVD and MET Subgroup, Cumulative Incidence Curves From PP and ITT Analyses With IPW, TMLE, and SL
Each plot emulates inferences among patients with No ASCVD and MET from a 2-arm RCT comparing DPP4i and GLP-1RA and represents unadjusted or adjusted estimates of cumulative incidence curves for MACE derived with IPW and TMLE with SL estimates of propensity scores with four weight truncation schemes: IPW and TMLE without weight truncation (untruncated), IPW with truncation of stabilized weights at value 20 (trunc20) or at the 99th percentile of weight values (trunc99), and TMLE with truncation of unstabilized weights at value 200 (trunc200). The red divider line separates results of Per-Protocol (PP) analyses (top half) from Intention-To-Treat (ITT) analyses (bottom half). Each plot displays a p value for the test that the average risk difference (ARD) through 2.5 years of follow-up (30 months) is 0.

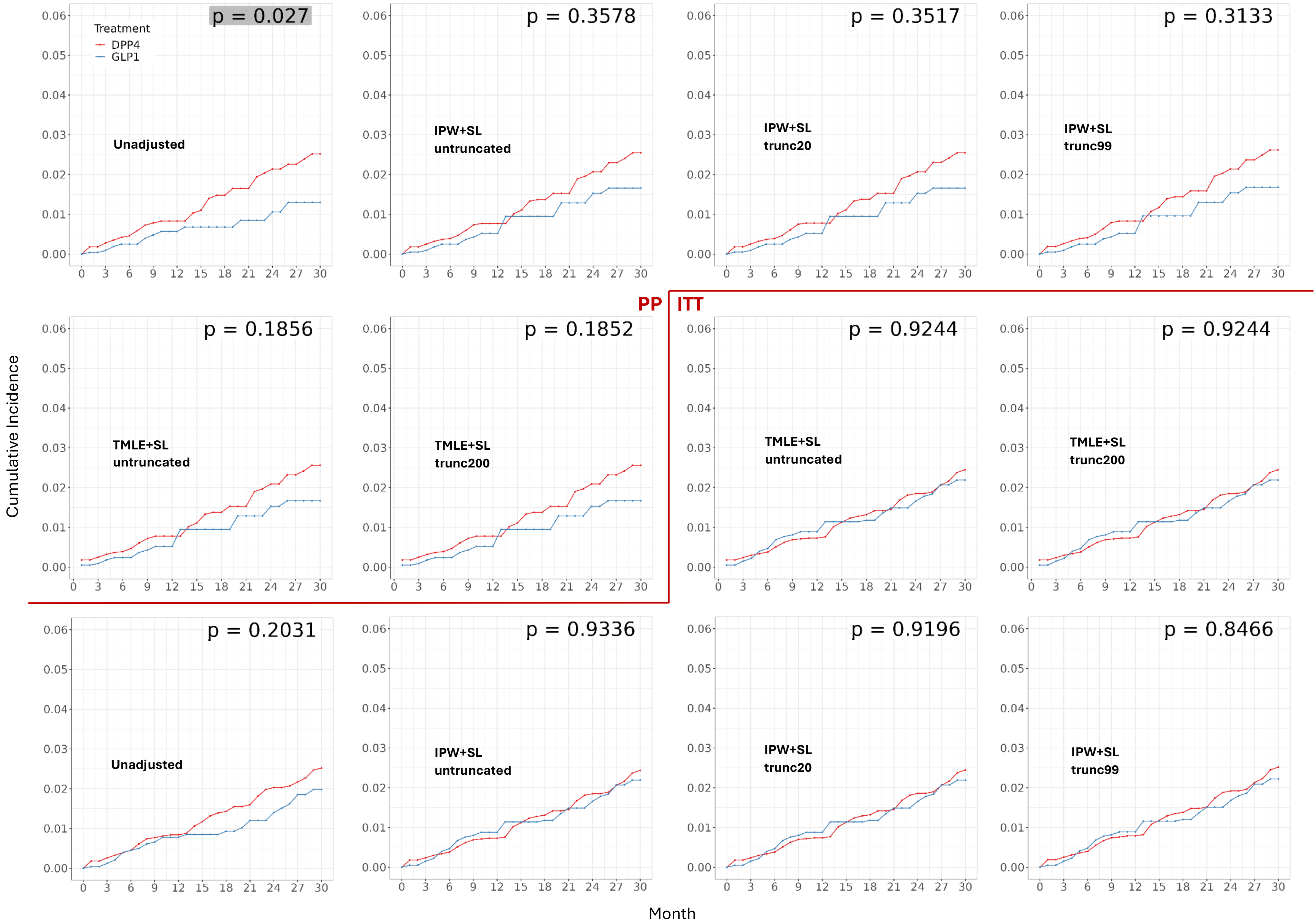

© 2025 Neugebauer R et al. *JAMA Network Open*.

eTable 33. MACE (Primary Definition), 2-Arm Drug Class Comparison, DPP4is vs GLP-1RAs, No ASCVD and MET Subgroup, RD and HR Effect Measures at 2.5 Years

Estimation results among patients with No ASCVD and MET from ITT and PP analyses of emulated 2-arm RCTs comparing MACE risks over 2.5 years between DPP4i and GLP-1RA initiators. For PP analyses, rates of protocol deviations are described by medication class initiated at baseline. Unadjusted point and interval estimates and adjusted point and interval IPW and TMLE estimates of risks, risk differences (RD), and hazard ratios (HR) based on propensity scores (PS) estimated with either logistic models or super learning (SL) are presented for four weight truncation schemes along with the corresponding 99th percentile and maximum value of the stabilized and unstabilized inverse probability weights used for implementing IPW and TMLE, respectively. RD is the risk in treatment arm minus the risk in control arm and NNT is the number needed to treat.

Analysis type	Protocol Deviations* by exposure group (%)	PS estimation	99th IP weights	Max IP weight	Estimator	Treatment (DPP4i) risk in %	Control (GLP-1RA) risk in %	RD [95% CI] in %	NNT	HR [95% CI]
PP	Discontinuation<br>DPP4i: 57.10<br>GLP-1RA: 42.51<br><br>Crossover<br>DPP4i: 7.91<br>GLP-1RA: 1.01	SL	[redacted]		Unadjusted	2.52	1.30	1.23 [0.04, 2.41]	82	1.98 [0.57, 3.39]
			17.35	455.84	TMLE untruncated	2.56	1.67	0.89 [-0.08, 1.87]		[redacted]
					TMLE truncated at 200	2.56	1.67	0.89 [-0.08, 1.87]		
			4.03	124.73	IPW untruncated	2.55	1.66	0.89 [-0.70, 2.47]		1.51 [0.23, 2.79]
					IPW truncated at 20	2.55	1.66	0.89 [-0.69, 2.48]		1.53 [0.23, 2.82]
					IPW truncated at 99th	2.62	1.68	0.94 [-0.66, 2.55]		1.58 [0.24, 2.92]
		Logistic model	5.22	372.93	IPW untruncated	2.42	1.59	0.83 [-0.71, 2.38]		1.25 [0.22, 2.27]
					IPW truncated at 20	2.49	1.60	0.89 [-0.67, 2.45]		1.33 [0.24, 2.41]
					IPW truncated at 99th	2.60	1.65	0.95 [-0.65, 2.55]		1.35 [0.25, 2.45]
ITT	[redacted]	SL	[redacted]		Unadjusted	2.52	1.98	0.54 [-0.45, 1.53]		1.52 [0.67, 2.37]
			11.58	199.96	TMLE untruncated	2.45	2.19	0.26 [-0.57, 1.08]		[redacted]
					TMLE truncated at 200	2.45	2.19	0.26 [-0.57, 1.08]		
			3.86	93.43	IPW untruncated	2.44	2.19	0.25 [-0.95, 1.45]		1.03 [0.32, 1.73]
					IPW truncated at 20	2.45	2.19	0.26 [-0.94, 1.46]		1.04 [0.33, 1.75]
					IPW truncated at 99th	2.52	2.22	0.30 [-0.91, 1.52]		1.08 [0.34, 1.81]
		Logistic model	4.94	198.62	IPW untruncated	2.39	2.05	0.34 [-0.93, 1.60]		0.84 [0.18, 1.51]
					IPW truncated at 20	2.43	2.06	0.38 [-0.90, 1.65]		0.88 [0.19, 1.58]
					IPW truncated at 99th	2.51	2.09	0.43 [-0.83, 1.68]		0.93 [0.23, 1.63]

* Discontinuation refers to the interruption of the comparator medication initiated on index date; Crossover refers to the initiation of the comparator medication initiated by patient at baseline in the other arm.

© 2025 Neugebauer R et al. *JAMA Network Open*.

eFigure 34. MACE (Primary Definition), 2-Arm Drug Class Comparison, SGLT2is vs GLP-1RAs, CONSORT Diagram
Flow diagram describing the inclusion and exclusion steps and counts leading to the creation of the cohort for emulating the 2-arm RCT to compare the risk of MACE in new users of SGLT2i and GLP-1RA along with sample sizes and counts for each observed end of follow-up type by treatment initiated at cohort entry.

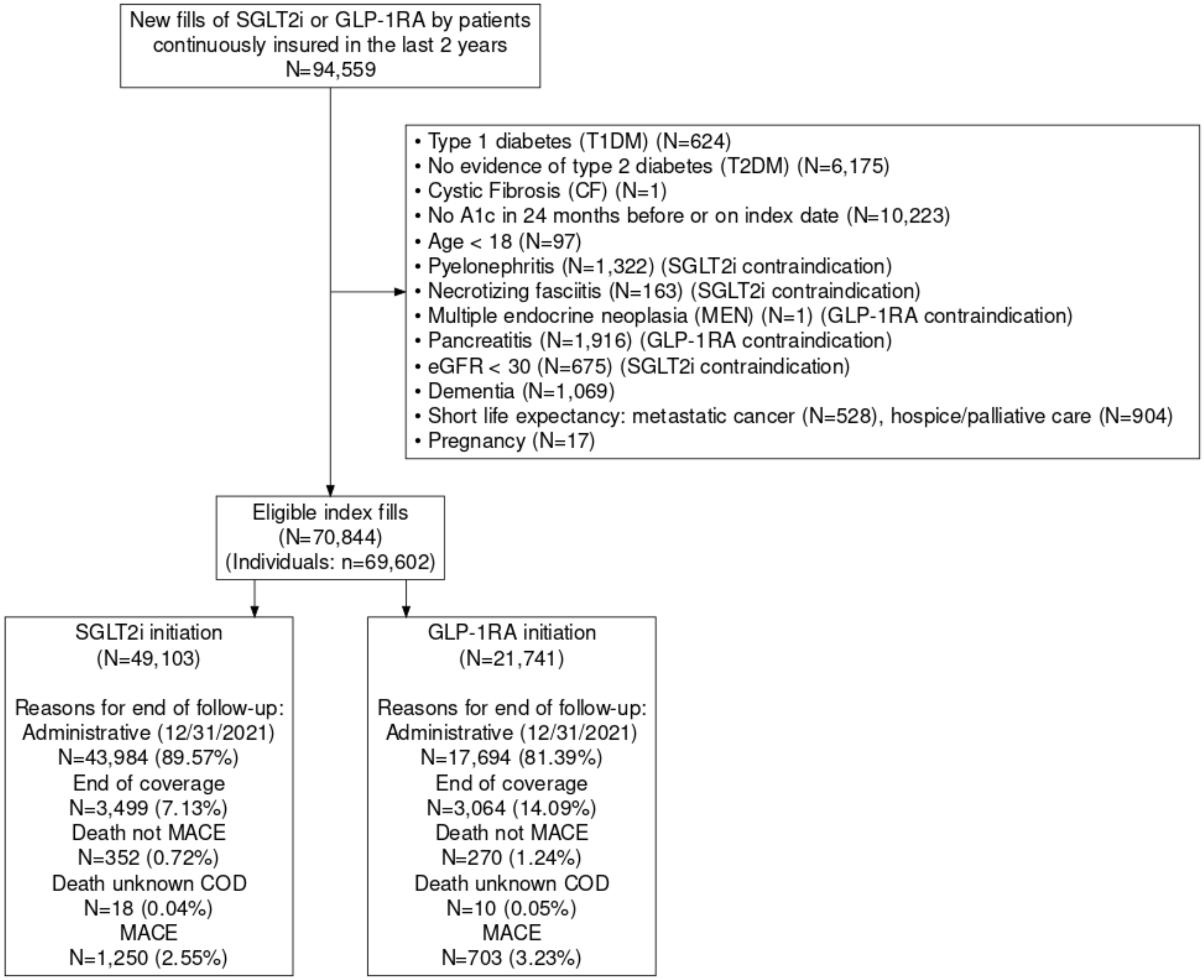

© 2025 Neugebauer R et al. *JAMA Network Open*.

eTable 34. MACE (Primary Definition), 2-Arm Drug Class Comparison, SGLT2is vs GLP-1RAs, Patient Characteristics at Baseline (Overall and by Medication Initiated)
Summary statistics of the baseline values for selected covariates in the cohort of patients used to emulate a 2-arm RCT for comparing SGLT2i and GLP-1RA. For each continuous variable, the mean and standard deviation are displayed for all patients in the cohort (last column) and by drug class initiated at cohort entry. For each categorical variable and for each possible level of that variable, the count and proportion are displayed instead.

	SGLT2i n = 49,103	GLP-1RA n = 21,741	Total n = 70,844
**Demographics**			
Age	60.54 (11.45)	56.51 (11.95)	59.31 (11.76)
Agegrp			
<45	4,395 (8.95%)	3,468 (15.95%)	7,863 (11.1%)
[45-65)	25,866 (52.68%)	12,632 (58.1%)	38,498 (54.34%)
[65-75)	13,997 (28.51%)	4,504 (20.72%)	18,501 (26.12%)
>=75	4,845 (9.87%)	1,137 (5.23%)	5,982 (8.44%)
Ethnicity			
Hispanic	13,624 (27.75%)	5,145 (23.66%)	18,769 (26.49%)
Nonhispanic	35,479 (72.25%)	16,596 (76.34%)	52,075 (73.51%)
Female Head Of Hh	0.139 (0.071)	0.139 (0.076)	0.139 (0.072)
Missing	913 (1.859%)	935 (4.301%)	1,848 (2.609%)
Hh Public Assistance	0.035 (0.033)	0.036 (0.034)	0.036 (0.033)
Missing	913 (1.859%)	935 (4.301%)	1,848 (2.609%)
Household Income Less 30k	0.176 (0.110)	0.204 (0.123)	0.185 (0.114)
Missing	913 (1.859%)	935 (4.301%)	1,848 (2.609%)
Houspoverty	0.083 (0.077)	0.093 (0.086)	0.086 (0.079)
Missing	914 (1.861%)	940 (4.324%)	1,854 (2.617%)
Index Yr			
2014	186 (0.38%)	634 (2.92%)	820 (1.16%)
2015	566 (1.15%)	893 (4.11%)	1,459 (2.06%)
2016	1,450 (2.95%)	1,242 (5.71%)	2,692 (3.8%)
2017	2,299 (4.68%)	1,862 (8.56%)	4,161 (5.87%)
2018	3,183 (6.48%)	3,160 (14.53%)	6,343 (8.95%)
2019	4,743 (9.66%)	3,318 (15.26%)	8,061 (11.38%)
2020	12,137 (24.72%)	4,227 (19.44%)	16,364 (23.1%)
2021	24,539 (49.97%)	6,405 (29.46%)	30,944 (43.68%)
Bmi	33.30 (7.31)	37.48 (8.08)	34.60 (7.80)
Missing	2,071 (4.22%)	587 (2.7%)	2,658 (3.75%)
Smoking Status			
Formersmoker	15,278 (31.11%)	6,875 (31.62%)	22,153 (31.27%)
Currentsmoker	3,664 (7.46%)	2,054 (9.45%)	5,718 (8.07%)
Passivesmoker	204 (0.42%)	100 (0.46%)	304 (0.43%)
Neversmoker	29,286 (59.64%)	12,365 (56.87%)	41,651 (58.79%)
Unknown	671 (1.37%)	347 (1.6%)	1,018 (1.44%)
Low Educ	0.149 (0.117)	0.141 (0.116)	0.147 (0.116)
Missing	913 (1.859%)	933 (4.291%)	1,846 (2.606%)
Mgr Male	0.051 (0.050)	0.044 (0.043)	0.049 (0.048)
Missing	914 (1.861%)	934 (4.296%)	1,848 (2.609%)
Ndi	0.24 (0.15)	0.25 (0.16)	0.24 (0.15)
Missing	915 (1.86%)	940 (4.32%)	1,855 (2.62%)
Pct Crowding	0.082 (0.080)	0.069 (0.078)	0.078 (0.080)
Missing	913 (1.859%)	935 (4.301%)	1,848 (2.609%)
Racegrp			
White	24,078 (49.04%)	13,131 (60.4%)	37,209 (52.52%)
Asian	9,809 (19.98%)	1,854 (8.53%)	11,663 (16.46%)
Blackorafricanamerican	4,416 (8.99%)	2,726 (12.54%)	7,142 (10.08%)
Hawaiianorpacificislander	1,032 (2.1%)	358 (1.65%)	1,390 (1.96%)
Americanindianoralaskanative	319 (0.65%)	120 (0.55%)	439 (0.62%)
Multirace	1,840 (3.75%)	1,155 (5.31%)	2,995 (4.23%)
Other	83 (0.17%)	107 (0.49%)	190 (0.27%)
Unknown	7,526 (15.33%)	2,290 (10.53%)	9,816 (13.86%)
Sex			

© 2025 Neugebauer R et al. *JAMA Network Open*.

	SGLT2i n = 49,103	GLP-1RA n = 21,741	Total n = 70,844
Female	20,968 (42.7%)	11,769 (54.13%)	32,737 (46.21%)
Male	28,134 (57.3%)	9,972 (45.87%)	38,106 (53.79%)
Unknown	1 (0%)	0 (0%)	1 (0%)
Site			
Kpnc	19,054 (38.8%)	4,034 (18.55%)	23,088 (32.59%)
Kpsc	20,418 (41.58%)	8,267 (38.02%)	28,685 (40.49%)
Kphi	3,283 (6.69%)	1,559 (7.17%)	4,842 (6.83%)
Hpi	1,483 (3.02%)	2,747 (12.64%)	4,230 (5.97%)
Hfhs	1,388 (2.83%)	1,810 (8.33%)	3,198 (4.51%)
Ghs	3,477 (7.08%)	3,324 (15.29%)	6,801 (9.6%)
Unemployment	0.039 (0.023)	0.041 (0.026)	0.040 (0.024)
Missing	912 (1.857%)	933 (4.291%)	1,845 (2.604%)
**Insurance**			
Ins Commercial	30,234 (61.57%)	13,925 (64.05%)	44,159 (62.33%)
Ins Highdeductible	2,760 (5.62%)	1,499 (6.89%)	4,259 (6.01%)
Ins Medicaid	4,668 (9.51%)	2,829 (13.01%)	7,497 (10.58%)
Ins Medicare	17,792 (36.23%)	5,987 (27.54%)	23,779 (33.57%)
Ins Medicare A	11,737 (23.9%)	4,265 (19.62%)	16,002 (22.59%)
Ins Medicare B	11,341 (23.1%)	4,023 (18.5%)	15,364 (21.69%)
Ins Medicare C	10,018 (20.4%)	3,580 (16.47%)	13,598 (19.19%)
Ins Medicare D	11,013 (22.43%)	3,805 (17.5%)	14,818 (20.92%)
Ins Other Coverage	11,010 (22.42%)	4,799 (22.07%)	15,809 (22.32%)
Ins Privatepay	7,726 (15.73%)	2,382 (10.96%)	10,108 (14.27%)
Ins Selffunded	1,138 (2.32%)	1,381 (6.35%)	2,519 (3.56%)
Ins Statesubsidized	945 (1.92%)	1,080 (4.97%)	2,025 (2.86%)
**Clinical data**			
A1c Age	45.10 (69.88)	53.98 (85.48)	47.83 (75.12)
Missing	27 (0.05%)	34 (0.16%)	61 (0.09%)
Acc Aha 201310yrcvdrisk	0.197 (0.155)	0.154 (0.138)	0.183 (0.151)
Missing	6,746 (13.738%)	2,493 (11.467%)	9,239 (13.041%)
Chf Dx Status			
0	43,536 (88.66%)	20,186 (92.85%)	63,722 (89.95%)
1	5,117 (10.42%)	1,391 (6.4%)	6,508 (9.19%)
999	450 (0.92%)	164 (0.75%)	614 (0.87%)
Cv Risk Subgrp			
Low	14,265 (29.05%)	8,694 (39.99%)	22,959 (32.41%)
Moderate	9,989 (20.34%)	4,656 (21.42%)	14,645 (20.67%)
High	15,942 (32.47%)	5,254 (24.17%)	21,196 (29.92%)
Other	2,339 (4.76%)	698 (3.21%)	3,037 (4.29%)
Unknown	6,568 (13.38%)	2,439 (11.22%)	9,007 (12.71%)
Diab Duration	8.09 (3.58)	7.19 (3.43)	7.82 (3.56)
Missing	1 (0%)	8 (0.04%)	9 (0.01%)
A1c	8.74 (1.58)	8.95 (1.77)	8.80 (1.65)
Missing	27 (0.05%)	34 (0.16%)	61 (0.09%)
Acr	73.11 (109.63)	58.03 (97.52)	68.78 (106.52)
Missing	6,036 (12.29%)	4,416 (20.31%)	10,452 (14.75%)
Afib Dx	3,632 (7.4%)	1,093 (5.03%)	4,725 (6.67%)
Alt	30.35 (21.38)	32.62 (21.70)	31.06 (21.51)
Missing	10,964 (22.33%)	4,320 (19.87%)	15,284 (21.57%)
Amputation Dxpx	605 (1.23%)	376 (1.73%)	981 (1.38%)
Anemia Dx	5,201 (10.59%)	2,327 (10.7%)	7,528 (10.63%)
Anxiety Dx	9,614 (19.58%)	5,972 (27.47%)	15,586 (22%)
Arrythmia Dx	3,264 (6.65%)	1,221 (5.62%)	4,485 (6.33%)
Ascvd Dxpx Max	7,925 (16.14%)	2,743 (12.62%)	10,668 (15.06%)
Ascvd Dxpx Ppv	2,339 (4.76%)	698 (3.21%)	3,037 (4.29%)
Asthma Dx	5,884 (11.98%)	3,234 (14.88%)	9,118 (12.87%)
Bariatric Px	500 (1.02%)	545 (2.51%)	1,045 (1.48%)

© 2025 Neugebauer R et al. *JAMA Network Open*.

	SGLT2i<br>n = 49,103	GLP-1RA<br>n = 21,741	Total<br>n = 70,844
Bipolar Dx	429 (0.87%)	416 (1.91%)	845 (1.19%)
Blind Dx	118 (0.24%)	59 (0.27%)	177 (0.25%)
Cad Dxpx Max	5,372 (10.94%)	1,690 (7.77%)	7,062 (9.97%)
Cad Dxpx Ppv	1,758 (3.58%)	501 (2.3%)	2,259 (3.19%)
Cad Dxpx Sens	1,891 (3.85%)	553 (2.54%)	2,444 (3.45%)
Cancer Mets Dx	0 (0%)	0 (0%)	0 (0%)
Cancer Nomets Dx	2,328 (4.74%)	1,418 (6.52%)	3,746 (5.29%)
Cevd Dxpx Ppv	528 (1.08%)	148 (0.68%)	676 (0.95%)
Cevd Dxpx Sens	2,479 (5.05%)	888 (4.08%)	3,367 (4.75%)
Chf Dx Ppv	1,252 (2.55%)	280 (1.29%)	1,532 (2.16%)
Chf Dx Sens	5,117 (10.42%)	1,391 (6.4%)	6,508 (9.19%)
Ckd Dx	17,447 (35.53%)	6,287 (28.92%)	23,734 (33.5%)
Copd Dx	2,492 (5.08%)	1,199 (5.51%)	3,691 (5.21%)
Coupled Dbp	72.69 (11.01)	74.11 (10.76)	73.12 (10.95)
Missing	944 (1.92%)	391 (1.8%)	1,335 (1.88%)
Coupled Sbp	130.13 (14.66)	130.03 (14.18)	130.10 (14.52)
Missing	944 (1.92%)	391 (1.8%)	1,335 (1.88%)
Covid Prd	36,676 (74.69%)	10,632 (48.9%)	47,308 (66.78%)
Creat	0.95 (0.30)	0.90 (0.29)	0.94 (0.30)
Missing	489 (1%)	467 (2.15%)	956 (1.35%)
Cysticfibrosis Dx	0 (0%)	0 (0%)	0 (0%)
Dbp	71.78 (11.11)	73.48 (10.83)	72.30 (11.06)
Missing	944 (1.92%)	391 (1.8%)	1,335 (1.88%)
Dementia Dx	0 (0%)	0 (0%)	0 (0%)
Depr Dx	6,351 (12.93%)	5,057 (23.26%)	11,408 (16.1%)
Dietitian	3,515 (7.16%)	3,041 (13.99%)	6,556 (9.25%)
Dka Dx	584 (1.19%)	496 (2.28%)	1,080 (1.52%)
Dka Dx Count	0.01 (0.13)	0.03 (0.23)	0.02 (0.17)
Esrd Dx	5,081 (10.35%)	1,701 (7.82%)	6,782 (9.57%)
Esrd Px	84 (0.17%)	62 (0.29%)	146 (0.21%)
Etoh Dx	848 (1.73%)	392 (1.8%)	1,240 (1.75%)
Fasciitis Dx	0 (0%)	0 (0%)	0 (0%)
Fpg	173.47 (64.47)	182.60 (73.67)	176.49 (67.78)
Missing	40,358 (82.19%)	17,427 (80.16%)	57,785 (81.57%)
Frailty Dx	4,471 (9.11%)	2,844 (13.08%)	7,315 (10.33%)
Gfr Epi 09	81.79 (22.00)	86.54 (23.22)	83.24 (22.49)
Missing	489 (1%)	467 (2.15%)	956 (1.35%)
Hdl	43.48 (11.29)	42.99 (11.27)	43.33 (11.29)
Missing	7,127 (14.51%)	2,808 (12.92%)	9,935 (14.02%)
Hgb	13.68 (1.59)	13.64 (1.53)	13.67 (1.57)
Missing	12,957 (26.39%)	5,586 (25.69%)	18,543 (26.17%)
Htn Dx	34,193 (69.64%)	14,846 (68.29%)	49,039 (69.22%)
Hypo Dx	128 (0.26%)	68 (0.31%)	196 (0.28%)
Hypo Dx Count	0.02 (0.20)	0.02 (0.18)	0.02 (0.19)
Hypo Dx Event	128 (0.26%)	68 (0.31%)	196 (0.28%)
Hypothyroidism Dx	5,881 (11.98%)	3,155 (14.51%)	9,036 (12.75%)
Ldl	82.06 (34.95)	88.26 (36.59)	84.00 (35.59)
Missing	6,254 (12.74%)	2,291 (10.54%)	8,545 (12.06%)
Leukemia Lymphoma Dx	293 (0.6%)	116 (0.53%)	409 (0.58%)
Lipid Dx	35,305 (71.9%)	15,121 (69.55%)	50,426 (71.18%)
Liver Dx	57 (0.12%)	62 (0.29%)	119 (0.17%)
Mci Dx	249 (0.51%)	121 (0.56%)	370 (0.52%)
Men2 Dx	0 (0%)	0 (0%)	0 (0%)
Nephropathy Dx	1,328 (2.7%)	973 (4.48%)	2,301 (3.25%)
Neuro Dx	1,440 (2.93%)	831 (3.82%)	2,271 (3.21%)
Pancreatitis Dx	0 (0%)	0 (0%)	0 (0%)
Pcr	0.000 (0.000)	0.000 (0.000)	0.000 (0.000)

© 2025 Neugebauer R et al. *JAMA Network Open*.

	SGLT2i n = 49,103	GLP-1RA n = 21,741	Total n = 70,844
Missing	42,575 (86.705%)	20,315 (93.441%)	62,890 (88.773%)
Potassium	4.29 (0.41)	4.26 (0.41)	4.28 (0.41)
Missing	1,690 (3.44%)	942 (4.33%)	2,632 (3.72%)
Pregnancy	0 (0%)	0 (0%)	0 (0%)
Pud Dx	55 (0.11%)	25 (0.11%)	80 (0.11%)
Pvd Dxpx Ppv	159 (0.32%)	85 (0.39%)	244 (0.34%)
Pvd Dxpx Sens	1,515 (3.09%)	677 (3.11%)	2,192 (3.09%)
Pyelo Dx	0 (0%)	0 (0%)	0 (0%)
Retinopathy Dxpx	2,036 (4.15%)	1,178 (5.42%)	3,214 (4.54%)
Rpg	197.65 (85.69)	203.29 (92.48)	199.65 (88.20)
Missing	28,052 (57.13%)	10,146 (46.67%)	38,198 (53.92%)
Sbp	129.27 (14.66)	129.42 (14.04)	129.31 (14.47)
Missing	944 (1.92%)	391 (1.8%)	1,335 (1.88%)
Schiz Dx	258 (0.53%)	160 (0.74%)	418 (0.59%)
Sodium	138.82 (2.89)	138.54 (2.88)	138.73 (2.89)
Missing	9,305 (18.95%)	2,638 (12.13%)	11,943 (16.86%)
Sud Dx	378 (0.77%)	300 (1.38%)	678 (0.96%)
Tc	159.18 (44.55)	164.66 (45.77)	160.89 (45.00)
Missing	7,099 (14.46%)	2,775 (12.76%)	9,874 (13.94%)
Trig	199.06 (155.12)	199.87 (163.03)	199.31 (157.60)
Missing	10,182 (20.74%)	4,400 (20.24%)	14,582 (20.58%)
Tsh	2.12 (1.87)	2.08 (1.93)	2.11 (1.89)
Missing	19,082 (38.86%)	7,859 (36.15%)	26,941 (38.03%)
Valvular Dx	1,658 (3.38%)	550 (2.53%)	2,208 (3.12%)
Vasculitis Dx	408 (0.83%)	252 (1.16%)	660 (0.93%)
Only Met No Ascvd	6,136 (12.5%)	2,630 (12.1%)	8,766 (12.37%)
Renal Function Status			
Lowrisk	22,777 (46.39%)	10,148 (46.68%)	32,925 (46.48%)
Moderaterisk	12,019 (24.48%)	4,755 (21.87%)	16,774 (23.68%)
Highrisk	5,670 (11.55%)	1,682 (7.74%)	7,352 (10.38%)
Veryhighrisk	2,519 (5.13%)	652 (3%)	3,171 (4.48%)
Unknown	6,118 (12.46%)	4,504 (20.72%)	10,622 (14.99%)
Total Visit C	1.00 (3.06)	0.96 (3.09)	0.99 (3.07)
Total Visit E	0.45 (2.13)	1.03 (3.26)	0.63 (2.54)
Total Visit N	0.28 (1.58)	0.25 (1.74)	0.27 (1.63)
**Concurrent medications**			
Aa	1 (0%)	7 (0.03%)	8 (0.01%)
Aceinhibitors	21,372 (43.52%)	9,013 (41.46%)	30,385 (42.89%)
Agi	506 (1.03%)	180 (0.83%)	686 (0.97%)
Anticoagulants	3,369 (6.86%)	1,145 (5.27%)	4,514 (6.37%)
Anticonvulsants	5,348 (10.89%)	3,463 (15.93%)	8,811 (12.44%)
Antidepressantcomb	1 (0%)	1 (0%)	2 (0%)
Antidepressantmaoi	7 (0.01%)	3 (0.01%)	10 (0.01%)
Antidepressantndri	1,090 (2.22%)	966 (4.44%)	2,056 (2.9%)
Antidepressantother	0 (0%)	0 (0%)	0 (0%)
Antidepressantsari	1,540 (3.14%)	1,011 (4.65%)	2,551 (3.6%)
Antidepressantsnri	2,196 (4.47%)	1,703 (7.83%)	3,899 (5.5%)
Antidepressantspo	40 (0.08%)	27 (0.12%)	67 (0.09%)
Antidepressantssri	4,691 (9.55%)	3,339 (15.36%)	8,030 (11.33%)
Antidepressanttca	1,261 (2.57%)	910 (4.19%)	2,171 (3.06%)
Antidepressantteca	339 (0.69%)	208 (0.96%)	547 (0.77%)
Antiplatelets	6,257 (12.74%)	2,210 (10.17%)	8,467 (11.95%)
Antipsychotic1stgen	91 (0.19%)	54 (0.25%)	145 (0.2%)
Antipsychotic2ndgen	734 (1.49%)	631 (2.9%)	1,365 (1.93%)
Anxiety	774 (1.58%)	621 (2.86%)	1,395 (1.97%)
Arb	14,190 (28.9%)	5,620 (25.85%)	19,810 (27.96%)
Benzodiazepines	1,513 (3.08%)	1,196 (5.5%)	2,709 (3.82%)

© 2025 Neugebauer R et al. *JAMA Network Open*.

	SGLT2i n = 49,103	GLP-1RA n = 21,741	Total n = 70,844
Betablockers	17,425 (35.49%)	6,650 (30.59%)	24,075 (33.98%)
Clonidine	537 (1.09%)	265 (1.22%)	802 (1.13%)
Dihydropyridineccb	9,997 (20.36%)	3,784 (17.4%)	13,781 (19.45%)
Dpp4	4,263 (8.68%)	2,290 (10.53%)	6,553 (9.25%)
Hypnoticother	369 (0.75%)	338 (1.55%)	707 (1%)
Injectableantipsychotic	3 (0.01%)	9 (0.04%)	12 (0.02%)
Ins	15,135 (30.82%)	11,478 (52.79%)	26,613 (37.57%)
Ins Analog	3,829 (7.8%)	4,758 (21.88%)	8,587 (12.12%)
Ins Combo	2,168 (4.42%)	1,337 (6.15%)	3,505 (4.95%)
Ins Human	12,448 (25.35%)	7,592 (34.92%)	20,040 (28.29%)
Ins La	12,418 (25.29%)	9,445 (43.44%)	21,863 (30.86%)
Ins Sa	5,562 (11.33%)	5,197 (23.9%)	10,759 (15.19%)
Ksparingdiuretics	2,800 (5.7%)	1,122 (5.16%)	3,922 (5.54%)
Lithium	54 (0.11%)	55 (0.25%)	109 (0.15%)
Loopdiuretics	5,300 (10.79%)	2,168 (9.97%)	7,468 (10.54%)
Meg	204 (0.42%)	226 (1.04%)	430 (0.61%)
Met	39,258 (79.95%)	16,277 (74.87%)	55,535 (78.39%)
Nondihydropyridineccb	1,034 (2.11%)	492 (2.26%)	1,526 (2.15%)
Otherlipidmeds	2,110 (4.3%)	1,042 (4.79%)	3,152 (4.45%)
Pcsk9mab	56 (0.11%)	26 (0.12%)	82 (0.12%)
Statins	39,628 (80.7%)	15,779 (72.58%)	55,407 (78.21%)
Stimulants	217 (0.44%)	210 (0.97%)	427 (0.6%)
Sunew	29,368 (59.81%)	9,460 (43.51%)	38,828 (54.81%)
Suold	0 (0%)	2 (0.01%)	2 (0%)
Thiazidediuretics	12,588 (25.64%)	5,957 (27.4%)	18,545 (26.18%)
Tir	0 (0%)	0 (0%)	0 (0%)
Tzd	3,355 (6.83%)	1,120 (5.15%)	4,475 (6.32%)
Only Met Therapy	7,271 (14.81%)	2,887 (13.28%)	10,158 (14.34%)

eFigure 35. MACE (Primary Definition), 2-Arm Drug Class Comparison, SGLT2is vs GLP-1RAs, Cumulative Incidence Curves From PP and ITT Analyses With IPW, TMLE, and SL
Each plot emulates inferences from a 2-arm RCT comparing SGLT2i and GLP-1RA and represents unadjusted or adjusted estimates of cumulative incidence curves for MACE derived with inverse probability weighting (IPW) and Targeted Minimum Loss-based Estimation (TMLE) with Super Learning (SL) estimates of propensity scores with four weight truncation schemes: IPW and TMLE without weight truncation (untruncated), IPW with

© 2025 Neugebauer R et al. *JAMA Network Open*.

truncation of stabilized weights at value 20 (trunc20) or at the 99th percentile of weight values (trunc99), and TMLE with truncation of unstabilized weights at value 200 (trunc200). The red divi der line separates results of Per-Protocol (PP) analyses (top half) from Intention-To-Treat (ITT) analyses (bottom half). Each plot displays a p value for the test that the average risk difference (ARD) through 2.5 years of follow-up (30 months) is 0.

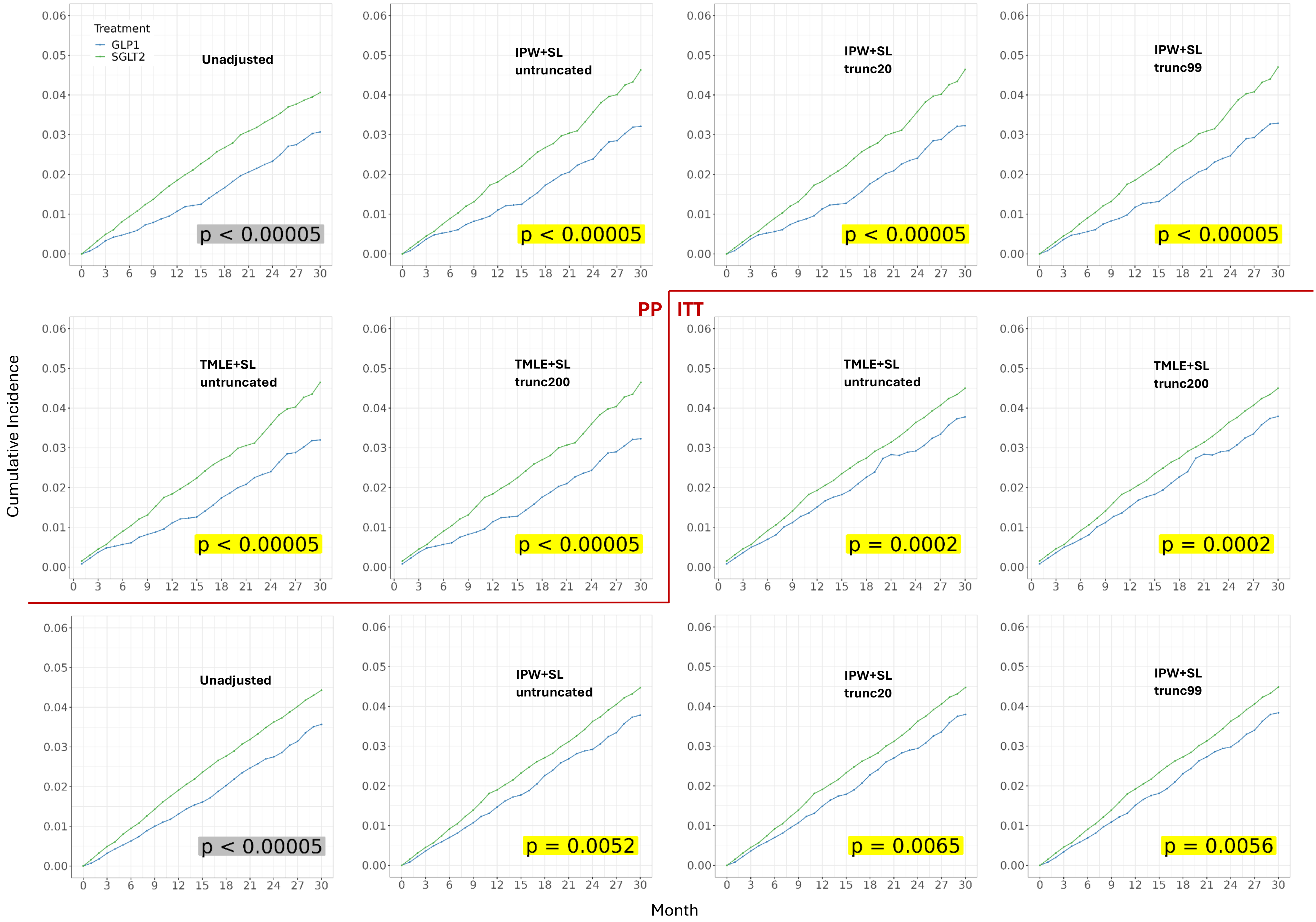

© 2025 Neugebauer R et al. *JAMA Network Open*.

eFigure 36. MACE (Primary Definition), 2-Arm Drug Class Comparison, SGLT2is vs GLP-1RAs, Cumulative Incidence Curves From Sensitivity PP Analyses With IPW, TMLE, and SL
Each plot emulates inferences from a 2-arm RCT comparing SGLT2i and GLP-1RA and represents unadjusted or adjusted estimates of cumulative incidence curves for MACE from sensitivity PP analyses referred to as "NoMBS PP" and "No3 PP". NoMBS PP analyses are restricted to patients without a history of MBS at baseline and the protocols they evaluate preclude metabolic bariatric surgery (MBS) procedures. The protocols in the No3 PP analyses preclude exposure to three medication classes: the comparator medication from the other arm, SU and DPP4i.

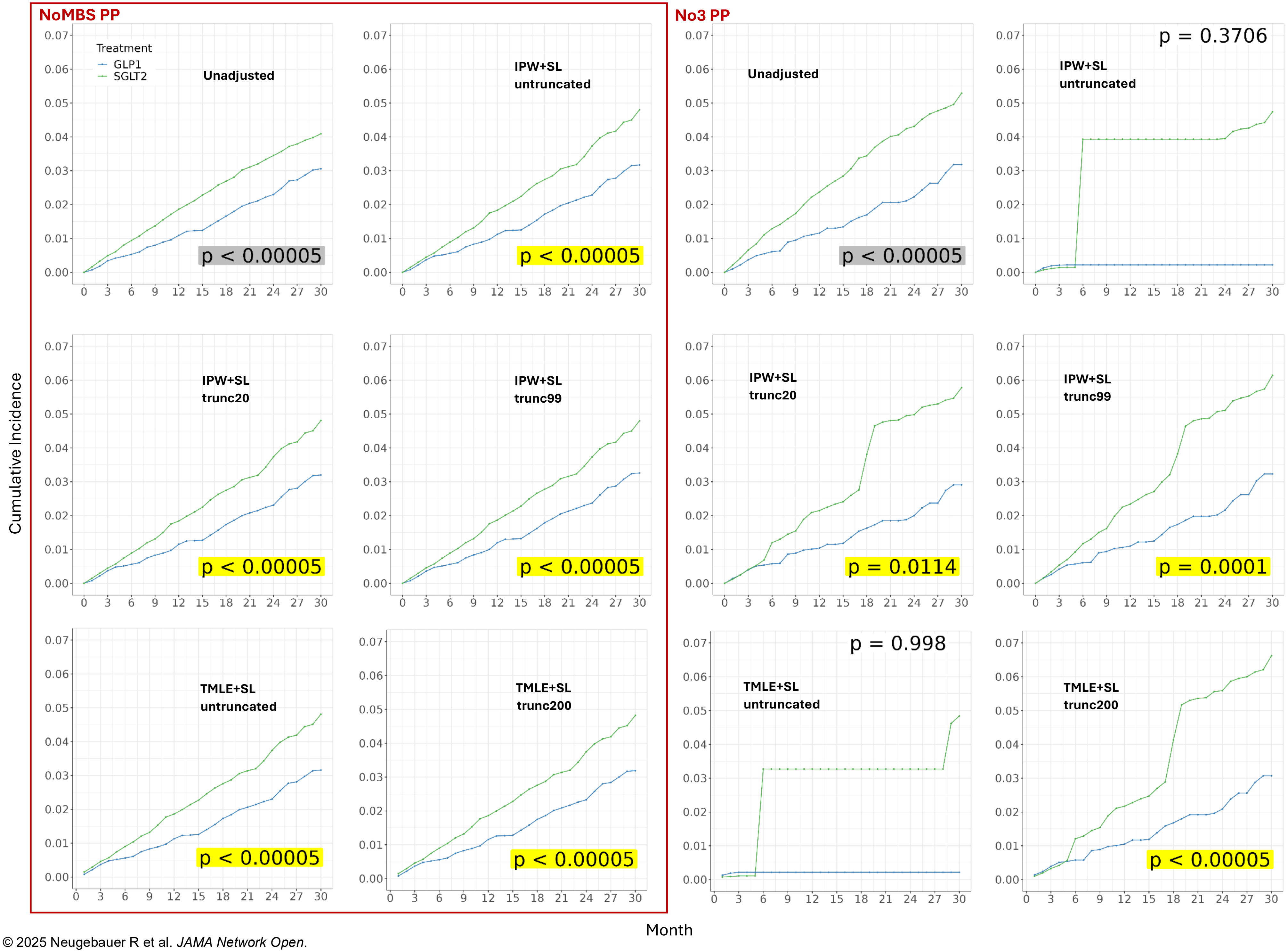

© 2025 Neugebauer R et al. *JAMA Network Open*.

eTable 35. MACE (Primary Definition), 2-Arm Drug Class Comparison, SGLT2is vs GLP-1RAs, RD and HR Effect Measures at 2.5 Years

Estimation results from ITT, PP, NoMBS PP, and No3 PP analyses of emulated 2-arm RCTs comparing MACE risks over 2.5 years between SGLT2i and GLP-1RA initiators. For PP analyses, rates of protocol deviations are described by medication class initiated at baseline. Unadjusted point and interval estimates and adjusted point and interval IPW and TMLE estimates of risks, risk differences (RD), and hazard ratios (HR) based on propensity scores (PS) estimated with either logistic models or super learning (SL) are presented for four weight truncation schemes along with the corresponding 99th percentile and maximum value of the stabilized and unstabilized inverse probability weights used for implementing IPW and TMLE, respectively. RD is the risk in treatment arm minus the risk in control arm and NNT is the number needed to treat.

Analysis type	Protocol Deviations* by exposure group (%)	PS estimation	99th IP weights	Max IP weight	Estimator	Treatment (SGLT2i) risk in %	Control (GLP-1RA) risk in %	RD [95% CI] in %	NNT	HR [95% CI]
PP	Discontinuation<br>SGLT2i: 25.58<br>GLP-1RA: 44.65<br><br>Crossover<br>SGLT2i: 5.01<br>GLP-1RA: 9.80	SL			Unadjusted	4.06	3.07	1.00 [0.39, 1.60]	100	1.74 [1.43, 2.05]
			16.85	1,129.30	TMLE untruncated	4.65	3.20	1.45 [1.05, 1.85]	69	
					TMLE truncated at 200	4.65	3.23	1.43 [1.03, 1.83]	70	
			3.22	181.05	IPW untruncated	4.63	3.21	1.42 [0.52, 2.32]	70	1.61 [1.23, 2.00]
					IPW truncated at 20	4.64	3.23	1.40 [0.50, 2.30]	71	1.61 [1.22, 1.99]
					IPW truncated at 99th	4.70	3.29	1.41 [0.50, 2.32]	71	1.61 [1.25, 1.97]
		Logistic model	4.72	658.60	IPW untruncated	5.22	3.58	1.64 [0.04, 3.25]	61	1.57 [1.17, 1.98]
					IPW truncated at 20	5.35	3.67	1.67 [0.03, 3.32]	60	1.56 [1.15, 1.96]
					IPW truncated at 99th	5.36	3.79	1.57 [0.12, 3.03]	64	1.54 [1.16, 1.91]
ITT		SL			Unadjusted	4.43	3.57	0.86 [0.41, 1.31]	116	1.45 [1.24, 1.65]
			10.66	721.62	TMLE untruncated	4.50	3.78	0.72 [0.39, 1.05]	139	
					TMLE truncated at 200	4.50	3.79	0.71 [0.37, 1.04]	141	
			3.00	148.26	IPW untruncated	4.47	3.78	0.70 [0.10, 1.29]	144	1.29 [1.04, 1.54]
					IPW truncated at 20	4.48	3.80	0.68 [0.09, 1.28]	147	1.28 [1.03, 1.53]
					IPW truncated at 99th	4.49	3.84	0.64 [0.07, 1.22]	155	1.28 [1.05, 1.51]
		Logistic model	4.27	682.81	IPW untruncated	4.54	3.96	0.58 [-0.24, 1.39]		1.23 [0.90, 1.57]
					IPW truncated at 20	4.53	4.01	0.52 [-0.26, 1.31]		1.22 [0.92, 1.52]
					IPW truncated at 99th	4.45	4.03	0.42 [-0.25, 1.09]		1.22 [0.99, 1.46]
NoMBS PP	Discontinuation<br>SGLT2i: 25.39<br>GLP-1RA: 43.97<br><br>Crossover<br>SGLT2i: 5.22<br>GLP-1RA: 10.83<br><br>MBS occurrence<br>SGLT2i: 0.25<br>GLP-1RA: 0.95	SL			Unadjusted	4.09	3.06	1.03 [0.42, 1.65]	97	1.73 [1.42, 2.03]
			17.00	1,244.96	TMLE untruncated	4.81	3.16	1.65 [1.23, 2.07]	61	
					TMLE truncated at 200	4.82	3.19	1.63 [1.20, 2.05]	61	
			3.19	214.37	IPW untruncated	4.80	3.17	1.63 [0.66, 2.59]	61	1.61 [1.23, 2.00]
					IPW truncated at 20	4.81	3.20	1.61 [0.64, 2.58]	62	1.61 [1.22, 1.99]
					IPW truncated at 99th	4.80	3.26	1.54 [0.61, 2.48]	65	1.60 [1.24, 1.96]
		Logistic model	4.69	671.59	IPW untruncated	5.36	3.45	1.91 [0.26, 3.57]	52	1.55 [1.15, 1.96]
					IPW truncated at 20	5.47	3.54	1.93 [0.24, 3.62]	52	1.54 [1.14, 1.94]
					IPW truncated at 99th	5.48	3.66	1.83 [0.34, 3.32]	55	1.51 [1.15, 1.88]
No3 PP	Discontinuation<br>SGLT2i: 8.27<br>GLP-1RA: 21.77<br><br>Crossover to comparator drug<br>SGLT2i: 1.93<br>GLP-1RA: 4.11<br><br>Initiation of one of the two non-comparator drugs<br>SGLT2i: 66.28<br>GLP-1RA: 50.50	SL			Unadjusted	5.29	3.18	2.10 [1.00, 3.20]	48	1.61 [1.07, 2.16]
			18.21	3.58e+17	TMLE untruncated	4.84	0.22	4.62 [-2552.47, 2561.71]		
					TMLE truncated at 200	6.62	3.07	3.55 [2.66, 4.43]	28	
			2.57	8.28e+15	IPW untruncated	4.74	0.22	4.52 [-3.90, 12.94]		0.96 [0.14, 1.79]
					IPW truncated at 20	5.78	2.91	2.87 [0.09, 5.65]	35	0.99 [0.60, 1.38]
					IPW truncated at 99th	6.14	3.23	2.91 [0.85, 4.98]	34	1.18 [0.72, 1.65]
		Logistic model	3.49	1.51e+21	IPW untruncated	16.86	0.00	16.86 [-16.26, 49.98]		16088.49 [-20118.12, 52295.11]
					IPW truncated at 20	5.40	2.90	2.49 [0.18, 4.81]	40	0.87 [0.51, 1.24]
					IPW truncated at 99th	6.06	3.18	2.88 [0.79, 4.98]	35	1.03 [0.60, 1.46]

* Discontinuation refers to the interruption of the comparator medication initiated on index date; Crossover refers to the initiation of the comparator medication initiated by patient at baseline in the other arm; MBS occurrence refers to patient's undergoing metabolic bariatric surgery (MBS).

© 2025 Neugebauer R et al. *JAMA Network Open*.

eFigure 37. MACE (Primary Definition), 2-Arm Drug Class Comparison, SGLT2is vs GLP-1RAs, ASCVD Subgroup, Cumulative Incidence Curves From PP and ITT Analyses With IPW, TMLE, and SL
Each plot emulates inferences among patients with ASCVD from a 2-arm RCT comparing SGLT2i and GLP-1RA and represents unadjusted or adjusted estimates of cumulative incidence curves for MACE derived with IPW and TMLE with SL estimates of propensity scores with four weight truncation schemes: IPW and TMLE without weight truncation (untruncated), IPW with truncation of stabilized weights at value 20 (trunc20) or at the 99th percentile of weight values (trunc99), and TMLE with truncation of unstabilized weights at value 200 (trunc200). The red divider line separates results of Per-Protocol (PP) analyses (top half) from Intention-To-Treat (ITT) analyses (bottom half). Each plot displays a p value for the test that the average risk difference (ARD) through 2.5 years of follow-up (30 months) is 0.

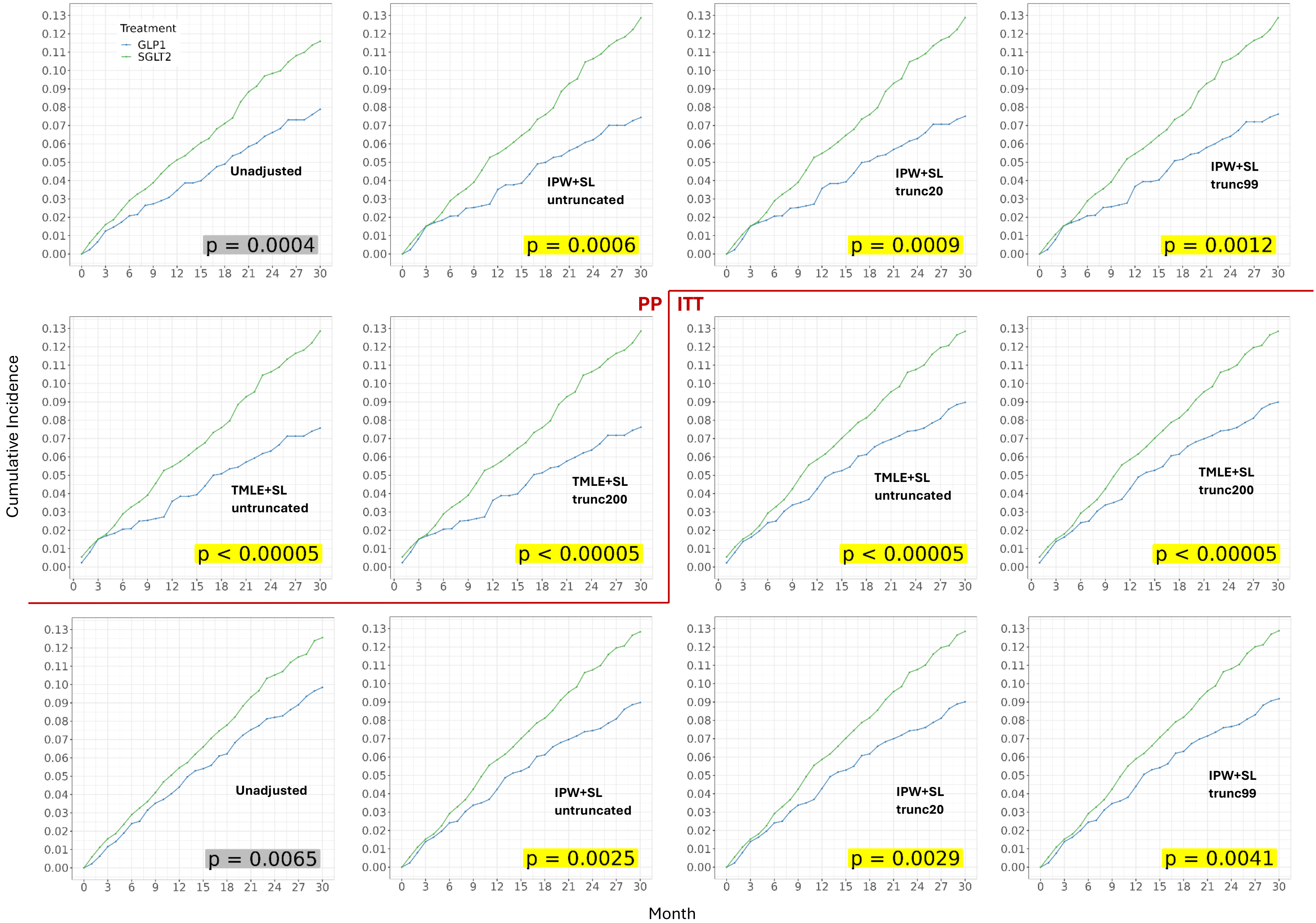

© 2025 Neugebauer R et al. *JAMA Network Open*.

eTable 36. MACE (Primary Definition), 2-Arm Drug Class Comparison, SGLT2is vs GLP-1RAs, ASCVD Subgroup, RD and HR Effect Measures at 2.5 Years

Estimation results among patients with ASCVD from ITT and PP analyses of emulated 2-arm RCTs comparing MACE risks over 2.5 years between SGLT2i and GLP-1RA initiators. For PP analyses, rates of protocol deviations are described by medication class initiated at baseline. Unadjusted point and interval estimates and adjusted point and interval IPW and TMLE estimates of risks, risk differences (RD), and hazard ratios (HR) based on propensity scores (PS) estimated with either logistic models or super learning (SL) are presented for four weight truncation schemes along with the corresponding 99th percentile and maximum value of the stabilized and unstabilized inverse probability weights used for implementing IPW and TMLE, respectively. RD is the risk in treatment arm minus the risk in control arm and NNT is the number needed to treat.

Analysis type	Protocol Deviations* by exposure group (%)	PS estimation	99th IP weights	Max IP weight	Estimator	Treatment (SGLT2i) risk in %	Control (GLP-1RA) risk in %	RD [95% CI] in %	NNT	HR [95% CI]
PP	Discontinuation<br>SGLT2i: 23.44<br>GLP-1RA: 45.10<br><br>Crossover<br>SGLT2i: 5.02<br>GLP-1RA: 11.81	SL			Unadjusted	11.59	7.89	3.70 [1.14, 6.26]	27	1.43 [1.04, 1.81]
			15.57	476.97	TMLE untruncated	12.86	7.57	5.30 [3.88, 6.71]	19	
					TMLE truncated at 200	12.86	7.62	5.25 [3.83, 6.66]	19	
			2.68	45.20	IPW untruncated	12.87	7.44	5.44 [2.20, 8.67]	18	1.47 [0.93, 2.02]
					IPW truncated at 20	12.88	7.50	5.38 [2.13, 8.64]	19	1.47 [0.93, 2.02]
					IPW truncated at 99th	12.87	7.62	5.25 [1.99, 8.50]	19	1.45 [0.94, 1.97]
		Logistic model	4.13	87.91	IPW untruncated	15.18	7.71	7.48 [2.69, 12.27]	13	1.56 [0.73, 2.38]
					IPW truncated at 20	14.82	7.81	7.02 [2.45, 11.59]	14	1.46 [0.74, 2.19]
					IPW truncated at 99th	14.13	7.80	6.33 [2.03, 10.64]	16	1.41 [0.84, 1.98]
ITT		SL			Unadjusted	12.56	9.85	2.71 [0.78, 4.65]	37	1.19 [0.93, 1.45]
			10.61	386.69	TMLE untruncated	12.84	8.97	3.88 [2.70, 5.05]	26	
					TMLE truncated at 200	12.84	8.99	3.85 [2.68, 5.02]	26	
			2.56	66.07	IPW untruncated	12.83	8.97	3.87 [1.68, 6.05]	26	1.30 [0.92, 1.67]
					IPW truncated at 20	12.85	9.01	3.84 [1.64, 6.04]	26	1.30 [0.92, 1.67]
					IPW truncated at 99th	12.89	9.18	3.71 [1.52, 5.90]	27	1.27 [0.92, 1.62]
		Logistic model	4.00	116.58	IPW untruncated	14.06	8.94	5.12 [2.13, 8.11]	20	1.42 [0.82, 2.02]
					IPW truncated at 20	13.89	9.03	4.87 [2.01, 7.72]	21	1.34 [0.81, 1.87]
					IPW truncated at 99th	13.17	9.15	4.02 [1.54, 6.50]	25	1.22 [0.83, 1.61]

* Discontinuation refers to the interruption of the comparator medication initiated on index date; Crossover refers to the initiation of the comparator medication initiated by patient at baseline in the other arm.

eFigure 38. MACE (Primary Definition), 2-Arm Drug Class Comparison, SGLT2is vs GLP-1RAs, No ASCVD Subgroup, Cumulative Incidence Curves From PP and ITT Analyses With IPW, TMLE, and SL
Each plot emulates inferences among patients with No ASCVD from a 2-arm RCT comparing SGLT2i and GLP-1RA and represents unadjusted or adjusted estimates of cumulative incidence curves for MACE derived with IPW and TMLE with SL estimates of propensity scores with four weight truncation schemes: IPW and TMLE without weight truncation (untruncated), IPW with truncation of stabilized weights at value 20 (trunc20) or at the 99th percentile of weight values (trunc99), and TMLE with truncation of unstabilized weights at value 200 (trunc200). The red divider line separates results of Per-Protocol (PP) analyses (top half) from Intention-To-Treat (ITT) analyses (bottom half). Each plot displays a p value for the test that the average risk difference (ARD) through 2.5 years of follow-up (30 months) is 0.

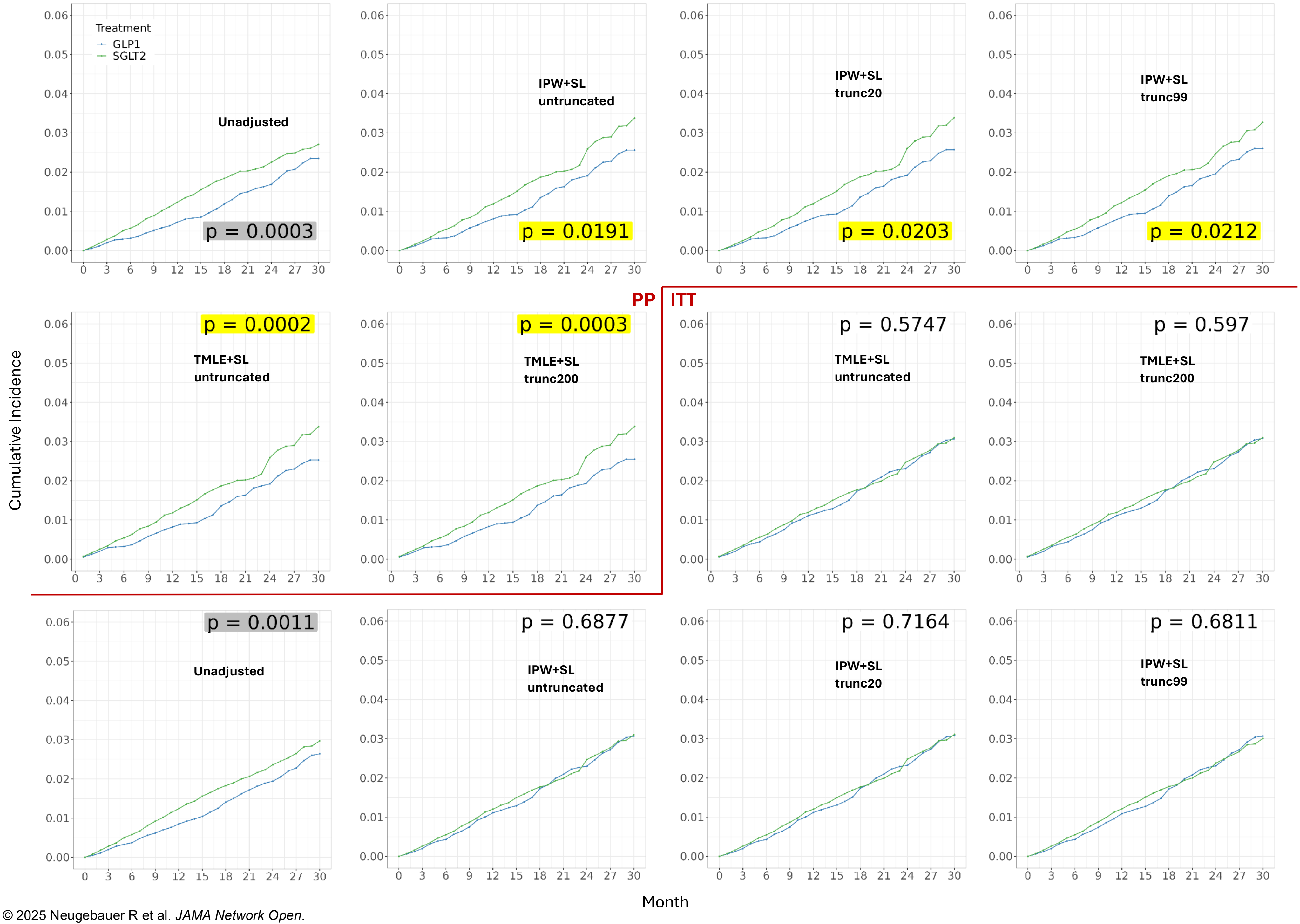

© 2025 Neugebauer R et al. *JAMA Network Open*.

eTable 37. MACE (Primary Definition), 2-Arm Drug Class Comparison, SGLT2is vs GLP-1RAs, No ASCVD Subgroup, RD and HR Effect Measures at 2.5 Years

Estimation results among patients with No ASCVD from ITT and PP analyses of emulated 2-arm RCTs comparing MACE risks over 2.5 years between SGLT2i and GLP-1RA initiators. For PP analyses, rates of protocol deviations are described by medication class initiated at baseline. Unadjusted point and interval estimates and adjusted point and interval IPW and TMLE estimates of risks, risk differences (RD), and hazard ratios (HR) based on propensity scores (PS) estimated with either logistic models or super learning (SL) are presented for four weight truncation schemes along with the corresponding 99th percentile and maximum value of the stabilized and unstabilized inverse probability weights used for implementing IPW and TMLE, respectively. RD is the risk in treatment arm minus the risk in control arm and NNT is the number needed to treat.

Analysis type	Protocol Deviations* by exposure group (%)	PS estimation	99th IP weights	Max IP weight	Estimator	Treatment (SGLT2i) risk in %	Control (GLP-1RA) risk in %	RD [95% CI] in %	NNT	HR [95% CI]
PP	<u>Discontinuation</u><br>SGLT2i: 25.99<br>GLP-1RA: 44.59<br><br><u>Crossover</u><br>SGLT2i: 5.01<br>GLP-1RA: 9.52	SL			Unadjusted	2.71	2.35	0.36 [-0.20, 0.93]		1.72 [1.32, 2.13]
			16.41	1,322.37	TMLE untruncated	3.38	2.53	0.85 [0.36, 1.33]	118	
					TMLE truncated at 200	3.39	2.55	0.84 [0.35, 1.32]	119	
			3.18	155.37	IPW untruncated	3.38	2.56	0.82 [-0.23, 1.87]		1.51 [1.05, 1.97]
					IPW truncated at 20	3.39	2.57	0.82 [-0.23, 1.87]		1.51 [1.05, 1.96]
					IPW truncated at 99th	3.27	2.60	0.66 [-0.22, 1.54]		1.51 [1.07, 1.95]
		Logistic model	4.68	5,092.97	IPW untruncated	3.42	2.61	0.80 [-0.43, 2.03]		1.53 [1.03, 2.02]
					IPW truncated at 20	3.65	2.67	0.98 [-0.30, 2.25]		1.51 [1.02, 2.00]
					IPW truncated at 99th	3.75	2.77	0.98 [-0.25, 2.22]		1.48 [1.01, 1.95]
ITT		SL			Unadjusted	2.97	2.64	0.33 [-0.09, 0.74]		1.47 [1.19, 1.75]
			10.27	838.98	TMLE untruncated	3.10	3.07	0.03 [-0.34, 0.41]		
					TMLE truncated at 200	3.10	3.08	0.03 [-0.35, 0.40]		
			2.95	177.15	IPW untruncated	3.10	3.07	0.03 [-0.65, 0.72]		1.13 [0.84, 1.43]
					IPW truncated at 20	3.11	3.08	0.02 [-0.66, 0.71]		1.13 [0.83, 1.43]
					IPW truncated at 99th	3.01	3.07	-0.06 [-0.62, 0.50]		1.17 [0.89, 1.45]
		Logistic model	4.26	726.78	IPW untruncated	3.08	3.19	-0.11 [-0.99, 0.78]		1.04 [0.64, 1.44]
					IPW truncated at 20	3.10	3.19	-0.09 [-0.93, 0.76]		1.07 [0.71, 1.43]
					IPW truncated at 99th	3.03	3.17	-0.14 [-0.81, 0.54]		1.15 [0.86, 1.45]

* Discontinuation refers to the interruption of the comparator medication initiated on index date; Crossover refers to the initiation of the comparator medication initiated by patient at baseline in the other arm.

© 2025 Neugebauer R et al. *JAMA Network Open*.

eFigure 39. MACE (Primary Definition), 2-Arm Drug Class Comparison, SGLT2is vs GLP-1RAs, No ASCVD and MET Subgroup, Cumulative Incidence Curves From PP and ITT Analyses With IPW, TMLE, and SL
Each plot emulates inferences among patients with No ASCVD and MET from a 2-arm RCT comparing SGLT2i and GLP-1RA and represents unadjusted or adjusted estimates of cumulative incidence curves for MACE derived with IPW and TMLE with SL estimates of propensity scores with four weight truncation schemes: IPW and TMLE without weight truncation (untruncated), IPW with truncation of stabilized weights at value 20 (trunc20) or at the 99th percentile of weight values (trunc99), and TMLE with truncation of unstabilized weights at value 200 (trunc200). The red divi der line separates results of Per-Protocol (PP) analyses (top half) from Intention-To-Treat (ITT) analyses (bottom half). Each plot displays a p value for the test that the average risk difference (ARD) through 2.5 years of follow-up (30 months) is 0.

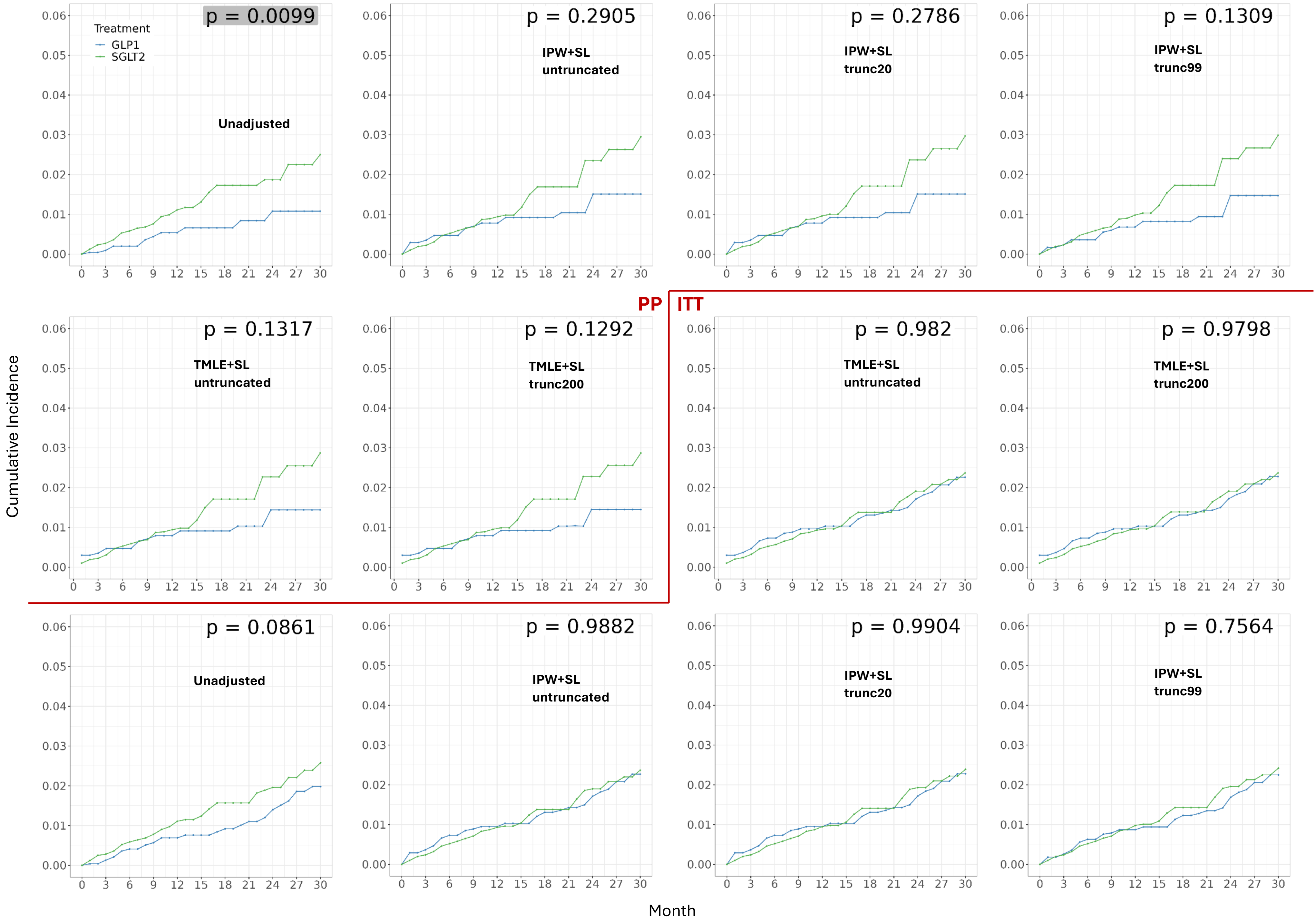

© 2025 Neugebauer R et al. *JAMA Network Open*.

eTable 38. MACE (Primary Definition), 2-Arm Drug Class Comparison, SGLT2is vs GLP-1RAs, No ASCVD and MET Subgroup, RD and HR Effect Measures at 2.5 Years
Estimation results among patients with No ASCVD and MET from ITT and PP analyses of emulated 2-arm RCTs comparing MACE risks over 2.5 years between SGLT2i and GLP-1RA initiators. For PP analyses, rates of protocol deviations are described by medication class initiated at baseline. Unadjusted point and interval estimates and adjusted point and interval IPW and TMLE estimates of risks, risk differences (RD), and hazard ratios (HR) based on propensity scores (PS) estimated with either logistic models or super learning (SL) are presented for four weight truncation schemes along with the corresponding 99th percentile and maximum value of the stabilized and unstabilized inverse probability weights used for implementing IPW and TMLE, respectively. RD is the risk in treatment arm minus the risk in control arm and NNT is the number needed to treat.

Analysis type	Protocol Deviations* by exposure group (%)	PS estimation	99th IP weights	Max IP weight	Estimator	Treatment (SGLT2i) risk in %	Control (GLP-1RA) risk in %	RD [95% CI] in %	NNT	HR [95% CI]
PP	Discontinuation<br>SGLT2i: 27.20<br>GLP-1RA: 41.06<br><br>Crossover<br>SGLT2i: 4.63<br>GLP-1RA: 6.88	SL	[redacted]	[redacted]	Unadjusted	2.50	1.08	1.42 [0.22, 2.62]	70	2.01 [0.56, 3.46]
			17.01	1,730.83	TMLE untruncated	2.87	1.44	1.42 [0.60, 2.25]	70	[redacted]
					TMLE truncated at 200	2.87	1.45	1.43 [0.60, 2.26]	70	[redacted]
			3.21	177.60	IPW untruncated	2.95	1.51	1.45 [-0.57, 3.46]		1.00 [-0.13, 2.14]
					IPW truncated at 20	2.97	1.51	1.47 [-0.55, 3.48]		1.00 [-0.13, 2.13]
					IPW truncated at 99th	2.99	1.47	1.52 [-0.50, 3.55]		1.25 [0.08, 2.43]
		Logistic model	4.34	48.30	IPW untruncated	2.83	1.40	1.43 [-0.45, 3.32]		0.77 [-0.17, 1.70]
					IPW truncated at 20	2.83	1.40	1.43 [-0.45, 3.32]		0.76 [-0.16, 1.69]
					IPW truncated at 99th	2.86	1.29	1.58 [-0.23, 3.38]		1.00 [0.00, 2.01]
ITT	[redacted]	SL	[redacted]	[redacted]	Unadjusted	2.58	1.98	0.60 [-0.45, 1.66]		1.53 [0.64, 2.41]
			11.89	1,076.36	TMLE untruncated	2.37	2.26	0.11 [-0.64, 0.85]		[redacted]
					TMLE truncated at 200	2.37	2.28	0.10 [-0.65, 0.84]		[redacted]
			3.17	188.47	IPW untruncated	2.37	2.27	0.10 [-1.19, 1.39]		0.87 [0.11, 1.62]
					IPW truncated at 20	2.39	2.28	0.11 [-1.19, 1.41]		0.87 [0.11, 1.62]
					IPW truncated at 99th	2.42	2.25	0.17 [-1.09, 1.42]		0.99 [0.26, 1.72]
		Logistic model	4.26	52.72	IPW untruncated	2.33	2.30	0.02 [-1.37, 1.42]		0.73 [0.01, 1.46]
					IPW truncated at 20	2.33	2.31	0.02 [-1.37, 1.42]		0.73 [0.01, 1.45]
					IPW truncated at 99th	2.37	2.20	0.17 [-1.11, 1.45]		0.89 [0.18, 1.60]

* Discontinuation refers to the interruption of the comparator medication initiated on index date; Crossover refers to the initiation of the comparator medication initiated by patient at baseline in the other arm.

© 2025 Neugebauer R et al. *JAMA Network Open*.

eFigure 40. MACE (Primary Definition), 2-Arm Drug Class Comparison, SGLT2is vs GLP-1RAs, HF Subgroup, Cumulative Incidence Curves From PP and ITT Analyses With IPW, TMLE, and SL
Each plot emulates inferences among patients with HF from a 2-arm RCT comparing SGLT2i and GLP-1RA and represents unadjusted or adjusted estimates of cumulative incidence curves for MACE derived with IPW and TMLE with SL estimates of propensity scores with four weight truncation schemes: IPW and TMLE without weight truncation (untruncated), IPW with truncation of stabilized weights at value 20 (trunc20) or at the 99th percentile of weight values (trunc99), and TMLE with truncation of unstabilized weights at value 200 (trunc200). The red divider line separates results of Per-Protocol (PP) analyses (top half) from Intention-To-Treat (ITT) analyses (bottom half). Each plot displays a p value for the test that the average risk difference (ARD) through 2.5 years of follow-up (30 months) is 0.

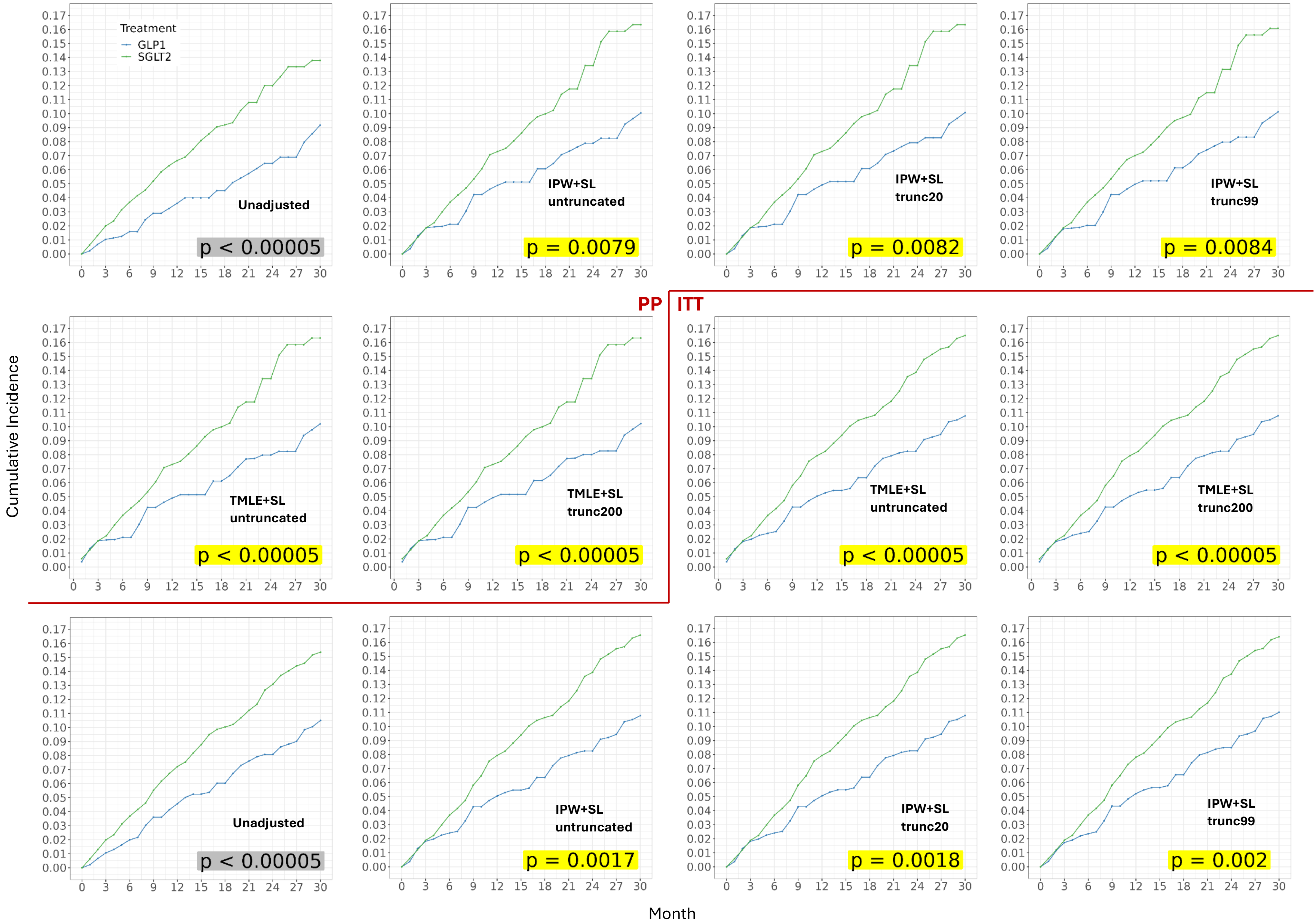

© 2025 Neugebauer R et al. *JAMA Network Open*.

eTable 39. MACE (Primary Definition), 2-Arm Drug Class Comparison, SGLT2is vs GLP-1RAs, HF Subgroup, RD and HR Effect Measures at 2.5 Years

Estimation results among patients with HF from ITT and PP analyses of emulated 2-arm RCTs comparing MACE risks over 2.5 years between SGLT2i and GLP-1RA initiators. For PP analyses, rates of protocol deviations are described by medication class initiated at baseline. Unadjusted point and interval estimates and adjusted point and interval IPW and TM LE estimates of risks, risk differences (RD), and hazard ratios (HR) based on propensity scores (PS) estimated with either logistic models or super learning (SL) are presented for four weight truncation schemes along with the corresponding 99th percentile and maximum value of the stabilized and unstabilized inverse probability weights used for implementing IPW and TMLE, respectively. RD is the risk in treatment arm minus the risk in control arm and NNT is the number needed to treat.

Analysis type	Protocol Deviations* by exposure group (%)	PS estimation	99th IP weights	Max IP weight	Estimator	Treatment (SGLT2i) risk in %	Control (GLP-1RA) risk in %	RD [95% CI] in %	NNT	HR [95% CI]
PP	Discontinuation<br>SGLT2i: 19.50<br>GLP-1RA: 42.27<br><br>Crossover<br>SGLT2i: 3.85<br>GLP-1RA: 11.29	SL			Unadjusted	13.79	9.18	4.61 [0.57, 8.65]	22	1.98 [1.23, 2.73]
			14.10	488.00	TMLE untruncated	16.32	10.20	6.11 [4.17, 8.06]	16	
					TMLE truncated at 200	16.32	10.23	6.08 [4.14, 8.03]	16	
			2.33	39.96	IPW untruncated	16.34	10.05	6.28 [1.04, 11.53]	16	1.46 [0.65, 2.27]
					IPW truncated at 20	16.34	10.08	6.26 [1.01, 11.51]	16	1.45 [0.65, 2.25]
					IPW truncated at 99th	16.08	10.13	5.95 [0.80, 11.10]	17	1.45 [0.71, 2.19]
		Logistic model	4.31	76.53	IPW untruncated	17.65	9.63	8.01 [1.82, 14.21]	12	1.33 [0.44, 2.21]
					IPW truncated at 20	17.65	9.73	7.91 [1.71, 14.12]	13	1.30 [0.44, 2.16]
					IPW truncated at 99th	17.71	10.03	7.68 [1.51, 13.86]	13	1.27 [0.50, 2.03]
ITT		SL			Unadjusted	15.37	10.50	4.87 [1.88, 7.87]	21	1.64 [1.14, 2.14]
			10.20	301.23	TMLE untruncated	16.50	10.77	5.73 [4.12, 7.35]	17	
					TMLE truncated at 200	16.50	10.78	5.72 [4.11, 7.34]	17	
			2.23	41.14	IPW untruncated	16.51	10.77	5.74 [2.02, 9.46]	17	1.52 [0.83, 2.21]
					IPW truncated at 20	16.52	10.79	5.72 [2.00, 9.44]	17	1.52 [0.83, 2.20]
					IPW truncated at 99th	16.40	11.01	5.39 [1.74, 9.04]	19	1.49 [0.87, 2.10]
		Logistic model	4.11	99.75	IPW untruncated	16.59	9.76	6.82 [2.70, 10.94]	15	1.48 [0.65, 2.30]
					IPW truncated at 20	16.59	10.25	6.34 [2.26, 10.41]	16	1.43 [0.64, 2.21]
					IPW truncated at 99th	16.63	10.78	5.85 [1.85, 9.85]	17	1.35 [0.69, 2.02]

* Discontinuation refers to the interruption of the comparator medication initiated on index date; Crossover refers to the initiation of the comparator medication initiated by patient at baseline in the other arm.

© 2025 Neugebauer R et al. *JAMA Network Open*.

eFigure 41. MACE (Primary Definition), 2-Arm Drug Class Comparison, SGLT2is vs GLP-1RAs, No HF Subgroup, Cumulative Incidence Curves From PP and ITT Analyses With IPW, TMLE, and SL
Each plot emulates inferences among patients with No HF from a 2-arm RCT comparing SGLT2i and GLP-1RA and represents unadjusted or adjusted estimates of cumulative incidence curves for MACE derived with IPW and TMLE with SL estimates of propensity scores with four weight truncation schemes: IPW and TMLE without weight truncation (untruncated), IPW with truncation of stabilized weights at value 20 (trunc20) or at the 99th percentile of weight values (trunc99), and TMLE with truncation of unstabilized weights at value 200 (trunc200). The red divider line separates results of Per-Protocol (PP) analyses (top half) from Intention-To-Treat (ITT) analyses (bottom half). Each plot displays a p value for the test that the average risk difference (ARD) through 2.5 years of follow-up (30 months) is 0.

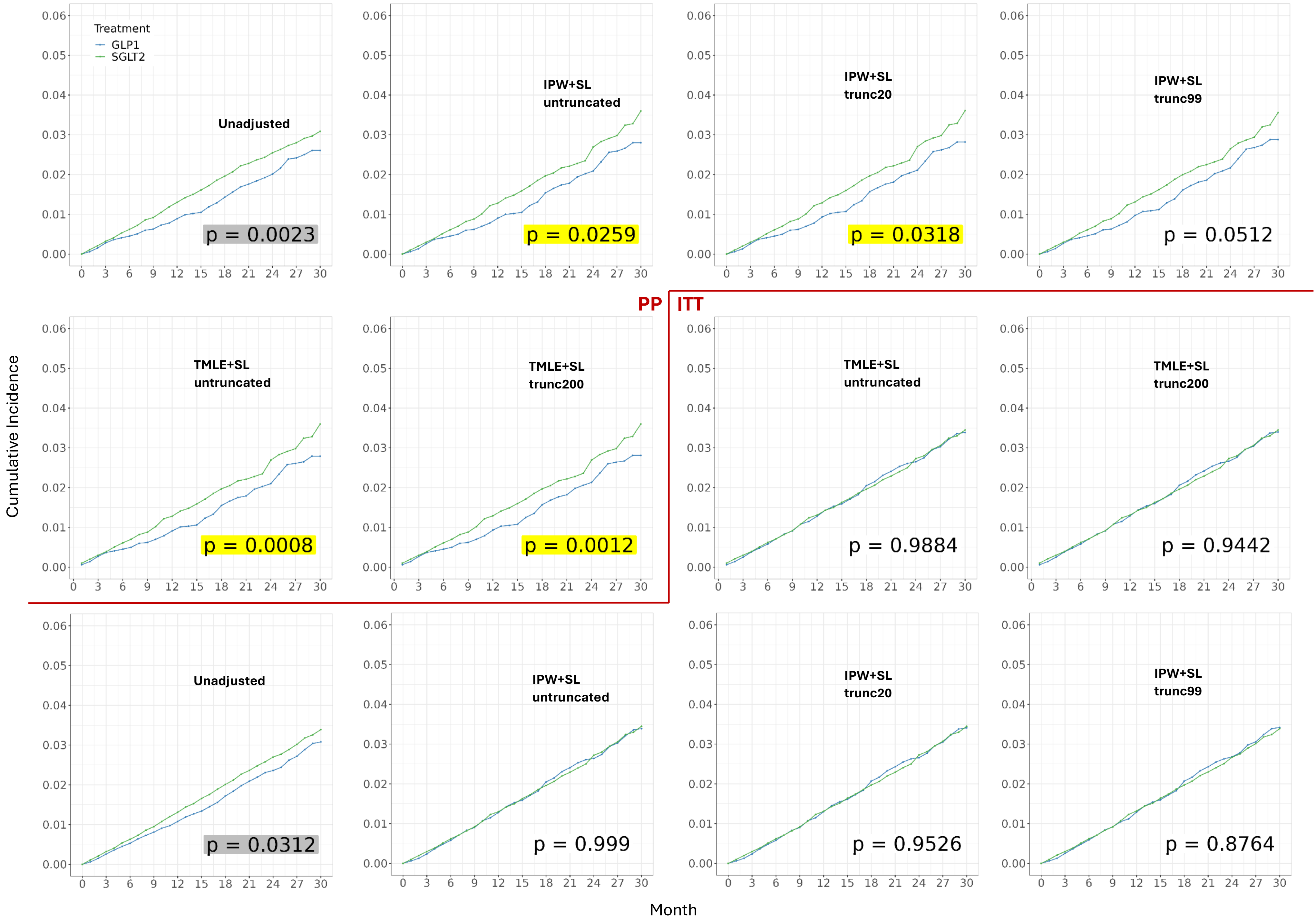

© 2025 Neugebauer R et al. *JAMA Network Open*.

eTable 40. MACE (Primary Definition), 2-Arm Drug Class Comparison, SGLT2is vs GLP-1RAs, No HF Subgroup, RD and HR Effect Measures at 2.5 Years

Estimation results among patients with No HF from ITT and PP analyses of emulated 2-arm RCTs comparing MACE risks over 2.5 years between SGLT2i and GLP-1RA initiators. For PP analyses, rates of protocol deviations are described by medication class initiated at baseline. Unadjusted point and interval estimates and adjusted point and interval IPW and TMLE estimates of risks, risk differences (RD), and hazard ratios (HR) based on propensity scores (PS) estimated with either logistic models or super learning (SL) are presented for four weight truncation schemes along with the corresponding 99th percentile and maximum value of the stabilized and unstabilized inverse probability weights used for implementing IPW and TMLE, respectively. RD is the risk in treatment arm minus the risk in control arm and NNT is the number needed to treat.

Analysis type	Protocol Deviations* by exposure group (%)	PS estimation	99th IP weights	Max IP weight	Estimator	Treatment (SGLT2i) risk in %	Control (GLP-1RA) risk in %	RD [95% CI] in %	NNT	HR [95% CI]
PP	Discontinuation<br>SGLT2i: 26.36<br>GLP-1RA: 44.82<br><br>Crossover<br>SGLT2i: 5.15<br>GLP-1RA: 9.72	SL			Unadjusted	3.09	2.61	0.48 [-0.10, 1.05]		1.44 [1.14, 1.74]
			16.53	1,571.01	TMLE untruncated	3.60	2.79	0.81 [0.38, 1.25]	123	
					TMLE truncated at 200	3.60	2.81	0.79 [0.36, 1.23]	126	
			3.19	217.14	IPW untruncated	3.60	2.80	0.80 [-0.15, 1.75]		1.41 [1.05, 1.77]
					IPW truncated at 20	3.61	2.82	0.79 [-0.16, 1.74]		1.41 [1.05, 1.76]
					IPW truncated at 99th	3.56	2.88	0.68 [-0.20, 1.57]		1.38 [1.03, 1.72]
		Logistic model	4.71	706.88	IPW untruncated	4.16	2.82	1.34 [-0.19, 2.87]		1.50 [1.07, 1.93]
					IPW truncated at 20	4.25	2.91	1.34 [-0.23, 2.90]		1.48 [1.06, 1.91]
					IPW truncated at 99th	4.19	3.02	1.17 [-0.21, 2.56]		1.39 [1.01, 1.78]
ITT		SL			Unadjusted	3.39	3.08	0.31 [-0.12, 0.74]		1.21 [1.01, 1.41]
			10.15	1,008.68	TMLE untruncated	3.45	3.39	0.06 [-0.29, 0.41]		
					TMLE truncated at 200	3.45	3.40	0.05 [-0.30, 0.40]		
			2.95	214.33	IPW untruncated	3.45	3.39	0.06 [-0.56, 0.68]		1.01 [0.79, 1.24]
					IPW truncated at 20	3.45	3.41	0.04 [-0.58, 0.67]		1.01 [0.79, 1.24]
					IPW truncated at 99th	3.39	3.42	-0.03 [-0.58, 0.53]		1.02 [0.81, 1.23]
		Logistic model	4.26	868.93	IPW untruncated	3.41	3.47	-0.06 [-0.88, 0.76]		1.01 [0.68, 1.33]
					IPW truncated at 20	3.44	3.49	-0.05 [-0.84, 0.74]		1.03 [0.73, 1.32]
					IPW truncated at 99th	3.34	3.49	-0.15 [-0.80, 0.50]		1.03 [0.80, 1.25]

* Discontinuation refers to the interruption of the comparator medication initiated on index date; Crossover refers to the initiation of the comparator medication initiated by patient at baseline in the other arm.

© 2025 Neugebauer R et al. *JAMA Network Open*.

eFigure 42. MACE (Primary Definition), 2-Arm Drug Class Comparison, SGLT2is vs GLP-1RAs, No HF and No ASCVD Subgroup, Cumulative Incidence Curves From PP and ITT Analyses With IPW, TMLE, and SL
Each plot emulates inferences among patients with No HF and No ASCVD from a 2-arm RCT comparing SGLT2i and GLP-1RA and represents unadjusted or adjusted estimates of cumulative incidence curves for MACE derived with IPW and TMLE with SL estimates of propensity scores with four weight truncation schemes: IPW and TMLE without weight truncation (untruncated), IPW with truncation of stabilized weights at value 20 (trunc20) or at the 99th percentile of weight values (trunc99), and TMLE with truncation of unstabilized weights at value 200 (trunc200). The red divider line separates results of Per-Protocol (PP) analyses (top half) from Intention-To-Treat (ITT) analyses (bottom half). Each plot displays a p value for the test that the average risk difference (ARD) through 2.5 years of follow-up (30 months) is 0.

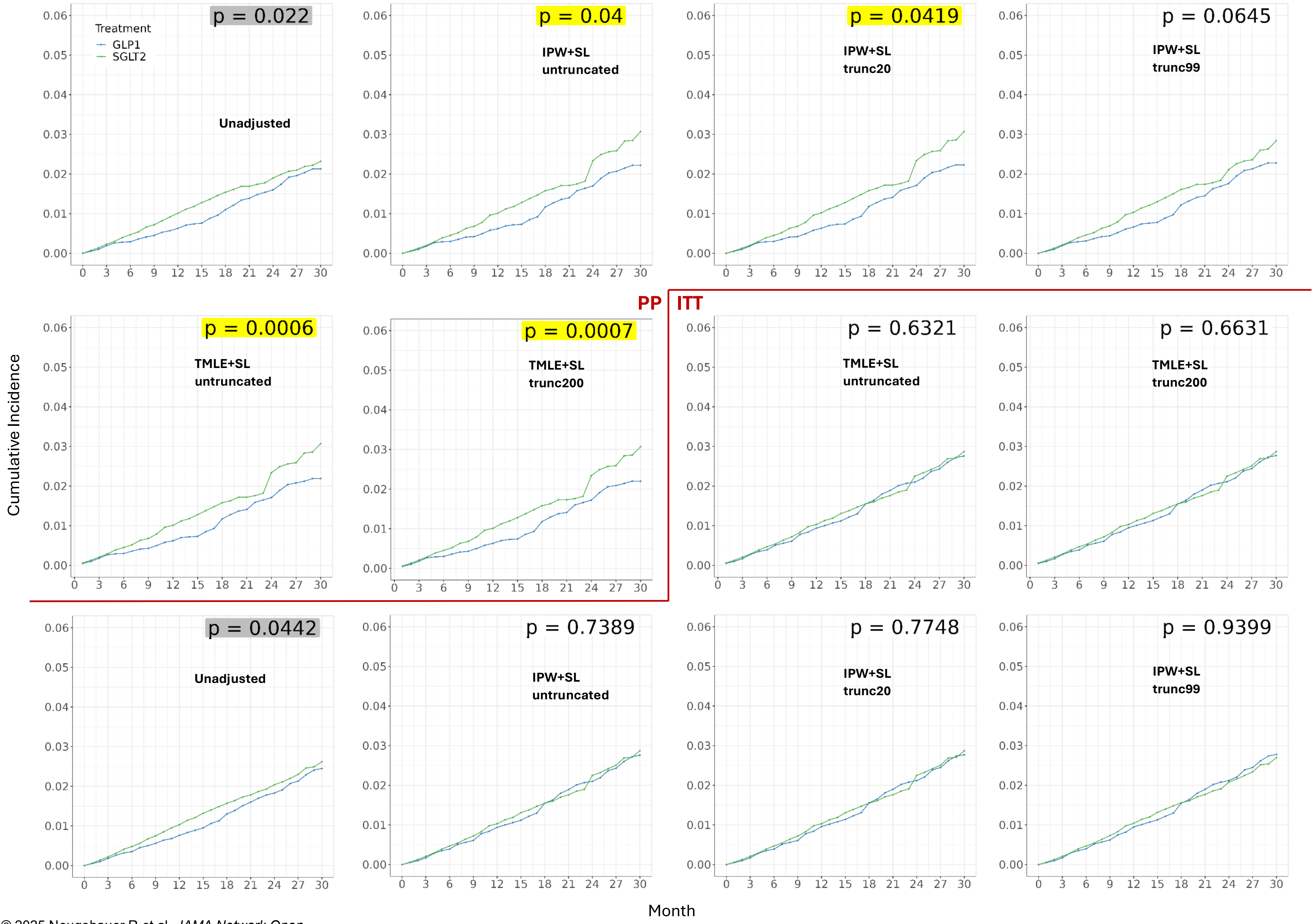

© 2025 Neugebauer R et al. *JAMA Network Open*.

eTable 41. MACE (Primary Definition), 2-Arm Drug Class Comparison, SGLT2is vs GLP-1RAs, No HF and No ASCVD Subgroup, RD and HR Effect Measures at 2.5 Years

Estimation results among patients with No HF and No ASCVD from ITT and PP analyses of emulated 2-arm RCTs comparing MACE risks over 2.5 years between SGLT2i and GLP-1RA initiators. For PP analyses, rates of protocol deviations are described by medication class initiated at baseline. Unadjusted point and interval estimates and adjusted point and interval IPW and TMLE estimates of risks, risk differences (RD), and hazard ratios (HR) based on propensity scores (PS) estimated with either logistic models or super learning (SL) are presented for four weight truncation schemes along with the corresponding 99th percentile and maximum value of the stabilized and unstabilized inverse probability weights used for implementing IPW and TMLE, respectively. RD is the risk in treatment arm minus the risk in control arm and NNT is the number needed to treat.

Analysis type	Protocol Deviations* by exposure group (%)	PS estimation	99th IP weights	Max IP weight	Estimator	Treatment (SGLT2i) risk in %	Control (GLP-1RA) risk in %	RD [95% CI] in %	NNT	HR [95% CI]
PP	Discontinuation<br>SGLT2i: 26.46<br>GLP-1RA: 44.72<br><br>Crossover<br>SGLT2i: 5.09<br>GLP-1RA: 9.46	SL			Unadjusted	2.32	2.13	0.19 [-0.35, 0.74]		1.56 [1.16, 1.96]
			16.40	1,906.81	TMLE untruncated	3.07	2.19	0.88 [0.36, 1.41]	113	
					TMLE truncated at 200	3.07	2.20	0.87 [0.35, 1.40]	115	
			3.18	227.11	IPW untruncated	3.07	2.22	0.84 [-0.31, 2.00]		1.56 [1.06, 2.06]
					IPW truncated at 20	3.07	2.23	0.84 [-0.31, 1.99]		1.55 [1.06, 2.05]
					IPW truncated at 99th	2.84	2.28	0.56 [-0.28, 1.40]		1.51 [1.03, 1.98]
		Logistic model	4.75	62,297.58	IPW untruncated	2.26	2.18	0.08 [-0.99, 1.15]		0.80 [0.11, 1.49]
					IPW truncated at 20	3.03	2.22	0.81 [-0.24, 1.87]		1.58 [1.02, 2.15]
					IPW truncated at 99th	3.17	2.30	0.86 [-0.23, 1.96]		1.51 [0.99, 2.03]
ITT		SL			Unadjusted	2.62	2.45	0.17 [-0.24, 0.58]		1.36 [1.08, 1.65]
			10.11	1,208.34	TMLE untruncated	2.87	2.76	0.11 [-0.28, 0.51]		
					TMLE truncated at 200	2.87	2.77	0.10 [-0.29, 0.50]		
			2.95	259.40	IPW untruncated	2.87	2.76	0.11 [-0.64, 0.86]		1.13 [0.81, 1.45]
					IPW truncated at 20	2.87	2.77	0.10 [-0.65, 0.86]		1.13 [0.81, 1.45]
					IPW truncated at 99th	2.70	2.78	-0.07 [-0.62, 0.48]		1.14 [0.85, 1.44]
		Logistic model	4.33	68,007.67	IPW untruncated	0.81	2.89	-2.08 [-3.12, -1.05]	48	0.45 [-0.04, 0.94]
					IPW truncated at 20	2.70	2.89	-0.18 [-0.95, 0.59]		1.08 [0.67, 1.49]
					IPW truncated at 99th	2.67	2.86	-0.20 [-0.86, 0.46]		1.13 [0.81, 1.45]

* Discontinuation refers to the interruption of the comparator medication initiated on index date; Crossover refers to the initiation of the comparator medication initiated by patient at baseline in the other arm.

© 2025 Neugebauer R et al. *JAMA Network Open*.

eFigure 43. MACE (Primary Definition), 2-Arm Drug Class Comparison, SGLT2is vs GLP-1RAs, No HF and ASCVD Subgroup, Cumulative Incidence Curves From PP and ITT Analyses With IPW, TMLE, and SL
Each plot emulates inferences among patients with No HF and ASCVD from a 2-arm RCT comparing SGLT2i and GLP-1RA and represents unadjusted or adjusted estimates of cumulative incidence curves for MACE derived with IPW and TMLE with SL estimates of propensity scores with four weight truncation schemes: IPW and TMLE without weight truncation (untruncated), IPW with truncation of stabilized weights at value 20 (trunc20) or at the 99th percentile of weight values (trunc99), and TMLE with truncation of unstabilized weights at value 200 (trunc200). The red divi der line separates results of Per-Protocol (PP) analyses (top half) from Intention-To-Treat (ITT) analyses (bottom half). Each plot displays a p value for the test that the average risk difference (ARD) through 2.5 years of follow-up (30 months) is 0.

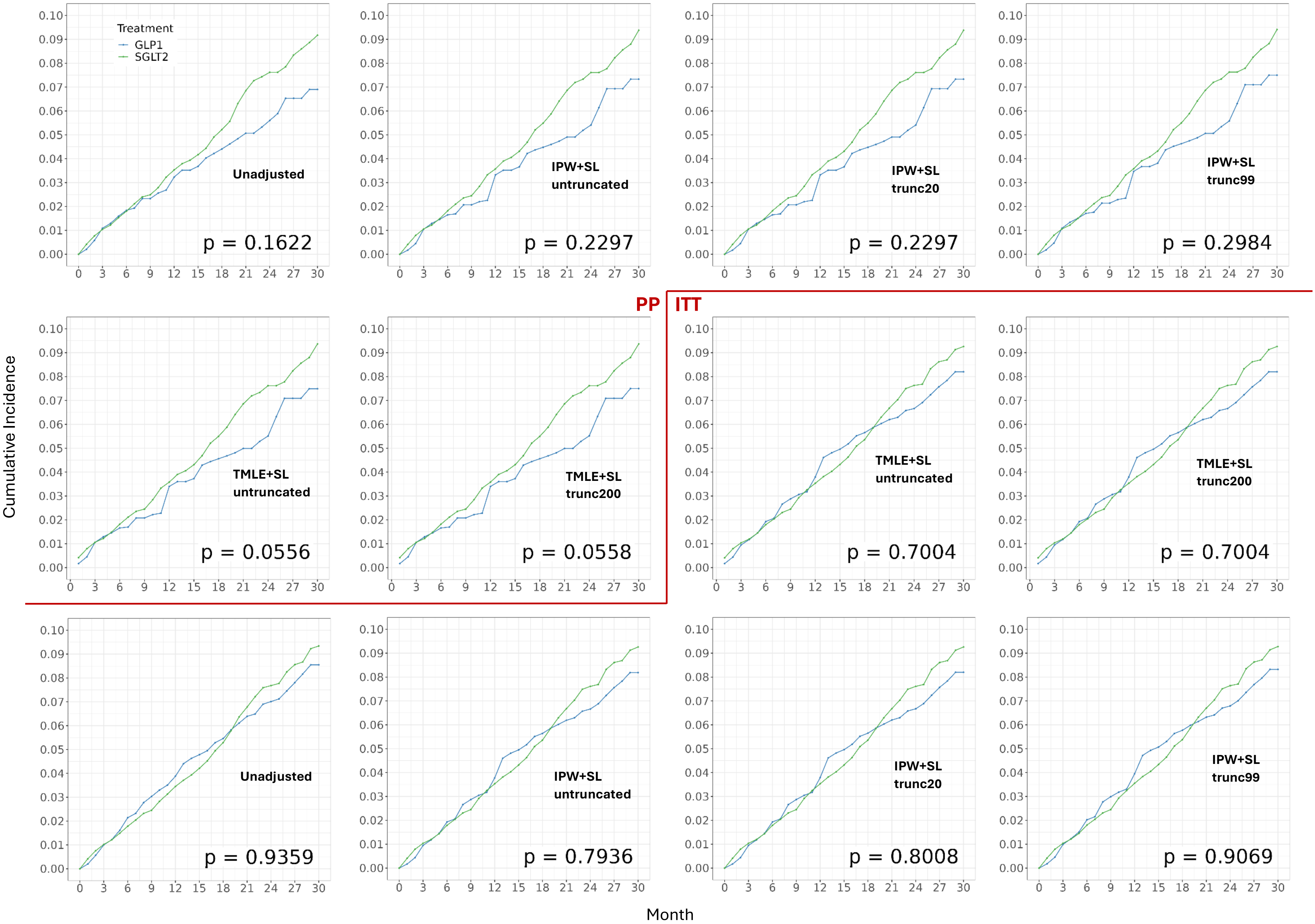

© 2025 Neugebauer R et al. *JAMA Network Open*.

eTable 42. MACE (Primary Definition), 2-Arm Drug Class Comparison, SGLT2is vs GLP-1RAs, No HF and ASCVD Subgroup, RD and HR Effect Measures at 2.5 Years

Estimation results among patients with No HF and ASCVD from ITT and PP analyses of emulated 2-arm RCTs comparing MACE risks over 2.5 years between SGLT2i and GLP-1RA initiators. For PP analyses, rates of protocol deviations are described by medication class initiated at baseline. Unadjusted point and interval estimates and adjusted point and interval IPW and TMLE estimates of risks, risk differences (RD), and hazard ratios (HR) based on propensity scores (PS) estimated with either logistic models or super learning (SL) are presented for four weight truncation schemes along with the corresponding 99th percentile and maximum value of the stabilized and unstabilized inverse probability weights used for implementing IPW and TMLE, respectively. RD is the risk in treatment arm minus the risk in control arm and NNT is the number needed to treat.

Analysis type	Protocol Deviations* by exposure group (%)	PS estimation	99th IP weights	Max IP weight	Estimator	Treatment (SGLT2i) risk in %	Control (GLP-1RA) risk in %	RD [95% CI] in %	NNT	HR [95% CI]
PP	Discontinuation<br>SGLT2i: 25.61<br>GLP-1RA: 45.72<br><br>Crossover<br>SGLT2i: 5.59<br>GLP-1RA: 12.01	SL			Unadjusted	9.17	6.90	2.27 [-0.56, 5.10]		1.10 [0.72, 1.49]
			13.18	223.58	TMLE untruncated	9.37	7.49	1.88 [0.46, 3.30]	53	
					TMLE truncated at 200	9.37	7.50	1.88 [0.46, 3.30]	53	
			2.60	17.02	IPW untruncated	9.38	7.33	2.05 [-1.50, 5.60]		1.20 [0.71, 1.69]
					IPW truncated at 20	9.38	7.33	2.05 [-1.50, 5.60]		1.20 [0.71, 1.69]
					IPW truncated at 99th	9.41	7.50	1.90 [-1.68, 5.48]		1.16 [0.68, 1.63]
		Logistic model	4.09	22.48	IPW untruncated	9.65	7.13	2.52 [-1.68, 6.72]		1.36 [0.77, 1.96]
					IPW truncated at 20	9.65	7.13	2.52 [-1.68, 6.72]		1.36 [0.77, 1.96]
					IPW truncated at 99th	9.51	7.25	2.26 [-1.88, 6.39]		1.29 [0.75, 1.83]
ITT		SL			Unadjusted	9.34	8.55	0.79 [-1.30, 2.88]		0.86 [0.62, 1.10]
			9.31	162.55	TMLE untruncated	9.26	8.20	1.07 [-0.19, 2.32]		
					TMLE truncated at 200	9.26	8.20	1.07 [-0.19, 2.32]		
			2.53	31.15	IPW untruncated	9.26	8.19	1.07 [-1.30, 3.44]		0.90 [0.60, 1.20]
					IPW truncated at 20	9.26	8.20	1.06 [-1.31, 3.44]		0.90 [0.60, 1.20]
					IPW truncated at 99th	9.28	8.32	0.97 [-1.40, 3.33]		0.87 [0.58, 1.16]
		Logistic model	4.03	46.77	IPW untruncated	9.50	7.82	1.68 [-1.07, 4.43]		0.98 [0.62, 1.34]
					IPW truncated at 20	9.55	7.83	1.73 [-1.02, 4.47]		0.98 [0.62, 1.34]
					IPW truncated at 99th	9.53	8.05	1.48 [-1.25, 4.21]		0.94 [0.60, 1.27]

* Discontinuation refers to the interruption of the comparator medication initiated on index date; Crossover refers to the initiation of the comparator medication initiated by patient at baseline in the other arm.

© 2025 Neugebauer R et al. *JAMA Network Open*.

eFigure 44. MACE (Primary Definition), 2-Arm Drug Class Comparison, SGLT2is vs GLP-1RAs, HF and No ASCVD Subgroup, Cumulative Incidence Curves From PP and ITT Analyses With IPW, TMLE, and SL
Each plot emulates inferences among patients with HF and No ASCVD from a 2-arm RCT comparing SGLT2i and GLP-1RA and represents unadjusted or adjusted estimates of cumulative incidence curves for MACE derived with IPW and TMLE with SL estimates of propensity scores with four weight truncation schemes: IPW and TMLE without weight truncation (untruncated), IPW with truncation of stabilized weights at value 20 (trunc20) or at the 99th percentile of weight values (trunc99), and TMLE with truncation of unstabilized weights at value 200 (trunc200). The red divider line separates results of Per-Protocol (PP) analyses (top half) from Intention-To-Treat (ITT) analyses (bottom half). Each plot displays a p value for the test that the average risk difference (ARD) through 2.5 years of follow-up (30 months) is 0.

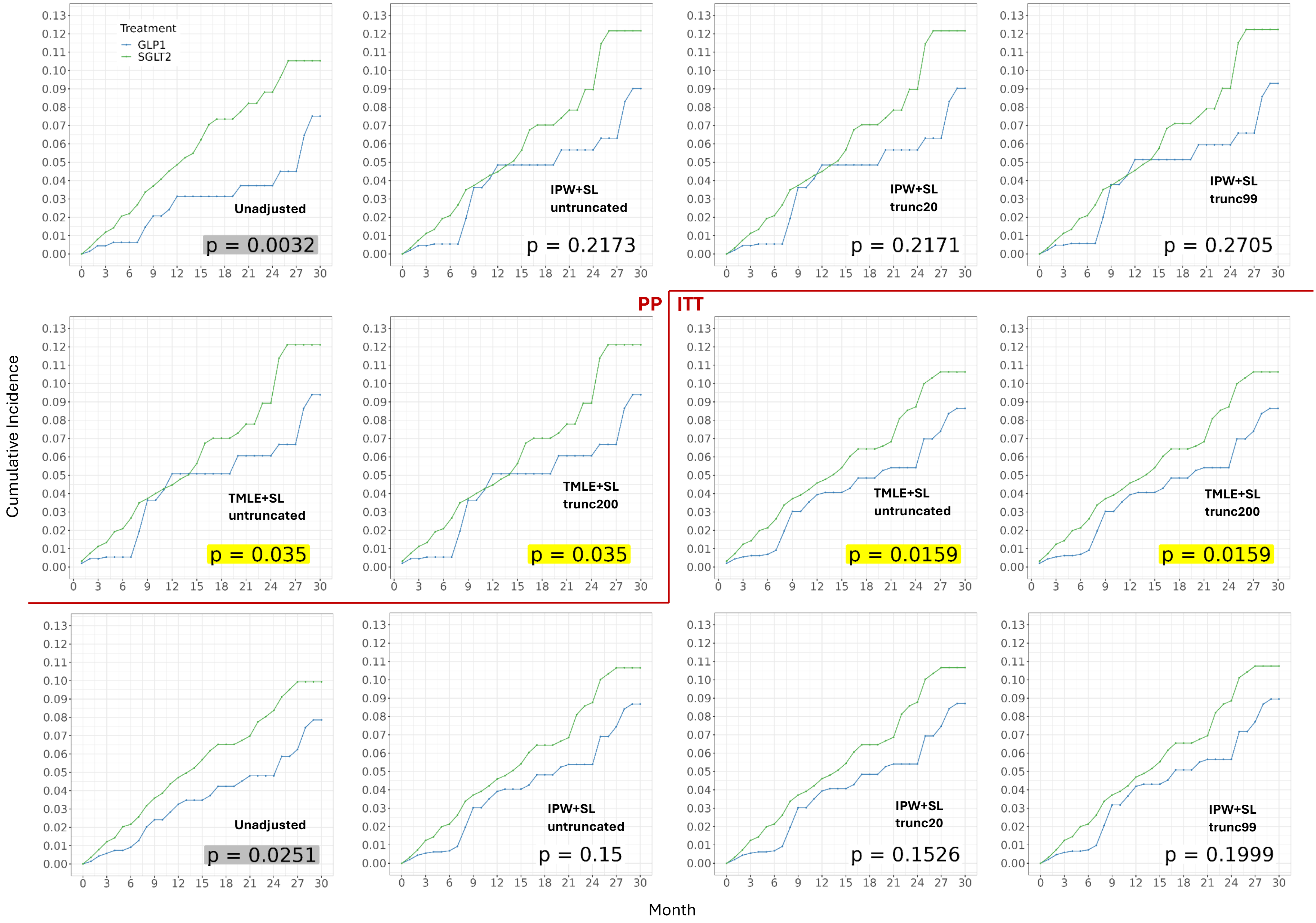

© 2025 Neugebauer R et al. *JAMA Network Open*.

eTable 43. MACE (Primary Definition), 2-Arm Drug Class Comparison, SGLT2is vs GLP-1RAs, HF and No ASCVD Subgroup, RD and HR Effect Measures at 2.5 Years

Estimation results among patients with HF and No ASCVD from ITT and PP analyses of emulated 2-arm RCTs comparing MACE risks over 2.5 years between SGLT2i and GLP-1RA initiators. For PP analyses, rates of protocol deviations are described by medication class initiated at baseline. Unadjusted point and interval estimates and adjusted point and interval IPW and TMLE estimates of risks, risk differences (RD), and hazard ratios (HR) based on propensity scores (PS) estimated with either logistic models or super learning (SL) are presented for four weight truncation schemes along with the corresponding 99th percentile and maximum value of the stabilized and unstabilized inverse probability weights used for implementing IPW and TMLE, respectively. RD is the risk in treatment arm minus the risk in control arm and NNT is the number needed to treat.

Analysis type	Protocol Deviations* by exposure group (%)	PS estimation	99th IP weights	Max IP weight	Estimator	Treatment (SGLT2i) risk in %	Control (GLP-1RA) risk in %	RD [95% CI] in %	NNT	HR [95% CI]
PP	Discontinuation<br>SGLT2i: 19.39<br>GLP-1RA: 41.03<br><br>Crossover<br>SGLT2i: 3.82<br>GLP-1RA: 10.99	SL			Unadjusted	10.53	7.51	3.02 [-2.41, 8.46]		2.09 [0.70, 3.48]
			15.56	236.59	TMLE untruncated	12.11	9.39	2.72 [0.33, 5.11]	37	
					TMLE truncated at 200	12.11	9.39	2.72 [0.33, 5.11]	37	
			2.88	23.23	IPW untruncated	12.16	9.02	3.13 [-4.41, 10.68]		1.46 [0.35, 2.57]
					IPW truncated at 20	12.16	9.03	3.14 [-4.41, 10.69]		1.46 [0.35, 2.57]
					IPW truncated at 99th	12.23	9.30	2.93 [-4.68, 10.53]		1.39 [0.34, 2.45]
		Logistic model	2.48	8.23	IPW untruncated	12.47	8.79	3.67 [-3.83, 11.17]		1.32 [0.23, 2.41]
					IPW truncated at 20	12.47	8.79	3.67 [-3.83, 11.17]		1.32 [0.23, 2.41]
					IPW truncated at 99th	12.51	8.78	3.73 [-3.70, 11.15]		1.33 [0.26, 2.41]
ITT		SL			Unadjusted	9.94	7.86	2.08 [-1.67, 5.83]		1.62 [0.74, 2.50]
			12.29	202.92	TMLE untruncated	10.63	8.64	1.99 [0.08, 3.89]	50	
					TMLE truncated at 200	10.63	8.64	1.99 [0.08, 3.89]	50	
			2.70	30.01	IPW untruncated	10.65	8.68	1.96 [-2.84, 6.77]		1.46 [0.52, 2.41]
					IPW truncated at 20	10.66	8.71	1.95 [-2.86, 6.76]		1.46 [0.52, 2.41]
					IPW truncated at 99th	10.75	8.95	1.80 [-3.06, 6.66]		1.39 [0.50, 2.27]
		Logistic model	2.53	15.86	IPW untruncated	10.56	8.77	1.79 [-2.77, 6.35]		1.37 [0.50, 2.24]
					IPW truncated at 20	10.56	8.77	1.79 [-2.77, 6.35]		1.37 [0.50, 2.24]
					IPW truncated at 99th	10.61	8.94	1.67 [-2.93, 6.27]		1.33 [0.49, 2.18]

* Discontinuation refers to the interruption of the comparator medication initiated on index date; Crossover refers to the initiation of the comparator medication initiated by patient at baseline in the other arm.

© 2025 Neugebauer R et al. *JAMA Network Open*.

eFigure 45. MACE (Primary Definition), 2-Arm Drug Class Comparison, SGLT2is vs GLP-1RAs, HF and ASCVD Subgroup, Cumulative Incidence Curves From PP and ITT Analyses With IPW, TMLE, and SL
Each plot emulates inferences among patients with HF and ASCVD from a 2-arm RCT comparing SGLT2i and GLP-1RA and represents unadjusted or adjusted estimates of cumulative incidence curves for MACE derived with IPW and TMLE with SL estimates of propensity scores with four weight truncation schemes: IPW and TMLE without weight truncation (untruncated), IPW with truncation of stabilized weights at value 20 (trunc20) or at the 99th percentile of weight values (trunc99), and TMLE with truncation of unstabilized weights at value 200 (trunc200). The red divider line separates results of Per-Protocol (PP) analyses (top half) from Intention-To-Treat (ITT) analyses (bottom half). Each plot displays a p value for the test that the average risk difference (ARD) through 2.5 years of follow-up (30 months) is 0.

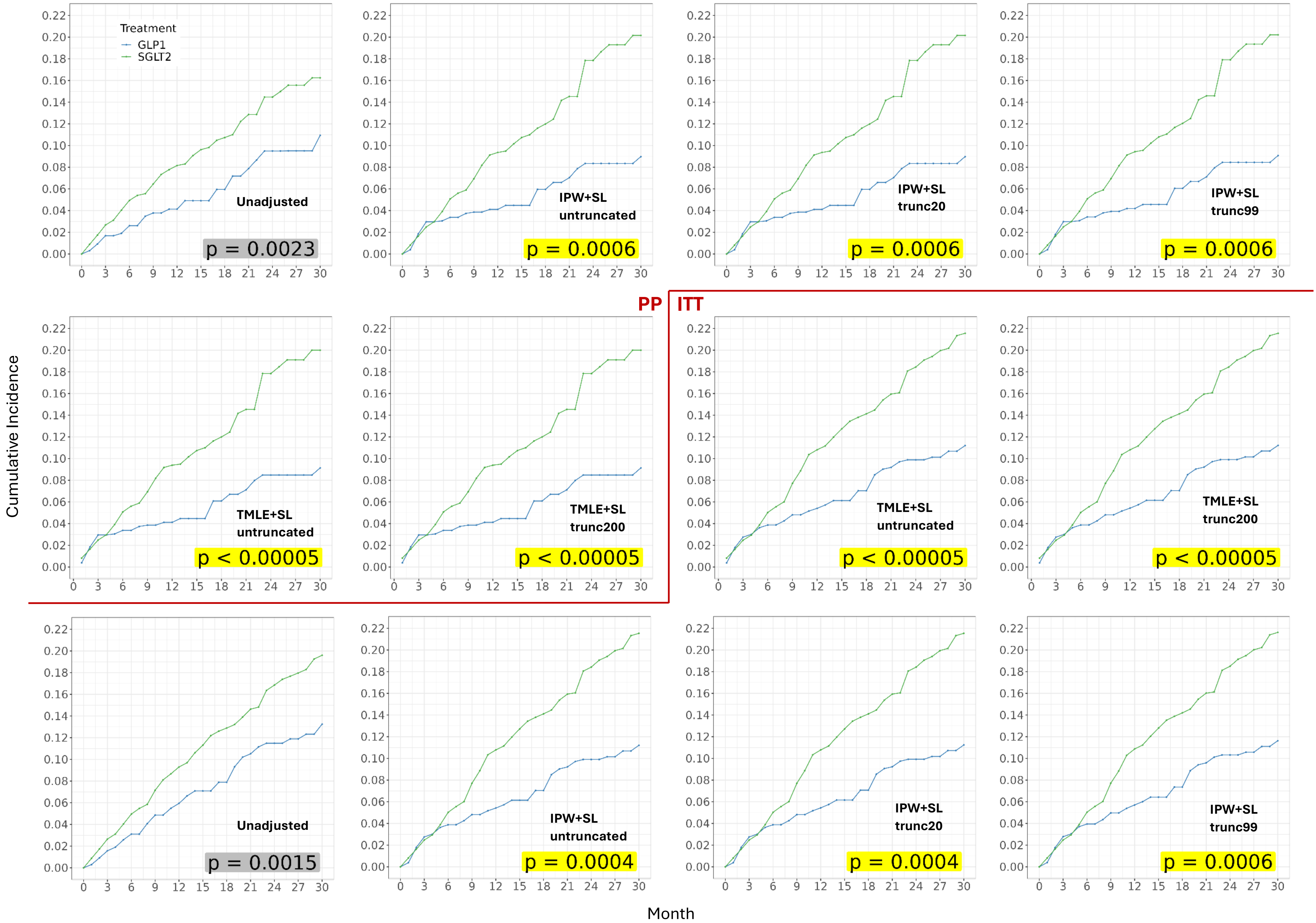

© 2025 Neugebauer R et al. *JAMA Network Open*.

eTable 44. MACE (Primary Definition), 2-Arm Drug Class Comparison, SGLT2is vs GLP-1RAs, HF and ASCVD Subgroup, RD and HR Effect Measures at 2.5 Years

Estimation results among patients with HF and ASCVD from ITT and PP analyses of emulated 2-arm RCTs comparing MACE risks over 2.5 years between SGLT2i and GLP-1RA initiators. For PP analyses, rates of protocol deviations are described by medication class initiated at baseline. Unadjusted point and interval estimates and adjusted point and interval IPW and TMLE estimates of risks, risk differences (RD), and hazard ratios (HR) based on propensity scores (PS) estimated with either logistic models or super learning (SL) are presented for four weight truncation schemes along with the corresponding 99th percentile and maximum value of the stabilized and unstabilized inverse probability weights used for implementing IPW and TMLE, respectively. RD is the risk in treatment arm minus the risk in control arm and NNT is the number needed to treat.

Analysis type	Protocol Deviations* by exposure group (%)	PS estimation	99th IP weights	Max IP weight	Estimator	Treatment (SGLT2i) risk in %	Control (GLP-1RA) risk in %	RD [95% CI] in %	NNT	HR [95% CI]
PP	Discontinuation<br>SGLT2i: 19.60<br>GLP-1RA: 43.60<br><br>Crossover<br>SGLT2i: 3.88<br>GLP-1RA: 11.61	SL			Unadjusted	16.25	10.94	5.31 [-0.59, 11.22]		1.97 [1.02, 2.92]
			16.42	593.75	TMLE untruncated	20.00	9.14	10.86 [8.11, 13.61]	9	
					TMLE truncated at 200	20.00	9.14	10.86 [8.11, 13.61]	9	
			2.52	45.29	IPW untruncated	20.16	8.98	11.18 [4.34, 18.02]	9	1.78 [0.53, 3.03]
					IPW truncated at 20	20.16	8.98	11.18 [4.34, 18.02]	9	1.76 [0.52, 3.00]
					IPW truncated at 99th	20.21	9.08	11.14 [4.29, 17.98]	9	1.73 [0.55, 2.91]
		Logistic model	2.50	30.27	IPW untruncated	19.91	9.56	10.34 [3.54, 17.15]	10	1.64 [0.43, 2.85]
					IPW truncated at 20	19.91	9.56	10.34 [3.54, 17.15]	10	1.63 [0.43, 2.84]
					IPW truncated at 99th	20.02	9.33	10.70 [4.01, 17.39]	9	1.79 [0.62, 2.95]
ITT		SL			Unadjusted	19.60	13.25	6.35 [1.83, 10.88]	16	1.61 [1.01, 2.21]
			12.05	323.14	TMLE untruncated	21.56	11.20	10.36 [7.84, 12.88]	10	
					TMLE truncated at 200	21.56	11.22	10.34 [7.82, 12.86]	10	
			2.48	40.70	IPW untruncated	21.54	11.21	10.33 [5.22, 15.44]	10	1.80 [0.80, 2.80]
					IPW truncated at 20	21.54	11.24	10.30 [5.19, 15.41]	10	1.78 [0.79, 2.77]
					IPW truncated at 99th	21.62	11.63	10.00 [4.88, 15.12]	10	1.71 [0.78, 2.63]
		Logistic model	2.53	31.77	IPW untruncated	21.61	11.61	10.00 [4.78, 15.23]	10	1.71 [0.73, 2.69]
					IPW truncated at 20	21.61	11.62	9.99 [4.77, 15.21]	10	1.71 [0.73, 2.68]
					IPW truncated at 99th	21.65	11.63	10.02 [4.93, 15.12]	10	1.73 [0.84, 2.62]

* Discontinuation refers to the interruption of the comparator medication initiated on index date; Crossover refers to the initiation of the comparator medication initiated by patient at baseline in the other arm.

© 2025 Neugebauer R et al. *JAMA Network Open*.

eFigure 46. MACE (Primary Definition), 2-Arm Drug Class Comparison, SGLT2is vs GLP-1RAs, Low CKD Risk Subgroup, Cumulative Incidence Curves From PP and ITT Analyses With IPW, TMLE, and SL
Each plot emulates inferences among patients with Low CKD Risk from a 2-arm RCT comparing SGLT2i and GLP-1RA and represents unadjusted or adjusted estimates of cumulative incidence curves for MACE derived with IPW and TMLE with SL estimates of propensity scores with four weight truncation schemes: IPW and TMLE without weight truncation (untruncated), IPW with truncation of stabilized weights at value 20 (trunc20) or at the 99th percentile of weight values (trunc99), and TMLE with truncation of unstabilized weights at value 200 (trunc200). The red divi der line separates results of Per-Protocol (PP) analyses (top half) from Intention-To-Treat (ITT) analyses (bottom half). Each plot displays a p value for the test that the average risk difference (ARD) through 2.5 years of follow-up (30 months) is 0.

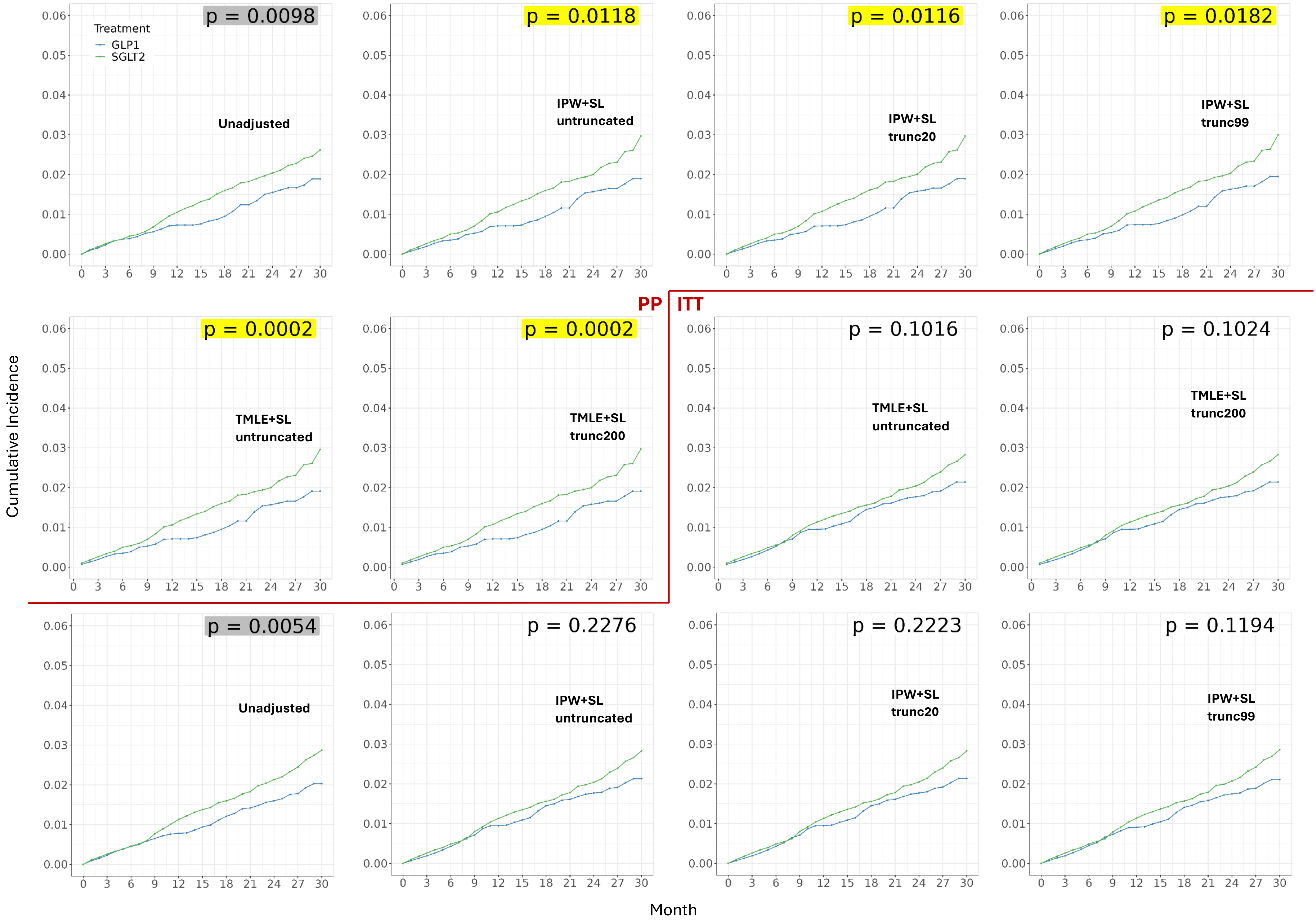

© 2025 Neugebauer R et al. *JAMA Network Open*.

eTable 45. MACE (Primary Definition), 2-Arm Drug Class Comparison, SGLT2is vs GLP-1RAs, Low CKD Risk Subgroup, RD and HR Effect Measures at 2.5 Years

Estimation results among patients with Low CKD risk from ITT and PP analyses of emulated 2-arm RCTs comparing MACE risks over 2.5 years between SGLT2i and GLP-1RA initiators. For PP analyses, rates of protocol deviations are described by medication class initiated at baseline. Unadjusted point and interval estimates and adjusted point and interval IPW and TMLE estimates of risks, risk differences (RD), and hazard ratios (HR) based on propensity scores (PS) estimated with either logistic models or super learning (SL) are presented for four weight truncation schemes along with the corresponding 99th percentile and maximum value of the stabilized and unstabilized inverse probability weights used for implementing IPW and TMLE, respectively. RD is the risk in treatment arm minus the risk in control arm and NNT is the number needed to treat.

Analysis type	Protocol Deviations* by exposure group (%)	PS estimation	99th IP weights	Max IP weight	Estimator	Treatment (SGLT2i) risk in %	Control (GLP-1RA) risk in %	RD [95% CI] in %	NNT	HR [95% CI]
PP	Discontinuation<br>SGLT2i: 26.83<br>GLP-1RA: 44.99<br><br>Crossover<br>SGLT2i: 5.01<br>GLP-1RA: 9.71	SL			Unadjusted	2.62	1.89	0.72 [0.03, 1.42]	138	1.47 [1.00, 1.94]
			15.21	1,386.32	TMLE untruncated	2.96	1.91	1.06 [0.63, 1.49]	95	
					TMLE truncated at 200	2.97	1.91	1.05 [0.62, 1.48]	95	
			2.86	149.72	IPW untruncated	2.97	1.90	1.07 [0.11, 2.03]	94	1.55 [0.98, 2.12]
					IPW truncated at 20	2.97	1.90	1.07 [0.11, 2.03]	93	1.55 [0.98, 2.12]
					IPW truncated at 99th	3.00	1.95	1.05 [0.08, 2.02]	95	1.50 [0.95, 2.05]
		Logistic model	4.39	190.81	IPW untruncated	3.44	4.16	-0.72 [-5.83, 4.39]		1.42 [0.81, 2.02]
					IPW truncated at 20	3.47	4.42	-0.94 [-6.27, 4.38]		1.41 [0.81, 2.02]
					IPW truncated at 99th	3.57	2.80	0.77 [-1.56, 3.10]		1.33 [0.78, 1.89]
ITT		SL			Unadjusted	2.87	2.03	0.84 [0.33, 1.36]	119	1.41 [1.04, 1.78]
			9.65	825.48	TMLE untruncated	2.83	2.14	0.69 [0.32, 1.06]	145	
					TMLE truncated at 200	2.83	2.14	0.69 [0.32, 1.06]	145	
			2.72	116.92	IPW untruncated	2.83	2.13	0.69 [0.07, 1.32]	144	1.21 [0.78, 1.63]
					IPW truncated at 20	2.83	2.14	0.70 [0.07, 1.32]	143	1.21 [0.78, 1.63]
					IPW truncated at 99th	2.86	2.11	0.74 [0.16, 1.33]	134	1.24 [0.85, 1.64]
		Logistic model	4.12	205.47	IPW untruncated	2.77	2.20	0.57 [-0.18, 1.32]		1.00 [0.56, 1.44]
					IPW truncated at 20	2.78	2.22	0.56 [-0.20, 1.32]		1.00 [0.56, 1.44]
					IPW truncated at 99th	2.80	2.16	0.63 [-0.02, 1.29]		1.07 [0.69, 1.44]

* Discontinuation refers to the interruption of the comparator medication initiated on index date; Crossover refers to the initiation of the comparator medication initiated by patient at baseline in the other arm.

© 2025 Neugebauer R et al. *JAMA Network Open*.

eFigure 47. MACE (Primary Definition), 2-Arm Drug Class Comparison, SGLT2is vs GLP-1RAs, Moderate CKD Risk Subgroup, Cumulative Incidence Curves From PP and ITT Analyses With IPW, TMLE, and SL
Each plot emulates inferences among patients with Moderate CKD Risk from a 2-arm RCT comparing SGLT2i and GLP-1RA and represents unadjusted or adjusted estimates of cumulative incidence curves for MACE derived with IPW and TMLE with SL estimates of propensity scores with four weight truncation schemes: IPW and TMLE without weight truncation (untruncated), IPW with truncation of stabilized weights at value 20 (trunc20) or at the 99th percentile of weight values (trunc99), and TMLE with truncation of unstabilized weights at value 200 (trunc200). The red divider line separates results of Per-Protocol (PP) analyses (top half) from Intention-To-Treat (ITT) analyses (bottom half). Each plot displays a p value for the test that the average risk difference (ARD) through 2.5 years of follow-up (30 months) is 0.

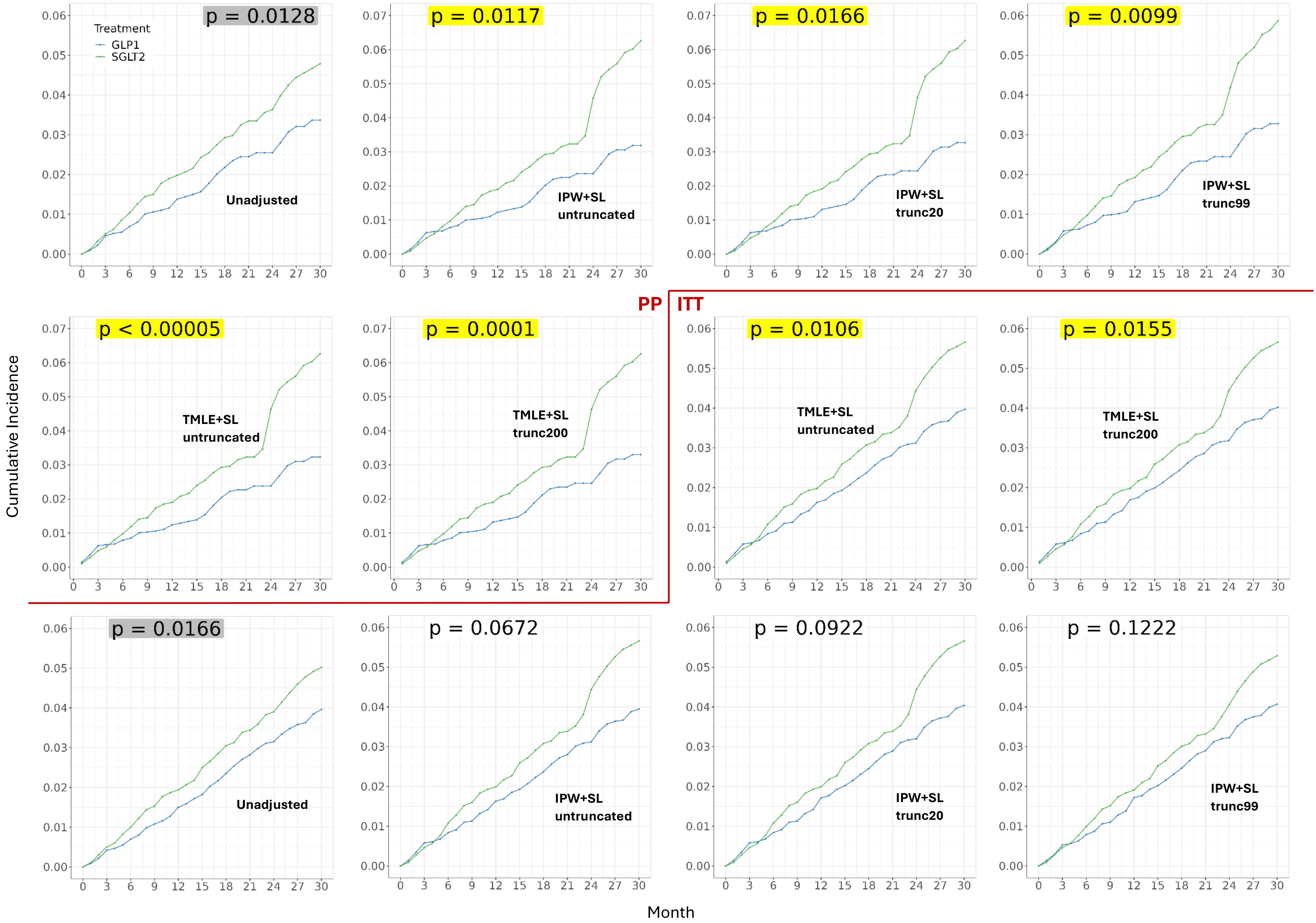

© 2025 Neugebauer R et al. *JAMA Network Open*.

eTable 46. MACE (Primary Definition), 2-Arm Drug Class Comparison, SGLT2is vs GLP-1RAs, Moderate CKD Risk Subgroup, RD and HR Effect Measures at 2.5 Years

Estimation results among patients with Moderate CKD risk from ITT and PP analyses of emulated 2-arm RCTs comparing MACE risks over 2.5 years between SGLT2i and GLP-1RA initiators. For PP analyses, rates of protocol deviations are described by medication class initiated at baseline. Unadjusted point and interval estimates and adjusted point and interval IPW and TMLE estimates of risks, risk differences (RD), and hazard ratios (HR) based on propensity scores (PS) estimated with either logistic models or super learning (SL) are presented for four weight truncation schemes along with the corresponding 99th percentile and maximum value of the stabilized and unstabilized inverse probability weights used for implementing IPW and TMLE, respectively. RD is the risk in treatment arm minus the risk in control arm and NNT is the number needed to treat.

Analysis type	Protocol Deviations* by exposure group (%)	PS estimation	99th IP weights	Max IP weight	Estimator	Treatment (SGLT2i) risk in %	Control (GLP-1RA) risk in %	RD [95% CI] in %	NNT	HR [95% CI]
PP	Discontinuation<br>SGLT2i: 24.68<br>GLP-1RA: 45.72<br><br>Crossover<br>SGLT2i: 4.68<br>GLP-1RA: 9.06	SL			Unadjusted	4.79	3.37	1.42 [0.09, 2.76]	70	1.47 [0.97, 1.97]
			14.71	2,350.47	TMLE untruncated	6.26	3.23	3.03 [1.96, 4.11]	33	
					TMLE truncated at 200	6.26	3.30	2.96 [1.88, 4.04]	34	
			2.81	253.37	IPW untruncated	6.26	3.19	3.07 [0.38, 5.76]	33	1.38 [0.69, 2.07]
					IPW truncated at 20	6.27	3.27	3.00 [0.30, 5.70]	33	1.36 [0.68, 2.03]
					IPW truncated at 99th	5.87	3.28	2.59 [0.47, 4.70]	39	1.42 [0.79, 2.06]
		Logistic model	4.41	106.47	IPW untruncated	6.20	3.35	2.85 [-0.21, 5.90]		1.40 [0.65, 2.14]
					IPW truncated at 20	6.20	3.39	2.82 [-0.24, 5.88]		1.38 [0.65, 2.11]
					IPW truncated at 99th	6.01	3.48	2.53 [-0.14, 5.20]		1.39 [0.71, 2.06]
ITT		SL			Unadjusted	5.02	3.96	1.06 [0.08, 2.05]	94	1.31 [0.94, 1.69]
			9.81	1,426.42	TMLE untruncated	5.66	3.97	1.69 [0.85, 2.53]	59	
					TMLE truncated at 200	5.66	4.02	1.64 [0.80, 2.48]	61	
			2.70	276.63	IPW untruncated	5.66	3.95	1.70 [0.03, 3.37]	59	1.23 [0.72, 1.75]
					IPW truncated at 20	5.66	4.04	1.63 [-0.05, 3.30]		1.21 [0.71, 1.72]
					IPW truncated at 99th	5.29	4.07	1.22 [-0.08, 2.53]		1.20 [0.76, 1.64]
		Logistic model	4.06	103.04	IPW untruncated	5.30	3.88	1.42 [-0.24, 3.09]		1.20 [0.69, 1.72]
					IPW truncated at 20	5.31	3.94	1.37 [-0.30, 3.04]		1.19 [0.68, 1.69]
					IPW truncated at 99th	5.23	4.10	1.12 [-0.37, 2.62]		1.18 [0.71, 1.66]

* Discontinuation refers to the interruption of the comparator medication initiated on index date; Crossover refers to the initiation of the comparator medication initiated by patient at baseline in the other arm.

© 2025 Neugebauer R et al. *JAMA Network Open*.

eFigure 48. MACE (Primary Definition), 2-Arm Drug Class Comparison, SGLT2is vs GLP-1RAs, High CKD Risk Subgroup, Cumulative Incidence Curves From PP and ITT Analyses With IPW, TMLE, and SL
Each plot emulates inferences among patients with High CKD Risk from a 2-arm RCT comparing SGLT2i and GLP-1RA and represents unadjusted or adjusted estimates of cumulative incidence curves for MACE derived with IPW and TMLE with SL estimates of propensity scores with four weight truncation schemes: IPW and TMLE without weight truncation (untruncated), IPW with truncation of stabilized weights at value 20 (trunc20) or at the 99th percentile of weight values (trunc99), and TMLE with truncation of unstabilized weights at value 200 (trunc200). The red divider line separates results of Per-Protocol (PP) analyses (top half) from Intention-To-Treat (ITT) analyses (bottom half). Each plot displays a p value for the test that the average risk difference (ARD) through 2.5 years of follow-up (30 months) is 0.

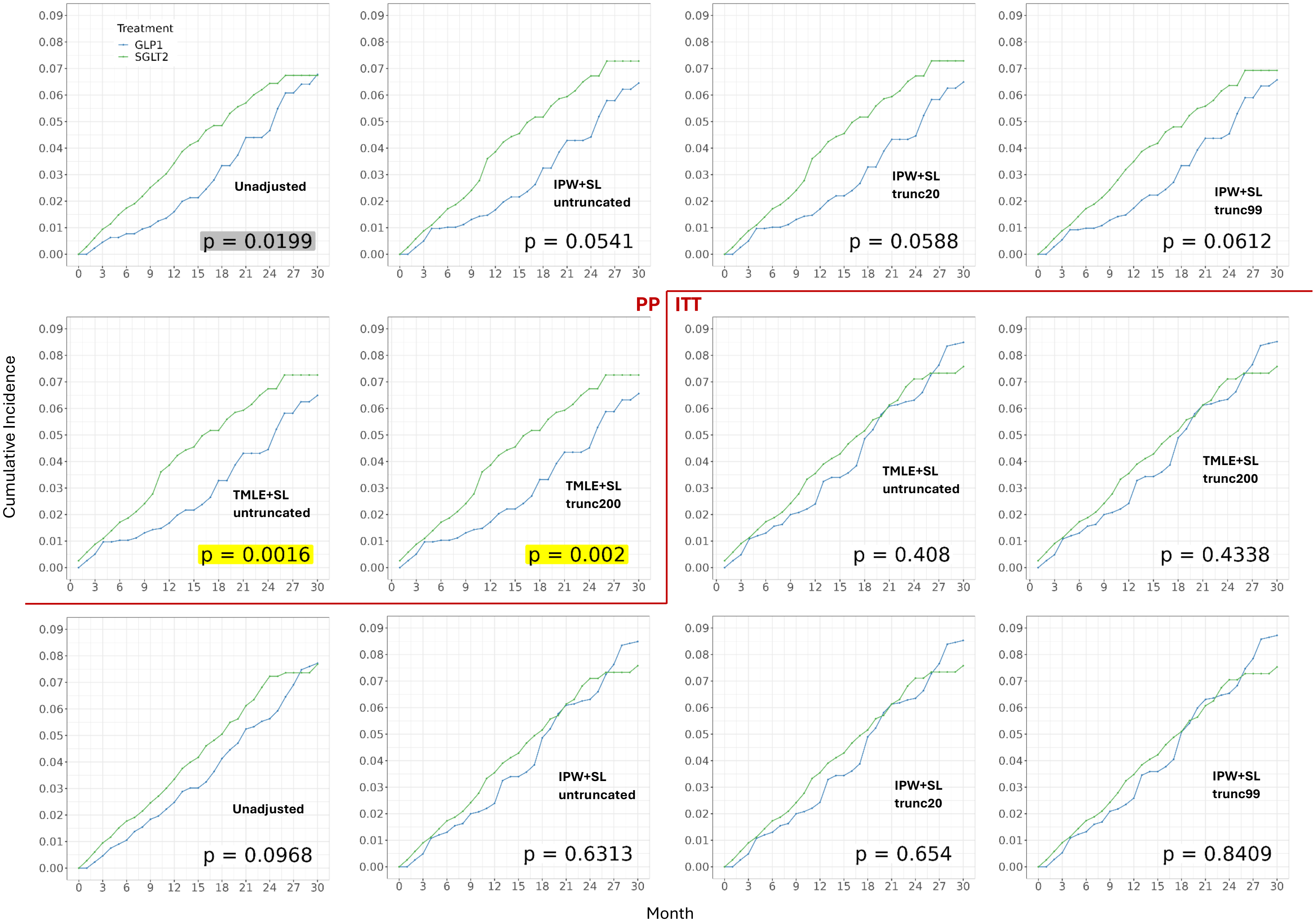

© 2025 Neugebauer R et al. *JAMA Network Open*.

eTable 47. MACE (Primary Definition), 2-Arm Drug Class Comparison, SGLT2is vs GLP-1RAs, High CKD Risk Subgroup, RD and HR Effect Measures at 2.5 Years

Estimation results among patients with High CKD risk from ITT and PP analyses of emulated 2-arm RCTs comparing MACE risks over 2.5 years between SGLT2i and GLP-1RA initiators. For PP analyses, rates of protocol deviations are described by medication class initiated at baseline. Unadjusted point and interval estimates and adjusted point and interval IPW and TMLE estimates of risks, risk differences (RD), and hazard ratios (HR) based on propensity scores (PS) estimated with either logistic models or super learning (SL) are presented for four weight truncation schemes along with the corresponding 99th percentile and maximum value of the stabilized and unstabilized inverse probability weights used for implementing IPW and TMLE, respectively. RD is the risk in treatment arm minus the risk in control arm and NNT is the number needed to treat.

Analysis type	Protocol Deviations* by exposure group (%)	PS estimation	99th IP weights	Max IP weight	Estimator	Treatment (SGLT2i) risk in %	Control (GLP-1RA) risk in %	RD [95% CI] in %	NNT	HR [95% CI]
PP	Discontinuation<br>SGLT2i: 19.88<br>GLP-1RA: 45.24<br><br>Crossover<br>SGLT2i: 3.55<br>GLP-1RA: 9.34	SL			Unadjusted	6.74	6.78	-0.03 [-2.66, 2.59]		2.29 [1.22, 3.36]
			17.83	1,112.37	TMLE untruncated	7.26	6.49	0.77 [-0.52, 2.06]		
					TMLE truncated at 200	7.26	6.56	0.70 [-0.58, 1.99]		
			3.04	87.32	IPW untruncated	7.28	6.45	0.83 [-2.34, 4.00]		2.04 [0.56, 3.51]
					IPW truncated at 20	7.29	6.49	0.79 [-2.38, 3.97]		2.04 [0.56, 3.51]
					IPW truncated at 99th	6.93	6.57	0.36 [-2.58, 3.31]		1.94 [0.69, 3.19]
		Logistic model	4.79	120.34	IPW untruncated	6.56	4.41	2.15 [-1.08, 5.38]		1.79 [0.29, 3.29]
					IPW truncated at 20	6.70	5.08	1.62 [-1.45, 4.69]		1.78 [0.29, 3.26]
					IPW truncated at 99th	7.16	5.69	1.47 [-1.60, 4.54]		1.80 [0.40, 3.20]
ITT		SL			Unadjusted	7.68	7.72	-0.04 [-1.95, 1.88]		1.41 [0.93, 1.89]
			12.86	698.63	TMLE untruncated	7.58	8.49	-0.91 [-2.00, 0.18]		
					TMLE truncated at 200	7.58	8.52	-0.94 [-2.03, 0.15]		
			2.91	100.14	IPW untruncated	7.58	8.49	-0.92 [-3.50, 1.67]		1.44 [0.75, 2.12]
					IPW truncated at 20	7.58	8.53	-0.95 [-3.54, 1.64]		1.43 [0.75, 2.11]
					IPW truncated at 99th	7.53	8.72	-1.19 [-3.76, 1.37]		1.33 [0.74, 1.93]
		Logistic model	4.47	318.99	IPW untruncated	8.33	9.21	-0.88 [-4.74, 2.98]		1.74 [0.51, 2.97]
					IPW truncated at 20	7.92	9.32	-1.40 [-4.85, 2.06]		1.51 [0.58, 2.44]
					IPW truncated at 99th	7.65	9.57	-1.92 [-5.17, 1.33]		1.33 [0.62, 2.05]

* Discontinuation refers to the interruption of the comparator medication initiated on index date; Crossover refers to the initiation of the comparator medication initiated by patient at baseline in the other arm.

© 2025 Neugebauer R et al. *JAMA Network Open*.

eFigure 49. MACE (Primary Definition), 2-Arm Drug Class Comparison, SGLT2is vs GLP-1RAs, Male Subgroup, Cumulative Incidence Curves From PP and ITT Analyses With IPW, TMLE, and SL
Each plot emulates inferences among Male patients from a 2-arm RCT comparing SGLT2i and GLP-1RA and represents unadjusted or adjusted estimates of cumulative incidence curves for MACE derived with IPW and TMLE with SL estimates of propensity scores with four weight truncation schemes: IPW and TMLE without weight truncation (untruncated), IPW with truncation of stabilized weights at value 20 (trunc20) or at the 99th percentile of weight values (trunc99), and TMLE with truncation of unstabilized weights at value 200 (trunc200). The red divider line separates results of Per-Protocol (PP) analyses (top half) from Intention-To-Treat (ITT) analyses (bottom half). Each plot displays a p value for the test that the average risk difference (ARD) through 2.5 years of follow-up (30 months) is 0.

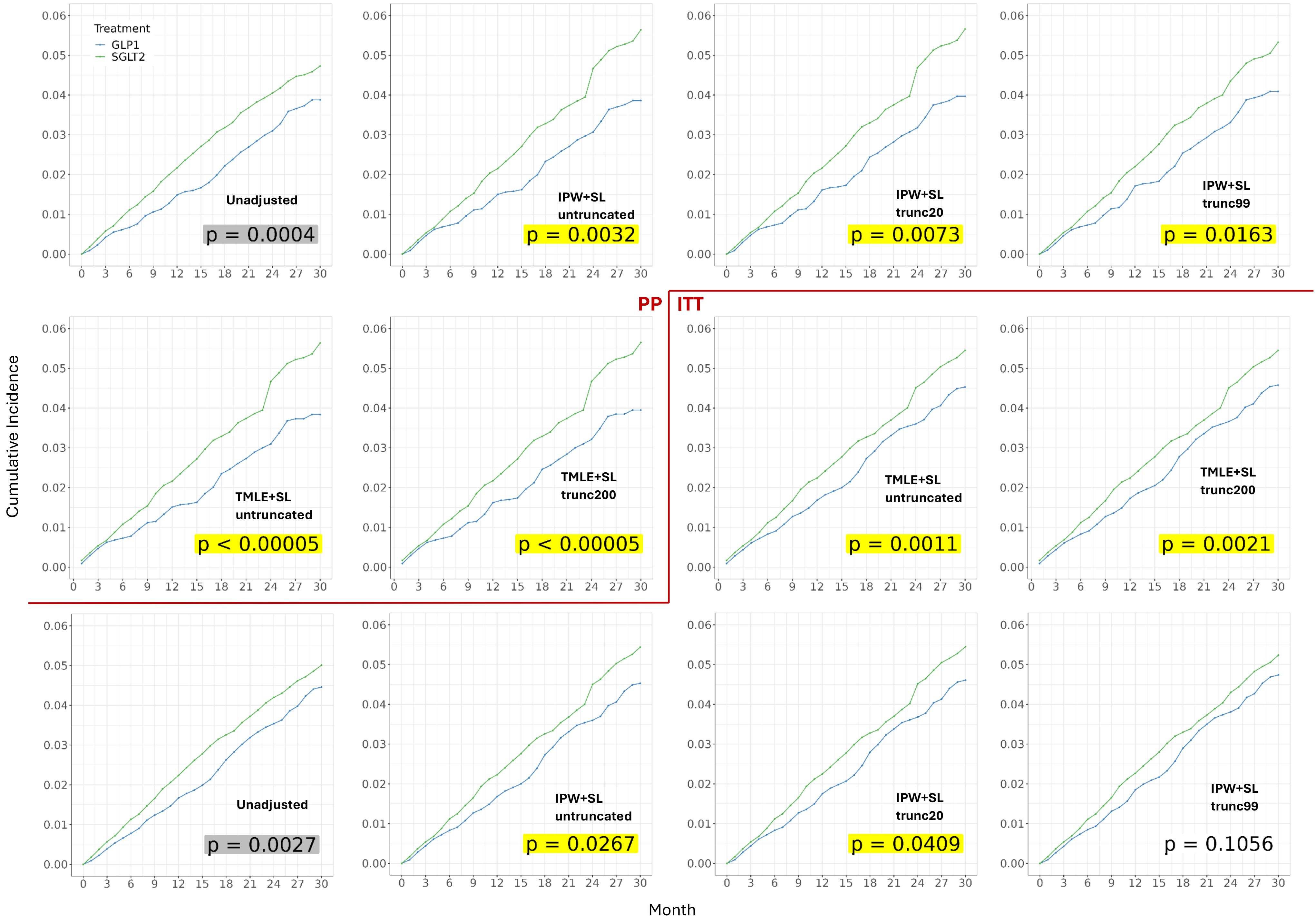

© 2025 Neugebauer R et al. *JAMA Network Open*.

eTable 48. MACE (Primary Definition), 2-Arm Drug Class Comparison, SGLT2is vs GLP-1RAs, Male Subgroup, RD and HR Effect Measures at 2.5 Years

Estimation results among Male patients from ITT and PP analyses of emulated 2-arm RCTs comparing MACE risks over 2.5 years between SGLT2i and GLP-1RA initiators. For PP analyses, rates of protocol deviations are described by medication class initiated at baseline. Unadjusted point and interval estimates and adjusted point and interval IPW and TMLE estimates of risks, risk differences (RD), and hazard ratios (HR) based on propensity scores (PS) estimated with either logistic models or super learning (SL) are presented for four weight truncation schemes along with the corresponding 99th percentile and maximum value of the stabilized and unstabilized inverse probability weights used for implementing IPW and TMLE, respectively. RD is the risk in treatment arm minus the risk in control arm and NNT is the number needed to treat.

Analysis type	Protocol Deviations* by exposure group (%)	PS estimation	99th IP weights	Max IP weight	Estimator	Treatment (SGLT2i) risk in %	Control (GLP-1RA) risk in %	RD [95% CI] in %	NNT	HR [95% CI]
PP	Discontinuation<br>SGLT2i: 23.01<br>GLP-1RA: 44.05<br><br>Crossover<br>SGLT2i: 4.21<br>GLP-1RA: 11.73	SL			Unadjusted	4.73	3.88	0.85 [-0.05, 1.76]		1.59 [1.24, 1.94]
			16.33	3,453.99	TMLE untruncated	5.64	3.84	1.81 [1.07, 2.54]	55	
					TMLE truncated at 200	5.65	3.95	1.69 [0.96, 2.43]	59	
			2.87	347.32	IPW untruncated	5.64	3.86	1.78 [0.14, 3.42]	56	1.49 [1.03, 1.95]
					IPW truncated at 20	5.66	3.97	1.69 [0.04, 3.35]	59	1.46 [1.02, 1.91]
					IPW truncated at 99th	5.33	4.09	1.24 [-0.02, 2.49]	81	1.45 [1.02, 1.87]
		Logistic model	4.32	348.76	IPW untruncated	5.50	3.97	1.53 [-0.11, 3.17]		1.43 [0.93, 1.92]
					IPW truncated at 20	5.57	4.13	1.43 [-0.26, 3.12]		1.39 [0.91, 1.86]
					IPW truncated at 99th	5.49	4.27	1.23 [-0.27, 2.72]		1.38 [0.94, 1.82]
ITT		SL			Unadjusted	5.01	4.46	0.55 [-0.13, 1.24]		1.41 [1.16, 1.66]
			11.12	2,117.64	TMLE untruncated	5.45	4.53	0.91 [0.35, 1.48]	109	
					TMLE truncated at 200	5.45	4.58	0.86 [0.30, 1.43]	116	
			2.80	378.21	IPW untruncated	5.44	4.53	0.90 [-0.23, 2.03]		1.36 [1.03, 1.70]
					IPW truncated at 20	5.45	4.61	0.85 [-0.29, 1.99]		1.35 [1.02, 1.68]
					IPW truncated at 99th	5.24	4.74	0.50 [-0.41, 1.42]		1.31 [1.00, 1.62]
		Logistic model	4.06	427.56	IPW untruncated	5.40	4.58	0.82 [-0.41, 2.04]		1.32 [0.95, 1.68]
					IPW truncated at 20	5.44	4.69	0.75 [-0.49, 2.00]		1.28 [0.93, 1.63]
					IPW truncated at 99th	5.34	4.83	0.51 [-0.58, 1.60]		1.26 [0.94, 1.58]

* Discontinuation refers to the interruption of the comparator medication initiated on index date; Crossover refers to the initiation of the comparator medication initiated by patient at baseline in the other arm.

© 2025 Neugebauer R et al. *JAMA Network Open*.

eFigure 50. MACE (Primary Definition), 2-Arm Drug Class Comparison, SGLT2is vs GLP-1RAs, Female Subgroup, Cumulative Incidence Curves From PP and ITT Analyses With IPW, TMLE, and SL
Each plot emulates inferences among Female patients from a 2-arm RCT comparing SGLT2i and GLP-1RA and represents unadjusted or adjusted estimates of cumulative incidence curves for MACE derived with IPW and TMLE with SL estimates of propensity scores with four weight truncation schemes: IPW and TMLE without weight truncation (untruncated), IPW with truncation of stabilized weights at value 20 (trunc20) or at the 99th percentile of weight values (trunc99), and TMLE with truncation of unstabilized weights at value 200 (trunc200). The red divider line separates results of Per-Protocol (PP) analyses (top half) from Intention-To-Treat (ITT) analyses (bottom half). Each plot displays a p value for the test that the average risk difference (ARD) through 2.5 years of follow-up (30 months) is 0.

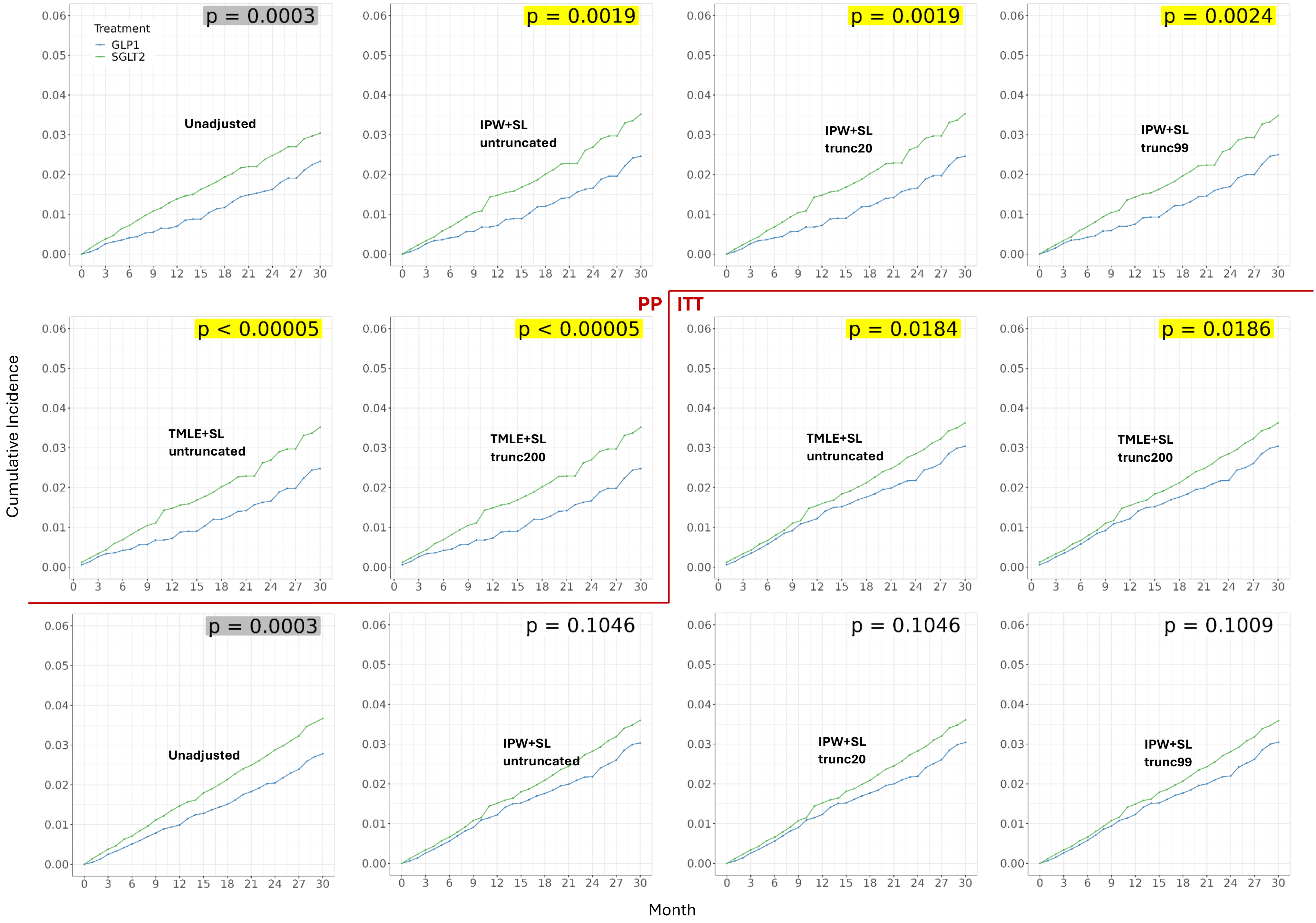

© 2025 Neugebauer R et al. *JAMA Network Open*.

eTable 49. MACE (Primary Definition), 2-Arm Drug Class Comparison, SGLT2is vs GLP-1RAs, Female Subgroup, RD and HR Effect Measures at 2.5 Years

Estimation results among Female patients from ITT and PP analyses of emulated 2-arm RCTs comparing MACE risks over 2.5 years between SGLT2i and GLP-1RA initiators. For PP analyses, rates of protocol deviations are described by medication class initiated at baseline. Unadjusted point and interval estimates and adjusted point and interval IPW and TMLE estimates of risks, risk differences (RD), and hazard ratios (HR) based on propensity scores (PS) estimated with either logistic models or super learning (SL) are presented for four weight truncation schemes along with the corresponding 99th percentile and maximum value of the stabilized and unstabilized inverse probability weights used for implementing IPW and TMLE, respectively. RD is the risk in treatment arm minus the risk in control arm and NNT is the number needed to treat.

Analysis type	Protocol Deviations* by exposure group (%)	PS estimation	99th IP weights	Max IP weight	Estimator	Treatment (SGLT2i) risk in %	Control (GLP-1RA) risk in %	RD [95% CI] in %	NNT	HR [95% CI]
PP	Discontinuation<br>SGLT2i: 29.03<br>GLP-1RA: 45.16<br><br>Crossover<br>SGLT2i: 6.09<br>GLP-1RA: 8.18	SL	[redacted]		Unadjusted	3.04	2.33	0.71 [-0.09, 1.52]		1.78 [1.26, 2.3]
			15.90	806.53	TMLE untruncated	3.52	2.48	1.04 [0.53, 1.55]	96	[redacted]
					TMLE truncated at 200	3.52	2.48	1.04 [0.53, 1.55]	96	
			3.17	154.87	IPW untruncated	3.52	2.46	1.06 [-0.07, 2.18]		1.75 [1.15, 2.35]
					IPW truncated at 20	3.53	2.46	1.06 [-0.07, 2.19]		1.75 [1.15, 2.36]
					IPW truncated at 99th	3.48	2.50	0.98 [-0.13, 2.09]		1.67 [1.11, 2.22]
		Logistic model	4.69	360.43	IPW untruncated	3.36	2.48	0.87 [-0.34, 2.09]		1.74 [1.12, 2.37]
					IPW truncated at 20	3.42	2.50	0.92 [-0.31, 2.14]		1.75 [1.12, 2.38]
					IPW truncated at 99th	3.62	2.57	1.04 [-0.23, 2.32]		1.70 [1.10, 2.31]
ITT	[redacted]	SL	[redacted]		Unadjusted	3.67	2.78	0.89 [0.30, 1.48]	112	1.39 [1.07, 1.71]
			9.46	495.56	TMLE untruncated	3.63	3.04	0.59 [0.17, 1.02]	169	[redacted]
					TMLE truncated at 200	3.63	3.04	0.59 [0.17, 1.01]	169	
			2.93	117.39	IPW untruncated	3.60	3.03	0.57 [-0.17, 1.31]		1.17 [0.82, 1.51]
					IPW truncated at 20	3.61	3.04	0.57 [-0.17, 1.31]		1.17 [0.83, 1.52]
					IPW truncated at 99th	3.59	3.05	0.54 [-0.17, 1.25]		1.15 [0.84, 1.46]
		Logistic model	4.27	1,196.71	IPW untruncated	3.63	3.03	0.59 [-0.45, 1.63]		1.03 [0.52, 1.54]
					IPW truncated at 20	3.65	2.96	0.68 [-0.24, 1.60]		1.10 [0.64, 1.56]
					IPW truncated at 99th	3.65	2.87	0.78 [0.01, 1.55]	128	1.17 [0.82, 1.51]

* Discontinuation refers to the interruption of the comparator medication initiated on index date; Crossover refers to the initiation of the comparator medication initiated by patient at baseline in the other arm.

© 2025 Neugebauer R et al. *JAMA Network Open*.

eFigure 51. MACE (Primary Definition), 2-Arm Drug Class Comparison, SGLT2is vs GLP-1RAs, Age <50 Subgroup, Cumulative Incidence Curves From PP and ITT Analyses With IPW, TMLE, and SL
Each plot emulates inferences among patients with Age <50 from a 2-arm RCT comparing SGLT2i and GLP-1RA and represents unadjusted or adjusted estimates of cumulative incidence curves for MACE derived with IPW and TMLE with SL estimates of propensity scores with four weight truncation schemes: IPW and TMLE without weight truncation (untruncated), IPW with truncation of stabilized weights at value 20 (trunc20) or at the 99th percentile of weight values (trunc99), and TMLE with truncation of unstabilized weights at value 200 (trunc200). The red divider line separates results of Per-Protocol (PP) analyses (top half) from Intention-To-Treat (ITT) analyses (bottom half). Each plot displays a p value for the test that the average risk difference (ARD) through 2.5 years of follow-up (30 months) is 0.

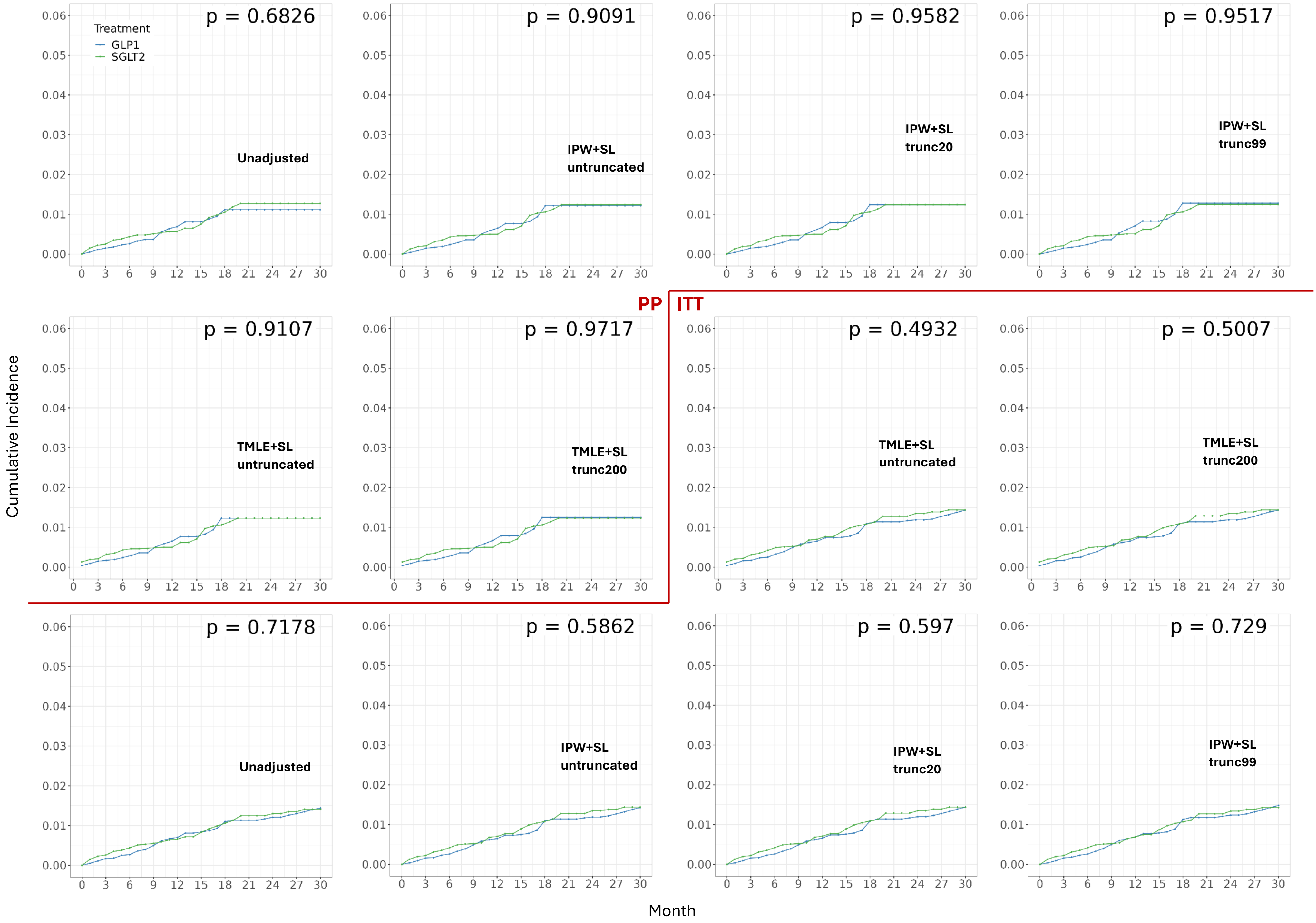

© 2025 Neugebauer R et al. *JAMA Network Open*.

eTable 50. MACE (Primary Definition), 2-Arm Drug Class Comparison, SGLT2is vs GLP-1RAs, Age <50 Subgroup, RD and HR Effect Measures at 2.5 Years

Estimation results among patients Age <50 from ITT and PP analyses of emulated 2-arm RCTs comparing MACE risks over 2.5 years between SGLT2i and GLP-1RA initiators. For PP analyses, rates of protocol deviations are described by medication class initiated at baseline. Unadjusted point and interval estimates and adjusted point and interval IPW and TMLE estimates of risks, risk differences (RD), and hazard ratios (HR) based on propensity scores (PS) estimated with either logistic models or super learning (SL) are presented for four weight truncation schemes along with the corresponding 99th percentile and maximum value of the stabilized and unstabilized inverse probability weights used for implementing IPW and TMLE, respectively. RD is the risk in treatment arm minus the risk in control arm and NNT is the number needed to treat.

Analysis type	Protocol Deviations* by exposure group (%)	PS estimation	99th IP weights	Max IP weight	Estimator	Treatment (SGLT2i) risk in %	Control (GLP-1RA) risk in %	RD [95% CI] in %	NNT	HR [95% CI]
PP	Discontinuation SGLT2i: 28.45 GLP-1RA: 47.25 Crossover SGLT2i: 6.94 GLP-1RA: 9.73	SL			Unadjusted	1.27	1.12	0.15 [-0.49, 0.79]		1.06 [0.51, 1.6]
			16.35	2,317.91	TMLE untruncated	1.23	1.23	-0.00 [-0.46, 0.47]		
					TMLE truncated at 200	1.23	1.25	-0.01 [-0.48, 0.45]		
			3.16	317.49	IPW untruncated	1.24	1.22	0.02 [-0.75, 0.79]		1.10 [0.49, 1.71]
					IPW truncated at 20	1.24	1.24	0.00 [-0.78, 0.78]		1.10 [0.49, 1.71]
					IPW truncated at 99th	1.25	1.28	-0.04 [-0.83, 0.75]		1.08 [0.48, 1.68]
		Logistic model	4.39	244.94	IPW untruncated	0.95	1.15	-0.20 [-0.87, 0.46]		1.00 [0.43, 1.56]
					IPW truncated at 20	0.95	1.17	-0.21 [-0.89, 0.46]		1.00 [0.43, 1.56]
					IPW truncated at 99th	0.98	1.21	-0.23 [-0.92, 0.45]		1.00 [0.43, 1.56]
ITT		SL			Unadjusted	1.41	1.44	-0.03 [-0.59, 0.53]		0.98 [0.56, 1.41]
			9.64	1,480.08	TMLE untruncated	1.44	1.43	0.01 [-0.42, 0.45]		
					TMLE truncated at 200	1.44	1.43	0.01 [-0.42, 0.44]		
			3.09	399.48	IPW untruncated	1.44	1.43	0.01 [-0.63, 0.64]		1.14 [0.54, 1.73]
					IPW truncated at 20	1.44	1.44	0.00 [-0.63, 0.64]		1.13 [0.54, 1.73]
					IPW truncated at 99th	1.43	1.48	-0.04 [-0.68, 0.59]		1.08 [0.54, 1.62]
		Logistic model	4.05	81.47	IPW untruncated	1.19	1.39	-0.21 [-0.78, 0.37]		0.97 [0.49, 1.46]
					IPW truncated at 20	1.19	1.40	-0.21 [-0.79, 0.37]		0.97 [0.49, 1.46]
					IPW truncated at 99th	1.23	1.45	-0.22 [-0.82, 0.37]		0.97 [0.49, 1.45]

* Discontinuation refers to the interruption of the comparator medication initiated on index date; Crossover refers to the initiation of the comparator medication initiated by patient at baseline in the other arm.

© 2025 Neugebauer R et al. *JAMA Network Open*.

eFigure 52. MACE (Primary Definition), 2-Arm Drug Class Comparison, SGLT2is vs GLP-1RAs, Age 50 to <65 Subgroup, Cumulative Incidence Curves From PP and ITT Analyses With IPW, TMLE, and SL
Each plot emulates inferences among patients with AGE 50 to <65 from a 2-arm RCT comparing SGLT2i and GLP-1RA and represents unadjusted or adjusted estimates of cumulative incidence curves for MACE derived with IPW and TMLE with SL estimates of propensity scores with four weight truncation schemes: IPW and TMLE without weight truncation (untruncated), IPW with truncation of stabilized weights at value 20 (trunc20) or at the 99th percentile of weight values (trunc99), and TMLE with truncation of unstabilized weights at value 200 (trunc200). The red divider line separates results of Per-Protocol (PP) analyses (top half) from Intention-To-Treat (ITT) analyses (bottom half). Each plot displays a p value for the test that the average risk difference (ARD) through 2.5 years of follow-up (30 months) is 0.

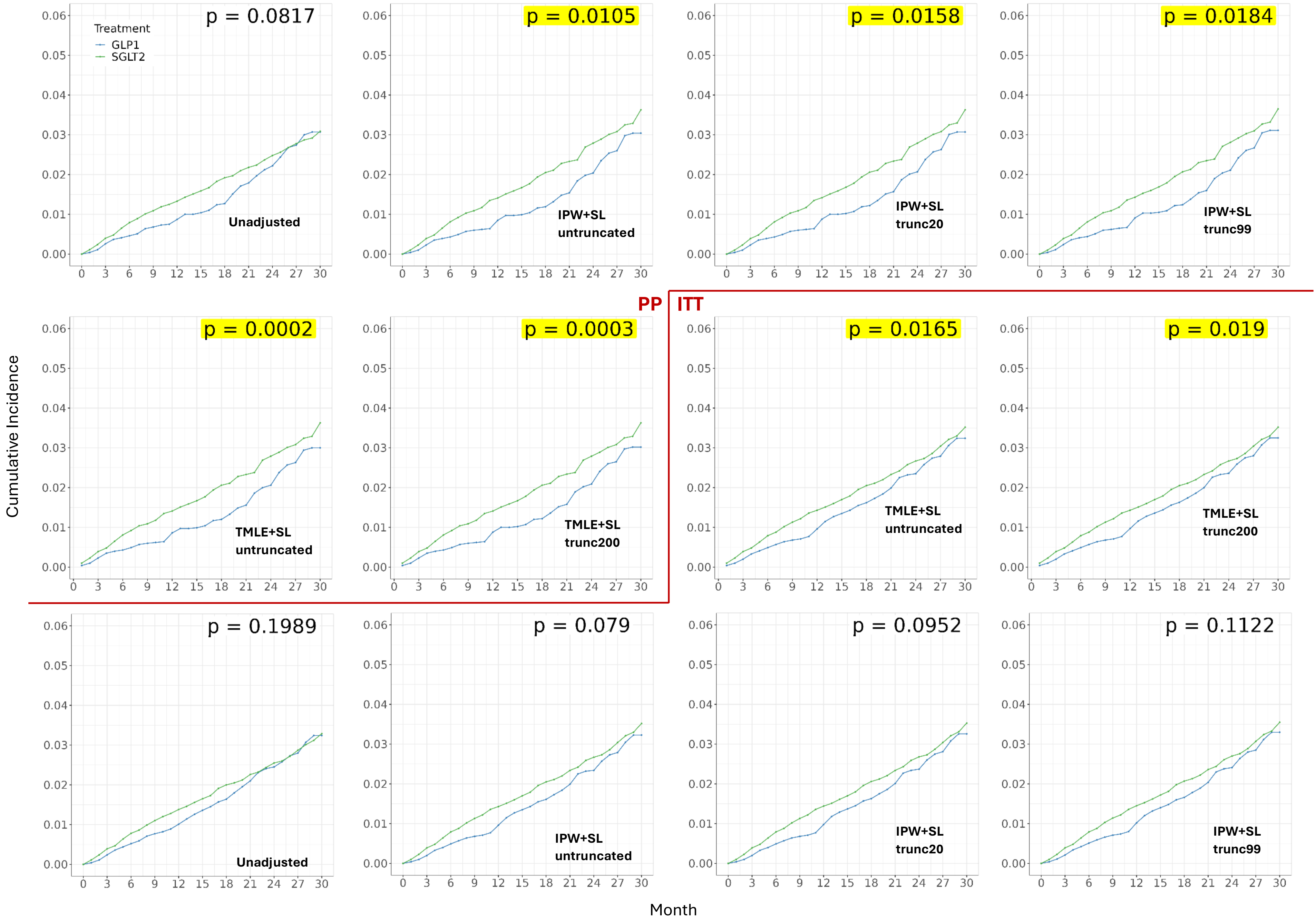

© 2025 Neugebauer R et al. *JAMA Network Open*.

eTable 51. MACE (Primary Definition), 2-Arm Drug Class Comparison, SGLT2is vs GLP-1RAs, Age 50 to <65 Subgroup, RD and HR Effect Measures at 2.5 Years

Estimation results among patients Age 50 to <65 from ITT and PP analyses of emulated 2-arm RCTs comparing MACE risks over 2.5 years between SGLT2i and GLP-1RA initiators. For PP analyses, rates of protocol deviations are described by medication class initiated at baseline. Unadjusted point and interval estimates and adjusted point and interval IPW and TMLE estimates of risks, risk differences (RD), and hazard ratios (HR) based on propensity scores (PS) estimated with either logistic models or super learning (SL) are presented for four weight truncation schemes along with the corresponding 99th percentile and maximum value of the stabilized and unstabilized inverse probability weights used for implementing IPW and TMLE, respectively. RD is the risk in treatment arm minus the risk in control arm and NNT is the number needed to treat.

Analysis type	Protocol Deviations* by exposure group (%)	PS estimation	99th IP weights	Max IP weight	Estimator	Treatment (SGLT2i) risk in %	Control (GLP-1RA) risk in %	RD [95% CI] in %	NNT	HR [95% CI]
PP	Discontinuation<br>SGLT2i: 25.46<br>GLP-1RA: 43.88<br><br>Crossover<br>SGLT2i: 5.68<br>GLP-1RA: 10.66	SL			Unadjusted	3.09	3.07	0.01 [-0.82, 0.85]		1.59 [1.14, 2.04]
			14.85	996.59	TMLE untruncated	3.63	3.00	0.63 [0.13, 1.13]	159	
					TMLE truncated at 200	3.63	3.02	0.60 [0.10, 1.10]	165	
			2.90	125.51	IPW untruncated	3.63	3.04	0.59 [-0.51, 1.68]		1.77 [1.17, 2.38]
					IPW truncated at 20	3.63	3.07	0.56 [-0.54, 1.67]		1.77 [1.17, 2.37]
					IPW truncated at 99th	3.65	3.11	0.54 [-0.56, 1.65]		1.73 [1.15, 2.30]
		Logistic model	4.43	358.64	IPW untruncated	3.87	3.22	0.65 [-0.98, 2.27]		1.75 [0.93, 2.57]
					IPW truncated at 20	3.86	3.30	0.57 [-1.04, 2.17]		1.66 [0.94, 2.38]
					IPW truncated at 99th	3.87	3.33	0.54 [-1.01, 2.10]		1.55 [0.98, 2.13]
ITT		SL			Unadjusted	3.29	3.24	0.05 [-0.56, 0.65]		1.27 [0.99, 1.56]
			9.57	650.98	TMLE untruncated	3.52	3.24	0.29 [-0.11, 0.68]		
					TMLE truncated at 200	3.52	3.25	0.28 [-0.12, 0.67]		
			2.74	141.88	IPW untruncated	3.52	3.23	0.29 [-0.45, 1.03]		1.41 [1.02, 1.79]
					IPW truncated at 20	3.53	3.26	0.27 [-0.47, 1.02]		1.40 [1.02, 1.79]
					IPW truncated at 99th	3.55	3.30	0.26 [-0.48, 0.99]		1.38 [1.01, 1.74]
		Logistic model	4.10	1,260.57	IPW untruncated	3.52	3.29	0.23 [-0.71, 1.16]		1.43 [0.93, 1.94]
					IPW truncated at 20	3.50	3.34	0.16 [-0.75, 1.07]		1.39 [0.93, 1.85]
					IPW truncated at 99th	3.44	3.40	0.03 [-0.80, 0.87]		1.26 [0.90, 1.63]

* Discontinuation refers to the interruption of the comparator medication initiated on index date; Crossover refers to the initiation of the comparator medication initiated by patient at baseline in the other arm.

© 2025 Neugebauer R et al. *JAMA Network Open*.

eFigure 53. MACE (Primary Definition), 2-Arm Drug Class Comparison, SGLT2is vs GLP-1RAs, Age ≥65 Subgroup, Cumulative Incidence Curves From PP and ITT Analyses With IPW, TMLE, and SL
Each plot emulates inferences among patients with AGE 65+ from a 2-arm RCT comparing SGLT2i and GLP-1RA and represents unadjusted or adjusted estimates of cumulative incidence curves for MACE derived with IPW and TMLE with SL estimates of propensity scores with four weight truncation schemes: IPW and TMLE without weight truncation (untruncated), IPW with truncation of stabilized weights at value 20 (trunc20) or at the 99th percentile of weight values (trunc99), and TMLE with truncation of unstabilized weights at value 200 (trunc200). The red divider line separates results of Per-Protocol (PP) analyses (top half) from Intention-To-Treat (ITT) analyses (bottom half). Each plot displays a p value for the test that the average risk difference (ARD) through 2.5 years of follow-up (30 months) is 0.

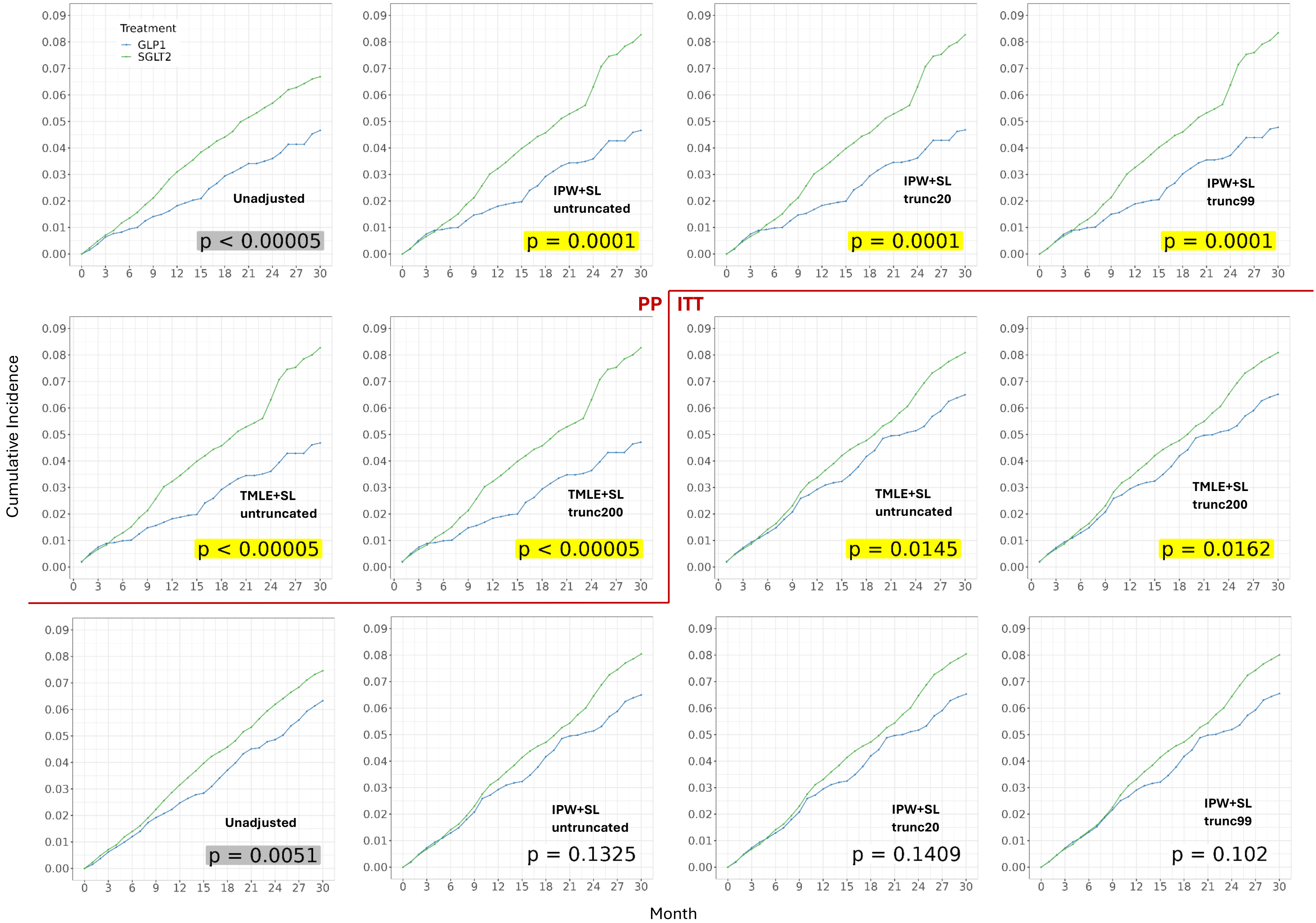

© 2025 Neugebauer R et al. *JAMA Network Open*.

eTable 52. MACE (Primary Definition), 2-Arm Drug Class Comparison, SGLT2is vs GLP-1RAs, Age ≥65 Subgroup, RD and HR Effect Measures at 2.5 Years

Estimation results among patients Age 65+ from ITT and PP analyses of emulated 2-arm RCTs comparing MACE risks over 2.5 years between SGLT2i and GLP-1RA initiators. For PP analyses, rates of protocol deviations are described by medication class initiated at baseline. Unadjusted point and interval estimates and adjusted point and interval IPW and TM LE estimates of risks, risk differences (RD), and hazard ratios (HR) based on propensity scores (PS) estimated with either logistic models or super learning (SL) are presented for four weight truncation schemes along with the corresponding 99th percentile and maximum value of the stabilized and unstabilized inverse probability weights used for implementing IPW and TMLE, respectively. RD is the risk in treatment arm minus the risk in control arm and NNT is the number needed to treat.

Analysis type	Protocol Deviations* by exposure group (%)	PS estimation	99th IP weights	Max IP weight	Estimator	Treatment (SGLT2i) risk in %	Control (GLP-1RA) risk in %	RD [95% CI] in %	NNT	HR [95% CI]
PP	Discontinuation<br>SGLT2i: 24.46<br>GLP-1RA: 43.38<br><br>Crossover<br>SGLT2i: 3.39<br>GLP-1RA: 8.33	SL			Unadjusted	6.69	4.66	2.03 [0.66, 3.39]	49	1.61 [1.19, 2.04]
			16.94	1,288.21	TMLE untruncated	8.27	4.68	3.59 [2.79, 4.39]	28	
					TMLE truncated at 200	8.27	4.71	3.56 [2.76, 4.36]	28	
			2.84	109.32	IPW untruncated	8.27	4.66	3.61 [1.48, 5.74]	28	1.50 [0.92, 2.09]
					IPW truncated at 20	8.27	4.68	3.59 [1.45, 5.72]	28	1.49 [0.91, 2.07]
					IPW truncated at 99th	8.34	4.78	3.56 [1.39, 5.72]	28	1.49 [0.96, 2.03]
		Logistic model	4.36	279.03	IPW untruncated	9.85	4.55	5.29 [1.68, 8.90]	19	1.63 [0.74, 2.53]
					IPW truncated at 20	9.60	4.65	4.95 [1.45, 8.44]	20	1.48 [0.74, 2.21]
					IPW truncated at 99th	8.96	4.70	4.25 [1.56, 6.95]	24	1.46 [0.86, 2.06]
ITT		SL			Unadjusted	7.46	6.33	1.13 [0.04, 2.23]	88	1.28 [1.02, 1.54]
			12.03	832.92	TMLE untruncated	8.09	6.50	1.59 [0.80, 2.39]	63	
					TMLE truncated at 200	8.09	6.52	1.57 [0.78, 2.36]	64	
			2.71	125.05	IPW untruncated	8.04	6.50	1.53 [-0.04, 3.11]		1.13 [0.78, 1.48]
					IPW truncated at 20	8.04	6.53	1.51 [-0.07, 3.09]		1.12 [0.78, 1.47]
					IPW truncated at 99th	8.01	6.55	1.46 [-0.00, 2.92]	69	1.13 [0.82, 1.43]
		Logistic model	4.28	342.69	IPW untruncated	9.22	6.66	2.56 [-0.26, 5.38]		1.24 [0.59, 1.89]
					IPW truncated at 20	8.85	6.77	2.08 [-0.51, 4.67]		1.10 [0.62, 1.58]
					IPW truncated at 99th	8.21	6.59	1.62 [-0.20, 3.43]		1.10 [0.76, 1.44]

* Discontinuation refers to the interruption of the comparator medication initiated on index date; Crossover refers to the initiation of the comparator medication initiated by patient at baseline in the other arm.

© 2025 Neugebauer R et al. *JAMA Network Open*.

eFigure 54. MACE (Primary Definition), 2-Arm Drug Class Comparison, SGLT2is vs GLP-1RAs, Asian Subgroup, Cumulative Incidence Curves From PP and ITT Analyses With IPW, TMLE, and SL
Each plot emulates inferences among patients in Race SUBGRP Asian from a 2-arm RCT comparing SGLT2i and GLP-1RA and represents unadjusted or adjusted estimates of cumulative incidence curves for MACE derived with IPW and TMLE with SL estimates of propensity scores with four weight truncation schemes: IPW and TMLE without weight truncation (untruncated), IPW with truncation of stabilized weights at value 20 (trunc20) or at the 99th percentile of weight values (trunc99), and TMLE with truncation of unstabilized weights at value 200 (trunc200). The red divi der line separates results of Per-Protocol (PP) analyses (top half) from Intention-To-Treat (ITT) analyses (bottom half). Each plot displays a p value for the test that the average risk difference (ARD) through 2.5 years of follow-up (30 months) is 0.

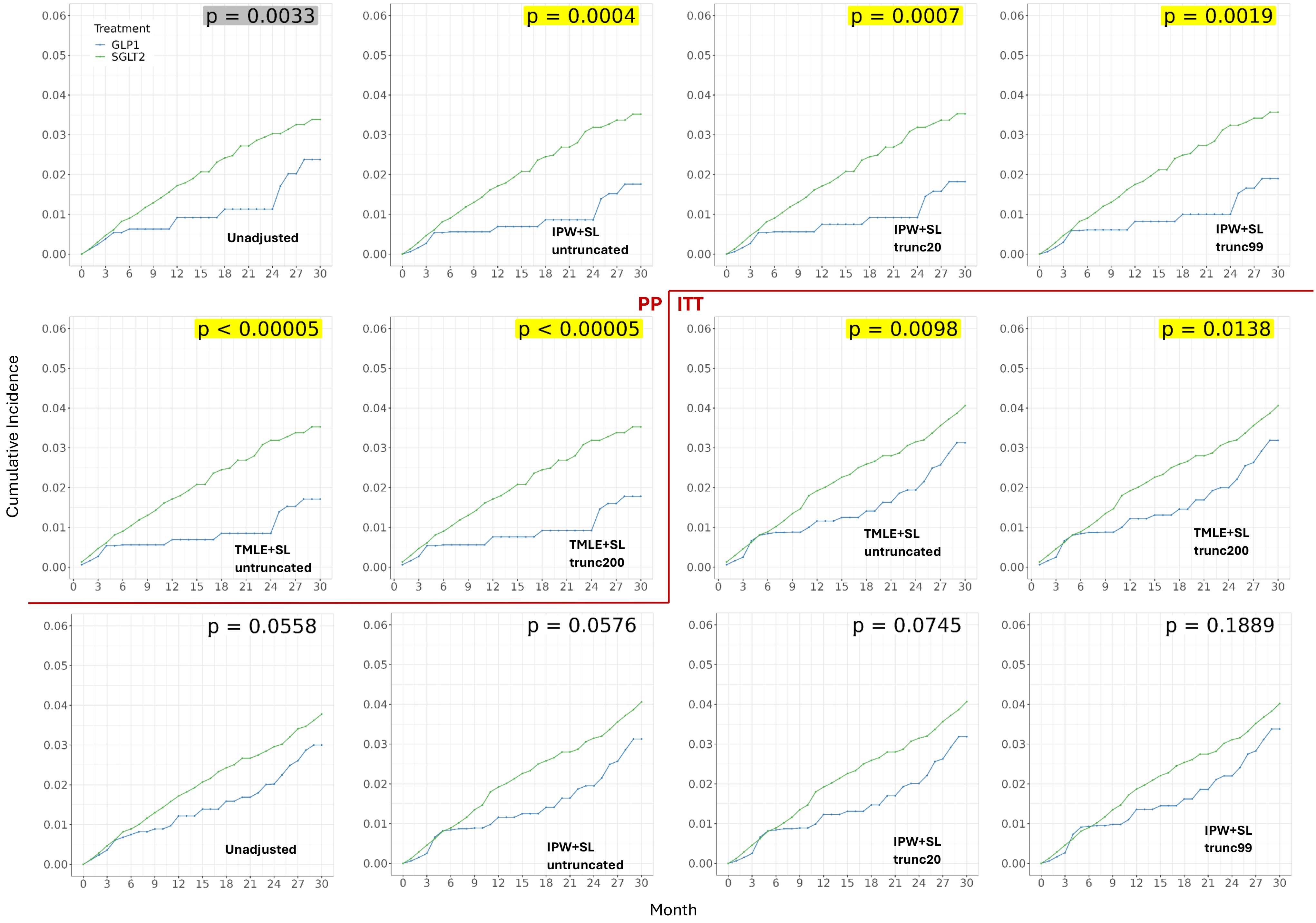

© 2025 Neugebauer R et al. *JAMA Network Open*.

eTable 53. MACE (Primary Definition), 2-Arm Drug Class Comparison, SGLT2is vs GLP-1RAs, Asian Subgroup, RD and HR Effect Measures at 2.5 Years

Estimation results among Asian patients from ITT and PP analyses of emulated 2-arm RCTs comparing MACE risks over 2.5 years between SGLT2i and GLP-1RA initiators. For PP analyses, rates of protocol deviations are described by medication class initiated at baseline. Unadjusted point and interval estimates and adjusted point and interval IPW and TMLE estimates of risks, risk differences (RD), and hazard ratios (HR) based on propensity scores (PS) estimated with either logistic models or super learning (SL) are presented for four weight truncation schemes along with the corresponding 99th percentile and maximum value of the stabilized and unstabilized inverse probability weights used for implementing IPW and TMLE, respectively. RD is the risk in treatment arm minus the risk in control arm and NNT is the number needed to treat.

Analysis type	Protocol Deviations* by exposure group (%)	PS estimation	99th IP weights	Max IP weight	Estimator	Treatment (SGLT2i) risk in %	Control (GLP-1RA) risk in %	RD [95% CI] in %	NNT	HR [95% CI]
PP	<u>Discontinuation</u><br>SGLT2i: 20.12<br>GLP-1RA: 42.43<br><br><u>Crossover</u><br>SGLT2i: 2.30<br>GLP-1RA: 12.10	SL			Unadjusted	3.39	2.38	1.01 [-0.59, 2.61]		1.87 [0.71, 3.03]
			22.34	2,927.70	TMLE untruncated	3.53	1.71	1.81 [1.15, 2.47]	55	
					TMLE truncated at 200	3.53	1.78	1.75 [1.09, 2.40]	57	
			2.65	179.67	IPW untruncated	3.52	1.76	1.77 [0.31, 3.22]	57	2.21 [0.31, 4.11]
					IPW truncated at 20	3.53	1.82	1.71 [0.25, 3.17]	58	2.17 [0.31, 4.03]
					IPW truncated at 99th	3.57	1.90	1.67 [0.18, 3.17]	60	1.99 [0.29, 3.69]
		Logistic model	3.77	113.45	IPW untruncated	3.61	1.77	1.84 [0.12, 3.57]	54	2.33 [0.58, 4.08]
					IPW truncated at 20	3.62	1.80	1.82 [0.08, 3.56]	55	2.27 [0.57, 3.97]
					IPW truncated at 99th	3.69	1.88	1.80 [0.04, 3.57]	55	2.07 [0.52, 3.62]
ITT		SL			Unadjusted	3.78	3.00	0.78 [-0.47, 2.03]		1.54 [0.77, 2.31]
			17.09	1,889.73	TMLE untruncated	4.06	3.13	0.94 [0.14, 1.73]	107	
					TMLE truncated at 200	4.06	3.19	0.88 [0.09, 1.66]	114	
			2.65	206.44	IPW untruncated	4.06	3.13	0.93 [-0.69, 2.56]		1.63 [0.57, 2.70]
					IPW truncated at 20	4.07	3.19	0.88 [-0.75, 2.51]		1.60 [0.56, 2.65]
					IPW truncated at 99th	4.02	3.38	0.64 [-1.02, 2.30]		1.42 [0.51, 2.33]
		Logistic model	3.80	153.29	IPW untruncated	3.84	4.33	-0.49 [-3.16, 2.17]		1.24 [0.34, 2.15]
					IPW truncated at 20	3.85	4.50	-0.65 [-3.41, 2.11]		1.20 [0.33, 2.07]
					IPW truncated at 99th	3.91	4.71	-0.80 [-3.64, 2.04]		1.13 [0.33, 1.93]

* Discontinuation refers to the interruption of the comparator medication initiated on index date; Crossover refers to the initiation of the comparator medication initiated by patient at baseline in the other arm.

© 2025 Neugebauer R et al. *JAMA Network Open*.

eFigure 55. MACE (Primary Definition), 2-Arm Drug Class Comparison, SGLT2is vs GLP-1RAs, Black Subgroup, Cumulative Incidence Curves From PP and ITT Analyses With IPW, TMLE, and SL
Each plot emulates inferences among patients in Race SUBGRP Black/African-American from a 2-arm RCT comparing SGLT2i and GLP-1RA and represents unadjusted or adjusted estimates of cumulative incidence curves for MACE derived with IPW and TMLE with SL estimates of propensity scores with four weight truncation schemes: IPW and TMLE without weight truncation (untruncated), IPW with truncation of stabilized weights at value 20 (trunc20) or at the 99th percentile of weight values (trunc99), and TMLE with truncation of unstabilized weights at value 200 (trunc200). The red divider line separates results of Per-Protocol (PP) analyses (top half) from Intention-To-Treat (ITT) analyses (bottom half). Each plot displays a p value for the test that the average risk difference (ARD) through 2.5 years of follow-up (30 months) is 0.

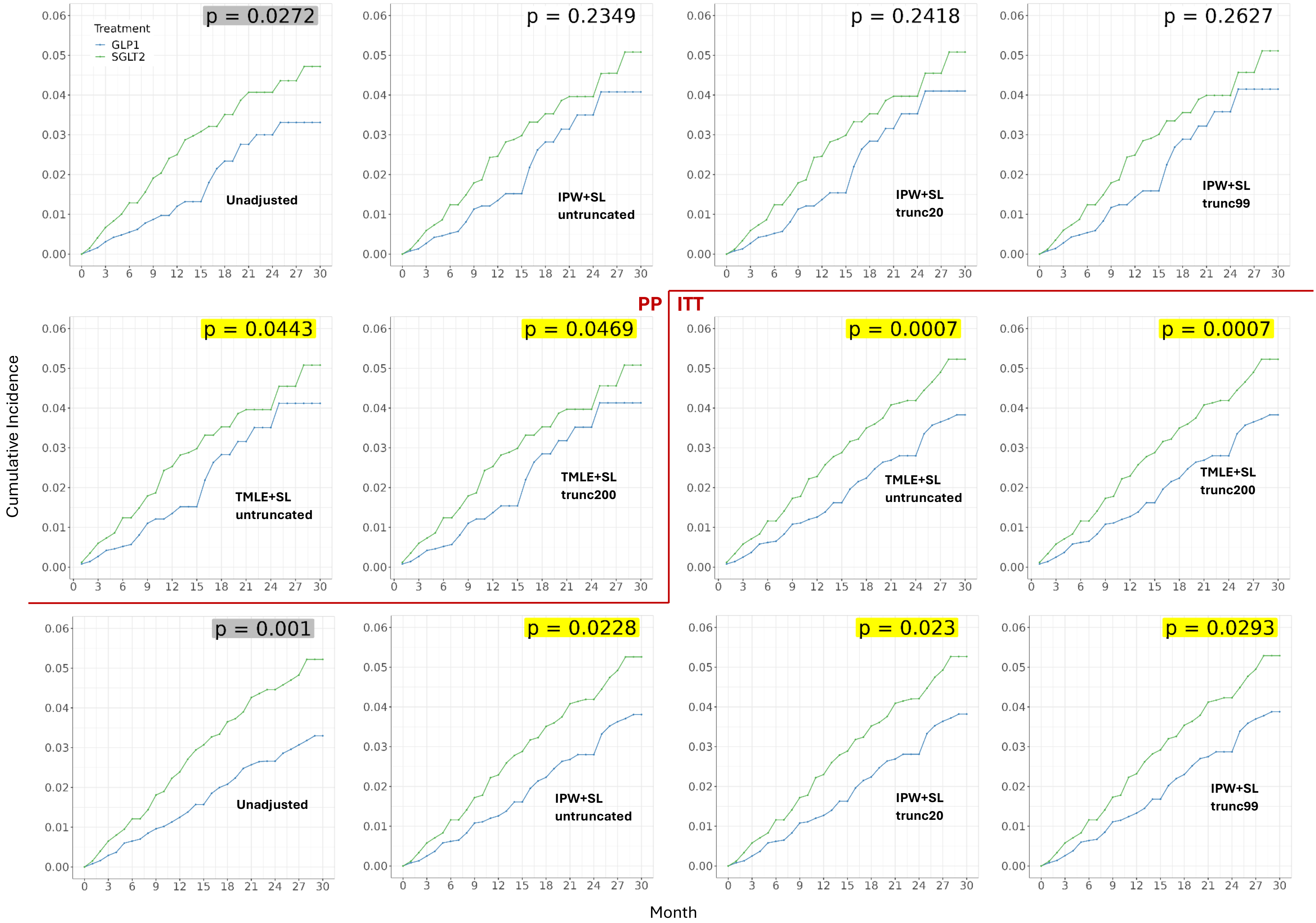

© 2025 Neugebauer R et al. *JAMA Network Open*.

eTable 54. MACE (Primary Definition), 2-Arm Drug Class Comparison, SGLT2is vs GLP-1RAs, Black Subgroup, RD and HR Effect Measures at 2.5 Years

Estimation results among Black patients from ITT and PP analyses of emulated 2-arm RCTs comparing MACE risks over 2.5 years between SGLT2i and GLP-1RA initiators. For PP analyses, rates of protocol deviations are described by medication class initiated at baseline. Unadjusted point and interval estimates and adjusted point and interval IPW and TMLE estimates of risks, risk differences (RD), and hazard ratios (HR) based on propensity scores (PS) estimated with either logistic models or super learning (SL) are presented for four weight truncation schemes along with the corresponding 99th percentile and maximum value of the stabilized and unstabilized inverse probability weights used for implementing IPW and TMLE, respectively. RD is the risk in treatment arm minus the risk in control arm and NNT is the number needed to treat.

Analysis type	Protocol Deviations* by exposure group (%)	PS estimation	99th IP weights	Max IP weight	Estimator	Treatment (SGLT2i) risk in %	Control (GLP-1RA) risk in %	RD [95% CI] in %	NNT	HR [95% CI]
PP	Discontinuation<br>SGLT2i: 27.86<br>GLP-1RA: 45.96<br><br>Crossover<br>SGLT2i: 5.44<br>GLP-1RA: 6.86	SL			Unadjusted	4.72	3.31	1.41 [-0.60, 3.41]		2.14 [1.02, 3.27]
			13.35	570.78	TMLE untruncated	5.08	4.12	0.96 [-0.22, 2.14]		
					TMLE truncated at 200	5.08	4.13	0.95 [-0.23, 2.12]		
			2.79	98.53	IPW untruncated	5.08	4.08	1.00 [-1.81, 3.80]		1.89 [0.74, 3.03]
					IPW truncated at 20	5.08	4.10	0.98 [-1.83, 3.79]		1.89 [0.75, 3.04]
					IPW truncated at 99th	5.11	4.15	0.95 [-1.86, 3.77]		1.85 [0.73, 2.98]
		Logistic model	4.82	73.79	IPW untruncated	4.29	3.80	0.50 [-1.98, 2.98]		1.61 [0.54, 2.69]
					IPW truncated at 20	4.33	3.80	0.53 [-1.95, 3.01]		1.63 [0.55, 2.72]
					IPW truncated at 99th	4.48	3.86	0.62 [-1.89, 3.13]		1.62 [0.54, 2.69]
ITT		SL			Unadjusted	5.22	3.30	1.93 [0.47, 3.38]	52	1.91 [1.05, 2.78]
			8.13	282.74	TMLE untruncated	5.23	3.83	1.40 [0.51, 2.30]	71	
					TMLE truncated at 200	5.23	3.83	1.40 [0.51, 2.30]	71	
			2.64	79.62	IPW untruncated	5.26	3.81	1.45 [-0.42, 3.32]		1.80 [0.87, 2.73]
					IPW truncated at 20	5.27	3.82	1.45 [-0.42, 3.32]		1.80 [0.87, 2.73]
					IPW truncated at 99th	5.29	3.88	1.41 [-0.47, 3.29]		1.76 [0.85, 2.66]
		Logistic model	4.64	79.44	IPW untruncated	4.50	3.51	0.99 [-0.72, 2.70]		1.61 [0.71, 2.50]
					IPW truncated at 20	4.54	3.52	1.02 [-0.69, 2.72]		1.61 [0.72, 2.51]
					IPW truncated at 99th	4.68	3.63	1.06 [-0.68, 2.79]		1.57 [0.71, 2.44]

* Discontinuation refers to the interruption of the comparator medication initiated on index date; Crossover refers to the initiation of the comparator medication initiated by patient at baseline in the other arm.

© 2025 Neugebauer R et al. *JAMA Network Open*.

eFigure 56. MACE (Primary Definition), 2-Arm Drug Class Comparison, SGLT2is vs GLP-1RAs, Native Hawaiian or Other Pacific Islander Subgroup, Cumulative Incidence Curves From PP and ITT Analyses With IPW, TMLE, and SL

Each plot emulates inferences among patients in Race SUBGRP Hawaiian/Pacific Islander from a 2-arm RCT comparing SGLT2i and GLP-1RA and represents unadjusted or adjusted estimates of cumulative incidence curves for MACE derived with IPW and TMLE with SL estimates of propensity scores with four weight truncation schemes: IPW and TMLE without weight truncation (untruncated), IPW with truncation of stabilized weights at value 20 (trunc20) or at the 99th percentile of weight values (trunc99), and TMLE with truncation of unstabilized weights at value 200 (trunc200). The red divider line separates results of Per-Protocol (PP) analyses (top half) from Intention-To-Treat (ITT) analyses (bottom half). Each plot displays a p value for the test that the average risk difference (ARD) through 2.5 years of follow-up (30 months) is 0.

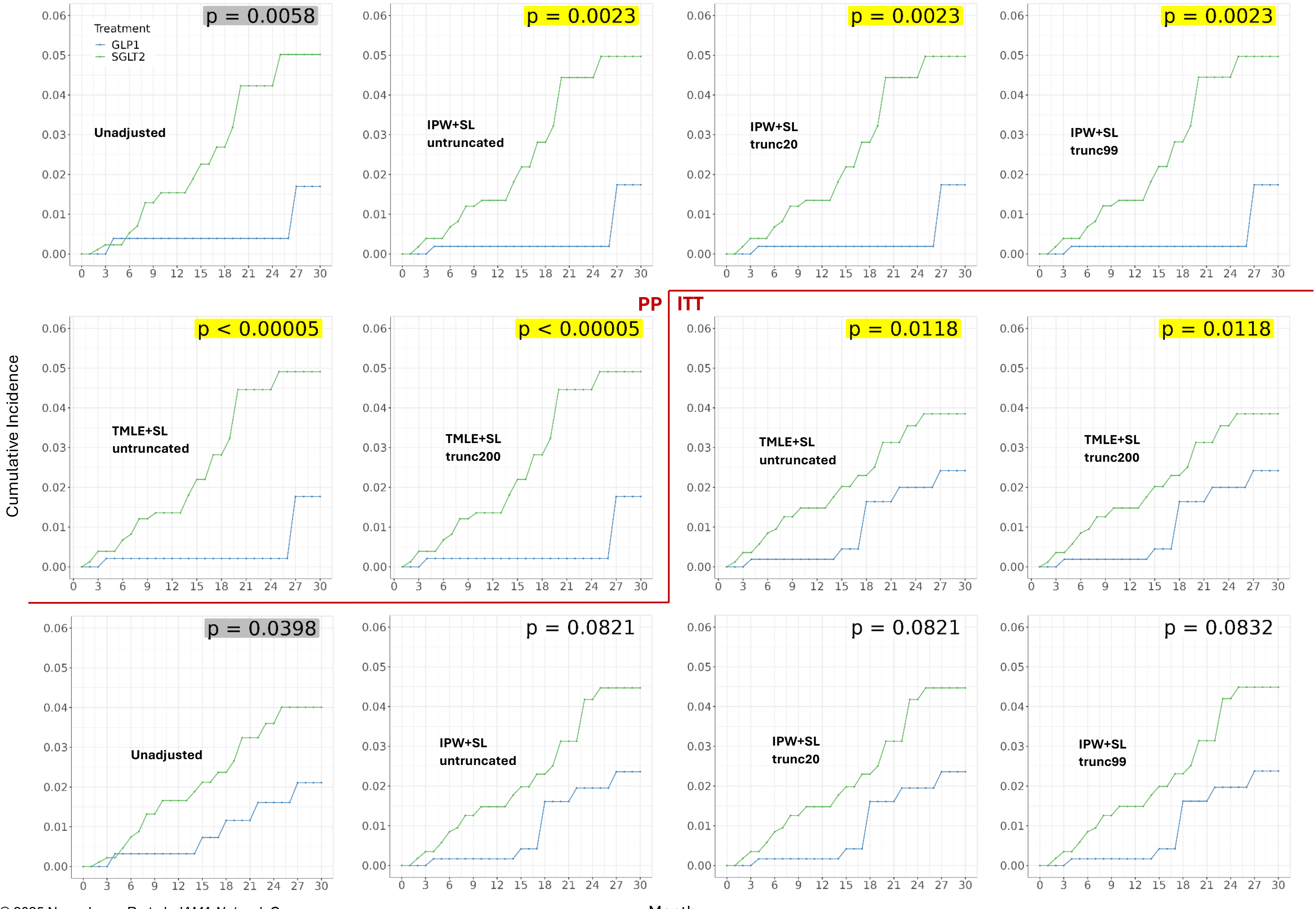

© 2025 Neugebauer R et al. *JAMA Network Open*.

eTable 55. MACE (Primary Definition), 2-Arm Drug Class Comparison, SGLT2is vs GLP-1RAs, Native Hawaiian or Other Pacific Islander Subgroup, RD and HR Effect Measures at 2.5 Years

Estimation results among Hawaiian Pacific Islander patients from ITT and PP analyses of emulated 2-arm RCTs comparing MACE risks over 2.5 years between SGLT2i and GLP-1RA initiators. For PP analyses, rates of protocol deviations are described by medication class initiated at baseline. Unadjusted point and interval estimates and adjusted point and interval IPW and TMLE estimates of risks, risk differences (RD), and hazard ratios (HR) based on propensity scores (PS) estimated with either logistic models or super learning (SL) are presented for four weight truncation schemes along with the corresponding 99th percentile and maximum value of the stabilized and unstabilized inverse probability weights used for implementing IPW and TMLE, respectively. RD is the risk in treatment arm minus the risk in control arm and NNT is the number needed to treat.

Analysis type	Protocol Deviations* by exposure group (%)	PS estimation	99th IP weights	Max IP weight	Estimator	Treatment (SGLT2i) risk in %	Control (GLP-1RA) risk in %	RD [95% CI] in %	NNT	HR [95% CI]
PP	<u>Discontinuation</u><br>SGLT2i: 29.13<br>GLP-1RA: 50.15<br><br><u>Crossover</u><br>SGLT2i: 3.02<br>GLP-1RA: 11.87	SL			Unadjusted	5.02	1.70	3.32 [-0.55, 7.19]		4.20 [-4.49, 12.90]
			11.49	282.15	TMLE untruncated	4.91	1.77	3.15 [1.55, 4.74]	32	
					TMLE truncated at 200	4.91	1.77	3.15 [1.55, 4.74]	32	
			2.17	72.26	IPW untruncated	4.97	1.74	3.22 [-0.94, 7.39]		7.45 [-7.99, 22.88]
					IPW truncated at 20	4.97	1.74	3.22 [-0.94, 7.39]		7.55 [-8.09, 23.18]
					IPW truncated at 99th	4.97	1.74	3.23 [-0.94, 7.39]		7.43 [-7.96, 22.82]
		Logistic model	2.23	10.05	IPW untruncated	4.70	1.66	3.04 [-0.81, 6.89]		5.59 [-6.00, 17.18]
					IPW truncated at 20	4.70	1.66	3.04 [-0.81, 6.89]		5.59 [-6.00, 17.18]
					IPW truncated at 99th	4.70	1.66	3.04 [-0.81, 6.89]		5.48 [-5.88, 16.84]
ITT		SL			Unadjusted	4.01	2.11	1.90 [-0.74, 4.53]		3.04 [-1.45, 7.52]
			8.51	225.87	TMLE untruncated	3.85	2.42	1.44 [-0.24, 3.12]		
					TMLE truncated at 200	3.85	2.42	1.44 [-0.24, 3.12]		
			2.22	87.72	IPW untruncated	4.47	2.36	2.11 [-1.58, 5.79]		5.33 [-2.64, 13.31]
					IPW truncated at 20	4.47	2.36	2.11 [-1.58, 5.79]		5.40 [-2.67, 13.46]
					IPW truncated at 99th	4.49	2.38	2.11 [-1.60, 5.82]		5.31 [-2.63, 13.26]
		Logistic model	2.28	14.25	IPW untruncated	3.79	2.04	1.75 [-0.98, 4.48]		4.64 [-2.38, 11.67]
					IPW truncated at 20	3.79	2.04	1.75 [-0.98, 4.48]		4.64 [-2.38, 11.67]
					IPW truncated at 99th	3.80	2.04	1.75 [-0.98, 4.48]		4.55 [-2.33, 11.42]

* Discontinuation refers to the interruption of the comparator medication initiated on index date; Crossover refers to the initiation of the comparator medication initiated by patient at baseline in the other arm.

© 2025 Neugebauer R et al. *JAMA Network Open*.

eFigure 57. MACE (Primary Definition), 2-Arm Drug Class Comparison, SGLT2is vs GLP-1RAs, Multiple Race Subgroup, Cumulative Incidence Curves From PP and ITT Analyses With IPW, TMLE, and SL
Each plot emulates inferences among patients in Race SUBGRP Multiracial from a 2-arm RCT comparing SGLT2i and GLP-1RA and represents unadjusted or adjusted estimates of cumulative incidence curves for MACE derived with IPW and TMLE with SL estimates of propensity scores with four weight truncation schemes: IPW and TMLE without weight truncation (untruncated), IPW with truncation of stabilized weights at value 20 (trunc20) or at the 99th percentile of weight values (trunc99), and TMLE with truncation of unstabilized weights at value 200 (trunc200). The red divi der line separates results of Per-Protocol (PP) analyses (top half) from Intention-To-Treat (ITT) analyses (bottom half). Each plot displays a p value for the test that the average risk difference (ARD) through 2.5 years of follow-up (30 months) is 0.

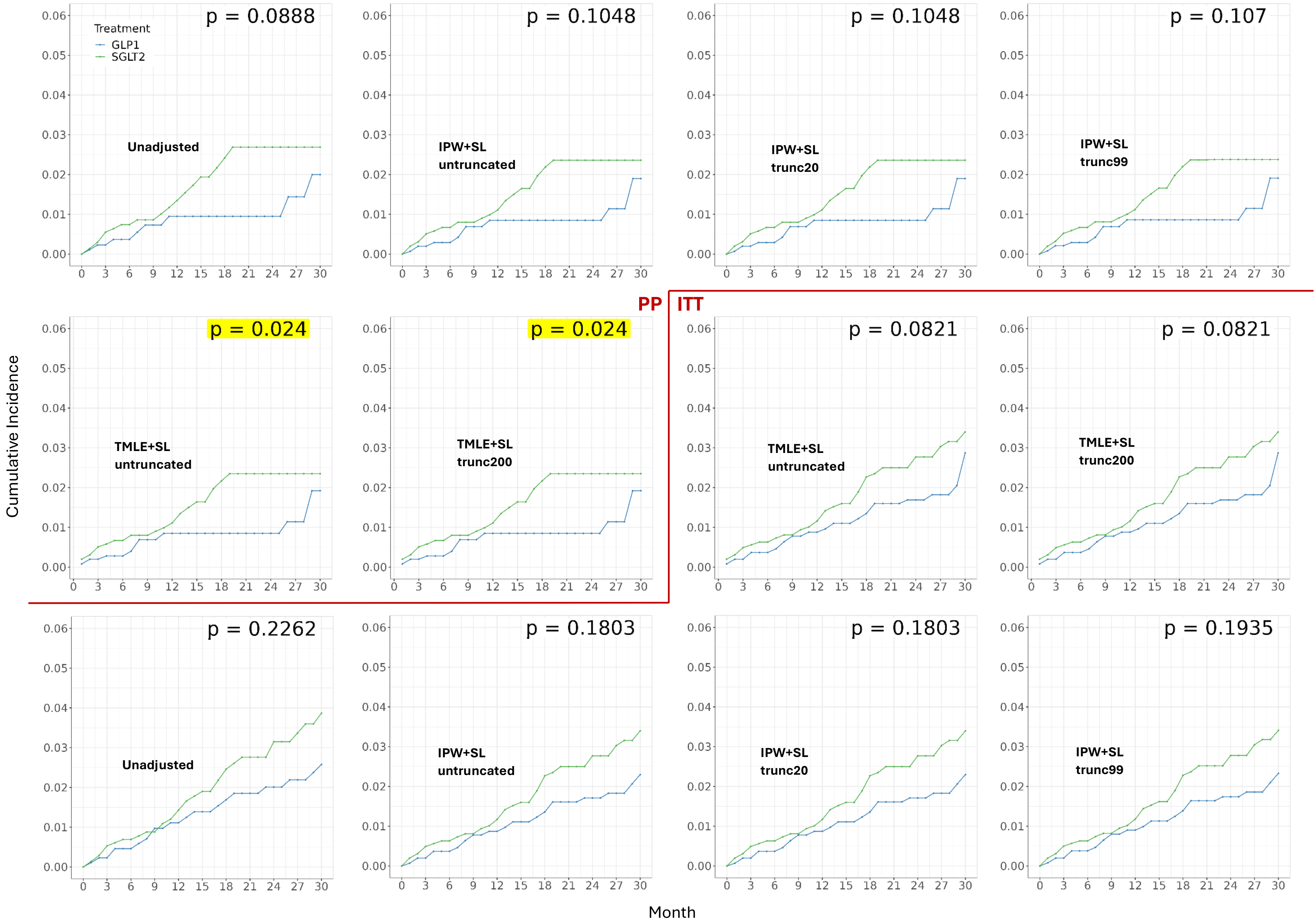

© 2025 Neugebauer R et al. *JAMA Network Open*.

eTable 56. MACE (Primary Definition), 2-Arm Drug Class Comparison, SGLT2is vs GLP-1RAs, Multiple Race Subgroup, RD and HR Effect Measures at 2.5 Years

Estimation results among Multiracial patients from ITT and PP analyses of emulated 2-arm RCTs comparing MACE risks over 2.5 years between SGLT2i and GLP-1RA initiators. For PP analyses, rates of protocol deviations are described by medication class initiated at baseline. Unadjusted point and interval estimates and adjusted point and interval IPW and TMLE estimates of risks, risk differences (RD), and hazard ratios (HR) based on propensity scores (PS) estimated with either logistic models or super learning (SL) are presented for four weight truncation schemes along with the corresponding 99th percentile and maximum value of the stabilized and unstabilized inverse probability weights used for implementing IPW and TMLE, respectively. RD is the risk in treatment arm minus the risk in control arm and NNT is the number needed to treat.

Analysis type	Protocol Deviations* by exposure group (%)	PS estimation	99th IP weights	Max IP weight	Estimator	Treatment (SGLT2i) risk in %	Control (GLP-1RA) risk in %	RD [95% CI] in %	NNT	HR [95% CI]
PP	Discontinuation<br>SGLT2i: 31.64<br>GLP-1RA: 49.28<br><br>Crossover<br>SGLT2i: 5.21<br>GLP-1RA: 13.62	SL			Unadjusted	2.69	2.00	0.69 [-1.42, 2.79]		2.18 [0.16, 4.20]
			10.21	230.87	TMLE untruncated	2.35	1.92	0.44 [-0.69, 1.57]		
					TMLE truncated at 200	2.35	1.92	0.44 [-0.69, 1.57]		
			2.55	42.34	IPW untruncated	2.36	1.90	0.46 [-1.68, 2.60]		2.01 [0.02, 4.00]
					IPW truncated at 20	2.36	1.90	0.46 [-1.68, 2.60]		2.00 [0.02, 3.98]
					IPW truncated at 99th	2.38	1.91	0.46 [-1.68, 2.60]		2.00 [0.02, 3.97]
		Logistic model	2.58	21.08	IPW untruncated	2.33	1.89	0.45 [-1.75, 2.64]		2.25 [0.06, 4.44]
					IPW truncated at 20	2.33	1.89	0.45 [-1.75, 2.64]		2.25 [0.06, 4.44]
					IPW truncated at 99th	2.35	1.91	0.44 [-1.76, 2.64]		2.23 [0.06, 4.39]
ITT		SL			Unadjusted	3.87	2.58	1.29 [-0.59, 3.16]		1.39 [0.48, 2.31]
			7.18	146.74	TMLE untruncated	3.40	2.87	0.53 [-0.67, 1.73]		
					TMLE truncated at 200	3.40	2.87	0.53 [-0.67, 1.73]		
			2.64	46.43	IPW untruncated	3.40	2.30	1.10 [-0.66, 2.85]		1.45 [0.44, 2.45]
					IPW truncated at 20	3.40	2.30	1.10 [-0.66, 2.85]		1.44 [0.44, 2.45]
					IPW truncated at 99th	3.41	2.33	1.08 [-0.68, 2.85]		1.43 [0.43, 2.42]
		Logistic model	2.64	28.56	IPW untruncated	3.64	2.25	1.38 [-0.42, 3.19]		1.59 [0.50, 2.68]
					IPW truncated at 20	3.64	2.25	1.38 [-0.42, 3.19]		1.59 [0.50, 2.68]
					IPW truncated at 99th	3.65	2.30	1.36 [-0.46, 3.18]		1.55 [0.49, 2.62]

* Discontinuation refers to the interruption of the comparator medication initiated on index date; Crossover refers to the initiation of the comparator medication initiated by patient at baseline in the other arm.

© 2025 Neugebauer R et al. *JAMA Network Open*.

eFigure 58. MACE (Primary Definition), 2-Arm Drug Class Comparison, SGLT2is vs GLP-1RAs, White Subgroup, Cumulative Incidence Curves From PP and ITT Analyses With IPW, TMLE, and SL
Each plot emulates inferences among patients in Race SUBGRP White from a 2-arm RCT comparing SGLT2i and GLP-1RA and represents unadjusted or adjusted estimates of cumulative incidence curves for MACE derived with IPW and TMLE with SL estimates of propensity scores with four weight truncation schemes: IPW and TMLE without weight truncation (untruncated), IPW with truncation of stabilized weights at value 20 (trunc20) or at the 99th percentile of weight values (trunc99), and TMLE with truncation of unstabilized weights at value 200 (trunc200). The red divider line separates results of Per-Protocol (PP) analyses (top half) from Intention-To-Treat (ITT) analyses (bottom half). Each plot displays a p value for the test that the average risk difference (ARD) through 2.5 years of follow-up (30 months) is 0.

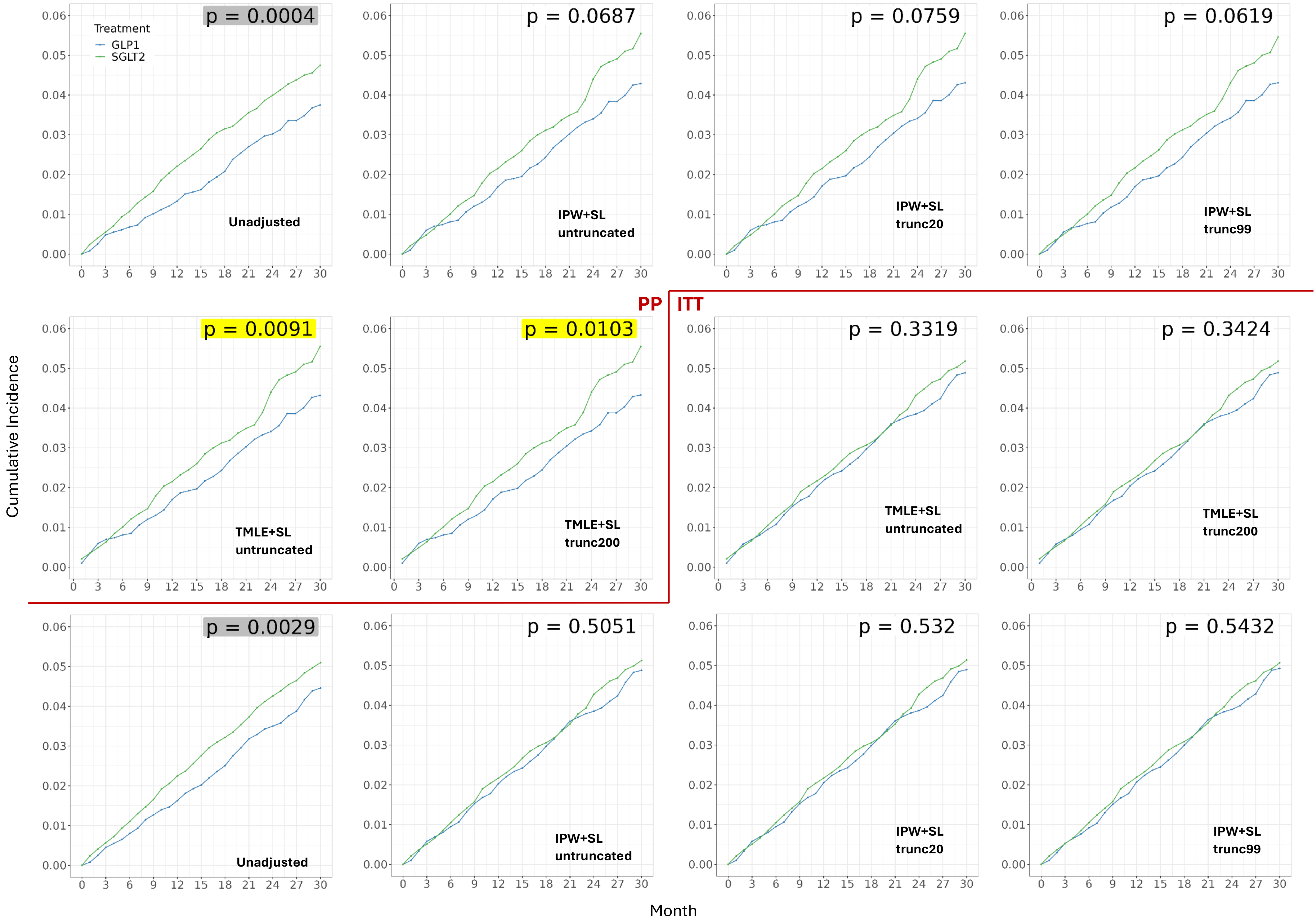

© 2025 Neugebauer R et al. *JAMA Network Open*.

eTable 57. MACE (Primary Definition), 2-Arm Drug Class Comparison, SGLT2is vs GLP-1RAs, White Subgroup, RD and HR Effect Measures at 2.5 Years

Estimation results among White patients from ITT and PP analyses of emulated 2-arm RCTs comparing MACE risks over 2.5 years between SGLT2i and GLP-1RA initiators. For PP analyses, rates of protocol deviations are described by medication class initiated at baseline. Unadjusted point and interval estimates and adjusted point and interval IPW and TMLE estimates of risks, risk differences (RD), and hazard ratios (HR) based on propensity scores (PS) estimated with either logistic models or super learning (SL) are presented for four weight truncation schemes along with the corresponding 99th percentile and maximum value of the stabilized and unstabilized inverse probability weights used for implementing IPW and TMLE, respectively. RD is the risk in treatment arm minus the risk in control arm and NNT is the number needed to treat.

Analysis type	Protocol Deviations* by exposure group (%)	PS estimation	99th IP weights	Max IP weight	Estimator	Treatment (SGLT2i) risk in %	Control (GLP-1RA) risk in %	RD [95% CI] in %	NNT	HR [95% CI]
PP	Discontinuation<br>SGLT2i: 25.99<br>GLP-1RA: 43.29<br><br>Crossover<br>SGLT2i: 7.69<br>GLP-1RA: 11.46	SL			Unadjusted	4.75	3.75	1.00 [0.05, 1.95]	100	1.61 [1.25, 1.98]
			13.72	846.54	TMLE untruncated	5.55	4.32	1.23 [0.46, 2.00]	81	
					TMLE truncated at 200	5.55	4.33	1.22 [0.44, 1.99]	82	
			2.90	123.17	IPW untruncated	5.55	4.29	1.26 [-0.32, 2.84]		1.26 [0.89, 1.64]
					IPW truncated at 20	5.55	4.31	1.24 [-0.34, 2.83]		1.26 [0.88, 1.64]
					IPW truncated at 99th	5.46	4.31	1.14 [-0.32, 2.60]		1.30 [0.95, 1.66]
		Logistic model	4.39	371.38	IPW untruncated	5.64	4.57	1.07 [-0.81, 2.94]		1.28 [0.87, 1.70]
					IPW truncated at 20	5.67	4.61	1.06 [-0.82, 2.94]		1.29 [0.87, 1.70]
					IPW truncated at 99th	5.84	4.70	1.14 [-0.79, 3.07]		1.28 [0.90, 1.67]
ITT		SL			Unadjusted	5.10	4.46	0.64 [-0.09, 1.37]		1.37 [1.12, 1.62]
			8.48	559.07	TMLE untruncated	5.18	4.89	0.29 [-0.28, 0.87]		
					TMLE truncated at 200	5.18	4.89	0.29 [-0.29, 0.86]		
			2.72	139.88	IPW untruncated	5.13	4.88	0.25 [-0.74, 1.25]		1.09 [0.84, 1.35]
					IPW truncated at 20	5.14	4.90	0.24 [-0.76, 1.23]		1.09 [0.84, 1.35]
					IPW truncated at 99th	5.07	4.93	0.13 [-0.78, 1.05]		1.11 [0.86, 1.36]
		Logistic model	3.95	435.04	IPW untruncated	4.90	4.63	0.27 [-0.67, 1.22]		1.12 [0.83, 1.41]
					IPW truncated at 20	4.93	4.66	0.26 [-0.69, 1.22]		1.12 [0.83, 1.41]
					IPW truncated at 99th	4.98	4.77	0.21 [-0.75, 1.17]		1.11 [0.85, 1.38]

* Discontinuation refers to the interruption of the comparator medication initiated on index date; Crossover refers to the initiation of the comparator medication initiated by patient at baseline in the other arm.

© 2025 Neugebauer R et al. *JAMA Network Open*.

eFigure 59. MACE (Primary Definition), 2-Arm Drug Class Comparison, SGLT2is vs GLP-1RAs, Hispanic Subgroup, Cumulative Incidence Curves From PP and ITT Analyses With IPW, TMLE, and SL
Each plot emulates inferences among patients where Ethnicity is Hispanic from a 2-arm RCT comparing SGLT2i and GLP-1RA and represents unadjusted or adjusted estimates of cumulative incidence curves for MACE derived with IPW and TMLE with SL estimates of propensity scores with four weight truncation schemes: IPW and TMLE without weight truncation (untruncated), IPW with truncation of stabilized weights at value 20 (trunc20) or at the 99th percentile of weight values (trunc99), and TMLE with truncation of unstabilized weights at value 200 (trunc200). The red divider line separates results of Per-Protocol (PP) analyses (top half) from Intention-To-Treat (ITT) analyses (bottom half). Each plot displays a p value for the test that the average risk difference (ARD) through 2.5 years of follow-up (30 months) is 0.

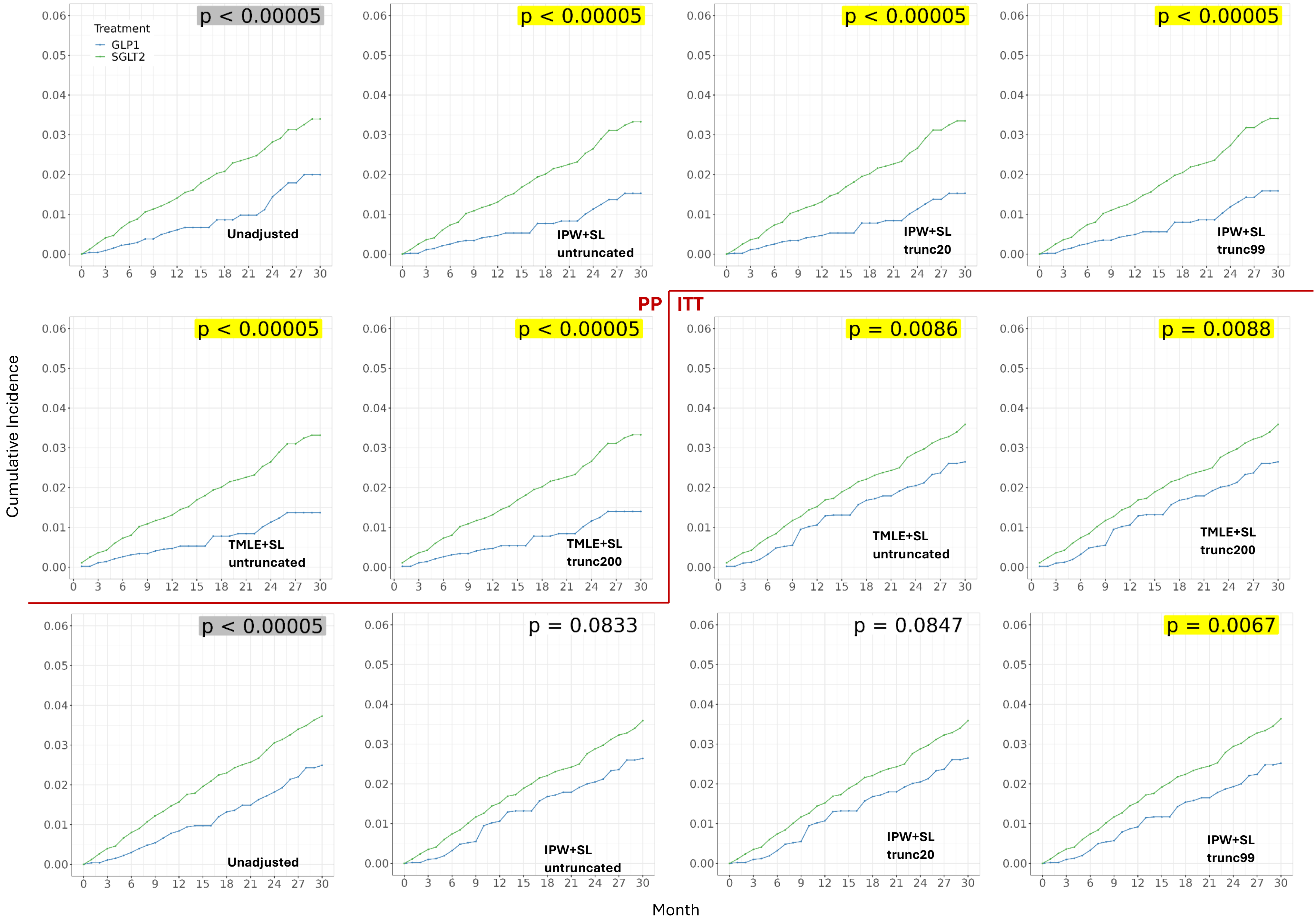

© 2025 Neugebauer R et al. *JAMA Network Open*.

eTable 58. MACE (Primary Definition), 2-Arm Drug Class Comparison, SGLT2is vs GLP-1RAs, Hispanic Subgroup, RD and HR Effect Measures at 2.5 Years

Estimation results among Hispanic patients from ITT and PP analyses of emulated 2-arm RCTs comparing MACE risks over 2.5 years between SGLT2i and GLP-1RA initiators. For PP analyses, rates of protocol deviations are described by medication class initiated at baseline. Unadjusted point and interval estimates and adjusted point and interval IPW and TMLE estimates of risks, risk differences (RD), and hazard ratios (HR) based on propensity scores (PS) estimated with either logistic models or super learning (SL) are presented for four weight truncation schemes along with the corresponding 99th percentile and maximum value of the stabilized and unstabilized inverse probability weights used for implementing IPW and TMLE, respectively. RD is the risk in treatment arm minus the risk in control arm and NNT is the number needed to treat.

Analysis type	Protocol Deviations* by exposure group (%)	PS estimation	99th IP weights	Max IP weight	Estimator	Treatment (SGLT2i) risk in %	Control (GLP-1RA) risk in %	RD [95% CI] in %	NNT	HR [95% CI]
PP	<u>Discontinuation</u><br>SGLT2i: 27.22<br>GLP-1RA: 46.61<br><br><u>Crossover</u><br>SGLT2i: 3.45<br>GLP-1RA: 6.34	SL			Unadjusted	3.40	2.00	1.40 [0.17, 2.63]	71	2.66 [1.31, 4.00]
			17.38	1,350.69	TMLE untruncated	3.32	1.37	1.95 [1.49, 2.40]	51	
					TMLE truncated at 200	3.33	1.40	1.93 [1.48, 2.38]	52	
			2.89	280.41	IPW untruncated	3.33	1.53	1.81 [0.63, 2.98]	55	3.08 [1.22, 4.94]
					IPW truncated at 20	3.35	1.53	1.82 [0.64, 2.99]	55	3.07 [1.22, 4.93]
					IPW truncated at 99th	3.41	1.59	1.82 [0.62, 3.02]	55	2.98 [1.18, 4.78]
		Logistic model	4.30	220.60	IPW untruncated	3.18	1.32	1.86 [0.74, 2.98]	54	2.97 [1.08, 4.85]
					IPW truncated at 20	3.20	1.34	1.86 [0.74, 2.98]	54	2.93 [1.07, 4.79]
					IPW truncated at 99th	3.29	1.41	1.88 [0.73, 3.03]	53	2.80 [1.03, 4.58]
ITT		SL			Unadjusted	3.73	2.49	1.24 [0.40, 2.08]	81	1.99 [1.26, 2.71]
			10.92	389.51	TMLE untruncated	3.59	2.65	0.95 [0.34, 1.55]	106	
					TMLE truncated at 200	3.59	2.65	0.94 [0.33, 1.55]	106	
			2.79	66.92	IPW untruncated	3.59	2.64	0.95 [-0.17, 2.07]		1.56 [0.67, 2.44]
					IPW truncated at 20	3.59	2.65	0.95 [-0.17, 2.07]		1.55 [0.67, 2.43]
					IPW truncated at 99th	3.64	2.52	1.12 [0.15, 2.09]	89	1.73 [0.94, 2.52]
		Logistic model	4.15	339.86	IPW untruncated	3.86	2.62	1.24 [-0.33, 2.82]		1.62 [0.30, 2.94]
					IPW truncated at 20	3.71	2.66	1.05 [-0.38, 2.47]		1.46 [0.36, 2.57]
					IPW truncated at 99th	3.57	2.53	1.04 [-0.02, 2.10]	96	1.55 [0.73, 2.36]

* Discontinuation refers to the interruption of the comparator medication initiated on index date; Crossover refers to the initiation of the comparator medication initiated by patient at baseline in the other arm.

© 2025 Neugebauer R et al. *JAMA Network Open*.

eFigure 60. MACE (Primary Definition), 2-Arm Drug Agent, Exenatide vs Liraglutide, Cumulative Incidence Curves From PP and ITT Analyses With IPW, TMLE, and SL
Each plot emulates inferences from a 2-arm RCT comparing agents Exenatide and Liraglutide and represents unadjusted or adjusted estimates of cumulative incidence curves for MACE derived with IPW and TMLE with SL estimates of propensity scores with four weight truncation schemes: IPW and TMLE without weight truncation (untruncated), IPW with truncation of stabilized weights at value 20 (trunc20) or at the 99th percentile of weight values (trunc99), and TMLE with truncation of unstabilized weights at value 200 (trunc200). The red divider line separates results of Per-Protocol (PP) analyses (top half) from Intention-To-Treat (ITT) analyses (bottom half). Each plot displays a p value for the test that the average risk difference (ARD) through 2.5 years of follow-up (30 months) is 0.

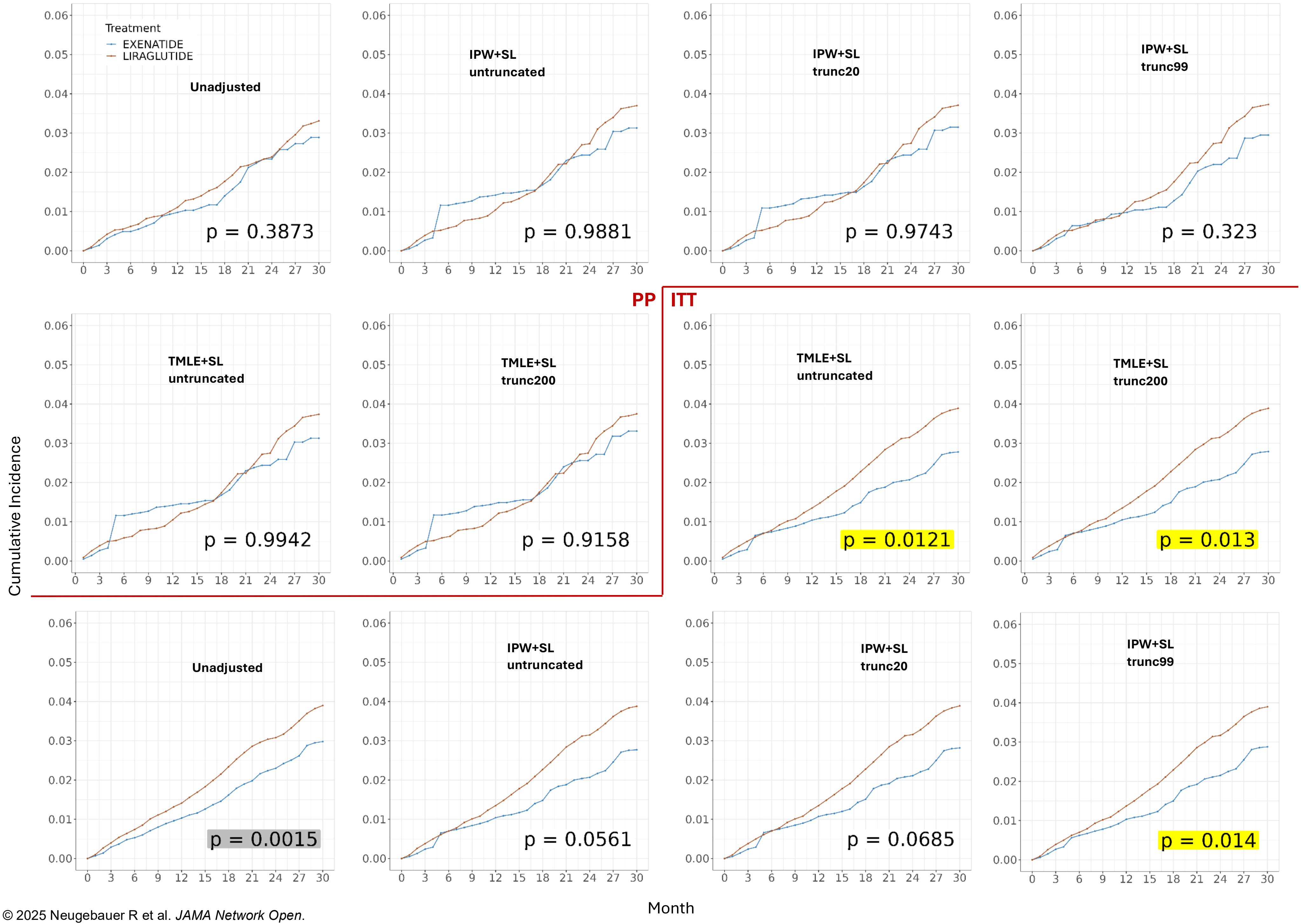

© 2025 Neugebauer R et al. *JAMA Network Open*.

eTable 59. MACE (Primary Definition), 2-Arm Drug Agent, Exenatide vs Liraglutide, RD and HR Effect Measures at 2.5 Years

Estimation results from ITT and PP analyses of emulated 2-arm RCTs comparing MACE risks over 2.5 years between Exenatide and Liraglutide initiators. For PP analyses, rates of protocol deviations are described by agent initiated at baseline. Unadjusted point and interval estimates and adjusted point and interval IPW and TMLE estimates of risks, risk differences (RD), and hazard ratios (HR) based on propensity scores (PS) estimated with either logistic models or super learning (SL) are presented for four weight truncation schemes along with the corresponding 99th percentile and maximum value of the stabilized and unstabilized inverse probability weights used for implementing IPW and TMLE, respectively. RD is the risk in treatment arm minus the risk in control arm and NNT is the number needed to treat.

Analysis type	Protocol Deviations* by exposure group (%)	PS estimation	99th IP weights	Max IP weight	Estimator	Treatment (Exe) risk in %	Control (Lira) risk in %	RD [95% CI] in %	NNT	HR [95% CI]
PP	Discontinuation<br>Exe: 70.83<br>Lira: 46.84<br><br>Crossover<br>Exe: 21.83<br>Lira: 1.20	SL	[redacted]		Unadjusted	2.89	3.31	-0.42 [-1.53, 0.70]		0.79 [0.50, 1.08]
			22.27	1,579.20	TMLE untruncated	3.13	3.74	-0.61 [-1.94, 0.71]		[redacted]
					TMLE truncated at 200	3.31	3.75	-0.44 [-1.75, 0.88]		
			4.49	172.23	IPW untruncated	3.13	3.70	-0.57 [-2.73, 1.59]		1.28 [-0.12, 2.68]
					IPW truncated at 20	3.15	3.71	-0.55 [-2.62, 1.52]		1.23 [-0.05, 2.52]
					IPW truncated at 99th	2.95	3.73	-0.77 [-2.45, 0.90]		0.88 [0.38, 1.38]
		Logistic model	6.96	3,641.75	IPW untruncated	11.06	4.10	6.96 [-11.05, 24.96]		9.56 [-8.85, 27.97]
					IPW truncated at 20	2.75	4.16	-1.41 [-3.73, 0.91]		1.04 [-0.01, 2.10]
					IPW truncated at 99th	2.96	4.25	-1.28 [-3.60, 1.03]		0.92 [0.32, 1.52]
ITT	[redacted]	SL	[redacted]		Unadjusted	2.98	3.90	-0.92 [-1.58, -0.26]	109	0.69 [0.50, 0.88]
			14.64	1,018.36	TMLE untruncated	2.78	3.89	-1.11 [-1.78, -0.44]	90	[redacted]
					TMLE truncated at 200	2.79	3.89	-1.10 [-1.77, -0.43]	91	
			4.70	284.98	IPW untruncated	2.77	3.88	-1.11 [-2.11, -0.11]	90	0.69 [0.29, 1.09]
					IPW truncated at 20	2.82	3.89	-1.07 [-2.08, -0.06]	93	0.70 [0.30, 1.11]
					IPW truncated at 99th	2.88	3.90	-1.03 [-1.93, -0.12]	97	0.67 [0.40, 0.94]
		Logistic model	7.17	1,755.16	IPW untruncated	5.03	3.71	1.32 [-5.78, 8.42]		2.76 [-2.31, 7.83]
					IPW truncated at 20	2.72	3.71	-1.00 [-2.21, 0.22]		0.71 [0.21, 1.21]
					IPW truncated at 99th	2.83	3.73	-0.90 [-1.92, 0.13]		0.71 [0.36, 1.05]

* Discontinuation refers to the interruption of the comparator medication initiated on index date; Crossover refers to the initiation of the comparator medication initiated by patient at baseline in the other arm.

© 2025 Neugebauer R et al. *JAMA Network Open*.

eFigure 61. MACE (Primary Definition), 2-Arm Drug Agent, Semaglutide vs Liraglutide, Cumulative Incidence Curves From PP and ITT Analyses With IPW, TMLE, and SL
Each plot emulates inferences from a 2-arm RCT comparing agents Semaglutide and Liraglutide and represents unadjusted or adjusted estimates of cumulative incidence curves for MACE derived with IPW and TMLE with SL estimates of propensity scores with four weight truncation schemes: IPW and TMLE without weight truncation (untruncated), IPW with truncation of stabilized weights at value 20 (trunc20) or at the 99th percentile of weight values (trunc99), and TMLE with truncation of unstabilized weights at value 200 (trunc200). The red divider line separates results of Per-Protocol (PP) analyses (top half) from Intention-To-Treat (ITT) analyses (bottom half). Each plot displays a p value for the test that the average risk difference (ARD) through 2.5 years of follow-up (30 months) is 0.

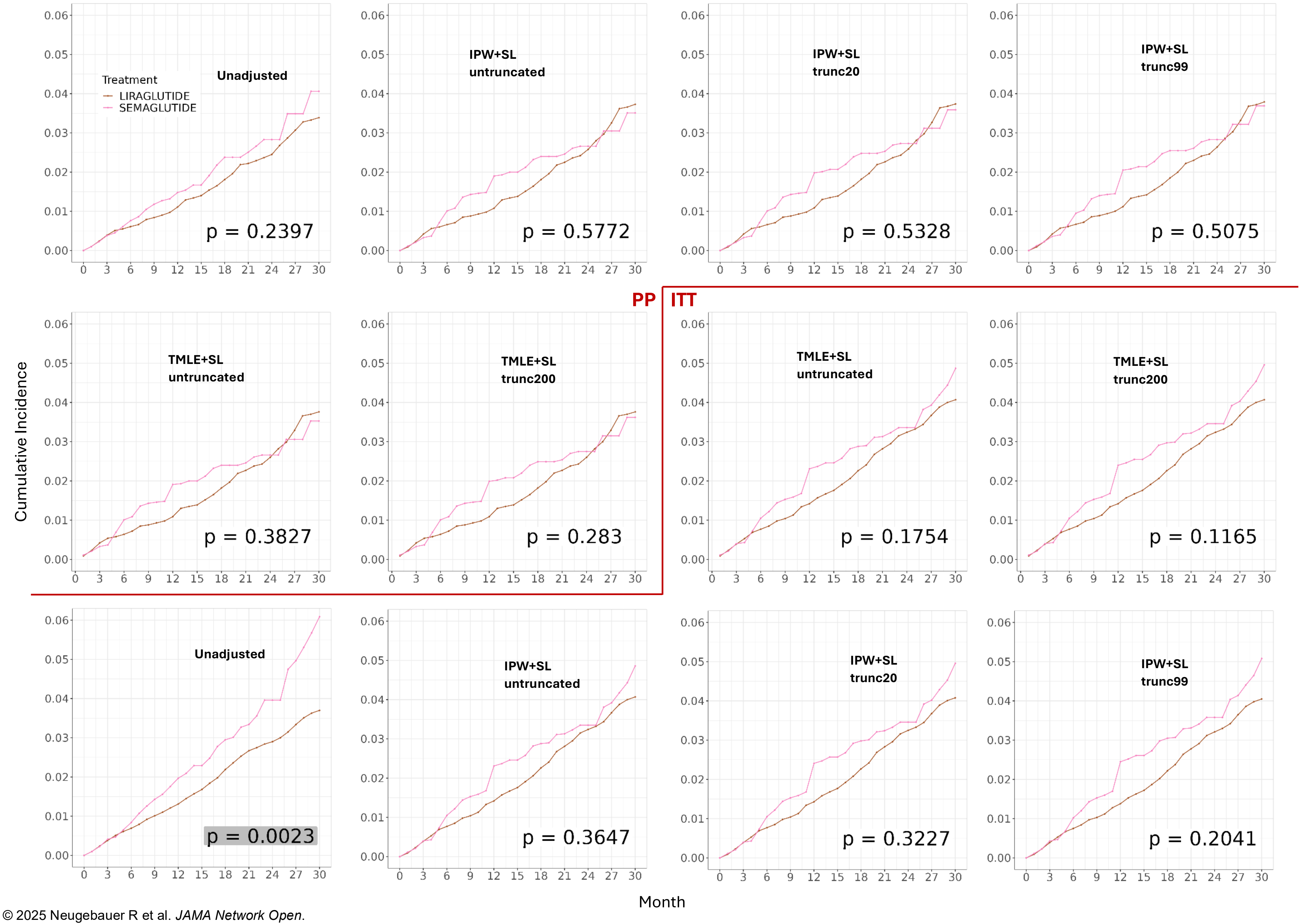

© 2025 Neugebauer R et al. *JAMA Network Open*.

eTable 60. MACE (Primary Definition), 2-Arm Drug Agent, Semaglutide vs Liraglutide, RD and HR Effect Measures at 2.5 Years

Estimation results from ITT and PP analyses of emulated 2-arm RCTs comparing MACE risks over 2.5 years between Semaglutide and Liraglutide initiators. For PP analyses, rates of protocol deviations are described by agent initiated at baseline. Unadjusted point and interval estimates and adjusted point and interval IPW and TMLE estimates of risks, risk differences (RD), and hazard ratios (HR) based on propensity scores (PS) estimated with either logistic models or super learning (SL) are presented for four weight truncation schemes along with the corresponding 99th percentile and maximum value of the stabilized and unstabilized inverse probability weights used for implementing IPW and TMLE, respectively. RD is the risk in treatment arm minus the risk in control arm and NNT is the number needed to treat.

Analysis type	Protocol Deviations* by exposure group (%)	PS estimation	99th IP weights	Max IP weight	Estimator	Treatment (Sema) risk in %	Control (Lira) risk in %	RD [95% CI] in %	NNT	HR [95% CI]
PP	Discontinuation<br>Sema: 31.98<br>Lira: 43.71<br><br>Crossover<br>Sema: 1.98<br>Lira: 4.56	SL			Unadjusted	4.06	3.39	0.67 [-0.82, 2.16]		1.23 [0.84, 1.62]
			17.71	1,374.78	TMLE untruncated	3.53	3.76	-0.23 [-0.99, 0.53]		
					TMLE truncated at 200	3.62	3.76	-0.14 [-0.90, 0.62]		
			3.86	112.77	IPW untruncated	3.51	3.73	-0.21 [-1.86, 1.44]		1.41 [0.64, 2.19]
					IPW truncated at 20	3.59	3.74	-0.14 [-1.85, 1.56]		1.42 [0.64, 2.20]
					IPW truncated at 99th	3.69	3.79	-0.09 [-1.84, 1.66]		1.39 [0.69, 2.09]
		Logistic model	5.02	379.79	IPW untruncated	3.68	3.43	0.25 [-1.75, 2.25]		1.66 [0.65, 2.68]
					IPW truncated at 20	3.73	3.54	0.20 [-1.84, 2.23]		1.68 [0.66, 2.70]
					IPW truncated at 99th	3.85	3.69	0.16 [-1.92, 2.24]		1.60 [0.71, 2.48]
ITT		SL			Unadjusted	6.09	3.70	2.39 [1.09, 3.70]	42	1.41 [1.05, 1.77]
			13.73	948.77	TMLE untruncated	4.87	4.07	0.80 [-0.00, 1.59]	126	
					TMLE truncated at 200	4.96	4.07	0.89 [0.10, 1.69]	112	
			3.76	119.30	IPW untruncated	4.86	4.07	0.79 [-0.72, 2.30]		1.45 [0.78, 2.11]
					IPW truncated at 20	4.96	4.08	0.88 [-0.70, 2.45]		1.45 [0.79, 2.12]
					IPW truncated at 99th	5.08	4.05	1.03 [-0.49, 2.56]		1.49 [0.89, 2.09]
		Logistic model	4.78	480.08	IPW untruncated	4.72	4.01	0.72 [-1.18, 2.62]		1.62 [0.78, 2.47]
					IPW truncated at 20	4.83	4.08	0.74 [-1.20, 2.69]		1.64 [0.79, 2.49]
					IPW truncated at 99th	4.85	4.00	0.85 [-0.85, 2.54]		1.59 [0.88, 2.29]

* Discontinuation refers to the interruption of the comparator medication initiated on index date; Crossover refers to the initiation of the comparator medication initiated by patient at baseline in the other arm.

© 2025 Neugebauer R et al. *JAMA Network Open*.

eFigure 62. MACE (Broader Definition), 2-Arm Drug Class, Sulfonylureas vs DPP4is, Cumulative Incidence Curves From PP and ITT Analyses With IPW, TMLE, and SL
Each plot emulates inferences from a 2-arm RCT comparing SU and DPP4i and represents unadjusted or adjusted estimates of cumulative incidence curves for MACE (expanded) derived with inverse probability weighting (IPW) and Targeted Minimum Loss-based Estimation (TMLE) with Super Learning (SL) estimates of propensity scores with four weight truncation schemes: IPW and TMLE without weight truncation (untruncated), IPW with truncation of stabilized weights at value 20 (trunc20) or at the 99th percentile of weight values (trunc99), and TMLE with truncation of unstabilized weights at value 200 (trunc200). The red divi der line separates results of Per-Protocol (PP) analyses (top half) from Intention-To-Treat (ITT) analyses (bottom half). Each plot displays a p value for the test that the average risk difference (ARD) through 2.5 years of follow-up (30 months) is 0.

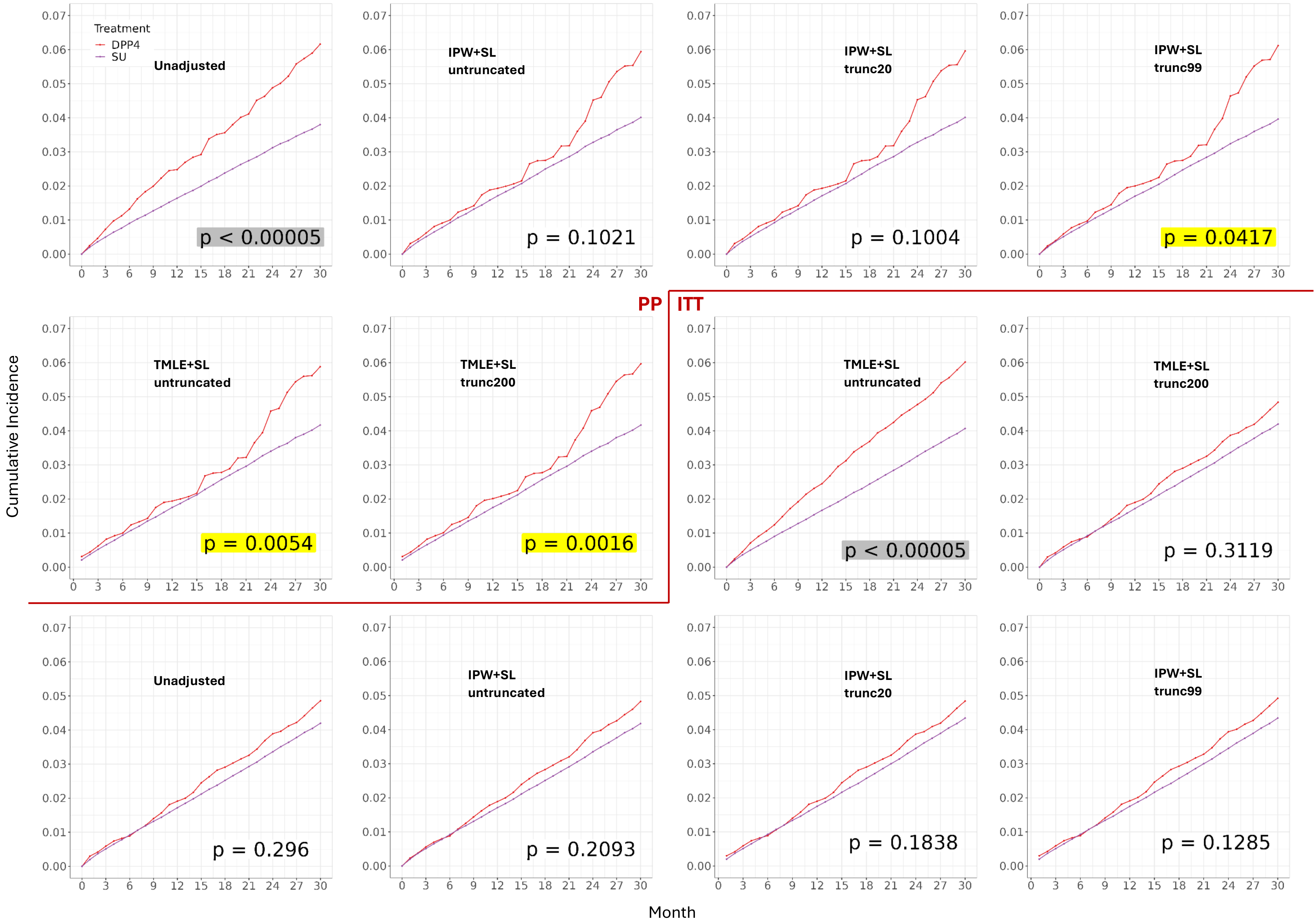

© 2025 Neugebauer R et al. *JAMA Network Open*.

eTable 61. MACE (Broader Definition), 2-Arm Drug Class, Sulfonylureas vs DPP4is, RD and HR Effect Measures at 2.5 Years

Estimation results from ITT and PP analyses of emulated 2-arm RCTs comparing MACE (expanded) risks over 2.5 years between SU and DPP4i initiators. For PP analyses, rates of protocol deviations are described by medication class initiated at baseline. Unadjusted point and interval estimates and adjusted point and interval IPW and TMLE estimates of risks, risk differences (RD), and hazard ratios (HR) based on propensity scores (PS) estimated with either logistic models or super learning (SL) are presented for four weight truncation schemes along with the corresponding 99th percentile and maximum value of the stabilized and unstabilized inverse probability weights used for implementing IPW and TMLE, respectively. RD is the risk in treatment arm minus the risk in control arm and NNT is the number needed to treat.

Analysis type	Protocol Deviations* by exposure group (%)	PS estimation	99th IP weights	Max IP weight	Estimator	Treatment (DPP4i) risk in %	Control (SU) risk in %	RD [95% CI] in %	NNT	HR [95% CI]
PP	Discontinuation<br>SU: 49.47<br>DPP4i: 53.33<br><br>Crossover<br>SU: 2.25<br>DPP4i: 14.44	SL			Unadjusted	6.16	3.80	2.35 [1.35, 3.35]	43	1.53 [1.28, 1.77]
			21.14	5,124.22	TMLE untruncated	5.88	4.17	1.71 [0.93, 2.49]	59	
					TMLE truncated at 200	5.97	4.17	1.80 [1.14, 2.47]	55	
			2.27	125.82	IPW untruncated	5.94	4.01	1.93 [0.20, 3.66]	52	1.14 [0.80, 1.49]
					IPW truncated at 20	5.96	4.01	1.94 [0.21, 3.68]	51	1.14 [0.80, 1.49]
					IPW truncated at 99th	6.12	3.96	2.16 [0.44, 3.88]	46	1.15 [0.86, 1.44]
		Logistic model	2.88	37,991.02	IPW untruncated	5.97	3.59	2.38 [-0.07, 4.84]		1.21 [0.73, 1.68]
					IPW truncated at 20	6.02	4.23	1.79 [-0.30, 3.88]		1.15 [0.74, 1.56]
					IPW truncated at 99th	6.12	4.07	2.05 [0.26, 3.84]	49	1.19 [0.86, 1.52]
ITT		SL			Unadjusted	6.02	4.07	1.95 [1.30, 2.61]	51	1.52 [1.32, 1.71]
			20.92	6,631.36	TMLE untruncated	4.84	4.34	0.50 [-0.06, 1.07]		
					TMLE truncated at 200	4.92	4.34	0.58 [0.02, 1.14]	171	
			1.87	30.27	IPW untruncated	4.84	4.20	0.64 [-0.36, 1.63]		1.12 [0.84, 1.40]
					IPW truncated at 20	4.86	4.20	0.66 [-0.34, 1.66]		1.12 [0.85, 1.40]
					IPW truncated at 99th	4.83	4.18	0.65 [-0.20, 1.51]		1.11 [0.89, 1.34]
		Logistic model	2.54	488.79	IPW untruncated	4.82	4.31	0.51 [-0.58, 1.61]		1.06 [0.77, 1.36]
					IPW truncated at 20	4.92	4.27	0.65 [-0.46, 1.76]		1.11 [0.81, 1.41]
					IPW truncated at 99th	5.13	4.23	0.90 [-0.07, 1.88]		1.16 [0.91, 1.42]

* Discontinuation refers to the interruption of the comparator medication initiated on index date; Crossover refers to the initiation of the comparator medication initiated by patient at baseline in the other arm.

© 2025 Neugebauer R et al. *JAMA Network Open*.

eFigure 63. MACE (Broader Definition), 2-Arm Drug Class, Sulfonylureas vs SGLT2is, Cumulative Incidence Curves From PP and ITT Analyses With IPW, TMLE, and SL
Each plot emulates inferences from a 2-arm RCT comparing SU and SGLT2i and represents unadjusted or adjusted estimates of cumulative incidence curves for MACE (expanded) derived with inverse probability weighting (IPW) and Targeted Minimum Loss-based Estimation (TMLE) with Super Learning (SL) estimates of propensity scores with four weight truncation schemes: IPW and TMLE without weight truncation (untruncated), IPW with truncation of stabilized weights at value 20 (trunc20) or at the 99th percentile of weight values (trunc99), and TMLE with truncation of unstabilized weights at value 200 (trunc200). The red divi der line separates results of Per-Protocol (PP) analyses (top half) from Intention-To-Treat (ITT) analyses (bottom half). Each plot displays a p value for the test that the average risk difference (ARD) through 2.5 years of follow-up (30 months) is 0.

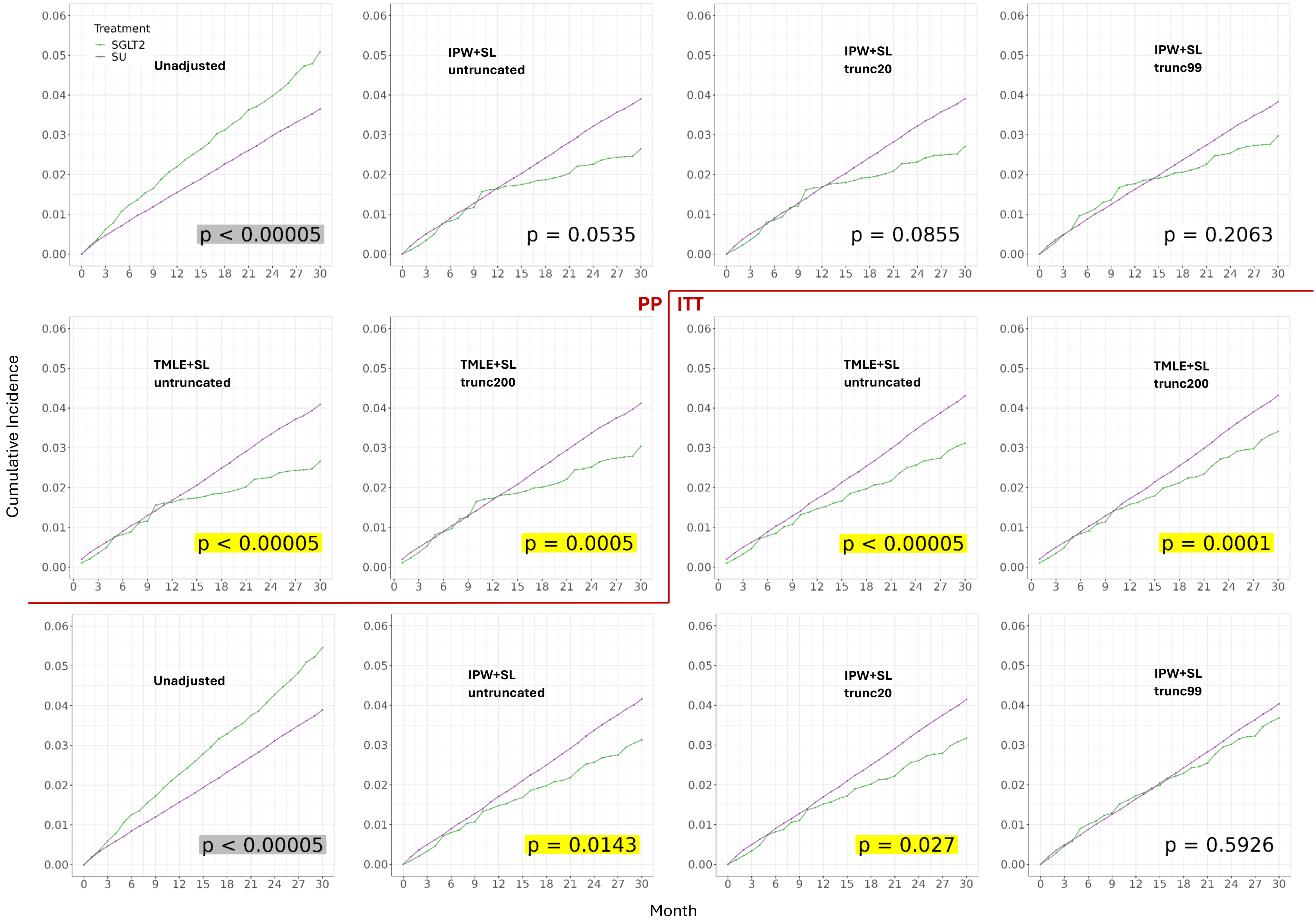

© 2025 Neugebauer R et al. *JAMA Network Open*.

eTable 62. MACE (Broader Definition), 2-Arm Drug Class, Sulfonylureas vs SGLT2is, RD and HR Effect Measures at 2.5 Years

Estimation results from ITT and PP analyses of emulated 2-arm RCTs comparing MACE (expanded) risks over 2.5 years between SU and SGLT2i initiators. For PP analyses, rates of protocol deviations are described by medication class initiated at baseline. Unadjusted point and interval estimates and adjusted point and interval IPW and TMLE estimates of risks, risk differences (RD), and hazard ratios (HR) based on propensity scores (PS) estimated with either logistic models or super learning (SL) are presented for four weight truncation schemes along with the corresponding 99th percentile and maximum value of the stabilized and unstabilized inverse probability weights used for implementing IPW and TMLE, respectively. RD is the risk in treatment arm minus the risk in control arm and NNT is the number needed to treat.

Analysis type	Protocol Deviations* by exposure group (%)	PS estimation	99th IP weights	Max IP weight	Estimator	Treatment (SU) risk in %	Control (SGLT2i) risk in %	RD [95% CI] in %	NNT	HR [95% CI]
PP	Discontinuation<br>SU: 49.59<br>SGLT2i: 27.04<br><br>Crossover<br>SU: 4.43<br>SGLT2i: 5.85	SL			Unadjusted	3.65	5.09	-1.43 [-2.18, -0.69]	70	0.71 [0.62, 0.80]
			15.43	3,827.58	TMLE untruncated	4.09	2.66	1.43 [1.04, 1.82]	70	
					TMLE truncated at 200	4.12	3.04	1.08 [0.73, 1.42]	93	
			2.71	1,231.44	IPW untruncated	3.90	2.65	1.25 [0.43, 2.08]	80	1.10 [0.77, 1.44]
					IPW truncated at 20	3.91	2.71	1.20 [0.36, 2.04]	83	1.07 [0.75, 1.39]
					IPW truncated at 99th	3.83	2.97	0.86 [0.15, 1.57]	116	0.93 [0.74, 1.13]
		Logistic model	3.48	7,073.52	IPW untruncated	4.15	2.62	1.53 [0.28, 2.78]	65	1.04 [0.59, 1.49]
					IPW truncated at 20	4.12	2.86	1.27 [0.12, 2.42]	79	0.94 [0.56, 1.33]
					IPW truncated at 99th	3.96	2.95	1.02 [0.30, 1.73]	98	0.90 [0.69, 1.10]
ITT		SL			Unadjusted	3.89	5.46	-1.57 [-2.13, -1.01]	64	0.70 [0.62, 0.77]
			12.13	1,607.56	TMLE untruncated	4.31	3.12	1.19 [0.84, 1.55]	84	
					TMLE truncated at 200	4.32	3.41	0.91 [0.56, 1.26]	110	
			2.39	1,256.83	IPW untruncated	4.16	3.13	1.04 [0.29, 1.78]	96	1.20 [0.89, 1.50]
					IPW truncated at 20	4.15	3.17	0.98 [0.23, 1.72]	102	1.16 [0.87, 1.46]
					IPW truncated at 99th	4.04	3.68	0.36 [-0.38, 1.11]		0.97 [0.78, 1.15]
		Logistic model	3.12	9,069.89	IPW untruncated	4.32	3.62	0.70 [-1.14, 2.53]		1.18 [0.74, 1.62]
					IPW truncated at 20	4.24	3.84	0.40 [-1.01, 1.80]		1.07 [0.71, 1.43]
					IPW truncated at 99th	4.09	3.76	0.32 [-0.48, 1.12]		0.94 [0.75, 1.13]

* Discontinuation refers to the interruption of the comparator medication initiated on index date; Crossover refers to the initiation of the comparator medication initiated by patient at baseline in the other arm.

© 2025 Neugebauer R et al. *JAMA Network Open*.

eFigure 64. MACE (Broader Definition), 2-Arm Drug Class, Sulfonylureas vs GLP-1RAs, Cumulative Incidence Curves From PP and ITT Analyses With IPW, TMLE, and SL
Each plot emulates inferences from a 2-arm RCT comparing SU and GLP-1RA and represents unadjusted or adjusted estimates of cumulative incidence curves for MACE (expanded) derived with inverse probability weighting (IPW) and Targeted Minimum Loss-based Estimation (TMLE) with Super Learning (SL) estimates of propensity scores with four weight truncation schemes: IPW and TMLE without weight truncation (untruncated), IPW with truncation of stabilized weights at value 20 (trunc20) or at the 99th percentile of weight values (trunc99), and TMLE with truncation of unstabilized weights at value 200 (trunc200). The red divider line separates results of Per-Protocol (PP) analyses (top half) from Intention-To-Treat (ITT) analyses (bottom half). Each plot displays a p value for the test that the average risk difference (ARD) through 2.5 years of follow-up (30 months) is 0.

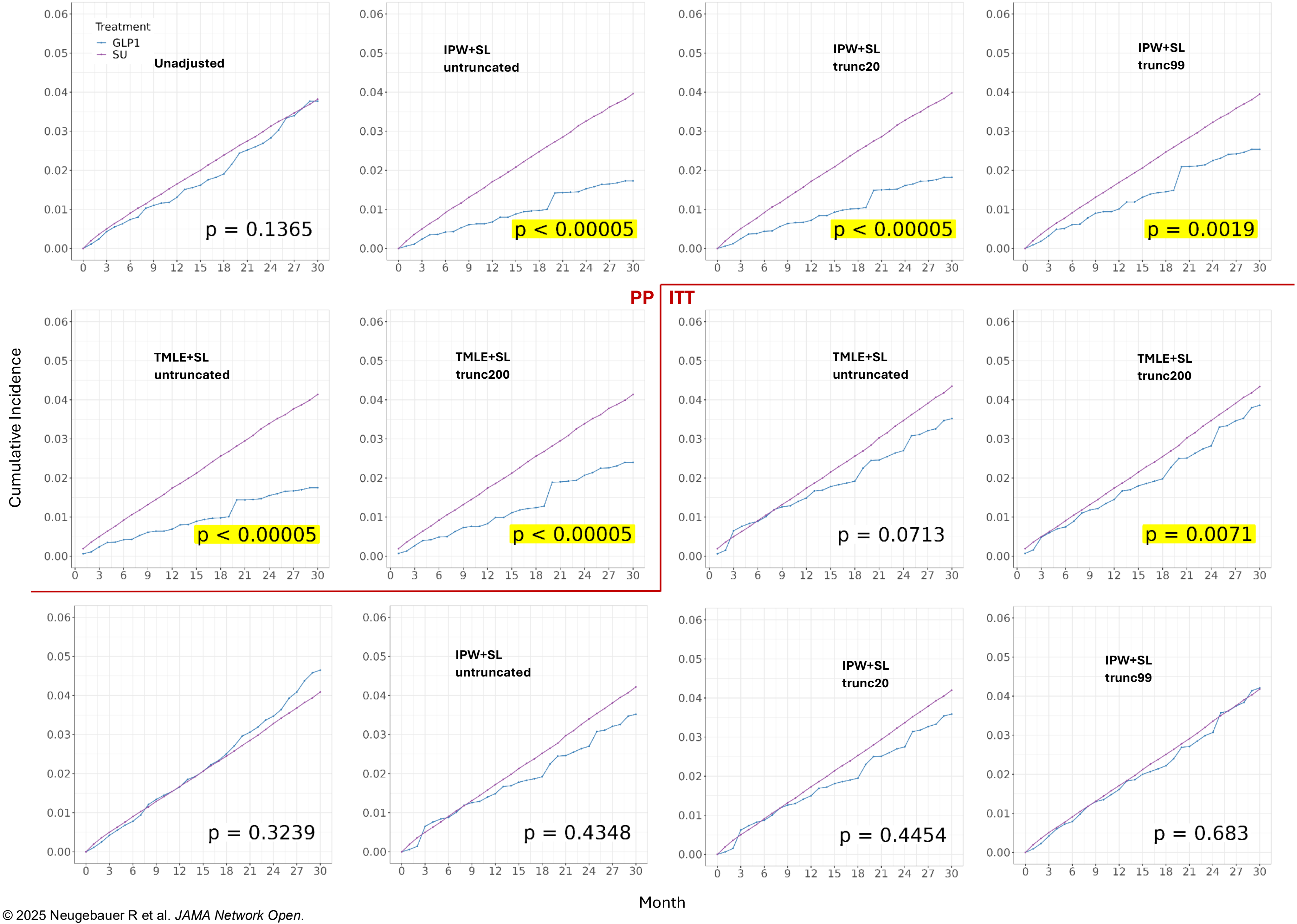

© 2025 Neugebauer R et al. *JAMA Network Open*.

eTable 63. MACE (Broader Definition), 2-Arm Drug Class, Sulfonylureas vs GLP-1RAs, RD and HR Effect Measures at 2.5 Years

Estimation results from ITT and PP analyses of emulated 2-arm RCTs comparing MACE (expanded) risks over 2.5 years between SU and GLP-1RA initiators. For PP analyses, rates of protocol deviations are described by medication class initiated at baseline. Unadjusted point and interval estimates and adjusted point and interval IPW and TMLE estimates of risks, risk differences (RD), and hazard ratios (HR) based on propensity scores (PS) estimated with either logistic models or super learning (SL) are presented for four weight truncation schemes along with the corresponding 99th percentile and maximum value of the stabilized and unstabilized inverse probability weights used for implementing IPW and TMLE, respectively. RD is the risk in treatment arm minus the risk in control arm and NNT is the number needed to treat.

Analysis type	Protocol Deviations* by exposure group (%)	PS estimation	99th IP weights	Max IP weight	Estimator	Treatment (SU) risk in %	Control (GLP-1RA) risk in %	RD [95% CI] in %	NNT	HR [95% CI]
PP	Discontinuation<br>SU: 49.75<br>GLP-1RA: 43.76<br><br>Crossover<br>SU: 1.56<br>GLP-1RA: 3.76	SL			Unadjusted	3.82	3.77	0.05 [-0.63, 0.74]		1.24 [1.02, 1.46]
			10.16	5,191.31	TMLE untruncated	4.14	1.75	2.38 [2.12, 2.64]	42	
					TMLE truncated at 200	4.14	2.40	1.74 [1.49, 1.98]	58	
			2.61	421.05	IPW untruncated	3.96	1.73	2.23 [1.51, 2.95]	45	2.39 [1.40, 3.38]
					IPW truncated at 20	3.98	1.82	2.16 [1.43, 2.90]	46	2.28 [1.35, 3.22]
					IPW truncated at 99th	3.95	2.54	1.41 [0.45, 2.37]	71	1.61 [1.03, 2.20]
		Logistic model	3.22	2,123.47	IPW untruncated	4.34	1.20	3.13 [2.34, 3.93]	32	2.70 [1.18, 4.23]
					IPW truncated at 20	4.17	1.62	2.55 [1.91, 3.19]	39	2.09 [0.99, 3.20]
					IPW truncated at 99th	4.05	2.35	1.70 [0.90, 2.50]	59	1.54 [0.95, 2.13]
ITT		SL			Unadjusted	4.09	4.65	-0.56 [-1.10, -0.03]	177	1.00 [0.87, 1.14]
			7.43	2,532.51	TMLE untruncated	4.35	3.52	0.83 [0.26, 1.40]	121	
					TMLE truncated at 200	4.34	3.86	0.48 [0.07, 0.89]	208	
			2.26	630.83	IPW untruncated	4.22	3.52	0.71 [-0.60, 2.01]		1.19 [0.51, 1.88]
					IPW truncated at 20	4.20	3.59	0.61 [-0.65, 1.88]		1.19 [0.57, 1.81]
					IPW truncated at 99th	4.18	4.21	-0.03 [-1.14, 1.08]		1.10 [0.79, 1.40]
		Logistic model	2.91	3,360.60	IPW untruncated	4.29	3.05	1.24 [-0.01, 2.50]	81	1.29 [0.58, 2.00]
					IPW truncated at 20	4.25	3.65	0.59 [-0.68, 1.87]		1.10 [0.57, 1.62]
					IPW truncated at 99th	4.21	4.34	-0.13 [-1.31, 1.06]		1.01 [0.72, 1.31]

* Discontinuation refers to the interruption of the comparator medication initiated on index date; Crossover refers to the initiation of the comparator medication initiated by patient at baseline in the other arm.

© 2025 Neugebauer R et al. *JAMA Network Open*.

eFigure 65. MACE (Broader Definition), 2-Arm Drug Class, DPP4is vs SGLT2is, Cumulative Incidence Curves From PP and ITT Analyses With IPW, TMLE, and SL
Each plot emulates inferences from a 2-arm RCT comparing DPP4i and SGLT2i and represents unadjusted or adjusted estimates of cumulative incidence curves for MACE (expanded) derived with inverse probability weighting (IPW) and Targeted Minimum Loss-based Estimation (TMLE) with Super Learning (SL) estimates of propensity scores with four weight truncation schemes: IPW and TMLE without weight truncation (untruncated), IPW with truncation of stabilized weights at value 20 (trunc20) or at the 99th percentile of weight values (trunc99), and TMLE with truncation of unstabilized weights at value 200 (trunc200). The red divider line separates results of Per-Protocol (PP) analyses (top half) from Intention-To-Treat (ITT) analyses (bottom half). Each plot displays a p value for the test that the average risk difference (ARD) through 2.5 years of follow-up (30 months) is 0.

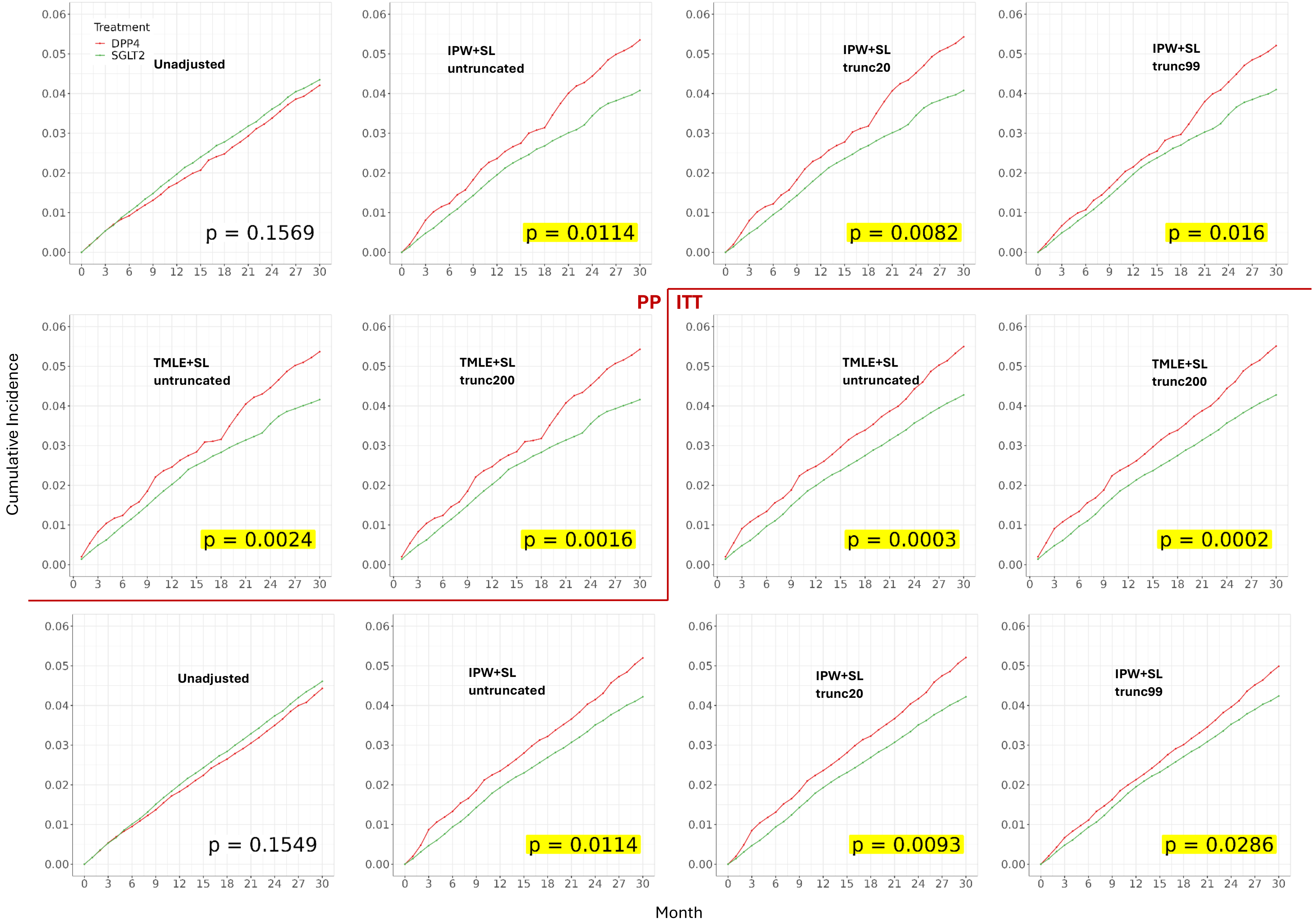

© 2025 Neugebauer R et al. *JAMA Network Open*.

eTable 64. MACE (Broader Definition), 2-Arm Drug Class, DPP4is vs SGLT2is, RD and HR Effect Measures at 2.5 Years

Estimation results from ITT and PP analyses of emulated 2-arm RCTs comparing MACE (expanded) risks over 2.5 years between DPP4i and SGLT2i initiators. For PP analyses, rates of protocol deviations are described by medication class initiated at baseline. Unadjusted point and interval estimates and adjusted point and interval IPW and TMLE estimates of risks, risk differences (RD), and hazard ratios (HR) based on propensity scores (PS) estimated with either logistic models or super learning (SL) are presented for four weight truncation schemes along with the corresponding 99th percentile and maximum value of the stabilized and unstabilized inverse probability weights used for implementing IPW and TMLE, respectively. RD is the risk in treatment arm minus the risk in control arm and NNT is the number needed to treat.

Analysis type	Protocol Deviations* by exposure group (%)	PS estimation	99th IP weights	Max IP weight	Estimator	Treatment (DPP4i) risk in %	Control (SGLT2i) risk in %	RD [95% CI] in %	NNT	HR [95% CI]
PP	<u>Discontinuation</u><br>DPP4i: 60.77<br>SGLT2i: 26.18<br><br><u>Crossover</u><br>DPP4i: 10.83<br>SGLT2i: 1.93	SL			Unadjusted	4.21	4.35	-0.14 [-0.69, 0.41]		0.89 [0.78, 1.00]
			18.32	2,401.48	TMLE untruncated	5.37	4.16	1.21 [0.61, 1.81]	83	
					TMLE truncated at 200	5.43	4.16	1.27 [0.67, 1.87]	79	
			4.49	274.25	IPW untruncated	5.35	4.08	1.27 [0.34, 2.20]	79	1.26 [0.97, 1.55]
					IPW truncated at 20	5.43	4.08	1.34 [0.41, 2.28]	75	1.27 [0.98, 1.56]
					IPW truncated at 99th	5.21	4.10	1.11 [0.29, 1.93]	90	1.14 [0.94, 1.33]
		Logistic model	6.05	2,936.59	IPW untruncated	5.74	4.47	1.27 [-0.40, 2.94]		1.25 [0.72, 1.79]
					IPW truncated at 20	5.74	4.43	1.31 [-0.06, 2.68]		1.11 [0.84, 1.38]
					IPW truncated at 99th	5.28	4.46	0.82 [-0.20, 1.84]		1.07 [0.87, 1.28]
ITT		SL			Unadjusted	4.43	4.61	-0.18 [-0.60, 0.24]		0.92 [0.82, 1.02]
			12.01	736.60	TMLE untruncated	5.50	4.28	1.22 [0.75, 1.68]	82	
					TMLE truncated at 200	5.51	4.28	1.22 [0.76, 1.69]	82	
			4.14	243.28	IPW untruncated	5.20	4.22	0.98 [0.31, 1.65]	102	1.26 [0.99, 1.53]
					IPW truncated at 20	5.21	4.22	0.99 [0.33, 1.66]	101	1.26 [1.00, 1.52]
					IPW truncated at 99th	4.99	4.24	0.75 [0.19, 1.32]	133	1.12 [0.95, 1.29]
		Logistic model	5.54	1,279.45	IPW untruncated	5.48	4.44	1.04 [-0.11, 2.19]		1.23 [0.78, 1.68]
					IPW truncated at 20	5.20	4.44	0.76 [-0.07, 1.60]		1.10 [0.85, 1.34]
					IPW truncated at 99th	5.01	4.42	0.59 [-0.07, 1.26]		1.05 [0.87, 1.24]

* Discontinuation refers to the interruption of the comparator medication initiated on index date; Crossover refers to the initiation of the comparator medication initiated by patient at baseline in the other arm.

© 2025 Neugebauer R et al. *JAMA Network Open*.

eFigure 66. MACE (Broader Definition), 2-Arm Drug Class, DPP4is vs GLP-1RAs, Cumulative Incidence Curves From PP and ITT Analyses With IPW, TMLE, and SL
Each plot emulates inferences from a 2-arm RCT comparing DPP4i and GLP-1RA and represents unadjusted or adjusted estimates of cumulative incidence curves for MACE (expanded) derived with inverse probability weighting (IPW) and Targeted Minimum Loss-based Estimation (TMLE) with Super Learning (SL) estimates of propensity scores with four weight truncation schemes: IPW and TMLE without weight truncation (untruncated), IPW with truncation of stabilized weights at value 20 (trunc20) or at the 99th percentile of weight values (trunc99), and TMLE with truncation of unstabilized weights at value 200 (trunc200). The red divider line separates results of Per-Protocol (PP) analyses (top half) from Intention-To-Treat (ITT) analyses (bottom half). Each plot displays a p value for the test that the average risk difference (ARD) through 2.5 years of follow-up (30 months) is 0.

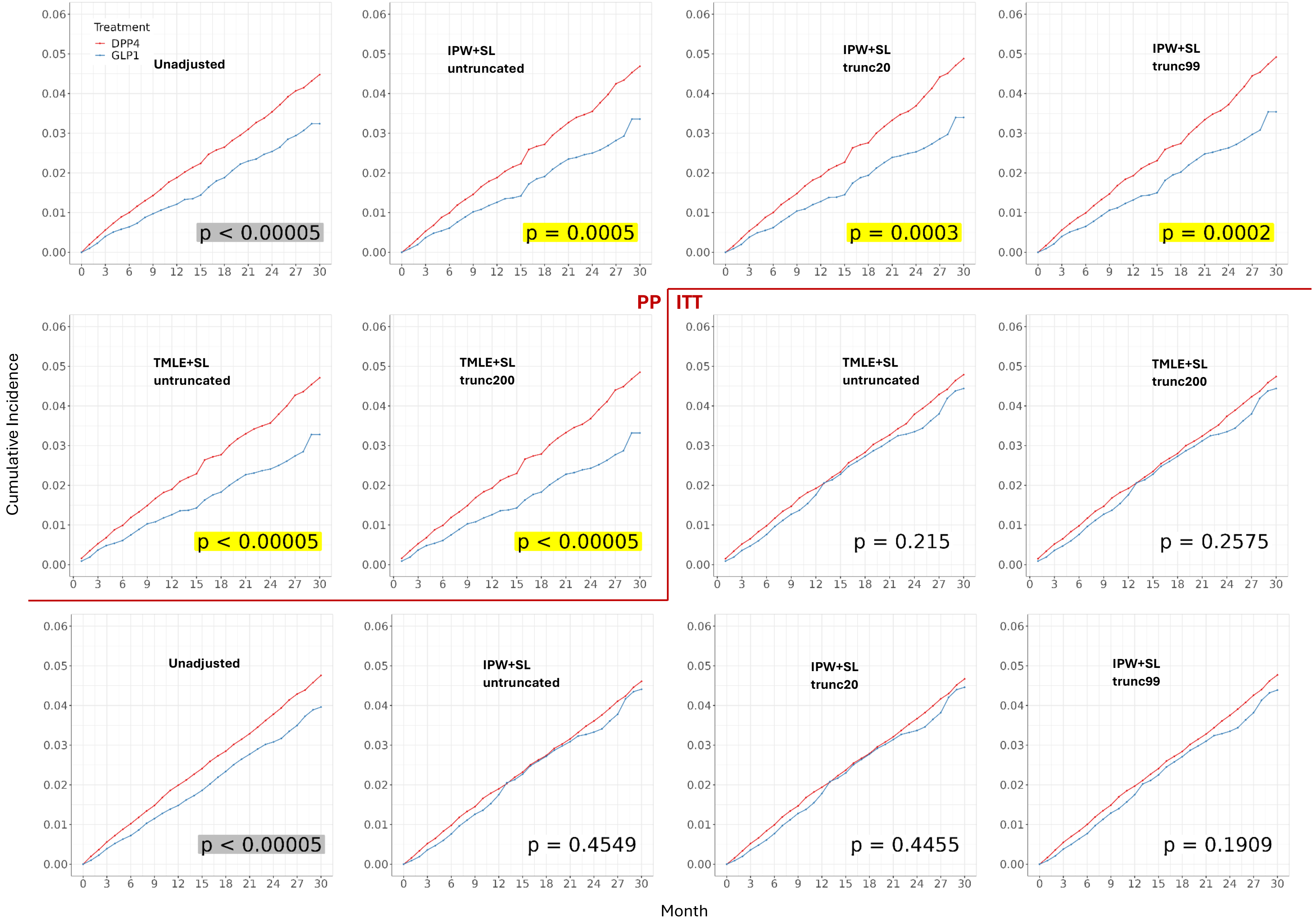

© 2025 Neugebauer R et al. *JAMA Network Open*.

eTable 65. MACE (Broader Definition), 2-Arm Drug Class, DPP4is vs GLP-1RAs, RD and HR Effect Measures at 2.5 Years

Estimation results from ITT and PP analyses of emulated 2-arm RCTs comparing MACE (expanded) risks over 2.5 years between DPP4i and GLP-1RA initiators. For PP analyses, rates of protocol deviations are described by medication class initiated at baseline. Unadjusted point and interval estimates and adjusted point and interval IPW and TMLE estimates of risks, risk differences (RD), and hazard ratios (HR) based on propensity scores (PS) estimated with either logistic models or super learning (SL) are presented for four weight truncation schemes along with the corresponding 99th percentile and maximum value of the stabilized and unstabilized inverse probability weights used for implementing IPW and TMLE, respectively. RD is the risk in treatment arm minus the risk in control arm and NNT is the number needed to treat.

Analysis type	Protocol Deviations* by exposure group (%)	PS estimation	99th IP weights	Max IP weight	Estimator	Treatment (DPP4i) risk in %	Control (GLP-1RA) risk in %	RD [95% CI] in %	NNT	HR [95% CI]
PP	<u>Discontinuation</u><br>DPP4i: 62.28<br>GLP-1RA: 45.36<br><br><u>Crossover</u><br>DPP4i: 6.00<br>GLP-1RA: 1.33	SL	[redacted]	[redacted]	Unadjusted	4.48	3.24	1.24 [0.66, 1.82]	80	1.48 [1.25, 1.72]
			24.05	32,670.83	TMLE untruncated	4.71	3.28	1.43 [0.70, 2.16]	70	[redacted]
					TMLE truncated at 200	4.85	3.32	1.53 [0.82, 2.24]	65	
			5.96	5,506.05	IPW untruncated	4.69	3.36	1.33 [0.23, 2.43]	75	1.51 [1.12, 1.90]
					IPW truncated at 20	4.88	3.40	1.47 [0.37, 2.57]	68	1.51 [1.12, 1.89]
					IPW truncated at 99th	4.92	3.54	1.38 [0.31, 2.46]	72	1.45 [1.11, 1.79]
		Logistic model	7.25	7,050.75	IPW untruncated	4.23	4.63	-0.40 [-4.03, 3.23]		1.47 [0.99, 1.94]
					IPW truncated at 20	5.08	4.20	0.88 [-1.64, 3.40]		1.40 [0.99, 1.82]
					IPW truncated at 99th	4.84	3.65	1.19 [-0.10, 2.48]		1.37 [1.02, 1.72]
ITT	[redacted]	SL	[redacted]	[redacted]	Unadjusted	4.76	3.96	0.81 [0.36, 1.25]	124	1.27 [1.11, 1.43]
			4.95	1,927.77	TMLE untruncated	4.79	4.44	0.35 [-0.25, 0.96]		[redacted]
					TMLE truncated at 200	4.74	4.44	0.30 [-0.30, 0.89]		
			5.35	795.44	IPW untruncated	4.61	4.41	0.19 [-0.64, 1.03]		1.06 [0.82, 1.29]
					IPW truncated at 20	4.67	4.46	0.21 [-0.63, 1.05]		1.06 [0.83, 1.29]
					IPW truncated at 99th	4.77	4.39	0.38 [-0.33, 1.09]		1.10 [0.89, 1.30]
		Logistic model	6.25	792.46	IPW untruncated	4.48	4.46	0.03 [-0.89, 0.94]		1.00 [0.75, 1.26]
					IPW truncated at 20	4.60	4.57	0.03 [-0.88, 0.94]		1.01 [0.76, 1.25]
					IPW truncated at 99th	4.67	4.45	0.23 [-0.54, 1.00]		1.07 [0.86, 1.27]

* Discontinuation refers to the interruption of the comparator medication initiated on index date; Crossover refers to the initiation of the comparator medication initiated by patient at baseline in the other arm.

© 2025 Neugebauer R et al. *JAMA Network Open*.

eFigure 67. MACE (Broader Definition), 2-Arm Drug Class, SGLT2is vs GLP-1RAs, Cumulative Incidence Curves From PP and ITT Analyses With IPW, TMLE, and SL

Each plot emulates inferences from a 2-arm RCT comparing SGLT2i and GLP-1RA and represents unadjusted or adjusted estimates of cumulative incidence curves for MACE (expanded) derived with inverse probability weighting (IPW) and Targeted Minimum Loss-based Estimation (TMLE) with Super Learning (SL) estimates of propensity scores with four weight truncation schemes: IPW and TMLE without weight truncation (untruncated), IPW with truncation of stabilized weights at value 20 (trunc20) or at the 99th percentile of weight values (trunc99), and TMLE with truncation of unstabilized weights at value 200 (trunc200). The red divider line separates results of Per-Protocol (PP) analyses (top half) from Intention-To-Treat (ITT) analyses (bottom half). Each plot displays a p value for the test that the average risk difference (ARD) through 2.5 years of follow-up (30 months) is 0.

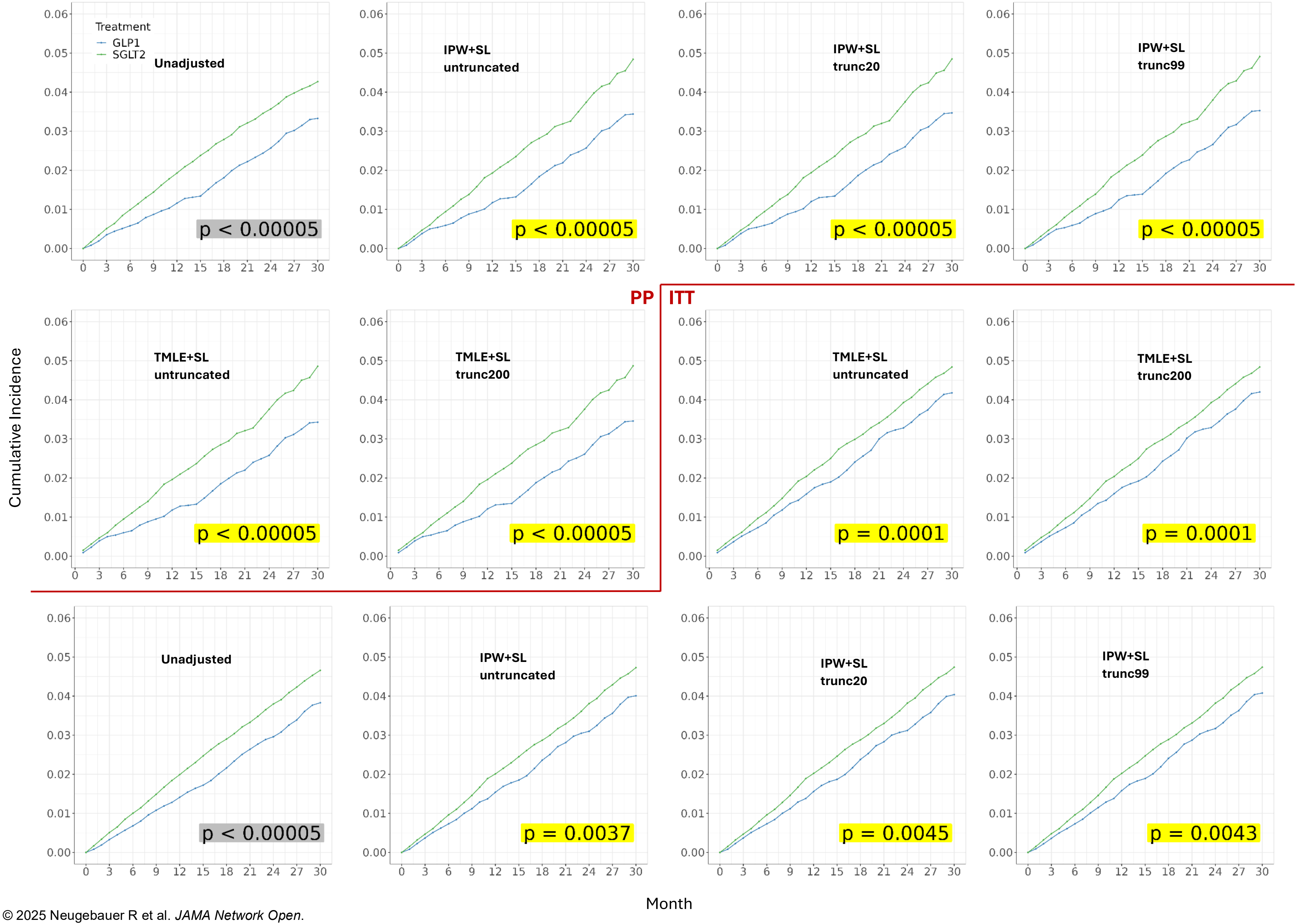

© 2025 Neugebauer R et al. *JAMA Network Open*.

eTable 66. MACE (Broader Definition), 2-Arm Drug Class, SGLT2is vs GLP-1RAs, RD and HR Effect Measures at 2.5 Years

Estimation results from ITT and PP analyses of emulated 2-arm RCTs comparing MACE (expanded) risks over 2.5 years between SGLT2i and GLP-1RA initiators. For PP analyses, rates of protocol deviations are described by medication class initiated at baseline. Unadjusted point and interval estimates and adjusted point and interval IPW and TMLE estimates of risks, risk differences (RD), and hazard ratios (HR) based on propensity scores (PS) estimated with either logistic models or super learning (SL) are presented for four weight truncation schemes along with the corresponding 99th percentile and maximum value of the stabilized and unstabilized inverse probability weights used for implementing IPW and TMLE, respectively. RD is the risk in treatment arm minus the risk in control arm and NNT is the number needed to treat.

Analysis type	Protocol Deviations* by exposure group (%)	PS estimation	99th IP weights	Max IP weight	Estimator	Treatment (SGLT2i) risk in %	Control (GLP-1RA) risk in %	RD [95% CI] in %	NNT	HR [95% CI]
PP	Discontinuation<br>SGLT2i: 25.58<br>GLP-1RA: 44.65<br><br>Crossover<br>SGLT2i: 5.01<br>GLP-1RA: 9.80	SL			Unadjusted	4.27	3.33	0.94 [0.31, 1.56]	107	1.70 [1.41, 1.99]
			17.10	1,035.11	TMLE untruncated	4.86	3.43	1.43 [1.01, 1.85]	70	
					TMLE truncated at 200	4.87	3.46	1.41 [0.99, 1.82]	71	
			3.23	176.35	IPW untruncated	4.84	3.44	1.40 [0.48, 2.33]	71	1.62 [1.24, 1.99]
					IPW truncated at 20	4.85	3.47	1.39 [0.46, 2.32]	72	1.61 [1.24, 1.98]
					IPW truncated at 99th	4.91	3.53	1.38 [0.44, 2.32]	72	1.61 [1.26, 1.95]
		Logistic model	4.73	653.42	IPW untruncated	5.42	3.79	1.63 [0.02, 3.24]	61	1.58 [1.19, 1.97]
					IPW truncated at 20	5.55	3.89	1.66 [0.01, 3.31]	60	1.56 [1.17, 1.95]
					IPW truncated at 99th	5.58	4.01	1.57 [0.11, 3.03]	64	1.54 [1.18, 1.90]
ITT		SL			Unadjusted	4.66	3.83	0.82 [0.36, 1.29]	121	1.42 [1.23, 1.62]
			10.64	656.25	TMLE untruncated	4.84	4.18	0.66 [0.32, 1.00]	152	
					TMLE truncated at 200	4.84	4.20	0.64 [0.30, 0.98]	156	
			2.99	134.84	IPW untruncated	4.73	4.01	0.71 [0.11, 1.32]	140	1.30 [1.05, 1.55]
					IPW truncated at 20	4.74	4.04	0.70 [0.09, 1.31]	142	1.30 [1.05, 1.55]
					IPW truncated at 99th	4.74	4.08	0.65 [0.06, 1.25]	153	1.29 [1.07, 1.52]
		Logistic model	4.27	675.03	IPW untruncated	4.76	4.22	0.54 [-0.29, 1.38]		1.25 [0.92, 1.58]
					IPW truncated at 20	4.76	4.27	0.49 [-0.31, 1.30]		1.24 [0.94, 1.53]
					IPW truncated at 99th	4.69	4.30	0.39 [-0.31, 1.08]		1.24 [1.01, 1.47]

* Discontinuation refers to the interruption of the comparator medication initiated on index date; Crossover refers to the initiation of the comparator medication initiated by patient at baseline in the other arm.

© 2025 Neugebauer R et al. *JAMA Network Open*.

eFigure 68. MACE (Primary Definition), Sulfonylureas vs DPP4is in ON TARGET DM and GRADE Trials, Cumulative Incidence Curves From PP Analyses With TMLE and SL for Emulating 2-Arm And 4-Arm Trials Compared to Cumulative Incidence Curves From the GRADE Trial

Comparison of results from the ON TARGET DM and GRADE studies that assessed the comparative effectiveness of SU versus DPP4i for preventing MACE. The top plot was produced using digitized data from Figure 3 Panel B of the following article from the GRADE study: *GRADE Study Research Group; Nathan DM, Lachin JM, Bebu I, Burch HB, Buse JB, Cherrington AL, Fortmann SP, Green JB, Kahn SE, Kirkman MS, Krause-Steinrauf H, Larkin ME, Phillips LS, Pop-Busui R, Steffes M, Tiktin M, Tripputi M, Wexler DJ, Younes N. Glycemia Reduction in Type 2 Diabetes - Microvascular and Cardiovascular Outcomes. N Engl J Med. 2022 Sep 22;387(12):1075-1088. doi: 10.1056/NEJMoa2200436. PMID: 36129997; PMCID: PMC9832916.* The bottom two plots display results from the ON TARGET DM PP analyses to emulate the 4-arm and 2-arm RCTs that compare MACE risks under sustained exposure to SU or DPP4i using TMLE and SL without weight truncation (untruncated).

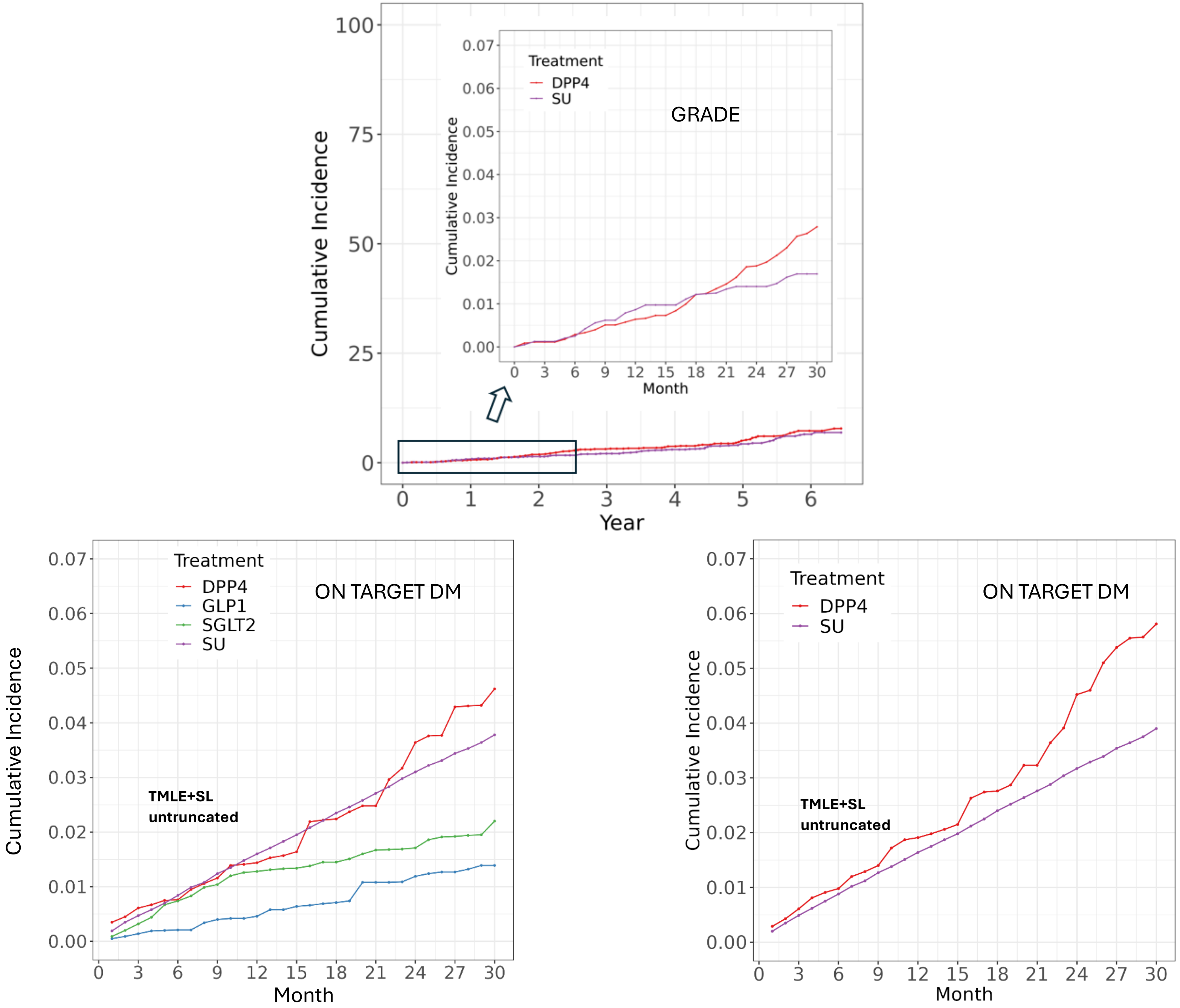

© 2025 Neugebauer R et al. *JAMA Network Open*.

eAppendix 4. MACE (Primary Definition), Sensitivity Analyses for Unmeasured Sources of Confounding and Selection Bias, Methodology Description

Inference from a given PP analysis might be invalid due to residual bias from unmeasured sources of confounding or selection bias. This residual bias can be referred to as the causal gap and denoted by δ.

If we knew the level of residual bias δ, we could adjust our inference by shifting the 95% confidence interval estimate obtained in the PP analysis by the actual value of the residual bias δ. The shifted confidence interval would allow us to conclude whether the inference we derived with available observed data would hold up or change had we been able to adjust for all sources (observed and unobserved) of confounding and selection bias.

The level of residual bias δ in our analysis is unknown, however, and it is not identifiable from the observed data.

Nevertheless, in a sensitivity analysis, we can *postulate* a range of plausible levels of residual biases δ to understand the robustness of findings from the PP analysis.

Subject-matter expertise may provide a plausible range for the causal gap δ. In the absence of this expertise, we can replicate the original PP analysis after excluding a subset of observed covariates from the adjustment set to derive one plausible level of bias denoted by $\hat{\delta}$ (i.e., the difference between the effect estimate resulting from artificially ignoring known confounders in the PP analysis and the original fully adjusted PP analysis estimate). The confidence intervals from the original PP analyses can then be shifted by (subtracting) $\hat{\delta}$ to understand whether our inference would change or be upheld if we could adjust for a plausible level of bias from unmeasured confounding or selection bias. To complement our understanding of the robustness of findings to unmeasured sources of confounding and selection bias, we can also compute the factor by which the value of the bias level $\hat{\delta}$ would need to be multiplied such that the resulting inference would change it from statistically significant to non-significant. This factor is referred to as the g-value.

We adopted the approach above by running the same six primary PP analyses for emulating 2-arm RCTs using TMLE and SL without weight truncation after excluding the following covariates assumed to be partial surrogates for unobserved health seeking behaviors, diet, or exercise in the original PP analyses: smoking_status, dietitian, ins_medicaid, and bmi.

Results from these six sensitivity analyses are displayed in the following Figures. They suggest that study findings are robust to plausible levels of unmeasured sources of confounding and selection bias.

© 2025 Neugebauer G et al. *JAMA Network Open*.

eFigure 69. MACE (Primary Definition), Sensitivity Analyses for Unmeasured Sources of Confounding and Selection Bias, All Pairwise Comparisons From 2-Arm Cohorts, Sulfonylurea vs DPP4i Results

Sensitivity analysis to evaluate the robustness to unmeasured sources of confounding and selection bias of the ON TARGET DM conclusion about the effect of sustained use of SU versus DPP4i on the 2.5-year MACE risk derived from the PP analysis with TMLE and SL (untruncated) using the 2-arm cohort. The top panel displays the shift in the point and 95% confidence interval estimates (y axis) with various levels of presumed causal bias δ (x axis). The point and confidence interval estimates highlighted in yellow indicate a plausible *corrected* effect estimate resulting from the adjustment for unmeasured covariates whose impact on inference would be the same as the impact on inference resulting from ignoring the following observed covariates: smoking_status, dietitian, ins_medicaid, and bmi. The bottom panel displays the risk difference point and interval estimates from the original PP analysis (first row), the analog estimates derived from the artificial analysis where the aforementioned observed covariates are ignored (second row), the resulting bias $\hat{\delta}$ introduced by ignoring these observed covariates (row 3, i.e., difference between row 2 and 1), and the corrected inference after shifting the original PP inference by $\hat{\delta}$. **This sensitivity analysis suggests that adjustment for a plausible level of unmeasured confounding or selection bias would result in strengthening the study evidence about the comparative effectiveness of SU and DPP4i obtained based on observed covariates**. The g-value that would result in a non-significant finding is -33.79.

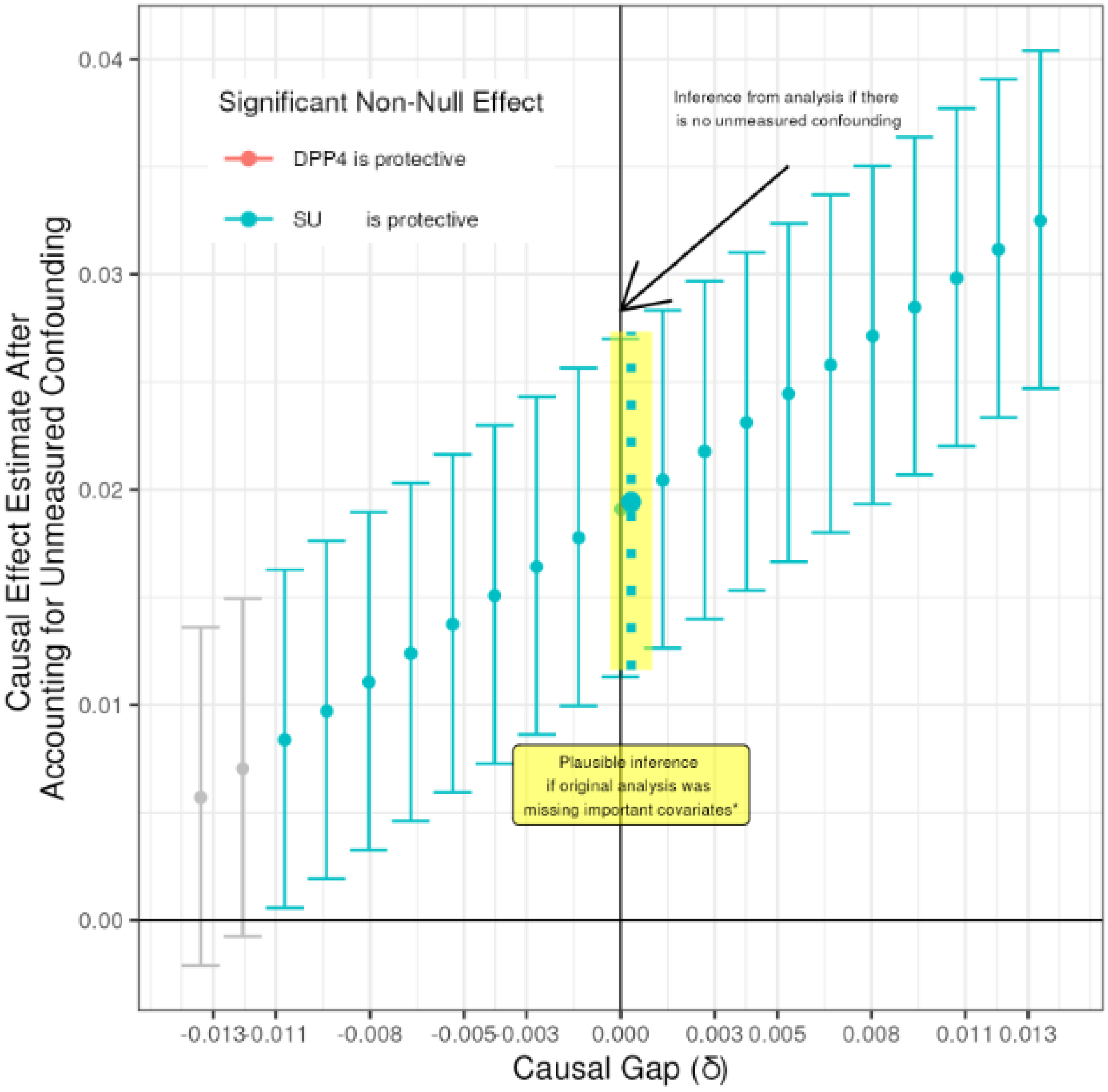

Estimate Type	RD	CIlow	CIhigh
Fully Adjusted	0.0191000	0.0113000	0.0270000
Artificial Unmeasured Confounding	0.0187655	0.0110034	0.0265277
Delta	-0.0003345		
Corrected Causal Effect	0.0194345	0.0116345	0.0273345

* RD, CIlow, and CIhigh stand for the risk difference (DPP4i risk minus SU risk), lower and upper bound of the 95% confidence interval, respectively.

© 2025 Neugebauer R et al. *JAMA Network Open*.

eFigure 70. MACE (Primary Definition), Sensitivity Analyses for Unmeasured Sources of Confounding and Selection Bias, All Pairwise Comparisons From 2-Arm Cohorts, Sulfonylurea vs SGLT2i Results

Sensitivity analysis to evaluate the robustness to unmeasured sources of confounding and selection bias of the ON TARGET DM conclusion about the effect of sustained use of SU versus SGLT2i on the 2.5-year MACE risk derived from the PP analysis with TMLE and SL (untruncated) using the 2-arm cohort. The top panel displays the shift in the point and 95% confidence interval estimates (y axis) with various levels of presumed causal bias δ (x axis). The point and confidence interval estimates highlighted in yellow indicate a plausible *corrected* effect estimate resulting from the adjustment for unmeasured covariates whose impact on inference would be the same as the impact on inference resulting from ignoring the following observed covariates: smoking_status, dietitian, ins_medicaid, and bmi. The bottom panel displays the risk difference point and interval estimates from the original PP analysis (first row), the analog estimates derived from the artificial analysis where the aforementioned observed covariates are ignored (second row), the resulting bias $\hat{\delta}$ introduced by ignoring these observed covariates (row 3, i.e., difference between row 2 and 1), and the corrected inference after shifting the original PP inference by $\hat{\delta}$. **This sensitivity analysis suggests that adjustment for a plausible level of unmeasured confounding or selection bias would result in slightly weakening the study evidence about the comparative effectiveness of SU and SGLT2i obtained based on observed covariates**. The g-value that would result in a non-significant finding is 13.6.

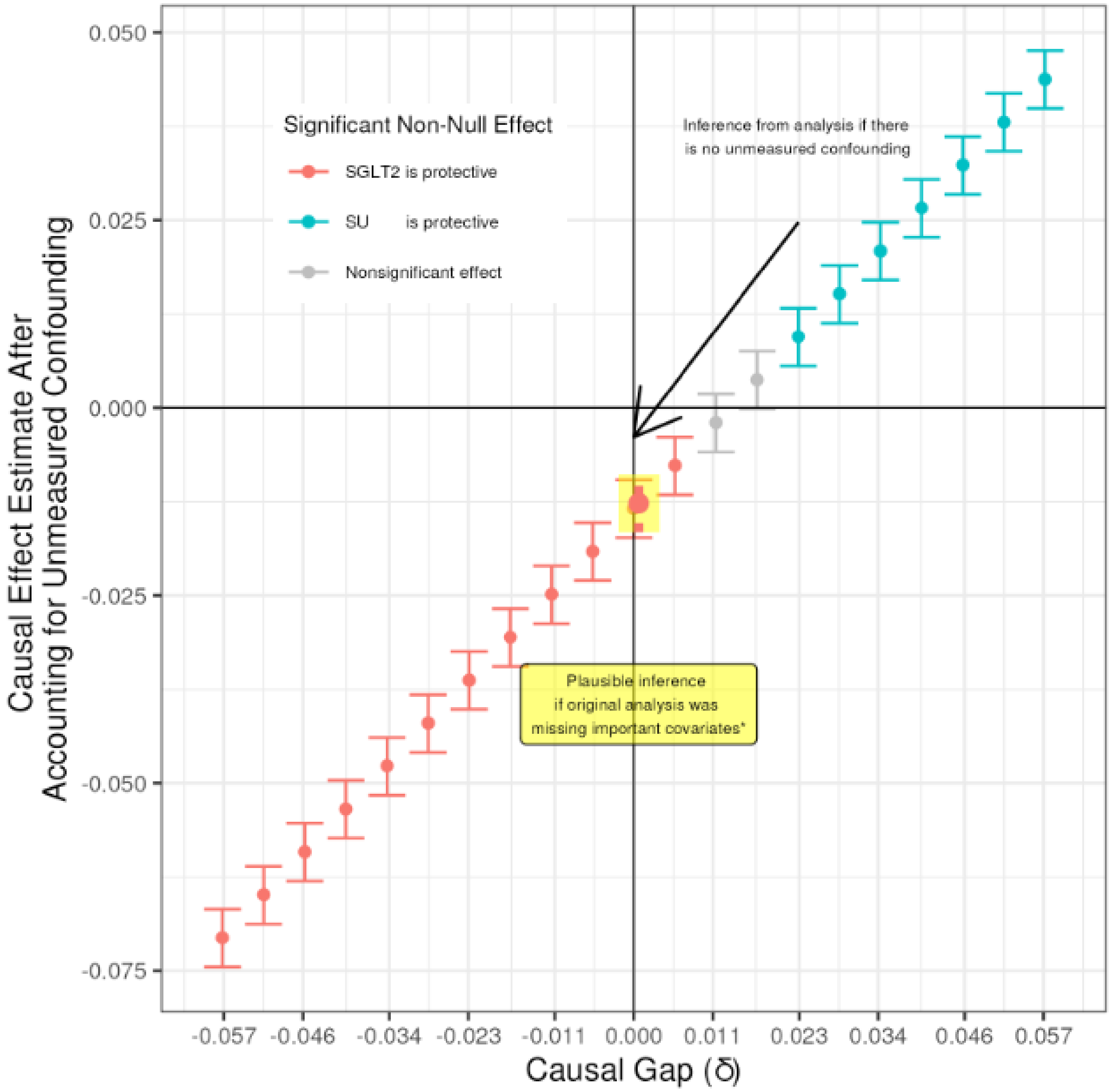

Estimate Type	RD	CIlow	CIhigh
Fully Adjusted	-0.013400	-0.0173000	-0.0096000
Artificial Unmeasured Confounding	-0.014106	-0.0178329	-0.0103792
Delta	-0.000706		
Corrected Causal Effect	-0.012694	-0.0165940	-0.0088940

* RD, CIlow, and CIhigh stand for the risk difference (SGLT2i risk minus SU risk), lower and upper bound of the 95% confidence interval, respectively.

© 2025 Neugebauer R et al. *JAMA Network Open*.

eFigure 71. MACE (Primary Definition), Sensitivity Analyses for Unmeasured Sources of Confounding and Selection Bias, All Pairwise Comparisons From 2-Arm Cohorts, Sulfonylurea vs GLP-1RA Results

Sensitivity analysis to evaluate the robustness to unmeasured sources of confounding and selection bias of the ON TARGET DM conclusion about the effect of sustained use of SU versus GLP-1RA on the 2.5-year MACE risk derived from the PP analysis with TMLE and SL (untruncated) using the 2-arm cohort. The top panel displays the shift in the point and 95% confidence interval estimates (y axis) with various levels of presumed causal bias δ (x axis). The point and confidence interval estimates highlighted in yellow indicate a plausible *corrected* effect estimate resulting from the adjustment for unmeasured covariates whose impact on inference would be the same as the impact on inference resulting from ignoring the following observed covariates: smoking_status, dietitian, ins_medicaid, and bmi. The bottom panel displays the risk difference point and interval estimates from the original PP analysis (first row), the analog estimates derived from the artificial analysis where the aforementioned observed covariates are ignored (second row), the resulting bias $\hat{\delta}$ introduced by ignoring these observed covariates (row 3, i.e., difference between row 2 and 1), and the corrected inference after shifting the original PP inference by $\hat{\delta}$. **This sensitivity analysis suggests that adjustment for a plausible level of unmeasured confounding or selection bias would result in slightly weakening the study evidence about the comparative effectiveness of SU and GLP-1RA obtained based on observed covariates**. The g-value that would result in a non-significant finding is 42.72.

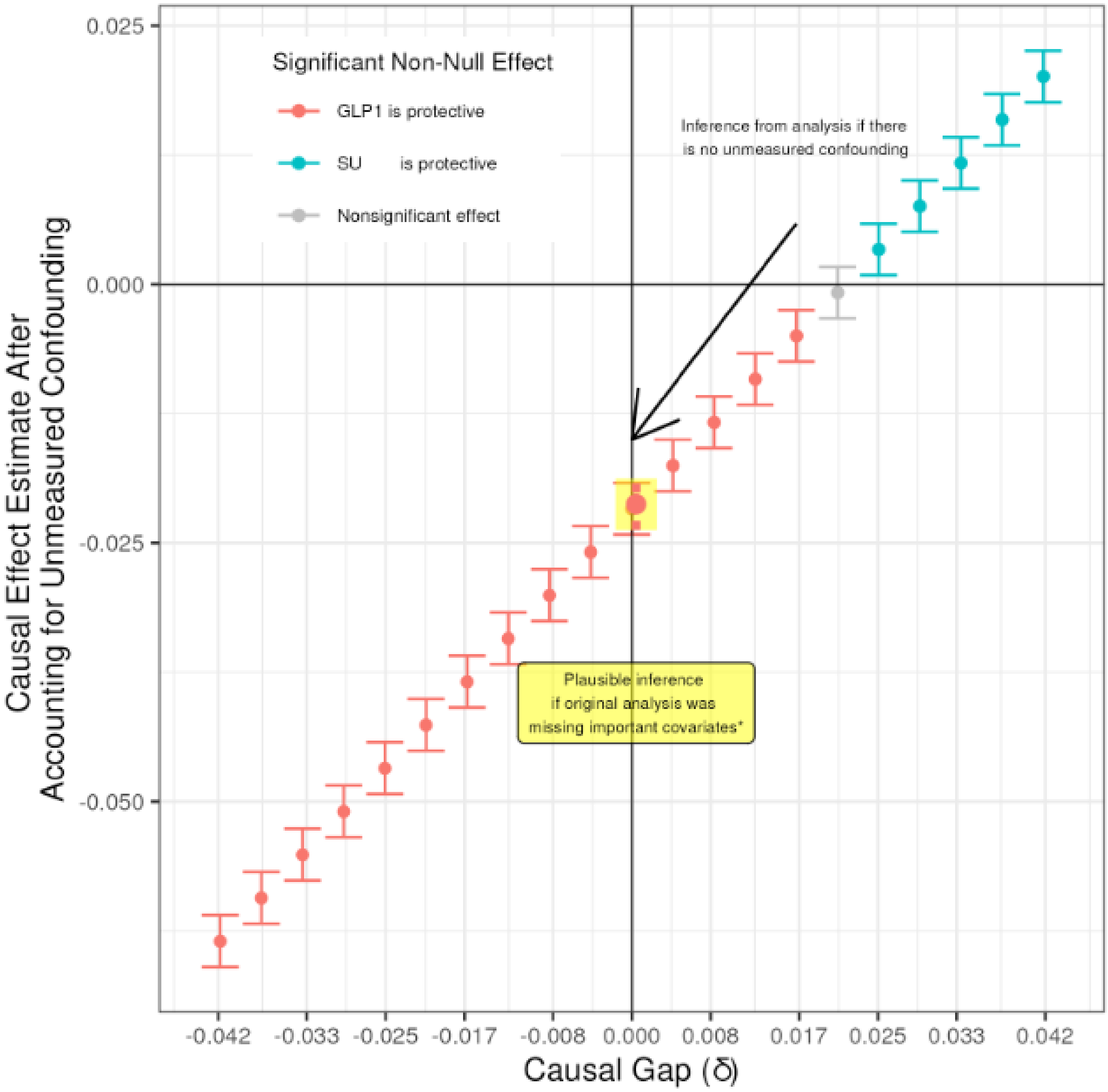

Estimate Type	RD	CIlow	CIhigh
Fully Adjusted	-0.0217000	-0.0242000	-0.0192000
Artificial Unmeasured Confounding	-0.0221494	-0.0246165	-0.0196823
Delta	-0.0004494		
Corrected Causal Effect	-0.0212506	-0.0237506	-0.0187506

* RD, CIlow, and CIhigh stand for the risk difference (GLP-1RA risk minus SU risk), lower and upper bound of the 95% confidence interval, respectively.

© 2025 Neugebauer R et al. *JAMA Network Open*.

eFigure 72. MACE (Primary Definition), Sensitivity Analyses for Unmeasured Sources of Confounding and Selection Bias, All Pairwise Comparisons From 2-Arm Cohorts, DPP4i vs SGLT2i Results

Sensitivity analysis to evaluate the robustness to unmeasured sources of confounding and selection bias of the ON TARGET DM conclusion about the effect of sustained use of DPP4i versus SGLT2i on the 2.5-year MACE risk derived from the PP analysis with TMLE and SL (untruncated) using the 2-arm cohort. The top panel displays the shift in the point and 95% confidence interval estimates (y axis) with various levels of presumed causal bias δ (x axis). The point and confidence interval estimates highlighted in yellow indicate a plausible *corrected* effect estimate resulting from the adjustment for unmeasured covariates whose impact on inference would be the same as the impact on inference resulting from ignoring the following observed covariates: smoking_status, dietitian, ins_medicaid, and bmi. The bottom panel displays the risk difference point and interval estimates from the original PP analysis (first row), the analog estimates derived from the artificial analysis where the aforementioned observed covariates are ignored (second row), the resulting bias $\hat{\delta}$ introduced by ignoring these observed covariates (row 3, i.e., difference between row 2 and 1), and the corrected inference after shifting the original PP inference by $\hat{\delta}$. **This sensitivity analysis suggests that adjustment for a plausible level of unmeasured confounding or selection bias would result in slightly weakening the study evidence about the comparative effectiveness of DPP4i and SGLT2i obtained based on observed covariates**. The g-value that would result in a non-significant finding is 86.89.

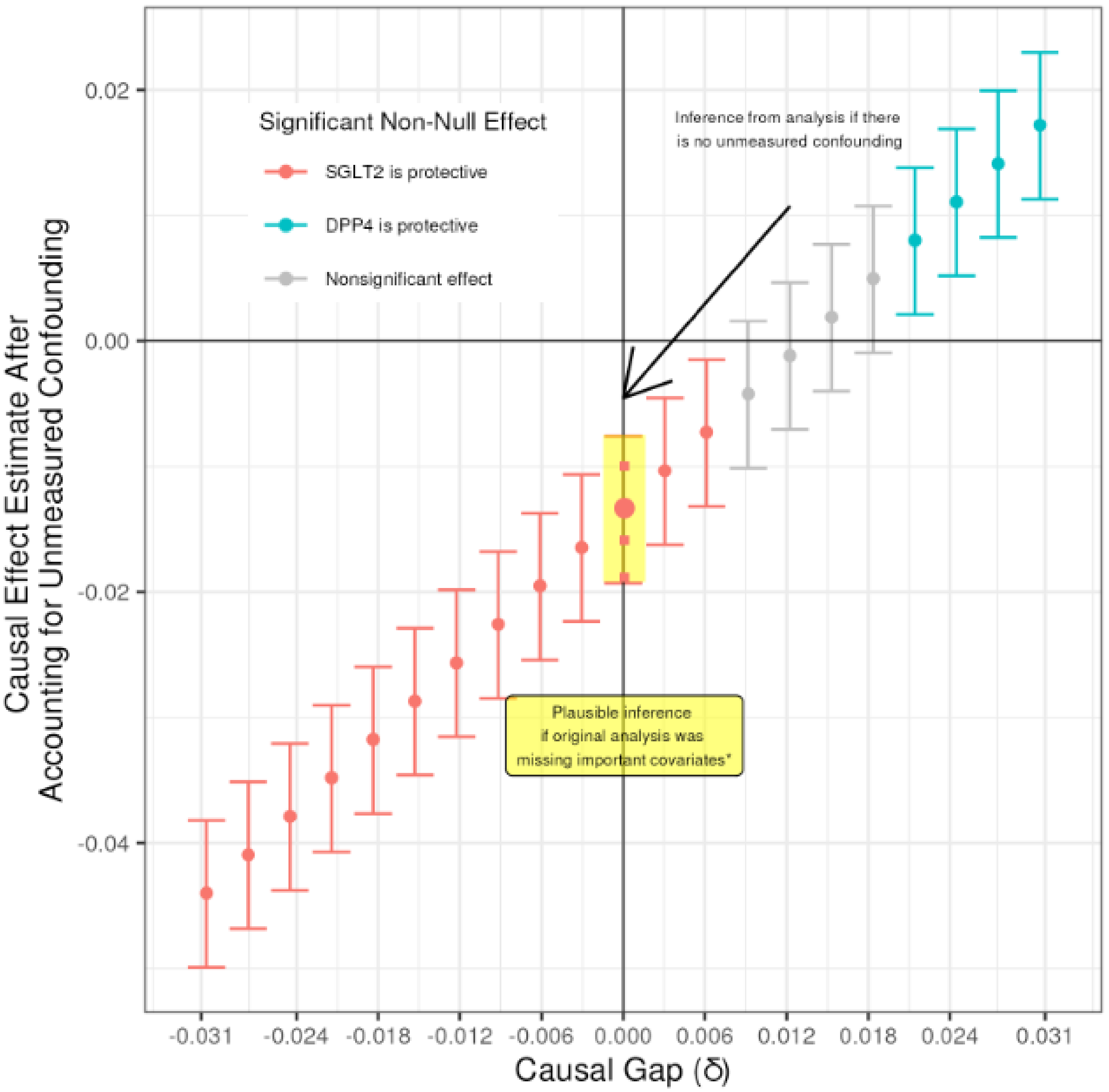

Estimate Type	RD	CIlow	CIhigh
Fully Adjusted	-0.0134000	-0.0193000	-0.0076000
Artificial Unmeasured Confounding	-0.0134875	-0.0193619	-0.0076130
Delta	-0.0000875		[illegible]
Corrected Causal Effect	-0.0133125	-0.0192125	-0.0075125

* RD, CIlow, and CIhigh stand for the risk difference (SGLT2i risk minus DPP4i risk), lower and upper bound of the 95% confidence interval, respectively.

© 2025 Neugebauer R et al. *JAMA Network Open*.

eFigure 73. MACE (Primary Definition), Sensitivity Analyses for Unmeasured Sources of Confounding and Selection Bias, All Pairwise Comparisons From 2-Arm Cohorts, DPP4i vs GLP-1RA Results

Sensitivity analysis to evaluate the robustness to unmeasured sources of confounding and selection bias of the ON TARGET DM conclusion about the effect of sustained use of DPP4i versus GLP-1RA on the 2.5-year MACE risk derived from the PP analysis with TMLE and SL (untruncated) using the 2-arm cohort. The top panel displays the shift in the point and 95% confidence interval estimates (y axis) with various levels of presumed causal bias δ (x axis). The point and confidence interval estimates highlighted in yellow indicate a plausible *corrected* effect estimate resulting from the adjustment for unmeasured covariates whose impact on inference would be the same as the impact on inference resulting from ignoring the following observed covariates: smoking_status, dietitian, ins_medicaid, and bmi. The bottom panel displays the risk difference point and interval estimates from the original PP analysis (first row), the analog estimates derived from the artificial analysis where the aforementioned observed covariates are ignored (second row), the resulting bias $\hat{\delta}$ introduced by ignoring these observed covariates (row 3, i.e., difference between row 2 and 1), and the corrected inference after shifting the original PP inference by $\hat{\delta}$. **This sensitivity analysis suggests that adjustment for a plausible level of unmeasured confounding or selection bias would result in strengthening the study evidence about the comparative effectiveness of DPP4i and GLP-1RA obtained based on observed covariates**. The g-value that would result in a non-significant finding is -1.24.

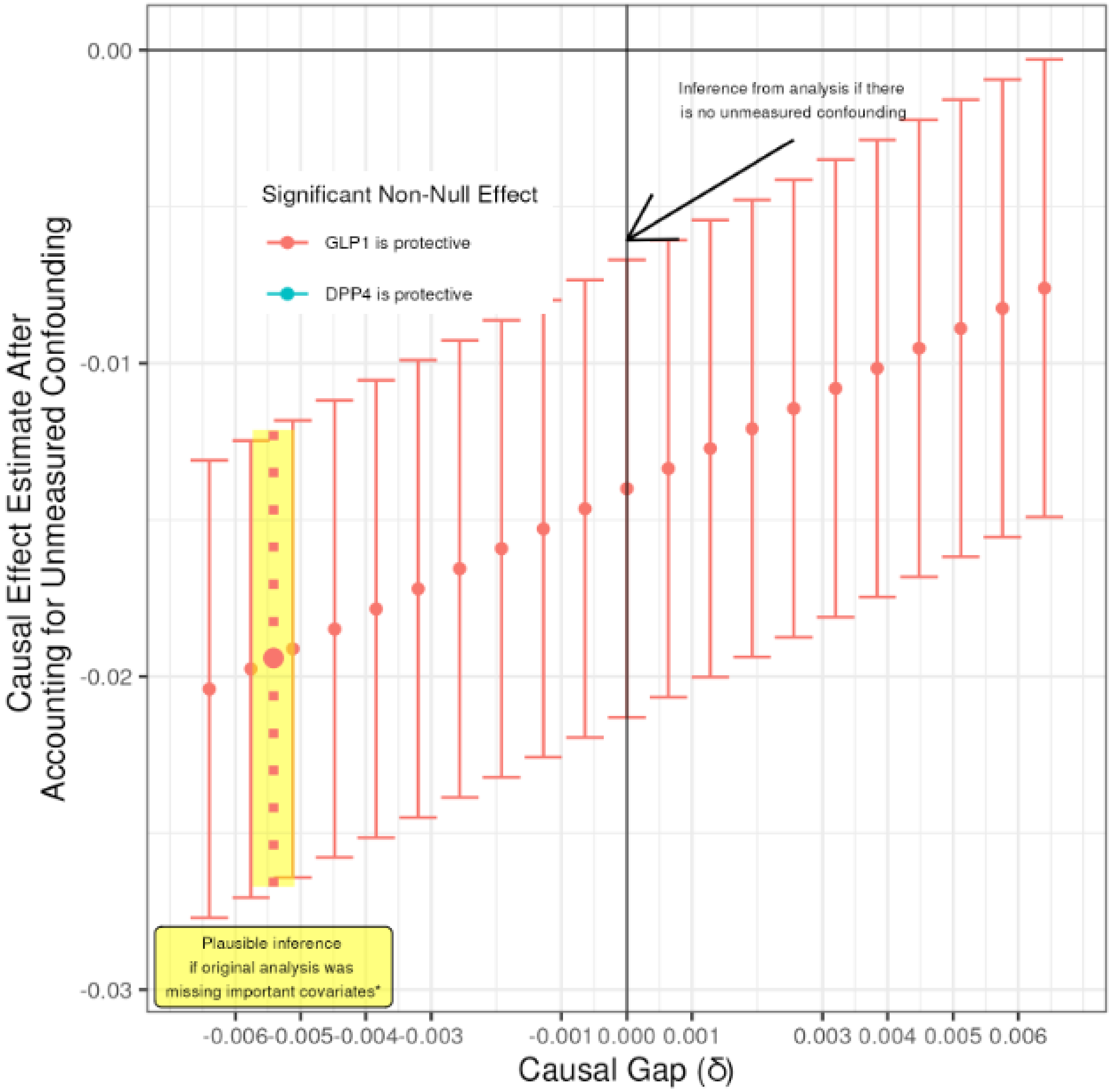

Estimate Type	RD	CIlow	CIhigh
Fully Adjusted	-0.0140000	-0.0213000	-0.0067000
Artificial Unmeasured Confounding	-0.0085865	-0.0191310	0.0019580
Delta	0.0054135		
Corrected Causal Effect	-0.0194135	-0.0267135	-0.0121135

* RD, CIlow, and CIhigh stand for the risk difference (GLP-1RA risk minus DPP4i risk), lower and upper bound of the 95% confidence interval, respectively.

© 2025 Neugebauer R et al. *JAMA Network Open*.

eFigure 74. MACE (Primary Definition), Sensitivity Analyses for Unmeasured Sources of Confounding and Selection Bias, All Pairwise Comparisons From 2-Arm Cohorts, SGLT2i vs GLP-1RA Results

Sensitivity analysis to evaluate the robustness to unmeasured sources of confounding and selection bias of the ON TARGET DM conclusion about the effect of sustained use of SGLT2i versus GLP-1RA on the 2.5-year MACE risk derived from the PP analysis with TMLE and SL (untruncated) using the 2-arm cohort. The top panel displays the shift in the point and 95% confidence interval estimates (y axis) with various levels of presumed causal bias δ (x axis). The point and confidence interval estimates highlighted in yellow indicate a plausible *corrected* effect estimate resulting from the adjustment for unmeasured covariates whose impact on inference would be the same as the impact on inference resulting from ignoring the following observed covariates: smoking_status, dietitian, ins_medicaid, and bmi. The bottom panel displays the risk difference point and interval estimates from the original PP analysis (first row), the analog estimates derived from the artificial analysis where the aforementioned observed covariates are ignored (second row), the resulting bias $\hat{\delta}$ introduced by ignoring these observed covariates (row 3, i.e., difference between row 2 and 1), and the corrected inference after shifting the original PP inference by $\hat{\delta}$. **This sensitivity analysis suggests that adjustment for a plausible level of unmeasured confounding or selection bias would result in strengthening the study evidence about the comparative effectiveness of SGLT2i and GLP-1RA obtained based on observed covariates**. The g-value that would result in a non-significant finding is -23.17.

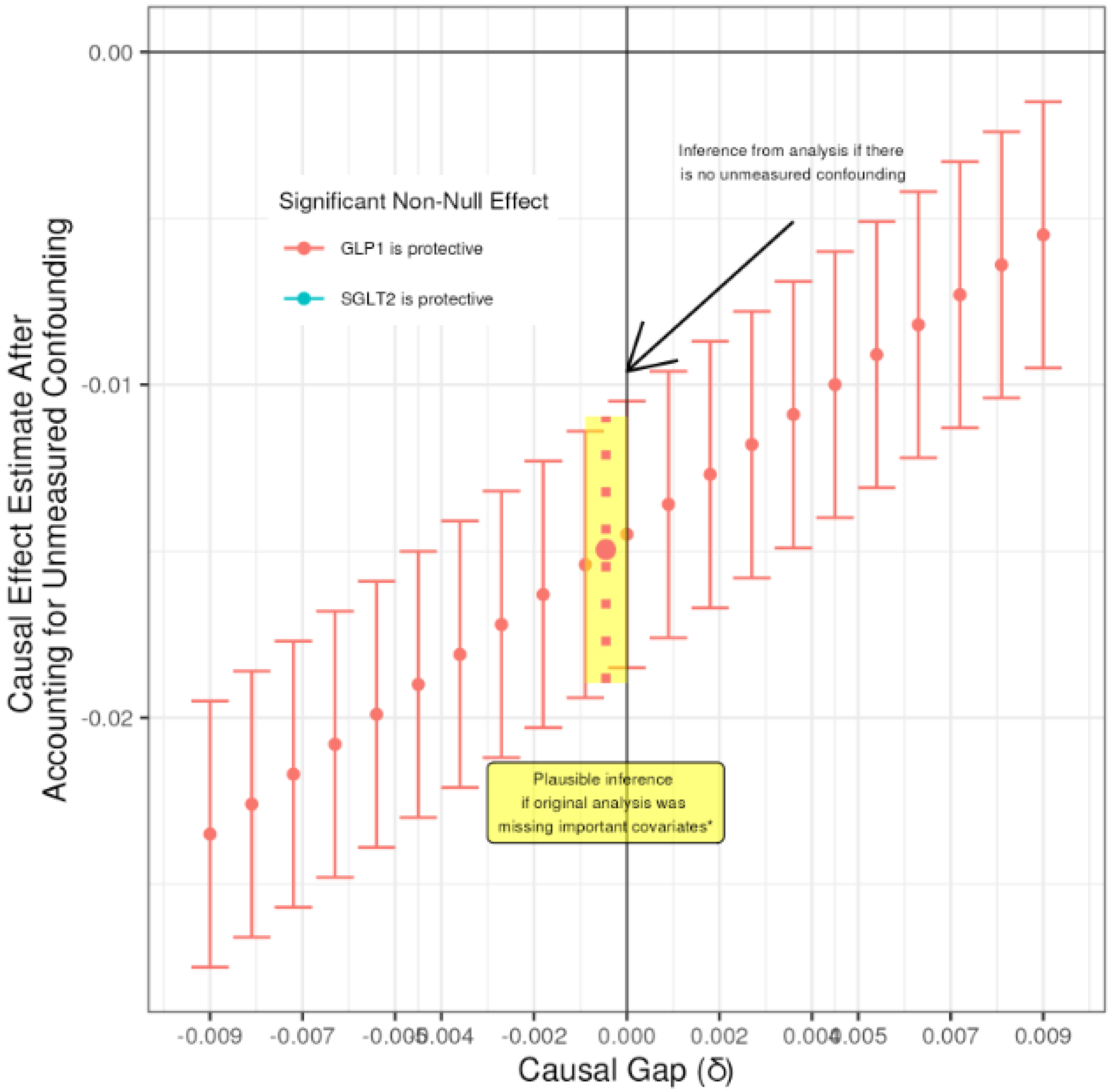

Estimate Type	RD	CIlow	CIhigh
Fully Adjusted	-0.0145000	-0.0185000	-0.0105000
Artificial Unmeasured Confounding	-0.0140469	-0.0181714	-0.0099224
Delta	0.0004531		
Corrected Causal Effect	-0.0149531	-0.0189531	-0.0109531

* RD, CIlow, and CIhigh stand for the risk difference (GLP-1RA risk minus SGLT2i risk), lower and upper bound of the 95% confidence interval, respectively.

© 2025 Neugebauer R et al. *JAMA Network Open*.

eReferences

1. Neugebauer R, Pimentel N, Hejazi N. LtAtStructuR R package. Accessed Nov 19, 2021. https://github.com/romainkp/LtAtStructuR
2. Stuart EA. Matching methods for causal inference: A review and a look forward. *Stat Sci*. Feb 1 2010;25(1):1-21. doi:10.1214/09-sts313
3. Hernán MA, McAdams M, McGrath N, Lanoy E, Costagliola D. Observation plans in longitudinal studies with time-varying treatments. *Stat Methods Med Res*. Feb 2009;18(1):27-52. doi:10.1177/0962280208092345
4. Kreif N, Sofrygin O, Schmittdiel JA, et al. Evaluation of adaptive treatment strategies in an observational study where time-varying covariates are not monitored systematically. *arXiv*. 2018;doi:10.48550/arXiv.1806.11153
5. Blake HA, Leyrat C, Mansfield KE, Tomlinson LA, Carpenter J, Williamson EJ. Estimating treatment effects with partially observed covariates using outcome regression with missing indicators. *Biom J*. Mar 2020;62(2):428-443. doi:10.1002/bimj.201900041
6. Gruber S, Lee H, Phillips R, Ho M, van der Laan M. Developing a Targeted Learning-Based Statistical Analysis Plan. *Statistics in Biopharmaceutical Research*. 2023/07/03 2023;15(3):468-475. doi:10.1080/19466315.2022.2116104
7. Robins J. A new approach to causal inference in mortality studies with a sustained exposure period—application to control of the healthy worker survivor effect. *Mathematical modelling*. 1986;7(9):1393-1512.
8. Hernán MA, Hernández-Díaz S. Beyond the intention-to-treat in comparative effectiveness research. *Clin Trials*. Feb 2012;9(1):48-55. doi:10.1177/1740774511420743
9. Robins J. Marginal Structural Models. *1997 Proceedings of the American Statistical Association*. 1998:1-10:chap Section on Bayesian Statistical Science.
10. van der Laan MJ, Rose S. *Targeted Learning in Data Science: Causal Inference for Complex Longitudinal Studies*. Springer Nature; 2018. https://link.springer.com/book/10.1007/978-3-319-65304-4.
11. Uno H, Claggett B, Tian L, et al. Moving beyond the hazard ratio in quantifying the between-group difference in survival analysis. *J Clin Oncol*. Aug 1 2014;32(22):2380-5. doi:10.1200/jco.2014.55.2208
12. Eaton A, Therneau T, Le-Rademacher J. Designing clinical trials with (restricted) mean survival time endpoint: Practical considerations. *Clin Trials*. Jun 2020;17(3):285-294. doi:10.1177/1740774520905563
13. van der Laan MJ, Rubin D. Targeted Maximum Likelihood Learning. *The International Journal of Biostatistics*. 2006;2(1)doi:doi:10.2202/1557-4679.1043
14. van der Laan MJ, Gruber S. Targeted minimum loss based estimation of causal effects of multiple time point interventions. *Int J Biostat*. 2012;8(1)doi:10.1515/1557-4679.1370
15. Neugebauer R, Schmittdiel JA, van der Laan MJ. Targeted learning in real-world comparative effectiveness research with time-varying interventions. *Stat Med*. Jun 30 2014;33(14):2480-520. doi:10.1002/sim.6099
16. Robins JM. Association, causation, and marginal structural models. *Synthese*. 1999;121(1-2):151-179.
17. van der Laan M, Robins J. *Unified Methods for Censored Longitudinal Data and Causality*. Springer; 2003.
18. de Leeuw J, Hornik K, Mair P. Isotone Optimization in R: Pool-Adjacent-Violators Algorithm (PAVA) and Active Set Methods. *Journal of Statistical Software*. 10/21 2009;32(5):1 - 24. doi:10.18637/jss.v032.i05
19. Westling T, van der Laan MJ, Carone M. Correcting an estimator of a multivariate monotone function with isotonic regression. *Electron J Stat*. 2020;14(2):3032-3069. doi:10.1214/20-ejs1740
20. Tibshirani R. Regression Shrinkage and Selection Via the Lasso. *Journal of the Royal Statistical Society: Series B (Methodological)*. 2018;58(1):267-288. doi:10.1111/j.2517-6161.1996.tb02080.x
21. Hastie T, Tibshirani R, Friedman J. *The elements of statistical learning*. 2nd ed. Springer; 2009.
22. van der Laan MJ, Polley EC, Hubbard AE. Super learner. *Stat Appl Genet Mol Biol*. 2007;6:Article25. doi:10.2202/1544-6115.1309
23. Coyle J, Hejazi N, Malenica I, Phillips R, Sofrygin O. sl3: Pipelines for Machine Learning and Super Learning. doi:10.5281/zenodo.1342293, R package version 1.4.5 https://github.com/tlverse/sl3
24. van der Vaart A. *Asymptotic statistics*. Cambridge series in statistical and probabilistic mathematics. Cambridge University Press; 1998.
25. Neugebauer R, Schmittdiel JA, van der Laan MJ. A Case Study of the Impact of Data-Adaptive Versus Model-Based Estimation of the Propensity Scores on Causal Inferences from Three Inverse Probability Weighting Estimators. *Int J Biostat*. May 1 2016;12(1):131-55. doi:10.1515/ijb-2015-0028
26. Neugebauer R, Laan M. Nonparametric causal effects based on marginal structural models. *Journal of Statistical Planning and Inference*. 02/01 2007;137:419-434. doi:10.1016/j.jspi.2005.12.008
27. Cole SR, Hernán MA. Constructing inverse probability weights for marginal structural models. *Am J Epidemiol*. Sep 15 2008;168(6):656-64. doi:10.1093/aje/kwn164
28. Petersen ML, Porter KE, Gruber S, Wang Y, van der Laan MJ. Diagnosing and responding to violations in the positivity assumption. *Stat Methods Med Res*. Feb 2012;21(1):31-54. doi:10.1177/0962280210386207

© 2025 Neugebauer R et al. *JAMA Network Open*.

29. Gruber S, Phillips RV, Lee H, Ho M, Concato J, van der Laan MJ. Targeted Learning: Toward a Future Informed by Real-World Evidence. *Statistics in Biopharmaceutical Research*. 2022;16:11 - 25.
30. Sofrygin O, van der Laan M, Neugebauer R. stremr R package: Streamlined Causal Inference for Static, Dynamic and Stochastic Regimes in Longitudinal Data. 2016. https://github.com/romainkp/stremr
31. Ueda P, Wintzell V, Dahlqwist E, et al. The comparative cardiovascular and renal effectiveness of sodium-glucose co-transporter-2 inhibitors and glucagon-like peptide-1 receptor agonists: A Scandinavian cohort study. *Diabetes Obes Metab*. Mar 2022;24(3):473-485. doi:10.1111/dom.14598
32. D'Andrea E, Wexler DJ, Kim SC, Paik JM, Alt E, Patorno E. Comparing Effectiveness and Safety of SGLT2 Inhibitors vs DPP-4 Inhibitors in Patients With Type 2 Diabetes and Varying Baseline HbA1c Levels. *JAMA Intern Med*. Mar 1 2023;183(3):242-254. doi:10.1001/jamainternmed.2022.6664
33. Edmonston D, Mulder H, Lydon E, et al. Kidney and Cardiovascular Effectiveness of SGLT2 Inhibitors vs GLP-1 Receptor Agonists in Type 2 Diabetes. *J Am Coll Cardiol*. Aug 20 2024;84(8):696-708. doi:10.1016/j.jacc.2024.06.016
34. Fu EL, Clase CM, Janse RJ, et al. Comparative effectiveness of SGLT2i versus GLP1-RA on cardiovascular outcomes in routine clinical practice. *Int J Cardiol*. Apr 1 2022;352:172-179. doi:10.1016/j.ijcard.2022.01.042
35. Htoo PT, Tesfaye H, Schneeweiss S, et al. Comparative Effectiveness of Empagliflozin vs Liraglutide or Sitagliptin in Older Adults With Diverse Patient Characteristics. *JAMA Netw Open*. Oct 3 2022;5(10):e2237606. doi:10.1001/jamanetworkopen.2022.37606
36. Kutz A, Kim DH, Wexler DJ, et al. Comparative Cardiovascular Effectiveness and Safety of SGLT-2 Inhibitors, GLP-1 Receptor Agonists, and DPP-4 Inhibitors According to Frailty in Type 2 Diabetes. *Diabetes Care*. Nov 1 2023;46(11):2004-2014. doi:10.2337/dc23-0671
37. McCoy RG, Herrin J, Swarna KS, et al. Effectiveness of glucose-lowering medications on cardiovascular outcomes in patients with type 2 diabetes at moderate cardiovascular risk. *Nat Cardiovasc Res*. Apr 2024;3(4):431-440. doi:10.1038/s44161-024-00453-9
38. Xie Y, Bowe B, Xian H, Loux T, McGill JB, Al-Aly Z. Comparative effectiveness of SGLT2 inhibitors, GLP-1 receptor agonists, DPP-4 inhibitors, and sulfonylureas on risk of major adverse cardiovascular events: emulation of a randomised target trial using electronic health records. *Lancet Diabetes Endocrinol*. Sep 2023;11(9):644-656. doi:10.1016/s2213-8587(23)00171-7
39. Do D, Lee T, Peasah SK, Good CB, Inneh A, Patel U. GLP-1 Receptor Agonist Discontinuation Among Patients With Obesity and/or Type 2 Diabetes. *JAMA Netw Open*. May 1 2024;7(5):e2413172. doi:10.1001/jamanetworkopen.2024.13172
40. Rodriguez PJ, Zhang V, Gratzl S, et al. Discontinuation and Reinitiation of Dual-Labeled GLP-1 Receptor Agonists Among US Adults With Overweight or Obesity. *JAMA Netw Open*. Jan 2 2025;8(1):e2457349. doi:10.1001/jamanetworkopen.2024.57349
41. Neugebauer R, Fireman B, Roy JA, Raebel MA, Nichols GA, O'Connor PJ. Super learning to hedge against incorrect inference from arbitrary parametric assumptions in marginal structural modeling. *J Clin Epidemiol*. Aug 2013;66(8 Suppl):S99-109. doi:10.1016/j.jclinepi.2013.01.016
42. Pirracchio R, Petersen ML, van der Laan M. Improving propensity score estimators' robustness to model misspecification using super learner. *Am J Epidemiol*. Jan 15 2015;181(2):108-19. doi:10.1093/aje/kwu253
43. Salditt M, Nestler S. Parametric and nonparametric propensity score estimation in multilevel observational studies. *Stat Med*. Oct 15 2023;42(23):4147-4176. doi:10.1002/sim.9852
44. Neugebauer R, Schmittdiel JA, Zhu Z, Rassen JA, Seeger JD, Schneeweiss S. High-dimensional propensity score algorithm in comparative effectiveness research with time-varying interventions. *Stat Med*. Feb 28 2015;34(5):753-81. doi:10.1002/sim.6377

© 2025 Neugebauer R et al. *JAMA Network Open*.
