# Supplementary material for: Glucose-Lowering Medication Classes and Cardiovascular Outcomes in Patients With Type 2 Diabetes
Source: JAMA Netw Open. 2025 Oct 15;8(10):e2536100. doi: 10.1001/jamanetworkopen.2025.36100 (PMC12529185; doi:10.1001/jamanetworkopen.2025.36100)
Supplement: Supplement 2. — Data Sharing Statement [file jamanetwopen-e2536100-s002.pdf]

Data Sharing Statement

Neugebauer. Glucose-Lowering Medication Classes and Cardiovascular Outcomes in Patients With Type 2 Diabetes. *JAMA Netw Open*. Published October 10, 2025.
doi:10.1001/jamanetworkopen.2025.36100

Data
Data available: Yes
Data types: Deidentified participant data, Data dictionary
How to access data: PCORI repository
When available: beginning date: 07-01-2026

Supporting Documents
Document types: None

Additional Information
Who can access the data: Researchers whose proposed use of the data was approved by PCORI's repository.
Types of analyses: For the approved analysis plan.
Mechanisms of data availability: After approval of a proposal
